# Genome-wide identification and expression analysis of MADS-box transcription factors reveal their involvement in sex determination of hardy rubber tree (*Eucommia ulmoides* oliv.)

**DOI:** 10.3389/fgene.2023.1138703

**Published:** 2023-02-21

**Authors:** Xianzhi Zhang, Xinyi Wang, Linsi Pan, Wei Guo, Yongquan Li, Wencai Wang

**Affiliations:** ^1^ College of Horticulture and Landscape Architecture, Zhongkai University of Agriculture and Engineering, Guangzhou, China; ^2^ Science and Technology Innovation Center, Guangzhou University of Chinese Medicine, Guangzhou, China

**Keywords:** Eucommia ulmoides, MADS-box transcription factors, sex determination, floral organ ABCDE model-related genes, genome-wide identification, expression analysis

## Abstract

*Eucommia ulmoides* is a famous rubber-producing and medicinal tree species that produces unisexual flowers on separate individuals from the earliest stage of stamen/pistil primordium formation. To explore the genetic regulation pathway of sex in *E. ulmoides*, comprehensive genome-wide analyses and tissue-/sex-specific transcriptome comparisons of MADS-box transcription factors were performed for the first time in this work. Quantitative real-time PCR technique was employed to further validate the expression of genes that are assigned to floral organ ABCDE model. A total of 66 non-redundant *E. ulmoides* MADS-box (EuMADS) genes were identified, they were classified into Type I (M-type, 17 genes) and Type II (MIKC, 49 genes). Complex protein-motif composition, exon-intron structure and phytohormone-response cis-elements were detected in MIKC-EuMADS genes. Furthermore, 24 differentially-expressed EuMADS genes (DEGs) between male and female flowers, and two DEGs between male and female leaves were revealed. Amongst the 14 floral organ ABCDE model-related genes, there were 6 (A/B/C/E-class) and 5 (A/D/E-class) genes displayed male- and female-biased expression respectively. In particular, one B-class gene EuMADS39 and one A-class gene EuMADS65 were almost exclusively expressed in male trees, no matter in flower or leaf tissues. Collectively, these results suggested a critical role of MADS-box transcription factors in sex determination of *E. ulmoides*, which is conducive to decoding the molecular regulation mechanism of sex in *E. ulmoides*.

## 1 Introduction

MADS-box genes encode a class of transcription factors (TFs) that contain a highly conserved MADS domain (∼60 amino acids, aa) and are able to combine CArG-box *cis*-elements to regulate gene expression at transcriptional level (Theissen et al., 2018). ‘MADS’ is abbreviated from initials of four proteins in different organisms: MCM1 from yeast (M), AGAMOUS from *Arabidopsis* (A), DEFICIENS from snapdragon (D) and SRF from human (S) (Dong et al., 2021). In plants, the MADS-box gene family is categorized into two subfamilies, i.e., Type-I (M-type) and Type-II (MICK). M-type subfamily generally contains SRF domain and is classified into three lineages, i.e., Mα, Mβ, and Mγ (Wang et al., 2019). Whereas, MIKC subfamily usually possesses SRF or MEF2 domains and is divided into two lineages: MIKC^*^ and MIKC^C^ (superscript C means classic) (Gutierrez et al., 2022). Wherein genes belong to MIKC^C^ are the most well-studied MADS-box genes by far, they contain as many as 13 subgroups: AG/SHP, AGL6, AGL12, AGL15, ANR1, AP1, AP3/PI, FLC, SEP, SOC1, SVP, TM8, and TT16 (Theißen et al., 2016; Alhindi and Al-Abdallat, 2021).

MADS-box TFs play critical roles in various processes of plant development, such as flowering control, floral organogenesis, and fruit ripening (Theissen and Saedler, 2001; Lai et al., 2022). Nearly all the well-known floral homeotic genes of ‘ABCDE model’ belong to the MADS-box family (Theien, 2001; Soltis et al., 2007). According to ‘ABCDE model’, the sepal, petal, stamen and carpel of bisexual flower are specifically regulated by A + E, A + B + E, B + C + E and C + E genes respectively, and ovules by C + D + E (Theissen and Saedler, 2001; Theißen et al., 2016). In the well-studied *Arabidopsis* there are 12 A/B/C/D/E-class genes, i.e., A-class: *APETALA1* (*AP1*) and *APETALA2* (*AP2*); B-class: *APETALA3* (*AP3*) and *PISTILLATA* (*PI*); C-class: *AGAMOUS* (*AG*); D-class: *SHATTERPROOF1*, *2* (*SHP1* and *SHP2*) and *SEEDSTICK* (*STK*); and E-class: *SEPALLATA1*, *2*, *3*, *4* (*SEP1*, *SEP2*, *SEP3* and *SEP4*) (Soltis et al., 2007). All these 12 genes, except for *AP2* belong to MADS-box family (Theien, 2001).

Recent studies revealed that plant sex is highly associated with MADS-box genes (Zhang et al., 2022). For example, *LcMADS42/46/47/51/75/93/100* are probably involved in the unisexual flower formation of litchi (*Litchi chinensis* Sonn.) (Guan et al., 2021). One MADS-box gene *QsPISTILLATA* is also exclusively expressed in male flowers of *Quercus suber* L., and is proved to be functional for stamen development (Sobral and Costa, 2017). Remarkably, B-class MADS-box TFs are essential for sex determination of several dioecious plants (Zhang et al., 2022). In spinach (*Spinacia oleracea* L.), B-class genes *SpAP3* and *SpPI* are expressed specifically in the floral primordial of male. Suppression of *SpAP3* or *SpPI* turned male individuals into female (Pfent et al., 2005; Sather et al., 2010). Similarly, in *Thalictrum dioicum* L., all B-class genes (*ThdAP3-1*, *ThdAP3-2a*, *ThdAP3-2b*, *ThdPI-1* and *ThdPI-2*) are only expressed in floral buds of male but not female at very initial stage. Knocking-down the expression of *ThdPI-1* or *ThdPI-2* also converted male individuals into female (Di Stilio et al., 2005; Larue et al., 2013).

*Eucommia ulmoides* Oliv. is known as the hardy rubber tree that can synthesize gutta-rubber (*trans*-1,4-polyisoprene, TPI) almost in the whole plant, especially in fruits (Wang et al., 2018). It is also the resource of well-known traditional Chinese medicine ‘DuZhong’ (Ouyang et al., 2021). Meanwhile, this species is a strict dioecious plant with stamen primordium or pistil primordium initiating in separate individuals, suggesting that its flowers are unisexual from inception (Wang et al., 2018; Qing et al., 2021). Some MADS-box TFs, especially them involved in ‘ABCDE model’ of flower development, have been suggested to take part in sex determination of dioecious plants with such type of unisexual flowers (Diggle et al., 2011; Zhang et al., 2022). However, the characteristics and roles of MADS-box TFs in sex determination of *E. ulmoides* remain largely unknown by far.

In this work, MADS-box TFs of *E. ulmoides* were identified throughout the genome and comprehensively characterized, e.g., their evolutionary relationships, protein motif compositions, gene structures, and phytohormone response *cis*-elements. Tissue- and sex-specific expression profiling of all the MADS-box genes were investigated based on transcriptome data. Quantitative real-time PCR (qRT-PCR) analyses were further conducted to uncover the sex-biased expression patterns of A/B/C/D/E-class genes involved in flower development. The results obtained herein will on the one hand shed lights on our understanding of the evolution and function of MADS-box TFs in *E. ulmoides*, on the other, will promote decoding the molecular regulation mechanism of sex in this dioecious plant.

## 2 Results

### 2.1 Genome-wide identification and synteny analysis of MADS-Box TFs in E. ulmoides

A total of 66 non-redundant MADS-box TFs were identified in *E. ulmoides* for the first time according to BLASTP and HMMER analyses, named as EuMADS01-66 (Table 1). Wherein 64 of them were derived from the haploid *E. ulmoide* genome (Li et al., 2020), the rest two were discovered in the male genome (Wuyun et al., 2018), with 58 members being commonly detected from both the haploid and male genomes. The identified 66 EuMADS TFs were further verified in SMART and CDD databases for the presence of conserved MADS domain. These MADS-box TFs were sized from 95 aa (EuMADS04) to 471 aa (EuMADS62) with an average of 232 aa. Their isoelectric points (pl) varied from 4.7 (EuMADS02) to 11.5 (EuMADS05), molecular weight (MW) were between 10.7 kDa (EuMADS04) and 53.4 kDa (EuMADS62), number of phosphorylation sites (Ser, Tyr and Thr sites) were from 11 (EuMADS04) to 72 (EuMADS62). As expected, subcellular locations of all the 66 EuMADS TFs were predicted to be in the nucleus. Information of the nucleotide and amino-acid sequences of these 66 *EuMADS* genes were documented in Additional Data1.

**TABLE 1:** Detailed information of the *EuMADS* genes.

Gene name	Gene ID	No. of Exon	Length (aa)	pl	MW (kDa)	No. of phosphorylation sites	Subcellular location	Group	Subgroup
*EuMADS01*	evm.model.Chr1.508	8	255	6.3	28.9	47	Nucleus	MIKC	SVP
*EuMADS02*	evm.model.Chr1.760	1	241	4.7	26.8	35	Nucleus	M-type	Mγ
*EuMADS03*	evm.model.Chr1.1507	8	247	7.6	28.6	42	Nucleus	MIKC	SEP
*EuMADS04*	evm.model.Chr2.157	2	95	11.2	10.7	11	Nucleus	MIKC	ANR1
*EuMADS05*	evm.model.Chr2.165	2	126	11.5	14.3	14	Nucleus	MIKC	ANR1
*EuMADS06*	evm.model.Chr2.849	8	247	9.8	28.8	44	Nucleus	MIKC	AG/SHP
*EuMADS07*	evm.model.Chr2.2202	13	360	6.7	41.4	55	Nucleus	MIKC	MIKC^*^
*EuMADS08*	evm.model.Chr4.480	1	137	7.8	15.6	21	Nucleus	M-type	Mα
*EuMADS09*	evm.model.Chr4.912_evm.model.Chr4.913	12	231	8.2	26.3	36	Nucleus	MIKC	MIKC^*^
*EuMADS10*	evm.model.Chr4.1258	8	241	9.8	28.1	41	Nucleus	MIKC	AG/SHP
*EuMADS11*	evm.model.Chr4.1858	8	211	8.6	24.5	28	Nucleus	MIKC	SOC1
*EuMADS12*	evm.model.Chr4.1859	8	209	8.6	24.3	27	Nucleus	MIKC	SOC1
*EuMADS13*	evm.model.Chr5.284	9	245	10.0	28.6	43	Nucleus	MIKC	AG/SHP
*EuMADS14*	evm.model.Chr6.601	1	214	4.9	24.0	25	Nucleus	M-type	Mγ
*EuMADS15*	evm.model.Chr6.713	1	181	7.5	20.7	20	Nucleus	M-type	Mα
*EuMADS16*	evm.model.Chr6.841_evm.model.Chr6.844	9	200	8.7	22.9	30	Nucleus	MIKC	SOC1
*EuMADS17*	evm.model.Chr7.299	1	242	9.9	27.4	27	Nucleus	M-type	Mγ
*EuMADS18*	evm.model.Chr7.936	10	238	10.2	27.3	36	Nucleus	MIKC	ANR1
*EuMADS19*	evm.model.Chr8.497	10	313	5.9	35.4	49	Nucleus	MIKC	MIKC^*^
*EuMADS20*	evm.model.Chr8.771	12	330	5.0	37.7	50	Nucleus	MIKC	MIKC^*^
*EuMADS21*	evm.model.Chr8.957_evm.model.Chr8.968	7	204	10.1	23.7	26	Nucleus	MIKC	SVP
*EuMADS22*	evm.model.Chr8.961	7	204	9.8	23.8	30	Nucleus	MIKC	SVP
*EuMADS23*	evm.model.Chr8.964.1	6	176	11.0	19.8	28	Nucleus	MIKC	SVP
*EuMADS24*	evm.model.Chr8.970	8	246	7.9	27.8	31	Nucleus	MIKC	SVP
*EuMADS25*	evm.model.Chr8.971	7	290	5.3	32.6	51	Nucleus	MIKC	SVP
*EuMADS26*	evm.model.Chr8.973	10	240	7.3	27.1	34	Nucleus	MIKC	SVP
*EuMADS27*	evm.model.Chr8.985	11	365	9.5	44.2	52	Nucleus	MIKC	ANR1
*EuMADS28*	evm.model.Chr8.1691	7	196	7.6	23.1	24	Nucleus	MIKC	AP3/PI
*EuMADS29*	evm.model.Chr8.2938.2	7	196	9.8	22.7	29	Nucleus	MIKC	SOC1
*EuMADS30*	evm.model.Chr9.65	4	183	7.5	20.8	28	Nucleus	MIKC	FLC
*EuMADS31*	evm.model.Chr9.66	3	109	11.2	12.6	16	Nucleus	MIKC	FLC
*EuMADS32*	evm.model.Chr9.67	8	178	8.1	20.3	24	Nucleus	MIKC	FLC
*EuMADS33*	evm.model.Chr9.70	8	243	8.0	27.9	41	Nucleus	MIKC	SEP
*EuMADS34*	evm.model.Chr9.275	8	244	7.9	28.4	34	Nucleus	MIKC	SEP
*EuMADS35*	evm.model.Chr9.1822	1	287	6.4	32.9	42	Nucleus	M-type	Mγ
*EuMADS36*	evm.model.Chr10.369	8	202	10.7	23.4	32	Nucleus	MIKC	TM8^#^
*EuMADS37*	evm.model.Chr10.894	6	215	10.4	24.8	37	Nucleus	MIKC	SEP
*EuMADS38*	evm.model.Chr10.903	8	247	9.5	28.6	33	Nucleus	MIKC	AP1
*EuMADS39*	evm.model.Chr10.1970_evm.model.Chr10.1971	7	228	9.2	26.6	38	Nucleus	MIKC	AP3/PI
*EuMADS40*	evm.model.Chr12.286	9	213	8.8	24.8	30	Nucleus	MIKC	SOC1
*EuMADS41*	evm.model.Chr12.846	7	200	7.2	23.2	27	Nucleus	MIKC	AGL12
*EuMADS42*	evm.model.Chr12.1177	1	230	6.3	25.6	31	Nucleus	M-type	Mα
*EuMADS43*	evm.model.Chr12.1179	1	229	6.3	25.6	31	Nucleus	M-type	Mα
*EuMADS44*	evm.model.Chr12.1521	7	272	6.8	32.3	37	Nucleus	MIKC	TT16
*EuMADS45*	evm.model.Chr13.676	11	361	5.9	41.3	60	Nucleus	MIKC	MIKC^*^
*EuMADS46*	evm.model.Chr13.1742	4	330	7.1	36.9	50	Nucleus	M-type	Mα
*EuMADS47*	evm.model.Chr13.1746	1	211	8.8	23.9	36	Nucleus	M-type	Mα
*EuMADS48*	evm.model.Chr15.40	6	234	9.2	26.4	36	Nucleus	MIKC	FLC
*EuMADS49*	evm.model.Chr15.353	7	180	9.8	21.1	24	Nucleus	MIKC	AP1
*EuMADS50*	evm.model.Chr15.579	9	236	9.9	27.3	29	Nucleus	MIKC	ANR1
*EuMADS51*	evm.model.Chr15.704	1	293	7.9	33.9	46	Nucleus	M-type	Mγ
*EuMADS52*	evm.model.Chr15.1893	1	205	10.3	22.9	27	Nucleus	M-type	Mα
*EuMADS53*	evm.model.Chr15.1909	3	197	10.2	22.4	21	Nucleus	M-type	Mα
*EuMADS54*	evm.model.Chr16.288	7	258	8.6	29.7	45	Nucleus	MIKC	AGL15
*EuMADS55*	evm.model.Chr16.1630	8	250	7.1	28.4	35	Nucleus	MIKC	SOC1
*EuMADS56*	evm.model.Chr16.1633	8	215	9.8	24.8	32	Nucleus	MIKC	ANR1
*EuMADS57*	evm.model.Chr17.474	2	168	8.5	19.7	20	Nucleus	M-type	Mγ
*EuMADS58*	evm.model.Chr17.480	2	204	7.9	23.9	18	Nucleus	M-type	Mγ
*EuMADS59*	evm.model.Chr17.842	8	238	10.0	27.5	33	Nucleus	MIKC	ANR1
*EuMADS60*	evm.model.Chr17.931	1	222	10.1	24.4	32	Nucleus	M-type	Mα
*EuMADS61*	evm.model.Chr17.1486	8	266	4.8	30.0	45	Nucleus	MIKC	AGL15
*EuMADS62*	evm.model.000007F_4.12	3	471	6.9	53.4	72	Nucleus	M-type	Mγ
*EuMADS63*	evm.model.000124F.33	7	197	7.3	22.7	33	Nucleus	MIKC	FLC
*EuMADS64*	evm.model.000124F.35	7	243	8.8	27.8	38	Nucleus	MIKC	SEP
*EuMADS65* ^*^	GWHGAAAL024617	8	241	7.2	28.1	33	Nucleus	MIKC	AP1
*EuMADS66* ^*^	GWHGAAAL024655	6	184	9.9	21.3	30	Nucleus	MIKC	AP1

* Note: Genes are detected from the male reference genome of *Eucommia ulmoides* (Wuyun et al., 2018). #*TM8* gene s present in *Solanum lycopersicum* (Wang et al., 2019) but not in *Arabidopsis thaliana* (Parenicová et al., 2003).

**FIGURE 1:**
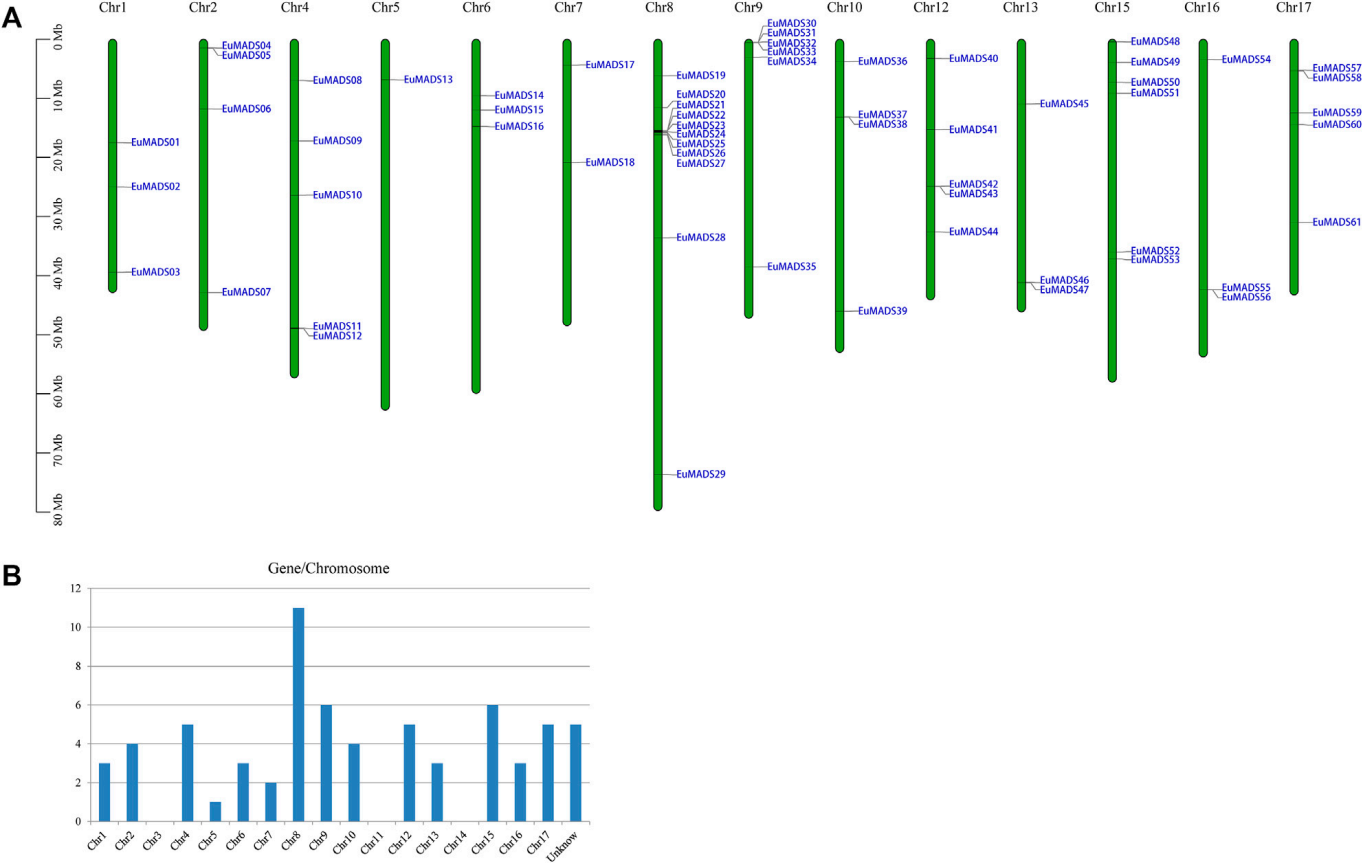
Chromosomal distribution of the 66 *EuMADS* genes. **(A)** Schematic representation of the chromosomal localization of *EuMADS* genes. Each vertical bar represents one chromosome indicated by numbers above. Chromosome length is scaled on the left. **(B)** The density of *EuMADS* genes on each chromosome. *EuMADS* genes with unknown chromosomal position are shown as unknown.

**FIGURE 2:**
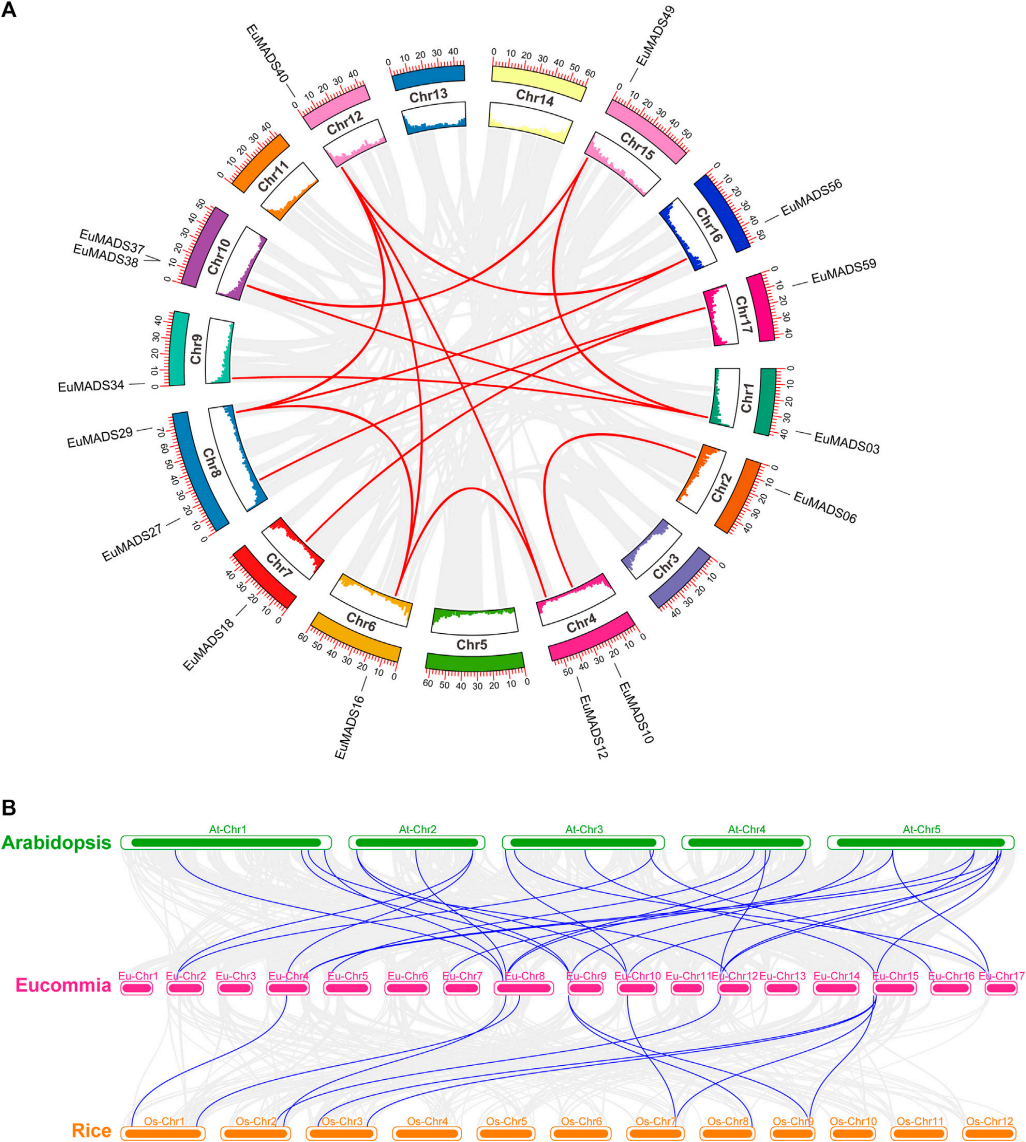
Synteny analysis of *EuMADS* genes within *E. ulmoides* and between species. **(A)** Inter-chromosomal relationships of *EuMADS* genes within *E. ulmoides*. Gray lines indicate all synteny blocks in *E. ulmoides* genome, red lines indicate duplicated *EuMADS* gene pairs. Inside boxes indicate gene density of each corresponding chromosome. **(B)** Collinear relationships of *EuMADS* genes between *E. ulmoides* and the other two species (*Arabidopsis* and rice). Gray lines indicate the syntenic blocks between *Eucommia* and *Arabidopsis*/rice, blue lines highlight the collinear *EuMADS* gene pairs.

The 66 *EuMADS* genes were then mapped to the assembled 17 chromosomes of haploid *E. ulmoides* (Li et al., 2020) (Figure 1A). As a result, a highly uneven distribution pattern of *EuMADS* genes was revealed, with Chromosome 8 (Chr8) harboring the maximum number of *EuMADS* genes (11, 16.7%), followed by Chr9 and Chr15 (both contain 6, 9.1%), and Chr4 and Chr17 (both contain 5, 7.6%). While only one was localized on Chr5, none was seen on Chr3, Chr11 and Chr14 (Figure 1B). Besides, five *EuMADS* genes were unable to be assigned to any of the 17 assembled chromosomes (Li et al., 2020). Moreover, intra-genome and inter-genome relations of *EuMADS* genes were analyzed by MCScanX. The results revealed that 15 out of 66 *EuMADS* genes locating in 14 pairs of segmental duplicates showed collinearity between chromosomes in *E. ulmoides* (Figure 2A, Supplementary Table S1). Meanwhile, synteny analyses of MADS-box genes between *Eucommia* and *Arabidopsis*/rice showed more collinear regions with *Arabidopsis* (27) than that with rice (11) (Figure 2B). This may suggest closer evolutionary relationship between the two eudicot plants, i.e., *Eucommia* and *Arabidopsis*.

### 2.2 Phylogenetic relationship and classification of EuMADS TFs

**FIGURE 3:**
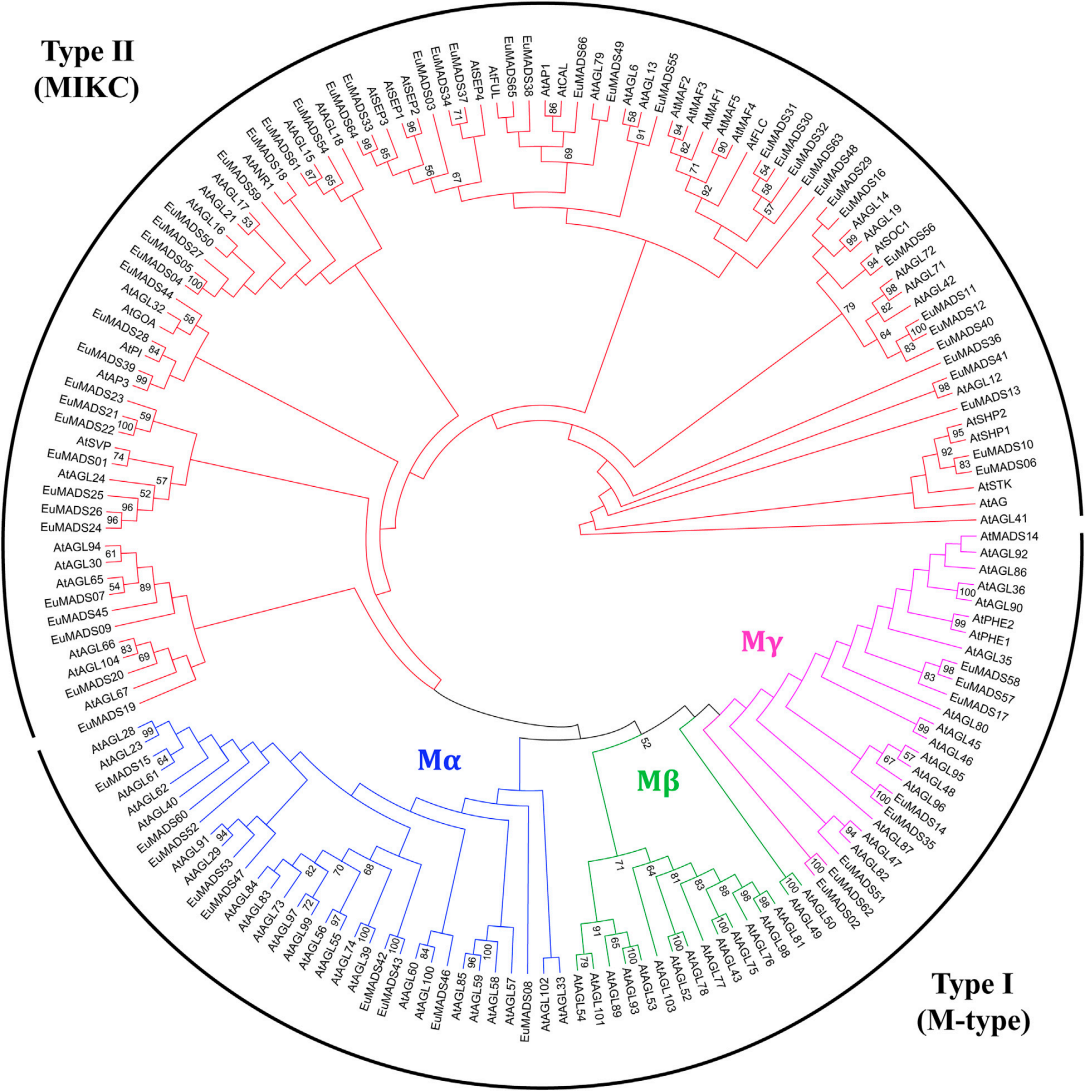
Maximum likelihood tree of MADS-box transcription factors in *E. ulmoides* and *A. thaliana*. The tree reveals two subfamilies of the MADS-box gene family, i.e., Type I (M-type) and Type II (MIKC). In M-type group, three subgroups, i.e., Mα, Mβ and Mγ are shown in blue, green and purple, respectively. MIKC group is colored in red.

In order to correctly classify the *EuMADS* genes, phylogenetic relationship among the EuMADS TFs (66) and MADS-box TFs of *A. thaliana* (AtMADS, 103) were analyzed. Maximum likelihood (ML) tree showed that 17 EuMADS TFs were clustered in Type I (M-type) subfamily, while the rest 49 belonged to Type II (MIKC) (Figure 3). In M-type subfamily, nine EuMADS TFs were clustered in Mα lineage while the remaining eight genes were distributed in Mγ lineage. The Mβ lineage was absent in *E. ulmoides*.

**FIGURE 4:**
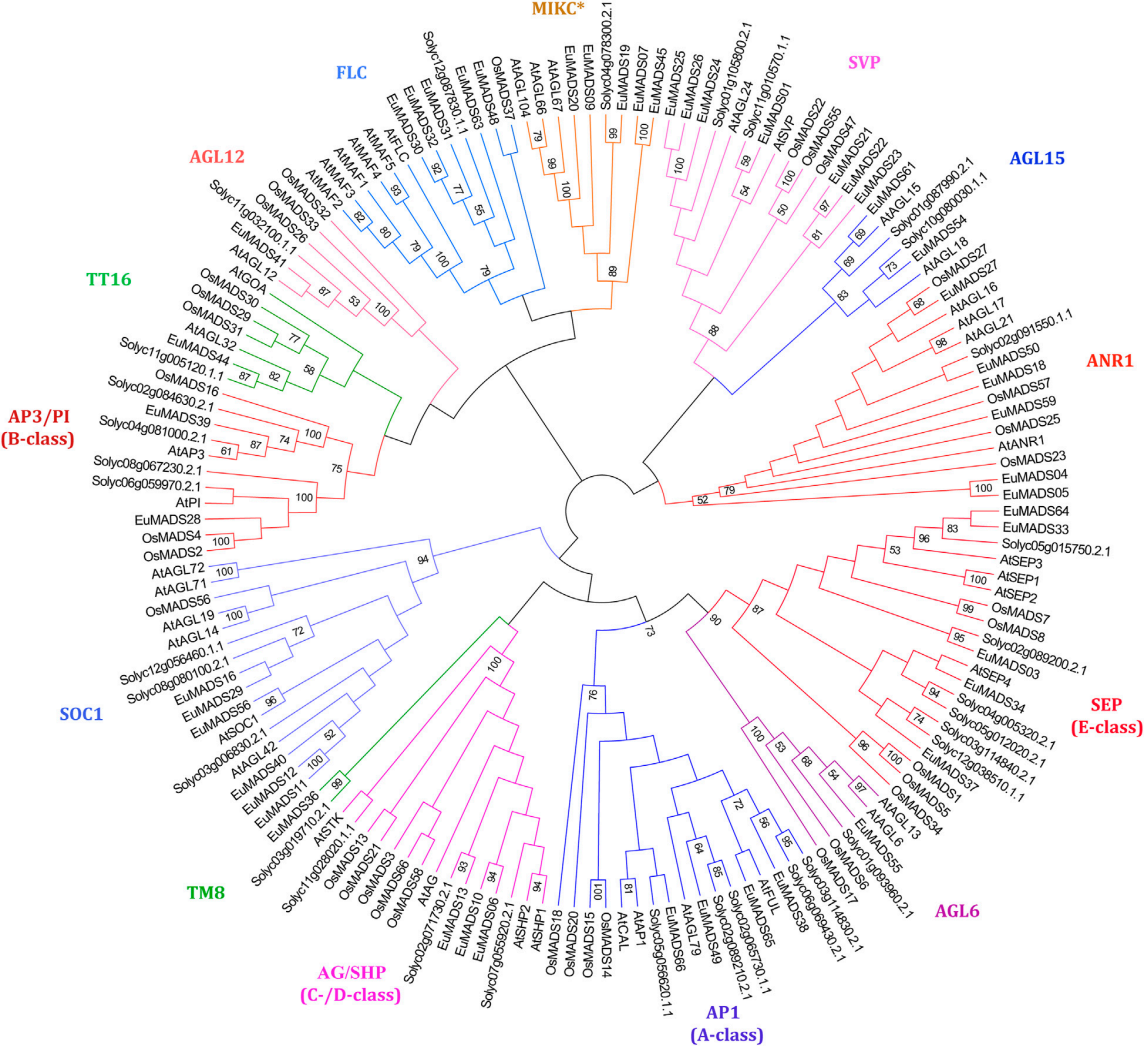
Maximum likelihood tree of MIKC MADS-box transcription factors in *E. ulmoides*, *A. thaliana*, *S. lycopersicum* and *Oryza sativa*. MIKC^*^ clade and 13 known MIKC^C^ subgroups are indicated in different colors.

MIKC MADS-box TFs of tomato (MIKC-SolyMADS, 32), rice (MIKC-OsMADS, 34) and *Arabidopsis* (MIKC-AtMADS, 42) were further used to classify MIKC-EuMADS TFs (49). The phylogenetic tree was branched into 14 clades, corresponding to the 13 known subgroups identified in MIKC^C^ lineage, plus MIKC^*^ subgroup (Figure 4). There were respectively four A-class genes (*EuMADS38*, *49*, *65*, and *66*), two B-class genes (*EuMADS28* and *39*), three C-/D-class genes (*EuMADS06*, *10* and *13*), and five E-class genes (*EuMADS03*, *04*, *33*, *37*, and *64*) detected in *E. ulmoides*. Some MIKC-EuMADS TFs were scattered across other nine clades of MIKC^C^. For instance, each of the AGL6, TM8, TT16, AGL12 subgroup harbored one different MIKC-EuMADS member, while AGL15, FLC subgroup contained two and five respectively, both SOC1 and ANR1 subgroup had six, SVP subgroup included seven. Note, there were only five MIKC-EuMADS TFs clustered in MIKC^*^ lineage.

### 2.3 Protein domain, gene structure, and promotor cis-element of EuMADS TFs

**FIGURE 5:**
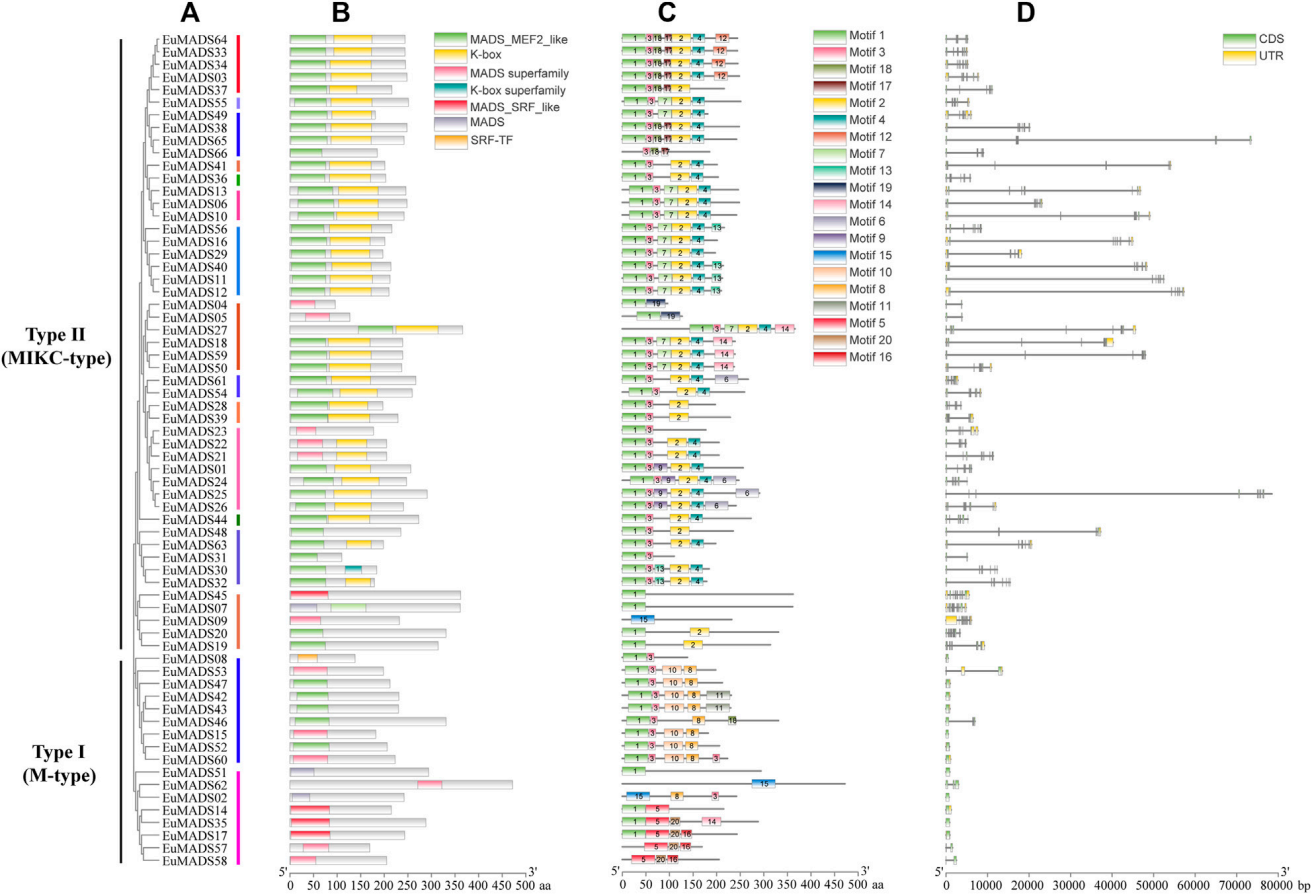
Protein domain, motif and gene structure of EuMADS transcription factors. **(A)** Phylogenetic relationship of the *EuMADS* gene family. Type I (M-type) including 2 clades and Type II (MIKC) including 14 clades are indicated by colored vertical lines. **(B)** Conserved protein domains of EuMADS transcription factors. **(C)** Protein motif composition of EuMADS transcription factors. The seven domains and 20 motifs are boxed in different colors, respectively. **(D)** Exon-intron structure of *EuMADS* genes. CDS, UTR and intron are represented by green, yellow boxes and black lines, respectively.

To obtain more information about functions of EuMADS TFs, the protein domain/motif composition, exon-intron organization, and *cis*-elements in promotor region were analyzed for each *EuMADS* gene. The conserved domains of 66 EuMADS TFs were identified using Batch CD-Search in NCBI. In summary, EuMADS TFs contained seven types of conserved domains in two categories, i.e., MADS (MADS-MEF2-like, MADS superfamily, MADS-SRF-like, MADS, SRF-TF) and K-box (K-box, K-box superfamily) (Figure 5B). As expected, all the 66 EuMADS proteins contained conserved MADS domain. Among the 49 Type II (MIKC-EuMADS) TFs, 38 proteins possessed both MADS and K-box domains, whereas the rest 11 proteins lacked K-box domain. Meanwhile, there was only MADS domain but no K-box domain in all the 17 Type I (M-type EuMADS) TFs. These results suggested functional differentiation among the EuMADS members. Moreover, the protein motif composition also varied obviously between the two EuMADS subfamilies (Figure 5C). Motif 1 was the most conserved motif in the EuMADS TF family and distributed in the vast majority of members (60/66, 90.9%). Motif 2 was present in most MIKC-EuMADS members (41/49, 83.7%), but not found in any M-type EuMADS TFs. Interestingly, motif composition of EuMADS TFs from the same phylogenetic clade was similar, indicating possible resemblance in biological functions. For example, protein motifs in the AP3/PI subgroup (B-class) all arranged conservatively as 1-3-2, while the AG/SHP subgroup (C-/D-class) all usually arrayed as 1-3-7-2-4.

Gene structure analysis showed that the composition of exon/introns between the M-type and MIKC subfamily were distinct (Table 1; Figure 5D). M-type *EuMADS* genes embodied 1-4 exons per gene (averagely *c.* 1.5), whereas MIKC genes housed 2–13 exons (averagely *c.* 7.8). Most M-type *EuMADS* genes (12/17, 70.6%) contained only one exon but none of introns, almost all the MIKC-*EuMADS* genes (45/49, 91.8%) harbored at least six exons and five introns. More exons in MIKC-*EuMADS* genes may imply their complexity and/or variation of biological functions. The number of exons in genes from the same phylogenetic clade was usually similar, e.g., seven exons present in *EuMADS28* and *EuMADS39*, both belong to clade AP3/PI (B-class). The size of *EuMADS* genes varied largely, from 527 bp (*EuMADS52*, Mα subgroup of M-type) to 78,479 bp (*EuMADS25*, clade SVP of MIKC).

**FIGURE 6:**
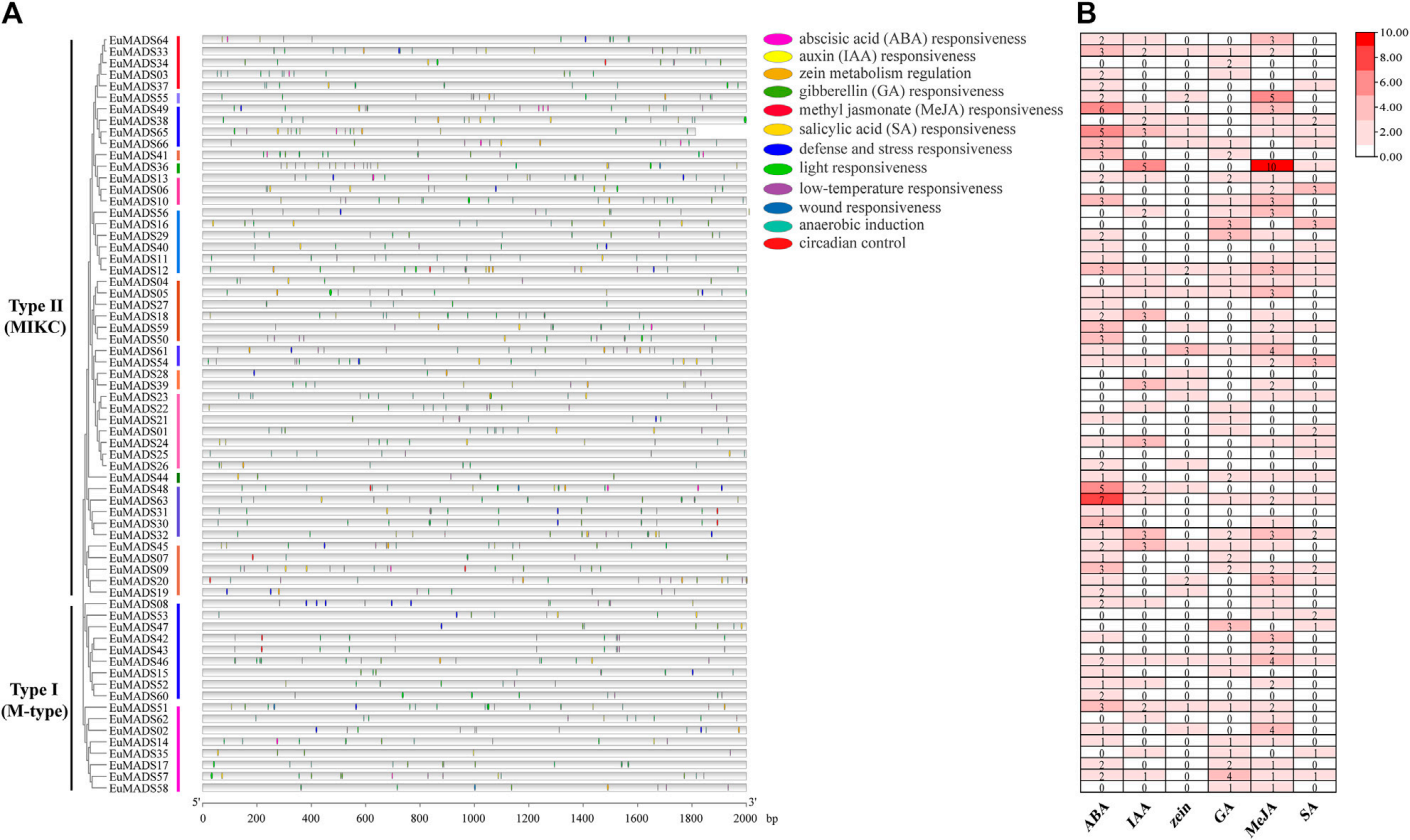
Predicted *cis*-acting regulatory elements for *EuMADS* genes. **(A)** The physical locations of *cis*-elements in the promoter region of each *EuMADS* gene, arranged by the phylogenetic tree. The 12 functional modules are shown as colored ellipse boxes. **(B)** Counts of phytohormone (ABA, IAA, zein, GA, MeJA, and SA) response *cis*-elements in each *EuMADS* gene.

The 2,000 bp promoter sequences upstream of the start codon (ATG) of each *EuMADS* gene were obtained from the genome of *E. ulmoides* (Wuyun et al., 2018; Li et al., 2020) to predict the *cis*-acting regulatory elements (CAREs) by PlantCARE. A total of 1,140 CAREs belonging to 22 types and 12 functional modules were found (Figure 6A, Supplementary Table S2), suggesting extensive biological functions of EuMADS TFs in the growth and development of *E. ulmoides*. Among the 12 functional modules, light-response CAREs had the largest number (313), including seven types, i.e., G-Box, Sp1, GT1-motif, 3-AF1 binding site, 4cl-CMA2b, ACE, and AAAC-motif. Meanwhile, *EuMADS63* in Type II (MIKC) subfamily contained the most CAREs (36), while *EuMADS35* of Type I (M-type) subfamily had the fewest (5). Notably, a total of 568 phytohormone-response CAREs were detected, consisting of 172 abscisic acid (ABA), 51 auxin (IAA), 31 zein, 60 gibberellin (GA), 212 methyl jasmonate (MeJA), and 42 salicylic acid (SA) (Figure 6B).

### 2.4 Tissue- and sex-specific expression profiling and protein-interaction analysis of EuMADS TFs

**FIGURE 7:**
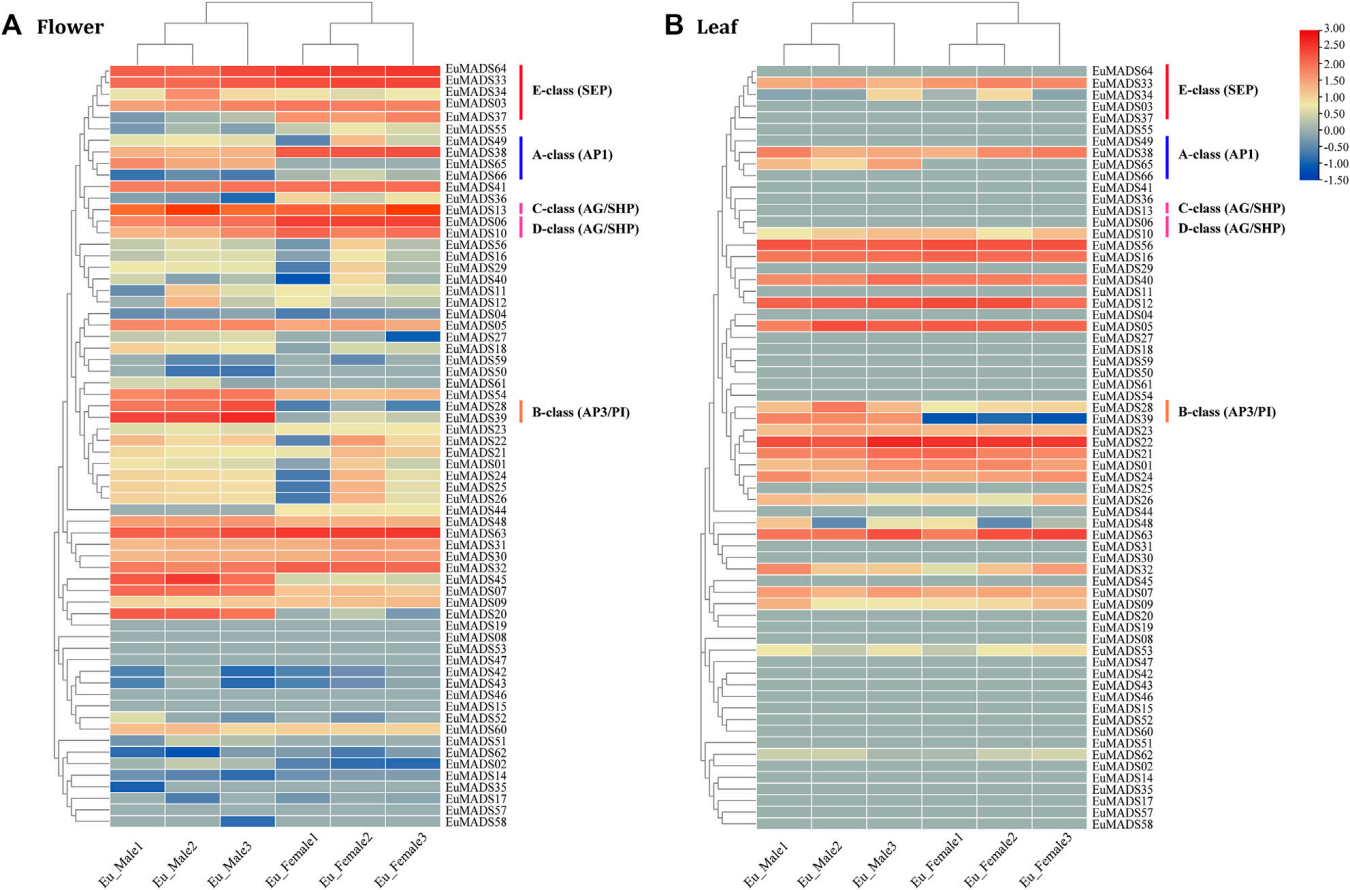
Expression profiling of EuMADS genes in tissues of **(A)** flower and **(B)** leaf. FPKM values of 66 *EuMADS* genes were calculated using transcriptome data with normalization. Log_2_(FPKM value) is displayed from low (blue) to high (red), indicating expression levels. A/B/C/D/E-class genes involved in flower development are highlighted by vertical lines in different colors.

To understand the biological functions of EuMADS TFs, expression patterns of all the 66 *EuMADS* genes were investigated in both reproductive (flower) and vegetative (leaf) tissues of male and female, *via* comparative transcriptome analysis. FPKM values were calculated to measure the expression level of each *EuMADS* gene. Overall, more *EuMADS* genes were expressed in flowers (59/66, 89.4%) than that in leaves (25/66, 37.9%, Figure 7). All the MIKC-*EuMADS* genes, excluding *EuMADS19* were actively expressed in flowers. On the contrary, more members of FLC (3/5, *EuMADS32*, *48*, and *63*), SVP (6/7, *EuMADS01*, *21*, *22*, *23*, *24*, and *26*), and SOC1 (4/6, *EuMADS12*, *16*, *40*, and *56*) were preferred to express in leaves than in flowers.

Based on the criteria of a minimal two-fold difference in expression, i.e., log2 (fold change value) *≥*1 or ≤ −1, and an adjusted *p*-value (*p*adj) < 0.05, we found that 24 *EuMADS* genes were differentially expressed genes (DEGs) between the male and female flowers (Figure 7A, Supplementary Table S3). Among the 24 DEGs, 16 members (*EuMADS02*, *07*, *13*, *18*, *20*, *27*, *28*, *34*, *39*, *45*, *48*, *49*, *51*, *54*, *61*, and *65*) displayed a male-biased expression pattern, while the rest eight (*EuMADS06*, *10*, *36*, *37*, *38*, *44*, *55*, and *66*) were preferentially expressed in females. In contrast, only two genes, i.e., *EuMADS39* (B-class gene) and *EuMADS65* (A-class gene) were differentially expressed between the male and female leaves, both appeared male-biased expression (Figure 7B, Supplementary Table S3). Notably, the vast majority of DEGs (19/24, 79.2%) belonged to the MIKC^C^ lineage of MIKC subfamily. This probably implied more important roles of MIKC^C^-*EuMADS* genes in floral sex differentiation of *E. ulmoides*.

**FIGURE 8:**
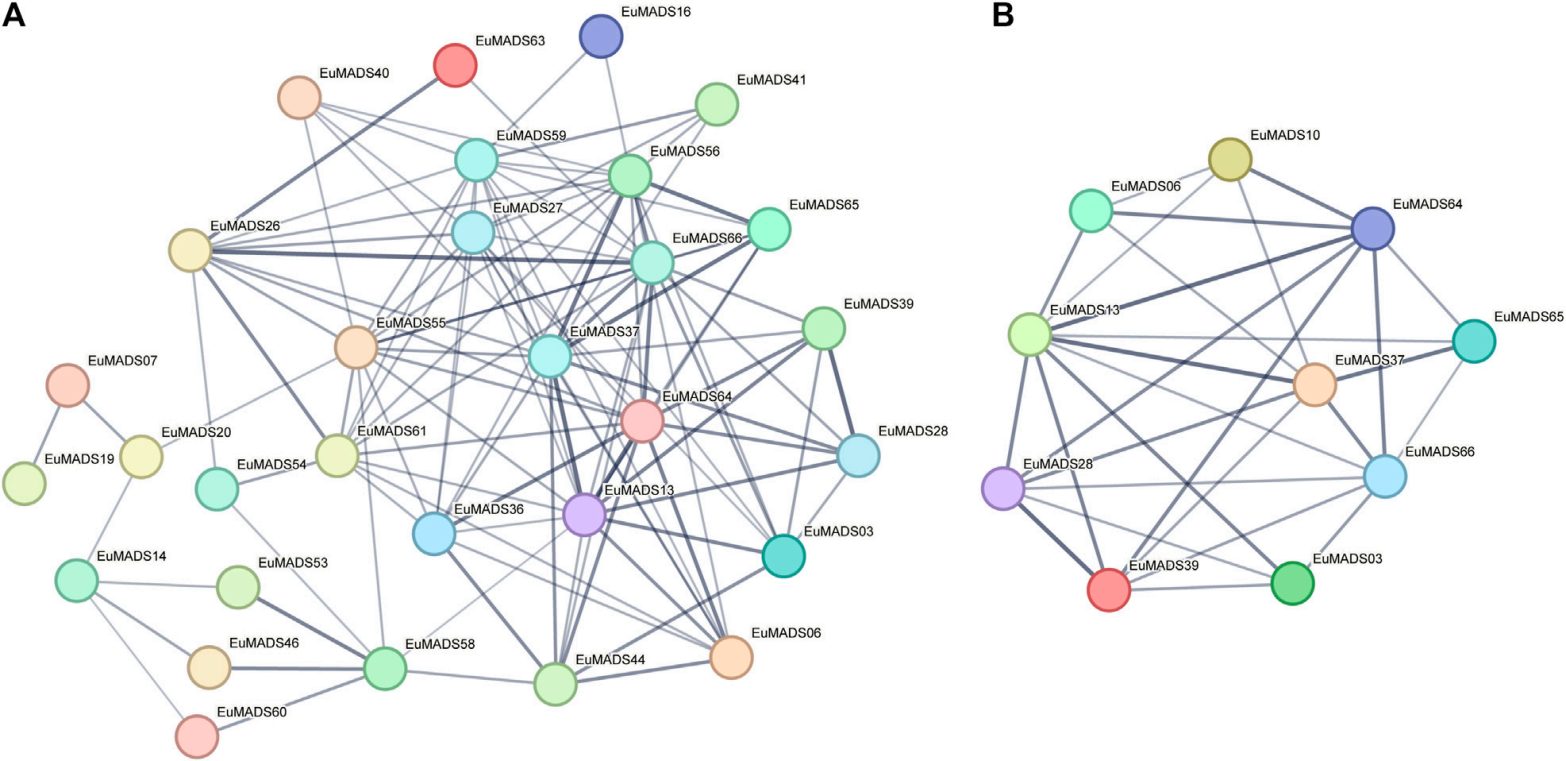
Putative interactions between EuMADS proteins. **(A)** Prediction of the protein interaction network among 66 EuMADS. **(B)** Prediction of the protein interaction network among 14 EuMADS involved in the floral organ ‘ABCDE model’.

Given that some EuMADS TFs may participate together in the same pathway, e.g., floral organ development, protein-interactive analysis was performed by STRING. The results showed that 30 out of 66 EuMADS proteins could interact with each other (Figure 8A, Supplementary Table S4). Among these putatively interacting proteins, 10 were involved in the floral organ ‘ABCDE model’ (Figure 8B). The two B-class genes (*EuMADS28* and *EuMADS39*) were shown to interact with each other, and both of them could interact with the C-class gene (*EuMADS13*). Some E-class genes, e.g., *EuMADS64* and *EuMADS37* could interact with all the members of B/C/D-class (*EuMADS06*, *10*, *13*, *28*, and *39*), and two A-class genes (*EuMADS65* and *EuMADS66*).

### 2.5 Sex-biased expression pattern of floral organ ABCDE model-related genes

**FIGURE 9:**
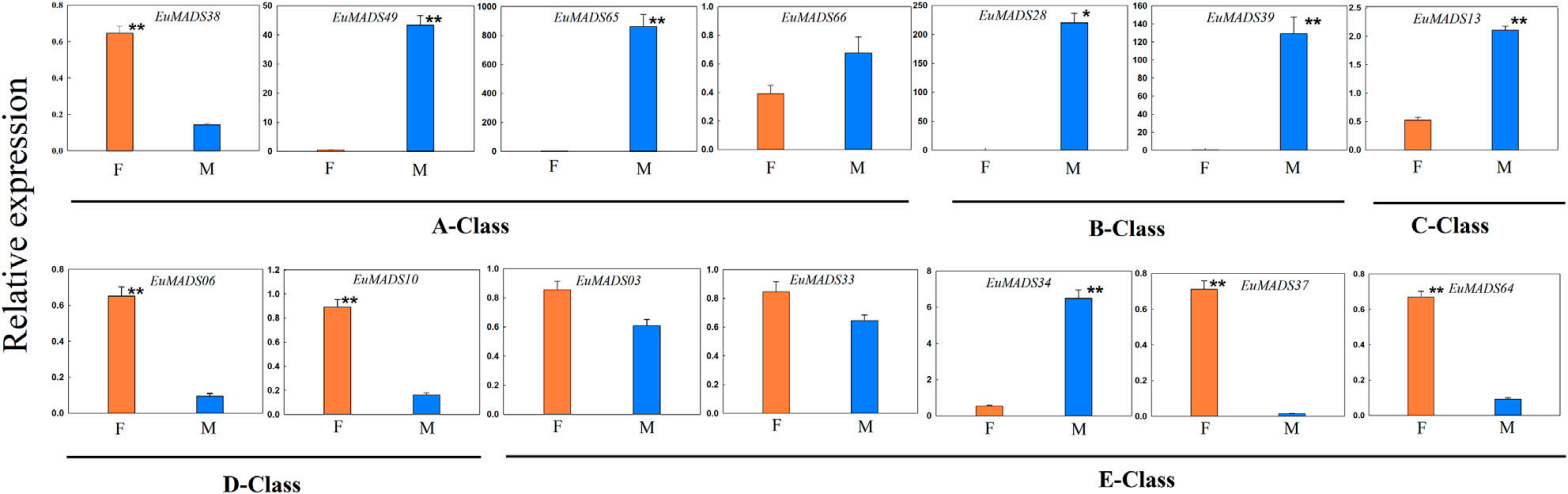
Quantitative real-time PCR (qRT-PCR) analyses of A/B/C/D/E-class EuMADS transcription factors in female (F) and male (M) flowers of *Eucommia ulmoides*. A-class genes (*EuMADS38*, *EuMADS49*, *EuMADS65* and *EuMADS66*), B-class genes (*EuMADS28* and *EuMADS39*), C-class gene (*EuMADS13*), D-class genes (*EuMADS06* and *EuMADS10*) and E-class genes (*EuMADS03*, *EuMADS33*, *EuMADS34*, *EuMADS37* and *EuMADS64*) are all underlined. The error bars were calculated from five biological replicates and two technical replicates. Significant differences between sexes are indicated with *(*p* < 0.05) and **(*p* < 0.01).

All the floral organ ABCDE model-related genes that were revealed active in flowers by transcriptome data (Figure 7) were further selected for qRT-PCR analysis. The result revealed most of A/B/C/D/E-class genes (11/14, 78.6%) were DEGs between the male and female flowers (Figure 9), in line with the above results of transcriptome analysis (Figure 7A, Supplementary Table S3). Remarkably, the expression patterns of most A/B/C/D/E-class genes between the male and female flowers were in accordance with their identities in determining floral organs. For example, B-class genes, i.e., *EuMADS28* and *EuMADS39* both highly expressed in the male flowers, but hardly expressed in the female flowers. Two D-class genes, *EuMADS06* and *EuMADS10* both showed female-biased expression patterns. One A-class gene (*EuMADS66*) and two E-class genes (*EuMADS03* and *EuMADS33*) displayed similar expression level between the male and female flowers. Nevertheless, there were some exceptions, for instance, *EuMADS49* and *EuMADS65* of A-class were highly expressed in male flowers, while their closely relative *EuMADS38* expressed preferably in female. C-class gene *EuMADS13* was expressed in both male and female flowers, but with a higher expression level in male. In E-class, *EuMADS34* showed male-biased expression, while *EuMADS37* and *EuMADS64* had male-biased expressions.

In addition, two B-class genes (*EuMADS28* and *EuMADS39*) were also actively transcribed in the male leaves (Figure 6B). *EuMADS39* that was homologous to *AP3* of *Arabidopsis* and *DEF* of snapdragon (*Antirrhinum majus* L.) (Table 2) showed a male-biased expression pattern consistently in both flowers and leaves (Supplementary Tables S1; Figures 6, 7). Notably, *EuMADS65* of A-class that shared homology with gene *FUL* in *Arabidopsis* and gene *DEFH28* in snapdragon was also expressed specifically in both flower and leaf of male individuals. These two genes were therefore most probably involved in sex determination of *E. ulmoides*.

**TABLE 2:** Function annotation of *EuMADS* genes involved in ‘ABCDE model’ of flower development.

Class	Gene Name	Ortholog in ^1^ *Arabidopsis* / ^2^snapdragon (^3^petunia)	Putative Function
A	*EuMADS38* ^a^	^1^ *FUL*/ ^2^ *DEFH28*	Control flowering time, floral meristem identity, and fruit development; paralogs of *AP1*
	*EuMADS49* *^b^*	^1^ *FUL* / ^2^ *DEFH28*	
	*EuMADS6* *^b^*	^1^ *FUL* / ^2^ *DEFH28*	
	*EuMADS66*	^1^ *AP1* / ^2^ *SQUA*	Control floral meristem identity and determine sepals and petals identity
B	*EuMADS28* *^b^*	^1^ *PI* / ^2^ *GLO*	Determine petals and stamens identity
	*EuMADS39* *^b^*	^1^ *AP3* / ^2^ *DEF*	
C	*EuMADS13* *^b^*	^1^ *AG* / ^2^ *FAR*	Determine stamens and carpels identity
D	*EuMADS06* ^a^	^1^ *SHP1*,*2* / ^2^ *PLE*	Regulate ovules development, fruit development and dehiscence
	*EuMADS10* ^a^	^1^ *SHP1*,*2* / ^2^ *PLE*	
E	*EuMADS03*	^1^ *SEP1*,*2* / ^3^ *FBP2*	Co-regulate floral development with A/B/C/D-class genes; Activate B-and C-class genes
	*EuMADS33*	^1^ *SEP3* / ^3^ *FBP2*	
	*EuMADS34* *^b^*	^1^ *SEP4* / ^3^ *FBP2*	
	*EuMADS37* ^a^	^1^ *SEP4* / ^3^ *FBP2*	
	*EuMADS64* ^a^	^1^ *SEP3* / ^3^ *FBP2*	

Note:

^a^ ^b^ represents female-biased expression; represents male-biased expression.

^1,2,3^represent ortholog genes in *Arabidopsis*, snapdragon and petunia respectively.

Abbreviations: AG: AGAMOUS; AP1: APETALA1; AP3: APETALA3; DEF: DEFICIENS; DEFH28: DEFICIENS-Homolog 28; FAR: FARINELLI; FBP2: Floral-Binding Protein 2; FUL: FRUITFULL; GLO: GLOBOSA; PI: PISTILLATA; PLE: PLENA; SEP1, 2, 3, 4: SEPALLATA1, 2, 3, 4; SHP1, 2: SHATTERPROOF1, 2; SQUA: SQUAMOSA.

## 3 Discussion

### 3.1 Genome-wide characterization of MADS-box TFs in E. ulmoides

MADS-box TFs play foundational and indispensable roles in floral organogenesis and flowering of plants (Theien, 2001; Theissen et al., 2018). In present work, 66 MADS-box TFs of *E. ulmoides* were identified (Table 1), they were unevenly distributed across the genome (mainly on 14 chromosomes, Figure 1). The number of MADS-box genes identified in present work (66) is fewer than that in previous study (100) (Wang and Zhang, 2017). This inconsistency may be largely attributed to the alternative splicing of genes (Parenicová et al., 2003; Arora et al., 2007). In *E. ulmoides*, 49 MADS-box genes of the Type II (MIKC) subfamily averagely contained 7.8 exons per gene (Table 1; Figure 5), which likely would generate >49 transcripts due to different exon combinants during mRNA processing. Under such circumstance, prediction of more putative *EuMADS* unigenes solely based on transcriptome data is probable. For example, two putative MADS-box unigenes (*Cluster-47702.5450* and *Cluster-47702.5456*) predicted previously (Wang and Zhang, 2017) were practically derived from the same gene locus of *EuMADS65* (Supplementary Table S3) in present work. In addition, the quality of data assembling (both transcriptome and genome) and approaches employed are certainly essential for MADS-box family screening (Wang et al., 2019; Guan et al., 2021).

The MADS-box TF family has been studied in certain species with bisexual flowers, e.g., tomato (137 genes) (Wang et al., 2019), foxtail millet (89 genes) (Lai et al., 2022) and American beautyberry (78 genes) (Alhindi and Al-Abdallat, 2021), and a few species with unisexual flowers, such as litchi (*L. chinensis*, 101 genes) (Guan et al., 2021), hop (*H. lupulus* L., 65 genes) (Gutierrez et al., 2022) and *Populus trichocarpa* Torr. & A. Gray ex Hook (105 genes) (Leseberg et al., 2006). Interestingly, the size of MADS-box TF family identified in *H. lupulus* (65) (Gutierrez et al., 2022) is very close to the numbers of EuMADS TFs we found here. Neither male nor female flowers in *E. ulmoides* comprise sepal and petal, only have bracts encompassing 8–12 stamens (male) or one pistil (female) (Wang et al., 2020). Likewise, in *H. lupulus* the female flowers entirely lack sepal and petal, while male flowers comprise five sepals with five stamens inside (Shephard et al., 2000). Their resembling dioecious sexual system and unisexual floral composition may reflect similar family size of MADS-box TFs in *E. ulmoides* and *H. lupulus*, or *vice versa*.

The 66 EuMADS TFs were categorized into two groups, i.e., Type I (M-type) with 17 members and Type II (MIKC) with 49 members (Table 1) according to phylogenetic relationships (Figure 3). Subsequently, the 17 M-type EuMADS TFs were divided into two subgroups (Mα and Mγ), but no Mβ members were found (Figure 3), same as the result from American beautyberry (*Callicarpa americana* L.) (Alhindi and Al-Abdallat, 2021). Additionally, conserved protein domain analysis confirmed the presence of MADS domain in all the 66 EuMADS TFs and K-box domain in most MIKC-EuMADS TFs (Figure 5), as previously reported in tomato (Wang et al., 2019), physic nut (Tang et al., 2020), and cultivated alfalfa (Dong et al., 2021). Meanwhile, MIKC-*EuMADS* genes had more exons (7.8 per gene on average) than the M-type members (1.5 per gene on average, Table 1 and Figure 5). Promotor *cis*-element analysis also predicted more phytohormone-response elements MIKC-*EuMADS* genes (Figure 6, Supplementary Table S2). All of these results showed more complicated characteristics of MIKC-EuMADS TFs, suggesting more functional diversification of these genes (Ng and Yanofsky, 2001; Bemer et al., 2010; Lin et al., 2020).

The 49 MIKC-EuMADS TFs could be classified into 13 MIKC^C^ subgroups (AG/SHP, AGL6, AGL12, AGL15, ANR1, AP1, AP3/PI, FLC, SEP, SOC1, SVP, TM8, and TT16) that are previously reported (Parenicová et al., 2003; Wang et al., 2019), and one MIKC^*^ subgroup (Table 1; Figure 4). Notably, in *A. thaliana*, there were six FLC members, i.e., *AtFLC* and *AtMAF1-5* (Figure 4, Supplementary Table S5). The *AtFLC* gene encodes a floral repressor to inhibit plant flowering (Theissen et al., 2018). When prolonged cold exposure in winter, the expression of *AtFLC* gene was epigenetically silenced by gene *COOLAIR*, leading to normal flowering, this is known as the vernalization flowering pathway (Hepworth and Dean, 2015). In the vernalization pathway, *AtFLC* represses the expression of *FT* (*FLOWERING LOCUS T*) and delays flowering (Hepworth and Dean, 2015). In certain species, e.g., hop (Gutierrez et al., 2022), foxtail millet (Lai et al., 2022) and litch (Guan et al., 2021), that require no vernalization for flowering, the *AtFLC* homologs are dispensable. As expected, the FLC lineage has been completely eliminated from hop (Gutierrez et al., 2022) and foxtail millet (Lai et al., 2022), and litchi only retained far fewer FLC members (1) compared to that (6) of *Arabidopsis* (Guan et al., 2021). *E. ulmoides* is a deciduous, temperate zone-growing tree, it would not flower without experiencing freezing winter (Wuyun et al., 2018; Li et al., 2020; Qing et al., 2021). It is thus easy to understand the presence of comparable number (5) of *AtFLC* homologs (*EuMADS30*, *31*, *32*, *48*, and *63*) in *E. ulmoides* (Table 1; Figure 4). Further functional analysis of these *EuMADS* genes would facilitate to understand the flowering regulation of *E. ulmoides*.

### 3.2 Essential position of floral organ ABCDE model-related genes in sex determination pathway of E. ulmoides

Sex determination of dioecious plants are usually reflected in floral phenotype, which are related with the expression of floral organ ABCDE model-related genes (Cronk and Muller, 2020; Zhang et al., 2022). In fig (*Ficus hispida* L.), a MADS-box gene *FhAG2* was found locating at the sex determination region of Y chromosome as potential male activator (Zhang et al., 2020). Likewise, studies on cycad (*Cycas panzhihuaensis* L. Zhou and S. Y. Yang) (Liu et al., 2022), papaya (*Carica papaya* L.) (Yu et al., 2007), ginkgo (*Ginkgo biloba* L.) (Liao et al., 2020; Gong and Filatov, 2022), *Nepenthes* pitcher species (Scharmann et al., 2019), and *Silene latifolia* Poir. (Matsunaga et al., 2003). also suggested floral MADS-box genes were putative sex determination genes to exhibit sex-linked inheritance. In *P. tremula* L., *ARR17* was a feminizing factor (Müller et al., 2020), CRISPR/Cas9-induced *arr17* mutants upregulated B-class gene *popPI* to turn female individuals into male (Leite Montalvão et al., 2022). Similarly, in *Diospyros lotus* L., B-class gene *DlPI* was negatively controlled by the feminizing gene *MeGI* and a regulation cascade of *OGI*-*MeGI*-*DlSVP*-*DlPI* was proposed for sex determination in persimmon (Akagi et al., 2014; Yang et al., 2019).

Herein we found that 24 out of the 66 *EuMADS* genes were DEGs between the male and female flowers, meanwhile two *EuMADS* genes were also DEGs between the male and female leaves (Figure 7, Supplementary Table S3). It is worth noting that most (11/14, 78.6%) of the floral organ ABCDE model-related genes showed sex-biased expression patterns in *E. ulmoides* (Figures 7A, 9). Unisexual flowers can be produced when the spatiotemporal expression domain of B- and C-class MADS-box genes are overlapped (Irish, 2017; Jabbour et al., 2022). As expected, two B-class genes we identified in *E. ulmoides*, i.e., *EuMADS28* and *EuMADS39* (Table 1; Figure 4) were both highly expressed in the male flowers based on transcriptome analysis and qRT-PCR validation (Figures 7A, 9). Protein interactive analysis also predicted protein-protein interactions among B/C/E-class genes (Figure 8). In particular, *EuMADS39* was expressed specifically in the male leaves too (Figure 7B), suggesting its constitutive-expression pattern in male individuals. This gene was previously described as *EuAP3* (Wang and Zhang, 2017) by our group or *EuDEF* (Zhu, 2019) by another team, consistent with its functional annotation here (Table 2). More importantly, *EuMADS39* was also physically adjacent to a male-linked molecular marker (MSL4) on Chr10 (Wang et al., 2020) and exhibited a persistently male-biased expression pattern during the whole period of floral bud differentiation (Qing et al., 2021). All these above clues lead us to hypothesize that B-class *EuMADS* genes, particularly *EuMADS39*, seem to participate in sex determination pathway of *E. ulmoides* as (in) direct players. Gene function verification of *EuMADS39* is urgently needed in the future.

According to the classical ‘ABCDE model’, we know that A/C/D/E-class MADS-box genes direct the organogenesis of (sepal and petal)/(stamen and carpel)/ovule/the whole floral organ, respectively (Theien, 2001; Theissen and Saedler, 2001; Soltis et al., 2007). As expected, D-class genes (*EuMADS06* and *EuMADS10*) both showed female-biased expression pattern (Figures 7A, 9), in line with its role in ovary development. However, expression patterns of certain A/C/E-class *EuMADS* genes unfit their identity as described in ‘ABCDE model’. For example, *EuMADS65* (A-class gene), homologous to *FUL* in *Arabidopsis* and *DEFH28* in snapdragon (Table 2), was specifically expressed in male individuals, both in the flowers and leaves (Figures 7, 9). In *A. thaliana*, *FUL* is paralog of *AP1* and plays multiply functions in controlling flowering time, meristem differentiation, and flower development, etc*.* (Ng and Yanofsky, 2001; Parenicová et al., 2003; Soltis et al., 2007). There are different flower arrangements between male and female individuals of *E. ulmoides*, with the male flowers forming a capitulum inflorescence while the female flowers being solitary on branch (Wang et al., 2020; Qing et al., 2021), similar to that in persimmon (Akagi et al., 2014; Yang et al., 2019) and kiwifruit (Akagi et al., 2018; Varkonyi-Gasic et al., 2021). It is thus possible for A-class *EuMADS65* functioning in determining the male flower inflorescence.

Notably, neither sepals nor petals were present in *E. ulmoides* female flowers (Wang et al., 2020; Qing et al., 2021). Given that A + B + E genes determine the petal identity (Theien, 2001; Theissen and Saedler, 2001; Soltis et al., 2007), the silence of B-class genes (*EuMADS28* and *EuMADS39*) in *E. ulmoides* female flowers (Figures 7A, 9) may partly contribute to the absence of petals. Meanwhile, the four A-class genes (*EuMADS38*, *EuMADS49*, *EuMADS65* and *EuMADS66*) showed varied expression patterns between the male and female flowers of *E. ulmoides* (Figures 7A, 9) and different protein-protein interactions (Figure 8), suggesting their functional differentiation during evolution. This may lead to the loss of ancestral function of A-class genes in determining sepal and petal identity, resuling in the absence of both sepals and petals in *E. ulmoides* female flowers. Additionally, the number of stamens (8–12) in each male flower is much larger than that of carpel (only 1) in each female flower of *E. ulmoides* (Zhu, 2019; Wang et al., 2020), as in poplar (Xue et al., 2020) and willow (Wang et al., 2022). This probably leades to higher expression level of C-class gene (*EuMADS13*) in male flowers than that in female flowers (Figures 7A, 9). Further functional analysis of A-class genes (e.g., *EuMADS65*) and the C-class gene (*EuMADS13*) is required to understand their roles in sex determination pathway of *E. ulmoides*.

## 4 Materials and methods

### 4.1 Identification of MADS-Box TFs, genome mapping and synteny analysis

The high-quality haploid genome of *E. ulmoides* (Li et al., 2020) was downloaded from NCBI (https://www.ncbi.nlm.nih.gov/data-hub/genome/GCA_016647705.1/), together with a male reference genome (Wuyun et al., 2018) from Genome Warehouse (https://ngdc.cncb.ac.cn/gwh/Assembly/13/show). The MADS-box protein sequences of *A. thaliana* (https://www.arabidopsis.org/) and rice (*Oryza sativa* L., http://rice.plantbiology.msu.edu/) were separately downloaded on 13 June 2022 (Additional Data1). Two methods were independently used to identify MADS-box TFs in *E. ulmoides.* In one approach, all the annotated *E. ulmoides* proteins were reciprocally BLASTed (Camacho et al., 2009) with AtMADS and OsMADS TFs, the best hits (score value ≥100 and e-value ≤ 1e^−10^) yielded were selected as candidate MADS-box TFs. In the other method, conserved SRF (PF00319) or MEF2 (PF09047) domains were retrieved from Pfam (Mistry et al., 2021) and used to identify the candidate MADS-box TFs in *E. ulmoides* by Hidden Markov Model (HMM) search in HMMER v. 3.0 (Potter et al., 2018). The shared sequences resulted from the above two methods were further validated by Conserved Domain Database of NCBI (CDD, http://www.ncbi.nlm.nih.gov/cdd/) and Simple Modular Architecture Research Tool (SMART, http://smart.embl-heidelberg.de/) (Letunic et al., 2012). Eventually, 66 non-redundant MADS-box TFs were obtained and named as EuMADS01 to 66. The online tool ExPasy (https://web.expasy.org/compute_pi/) was employed to determine their physicochemical properties, including length of amino acid (aa), isoelectric points (PI) and molecular weight (MW, kDa). Phosphorylation sites, i.e., Ser, Tyr and Thr sites, of EuMADS TFs were counted in Geneious V9.0 (Kearse et al., 2012). Subcellular localizations of EuMADS TFs were further predicted by Plant-mPLoc (http://www.csbio.sjtu.edu.cn/bioinf/plant-multi/) (Chou and Shen, 2010).

Based on GFF3 file of genome annotation (https://www.ncbi.nlm.nih.gov/data-hub/genome/GCA_016647705.1/), the physical positions of 66 *EuMADS* genes were mapped against the assembled 17 chromosomes of *E. ulmoides* (Li et al., 2020). TBtools software (Chen et al., 2020) was used for visualization of genes on chromosomes. The number of *EuMADS* genes per chromosome was then counted and plotted. Moreover, the information of length and gene-density of 17 chromosomes of *E. ulmoides* were obtained from the reference genome (Li et al., 2020). The multiple collinearity scan toolkit X (MCScanX) program (Wang et al., 2012) was performed with default parameters for collinearity analysis among the 66 *EuMADS* genes. Then the circos map was drawn using TBtools software (Chen et al., 2020). Similarly, the homology of these *EuMADS* genes among *E. ulmoides*, *A. thaliana*, and *O. sativa* were also analyzed using MCScanX (Wang et al., 2012). The synteny genome blocks between species were then visualized in TBtools (Chen et al., 2020).

### 4.2 Phylogenetic analysis and classification of EuMADS TFs

MADS-box TFs from *A. thaliana* (Parenicová et al., 2003), rice (*O. sativa*) (Arora et al., 2007) and tomato (*Solanum lycopersicum* L.) (Wang et al., 2019) were used for the classification of MADS-box family in *E. ulmoides*. Full-length protein sequences of AtMADS and EuMADS (Additional Data1, Supplementary Table S5) were aligned by using MUSCLE program (Edgar, 2004) in Geneious V9.0 (Kearse et al., 2012) with default parameters. An unrooted maximum likelihood (ML) phylogenetic tree was constructed by MEGA-X software (Kumar et al., 2018), with 1,000 bootstrap replicates under JTT model. The gaps and missing data were manually checked and processed by partial deletion (Wang and Zhang, 2017; Wang et al., 2018). Type I (M-type) and Type II (MIKC) MADS-box TFs of *E. ulmoides* were determined based on the phylogenetic tree of AtMADS and EuMADS.

Subsequently, to better categorize the EuMADS TFs of MIKC subfamily (MIKC-EuMADS), MIKC protein sequences of tomato (MIKC-SolyMADS), *Arabidopsis* (MIKC-AtMADS) and rice (MIKC-OsMADS) were all utilized. MIKC-SolyMADS were downloaded from PlantTFDB v5.0 database (Jin et al., 2014) and aligned with MIKC-AtMADS, MIKC-OsMADS and MIKC-EuMADS (Additional Data1, Supplementary Table S5) using the MUSCLE tool (Edgar, 2004). MEGA-X software (Kumar et al., 2018) was applied to infer the ML tree under JTT model with a bootstrap value of 1,000. The resulted ML tree was visualized in FigTree viewer (http://tree.bio.ed.ac.uk/software/figtree/). The MIKC-EuMADS TFs were then classified into different subgroups following the phylogenetic relationships with MIKC-AtMADS, MIKC-OsMADS and MIKC-SlMADS. The subgroups were named following available classification systems in *Arabidopsis* and tomato (Parenicová et al., 2003; Arora et al., 2007; Wang et al., 2019).

### 4.3 Protein domain, gene structure, and cis-element analyses of EuMADS TFs

Conserved protein domains of the 66 EuMADS TFs were blasted in the NCBI-CDD website (https://www.ncbi.nlm.nih.gov/Structure/bwrpsb/bwrpsb.cgi) (Marchler-Bauer et al., 2015). The online Multiple Em for Motif Elicitation (MEME) program (http://meme-suite.org/tools/meme) (Bailey et al., 2009) was also employed to analyze the protein motifs of EuMADS TFs. Parameters for MEME search were set as follows: maximum number of motifs = 20, motif width = 6–60, number of repetitions = 0/1, according to our knowledge of plant MADS-box proteins (Wang et al., 2019; Alhindi and Al-Abdallat, 2021; Dong et al., 2021; Gutierrez et al., 2022). Moreover, exon-intron structures of the *EuMADS* genes were constructed based on the alignment between the full-length coding sequences (CDS) and the corresponding genomic sequences (Wuyun et al., 2018; Li et al., 2020). The annotation information for each *EuMADS* gene was also extracted from the GFF3 files (PRJNA599775 and PRJCA000677) of the genome data to verify the constructed gene structure. The protein domains, motif composition and gene structure of EuMADS TFs were then visualized using TBtools (Chen et al., 2020).

The 2,000 bp promotor sequences upstream the initiation codon (ATG) of each *EuMADS* gene were extracted from the genome data (Wuyun et al., 2018; Li et al., 2020) in TBtools (Chen et al., 2020). The online PlantCARE tool (http://bioinformatics.psb.ugent.be/webtools/plantcare/html/) was then performed for the *cis*-acting regulatory element (CARE) prediction. The *cis*-elements related to phytohormone responsiveness, defense and stress responsiveness, light responsiveness, low-temperature responsiveness, wound responsiveness, anaerobic induction, and circadian control were analyzed. In the phytohormone-response *cis*-elements, ABRE was involved in ABA-responsiveness, AuxRR-core and TGA-element were involved in IAA-responsiveness, O2-site was involved in zein-metabolism, GARE-motif, TATC-box and P-box were involved in GA-responsiveness, TGACG-motif and CGTCA-motif were involved in MeJA-responsiveness, and TCA-element was involved in SA-responsiveness (Alhindi and Al-Abdallat, 2021; Ye et al., 2022) The location of *cis*-elements in promoter region of each *EuMADS* gene was visualized in TBtools (Chen et al., 2020). *Cis*-elements associated with phytohormones (ABA, IAA, zein, GA, MeJA, and SA) were also counted and plotted in TBtools (Chen et al., 2020).

### 4.4 Tissue- and sex-specific expression analysis of EuMADS TFs by RNA-seq and protein-interaction prediction

To reveal the expression patterns of MADS-box genes in both vegetative and reproductive tissues of *E. ulmoides*, leaves and flowers from multiple individuals of male and female were sampled for transcriptome analysis. The RNA-seq data of male and female leaves with three biological replicates were from our previous work (Wang and Zhang, 2017). Male and female flowers from the same corresponding trees that offered leaf tissues were collected in April 2021. Flower samples were named as Eu_Male1, Eu_Male2, Eu_Male3 and Eu_Female1, Eu_Female2, Eu_Female3. Total RNA was extracted using RNeasy Plant Mini Kit (74904, Qiagen, German). mRNA library construction and Illumina sequencing were conducted according to manufacturer’s procedures at BGI Technologies Corporation (Shenzhen, China).

About 6 Gb clean reads were generated for each sample after quality control. These reads were then used to quantify the expression level of each *EuMADS* gene using RSEM software (Li and Dewey, 2011). FPKM (fragments per kilobase of transcript per million mapped) values of *EuMADS* genes were calculated from the uniquely mapped reads (Zhao et al., 2021). For each tissue, i.e., flower and leaf, reads from male and female individuals were batched into one dataset, respectively for differential expression analysis with DESeq R package v1.10.1 (Anders, 2010). Benjamini and Hochberg’s approach was employed to adjust the resulting *p*-values for false discovery rate (FDR) control (Benjamini and Hochberg, 1995). Genes with log_2_ (fold change value) ≥ 1 or ≤ −1, and padj < 0.05 were considered as differentially expressed genes (DEGs) (Wang and Zhang, 2017; Guan et al., 2021), i.e., male- or female-biased expression. Flower RNA-seq data newly generated in this study were deposited in SRA database (accession number: PRJNA399774).

Given that MADS-box proteins often form dimers or polymers with each other to play roles in plant growth and development (Theissen and Saedler, 2001; Theißen et al., 2016; Ruelens et al., 2017). The protein sequences of 66 EuMADS TFs were submitted to the STRING website (https://cn.string-db.org/) (Szklarczyk et al., 2015) for protein interactions prediction. Then the protein-protein interaction network was exported and beautified by the Adobe Illustrator tool (McLean, 2002). The protein interactions of the identified 14 A/B/C/D/E-class *EuMADS* genes was also predicted in the same way.

### 4.5 Sex-biased expression analysis of floral organ ABCDE model-related genes by qRT-PCR

Ten flower samples were respectively collected from five male and five female *E. ulmoides* individual trees growing on the campus of Northwest A&F University, Yangling, China (34°16′56″N, 108°04′27″E) in April 2021 for quantitative Real-Time PCR (qRT-PCR) analysis. The collected male and female flowers was about to open. Because there are no sepal and petal in either male or female flowers (Wang and Zhang, 2017; Wuyun et al., 2018; Zhu, 2019), only stamens and pistils were isolated and immediately immersed into liquid nitrogen before stored at −80°C until use. Total RNA extraction and cDNA synthesization were performed using RNeasy Plant Mini Kit (74904, Qiagen, German) and SuperScript™ IV VILO™ Master Mix (Thermo Fisher, United States of) respectively following the manufacturer’s instructions.

qRT-PCR analysis was conducted according to the manuals of Hieff qRT-PCR SYBR Green Master Mix (YEASEN, Shanghai, China) on a LightCycler 480 II Real-Time PCR Platform (Roche, Germany). The reaction system (20 μL) consisted of 10.0 μL of qRT-PCR Mix, 1.0 μL cDNA, 0.4 μL forward primer, 0.4 μL reverse primer, and 8.2 μL of ddH_2_O. *EuGAPDH* gene was used as internal control for data normalization (Liu et al., 2018). 2^−ΔΔCT^ method was applied to calculate the relative gene expression of 14 A/B/C/D/E-class *EuMADS* genes (Livak and Schmittgen, 2001). Primers used for *EuMADS* and *EuGAPDH* genes (Supplementary Table S6) were designed using the online program Primer3 (http://bioinfo.ut.ee/primer3-0.4.0/primer3/). Five biological replicates and two technical replicates were carried on for each gene. Differences of gene expression level between male and female flowers were analyzed *via* ANOVA followed by Student’s t-test in SPSS software (v.24, IBM).

The identified 14 A/B/C/D/E-class *EuMADS* genes were also functionally annotated by the online BLAST in NCBI database (https://blast.ncbi.nlm.nih.gov/Blast.cgi). The most similar genes in model plants (*Arabidopsis,* snapdragon/petunia) of flower development studies (Theißen et al., 2016; Irish, 2017; Ruelens et al., 2017) were screened as orthologous genes to predict gene function.

## 5 Conclusion

In this study, genome-wide comprehensive analyses of the MADS-box TF family in *E. ulmoides* were performed for the first time. A total of 66 EuMADS TFs were identified, including 49 Type II (MIKC) members and 17 Type I (M-type) members. All the previously recorded 13 MIKC^C^ subgroups were detected in *E. ulmoides*, but the Mβ lineage was missing. More complicated protein-motif composition, exon-intron architecture, and phytohormone-response *cis-*elements were found in MICK-EuMADS TFs, suggesting more diverse biological functions in these genes. Furthermore, tissue- and sex-specific transcriptome analyses revealed 24 DEGs between male and female flowers, and two DEGs between male and female leaves. qRT-PCR validation of the 14 *EuMADS* genes involved in the floral organ ‘ABCDE model’ showed six male-biased A/B/C/E-class genes, five female-biased A/D/E-class genes and three non-biased A/E-class genes in *E. ulmoides* flowers. Notably, the B-class gene *EuMADS39* (ortholog of *AtAP3*) and the A-class gene *EuMADS65* (ortholog of *AtFUL*) were significantly expressed in the male individuals, no matter in flower or leaf tissues. In short, these results suggested essential roles of floral organ ABCDE model-related MADS-box TFs in sex determination of *E. ulmoides*, as demonstrated in dioecious poplar (Leite Montalvão et al., 2022), persimmon (Yang et al., 2019) and kiwifruit (Ye et al., 2022) recently.

## Data Availability

The datasets presented in this study can be found in online repositories. The names of the repository/repositories and accession number(s) can be found in the article/Supplementary Material.
