# Supplementary material for: Genome-wide identification and expression analysis of MADS-box transcription factors reveal their involvement in sex determination of hardy rubber tree (Eucommia ulmoides oliv.)
Source: Front Genet. 2023 Feb 21;14:1138703. doi: 10.3389/fgene.2023.1138703 (PMC9988917; doi:10.3389/fgene.2023.1138703)
Supplement: Supplementary file 1 [file DataSheet1.DOCX]

#nucleotide sequences of MADS-box genes in Eucommia ulmoides

>EuMADS01

ATGGCGAGAGAGAAAATACAGATCAGGAAGATCGATAACACGACGGCGAGGCAGGTCACTTTCTCGAAGAGGCGAAGGGGGCTTTTCAAGAAGGCGGAGG

AGCTATCGGTTCTCTGCGATGCGCACGTTGCCCTCATCGTCTTCTCGTCCACCGGGAAGCTCTTCGAGTATTCCAGCTCCAGCATGAAGGAAATACTAGA

AAGGCATAATTTGCATTCAAAGAATCTTGAGAAGCTTGAACAACCATCTCTTGAATTGCAGCTAGTAGAGAGCAGTGACTGCACTAGATTAAACAAGGAA

GTTGCTGAGAAAACTCATCAACTGAGGCGGATGAGAGGAGAGGACCTTCAAGGTTTAAGCATCGAAGAATTGCAGCAATTAGAGCGGTCTCTTGAAGTCG

GATTGAGCCGCGTGATAGAGAAAAAGACTGAGAAAATCATGAAAGAGATCAGTCATCTTCAGCAAAAGGGATTGCAACTCATGGAGGAAAATGAGCTACT

GAGACAACAAGTGATGAATGTAAGTAATGGTCGAGATCCGGTTGGAGCTGATTCCTATAATGTGGTATATGAGGAAGGTCAATCATCGGAGTCTATTACC

AACGTCTGCAACTCCTCGGAGCCTCCACAAGACTCTGAAAGCTCAGACACTTCCCTCAAGTTGGGCTTATTTTACATCCGATGTAAGATAAACCCAACAA

TCTCCTCCTTACACATCGAGCCTTTGGTTGGCTTACCCTCAGATAGCCTCTCCCTTTGTGGCTATTAG

>EuMADS02

ATGACGATTGGAAAGAAAAGAAAGAATTGCATAATCAAGAAGACGATGGAGCTCTCCATCCTTTGCGACGTCAAGGCCTGCATAATTATATCCAACGCCG

ACGGAAAATTCGAGACGTGGCCGGAGAATCGGACGGAAGTGGAGGGCGCGATCGGTGCCTACTTCAAGGCCATGAAAGAAGGGAGTAGATTCAGAAAATC

GAAGGGTCAAAAGAGTAATGTTACTGCGGAGCTTTCGACGGGAACTCGAACGGAGAACACCGGTGGTGTAGTCGGCGAGGGAGCTTTGAAGAAGGTTTTC

GGTGATTTGGATGATGGATTTCTCGACGGCTTGTCGATGGATTCTCGAAAGAGTTTCTTGATGGCGATGGAGTCGAAGATTAAAAAGATAGATAAGAGGC

GAGAGTTCTTGAAGAATGGCGGTCTGCAGGGTAACGAACAGAAACTGTTTGAAGAAATTCTACCCAAAAGCCCTTTGTCGTTCCCATATGGCGATTGGGG

AGGAAACATATTTGAGATTTTCCCAGAAATTTATGAACCATTGCCGACTGATTTCGAAGCCGAAAACATCGGGGACATCTACGAGACTTCCAGTTCGCCG

CTGGCGAATTTCTTGAATGACTCGGTAAACCCTGATGCTGGAATTACTGTCGGCGGTAACCAATGGATTCCGCCTCTTGACGATGTAAGTTTTGATTGGA

CGACGCACGAGATGATTCTTAATTGA

>EuMADS03

ATGGGTAGGGGAAGAGTGGAGCTGAAGAGGATAGAGAACAAAATAAATCGGCAGGTGACATTTGCAAAGAGAAGAAATGGGTTGCTCAAGAAAGCTTATG

AGCTCTCGGTTCTCTGTGATGCCGAAATTGCCCTTATCATCTTCTCCAGCAGAGGCAAGCTCTATGAATTCTGCAGCACCTCCAACATGCTCAAGACGCT

CGAGCGATACCAAAAATGCAGCTACAGCACGGCGGAGGTCGACCAGGCGGGCAAAGAGATGGAGGAAAGCAGCTATAGAGAATACATGAAGCTGAAAACC

AAGTATGAGTCGTTGCAGCAATATCAAAGGCAGTTGCTTGGAGAAGAATTGGGACCCCTTAACATAAAGGAGCTAGAGCAGCTTGAGCATCAACTTGATT

CATCCTTGAAGCACATTAGATCCACAAGGACCCAAACTATGGTTGAACAGCTTGCAGATCTTCAGGCCAAGGAGAAGATGTGGCTTGAGGCTAACAAGTC

ACTAGAGAGGAAGCTTGAAGATATATATGCAGAAAATCAGCTTCCTCCACCATGGGCAGGTGTAGAGCAAAGCAGTTCATTTGAACCCCACCACCATCAG

CAGGCTCACTCTCAAGCCTTCTTTCAACCCCTGCAATGTGATCCCACTTTGCAGATAGGGTATAATCAAGTAGGTTCAAGCCATATGGCAACTTCAAGTC

ATGCCCAAAATGTCAATAACTTTCTTCCAGGGTGGATGCTCTAA

>EuMADS04

ATGGGGAGAGGAAAGATTGTGATAAAGAGGATCGACAATTCAACGAGTAGGCAGGTGACGTTCTCGAAGAGGAGAAACGGATTGCTGAAGAAGGCGAAGG

AGCTGGCGATCCTATGCGATGTGGAAGTCGGAGTCATGATCTTTTCCAGCACGGATCGTAGAAGTGGATCGGAGTTGAGCGAACCACCAGTCCACCGAAT

TGAGAAATTGGAGGGCGATTTTGTCGGAGGCCGAGAAATCAGAGGGCGAATTTGTCGAAAGCTGAAAGCGTTGATGGCGGAACGCTGA

>EuMADS05

ATGATTGTCGCCCAACCACCGCCGTGCCTGCACACGAAGAAACCACCGCCGAAGTCAAGGATATTCTTTATCTGGAAGTCAAGGAAGTTAGGGATGGGGA

GAGGAAAGATTGTGATAAAGAGGATCGACAATTCAACGAGTAGGCAGGTGACGTTCTCGAAGAGGAGAAACGGATTGCTGAAGAAGGCGAAGGAGCTGGC

GATCCTGTGCGATGTGGAAGTCGGAGTCATGATCTTTTCCAGCACGGATCGTAGAAGTGGATCGGAGTTGAGCGAACCACCAGTCCACCGAATTGAGAAA

TTGGAGGGCGATTTTGTCGGAGGCCGAGAAATCAGAGGGCGAATTTGTCGAAAGCTGAAAGCGTTGATGGCGGAACGCTGA

>EuMADS06

ATGGCGTTTCGCGATCAAGAGACTGAGGCCTCTTCTCAGAGGAAAATGGGGAGAGGGAAGATAGAGATCAAGAGGATTGAGAACACGACCAATCGACAAG

TCACTTTCTGTAAGCGTCGCAACGGTTTGCTCAAGAAGGCCTACGAGTTGTCCGTGCTTTGTGATGCCGAGATCGCCCTCATCGTCTTCTCCAGCCGCGG

CAGGCTTTACGAGTACTCCAACCACAGTGTTAGGTCGACAATTGATAGGTACAAGAAAGCGTGTGTTGATTCCACCAGTTCAGTTACTGTTTCTGAAGCT

AACACCCAGTTTTATCAGCAAGAATCCACCAAACTTCGGAGACAAATCCGAGACATGCAGAACTCCAACAGACACATTCTGGGTGAGGCCATTAGCTCTT

TATCCTTCAAAGAACTCAAGAATCTGGAAGGTAGATTGGAGAAGGCCATCGGAAGAATTCGTTCGAAGAAGAATGAGATGCTGTTTGCCGAAATTGAGCT

CATGCAGAAGAGGGAGATCGAGATGCAAAATTTTAATATGTACCTCCGAGCAAAGATAGCTGAGAACGAGAGAGCGCAGCAGCAAATGAACTTGATGCCT

ACAGAAGAAGAAGCGGCGGCCGGGAGATCAGATTATCATCAGATTTCGTATGATGCTCGGAATTTCCTGCCCGTAAATCTCCAACTGGAACCTAGCCACA

CTCAGTATTCTCGCCATGGCGAAACCACTCTCCAGCTTGTCTAA

>EuMADS07

ATGGGGAGGGTTAAGCTGAAGATAAAGAGATTAGAGAGCACTAGCAATCGGCAGGTAACTTATTCAAAGCGGAGGAGTGGAATTTTAAAGAAAGCTAGAG

AATTGTCGATATTATGCGACATTGACATTATACTCCTAATGTTTTCACCAACTGGAAAGCCTACATTATTTCGAGGAGAACACAGCAACATTGACGAGGT

TATTGCAAAATTTTCTCAGTTGACTCCTCAAGAAAGGGCAAAAAGGAAGATGGAGAGCCTTGAAGTCTTGAAGAAAACTTTTAAGAAGCTTGATCATGAT

GTAAATGTACAAGACTTTCTAGGCCAAAGCACTCAGTCGGTTGAGGAATTGAGCCACCATGCAAGGACTTTGAGAGCTCAAGTTGAAGAAGTACATAAGA

GACTAAGCTTTTGGAGTAATCTCGAAAAGATTGAAGATATTGAACATCTAGGACAAATGGAAGATTCTTTGAGGGAATCACTTGGCCGAATCCGCATACA

AAGTGAAAATTTTGGAAAACATCAACTTATGCCGCTCGATTGCAGTACCCAGTTCCAACATGGGATGCATTTGCCGATGATGATAGGCGGTAGTACACAA

GAAGCCCAAACGCTCTCATGGCTTCCCAATAATGAGAATCACCATATGATATTGTCTGAGGAACCAAATTTTCTTCTTCAAAGGGAACTTGAGTGTTCAA

GAGATGCCACACTTCCGACCTATAGTGGTTTCTTTTGCAATGGCAAGCAAATAGATATTAACAACACAGGACAGGTTGAAGATACAAGGCAAGATGGTGT

TACCTTGAATGATTGGAATCACACTGCATGCTTGAGACAAAAACTCAACGAGGAATTTGCTTACAATCCGTTTAGTAATCTAGAGTTGCAAGAACCGGAG

AAATTGAAACCTGGGACGGATATTAACTTGCAACAAAACTCGTTCGATTATCAAATTAACGGCAATTTTGAACTACCTAGACCTGCCTACAGCAATGTGC

CTGTTTGGGTCACTCCATCTGGACCTTGTGGAATTACCATGTTTAATGAGAAAGCATACTCTCAGCAACCAACTCAACTTTAA

>EuMADS08

ATGGGTACCGGAAAGAAGAAGATAGAGATCAAGAGGAAGGAGAAAGAGTCGGCAAGGGTGGTCACTTTCAGCAAGAGACGCACCGGCCTGTTCAAGAAGG

CGGGCGAGTTCCACCAGCTCACCGGTACCCACGTCGCCATCATGGTGTTCTCGCCGGCCGGCCGGCCGTACACTCACAGCGAACCCTCCTCCTTCGAGGC

CATCGCCCAGCAGTACCTGAAGTCAGGCTCCGGAGATGATGATGATCATCAATGGAGGAGTTTCATGGACTCAATTCGGCCTGAATCAATGGAGACCGAG

GGCGATCTTCTGGAAATGAAGATGGCTTTGGAAGACCTTCGAAAGAAGTTGATTCAGAAGGCGGAGGATGAGTTCATAGCCTCATTATTGGCTTTGGAAA

ACCCTAAAGATTAG

>EuMADS09

ATGAAGAGAATTGAGAACCCGTCGTCTCGTCAAGTTACATACTCAAAGCGCAAAGATGGACTTGTCAAGAAGGCTAACGAATTATCTGTTCTGTGTGATA

CTGATGTTGCTCTTATTATGTTTTCTCCTACTGGTCGATTGACTAGCTTTGCTAGAAATGGAAGGGTTGAGGATATTTTTCTTCGCTTTGTCAATCGTCC

CGATGAATTGAAAGGGGGACCAATTTGGAATGAAGAGTATCTATCCCTGAGGCTCAAGCAGTTGAAATACGAAGGAGAAATGCTCGATAAGATTTCAACA

ATAGAGGCTCTTGAGGAAAAGCTTGATAAGCTTAATCGACGACAACGTGAGGCACATGAAAAAATGAGGTATTATGATCCGGATGTGGAGAAGATTGGTT

CAGTTCTTGAGGCTGGAGTTCACCAGCAGTTCCTTTCTTGTGCACTTCAACGAGTTGAACTATCGAAAGCCAAGTTGTTAGGCAGTGGGAATGTGTCACA

AAGGCCAGAAAGTATTGAGGTAGCTACAGGCTATACAGAGGATACGTCTTCATTCACCAACGAGTCTTCAGATTCAAACCGAAATAGGGTTCGACCACCG

AATGAGCTTAATGATGAAGGAACACATCAACCGGCGGGACCCCATTTAAGCCTTGGATTCATACAGGCACAGAAGCAGTGGAAACTCCTTGGGTGA

>EuMADS10

ATGGAGTTTTGCAATCAAGAATCTGAGATTTCACCCCACAAGAAAATGGGGAGAGGAAAGATCGAAATCAAGAAGATCGAGAACACGACAAACCGGCAAG

TGACCTTCTGTAAGCGCCGTAACGGCCTCCTCAAGAAGGCCTACGAGTTATCAGTGCTGTGCGATGCTGAAGTGGCCCTCATCGTCTTCTCCAACCGAGG

CCGCCTTTATGAGTACGCTAGCAACAGTGCAAGATCTACGATAGAAAAGTACAAGAAATCTTGTATTGATTCCACAAGTATAGGATCTGCATTCGAAACT

AACACTCAGTTCTACCAACAAGAAGCAACCAAACTACGACGACAAATTCGTGATATGCAAAATTCAAATAGACATATTATGGGTGAGGCTCTTAGCTCTT

TGTCGTTCAAGGATATGAAGAATTTGGAAAGCAGACTGGAAAAGGCCATCGCGAGAATTAGATCAAAAAAAAATGAAATGTTGTTCGCGGAAATTGAGCA

TATGAAAAAGAGGGAGATTGAATTACAAAATTCTAACATGTATCTACGAGCAAAGATAGCAGAGAATGAGAATGTACAACGGAAAATGAACGTGATGTCG

GGGTATGAGTATCAGTCTGACATGACTTCACAACCATTTGATGGTAGGAATTTTATTCCAGTAAACCTCCTTGAGCCTAACCCCAACTACTCTCGACAAG

ACCAAACAGCTCTCCAACTCGTTTAA

>EuMADS11

ATGGAAATGGTTAGAGGGAAGATTCAGATAAAGAGGATTGAGAATTCAACAAGCAGACAGGTGACGTTCTCAAAGCGCAGAACTGGTTTGTTGAAGAAAG

CTTATGAGCTCTCAGTTCTTTGTGAAGCTGAAGTTGGTTTGGTCATTTTCTCTCAAAATGGAAGGCTCTATGAGTACTCAAGTTCCGACATGCGTATGAT

ATTTAAAAGGTACCTTGAAATATCAGCCAAAGAAGACCAAAGCAGCAACCCTGACACCCAACGGCAGCTTCAGCATTTGAAGCATGAATCTGCAAAATTG

GCCAAGAAGATAGAGCACCTTGAAAATTCTCAAAGGAAGCTCTTAGGGCAAAATTTGGCAACATGTTCCATAGATGAACTTCAAGAGATAGACGGCCAGT

TGGAGCGAAGCTTGAAAAACATCAGGCTTAAAAAGGCTCAGGTATTCACAGAGGAAATTGATAGATTAAAAGAAAAGGAGAGACTTTTGCTTGAAGAAAA

TGCAAGATTATGTGACGAGTGTGCGGTGAAGCAATTGAAGCCACCAACAAAAGAGAAAGAAATTGCAAAATGCAACCACAATGCAGAAGTGGAGACTGAA

CTGTTTATTGGCTTACCTGAGATGCGTTGCTCTTAG

>EuMADS12

ATGGTTAGAGGGAAGATTCAGATAAAGAGGATTGAGAATTCAACAAGCAGACAGGTGACGTTCTCAAAGCGCAGAAATGGTTTGTTGAAGAAAGCTTATG

AGCTCTCAGTTCTTTGTGAAGCTGAAGTTGGTTTGGTCATTTTCTCTCAAAATGGAAGGCTCTATGAGTACTCAAGTTCCGACATGCATATGATATTTAA

AAGGTACCTTGAGATATCAGCCAAAGAAGACCAAAGTAGCAACCCTGAAACCCAACAGCAGCTTCAGCATTTGAAGCATGAATCTGCAAAATTGGTCAAG

AAGATTGAGCACCTTGAAAATTCTCAAAGGAAGCTCTTAGGGCAAAATTTGGCAACATGTTCCATAGATGAACTTCAGGAGATAGACGGCCAGTTGGAGC

GAAGCTTGAAAAACATCAGGGTTAAAAAGGCTCAGGTATTCACAGAGGAAATTGATAGATTAAAAGAAAAGGAGAGACTTTTGCTTGAAGAAAATGCAAG

ATTATGTGACGAGTGTGGGGTGAAGCAATTAAAGCCACCAACAAAAGAGAAAGAAATTGCAAAATGCAACCGCAATGCAGAGGTGGAGACTGAATTGTTT

ATTGGCTTACCTGAAATGCGTTGCTCTTAG

>EuMADS13

ATGGATTTTCAAGATCAATCAAGGGAGATCTCTCCTCAGAGGAAAATGGGTAGAGGAAAGATCGAGATCAAACGGATCGAAAACACGACAAATCGACAGG

TCACGTTCTGCAAAAGGCGCAATGGATTGCGCAAGAAGGCCTATGAATTATCTGTTCTATGTGATGCTGAGATTGCCCTTGTTGTCTTCTCTAATCGCGG

TCGACTCTATGAGTATGCAAACGACAGTGTGAAGGCAACAATTGAGAGGTATAAGAAGGCATCCTCAGATTCTCCCAACACAAGCTCAATTTCTGAAGCT

AATGCTCAGTTCTACCAGCAGGAATCCTCCAAACTGCGTGTGCAAATTGCAAATTTGCAGAATCAAAGCAGGTATATGCTTGGGGAATCTCTGAGTTCAT

TATATGTCAAGGATCTCAGGAATCTGGAGTCCAAAATAGAGAGAGGCATTAGCCGAATCCGGTCAAAAAAGAATGAGCTGATGTTTGCTGAGATTGAGTA

TATGAAAAAGAGGGAATTTGAATTGCATAACAACAACCACTACCTCAGAACAAAGATAGCTGAGAATGATAGAGCACAACAGCAGCAGCTACATATGAAT

CTGATGCCAGGGGTTCCCGAATATGAGCTCCCTTTGGCCACTCAGCCACCTTTTGATGGTCGAAACTATCTACAAACCAACGTTAATCAGTACGCCGGCA

GCAGCCGCCAAGACCAGACTGTCCTTCAGTTGGTTTGA

>EuMADS14

ATGACAAGAACGAAGCTCAAACTTGGAGTAATTCCAAGTGAAAGCACAAGGAAATTAACCCTAAAGAGGAGGAGAGCAAATTTGTTCAAGAAAACAGAGG

AGCTATCGGTCCTTTGCGACGTGGAGGCAGGGATTATTGTTTATAGTCCTGAAGAGGAAACTCCGGCAGTTTTTCCGTCGCTGGAGGCGGTGGCGGGGAT

GGTGGCGAGGTTCAAGAGTTTGCCGGAGATGGAGCGGAGCAAGAAGGCGGTGACGAACGAGGGGTATTTGAGGGATAGGGCGGCGTCGGAGGGTGGAAAA

GTGAGGAGCGAGGAGGAGAGGAACGATGAGAAGGAGATGCAAGTGATAATGAATCAATTGATTGGGGGTATGGATTTGAGTGAACTCGATGAGAGGCAGT

TGAATGGGATGTTTGGTTATGCGGCTAGGAGGTTGAAGAGGCTTGAGGTGAGGGACCGGGAGCTGGAGCGGCTTGATCCGCCACTGCCGCCTCCGCCACC

GCCGCCACAGAGTGAGGCAGAGGTGGTGGCTGATAGGGCACAAGGTGGCAGCAGTGGTGGTGGAGATGTGGTTGATCGGTGGTTTGTGGGTGATGAATTG

CCTGATGACCTAAATCAGTCATGGCCTCACATTTTCTCCCCTTGA

>EuMADS15

ATGGCAAAGAAGCCAAGTTTGGGTCGTCAAAAGATTAAAATAGAAAATATACAGACAAAAACTCATTTACAAGTAACCTTCTCTAAACACAAGTCAGGTT

TTTTAAAAAAGGCGAGCGAACAGTGCACGATTTGCGGAGTTGAAATCGCGATAGTAGTGTTTTTACCGGCAGGAAAAGTCTTTTGTTTCGGTCATATCAA

TGTGGATTCAATTGTCGATAAATTCTTGGGACTTAATCGCCCTTTAAATTACATTGATATCCCTAATTTCGTTGAAGCCCATAAGACTGTTCGTATTCCT

GAACTAATTTGTCAACTGACTCAAATTCTCAACGAATTAGAATTTGAGAAAAATCATGGTGAGACGTTTGATCTAATGAGGAAGGCTAGCCGAAATGGCC

AAAACTGGTGGGAGGGCCCAATTGATGAGATGGACTTGAATGAGCTTGTGCAACTTAGGGATTCCATGGAGGAGCTCAAGAAAAATGTTACTAATCAAGC

CAACATGCTTTTGGTTGAGGCCTCTAGGGAAACGTCATCGTTTTAA

>EuMADS16

ATGGTGAGGGGGAAGACCCAGATGAAAAGGATCGAGAACTCAACAAGCAGGCAAGTGACCTTCTCAAAGCGCAGAAGTGGGCTCCTCAAGAAGGCCTTTG

AGCTGTCTGTTCTTTGTGATGCTGAAGTTGCTCTTATCATATTCTCCACTAGAGGCAAGCTGTATGAATTTGCTAGCTCCAGCGCTAGCAAGACCATAGA

ACGGTACGAGAAGAATGCAAATGACCAAGCTGATGTTATCGTAAAAGCAGTTGAAAAAAATATACAGAATTTGAAGGAAGAGACAAGTACCGTGACTAAG

CAGATTGAGCTTCTTGAAGATTCCAAAAGGAAACTCTTGGGAGATGGTTTGGAGTCTTGTACGACGGAGGAGCTAGAAGAGATTGAAGGCCAATTGGAAC

GAAGTTTGAGCAACATTAGGGCTAGAAAGAATCAGTTGCTTAAGGAGAAGATTGAGCAGCTCAAGGAACAGGAGGGGATGCTAGCAAAGGAAAATGAAAA

TTTACGAAAGGTGCAATTTTTGGAACTTTCACTTGCACGGCTGGAATTTCCCACAAGGCAAGTTTTCGAAGTGGAAACCAAACTATTCATGGGACCACCC

TAA

>EuMADS17

ATGACGAGGAAGAAGGTGAAGCTCGCCTTCATCGCCAATGACGCTTCCAGAAAAGCCACCTTCAAGAAGCGGAAGAAGGGTTTGATGAAGAAAGTCAGTG

AGTTAACCACCCTCTGCGGCATTCAAGCCTGCGCGATCATCTACAGCCCCTACGAGTCCCGGCCGGAGGTTTGGCCGAACGTCCTGGGTGCGCAGCAGGT

GGTGGCCAAGTTCAAGACGATGCCGGAGATGGAGCAGAGCAAGAAGATGGTGAACCAGGAGGGGTTCATCCGGCAGAGGATCGGGAAGGCGAGTGAACAG

CTGAAGAAGCTGGCGAAGGAGAATCGGGAGAAAGAGATGACGGAGGTGATGTTCCGGTGTTTGACGGGGTCGGGGCTGCAGAATCTGATGGCGGCGGATC

TGAATGATCTGGGGTGGCTGATTGATCGGAATCTGAAGGAGATTGGCCGGAGGATTGATGACCTGAGGAAAAATGGTCCGGGTTCGGGTTCGTCTGGAAC

GATGGCGGCGGCGGCGACAATGGTGGAGGCGGTGGAGCAGAAGCCGGAGATGGATCAATTCGCGGTGGACGTGATGACGCAGCGGCCGCCGTGGTTGACG

GAGTGGATGAATAATCCGATAGAGAACTTAGGGTTTGGGCATGGCGATGAGACGATGATGATGATGATGAATAACATGCCGTTTGGGGATAATCTGAATC

CGATGTGGTCCAGTTCTTTCTTTCCTTAA

>EuMADS18

ATGGGGAGAGGGAAGATTGTGATAAGAAGGATCGACAACTCGACGAGTAGGCAGGTGACGTTTTCCAAGAGGAGAAGTGGGTTGCTCAAGAAGGCCAAGG

AGCTATCAATCCTTTGTGATTCCGAAGTCGGATTGATTATCTTCTCCTGCACCGGCAAGCTCTACGAATTCTCAAGCACCAGCATGAAATCTATAATCGA

AAGATACAACAAGATGAAGGAGGAGCAATACCACCTGGTGAATCCAGCTTCAGAGCTCAAGCTTTGGCAGAGGGAAGCGGCAAGCTTGAGGCAACAGCTA

CATGATTTGCAAGAAAAGCACAGGAAGCTGCTGGGAGAAAAACTTTCGGGTTTGGGCATCAATGAACTACAGAGTCTGGAAAATCAGCTTGAAATGAGCC

TGAAAGGTGTCCGGATGAAGAAGCATCAGATACTGGCTGATGAGATTGGAGAACTGAACCGCAAGGGGTCTCTCATTCATCAAGAAAATGTAGATTTATA

CACAAAGGTTAACCTCATGCATCAAGAGAACATACAACTGCAGACAAAGGTTCATGGAGCAAGTGGCATGCAAGAAGGCAGAACTTCACATATTTCATAT

GGACTCAACAATATATACGACTTGTATGCACCCATAAACCTCCAGTTAAGCCAGCCAAAACAGTGGAACAAGGAAATACCAGCAAAAGCAACAAAGATTG

GGCTACAACTGCAATAG

>EuMADS19

ATGGGGAGAGTGAAACTTGCGATAAAGAAGATAGAGAACACAACGAACAGGCAGGTGACATTCTCAAAAAGGAGAAATGGACTTATCAAGAAGGCTTATG

AACTCTCTGTTCTCTGTGATGTTGATGTTGCTCTTATCATGTTCTCTCCTTCAGGAAGACTCAGCATCTTCTCCGGAAACAAAAGCATCGAAGATATCGT

GGCACGGTTTTTGAATCTCCCTGAGCATGAACGAGGAAGGATCAACAGCCAAGAGACTCTCCAAAGGGCTCTGGGTAAGTTGAAAACTCGATCAGATCCA

ACAAATCAAACAACCAGCCCAGGTAGTGTTGATTCTCAACTTGAGGAAATTCAACTAGAAATTATCAGGTGTAAGACAAAATTGGAGGAAATGGAGAAAC

GATTAAGGATATTTGAAGGCGATCCTGTCGAAATCACAACACTGTGTGAGGCTGAATTCCGGGAACGAATCCTCAAGGAGACACTAAAGAATGTTCATCT

ACGCAAGAAAGTATTAGAGGAAGAGTACAATAAATCTCCAGGTGATGGACAGCAAACTCCAGAGACGGTTAATATGATTGATTTGAGTAGTAGAAATCCA

AACAATGTCTTGGATTGGCTTCCACAGGGTGATCCACAAGTCCAAATCCTCAATTTTTTAGACTCCAATGGCCTACTTTCACAAAGAGATGACGAAAATA

TATTTGGACAAAGCTCAAGTGTTGTCAATGGACAAAACCTTCATGTCGATGAAGGCCATTTGAGCCCTAGCAGTGGCTTGGAGGATAATAGTAGTCTCCA

ACAACCCGAATTCGGACATGTTATCGATGTTAATCTTTCTCCGTGGACGCCATTTTATTCAACAGGAAATGGACACTTCTCAGCATCTCAGCCGAGAGAA

CACGCACTACTTGAACTCTTTTTGTCTCATTTTAGGCAATGA

>EuMADS20

ATGGGCCGTGTTAAATTGGAGATAAAGCGAATAGAGAACAACACGAATCGACAAGTTACGTTTTCCAAACGTAGAAATGGGCTCATCAAGAAGGCGTATG

AGCTGTCGATTCTGTGCGATATTGATATTGCTCTTATCATGTTTTCGCCGTCGGGTCGTCTCAGTCACTTTTCTGGCAAGAAAAGGATTGAGGATGTGTT

CGCTCGTTTTATAAGTCTGCCGGATGACGAGAGAGAAAGAGCTGTAGCGGAAGAACAAGGCGGAGAACATTACCGAAATGCTCTTTGCAACAGAGAGTAT

TTCATCAGGACTCTGAAGCAACTTAGGTTTGAAAATGACATCGCTCGTCAACTCGCCAATTCAACGGCCATTAACTCTGAGATTGAGGAACTCCGACAAG

AAATTAGTGGGTTACAGCAACAACTTCTAATGTCAGAGGATCAACTAAGAATTTTTGAGCCTGATCCATGGAAGATGGCATCAACGGAGGAGTTTGAATC

ATGCGAGAAACAACTTGAAGACACTTTAGCCCGTGTCGTGGAAAGAAAGAAATTTTTGATGAGCAACAATCTCTCTTCCTATGCTTCACCACTACAGGAA

TTGCCGACCTCTTTTGAGACTGAAGCTGTCCGTTGGTCACTAGAACACGGTCACAACCATGCCCCTATTTTCGATTCCTCTGCTCCTTTGATTCCAGTTA

GGGATAACATGTCAGCGATTTACACGCAATTGTCTCAAGAAAGCAGTTCGAACATGGAGCAACAACAACACAACCTTGAGGAATGCCATGCCAACAATCC

GAGTGACGGTGATTTTCTTCAATGGCAGCACGCTGGAACCCAAATGGTTCTTCATCCCCCTCACATTCCACCATCTTTGTTCTCAAGCGGACTTCCTATT

CAGACTGCAATGATGCAACTTGATCAGGTGGAGATACCGTTTAACGGATCGAGCGTGCAATCGGAAAATGGTGCTGCAAACTATGGAAACTAA

>EuMADS21

ATGGTGAGGAAGAAGATCCAGATCAAGAAGATTGAAAAGACAGCGGCCAGGCAAGCCACCTTCTCCAAGCGAAGAAGAGGGCTTCTAAACAAAGCTTCAG

AGCTTGCTGTTCTTTGCGATGCCCAGGTTGCCCTCATAATCATCTCTGAAAGTGGAAATTTATTCAAGTATTCCAGCTCAAGCATGTCTGATATACTGAT

GAAGTATAATTTCCAATCTAATTTGGAAAAACTTGTCTGCCCTTCACGAAAACTACAGCAAATAGGATTTGGCGACGAAAGTGCAAAGAAGATGCGTCAG

TTGAGGCAGATGATGGGAGAGGATATACAAAGATTAAATATAGAGGAACTGCAGCAATTACAAAACGTTATTGAAGTTGGATTAACTCGTGTGATCAGGA

CAAAGAACGAGCGGATTCTGGATGGCATAGCAGTTCTTCAGAGGAAGCATGCTGAGATTATGGAAGAGAAGGAGCAACTGAGAAACAAAACGACAATGAT

TTTAAATGAAGAGCGGTTTGATCTTGGCATTATTAGTCATGAAAATCCCGAGAAAGAAAATTGGCTTGATACATCTCTGCAACTCGCTAGGGTTTATGAC

AGCTATAAACCCTAG

>EuMADS22

ATGGTGAGGAAGAAGATCCAGATCAAGAAGATTGAAAAGACAGCGGCCAGGCAAGCCACCTTCTCCAAGCGAAGAAGAGGGCTTCTAAACAAAGCTTCAG

AGCTTGCTGTTCTTTGCGATGCCCAGGTTGCCCTCATAATCATCTCTGAAAGTGGAAACTTATACAAGTATTCCAGCTCAAGCACGTCCGATATACTGGA

GAAGTATAATTTCCAATCCAATTTGGAAAAACTTGTTGGCCCTTCACGAAAACTACAGAAAATAGGTTTGGGCGAGGAAAGTACAAAGAAGATGCGTCAG

TTGAGACAGATGATGGGAGAGGATATACAGAGATTGGATATGGAGGAACTGCAGCAATTACAGGACGTTATTGAAGTTGGATTAACTCGTGTGATCAAGA

CAAAGAACGAACGGATTTTGGATTGCATTGCAACTCTTCAGAGGAAGCATGCTGAGATTATGGAAGAGAATGAGCAACTGAGAAACAAGACAACAATGAT

CTTAAACGAAGAGCGCTTTGATCTTGACAGTATTAGTTGTGAAAATCGGGAGAAAGAAAATTGGCTCGATACATCCCTGCGACTAGCTAGGGTTTATGAT

CGCCATAAAACCTAG

>EuMADS23

ATGGTGAGGAAGAAGATCCAGATCAAGAAGATTGAAAAGACATCGGCCAGGCAAGCCACCTTCTCCAAGCGAAGAAGAGGGCTTCTGAGCAAAGCTTCAG

AGCTTGCTGTTGTTTGCGATGCCCAGGTTGCCCTCGTAATCATCTCCGAAAGTGGAAAATTATTCAATCATTCCAGCTCAAGTGGCTGCTCAATCACCTC

CTTACTTATGCCGCTGCGCAATCGACGAAAGACACCGCTTCACTCAATCCAAGAGTTATTTCCTTCACCGGCCGATGACACTAATGGTGACACCAAATTG

GACGATGATGCTGAGCATAATGGCTTCAAGTGGTTTGTCAAAGTTCACCCTGCGCGATTCAATGAGGGGATGACAGTCAACCGAAAGAGCAGGAACAAGT

TGATAAGGGGGAGTCATGTTGAGCCCAAAAAATCGAGAAAGTGTGGCAAACAGAAATTAGACACTGAATTTGGGGTAGTAAGAGGGATAGACTTAAAAAA

TGTACATGCGGTGAGTACATATTTTCTTTAG

>EuMADS24

ATGTTATTTTTTTATTTATTTATCAATATTTGTGGGATTGAAGGAGGGAAAATGGCGAGAGAGAAGATCAAAATAAAGAAAATAGACAACATAACGGCGA

GGCAAGTGACTTTCTCCAAGAGACGAAGAGGACTCTTCAAGAAGGCCGCCGAGCTCTCCGTTCTCTGCGATGCCCAAGTTGCGCTCATCGTCTTTTCCAC

CACCGGCAAACGATTTGAGTTCGCTAGCTCCAGCATGAAGGATATTCTTAATAAGTATAAAAAGCACTCGGATAATATCGATAAGATTGATCCACCTTCT

CTCAATCCCCAGGTAGAGGACAACGATGATCATGCCAGATTGAGCAAAGAGGTTGCGGACAAGGCCAATCAGTTGAGACAGATGAGGGGTGAGGATCTTC

AAGTCTTAAGTATTGATGAACTGCAGCATTTAGAGAAAATGCTGGAAGGAGGACTTAGCCGTGTGCTTGAAACAAAGGGTGAACGAATCATGAAAGAGAT

TTCCAACCTTCAAAGGAAGGGTGCTCAATTGATGGAAGAGAACAAGCAATTGAAAGAGAAAATGGATATGCTTTGTAAAGGAAAAAAACCTCTGCTGATC

GAACTTGAAAATACAGTGATCCCAGAAGAGCAAGGTCAATCTTCGGACTCCGCCACCAATGCCTACAGTTGCAATAGTGGACCTCCGACGGATGATGATT

TCTCCGATACTTCTCTCAAACTCGGGTTACCCTTCAATTAA

>EuMADS25

ATGGCGAGAGAGAAGATCAAAATAAAGAAAATAGACAACGTAACGGCGAGGCAAGTGACTTTCTCCAAGAGAAGAAGAGGACTCTTCAAGAAGGCCGCCG

AGCTTTCCATTCTCTGCGATGCCCAAGTTGCGCTCATCGTCTTTTCCACCACCGGCAAACGATTTGAGTTCGCCAGCTCCTGCATGAAGGATATTCTTAA

TAAGTATAAAATGCACTCGGATAATATCGATAAGGTTGATCCACCTTCTCTCAATCCCCAGGTAGAGGACGACGATGATCATGCAAGATTGACCAAAGAG

GTTGCGGACAAGACCCATCAGTTGAGACAGATGAGGGGTGAGGATCTTCAAGGTTTAAGTGTCGATGAACTGCAGCATTTAGAGAAAATGCTTGAAGGAG

GACTTAGCCGTGTGCTTGAAACAAAGGGTGAACGAATCATGAATGAGATTTCCAACCTTCGCTGGAAGATGGATATGCTTTGGAAAGGAAAGAATCCTCT

GCAGATCAAACTTGAAAATACAATGATCTCAGAAGATCAAGGTCAATCAACAAACTCAACTACCAACAGCGGAAAGAAACCTCTGCTGATTGAACTTGAA

AATACAACAATCCCAGAAGATCAAGGAAAGAAACCTCTGCAGATCGAACTTGAAAATACAACGATCTCAGAAGATCAAGGTCAATCAACGGACTCAACCA

CCAATGCCTACAATTGCAACAGCGGAAAGAAACCTCTGCTGATCGAACTTGAAAATACAACAATCCCGGAAAATCAAGGTCAATCATCGGACTCAACCAC

CGATGCCTACAGTTGCAACAACGGACCTTCTACGAAGGATGATTTCTTTGATACTTCTCTCAAACTCGGGTAA

>EuMADS26

ATGGCGAGGGAGAAGATCAAAATAAAGAAAATAGACAACATAACGGCGAGGCAAGTGACTTTCTCCAAGAGAAGAAGAGGACTCTTCAAGAAGGCCGCCG

AGCTTTCCGTTCTCTGTGATGCCCAAGTGGCGCTCATCGTCTTTTCCACCACCGGCAAACGATTTGAGTTCGCCAGCTCCAGCATGAAGGATATCCTTAA

GAAGTATAAAATGCACTCGGATAATATCGATAAGGTTGATCCACCTTCTCTCAATCCCCAGGTAGAGGACAACGATGATCGTGCAAGATTGAGCAAAGAG

GTTGCGGACAAGACCCATCAGTTGAGACAGATGAGGGGTGAGGATCTTCAAGGCTTAGGTGTTGATGAACTGCAGGATTTAGAGAAAATGCTTGAAGGAG

GACTTAGCCGTGTGCTTGAAACAAAGGGTGAACGAATCATGAATGAGATTTCCAACCTTCAAAGGAAGGGTGCTCAATTGGTGGAAGAGAACGAGCAATT

GAAAGAGAAAATGGATATGCTTTGCAAAGGAAATAAACCTCTGCTAGTTGAACTTGAAAATACAACGATCCCGGAAGATCAAGGTCAATTGTCGGACTCA

ACCACCAATGCCTACAGTTGCAACAGCGGACCTTCGACGGAGGATGATTTCTCTGATACTTCTCTCAAACTCGGGGGTCGAAGATACATTCCAAATGGCA

TAGAGTGTAGATTTTCGAAATAA

>EuMADS27

ATGGCATTGGTCGGCGATAGGTCGATTGATGACAATGACAGTGAACTGAACAATCGGTGTTGGTGTGCTATTGCGGTATGGTCTGCGACGGTGATGGAGC

TTCTCATTCATTATATTCGTCACGTTAACCTTGCCCGTTTTGGATCGTTTTTCATGCCCAATAGGGCTAAAAATGTCAAACCAAAACTCCCAAAATGGGA

AGTAAAATACCGCCAATACCACCATCCACGCGTCCATGATCTATTGGCTACACATCGCTTTCAGGAATCAACAACCAATTGCGATGGCTGTCACGCACTT

TCCTTATATGGCCGATCGTGTGAAATCAGAAACTCTTACCAAAACGACGATCGCAGAGTCTCCTTTTGTCTCCTTCACCAAAACCTCCACTCTCAAATAT

TCTTTATCTGGAAGTCAAGGAAGTTAGGGATGGGGAGAGGAAAGATTGTGATAAGGAGGATCGACAATTCAACGAGCAGGCAGGTGACGTTCTCGAAGAG

GAGGAACGGATTGCTGAAGAAGGCAAAGGAGTTGGCGATTCTGTGCGATGCGGAAGTCGGAGTCATGATCTTCTCCAGCACTGGCAAGCTCTACGATTAC

GCTAACACCAGCAGCATGAAGTCCGTGATTGAAAGGTACAACAAGTCCAAAGAGGAACCTCGCCAATTGGCAAATCCAATTTCAGAGGTCACGTTTTGGC

AGCAGGAAGCAGCAGTTTTGAGGCAACAACTGCAAAATTTGCAAGACAATCATCGGCAAATGATGGGGGAAGAACTTTCTCGTTTAAGCGTGACAGAACT

ACAAAACTTGGAGAACCAGCTGGAAATGAGTCTTCGAGGTGTCCGCATTAAAAAGGATCAGGTTTTAATAGATGAAATAGAAGAACTGAACAAGAAGGGA

AGCCTCATTCACCAAGAAAATGTGGAACTGTATAAGAAGGTTTATGGCACTAGGCGTGCAAATGGAGAAAACAGAAATGCTCTTCTCACAAATGGTTTGA

GCATTGGAGAAGAGCTGCATGCATCTGTTCATCTACAGCTTTGCCAGCCACAGCAACAAAACTACGAAACACCAGCACAAGCTACAAACTTGGGATAA

>EuMADS28

ATGGGGAGAGGGAAGATTGAGATAAAAAGGATAGAGAATTCAAGTAACAGGCAGGTGACCTATTCAAAAAGAAGGAATGGGATTATTAAGAAGGCTAAAG

AGATCAGTGTTCTATGTGATGCTCAGGTATCTCTCGTTATCTTTGGTAGCTCCGGCAAGATGCATGAGTACTGTAGTCCTTCTACCAATTTAATAGATAT

GTTGGATAAGTACCAGAGGCAGTCTGGTAAGAGATTGTGGGATGCTAAACATGAGAATCTCAGCAACGAAATTGAAAGAATCAAGAGAGAGAATGACAAT

ATGCAGATCGAGCTCAGGCACCTTAAAGCTGAGGATATCACATCTATGCAATACAAAGAACTCATGGCTATGGAAGAGGTCCTCGAAAATGGTCTCACGA

GTGTCCGTGAAAAAAAGATGGAGTTATTCAATGCGACTAAGGAAAATGATGAAATTCTGGAGGAGGAAAATCGTCGACTTAATTACATGTTGCATCAACA

AGAGATGGAAGCGATGAACAACAATATGAGAGGGATGCCTTTCCCATTTCGGGTGCAACCATCACAGCCAAATCTACATGAGATAATTTAG

>EuMADS29

ATGGTGAGAGGAAAGATTCAGATGAAGAGGATAGAGAATGCAGCAAACAGGCAAGTGACATTTTCAAAGAGAAGAAATGGGCTCCTCAAGAAGGCCTTCG

AGCTGTCTGTTCTTTGTGATGCTGAAGTTGCCCTTATTGTCTTCTCTTCTAATGGCAGACTCTACGAGTTCTCAAGTTCAAGCATTAACAAGACAATCGA

CCGTTATCAGAAGAACACAAAGAACCTCGGATTCTGTCCTAAACCAGTTCAAGAAAATTATCAGCTATTGAAGGATGAGGCTGCTAAAATGACCAAAAAG

ATCGAGGTTATTGAAGATTATAAAAGAAAACTGTTGGGGAGTGGTTTGGATTGTTGCTCTACCGATGAGCTTCAAAATGTTGAGGTTCAATTAGAGAAAA

GTTTGAGAAATATTAGGGCCAGAAAGAATCAGCTATTAAAGGAGAAGATTCAGAAGATGAAGGAGGAGGAGTTAATTCTAGCAAAGGAAAATTCACAACT

AAAGAAGGTCAGTTTTGACATTTCAATAACAACGCGTGTGTGTATATGTATATACACTTGTTGGTTTTATAGTAAGGTGTGTTTGTGTTAG

>EuMADS30

ATGGGGCGAAGGAAGGTGGAGATAAAGCGAATCGAGAACAAGAGCAAGAGACAGGTTGCCTTCTCAAAGAGACGAAATGGATTGATGAAGAAAGCTCGAG

AGTTGTCCGTTCTTTGCGACGTCGACGTCGCCGTCGTCATTTTCTCCACACGTGGGAAGCTCTACGACTTCTCCGGCGGCCACAGTTTGGCCGAGATTCT

TCAACGACATCAGAGCTGCGCAGAAGCAGAAGGAGAGATTTCTACTGGAGCCCATGAGGCACAGGTTCAACCTGAATCGTCAAGTTTCATATCCAGTAGG

GAGCTCTTGCAGATAGTTGAGAGTTTTGAAGAACCAAATGCTAAGCAGCTTACTGTAACTGATCTTGAGGAGGTAGAGAATCAACTTGATGCTGCTTTAG

TACAAACAAGATCCAGGAAGACACAGCTTATGATGGAGTCATTAAAAGATCTTCGGGATGAGGAGAAAAAGCTGAATGAAGAGAAAAAGTCTCTGCAGAA

CGAGATAGCCAACTACAGGACTCAACCACTGGTAACTCTCTCTCTTCTTTAA

>EuMADS31

ATGGGGCGAAGGAAGGTGGAGATAAAGCGAATCGAGAACAAGAGCAAGAGACAGGTTGCCTTCTCAAAGAGACGAAATGGATTGATGAAGAAAGCTCGAG

AGTTGTCCGTTCTTTGCGACGTCGACGTCGCCGTCGTCATTTTCTCCACACGTGGGAAGCTCTACGACTTCTCCGGCGGCCACAGACTAAGCTTCGATTT

CTCATATAAGGTAGATATCCACCGCCCTTCGACTATTGGTATATTTGTTAACAACGTTCATAATCAGTCCGTGAATCAAAGATTTCTTGTACCGAGGATT

ACATTTTGCAATGATTCTGTGAGCAAATGA

>EuMADS32

ATGGGGCGAAGGAAGGTGGAGATAAAGCGAATCGAGAACAAGAGCAAGAGACAGGTTGCCTTCTCAAAGAGACGAAATGGATTGATGAAGAAAGCTCGAG

AGTTGTCCGTTCTTTGCGACGTCGACGTCGCCGTCGTCATTTTCTCCACCCGTGGGAAGCTCTACGACTTCTCCGGCGGCCACAGTTTGGCCGAGATTCT

TCGACGACATCAGAGCTGCGCAGAAGCAGAAGGAGAGATTTCTACTGGAGCCCATGAGGCACAGCAAATTCACCCTGAATCTTCAAGTTTCATATCCAGT

AAGGAGCTCTTGCAGATAGTAGAGAGTTTTGAAGAACCAAATGCCAAGCAGCTTACTGTAACTGATCTTGAGGAGGTAGAGAATCAACTTGATGCTGCTT

TAGTACAAACAAGATCCAGGAAGACACAGCTTATGATGGAGTCATTAAAAGACCTTCAGGATGAGGAGAGGAAACTGAGGGAAGAGAAAAAGTTAATGCA

GAACGAGATAGCCAACTGCAGGACTCAACCACCGTGA

>EuMADS33

ATGGGGAGGGGTAGAGTTGAGCTGAAGAGGATAGAGAACAAGATCAACAGGCAAGTGACCTTCGCTAAGCGAAGGAATGGATTACTGAAGAAAGCATACG

AGCTTTCCGTGCTTTGCGATGCAGAGGTGGCTTTGATCATCTTCTCCAATAGAGGAAAACTGTACGAGTTTTGCAGTAGCTCAAGTATGCTGAAAACTCT

CGAGAGGTATCAAGAGTCCAACTATGGAGCGCCGGAGACAAATGTATCCACCAGAGATGCACTGGAAATAAGCAGTCAGCAGGAGTATCTCAAACTTAAA

GGACGATATGAAGCCTTACAACGATCCCAAAGAAATCTTCTCGGTGAGGATCTTGGACCCCTTAACAGCAGGGAGCTTGAATCGCTCGAGCGCCGACTTG

ATACCTCCCTGAAGCTGATCAGATCAACAAGGACTCAGTGCATGCTGGATTCTCTCACTGATCTTCAGACTAAGGAGCACGCACTCAATGAAGATAATAG

GGCATTGAGACAAAGGCTGATGGAAGGGAACCATATAAATTCATTCGAATGGAATCAAAATGCACATGATCATGTGTTTGGTCGACAACAAGCTCAACCT

CACGACAATGTCTTCTTCCATCCCTTGGAGTGTGAACCAACCTTACAAATCGGATATCCTACAGATCCAATAACAGTAACCACAGCAGCAGGTCCCAGTT

TGAGTAATTACATGGGTGGATGGTTGCCATGA

>EuMADS34

ATGGGTAGAGGGAGGGTTGAGCTGAGAAGAATTGAGAACAAGATAAATCGGCAGGTAACGTTTGCAAAGAGAAGGAATGGTCTGCTGAAGAAGGCTTATG

AGCTCTCTCTTCTCTGTGATGCTGAGGTTGCTCTCATCATCTTCTCCAACCGTGGCAAGCTCCATGAGTTCTGCAGCACCTCTAGCATGGCCGAGATACT

GGAGAAGTATCGGAGATGTAGTTATGGTGCATTTGAAGCAAACCAACCGCCTGATCATACACAGGGCAGCTACCAGGAGTATTTGAAGCTTAAGACCAGA

GTTGACGTTTTAGAACAATACCAGAGACATCTTCTTGGGGAAGGTTTGGGGGAGTTGGGCATAAAGGAACTGGAGCAGCTTGAGCGACAACTAGACGTGT

CCCTGAAGAAAATCAGATCCAAGGAGACTCAATTTATGCTTGACCAGCTCTCTGCTCTTCAACAAAAGCAACAGGAATTACTAGAAGCCAACAAAGCCTT

AAGGTCCAAGATAGAAGAGAGCAATGCAACCCTTCAATTAGCACCATGGGAAGCAAGGGACTGCCCTATGCCATTCCGGCACCAGCCTCCACAGACCGAG

GAATTCTTTCAGCCCCTGGAATGCAATAATACGTCGCAAATCAGGTACACTTCAGTATTACCTCAGGACGAGCTGAACAATGCTACATCAACTCGGCATG

CCTATAATGAAATGATCCCCGGGTGGATGCTTTGA

>EuMADS35

ATGAGAAAGGGGAAAACAATACTTGAAAGGATTCCCAATGAAACTGCAAGAAGAACGAGTCTCAGAAAGCGGAGGGATGGACTGATTAAGAAGGCAAGAG

AGCTTTCTATATTGTGCAACGTGCAACTTGGTGTAGTTGTCTATAGCCCAGATGAGGATACTCCGGTGACTTGGCCATCTCAAGAAGTAACGGCCCAGAC

ATTGGCAAGGTTCCAAGAGGTGCCTGAGGTCGAGAAAAGCAAGAAAATGGTGACACATGAAACCTACCTAGCCGATAGGGTGACGAAAGAGAGCGAAAAA

ATAAGGAAGTTTGAGAGAGATAATGATGAAAAAGAACATCAACTAATCATGACCCAATTGGTTGAAGGTAAGAGCTTAAATGAACTTGAAGTGATGCAAC

TTCAAGGAATGCTCTATTTCATGGCAAAGGAGTTGAAGAAGCTTGAAAAAAGGGACCAAGATTTGGATCGACAAGCCCCTCCTTCTCATTTACCACCACC

ACCTCCTCATCTTGGTCATCTTCCCCCTCCTTCTCAACTTGGTTATCATCCTCATCCACCTCCAGGTTATCATGCATATTATCCTCCTCCTGCTCCTTCT

CCTCAGCCAACGGAATTGGATGATGCTGCACAGGTTCAAGGGATAACTGATCCCAGCACCTTGGTTCCATTCTCTATGGAGTATCTGATGGACGATAAAT

GGTTTATGGGTACTATTTTTGAATATAAGGACGTTCCAGGCCCTCGTGGAGAAGGCAACGTACGGTTACAACCTGAATATGCTTCCAGCAGTGGTCCAAG

CAATGTTGTTGATGAACATCAAAACGACCCCAATCAAACATGGCCTAAACACTTTTTCCCTTGA

>EuMADS36

ATGGGGAGAGGGAAGGTGGAGCTGAGGAGGATAGAGAACTCGACGAGCAGGCAGGTTACCTTCTCAAAGCGGCGAAACGGCCTCCTTAAGAAGGCCTGCG

AGCTCTCCGTCCTCTGCGATGCCGAGGTCGCCCTCATCATTTTCTCCCCCTCCGGCAAGGCCTATCAGTACGCCAGCCAAGACATAGGCAGGACCATTTC

CAGGTACCGCAATGAAACGGGTTTTTTCAATCCCAACAATCAACTCTTTAGATCCACTGAGATTTTGAGGAGTGAACTGGATGAGTTAAGGAGAAGAGTG

GAAACAATGGAAACAAGAAACAAGCATGTTGCTGGGCAAGATTTAAATATGCTTGGAATGAAAGACTTGAAGCGATTAGAGCGACAATTGAGAACTGGAG

TTGAACGCATACGCTCTAAAAAGAGGCGCATTGGGTCAGGGTACATCAATTCTCTGAAGAAAAAGCGTAGAGACCTACAAGAAGAGAACACTCATCTACA

AAAGAGGCTGGAAGAGCTGCCGGAGCTCGGTGGAAGCTCAAGAATGTGTGGAGCAACACCATTTGATGTATTTCAAAGGTATGCTGAAGAAGGGACTTCA

GCAATATAG

>EuMADS37

ATGGGGAGAGGGAAAGTAGAGCTGAAGAGGATAGAGAACAAAATAAACAGGCAAGTAACCTTTGCCAAGAGAAGAAATGGGGTTCTCAAAAAGGCTTATG

AGCTCTCCATTCTCTGTGATGCTGAGGTCGCTCTCATCATCTTCTCTAGCCGTGGGAAGCTCTATGAATTCTGCAGCGGCTCTAGCATGACAAAAACACT

GGAAAAATATCAAAAATGCAGTTTTGGTTCTCTAGAACCTGATCCAATGATCAATGGGAATCAGCATAGCTACCACGAATATCTCAGACTGAAGGCAAAA

GTTGAAGTCATGCAACGATCTCAGAGAAACCTTCTTGGGGAAGATTTGGGGTCGTTAAACTCAAAGGAGATGGAGCAGCTTGAGCACCAACTAGAAATTT

CACTCAAACAAATCAGATCAACAAAGAAGTTGGCTTTTTCTGCTTCATTCTTCTTGTTCGGTGGATTTTTCCCAAATCGGATGTGGAAAGGAGTGACGGT

CAGATCCAAGACTACCCTACACAGGTATCAGAGCTCAGGTTACAACAATTCTGACCCAATAACCACAAAAAGAGAGATTGGTAGAGGTTTAGCATATCAA

AGGCGCCCGATGTGTGAGGAGAAAATTGTTGGATTTATCCCACATTGA

>EuMADS38

ATGGGGAGAGGAAGGGTTCAGCTGAGGAGAATCGAGAACAAGATAAATCGGCAAGTGACTTTCTCGAAACGGCGATCTGGGCTGCTGAAGAAGGCTCATG

AGATCTCTGTTCTCTGCGACGCGGAGGTCGCTTTGATTATCTTCTCCACCAAAGGGAAGCTTTTTGAGTACGCCACCGATGCTAGCATGGAAAGAATCCT

TGAACGGTATGACAGATACTCATATGCCGAAAAGCGACTTGTTGCACCTGACGTTGAATCACAGGGAAGCTGGACGTTCGAACATGCTAGGCTCAAAGCT

AGAGTCGAAGTTTTACAGAAAAATCAAAGGCATTATATGGGAGAAGATCTTGATTCTTTGAGTATGAAAGACCTTCAGAATTTGGAGCACCAGCTAGATG

TGGCTCTCAAGCATATCAGGTCAAGGAAGAACCAACTCATGCATGAATCCATTTCTGAGCTCCAGAGGAAGGATAAGGCATTGCAGGAGCAAAATAACTT

GCTTGCAAAGAAGGTGAAGGAGAAGGAGAAGATGGATTTGGCTCAACAAGATCAAGTTTTGGGGCCGAGTAGTAACGACTTGAACTCCTCTTTTGTCATA

CAACAGCCCTTACCCTCCTTGAACATTGGAAGTGATGCATATGAAGAAGCCGAAGGAAGAAGAGAAGTTGAAGGAATAGCAGCAAGCCAACATCAGACGT

ATGTATCCATGCCCCCTTGGATGCTTCACCATGTTAATCGGTAG

>EuMADS39

ATGGCTAGGGGGAAGATCCAGATCAGGCGCATCGAGAACTCGACGAACCGGCAGGTGACTTACTCGAAGAGGCGGAATGGTTTGTTCAAGAAAGCGAATG

AGCTCTCGGTTCTGTGCGACGCGAAGGTCTCCATCGTCATGTTCTCCAGCACGCACAAGCTTCACGAGTACATCAGCCCTACCACCACAATGAAGGAGTT

CTTCGATCAGTACCAGAGGACCGTCGGAGTTGACCTTTGGAGCTCCCACTATGAGAAAATGCAAGAACAGTTGAGGAAGCTGAAAGATGTGAATAGGAAT

CTCCGGAGGGAGATAAGGCAGAGGATGGGAGAGAGTTTGAACGATCTGAAGTATGATGAACTGTGCGATCTTGAGCAAGATATGGACAATTCTGTGAAGA

TCATTCGCGAGCGGAAGTACAAGGTGATCACTAATCAGATTGACACCTACAAGAAAAAGCTAAGGAATGTGGAAGAAATCCACAGAAACCTTCTCCATGA

ATTTGATTTGAGGGACGAGGATCCACACTATGGTTTAGTCGACAATGGTGGGGATTATGATACTTCTGCTGTTCTTGGTTTTGCAAGCGCAGGCCCACGC

ATACTCTCCCTGCGCCTGCAGCCCACCCAAACAAACATCCACGCTGGAGGAGGATCGGATCTCACAACCTTCGCTTTGCTCGAGTAG

>EuMADS40

ATGGTGAGAGGGAAGATTGAGATGAAGAGGATAGAAAAAGCAACAAGGAGGCAAGTGACATTTTCGAAGAGGAGAAATGGGTTGTTGAAGAAGGCTTATG

AGCTATCGGTTCTCTGCGATTCTGATGTTGCTTTGATCATCTTCTCTCAGACTGGAAGGCTCTATGAATTTTCAAGCTCAAACTTGCAAACAATAATCGA

GCGATACCGCGAATGTGAGAAAGATCAAAATCGAGACAACGGTCCTGAAATCGAACGATATATTCAGCATCTGAAACAAGAATCTGCAGGCATGGCGAAG

AAGATAGAGCACCTTGAACTCTCCCAACGGAAACTTTTGGGGCAAAATCTTGAATCATGTTCAGTAGATGAACTCCAAGATATTGGAAGCCAACTTGAGC

AAAGCTTGAAAAATATCAGGAGGAGAAAGGATGATTTATTGAAGGAGGAAATTAAACAATTAAAAGCAAAGGAGAGATTTCTGCTAGAGGAAAAAGGCAG

GTTATCTGAAAAGTGTGTAGCAAAGACGAGACAACCGTCGTTGTGGATGAATGAAGGTGAGAGATGCGGCCAAAGTAGCCCGAGTTCAGAAGTGGAGACT

GAATTATTTATTGGTCTGCCTGAGATGCGTTGCACGTTGTAA

>EuMADS41

ATGGCTCGTGGGAAGGTACAGATGAAGAGGATCGAGAATCCGGTGCACCGGCAGGTCACTTTCTGCAAGCGTCGCGCGGGGCTTCTTAAGAAGGCCAAGG

AGCTCTCCGTTCTTTGCGACGCTGAGATTGGCTTGTTCGTTTTCTCCGCCCATGGCAAGCTTTACGAACTCGCCACTAAAGGAACCATGCAAGGCCTTAT

CGAGAGGTACATGAAGTCTACCGGGGGGATGGAACCTGAACAACCCAATCAAACCCAACTTCATGACTCAAAAGTGCAGATTAACCTACTGAAAGATGAG

ATTGATGTACTTCAGAAGGGGCTTAGGTACTTGTATGGAGATGATACTGAAAATATGACATTGGACGGGTTACACATACTCGAGAAGCATATGGAAATCT

GGATTTCCCATATACGATCTATAAAGATGGAAATTATGTTTCAAGAGATACAAGTATTGAAGAACAAGGAAGGGATGCTGAATACTGTCAATAAGTATTT

ACAAGAGATGATCGATGACCATTATGAGACCACTAACATCGTTCCAGTGATGACTAACACAACATACCCACTAACAATACAGAATGAGATATTTCCATTC

TGA

>EuMADS42

ATGAACGACAACAACATGCAGAAGAAGAAGAAGACAACCCAGGGAAGAAAGAAGATCGAAATAAAGGAAATAGAGGGAAAGAGCAACAAGCAGGTTACCT

TCTCCAAGCGTCGTTCTGGCCTCTTCAAGAAAGCCGGCGAGCTCTGCGTCTTGACTGGTGCCGAAGTTTCCGTCATAGTCACATCCAATGCCGGTCGCGT

CTTCGCCTTCGGACACCCTAGCGTCGACGCCGTCGTTGACCGCTGCCTCGCGGGTGGGAGTTACAAATCCGACGCGATCGAGAAACGGTACCCTTACGCC

CACGAGTTTAACCAGCACTACGACAGGGTATCGGAGGAATTGGAAGCGGAGAAGAAGCGGAGAGAGGCAATCGACGAGAGAGAGCCGGATGGGGCGGCGG

AGGATAGGGCGTTTTGGTGGAGCGAACCGGTGGACGATATGGGGTTGGAGGAGTTGGAGAAGTACTTGGCTTCGATGGAGGAGTTGAAACAGAAGGTGGA

GATGAGGGCTGAGGAATTGAGGATGATGAAAATGGCGGCGGCGGCGGCGGTGGGTGGTGGTGCTTCTTCTTCGTATTCAAACGCGGGAATGAATGCGTTG

GTGGATCATAATCATATGGTGAGTCAAACGGCTGCTAGTTATGGCAATCCTATGGTTCCTCTGGAGTATGATTTTGGACATGGCCAAATCTGA

>EuMADS43

ATGAACGACAACAACATGCAGAAGAAGAAGAAGACAACCCAGGGAAGAAAGAAGATCGAAATAAAGGAAATAGAGGGAAAGAGCAACAAGCAGGTTACCT

TCTCCAAGCGTCGTTCTGGCCTCTTCAAGAAAGCCGGCGAGCTCTGCGTCTTGACTGGTGCCGAAGTTTCCGTCATAGTCACATCCAATGCCGGTCGCGT

CTTCGCCTTCGGACACCCTAGCGTCGACGCCGTCGTTGACCGCTGCCTCGCGGGTGGGAGTTACAAATCCGACGCGATCGAGAAACGGTACCCTTACGCC

CACGAGTTTAACCAGCACTACGACAGGGTATCGGAGGAATTGGAAGCGGAGAAGAAGCGGAGAGAGGCAATCGACGAGAGAGAGCCGGATGGGGCGGCGG

AGGATAGGGCGTTTTGGTGGAGCGAACCGGTGGACGATATGGGGTTGGAGGAGTTGGAGAAGTACTTGGCTTCGATGGAGGAGTTGAAACAGAAGGTGGA

GATGAGGGCTGAAGAATTGAGGATGATGAAAATGGCGGCGGCGGCGGCGGGTGGTGGTGCTTCTTCTTCGTATTCAAACGCGGGAATGAATGCGTTGGTG

GATCATAATCATATGGTGAGTCAAACGGCTGCTAGTTATGGCAATCCTATGGTTCCTCTGGAGTATGATTTTGGACATGGCCAAATCTGA

>EuMADS44

ATGGGAAGGAGAAAGGCAGAGGTGAAGATGATTGAGAACAAAAGTAGCAGACAGGTGACATTTACGAAGAGGAGACAAGGGCTTTTGAAGAAGACCCATG

AGCTCTCTGTCCTTTGTGATGCTCAGATAGGACTCATCATCTTCTCAAGCAAAGGAAGGCTCTTTCAGTATTCCTCTCACCCACACAGCATGGGAGATAT

CATAGAGAGGTACCACCAGACAACAGGCAGTTCTATTCCTCACCATGACAACCAGGAAGAAATGTGGAAGGAAATGAGGCAATTAAAGCAGAAAACAATG

GATCTTCAGTTGAGTCTTCAACGCTACAAAGGTGATGACTTGAGCTTTGTAAGGGTTGAGGAATTGGATCAGCTTGAACGCCAACTTGAGCACTCAGTTA

AAAAGGTCCGGGCTAGAAAGTTTGAACTCTTGCAGCAGCAGTTGGACAATCTTCAGAGAAGTGAAAAGCTGCTGGAGAAGGAAAATCAAGAGATGTACCA

GTGGTTGATGAACAATCAGATTCAGAAGCACCAAGCAGAGGTAGGGCAGGAAGATCAACAAGCAGTGACTGAGCTGAAGTTCGTGGAAGTACAAGATGAG

CAGCACCAGCAACAACAAGTGTTGGAGCAATTCCCATTCTATGGGGAGGAGCAGCCTAGTGATGTACTGCTACAACTTGCAACCACTCTCCATGACAATC

CTTACCGCCTCCAGCCGATACAGCCCAACCTCCAAGATGCCACCGCCAATACAATACTCCATAAATATGGCGGCTATGGCTCTGATCACTATCGTCACCA

TCATGATCATTACGAGTGA

>EuMADS45

ATGGGGCGGGTCAAGTTGAAATTAAAGAGGTTGGAGAACAATACTTGTCGGCAAACGACTTTTTCAAAGAGAAAGAATGGGCTAATCAAGAAGGCTAGCG

AGTTATCCATACTATGCGACATCGATATTGTCCTTCTCATGTTCTCACCGAGTGGCAAGCCTACTCTATTTAAAGGACAAAATAGCATTGAAGATATCAT

TTTAAAGTTTGCGCAGTTAACTCCGCAGGAAAGAGCTAAGAGGAAATTGGAGAGCCTTGAAGCACTGAAGAGAACCTTTAAGAAGTTGGATCATGATGTT

AATCTTGAAGAATTTTTCGGTCCAAGCTATCAATCGGTTGAAGATTTGACTGATCGAGTGAATTTCTATAGAACTCAACTTTCAGAAGTCCAGAACAGAT

TGAGCTATTGGACCAACCTGGACAAGATTGAAGGCATGCAACACCTTGGACAAATGGAAGATTCTCTCAAGCAATCCATTTCTCAAATTCGGGCACATAA

GGAAAATCTTGTCAAGCAGCAAGAACTAGCTATTCAATGTGCGAACCAGTTTGAAAATGGGATGCATTTACCTTACGATTTGGGTATGGAGCAACAATTC

CAACCTTCATCGTGGATTCAGAACGATCACAGTCAAAATATCACACTGTCAACCGACTTAAATTTGCTTTCCCAAAGGGATTCAGAGTGTTCCGGAGTGA

CATCCTTTGGGAATTGTTCTAATTTCTTTGGTGTTAGCGAAAAACTAGAAATGTCTAAAATCGGGCATGAAAATAGCAGTTTGGGCGAACTATGCACAAA

CGAATCTCTAAGTCTACAAATGTCTGAACAACAACAACAACCTTCTTGTTATCCATCTTACGGTTTCGGTTCGTTGTCTGGTAAAACTTTTCAACAAAAC

ATCCATGAAAATGCTTTGAACTTTTGTCTGGACGCGAATTTTGAGTTTCCTCAATCTGGATATGAGTTTAGCCATCAGGGTTGGGCTGCTTCTGGTCCCG

AAGGTCATGCAATTCAATCATTCGACGAGAATTTCTTTCCCCAGGCAAGATTTCTTGCCCTTTTCGTCTCGTTTCTTTGGTTTTGA

>EuMADS46

ATGGAGAACAAACGGAAAAAGCAAACATTGGGCCGCCAAAAGATTGAAATCAAGAAGATAGAGACGAACAGCAACTTACAAGTCACCTTCTCTAAACGCC

GCGCCGGACTTTTCAAGAAAGCCGCCGAGCTCAGCCTCCTCTGCGGCTCTGACGTGGCGGCGTTCGTGAACTCTCCCACCGGAAAAGTCTATGCCTTCAG

TAACCTCCCGGTGGATGTCCTCCTCGACCGCTACTTGAACCAAACAGCACCTTCTTCTTCTTCGTCATTGTCTTCTCTTTTGCCTTTTTCAGGCCCAACC

GGGCCTCTCCACGAGGTGGCGAAGAAGAAGTACATGGAGGTTAAGACCCAGTTAGAGGTGGAGGAGGAGAGAGAGAGGAGGAAGGCGGCGGTGACAATGG

TGGTGGCTGAGGGCGGTGGTGTAGGAGGAGGAGGAGACGGGTTTTGGTGGGACCAACCCACGGAGGGGCTAGGGTTACAGGAGCTTGAGCAGTACGTGGC

AGCGATGGAGGAGATGAAGAAGAAGGTGGTGGAGCGGGCGGAGGAGCTGGCGGTGGCCAGCGCTTCTCCATCAAACTTACTCATCTCCGCCTTCATCACG

GAGGCTTCAACTGAGGAAAACTCTTTAGATTTTGAGCTCATGTTGTTTTATCAGAGGTTGGACCATAAAGTTGCGGGTGCATCTTACCGCAAGTGTTCAT

TCGGCACAATTCAACTTGAGCAACTCTTGTGGGATCCTCCCATTGGTTGGTGGAGGGGCTCTATCCTGGAGCCCTTTATCCTTGATCACTCACAGCTTGG

ATCCAAGAATCTTCTCTTTCATCATCCGAAGTTTGTGCTGAAAATAGGGGACTTCAACCCTACATATCCTGTAAAATTTCAACAGATTTTGTTCTCGAAG

TTCTTTGTCCCCAATGCTTGGGGAAGGGAATCTAGTTTTGTGCTCGACAACAATCTCCTATTAGGCCTGGGAAGTGATCTGAGCTGGTGGTGA

>EuMADS47

ATGGAGAACAAGCGAAGCAGGGGTCGACAGGTGATTCCCATGCAGAAGATTGAGAACGAAGAAGATCGCTACGCCACCTTCTCAAAGCGACGCAAAGGTT

TATACAAGAAGGCGAGCGAGCTCTGCACCCTCTGCGAAGCTGATGTTGGCATAGTCATCTTCTCCCCCACCGGTAAGCCCTTCTCTTTCGGCCATCCCAG

TATCGAGTCAGTCTCGTACCGGTTCACCCATCAGAATCAGCTGCCGAACGACAGCTCTCGCCTGGTTGACGCCCACCGTCGCGCCCAGGTGAACGAGCTG

AACCAGCGTCTGAACGAGCTGAGCGCGAAAATGGAGTTCGAGAAAGAGAGGGGGAGGAAGTTGGATGAGATGATAGCGGCACGCAAGGAGAAGGGTTGGT

GGGAAGCGCCGGACGAGGAGCTGAACAAGGAGCAGGTCGGACATCTGAGGTCGTGGTTAATCGATGCGCAGTTCAAGATAAGCAGTCGCCTGAACGAGCT

GTGTGCCGGGGCGTCGTCGTCGTCATTTCAGGTGGAAAATACACCTCAGTTGCCTCATCCATCGACTAGTGTTGGAATAAGTTTCGCTAGCTCTTCAAGC

CCTACTGGATATGGTCATGGAGGCATGCCTTTCTAA

>EuMADS48

ATGGTGAGAGGGAAACTGCAGTTTAGGTTGATCGAAGACCGGAACAGCCGACAGGTTACCTTCTGGAAGCGACGCTCTGGATTGATGAAGAAAGCTCGTG

AGCTCTCGGTGCTCTGCGGCGTAGACGTCGCGCTTCTCATCTTCTCCGATAAAGGCAAGCTTTACGAGTATAGCAGTAATAACTGCTGGGGAGAAATTCT

TGATCGCTACCAGATATCTAAAAATGTTGCACAACTTCCTGCAAGCAGCATTGATAATAAGATGACTGCTGCTGGATATCTGATGGATCATTTGTCAGAC

AAGGAAGTCCTATCAGTCATCCAAAGGCGCCTTGAAGGAGGAGGGCATCATTTCAACTTGACGAAGCTCGATCAACTGGAGCACCATCTGAATGCCTTGG

TCGGAAGCATTCAAATCAGAAAGAAACAACTGATGGCTGAACGTCTGACACCCCCCTCTGAATTGATGGCAGCTACGATGAACAACAGCTACTACCCTCA

CCAATTCCCGCCTGCCCACTACCCAACTCCAGGGATGGAGACGCTCGCTCCAAATATCGCCGGCATACAGATGGGACCGGCGATGAACAACCAGTATCTC

AACCAATTCCCGCCCGCCTACTACCCAAACGTAGGGATGGAGACGATCAACACGGGCTTTGAAGTCTTCGGAGTGAACAGTGATTCTTTCGGGAAGCTCA

CCTAA

>EuMADS49

ATGGGGAGAGGTAGGGTTCAGCTGAGACGGATCGAGAACAAGATTAGCCGTCAGGTGACGTTCTCGAAGCGGCGGTCGGGGCTGTTGAAGAAGGCTCATG

AGATCTCCGTTCTCTGCGATGCTGATGTTGCTTTGATTGTGTTCTCAACTCTGGGGAAGCTCTTTGAGTACTCCACTGACTCCAGCATGGAGAGGATACT

AGAAAGATATGAGGGCTACTGTTATGCAGAGAAACAGCTTAAGGCACCTGATCAATCCGAAGGCCAGGCATATTGGCCTTTCGAGTATCCCAAACTCAGT

GCTAAAATTGAAGTCCTACAAAGGAACATAAGGCACTATGTGGGAGAAGAACTTGATCCCTTAGGCCTAAGGGAGCTTCAGGGTTTGGAGCAACAGCTCG

ACACTGCTCTCAAGCGGATTCGAACAAGAAAAAACCAACTTATGCATGACTCCATTAATGAGCTCCGCAAGAAGGAAAAAGCATTGCGGGATCAAAACAA

CCAACTGGAAAAGAAGGTATATATAGCACAAGGTGAACTGTAA

>EuMADS50

ATGGGGAGAGGGAAGATTGCAATAAGAAGGATCGATGACACGACGAGCAGGCAAGTGACTTTCTCAAAGAGAAGGAATGGGCTTCTGAAGAAGGCAAAGG

AGCTTTCTATACTCTGTGATGCTGAAGTTGGAGTCATCATCTTCTCCAGCACAGGAAAGCTCTATGAATTTGCAAGCACCAGCATGAAAACAGTGATCGA

AAGATATAGTAAAATAAAAGAGGAACACCATCAACAACTGCATGGTCCAGCATCAGAAGTCAAGTTTTGGCAAAAGGAGGCAGAAATGTTGAGGCAGCAA

TTACACAACTTGCAAGAAAACATCCGAAAATTGATGGGAGAAGAGTTGTATGGTTTGGGAGTCCAAGACCTACAAAATTTAGAAAACCAACTGGAAATGA

GTTTGCATGGTGTAAGAATGAAAAAGGAGCAGATATTGACTGATGAGATACAAGAGTTGAATCGAAAGGGGAGCCTTATACATCAAGAAAATATAGAGCT

GTGTAAGAAGGTAAACCTCATCCGGGAAGAAAACATCAAATTGTATAAGAAGGTTTGTGGAACAAGGTATCCAACTGCAGGAGATGGTAATGGTTTCATT

CCACATGGTTTTAGCATTCGTGAGAAGATGCATCTCCCCATTCATCTCCAGCTAAGCCCGCCAGACCAACAAATTTTCCAAACACTAGCAAGAGCAACAA

ATTTGAGGTGA

>EuMADS51

ATGGGGCGGGCAAAGCTAAATATGGAGCTGATAAGTAACCCTAATTCTAGGAAGACAACATTCCGGAAGAGAAAGGACGGATTAAAGAAGAAGGCTCGAG

AATTGTCCGTTCTCTGCGGCGTCGACGTCGGCCTTATTATCTACGGACCCAATCACGACGATCGTCCGGAGTCGCCGGATATTTGGCCGGCGGATCCCAG

TAAGATTAGGGAACTGATCAACAGCTACAGAACACAGCGGAACGAAGATCGCGTGAAGAGGACGTTCGATCTCTCGACGTTCTTCGACGACCGGAAGCGA

AAGGCGGAGGAATCGCTCGTCAAGCTGCGGCGGAAGAACGACGAGGCGAAGTTTCCGACGTGGGACGACCGGTACAACAACTTGACGGAAGGTCAACTGA

TGGAATTTTCCGAACTTTTGGATGGGAAGCTCGAATCGGCGAGGGCTAGGGTAGAGCTCATGAAGGTGAATCGGAAATGGACGTGTTCTGATATTGTTCC

GGCGCCGGCGATGGTGCCGACGCCGTTAATGATCGAGAACCCCTACGACGACCCGATCCAGAACTCGTATTCATTCGTGACGCAGCGATTGAAACTGGAG

GCAGTCATTCACCAGCAGGAGCCCCTTTCGTCGTACAATTTTCAGAACGAAATCGCGCCGCCGGTATGCTTTCAGGCAACCCATTTCGACCCGTATACAC

CGCGTGTCGGACCGATCTTTTATGACCCACTTTCGATGTACTACTGTGGTCCGGCGGCTCAGCCGATGCCGCAGTTCATGCATTATCCGGCGCCGATCGC

CGGTTCGTCGACCCAATTGCAGGCTTCGCCGATGGACGATTACTTTGAATTCAGTGATTTTCAGATGAAAAATTCAAAATAA

>EuMADS52

ATGGATAAGAAGAGCAGAGGCCGACAAAAGATTGAAATGATGAAAATGTCCAAGGGTAAGAACCTCCTCGTCACATTCTCCAAACGCCGCTCCGGACTCT

TCAAGAAGGCCAGCGAGCTCAGCACCCTCTGTGGGGCCGAGATTGCCATCCTCGTCTTCTCCCCCGCAAATAAGGCCTTCTCCTTCGGCCACCCTTCTGT

TGACGCCATCCTCAACCGCTTCCTCGGGTTCCGCGCCTCCCAAGGCTCTGGCGGGACCTCCCATCTCGTGGAGGCCCACCGGAGCGCAGGGGTCCGGGAG

CTGAACAATCAGATGACGGATGTGATGGGTAAACTGGAGGCGGAGAGGAAGCGCGGGGAGGAGCTGGAGAAGATAAGGAAAATCGGGCGGGAGAAGCAGT

GGTGGGAAGCTCCGGTGGAGACGATGAGGTTACATGAGCTTGAACAGCTGAGGTTAACAATGGAGGATCTAAAAAAGGAGGTGGGGAAACAGGCGGAGAA

GGTCATGCTGGAGGCTTCGAATCCCCCACCGTTCTATAATGGGGGGTCCGAATCAGGGTTGGGGCTGTCCATGACTCCTCATGGTTATGCGCTTGGATAT

GGAGATGGCTTTTTCTGA

>EuMADS53

ATGGCAAGGAAGGTGAGCTTGGGCCGTAGAAAGATCCCGATGAAGATGATTCCGGCAAAAGATGCTCGCCAAGTTGCCTTCTCGAAGCGCCGGGCTAGCC

TCTTTAGGAAGGCGCACGAGCTTTGTACCTTGACTGGTGCGCAGGTGGTTATCATTGTCTTTTCACCGGGTGGCAAGCCCTACTCATTTGGGCACCCTTC

TGTTGAGGAAGTCATAAATAGGTTCCTTAACCTGCAGCGCATGCCAAATCCCGGTGACCAGGATCATCAGTATGTGGCAAATGTGGCTAGTCTCAACCAC

CGCTACAATGAGTTACGTGACCTGCTGGAGGAAGAGAAAAAACGGGGTGAGAAGCTGAAGCAGATGGAGATGGAGAGGCGCAAGCACAGCTTGGTGGGAG

CACCTGTGGATGAGCTCAACGTGCCTCAGCTAATGTTCTCCAAGATGCTCATGGAGAACCTTCGTGACAAGGTTGCGAAGCGGATTGAAGAGCTTTCGAT

CAAGAGTTCTGTTCAGACAGCATCATCTTCTTTGGGAAAAGTTGTTGGAGATTTCGACCTGAATCTCCCCGTCCCAGATGAGATGGAGGAGTAA

>EuMADS54

ATGCTCTCTGTTCTTGAGGAGAAGAAGAAGAAGAGAGAGAAAATGGGTAGGGGTAAAATCGAGATAAAAAAGATTGAGAATGTGAATAGCCGGCAGGTTA

CCTTCTCTAAACGGAGAAATGGGTTGATGAAGAAGGCGAAGGAGCTGTCGATTCTCTGCGACGCCGAGGTCGGCGTCGTCATCTTTTCCAGCACCGGAAA

ACTCTACGAATTCGCCAGCTCCCGCATGGAACATATTCTTGCAAGATACAATAAATGTCCAGATTCTTCAGAAGTTCCTTCAGTTGAAAATGTCACTGAG

CATGAATCACCGCCTGAGGTTAATGCTTTAGTTACTGAGATTGTTAAACTACAGAAGCTATACCGGAGGATGATGGGCAAGGAACTAGAAGGGTTGAGCT

TTAAGGAGTTGCAGCAGTTGGAACATGAGCAAAATGAGGGCATATCATCTGTTAAGGATAGAAAGGAGAAAGTGCTTTTGGAACAACTTGAGAAATCAAG

ATTGCAGGAACAAAGAACCATGCTGGAAAATGAGAATTTGCATAAACAGATCGAAGAGCTTCAGCGTAACTCTATGCCTTATCTCGAGCCTCAATCTGTT

ATTCCAACGAAAAATTCTTTTGCCAGTTCGAGCACAGTCTGTAATTGCAGATCAGAAAAAGAATGCGATTCGGATACTTCTCTACGCTTGGGGTTTTCAA

GTGATTGTATTTATGAAAAGAGAAAATTACCAAAGATTGAATCGACCTCCAATGACTGGGGTAGCCAAAAGAGCTAG

>EuMADS55

ATGGGGAGAGGGAGAGGGAGAGGGAGAGTGGAAGTGAAGAGGATAGAGAACAAGATCAACCGTCAGGTGACCTTCTCCAAAAGACGGAACGGTCTGCTGA

AGAAAGCACACGAGCTTTCCGTTCTATGTGACGCCGAGGTCGCTCTTCTCATCTTCTCTAGCCGCGGCAAGCTCCATGAGTTCGGTAGCCATGGTGTGCC

CAGAATCCTCGAACGCTATCAACAATGCACCTTTCATTCTCAGGAGAAGAGTGTTCCCAGTGAAACACAGAGTTGGGACAAAGAGATGTCGAAGTTAAAG

GCAAAGTATGAATCTCTTCAACGCACTCAAAGGCATTTGCTTGGAGAAGATCTTGGGCCACTGAGTGTTAAAGAGTTGCAAATTCTTGAGAAACAGCTTG

AAGGAGCTCTTACACAAGCAAGACAAAGGAGGGCGGAGATGATGATAGAACAAATGGAAAAGCTTCGCGTAAAGGAACGTAAGCTGGGGGAAATGAACAA

GGAGCTCAAGATTAAGATTGAAGCAGAAAGGCATGGTCTTCAAGGCCTTCCATTTGCATGGATTTCCACTAGTACTTATAGTGCTGGAAATAGTAGCTTG

TCTATGCACCCTTCTCACTCCATTCCCATGGAATGTCAACCAGTCTTACAAATGCAAATGGGGAATTATCAGCTTTTTTCGGGGGAAGGACCATCGGGTC

ACGGGAACGGTGCCGCCGATGAGACCAACTTCGTTCAGGGTTGGTCGCTCTAA

>EuMADS56

ATGGTGAGAGGGAAGACCCAAATGAGACGGATAGAGAACGCGACAAGCAGGCAAGTGACCTTCTCCAAGCGCCGTAATGGACTTCTTAAGAAGGCTTTTG

AGCTCTCCGTTCTCTGCGATGCCGAAGTTGCTTTAATTGTCTTCTCTCCCAGAGGCAAACTCTATGAATTCGCTAGCTCAAGCATGCACGAGACAATAGA

GCGCTATCGGGAACACGTTAAAGATGTTCGAGTTGACAACAGTACTACTTCAACTGCACACAACGTTCAGCATCTGAAGCGTGAAACTGAAAGTCTGGCG

AAGAAGATTGAGCACCTCGAAGTAGCAAAAAGGAAACTCCTTGGTGAAGGATTGGGGACATGCAGCATTGAAGAACTTCAGCAGATAGAACAACAATTGG

AGCGTAGCGTAACCACTGTCCGAGCAAGAAAGATGCAGGTGTTCAAGGAGCAGATTAATCAACTAAAAGAAAAGGAGAAAATCCTGGCTGCTGAAAATGC

TGTCCTACAAGAGAAGTGCGGGAGAGAGACACCGCAAGGATCGGATGTAGAGAGAGAGAGTTTGCCAGAGAGCAGTGAGAAATCAGATGTGGAGACTGAA

TTGTTCATCGGATTACCAGAAAGGAGAGTCAACCGTATGCGCAAGTAA

>EuMADS57

ATGGAGAATCATGCCAAGATTGTAAGAGATTTTTCCATCCAGACGGCGATTATCCTGCCAATCCCGAAGGATGCTGCTGGCAAGGTGAGCGAGTTGAGCA

CACTTTGCGGAATCAGTGCGTGCGCTATCATCTATAGTCCGTACGAATCCCTGTCAGACATTTGGCCGAACACCTTGGGTGCACAACGCATTCTCGCCAA

TTTCAAGCACATGCCGGAGATGGAACAAAGCAAGAAGATGGTAAATCAGGAAGGCTTCATCCGGCATAGAATCGTTAAGGTGATAGAGCAGCTGAAGAAG

CTCCGCAAGGACAACCGCGAGAAGGAGGTAACAAAGGTCATGTTCCAATGCCTGACCGACGGGAAAGAACTTCAGAACTTGATCATGGTGGACCTCAATG

ATTTGGGGTGGATGGTGGATCGGTGTTTGAAGGATATCAAAGATATGATCCAGCTCATTTGTCAAAGATTCACCCTCCATTACCATTACCATTACCATTA

CCATTAA

>EuMADS58

ATGGTAAGCGAGTTGAGCACACTCTGCGGAATCAGCGCGTGCGTTATCATCTACAGTCCGTACGAGTCCCGGCCGGACATTTGGCCGAACACCTTGGGTG

CACAGCGCGTTCTCGCCGATTTCAAGCGCATGCCGGAGATGAAACAGAGCAAGAAGATGGTGAACCAGGAAGGGTTCCTCCGGCAGAGGATCGTCAAGGC

GACGGAGCAGCTGAAGAAGCTGCACAAGGACAACCGCGAGAAGGAGGTGAAGGAGGTCATGTTCCGGTGCCTGACCGACAGGAAGGAGCTTCAGAACTTG

ATCATGGCGGACCTCAATGATTTGGGGTGGATGGTGGATTGGTGTTTGAAGGAGATTGATCAGAGGGTTGAGTCGTTGAGGAAAATGACGCCGGCTCCGC

GAGAGGCGGTTGTTGAGGTTCCGGCGGAGGAGCAGAAGCCGGTGGTTGCTTTTGTGGCGGACGCGATGCAGAGGCCGGAGTGGTTTCTGGAGTGGATGAT

GAACAATCCGAGTGATGACATGAGCATGATGATGCCATTTGGAGATGGGCATGATGGTCAAGTGCCTTCTTTCCTTAAGAGAGAGAGGAGAAAGAAACTG

ACACCTCGTGATTAG

>EuMADS59

ATGGGTAGAGGAAAGATTGCGATCAGACGGATCGACAACTCGACAAGTAGGCAAGTGACTTTCTCAAAGAGAAGAAATGGATTGCTCAAGAAGGCGAGGG

AGCTCTCGATCCTATGCGATGCAGAAGTCGGCTTGATAATCTTCTCTAGCACAGGAAAGCTCCATGATTTTGCTAGCACAAGCATGAAATCTGTGGTGGA

ACGATACAGCAAACTGAAAGAGGAACACCAGCAGCAGCAGCTAATGCATCCTACTTCTGAGGTCAAGTTTTGGCAAAGGGAAGCGGCAAGCTTGAGACAA

CAGCTAAAATACTTGCAAGAAAGCCACAGGCAGCTATTGGGAGAAGAATTATGCGGCCTGAACCTCAAAGATCTACAAAATCTGGAAAATCAACTTGAAA

TGAGTTTGAAAGGTGTTCGAATGACAAAGGATAAGATTCTAACTGATGAGATTACAGAACTAAACAGAAAGAGAAGTGTCATTCATCAAGAAAATACAGA

ATTGCATAAGAAGGTTAACCTCTTGCATCAAGAGAATACAGAATTGAAGAAGAAGGTTCATGGAGCAAGTAGCATGAATGAAGCAAAAAGAAACAAACAT

GTTCCATATGAACTCGCCAATGGATATGACTTGCATGCACCCATAAATCTCCATCTAAGTCAGCCAAACTCTCAGGATAATGAAATACCAGCAGAAGTAA

TGAAGCTCGGGCTATGA

>EuMADS60

ATGCCGAGATCGACTAAGGGTCGCCAAAAGGTTGCAATGGAGAAGATGCAAAGCCAGAGTAACTTACAGGTGACCTTCTCGAAGCGCCGCTCCGGCCTCT

TCAAGAAGGCGAGCGAGCTCTGCACGATGTGCGGCGCCGAAATGGTGCTAATCGTCTTCTCTCCCGGCGATAAGGTCTTCTCCTTCGGCCACCCCTGCGT

GGAGACCGTCGTCAACCGATTCCTCTCCAACAGCAACGCTCCCCCGGTTGATAGTACCGGCACCGAAGCGTTCATCGAGGCTCGCCGCAACGCCGGAATT

CGCGATCTGAACGCCCGGCTCGAGCACGTGGAGCGCGCGCTGGAGGCGGAGAAGAAGCGTGGGGAAATGCTGGATCAGATCAGGAAGGGTTCCCGGGGCC

GGAACTGGTGGGAGGGTCCCGTCGAAGATCTCAGCCTGCAACAGCTCGAGGAATTGAAGGGAGCCATGGAGAATCTGAAGGTGAAAGTGAGCCAACAGAA

GCAGAAGCTGATAGTTGAGGAAGCTTCAAACATATTCCCGGGCGCCATCAATGGGGGCGCAAGTAGTTCTTTGGGGGGGGGTGTTTATCAATTTGGTGCC

AATAGTTCCTCCATGACCATGCCTTCTCAAGGGCTGCTTAACCGTGGCTATGGCCGTGGATTTTACTGA

>EuMADS61

ATGGGTCGAGGAAAGATTGAGATAAAGAAGATCTTGGATGCGAATGCGAGGCAAGTCACTTTCTCCAAGAGACGCAATGGGTTACTGAAGAAGGCTAAAG

AACTCTCCATTCTTTGCGATGCCGAAGTTGCGGTTATCATTTTCTCGAATACTGGCAAACTTTTCGATTTCTCGAGTACTAGCATGAACCAAACACTTTC

AAGATATGACAAATGCCAACATTCTTCTGAGGTTTCCCCAGTAGAACAGATAACTGAGAATCAGGAGCTTACGAAGATTAAAGATATGGAAGAGGAAATA

GCAGAGCTCAAATCAAGACAACTGCAATTGTTGGGTAAGGATCTTAGCGGGGTGGGCTTCAATGAGCTACAGCAGTTAGAACAGCAATTATACGGAGGGT

TGTTATCCGTGATGAACAAGAAGGAGCAATTACTGAGGGAGGAAATAGGAGAATCAAGAGCGCAGGAGGAAAGGGCAATGGTTGAAAATGAGTATTTGCG

CAGAAAGGTAGAGGAGCTTGAGAAATTGGTTCCCTTAATGAACAGTAGGCCAGCGCCAGTGCCTCTGCCTTTGCCCCAACAATATATTGGTGGATATGGG

CCAATGAAACAACAGGACGACATAGCCGCCATAGCCGCCACCACCACCACTACTACTGCTAGTTGCCTGGAAGTATTCAACTTCAATGACGATGAGACTG

CAGCAGCAGCAGACTCTGACACCACCTTGACGCTCGGGCTCTCATATAGAAAGAGGAAGTCACCTGAGGAGGAGAAAAGCGAGAGTACAATGTTTTACTG

A

>EuMADS62

ATGAGAAAAGAAATTGTGAACCTTGGAGAACTGGTTGACGATAGTAGAGGAAGATTCAGAAGGACAATTCATAGAATCGCCATTGTTATCATCAATTTTG

ACCTCCGATTTCAGAAGGAGGATTCACGGAATTGCTTTGTTGCCGTGGTCGTCGCCTCTCTCTCTCTCTTTCTCTCAAATTGTTGCCAGATATATTCAAG

ATCTGGCCACGACGGTGGAAGCACCGTTCTCGTGACCGGCACCGTTCTAAATGAGTCATCGATTGGCTGGACGACAAGGCAATGGCGAAAATCGTCGATC

GACGTTGTGAAGACCGCAGTGAAAGCGAACATATGTGTTGAAGACTCTGGAATTGTTCACGATGATGTTGATTATTTTAGAAATTTGCTTTATGTTCATG

AAACTCTAACGGCAAGATCGGCGTCGTCGTACCATCCAGAGCCATGGCGTAATCGGATAACTGAAATCGATTTGAGGAGGAAGATTGACGATCCCTTGAA

GTATGTGTACGCCGGTTTTCCAATCGTGAACTTTGTCGATCTGGTCTACCAATTTCTCGACGTGAGCTTTGAGGTTGTTCTGGGTAGCAAAAGAGGCGAG

AGAGAGAGAGAAGACTGGTGCAAGCCTGGAGCAAGCGGCGATTGTAGAGGGAGAGGAAGATCATTAAAAGCAATCTTTCCTCTTTGTTCATGGAGTTCTT

TTCGCCGATATGTACGTCTCTCTTCACTGTGCCTATTCACTCAGTCTCTCCACTCTCTTTCTCTTGCCGACGCGAAAGACATGGTGACCCACCGTCCCTC

TGCAGCCGGCAAGAAAAGAAAGCTCTGCATACTGAAGAAGGCGATGGAGCTGTCCATCCTTTGCGACGTCAAGGCCTGCGTTATTCTTCAAAATCCCGAT

GGTAAGTTCGAGACTTGGCCTGAAAATCGGACAGAAGTCGAAGCCACAATCAGTGCCTACTTCAAGAGGATGAAAGAAGGGGAGAAATTCAGGAAATTGA

AGGGCCAAAAGAGTTGTAATTCTGGAGAAAAAACGATCGGAGATGGTGCCGGAGAAGGGAATTCGAAGAACGCATTTGGCGATTTGGATGACGGATATCT

CGACGGCTTGTCGAGGGATTCCCGGAAGACTTTCTTGAGAGAGGTTGAATCCAAAATCAAGGAGATAGACAACCGAAACGATTTCTCGAAGAACAGCGGT

GTGATCGATAATGAACACCAAGTGTTCGATGAAATTTCCCAACCAGAAAACCCTAGTGCGTTCGTGTATGATGATTTGATCAACACGGATGAGATCTTTC

CTGAATTTTTGCAACGTAATATCGACCCCATAAACATCGGAGACTATGAATTTGGCTGCGAACAGCCGCCATTGAATATCTTGAATGGTCCAGAGCCCTT

TCAACCCTCATGCTAA

>EuMADS63

ATGGGGCGGAGGAAGGTGGAGATGAAGCGGATCGAGAATAACAGCAGCAGACAAGTCACGTTCTCCAAGCGGCGAGCCGGATTGATTAAGAAGGCTAAAG

AGCTCTCCGTACTCTGCGACGTCGATCTCGCTCTCCTCATTTTCTCCAGCCGCGGCAAGCTCTACGAGTTCTCCACCACTCGCAGTTCCATCGAGATCAT

TCAAAGATATGAAAAGCTCCTCGAAGAAGGAAAGATTTCAAAAGGAGAAGGTTATGAAGCAGAGAAATTGCACTCTGAATGTGTAAATTACCTGCAAAGC

GGAGAGCTGCTGCAAATGGTTGACAAGTCCCTTAAGGAACCTTATGTTGAGGAGATAAGTGTAACCGACCTTGTGCAACTAGAAAGCCAACTTAGTGCTG

CCTTAATTCTAACAAAATCGAGGAAGACACAGTTAATGATGGAATTCATACAAAGTCTACGTGAAAAGGAAAGGATGCTGAGAGAAGAAAATGGTCTTCT

CGTGAAAGAGATAGCTGCAATGGAGGAAGACAGTAACACAAACGAGCTGGTGATCTCTAATCCTCCTCAACGCCAAACACTACAGTTGCTCTAA

>EuMADS64

ATGGGGAGAGGTAGGGTTGAGCTGAAGAGGATAGAGAACAAGATCAACAGGCAGGTGACCTTTGCTAAGCGAAGGAATGGGCTTTTGAAGAAAGCATACG

AGCTTTCCGTGCTTTGCGATGCTGAGGTCGCTTTGATCATCTTCTCCAATAGAGGAAAGCTGTACGAGTTTTGCAGCAGCTCAAGTATGCTGAAAACACT

AGAAAGGTATCAAAAGTGCAACTATGGAGCACCAGAGGCCAACGTATCCGAAAGGGAGGCACTAGAATTAAGCAGTCAACAAGAATATCTGAAGCTCAAA

GGGCGTTATGAAGCATTACAACGATCCCAGAGAAATCTTCTTGGGGAGGATCTTGGGCCACTGAACAGCAGGGAACTCGAGTCTCTAGAGAGACAACTCG

ATATGTCACTGAAACAAATCAGATCAACGCGCGTAAGTACTCGATACATCCATTTCTTGATTTTCCAAAATAAAAATGAACAAGCATTGAATGAAGCGAA

TAAAACTCTGAGGGATAGGCTGATGGAACAAGGAAACCAAATAAATTCATTCCACTGGAATCCAAACGGGCAAGATTCCGGCTATGGGAGACAACCGGCG

GAGGCTGCTCAAGCTCAGGCCGATGTCTTCTATCACCCCCTCGACTGTGAACCCACTTTACAAATAGGATATCCGACAGATCCAACGGCAGCAGCAGGCC

CAAGCGTCAGTAACTACCTGCCGGGATGGTAG

>EuMADS65

ATGGGGAGGGGAAAGGTTCAACTCAAGAGAATTGAGAATAGCATCAATCGCCATGTGACTTTCTCGAAACGGCGTTCCGGTTTGCTCAAGAAAGCACATG

AGATCTCAATTCTTTGCGACGCGGAGGTCGCTTTGATTGTCTTCTCCACAAAAGACAAGCTATTTGAGTATGCGACTCATTCCTGCATGGATAGGATCCT

TCAGCGGTACGAAAGATACTCAAATTCAGAGAGGCAGTTTGTTTCACCTGATCTTGAATCTCAGGGAAGCTGGACCTTGGAACATGCAGAGCTCGAGGCT

AGGAGGGAAGTTCTAGAGAGAAATCACAAGCGTTACATGGGAGAAGACCTAGATTCCTTGAGTCTAAGAGAGCTTCAGAATTTGGAGCACGAACTTGATG

TGGCTCTTAAAAATATCAGGTCAAGGAAGGACCAGCTCATGTATGAATCCATTTCCGTGTTGCAAAAAAAGGATAAGGCATTGCAGGAGCAAAACACCTT

GCTTTTAAATAAGATAAGGCAGAAGGAGGAGGAGCTTGCCCATCAAGCGCAGTTGGAGCACAACACTAACTATGCGAACTTCCCCCCTCATGTTCTATCA

CATCCACTGTCCAACATTGGAGATGCATACCATGCTGAAGGGAGTGGAGACGTTGAAGGCATACCTCATCAACATCAAATTAATTCTTTCATGCCCCCTT

GGATGCTCCGCCATATAAATGGATAG

>EuMADS66

ATGAAGCGGATAGCGAATAAGATAAACAGACAAGTGACGTTTTCAAAGAGGAAGGGAGGGTTAATGAAGAAAGCTAATGAGATATCGGTACTCTGTGATG

CTGAGGTGGCTCTCATTGTCTTCTCCCACAAGGGAAAGCTCTTCCAGTACTCCAGTACTCATTCTTGCATGGAAGGCATTCTCGATCGATATGAGAGATA

TTCCTTCTCTGGTAGAGAGCATGTCGTTAATGAAACAAAATTATCGGGAAGCTGGACCATGGAATATACTAAAATGAAGGCCAAAATGGAGCTTTTACAG

GAGAATATCAGGAGAAGACAATGGAAGATCAAAACAATAGAGGAGAATGAGAAGACAAAGGCAAATCTGCAAGAGCAGCAAAACCATGGCCTTAATTCAT

CATCATTCTACATGGCCCAACCACTTCTTCCAAATCTTGACATTGGGGGTATTTACCAGGAAGGAGGAGGAAGAAGGAATCATGAACTTGACCTTACACG

TGAGCCACTGTTTCTGACAAACTCATGTCAACTGGGATTCTTCCCCTCACATTAA

#amino acid sequences of MADS-box genes in Eucommia ulmoides

>EuMADS01

MAREKIQIRKIDNTTARQVTFSKRRRGLFKKAEELSVLCDAHVALIVFSSTGKLFEYSSSSMKEILERHNLHSKNLEKLEQPSLELQLVESSDCTRLNKE

VAEKTHQLRRMRGEDLQGLSIEELQQLERSLEVGLSRVIEKKTEKIMKEISHLQQKGLQLMEENELLRQQVMNVSNGRDPVGADSYNVVYEEGQSSESIT

NVCNSSEPPQDSESSDTSLKLGLFYIRCKINPTISSLHIEPLVGLPSDSLSLCGY

>EuMADS02

MTIGKKRKNCIIKKTMELSILCDVKACIIISNADGKFETWPENRTEVEGAIGAYFKAMKEGSRFRKSKGQKSNVTAELSTGTRTENTGGVVGEGALKKVF

GDLDDGFLDGLSMDSRKSFLMAMESKIKKIDKRREFLKNGGLQGNEQKLFEEILPKSPLSFPYGDWGGNIFEIFPEIYEPLPTDFEAENIGDIYETSSSP

LANFLNDSVNPDAGITVGGNQWIPPLDDVSFDWTTHEMILN

>EuMADS03

MGRGRVELKRIENKINRQVTFAKRRNGLLKKAYELSVLCDAEIALIIFSSRGKLYEFCSTSNMLKTLERYQKCSYSTAEVDQAGKEMEESSYREYMKLKT

KYESLQQYQRQLLGEELGPLNIKELEQLEHQLDSSLKHIRSTRTQTMVEQLADLQAKEKMWLEANKSLERKLEDIYAENQLPPPWAGVEQSSSFEPHHHQ

QAHSQAFFQPLQCDPTLQIGYNQVGSSHMATSSHAQNVNNFLPGWML

>EuMADS04

MGRGKIVIKRIDNSTSRQVTFSKRRNGLLKKAKELAILCDVEVGVMIFSSTDRRSGSELSEPPVHRIEKLEGDFVGGREIRGRICRKLKALMAER

>EuMADS05

MIVAQPPPCLHTKKPPPKSRIFFIWKSRKLGMGRGKIVIKRIDNSTSRQVTFSKRRNGLLKKAKELAILCDVEVGVMIFSSTDRRSGSELSEPPVHRIEK

LEGDFVGGREIRGRICRKLKALMAER

>EuMADS06

MAFRDQETEASSQRKMGRGKIEIKRIENTTNRQVTFCKRRNGLLKKAYELSVLCDAEIALIVFSSRGRLYEYSNHSVRSTIDRYKKACVDSTSSVTVSEA

NTQFYQQESTKLRRQIRDMQNSNRHILGEAISSLSFKELKNLEGRLEKAIGRIRSKKNEMLFAEIELMQKREIEMQNFNMYLRAKIAENERAQQQMNLMP

TEEEAAAGRSDYHQISYDARNFLPVNLQLEPSHTQYSRHGETTLQLV

>EuMADS07

MGRVKLKIKRLESTSNRQVTYSKRRSGILKKARELSILCDIDIILLMFSPTGKPTLFRGEHSNIDEVIAKFSQLTPQERAKRKMESLEVLKKTFKKLDHD

VNVQDFLGQSTQSVEELSHHARTLRAQVEEVHKRLSFWSNLEKIEDIEHLGQMEDSLRESLGRIRIQSENFGKHQLMPLDCSTQFQHGMHLPMMIGGSTQ

EAQTLSWLPNNENHHMILSEEPNFLLQRELECSRDATLPTYSGFFCNGKQIDINNTGQVEDTRQDGVTLNDWNHTACLRQKLNEEFAYNPFSNLELQEPE

KLKPGTDINLQQNSFDYQINGNFELPRPAYSNVPVWVTPSGPCGITMFNEKAYSQQPTQL

>EuMADS08

MGTGKKKIEIKRKEKESARVVTFSKRRTGLFKKAGEFHQLTGTHVAIMVFSPAGRPYTHSEPSSFEAIAQQYLKSGSGDDDDHQWRSFMDSIRPESMETE

GDLLEMKMALEDLRKKLIQKAEDEFIASLLALENPKD

>EuMADS09

MKRIENPSSRQVTYSKRKDGLVKKANELSVLCDTDVALIMFSPTGRLTSFARNGRVEDIFLRFVNRPDELKGGPIWNEEYLSLRLKQLKYEGEMLDKIST

IEALEEKLDKLNRRQREAHEKMRYYDPDVEKIGSVLEAGVHQQFLSCALQRVELSKAKLLGSGNVSQRPESIEVATGYTEDTSSFTNESSDSNRNRVRPP

NELNDEGTHQPAGPHLSLGFIQAQKQWKLLG

>EuMADS10

MEFCNQESEISPHKKMGRGKIEIKKIENTTNRQVTFCKRRNGLLKKAYELSVLCDAEVALIVFSNRGRLYEYASNSARSTIEKYKKSCIDSTSIGSAFET

NTQFYQQEATKLRRQIRDMQNSNRHIMGEALSSLSFKDMKNLESRLEKAIARIRSKKNEMLFAEIEHMKKREIELQNSNMYLRAKIAENENVQRKMNVMS

GYEYQSDMTSQPFDGRNFIPVNLLEPNPNYSRQDQTALQLV

>EuMADS11

MEMVRGKIQIKRIENSTSRQVTFSKRRTGLLKKAYELSVLCEAEVGLVIFSQNGRLYEYSSSDMRMIFKRYLEISAKEDQSSNPDTQRQLQHLKHESAKL

AKKIEHLENSQRKLLGQNLATCSIDELQEIDGQLERSLKNIRLKKAQVFTEEIDRLKEKERLLLEENARLCDECAVKQLKPPTKEKEIAKCNHNAEVETE

LFIGLPEMRCS

>EuMADS12

MVRGKIQIKRIENSTSRQVTFSKRRNGLLKKAYELSVLCEAEVGLVIFSQNGRLYEYSSSDMHMIFKRYLEISAKEDQSSNPETQQQLQHLKHESAKLVK

KIEHLENSQRKLLGQNLATCSIDELQEIDGQLERSLKNIRVKKAQVFTEEIDRLKEKERLLLEENARLCDECGVKQLKPPTKEKEIAKCNRNAEVETELF

IGLPEMRCS

>EuMADS13

MDFQDQSREISPQRKMGRGKIEIKRIENTTNRQVTFCKRRNGLRKKAYELSVLCDAEIALVVFSNRGRLYEYANDSVKATIERYKKASSDSPNTSSISEA

NAQFYQQESSKLRVQIANLQNQSRYMLGESLSSLYVKDLRNLESKIERGISRIRSKKNELMFAEIEYMKKREFELHNNNHYLRTKIAENDRAQQQQLHMN

LMPGVPEYELPLATQPPFDGRNYLQTNVNQYAGSSRQDQTVLQLV

>EuMADS14

MTRTKLKLGVIPSESTRKLTLKRRRANLFKKTEELSVLCDVEAGIIVYSPEEETPAVFPSLEAVAGMVARFKSLPEMERSKKAVTNEGYLRDRAASEGGK

VRSEEERNDEKEMQVIMNQLIGGMDLSELDERQLNGMFGYAARRLKRLEVRDRELERLDPPLPPPPPPPQSEAEVVADRAQGGSSGGGDVVDRWFVGDEL

PDDLNQSWPHIFSP

>EuMADS15

MAKKPSLGRQKIKIENIQTKTHLQVTFSKHKSGFLKKASEQCTICGVEIAIVVFLPAGKVFCFGHINVDSIVDKFLGLNRPLNYIDIPNFVEAHKTVRIP

ELICQLTQILNELEFEKNHGETFDLMRKASRNGQNWWEGPIDEMDLNELVQLRDSMEELKKNVTNQANMLLVEASRETSSF

>EuMADS16

MVRGKTQMKRIENSTSRQVTFSKRRSGLLKKAFELSVLCDAEVALIIFSTRGKLYEFASSSASKTIERYEKNANDQADVIVKAVEKNIQNLKEETSTVTK

QIELLEDSKRKLLGDGLESCTTEELEEIEGQLERSLSNIRARKNQLLKEKIEQLKEQEGMLAKENENLRKVQFLELSLARLEFPTRQVFEVETKLFMGPP

>EuMADS17

MTRKKVKLAFIANDASRKATFKKRKKGLMKKVSELTTLCGIQACAIIYSPYESRPEVWPNVLGAQQVVAKFKTMPEMEQSKKMVNQEGFIRQRIGKASEQ

LKKLAKENREKEMTEVMFRCLTGSGLQNLMAADLNDLGWLIDRNLKEIGRRIDDLRKNGPGSGSSGTMAAAATMVEAVEQKPEMDQFAVDVMTQRPPWLT

EWMNNPIENLGFGHGDETMMMMMNNMPFGDNLNPMWSSSFFP

>EuMADS18

MGRGKIVIRRIDNSTSRQVTFSKRRSGLLKKAKELSILCDSEVGLIIFSCTGKLYEFSSTSMKSIIERYNKMKEEQYHLVNPASELKLWQREAASLRQQL

HDLQEKHRKLLGEKLSGLGINELQSLENQLEMSLKGVRMKKHQILADEIGELNRKGSLIHQENVDLYTKVNLMHQENIQLQTKVHGASGMQEGRTSHISY

GLNNIYDLYAPINLQLSQPKQWNKEIPAKATKIGLQLQ

>EuMADS19

MGRVKLAIKKIENTTNRQVTFSKRRNGLIKKAYELSVLCDVDVALIMFSPSGRLSIFSGNKSIEDIVARFLNLPEHERGRINSQETLQRALGKLKTRSDP

TNQTTSPGSVDSQLEEIQLEIIRCKTKLEEMEKRLRIFEGDPVEITTLCEAEFRERILKETLKNVHLRKKVLEEEYNKSPGDGQQTPETVNMIDLSSRNP

NNVLDWLPQGDPQVQILNFLDSNGLLSQRDDENIFGQSSSVVNGQNLHVDEGHLSPSSGLEDNSSLQQPEFGHVIDVNLSPWTPFYSTGNGHFSASQPRE

HALLELFLSHFRQ

>EuMADS20

MGRVKLEIKRIENNTNRQVTFSKRRNGLIKKAYELSILCDIDIALIMFSPSGRLSHFSGKKRIEDVFARFISLPDDERERAVAEEQGGEHYRNALCNREY

FIRTLKQLRFENDIARQLANSTAINSEIEELRQEISGLQQQLLMSEDQLRIFEPDPWKMASTEEFESCEKQLEDTLARVVERKKFLMSNNLSSYASPLQE

LPTSFETEAVRWSLEHGHNHAPIFDSSAPLIPVRDNMSAIYTQLSQESSSNMEQQQHNLEECHANNPSDGDFLQWQHAGTQMVLHPPHIPPSLFSSGLPI

QTAMMQLDQVEIPFNGSSVQSENGAANYGN

>EuMADS21

MVRKKIQIKKIEKTAARQATFSKRRRGLLNKASELAVLCDAQVALIIISESGNLFKYSSSSMSDILMKYNFQSNLEKLVCPSRKLQQIGFGDESAKKMRQ

LRQMMGEDIQRLNIEELQQLQNVIEVGLTRVIRTKNERILDGIAVLQRKHAEIMEEKEQLRNKTTMILNEERFDLGIISHENPEKENWLDTSLQLARVYD

SYKP

>EuMADS22

MVRKKIQIKKIEKTAARQATFSKRRRGLLNKASELAVLCDAQVALIIISESGNLYKYSSSSTSDILEKYNFQSNLEKLVGPSRKLQKIGLGEESTKKMRQ

LRQMMGEDIQRLDMEELQQLQDVIEVGLTRVIKTKNERILDCIATLQRKHAEIMEENEQLRNKTTMILNEERFDLDSISCENREKENWLDTSLRLARVYD

RHKT

>EuMADS23

MVRKKIQIKKIEKTSARQATFSKRRRGLLSKASELAVVCDAQVALVIISESGKLFNHSSSSGCSITSLLMPLRNRRKTPLHSIQELFPSPADDTNGDTKL

DDDAEHNGFKWFVKVHPARFNEGMTVNRKSRNKLIRGSHVEPKKSRKCGKQKLDTEFGVVRGIDLKNVHAVSTYFL

>EuMADS24

MLFFYLFINICGIEGGKMAREKIKIKKIDNITARQVTFSKRRRGLFKKAAELSVLCDAQVALIVFSTTGKRFEFASSSMKDILNKYKKHSDNIDKIDPPS

LNPQVEDNDDHARLSKEVADKANQLRQMRGEDLQVLSIDELQHLEKMLEGGLSRVLETKGERIMKEISNLQRKGAQLMEENKQLKEKMDMLCKGKKPLLI

ELENTVIPEEQGQSSDSATNAYSCNSGPPTDDDFSDTSLKLGLPFN

>EuMADS25

MAREKIKIKKIDNVTARQVTFSKRRRGLFKKAAELSILCDAQVALIVFSTTGKRFEFASSCMKDILNKYKMHSDNIDKVDPPSLNPQVEDDDDHARLTKE

VADKTHQLRQMRGEDLQGLSVDELQHLEKMLEGGLSRVLETKGERIMNEISNLRWKMDMLWKGKNPLQIKLENTMISEDQGQSTNSTTNSGKKPLLIELE

NTTIPEDQGKKPLQIELENTTISEDQGQSTDSTTNAYNCNSGKKPLLIELENTTIPENQGQSSDSTTDAYSCNNGPSTKDDFFDTSLKLG

>EuMADS26

MAREKIKIKKIDNITARQVTFSKRRRGLFKKAAELSVLCDAQVALIVFSTTGKRFEFASSSMKDILKKYKMHSDNIDKVDPPSLNPQVEDNDDRARLSKE

VADKTHQLRQMRGEDLQGLGVDELQDLEKMLEGGLSRVLETKGERIMNEISNLQRKGAQLVEENEQLKEKMDMLCKGNKPLLVELENTTIPEDQGQLSDS

TTNAYSCNSGPSTEDDFSDTSLKLGGRRYIPNGIECRFSK

>EuMADS27

MALVGDRSIDDNDSELNNRCWCAIAVWSATVMELLIHYIRHVNLARFGSFFMPNRAKNVKPKLPKWEVKYRQYHHPRVHDLLATHRFQESTTNCDGCHAL

SLYGRSCEIRNSYQNDDRRVSFCLLHQNLHSQIFFIWKSRKLGMGRGKIVIRRIDNSTSRQVTFSKRRNGLLKKAKELAILCDAEVGVMIFSSTGKLYDY

ANTSSMKSVIERYNKSKEEPRQLANPISEVTFWQQEAAVLRQQLQNLQDNHRQMMGEELSRLSVTELQNLENQLEMSLRGVRIKKDQVLIDEIEELNKKG

SLIHQENVELYKKVYGTRRANGENRNALLTNGLSIGEELHASVHLQLCQPQQQNYETPAQATNLG

>EuMADS28

MGRGKIEIKRIENSSNRQVTYSKRRNGIIKKAKEISVLCDAQVSLVIFGSSGKMHEYCSPSTNLIDMLDKYQRQSGKRLWDAKHENLSNEIERIKRENDN

MQIELRHLKAEDITSMQYKELMAMEEVLENGLTSVREKKMELFNATKENDEILEEENRRLNYMLHQQEMEAMNNNMRGMPFPFRVQPSQPNLHEII

>EuMADS29

MVRGKIQMKRIENAANRQVTFSKRRNGLLKKAFELSVLCDAEVALIVFSSNGRLYEFSSSSINKTIDRYQKNTKNLGFCPKPVQENYQLLKDEAAKMTKK

IEVIEDYKRKLLGSGLDCCSTDELQNVEVQLEKSLRNIRARKNQLLKEKIQKMKEEELILAKENSQLKKVSFDISITTRVCICIYTCWFYSKVCLC

>EuMADS30

MGRRKVEIKRIENKSKRQVAFSKRRNGLMKKARELSVLCDVDVAVVIFSTRGKLYDFSGGHSLAEILQRHQSCAEAEGEISTGAHEAQVQPESSSFISSR

ELLQIVESFEEPNAKQLTVTDLEEVENQLDAALVQTRSRKTQLMMESLKDLRDEEKKLNEEKKSLQNEIANYRTQPLVTLSLL

>EuMADS31

MGRRKVEIKRIENKSKRQVAFSKRRNGLMKKARELSVLCDVDVAVVIFSTRGKLYDFSGGHRLSFDFSYKVDIHRPSTIGIFVNNVHNQSVNQRFLVPRI

TFCNDSVSK

>EuMADS32

MGRRKVEIKRIENKSKRQVAFSKRRNGLMKKARELSVLCDVDVAVVIFSTRGKLYDFSGGHSLAEILRRHQSCAEAEGEISTGAHEAQQIHPESSSFISS

KELLQIVESFEEPNAKQLTVTDLEEVENQLDAALVQTRSRKTQLMMESLKDLQDEERKLREEKKLMQNEIANCRTQPP

>EuMADS33

MGRGRVELKRIENKINRQVTFAKRRNGLLKKAYELSVLCDAEVALIIFSNRGKLYEFCSSSSMLKTLERYQESNYGAPETNVSTRDALEISSQQEYLKLK

GRYEALQRSQRNLLGEDLGPLNSRELESLERRLDTSLKLIRSTRTQCMLDSLTDLQTKEHALNEDNRALRQRLMEGNHINSFEWNQNAHDHVFGRQQAQP

HDNVFFHPLECEPTLQIGYPTDPITVTTAAGPSLSNYMGGWLP

>EuMADS34

MGRGRVELRRIENKINRQVTFAKRRNGLLKKAYELSLLCDAEVALIIFSNRGKLHEFCSTSSMAEILEKYRRCSYGAFEANQPPDHTQGSYQEYLKLKTR

VDVLEQYQRHLLGEGLGELGIKELEQLERQLDVSLKKIRSKETQFMLDQLSALQQKQQELLEANKALRSKIEESNATLQLAPWEARDCPMPFRHQPPQTE

EFFQPLECNNTSQIRYTSVLPQDELNNATSTRHAYNEMIPGWML

>EuMADS35

MRKGKTILERIPNETARRTSLRKRRDGLIKKARELSILCNVQLGVVVYSPDEDTPVTWPSQEVTAQTLARFQEVPEVEKSKKMVTHETYLADRVTKESEK

IRKFERDNDEKEHQLIMTQLVEGKSLNELEVMQLQGMLYFMAKELKKLEKRDQDLDRQAPPSHLPPPPPHLGHLPPPSQLGYHPHPPPGYHAYYPPPAPS

PQPTELDDAAQVQGITDPSTLVPFSMEYLMDDKWFMGTIFEYKDVPGPRGEGNVRLQPEYASSSGPSNVVDEHQNDPNQTWPKHFFP

>EuMADS36

MGRGKVELRRIENSTSRQVTFSKRRNGLLKKACELSVLCDAEVALIIFSPSGKAYQYASQDIGRTISRYRNETGFFNPNNQLFRSTEILRSELDELRRRV

ETMETRNKHVAGQDLNMLGMKDLKRLERQLRTGVERIRSKKRRIGSGYINSLKKKRRDLQEENTHLQKRLEELPELGGSSRMCGATPFDVFQRYAEEGTS

AI

>EuMADS37

MGRGKVELKRIENKINRQVTFAKRRNGVLKKAYELSILCDAEVALIIFSSRGKLYEFCSGSSMTKTLEKYQKCSFGSLEPDPMINGNQHSYHEYLRLKAK

VEVMQRSQRNLLGEDLGSLNSKEMEQLEHQLEISLKQIRSTKKLAFSASFFLFGGFFPNRMWKGVTVRSKTTLHRYQSSGYNNSDPITTKREIGRGLAYQ

RRPMCEEKIVGFIPH

>EuMADS38

MGRGRVQLRRIENKINRQVTFSKRRSGLLKKAHEISVLCDAEVALIIFSTKGKLFEYATDASMERILERYDRYSYAEKRLVAPDVESQGSWTFEHARLKA

RVEVLQKNQRHYMGEDLDSLSMKDLQNLEHQLDVALKHIRSRKNQLMHESISELQRKDKALQEQNNLLAKKVKEKEKMDLAQQDQVLGPSSNDLNSSFVI

QQPLPSLNIGSDAYEEAEGRREVEGIAASQHQTYVSMPPWMLHHVNR

>EuMADS39

MARGKIQIRRIENSTNRQVTYSKRRNGLFKKANELSVLCDAKVSIVMFSSTHKLHEYISPTTTMKEFFDQYQRTVGVDLWSSHYEKMQEQLRKLKDVNRN

LRREIRQRMGESLNDLKYDELCDLEQDMDNSVKIIRERKYKVITNQIDTYKKKLRNVEEIHRNLLHEFDLRDEDPHYGLVDNGGDYDTSAVLGFASAGPR

ILSLRLQPTQTNIHAGGGSDLTTFALLE

>EuMADS40

MVRGKIEMKRIEKATRRQVTFSKRRNGLLKKAYELSVLCDSDVALIIFSQTGRLYEFSSSNLQTIIERYRECEKDQNRDNGPEIERYIQHLKQESAGMAK

KIEHLELSQRKLLGQNLESCSVDELQDIGSQLEQSLKNIRRRKDDLLKEEIKQLKAKERFLLEEKGRLSEKCVAKTRQPSLWMNEGERCGQSSPSSEVET

ELFIGLPEMRCTL

>EuMADS41

MARGKVQMKRIENPVHRQVTFCKRRAGLLKKAKELSVLCDAEIGLFVFSAHGKLYELATKGTMQGLIERYMKSTGGMEPEQPNQTQLHDSKVQINLLKDE

IDVLQKGLRYLYGDDTENMTLDGLHILEKHMEIWISHIRSIKMEIMFQEIQVLKNKEGMLNTVNKYLQEMIDDHYETTNIVPVMTNTTYPLTIQNEIFPF

>EuMADS42

MNDNNMQKKKKTTQGRKKIEIKEIEGKSNKQVTFSKRRSGLFKKAGELCVLTGAEVSVIVTSNAGRVFAFGHPSVDAVVDRCLAGGSYKSDAIEKRYPYA

HEFNQHYDRVSEELEAEKKRREAIDEREPDGAAEDRAFWWSEPVDDMGLEELEKYLASMEELKQKVEMRAEELRMMKMAAAAAVGGGASSSYSNAGMNAL

VDHNHMVSQTAASYGNPMVPLEYDFGHGQI

>EuMADS43

MNDNNMQKKKKTTQGRKKIEIKEIEGKSNKQVTFSKRRSGLFKKAGELCVLTGAEVSVIVTSNAGRVFAFGHPSVDAVVDRCLAGGSYKSDAIEKRYPYA

HEFNQHYDRVSEELEAEKKRREAIDEREPDGAAEDRAFWWSEPVDDMGLEELEKYLASMEELKQKVEMRAEELRMMKMAAAAAGGGASSSYSNAGMNALV

DHNHMVSQTAASYGNPMVPLEYDFGHGQI

>EuMADS44

MGRRKAEVKMIENKSSRQVTFTKRRQGLLKKTHELSVLCDAQIGLIIFSSKGRLFQYSSHPHSMGDIIERYHQTTGSSIPHHDNQEEMWKEMRQLKQKTM

DLQLSLQRYKGDDLSFVRVEELDQLERQLEHSVKKVRARKFELLQQQLDNLQRSEKLLEKENQEMYQWLMNNQIQKHQAEVGQEDQQAVTELKFVEVQDE

QHQQQQVLEQFPFYGEEQPSDVLLQLATTLHDNPYRLQPIQPNLQDATANTILHKYGGYGSDHYRHHHDHYE

>EuMADS45

MGRVKLKLKRLENNTCRQTTFSKRKNGLIKKASELSILCDIDIVLLMFSPSGKPTLFKGQNSIEDIILKFAQLTPQERAKRKLESLEALKRTFKKLDHDV

NLEEFFGPSYQSVEDLTDRVNFYRTQLSEVQNRLSYWTNLDKIEGMQHLGQMEDSLKQSISQIRAHKENLVKQQELAIQCANQFENGMHLPYDLGMEQQF

QPSSWIQNDHSQNITLSTDLNLLSQRDSECSGVTSFGNCSNFFGVSEKLEMSKIGHENSSLGELCTNESLSLQMSEQQQQPSCYPSYGFGSLSGKTFQQN

IHENALNFCLDANFEFPQSGYEFSHQGWAASGPEGHAIQSFDENFFPQARFLALFVSFLWF

>EuMADS46

MENKRKKQTLGRQKIEIKKIETNSNLQVTFSKRRAGLFKKAAELSLLCGSDVAAFVNSPTGKVYAFSNLPVDVLLDRYLNQTAPSSSSSLSSLLPFSGPT

GPLHEVAKKKYMEVKTQLEVEEERERRKAAVTMVVAEGGGVGGGGDGFWWDQPTEGLGLQELEQYVAAMEEMKKKVVERAEELAVASASPSNLLISAFIT

EASTEENSLDFELMLFYQRLDHKVAGASYRKCSFGTIQLEQLLWDPPIGWWRGSILEPFILDHSQLGSKNLLFHHPKFVLKIGDFNPTYPVKFQQILFSK

FFVPNAWGRESSFVLDNNLLLGLGSDLSWW

>EuMADS47

MENKRSRGRQVIPMQKIENEEDRYATFSKRRKGLYKKASELCTLCEADVGIVIFSPTGKPFSFGHPSIESVSYRFTHQNQLPNDSSRLVDAHRRAQVNEL

NQRLNELSAKMEFEKERGRKLDEMIAARKEKGWWEAPDEELNKEQVGHLRSWLIDAQFKISSRLNELCAGASSSSFQVENTPQLPHPSTSVGISFASSSS

PTGYGHGGMPF

>EuMADS48

MVRGKLQFRLIEDRNSRQVTFWKRRSGLMKKARELSVLCGVDVALLIFSDKGKLYEYSSNNCWGEILDRYQISKNVAQLPASSIDNKMTAAGYLMDHLSD

KEVLSVIQRRLEGGGHHFNLTKLDQLEHHLNALVGSIQIRKKQLMAERLTPPSELMAATMNNSYYPHQFPPAHYPTPGMETLAPNIAGIQMGPAMNNQYL

NQFPPAYYPNVGMETINTGFEVFGVNSDSFGKLT

>EuMADS49

MGRGRVQLRRIENKISRQVTFSKRRSGLLKKAHEISVLCDADVALIVFSTLGKLFEYSTDSSMERILERYEGYCYAEKQLKAPDQSEGQAYWPFEYPKLS

AKIEVLQRNIRHYVGEELDPLGLRELQGLEQQLDTALKRIRTRKNQLMHDSINELRKKEKALRDQNNQLEKKVYIAQGEL

>EuMADS50

MGRGKIAIRRIDDTTSRQVTFSKRRNGLLKKAKELSILCDAEVGVIIFSSTGKLYEFASTSMKTVIERYSKIKEEHHQQLHGPASEVKFWQKEAEMLRQQ

LHNLQENIRKLMGEELYGLGVQDLQNLENQLEMSLHGVRMKKEQILTDEIQELNRKGSLIHQENIELCKKVNLIREENIKLYKKVCGTRYPTAGDGNGFI

PHGFSIREKMHLPIHLQLSPPDQQIFQTLARATNLR

>EuMADS51

MGRAKLNMELISNPNSRKTTFRKRKDGLKKKARELSVLCGVDVGLIIYGPNHDDRPESPDIWPADPSKIRELINSYRTQRNEDRVKRTFDLSTFFDDRKR

KAEESLVKLRRKNDEAKFPTWDDRYNNLTEGQLMEFSELLDGKLESARARVELMKVNRKWTCSDIVPAPAMVPTPLMIENPYDDPIQNSYSFVTQRLKLE

AVIHQQEPLSSYNFQNEIAPPVCFQATHFDPYTPRVGPIFYDPLSMYYCGPAAQPMPQFMHYPAPIAGSSTQLQASPMDDYFEFSDFQMKNSK

>EuMADS52

MDKKSRGRQKIEMMKMSKGKNLLVTFSKRRSGLFKKASELSTLCGAEIAILVFSPANKAFSFGHPSVDAILNRFLGFRASQGSGGTSHLVEAHRSAGVRE

LNNQMTDVMGKLEAERKRGEELEKIRKIGREKQWWEAPVETMRLHELEQLRLTMEDLKKEVGKQAEKVMLEASNPPPFYNGGSESGLGLSMTPHGYALGY

GDGFF

>EuMADS53

MARKVSLGRRKIPMKMIPAKDARQVAFSKRRASLFRKAHELCTLTGAQVVIIVFSPGGKPYSFGHPSVEEVINRFLNLQRMPNPGDQDHQYVANVASLNH

RYNELRDLLEEEKKRGEKLKQMEMERRKHSLVGAPVDELNVPQLMFSKMLMENLRDKVAKRIEELSIKSSVQTASSSLGKVVGDFDLNLPVPDEMEE

>EuMADS54

MLSVLEEKKKKREKMGRGKIEIKKIENVNSRQVTFSKRRNGLMKKAKELSILCDAEVGVVIFSSTGKLYEFASSRMEHILARYNKCPDSSEVPSVENVTE

HESPPEVNALVTEIVKLQKLYRRMMGKELEGLSFKELQQLEHEQNEGISSVKDRKEKVLLEQLEKSRLQEQRTMLENENLHKQIEELQRNSMPYLEPQSV

IPTKNSFASSSTVCNCRSEKECDSDTSLRLGFSSDCIYEKRKLPKIESTSNDWGSQKS

>EuMADS55

MGRGRGRGRVEVKRIENKINRQVTFSKRRNGLLKKAHELSVLCDAEVALLIFSSRGKLHEFGSHGVPRILERYQQCTFHSQEKSVPSETQSWDKEMSKLK

AKYESLQRTQRHLLGEDLGPLSVKELQILEKQLEGALTQARQRRAEMMIEQMEKLRVKERKLGEMNKELKIKIEAERHGLQGLPFAWISTSTYSAGNSSL

SMHPSHSIPMECQPVLQMQMGNYQLFSGEGPSGHGNGAADETNFVQGWSL

>EuMADS56

MVRGKTQMRRIENATSRQVTFSKRRNGLLKKAFELSVLCDAEVALIVFSPRGKLYEFASSSMHETIERYREHVKDVRVDNSTTSTAHNVQHLKRETESLA

KKIEHLEVAKRKLLGEGLGTCSIEELQQIEQQLERSVTTVRARKMQVFKEQINQLKEKEKILAAENAVLQEKCGRETPQGSDVERESLPESSEKSDVETE

LFIGLPERRVNRMRK

>EuMADS57

MENHAKIVRDFSIQTAIILPIPKDAAGKVSELSTLCGISACAIIYSPYESLSDIWPNTLGAQRILANFKHMPEMEQSKKMVNQEGFIRHRIVKVIEQLKK

LRKDNREKEVTKVMFQCLTDGKELQNLIMVDLNDLGWMVDRCLKDIKDMIQLICQRFTLHYHYHYHYH

>EuMADS58

MVSELSTLCGISACVIIYSPYESRPDIWPNTLGAQRVLADFKRMPEMKQSKKMVNQEGFLRQRIVKATEQLKKLHKDNREKEVKEVMFRCLTDRKELQNL

IMADLNDLGWMVDWCLKEIDQRVESLRKMTPAPREAVVEVPAEEQKPVVAFVADAMQRPEWFLEWMMNNPSDDMSMMMPFGDGHDGQVPSFLKRERRKKL

TPRD

>EuMADS59

MGRGKIAIRRIDNSTSRQVTFSKRRNGLLKKARELSILCDAEVGLIIFSSTGKLHDFASTSMKSVVERYSKLKEEHQQQQLMHPTSEVKFWQREAASLRQ

QLKYLQESHRQLLGEELCGLNLKDLQNLENQLEMSLKGVRMTKDKILTDEITELNRKRSVIHQENTELHKKVNLLHQENTELKKKVHGASSMNEAKRNKH

VPYELANGYDLHAPINLHLSQPNSQDNEIPAEVMKLGL

>EuMADS60

MPRSTKGRQKVAMEKMQSQSNLQVTFSKRRSGLFKKASELCTMCGAEMVLIVFSPGDKVFSFGHPCVETVVNRFLSNSNAPPVDSTGTEAFIEARRNAGI

RDLNARLEHVERALEAEKKRGEMLDQIRKGSRGRNWWEGPVEDLSLQQLEELKGAMENLKVKVSQQKQKLIVEEASNIFPGAINGGASSSLGGGVYQFGA

NSSSMTMPSQGLLNRGYGRGFY

>EuMADS61

MGRGKIEIKKILDANARQVTFSKRRNGLLKKAKELSILCDAEVAVIIFSNTGKLFDFSSTSMNQTLSRYDKCQHSSEVSPVEQITENQELTKIKDMEEEI

AELKSRQLQLLGKDLSGVGFNELQQLEQQLYGGLLSVMNKKEQLLREEIGESRAQEERAMVENEYLRRKVEELEKLVPLMNSRPAPVPLPLPQQYIGGYG

PMKQQDDIAAIAATTTTTTASCLEVFNFNDDETAAAADSDTTLTLGLSYRKRKSPEEEKSESTMFY

>EuMADS62

MRKEIVNLGELVDDSRGRFRRTIHRIAIVIINFDLRFQKEDSRNCFVAVVVASLSLFLSNCCQIYSRSGHDGGSTVLVTGTVLNESSIGWTTRQWRKSSI

DVVKTAVKANICVEDSGIVHDDVDYFRNLLYVHETLTARSASSYHPEPWRNRITEIDLRRKIDDPLKYVYAGFPIVNFVDLVYQFLDVSFEVVLGSKRGE

REREDWCKPGASGDCRGRGRSLKAIFPLCSWSSFRRYVRLSSLCLFTQSLHSLSLADAKDMVTHRPSAAGKKRKLCILKKAMELSILCDVKACVILQNPD

GKFETWPENRTEVEATISAYFKRMKEGEKFRKLKGQKSCNSGEKTIGDGAGEGNSKNAFGDLDDGYLDGLSRDSRKTFLREVESKIKEIDNRNDFSKNSG

VIDNEHQVFDEISQPENPSAFVYDDLINTDEIFPEFLQRNIDPINIGDYEFGCEQPPLNILNGPEPFQPSC

>EuMADS63

MGRRKVEMKRIENNSSRQVTFSKRRAGLIKKAKELSVLCDVDLALLIFSSRGKLYEFSTTRSSIEIIQRYEKLLEEGKISKGEGYEAEKLHSECVNYLQS

GELLQMVDKSLKEPYVEEISVTDLVQLESQLSAALILTKSRKTQLMMEFIQSLREKERMLREENGLLVKEIAAMEEDSNTNELVISNPPQRQTLQLL

>EuMADS64

MGRGRVELKRIENKINRQVTFAKRRNGLLKKAYELSVLCDAEVALIIFSNRGKLYEFCSSSSMLKTLERYQKCNYGAPEANVSEREALELSSQQEYLKLK

GRYEALQRSQRNLLGEDLGPLNSRELESLERQLDMSLKQIRSTRVSTRYIHFLIFQNKNEQALNEANKTLRDRLMEQGNQINSFHWNPNGQDSGYGRQPA

EAAQAQADVFYHPLDCEPTLQIGYPTDPTAAAGPSVSNYLPGW

>EuMADS65

MGRGKVQLKRIENSINRHVTFSKRRSGLLKKAHEISILCDAEVALIVFSTKDKLFEYATHSCMDRILQRYERYSNSERQFVSPDLESQGSWTLEHAELEA

RREVLERNHKRYMGEDLDSLSLRELQNLEHELDVALKNIRSRKDQLMYESISVLQKKDKALQEQNTLLLNKIRQKEEELAHQAQLEHNTNYANFPPHVLS

HPLSNIGDAYHAEGSGDVEGIPHQHQINSFMPPWMLRHING

>EuMADS66

MKRIANKINRQVTFSKRKGGLMKKANEISVLCDAEVALIVFSHKGKLFQYSSTHSCMEGILDRYERYSFSGREHVVNETKLSGSWTMEYTKMKAKMELLQ

ENIRRRQWKIKTIEENEKTKANLQEQQNHGLNSSSFYMAQPLLPNLDIGGIYQEGGGRRNHELDLTREPLFLTNSCQLGFFPSH

#nucleotide sequences of MADS-box genes in Arabidopsis thaliana

>AT1G01530.1 | Symbols: AGL28 | AGAMOUS-like 28 | chr1:192640-193662 REVERSE LENGTH=744

ATGGCGAGAAAGAATCTTGGTCGTAGAAAAATAGAGTTGGTAAAAATGACCAACGAATCAAACCTTCAAGTTACGTTTTCAAAAAGAAGATCTGGTCTTT

TCAAAAAAGGCAGTGAACTTTGCACCTTATGTGACGCGGAAATTGCGATAATCGTGTTTTCACCCTCGGGAAAAGCATACTCTTTTGGTCATCCAAATGT

TAACAAGTTACTTGACCACTCTTTAGGGCGTGTTATAAGACACAACAACACAAACTTTGCTGAAAGCCGCACAAAGCTCCGTATTCAAATGCTCAATGAG

TCTTTAACTGAGGTGATGGCTGAAAAAGAAAAAGAACAAGAGACCAAGCAGTCAATAGTCCAAAATGAAAGAGAGAACAAGGACGCTGAGAAGTGGTGGA

GGAACTCTCCAACAGAACTCAACTTAGCTCAATCAACTTCCATGAAATGTGATCTTGAAGCTTTGAAGAAGGAAGTTGATGAAAAAGTCGCTCAACTTCA

TCATAGAAACCTAAACTTCTATGTTGGAAGTTCTAGCAATGTTGCTGCTCCAGCAGCTGTTAGTGGTGGTAATATCTCCACAAACCATGGTTTCTTTGAT

CAAAACGGAAACTCTACTTCTGCTCCAACACTGCCGTTTGGATTTAATGTTATGAATCGCACACCAGCTGGGTACAACAGTTACCAACTCCAAAACCAGG

AGGTTAAACAAGTCCATCCTCAGTATTGGGCTCGTTACTATTAG

>AT1G17310.1 | Symbols: AGL100 | agamous-like 100 | chr1:5928014-5928667 REVERSE LENGTH=654

ATGAAAGATTTGTTCATGGAAGGAGAAAGAGAAACTTCATCGATGACTTGCTTGACGCCCAAGGATTCTGTTCAAAGCCCTAACATGTTGGTTAGACAAC

CAAAAAAGGAGACAACAACACAAACCCCTAAAACGACACGAGGAAGACAGAAGATAGAGATCAAGAAAATCGAGGAAGAGACCAAGAGACAAGTAACGTT

CTCGAAACGCCGTCGTGGGCTGTTCAAGAAATCCGCTGAATTGAGCGTTCTCACGGGCGCAAAAATCGCCGTCATAACGTTCTCTAAATGCGATAGGATC

TACAGATTTGGCCATGTAGACGCATTGATAGACAAGTATCTTCGTAAGAGTCCGGTGAAGTTGGAGGGATATTCCGGTGATAACGCGGCGGATGAGGAAA

GTAGGAGACCGTGGTGGGAGCGTCCGGTGGAGAGTGTGCCAGAAGAGGAGCTTGAGGAGTACATGGCAGCATTGAGTATGTTGAGAGAAAATATAGGGAA

AAAGATTGTGGCGATGGGTAACGATCGGACGGTTGATATGGTTCCGGCATGGCCAATCAATGTGATGGGATGGAAACCAACGATGGATATGCAAAAATTG

GAAAATCTGACGGATGGGGTTAATCGATGCCGTGTAGGTCAAAACGGTGATTGA

>AT1G18750.1 | Symbols: AGL65 | AGAMOUS-like 65 | chr1:6467266-6469640 FORWARD LENGTH=1170

ATGGGAAGGGTTAAGTTGAAGATTAAAAGACTTGAGAGCACAAGCAACAGGCAAGTTACATACACGAAGAGAAAAAATGGGATTTTGAAGAAAGCCAAAG

AGTTATCGATTTTGTGTGATATTGATATTGTCCTTCTTATGTTTTCCCCTACCGGAAGAGCTACTGCTTTCCATGGAGAACACAGTTGCATTGAAGAGGT

TATTTCCAAGTTTGCGCAATTAACTCCACAAGAAAGGACAAAAAGGAAACTGGAGAGCCTTGAAGCATTGAAGAAAACTTTTAAGAAACTGGATCATGAT

GTAAATATACATGACTTTTTAGGAGCAAGGAATCAAACTATTGAGGGTCTAAGTAACCAAGTAGCCATTTACCAAGCTCAGCTAATGGAGTGTCATAGGA

GGTTGAGTTGTTGGACGAACATCGATAGAATAGAAAACACTGAGCACCTCGATTTATTGGAAGAATCATTGAGGAAATCCATTGAAAGAATCCAGATTCA

CAAGGAACATTACAGAAAGAACCAACTCTTGCCAATAGAATGTGCAACAACACAGTTTCACAGCGGGATACAGTTGCCTATGGCGATGGGAGGTAATAGT

AGTATGCAAGAAGCTCACTCCATGTCTTGGCTTCCTGATAATGATCACCAGCAAACAATCTTACCTGGTGATTCCAGTTTTCTTCCCCATAGAGAGATGG

ATGGTTCGATTCCCGTTTACTCAAGCTGCTTCTTTGAGTCTACGAAACCAGAAGATCAGATATGCAGCAACCCGGGACAACAGTTTGAGCAGTTAGAACA

ACAAGGAAACGGTTGTTTGGGGTTACAACAACTTGGAGAGGAATATTCATATCCTACACCGTTTGGTACTACTTTGGGAATGGAAGAAGATCAAGAGAAA

AAGATAAAATCTGAAATGGAATTGAACAACTTGCAACAACAGCAACAGCAACAACAACAACAACAACAACAAGATCCTTCAATGTATGATCCCATGGCTA

ATAATAATGGTGGCTGCTTTCAGATTCCTCATGATCAGTCCATGTTTGTCAATGATCATCATCATCATCATCACCACCATCATCAAAATTGGGTTCCAGA

TTCAATGTTTGGTCAGACTTCTTACAACCAGGTTTGTGTGTTCACACCTCCATTGGAACTATCTAGGTAG

>AT1G22130.1 | Symbols: AGL104 | AGAMOUS-like 104 | chr1:7812387-7814259 REVERSE LENGTH=1008

ATGGGTCGGGTGAAATTAGAAATCAAGAGAATAGAGAACACAACGAATCGACAAGTTACGTTTTCAAAACGTAGAAATGGTTTGATTAAGAAAGCTTATG

AATTGTCGATTCTTTGTGATATCGACATTGCTCTTATCATGTTCTCTCCTTCTGATCGCCTTAGCCTTTTCTCCGGAAAAACTCGGATTGAAGACGTTTT

TTCAAGATTCATCAATCTTCCTAAACAAGAACGAGAGAGTGCTCTATACTTTCCTGACCAGAATAGACGCCCAGATATTCAAAACAAAGAGTGTCTACTA

AGGATTCTGCAGCAACTCAAGACTGAAAATGACATCGCTCTTCAAGTCACGAACCCTGCAGCTATCAACTCTGACGTCGAGGAACTTGAGCATGAAGTTT

GTAGGTTACAACAACAACTCCAAATGGCAGAAGAAGAACTAAGGAGATACGAACCGGATCCAATAAGATTCACGACCATGGAGGAATACGAAGTTTCTGA

GAAGCAACTTCTCGACACTCTAACACATGTTGTCCAACGGCGAGACCATCTCATGAGCAACCATTTATCTTCGTATGAAGCATCTACTATGCAACCAAAC

ATTGGTGGTCCTTTTGTAAATGACGTCGTTGAAGGTTGGCTACCTGAAAATGGGACCAATCAAACCCATTTGTTTGATGCATCGGCCCATTCTAACCAAC

TCAGAGAATTATCATCGGCAATGTATGAACCATTGTTGCAAGGAAGTAGCTCAAGCTCGAACCAAAACAACATGAGTGAATGCCACGTGACGAACCACAA

TGGTGAAATGTTCCCTGAGTGGGCTCAGGCGTATTCATCCTCGGCCTTATTCGCTTCTATGCAGCAACAGCATGAAGGTGTGGGTCCCAGTATAGAAGAG

ATGATGCCAGCTCAGCAGAGTGATATTCCGGGAGTGACGGCGGAGACACAAGTTGATCATGAAGTCTCCGACTATGAAACAAAGGTTCCTCAACTCAGTA

GCCAATAA

>AT1G22590.2 | Symbols: AGL87 | AGAMOUS-like 87 | chr1:7983511-7984002 FORWARD LENGTH=492

ATGGGAAGAAGAAAAGTTACACACCAATTGATTTCTGACAACGCCACTCGCCGAGTTACATTCAGAAAACGCAAAGATGGTTTATTGAAGAAGATCTATG

AACTGACCGTTCTATGTGGTTTACCCGCTTGTGCCATCATCTACAGCGAATACAAAGATGGCCCTGAGCTCTGGCCAAACCTTAACGAGGTTCGTTCTAT

TCTTAACAGGTTAAGCGAACTTCCGGTAGAGAAGCAGACCAAATACATGATGGACCAGAAGGATCTCATGAACAAGATGATCCAAGACGCAGAGAAGAAG

TTGGAGAAGGAGAAGATGCATACTCGTGCGATGAAACTTGGGTTAATGGCTGGGTCTAATGATCTAATCACCGATACTGATTGTTCGGAGGAACTGGCCA

GAGCAGCTGATGTGGTTGATAAGAAGCTCAAAGCCATCAGAGAGAGAATCAAGGCTGTTGAAGCAGGAGCACCAATCATCAAGAGAGACTAG

>AT1G24260.1 | Symbols: AGL9, SEP3 | SEPALLATA3, AGAMOUS-like 9 | chr1:8593790-8595862 REVERSE LENGTH=753

ATGGGAAGAGGGAGAGTAGAATTGAAGAGGATAGAGAACAAGATCAATAGGCAAGTGACGTTTGCAAAGAGAAGGAATGGTCTTTTGAAGAAAGCATACG

AGCTTTCAGTTCTATGTGATGCAGAAGTTGCTCTCATCATCTTCTCAAATAGAGGAAAGCTGTACGAGTTTTGCAGTAGTTCGAGCATGCTTCGGACACT

GGAGAGGTACCAAAAGTGTAACTATGGAGCACCAGAACCCAATGTGCCTTCAAGAGAGGCCTTAGCAGAACTTAGTAGCCAGCAGGAGTATCTCAAGCTT

AAGGAGCGTTATGACGCCTTACAGAGAACCCAAAGGAATCTGTTGGGAGAAGATCTTGGACCTCTAAGTACAAAGGAGCTTGAGTCACTTGAGAGACAGC

TTGATTCTTCCTTGAAGCAGATCAGAGCTCTCAGGACACAGTTTATGCTTGACCAGCTCAACGATCTTCAGAGTAAGGAACGCATGCTGACTGAGACAAA

TAAAACTCTAAGACTAAGGTTAGCTGATGGGTATCAGATGCCACTCCAGCTGAACCCTAACCAAGAAGAGGTTGATCACTACGGTCGTCATCATCATCAA

CAACAACAACACTCCCAAGCTTTCTTCCAGCCTTTGGAATGTGAACCCATTCTTCAGATCGGGTATCAGGGGCAACAAGATGGAATGGGAGCAGGACCAA

GTGTGAATAATTACATGTTGGGTTGGTTACCTTATGACACCAACTCTATTTGA

>AT1G26310.1 | Symbols: CAL1, AGL10, CAL | AGAMOUS-like 10, CAULIFLOWER | chr1:9100330-9103510 REVERSE LENGTH=768

ATGGGAAGGGGTAGGGTTGAATTGAAGAGGATAGAGAACAAGATCAATAGACAAGTGACATTCTCGAAAAGAAGAACTGGTCTTTTGAAGAAAGCTCAGG

AGATCTCTGTTCTTTGTGATGCCGAGGTTTCCCTTATTGTCTTCTCCCATAAGGGCAAATTGTTCGAGTACTCCTCTGAATCTTGCATGGAGAAGGTACT

AGAACGCTACGAGAGGTATTCTTACGCCGAGAGACAGCTGATTGCACCTGACTCTCACGTTAATGCACAGACGAACTGGTCAATGGAGTATAGCAGGCTT

AAGGCCAAGATTGAGCTTTTGGAGAGAAACCAAAGGCATTATCTGGGAGAAGAGTTGGAACCAATGAGCCTCAAGGATCTCCAAAATCTGGAGCAGCAGC

TTGAGACTGCTCTTAAGCACATTCGCTCCAGAAAAAATCAACTCATGAATGAGTCCCTCAACCACCTCCAAAGAAAGGAGAAGGAGATACAGGAGGAAAA

CAGCATGCTTACCAAACAGATAAAGGAGAGGGAAAACATCCTAAGGACAAAACAAACCCAATGTGAGCAGCTGAACCGCAGCGTCGACGATGTACCACAG

CCACAACCATTTCAACACCCCCATCTTTACATGATCGCTCATCAGACTTCTCCTTTCCTAAATATGGGTGGTTTGTACCAAGAAGAAGACCAAACGGCGA

TGAGGAGGAACAATCTGGATCTGACTCTTGAACCCATTTACAATTACCTTGGCTGTTACGCCGCTTGA

>AT1G28450.1 | Symbols: AGL58 | AGAMOUS-like 58 | chr1:10003966-10004523 FORWARD LENGTH=558

ATGAATCCCAAGAAAACCAAAGGAAAACAAAAGATTAACATCAAGAAAATTGAAAAAGACGAAGACAGATCGGTCACATTGTCTAAGCGTCTAAATGCTA

TCTACACTATGATCATTGAGCTTTCCATTCTCTGTGGTGTTGAAGTTGCGTTTATCGGGTATTCTTGCTCTGGAAAACCATACACATTCGGCAGTCCGTC

CTTCCAAGCCGTGGTGGAGCGGTTTCTCAACGGCGAGGCCTCGTCGTCTTCTTCATCATCGTTGCAACGATCGGTCAAGAATGCTCACAAGCAAGCGAAG

ATTCAAGAGCTTTGCAAAAGATACAATAGATTGGTGGAGGAGTTAAAGGTGGACGAAGTCAAAGTCAAGAAGGCAGCTGCATTGGCGGAGACGAGGGCCG

TGAATAAGGATGCGTGGTGGAAAGCAGATCCAAATGACGTGAAGGATCACGAAAAGGCGAAGAAGATGATGGAGAAGTATCAAGAGCTCAAAGAGAAACT

GCGTGAGGAAGTTGCTTTAAGGATCAAGAGAGGACATGATGAGAATAATAACAAATGA

>AT1G28460.1 | Symbols: AGL59 | AGAMOUS-like 59 | chr1:10006230-10006778 FORWARD LENGTH=549

ATGAATCCCAAGAAAACCAAAGGAAAACAAAAGATTAACATCAAGAAAATTGAAAAAGATGAAGGCAGATCGGTCACATTCTCTAAGCGTCTAAATGGTA

TCTACACTAAAATCAGTGAGCTTTCTATTCTCTGTGGTGTTGAAGTTGCGTTTATCGGGTATTCTTGCTCTGGAAAACCATACACTTTTGGCAGTCCATC

CTTCCAAGCCGTGGCGGAGCGGTTTCTCAACGGCGATGCCTCGTCGTCTTCTTCATCATCATTGGTCATGAATGCTCACAAGCAAGCGAAGATTCAAGAG

CTTTGCAAAAAATACAATAGATTGGTGGAGGAGTTAAAGGTGGACGAAGTCAAAGTCAAGAAGGCAGCTGCATTGGCGGAGACGAGGGTCGTGAATAAGG

ATGTGTGGTGGAAAGTGGATCCAAATGACGTGAAGGATCACGAAAAGGCGAAGAAGATGATGGAGAAGTATCAAGAGCTCTATGATAAACTGTGTGAGCA

AGCTGCTTCAAGGATCAAGAGAGGACATGATGAGAATAATAACAAATGA

>AT1G29962.1 | Symbols: AGL64 | AGAMOUS-like 64 | chr1:10496730-10497287 FORWARD LENGTH=558

ATGAAACCCAAGAAAACCAAAGGAAAACAAAGGATTAACATCAAGAAAATTGAAAAAGACGAAGACAGATTGGTCACATTGTCTAAGCGTCGAAATGGTA

TCTACACTAAACTCAGTGAGCTTTCCATTCTCTGTGGTGCTGAAGTTGCGTTTCTCGGGTATTCTTGCTCTGGAAAACCATACACATTCGGCAGTCCGTC

CTTCCAAGCCGTGGCAGAGCGGTTTCTCAACGGCGAGGCCTCGTCGTCTTCTTCATCATCGTTGCAACGATCGGTCATGAATGCTCATCAGCAAGCGAAG

ATTCAAGAGCTTTGCAAAGTATACAATAGATTGGTGGAGGAGATAACGGTGGAAGAAGTCAAATTAAAGAAGACGGCTGCATTGGCGGAGATGATGCCCA

TGAATGAGGATGCGTGGTGGAAAGTGGATCCAAATGACGTGAAGGATCGCGAAGAGGTGAAGAAGATGATGGAGAAGCATCAAGAGCTCTATGAGAAACT

GTGCGAGGAAGCTGCTTCAAGGATCAAGAGAGGACATGATGAGAATAATAACAAATGA

>AT1G31140.1 | Symbols: GOA, AGL63 | AGAMOUS-like 63, GORDITA | chr1:11118031-11119673 FORWARD LENGTH=642

ATGAGGAAAGGTAAGAGAGTGATAAAAAAGATAGAGGAGAAAATAAAGAGACAAGTGACATTCGCAAAGAGAAAGAAGAGTCTAATCAAGAAGGCATATG

AACTCTCTGTTCTCTGCGATGTCCACCTTGGTCTCATCATCTTCTCTCACTCCAACAGGCTCTACGATTTCTGCTCCAACTCTACCAGCATGGAGAATCT

CATCATGAGATACCAAAAGGAAAAAGAAGGTCAAACCACTGCAGAACACAGTTTCCACTCGTGTTCAGATTGCGTGAAGACGAAGGAATCAATGATGAGA

GAGATAGAGAATCTTAAGCTGAATCTTCAATTGTACGACGGACATGGCTTGAATCTCTTGACCTACGACGAGCTCCTTTCTTTTGAGCTCCATCTCGAAT

CTTCTCTACAACATGCTCGAGCTCGCAAGTCTGAGTTCATGCATCAGCAGCAGCAGCAACAAACAGATCAAAAGCTTAAGGGAAAAGAAAAGGGTCAAGG

AAGCTCTTGGGAGCAGCTGATGTGGCAAGCAGAGAGACAGATGATGACGTGTCAAAGACAAAAAGATCCTGCGCCGGCGAATGAAGGAGGAGTTCCTTTT

TTACGGTGGGGAACAACCCACCGACGTTCTTCACCTCCTTAA

>AT1G31630.1 | Symbols: AGL86 | AGAMOUS-like 86 | chr1:11318528-11319547 REVERSE LENGTH=1020

ATGAGGTCGAAAATTAAGTTATCACTCATAGCTAATAAGACCTCAAGGAGAACCACATTCAGGAAGAGGAAGGGAGGGATAACGAACAAACTCCATGAGC

TAACAACTCTCTGCGGCGTCAAAGCATGTGCGGTAATCTCCAGTCCGTACGAGAATCCAGTGGTGTGGCCGTCAACCGAAGGTGTTCAAGAGGCGGTTTC

CATGTTTATGGAGAGGCCGGCGACAGAACAATCCAAGCTGATGATGAGTCATGAGACCTACTTGCAGGACAAAATTACCAAAGAAACAAAGAAACTGGAG

AGTCTACGTCGTGAAAACCGAGAATCTCAGCTTAGGCAATTTATGTTTGATTGTGTTGAAGGTAAGATGAGTGAGCATCAGTATGGTGCAAGGGACCTTC

AAGATTTAAGTCTTTATATTGATCACTATATCAATCAGCTTAATTCAAGTGTCATGCTCCTTACAAACAATGGTGCGTCTTCTTCTTCCTTTCCTCCTCC

GCTTCATACTTCAGTTGCGGGTGCGGGTGCGGGTGCGGGTGCTGCTCCTCTTGTTGTTGCGGGTGCGGGTGCGGCTCCTCTTGCTGTTGCGGGGGCGGGT

GCGAGTCCTCTTGCTGTTGCGGGTGTGGGTGCGGCTCCTCTTGCTGTTGCGGGTGCCGGTCCTCCTATGGCTCAGAATCAGTATGAGCCGATTCAGCCCT

ATATCCCTACTGCTTTTAGTGATAATATTCAATACCAAGCTCCTGTGGATTTTAATCATCAGATCCAACATGGAATCTATGATAATCTCAGTTTGGATCC

AAATCATCAGTATCCGTTTCAAGATGATCCATTCATGGAGATGTTGATGGAATATCCTTATGAACAAGTGGGTTATGCTGCAGAGCATGCACACATCCCT

TTCATGAACGGAAACTACTACAACTACCACCAACCACCAACCGTTGGTCTTACTACCACCGGTCACATGCCTTCCAACAACGCCACCACCACCACCACCA

CCAACACCACCGTTGTGTGA

>AT1G31640.1 | Symbols: AGL92 | AGAMOUS-like 92 | chr1:11322692-11324176 REVERSE LENGTH=1395

ATGAGGACGAAGACTAAGTTAGTACTCATACCTGATAGACACTTTCGGAGAGCCACATTCAGGAAGAGGAATGCAGGGATAAGGAAGAAACTCCACGAGC

TGACAACTCTCTGTGACATCAAAGCATGTGCGGTAATCTACAGTCCGTTCGAGAATCCAACGGTGTGGCCGTCAACCGAAGGTGTTCAAGAGGTGATTTC

GGAGTTCATGGAGAAGCCGGCGACAGAACGGTCCAAGACGATGATGAGTCATGAGACTTTCTTGCGGGACCAAATCACCAAAGAACAAAACAAACTAGAG

AGTCTACGTCGTGAAAACCGAGAAACTCAGCTTAAGCATTTTATGTTTGATTGCGTTGGAGGCAAGATGAGTGAGCAACAGTATGGTGCAAGGGACCTTC

AAGATTTAAGTCTTTTTACTGATCAATATCTTAATCAGCTTAATGCCAGGAAGAAGTTCCTTACAGAATATGGTGAGTCTTCTTCTTCTGTTCCTCCTCT

GTTTGATGTTGCGGGTGCCAATCCTCCTGTTGTTGCAGATCAAGCTGCGGTAACTGTTCCTCCTTTGTTTGCTGTTGCGGGTGCCAATCTTCCTGTTGTT

GCTGATCAAGCTGCGGTAACTGTTCCTCCTCTGTTTGCTGTTGCGGGTGCCAATCTTCCTGTTGTTGCAGATCAAGCTGCGGTTAATGTTCCTACTGGAT

TTCATAACATGAATGTGAACCAGAATCAGTATGAGCCGGTTCAGCCCTATGTCCCTACTGGTTTTAGTGATCATATTCAATATCAGAATATGAACTTCAA

TCAAAACCAACAAGAGCCGGTTCATTACCAGGCTCTTGCTGTTGCGGGTGCCGGTCTTCCTATGACTCAGAATCAGTATGAGCCCGTTCACTACCAGAGT

CTTGCTGTCGCGGGTGGCGGTCTTCCTATGAGTCAGTTGCAGTATGAGCCGGTTCAGCCTTATATCCCTACTGTTTTTAGTGATAATGTTCAATATCAGC

ATATGAATTTGTATCAAAATCAACAAGAGCCGGTTCACTACCAAGCTCTTGGTGTTGCAGGTGCCGGTCTTCCTATGAATCAGAATCAGTATGAGCCGGT

TCAGCCCTATGTCCCTACTGGTTTTAGTGATCATTTTCAGTTTGAGAATATGAATTTGAATCAAAATCAACAGGAGCCGGTTCAATACCAAGCTCCTGTT

GATTTTAATCATCAGATTCAACAAGGAAACTATGATATGAATTTGAACCAGAATATGAAGCATGCACACATCCCTTTCATGGACGGAAACTACTACAACT

ACCACCAGCCACCAACCGTTGGTCTTACTTCCACCGGTCACATGCCTTCCACCACCACCACCACCACCAACAACAACAACAACAACAATGTGTGA

>AT1G33070.1 | Symbols: no symbol available | no full name available | chr1:11982889-11985299 FORWARD LENGTH=498

ATGCCCATGAGGAAGGAAGGGATAACGAAGAAACTTTATGAGTTGGCAACTCTCTGCGACATCAAAGCATGTGCGGGTAAGATAAGTGAGCATCAGTATG

GTGCAAGGGACCTTCAAGATTTAAGTTTTCATATTGATCACTATATCAATCAGCTTAATTCCAGGGTCAAGATCCTTACAAACAATGGTGAGTCTTCTTC

TTCCGTTCCTCCTCTACTTCATACTTCAGTTGCGGGTGCGGGTGCAGCTCCTCTTCCTGTTGCGGGTGCCGATCTTCCGATGGATCAGAATCAGTATGAG

CCGATTCAGCTCTATATCCCTATTGGTCTTAGTTATCATATTCAATACCAACATGAAATCTATGATAATTTCAAGCATGCGCACATCCTTTTCATGAACG

GAAACTACTACAACTACCACCAAACACCAACCGTTGGTCTTACTACCACCGGTCACATGCCTTCCAACAACAACACCACCCCCACCACCGATGTGTGA

>AT1G46408.1 | Symbols: AGL97 | AGAMOUS-like 97 | chr1:17232135-17232935 REVERSE LENGTH=801

ATGGGTGGCGTGAAGAGGAAGATTGCTATAGAGAAGATACAGAACAAAAATCCACGAGCGGTTTCATTCTCCAAACGTCGTAAGGGTCTGTATAGCAAAG

CTTCTGAACTTTGTCTTCTCTCGGACGCAGAGATTGCGATCATAGCGACTCCTGTTTCTTCTAATTCCAACGCCGCTTTCTACAGTTTTGGCCACTCCTC

TGTTGATAATGTCGTCGCTGCTTTTCTCGCCAATCAGCGTCCTTGTGATGAAAGGTTTTGGTGGGAAGATGAGAGTCTTCTAAAATCAGAGAATCTGGAG

GAGTTGAGAGAGGCGATGGATTCAATGTCGACTATGTTGCGAGATCTCAAGGAGTTGGAGAAGCAAAGAGATCATCAAACGCAAACCCTAATTCATCAGC

CGTGTTCTGCAAGGGTTTGCATTCAAGATTATGTAACTGTGAATTTCGATGGGTTTAACACGGAAGAGCAAACCCTAGCGGTTTCTGACAATAGTAACAA

CAACGGTTTACTTGGAAACTTGGATGAGTGCAATGAAGATTTTGATGATCTCGATCAAATCTTCGATACGGTGACAAACTCTGAATTTTTATCGGTGAAT

TTGGAGATGGATGATGTAACTGTGAATTCTGAAGGGAACACGGAAGAGCAAACCCTAGCGGTTTCTGACAATAGCAACAACAACGGTTTACTTGGAAACT

TGGATGAGTGCAATGAAGATTTTGATGATCTCGATCAAATCATTGAGTATTTGACAAGCTCTGAAGCTTTATCCATGAATTTAAAGATGGATGATGTCTG

A

>AT1G47760.1 | Symbols: AGL102 | AGAMOUS-like 102 | chr1:17572451-17573159 FORWARD LENGTH=555

ATGGGTCGTAGAAAAATAGAGATAAAATTTATTGAAGATAGCATCGAAAGGAAAGCGACGTTTTCAAGACGCCGAAATGGCATTTTTAAGAAGGCTGATG

AACTCGCGAAGTTGTGTAATGTAGAGATTGCTGTCTTGGTCATCTCCCCCACTAATATACCATACACATACGGTTATCCATGCTTCAACGATGTGGTTGA

GCGTATTCAAAATCCTAGTGCTTCGTCCAAACTCAGGAGTCTCATGAAAGAATTGGAACAAATCAAAGAGTTTCAGGAGGATTTGAGGAAGAAACAGCAA

AGAAACCTTGAAAAGTCCAATATGAAAGAGAATGTAGATCTGAAATTGGAGGACTTAGTTGCTTTCAAGGCGAAACTCGAGGCCTATCAAGCAGGTTTAA

AGAGGAAACATGTAGAGATGGAGGATTTGTCTTCTCCATCGATACTCTCAAAGAATACAAAGAATAAGATGATGAGGACCGAGTATTCGTCTGGACAGAG

CAAAGGAATGTATGAGTTTCGGGCTTTCGGCCCAGGATTTCTTGGAACTATTTAG

>AT1G48150.1 | Symbols: AGL74 | agamous-like 74 | chr1:17785397-17786368 FORWARD LENGTH=972

ATGCAGTCGTCGAACGTAACTGATTGCACAATGAGGAAAAGAGGAACAAAAAGAAAGATTGAAATTGAGAAACGCATGACTAAACAACAACGATCCGTCG

CTTGCAGCAAACGCCGTCCCACACTCTTCTCCAAAGCGGCTGATCTTTGCCTTCTCTCCGGAGCCAACATAGCCGTTTTTGTAACTTCTCCTGACGAGAA

CTCCGATGTCGTTTATTCATTCTCGGGCTACTCTCATGCCTCTGAAATTGTTGACTGTTACCTCAACAACAAGTCTCCTCCCAAGACTACTATTAACCCT

GAATCAGCCAAGTTTTGGTGGGAAGAGCCCGATCTTTACCGTGATTGTGACGATCTGTCTGAGTTAAGAATCATCGAAGATCGTTTGATGAGAACGAAGA

AGCATCTCATGGATTATCTTGAGAAGAAAGAAAAATCACACTCTGTTTCTAAATCCGATCAAAACCCTAACAACGACAGTGGGTCTTCTTCTTCGTCTTC

TCAAATTGCTTCTGATTTTGGTCAAAACCCTAGTACCTTGTCTCCGTCTTCCTTAAAAATTGTTTCTTTTGATCAGAACTCTTACTCCTCACTTGAGCCT

TCGTCACAAGTTACTACTTGTTTTGATCAAAACCCTGTTTTCTCTGTTGGCAGCGAGTCATCATCTGATCAATCTCGTTACTTGGTGAATGAAGATTCTG

GATTTGTTGACGGCCTCTTATGTGAAACAGAGGAAGAGAATAACGGAATGCGTCTTCCCCAAGAAACACAAACACAACCGATGTTCACAGAGGAGGATCA

GAGCTTTTGGGAAAACCTAGATGTCGACGACGTGTTTGGTCTTTTTAAGGACGACAACAATCTTGAGGTTCCTCTTCAAGATCATTCCTCAACCAACGAA

GACGACGAGTTATTGATCGACATAAGTGAATACTTAAGCGAAGAAGCAATGGAATGCCCATGTTTTAGTTAG

>AT1G54760.1 | Symbols: AGL85 | AGAMOUS-like 85 | chr1:20433912-20434397 FORWARD LENGTH=486

ATGAAGACAGATTGGTCACATTATCTAAGCGTCGAAATGGAATCTACAATAAGCAATGAGCTTTCCATTCTCTGTGGTGCTGAAGTTGCGTTTCTCGGAT

ACTCTTGCTCAGGAAAACCATACACATTCGGCAGTCCGTCTTTTCAAGCCGTGGCAGAGCGATTTCTCAATCGTGAGGCCTCATCATCGTTGCAACGATC

GGTCATGAATGCTCACCAACAAGCGAAGATTCAAGAGCTTTGCAAAGTATACAATAGAATGGTGGAGGAGGCAAAGACGGAAGAAGCCAAAGTAAAGAAG

GCAGCTGCATTAGCGGAGACAATGCCCGTAGATGAGGATGCGTGGTGGAAAGTGGATCCAAAGGAGGTGGAAGATCACGAAGAGGCGAAGAAGATAATGG

AAAAGTGTGAAGGGCTCTATGAGAAACTGTGTAATGAAGCTGCTGCAAGGATCCAAAGAGGAGATGCTGAGAATAATAACAAATGA

>AT1G59810.1 | Symbols: AGL50 | AGAMOUS-like 50 | chr1:22008604-22009455 FORWARD LENGTH=852

ATGGCTCCTCGTCAGAAGAAACCTAACAAGTCTGATGATGATGATGATTTGCGTAGGAAGAAACAGAGCTTTTTCAAACAGAGATTTCCAGGCTTTAAGA

AGAAAGCCTCTGAGCTCTCTGTTCTCTGCGGCAACTCTGTCGGTTTCATCTGTTACGGTCCCGACAGCGATCTTCATGTTTGGCCTCAGTCTCAAGATCA

TAACCCACAAGCCCTACATGAGATCGTTGCCAAGTTCAATGCCTTGAGTGATGAGAGGAGGAAGAATCATGCATGTGATCTCAATGACTTCCCTCATCAT

CTCAAGGGTTTGTCTCGTGAGGAGTTAAGGAAGCATCTCCTTCACCTTGACTCTCAATTACTTGGAGTCAGGGAACAAAAGATAGAAATTCTTAAGAAGA

CGCTCACGGGTTCTTCCGAAAAAGATGGTGCTAGGGTTTCAGAGAACTCGGCCATCTCCGATCACAAGTTGAAGATAGAGCCTCATTTGAAGGATATATT

GTCGGAAGATCATCTAATTAGGGTTTCAGATAAGAAGCTGGGTTCGTGTGATGTATTTGACGAGTTGGCTTACGTGGTCCGCGGGTCAAGGAATTTGAAC

GAGAATGTTTCCAAGTACGAATCAAAAGATGCAGATAACACGGGACTGGATCATCTTGTAACTTTAGGCGGTGATTATCTTCAGGAGGCGGCAGCAGAAC

TTTACCAAACTTACAATCTAGGGAATTTTTGTGATGATCATGTTTGGGATCTGGAGTTTGCCTCAAGACTACCACTACTTCATACTTTTAGCGATCCTCT

CATGACCACCAATACTTGCCAAACGATGAGCACCGATATGATTTCAATTTGA

>AT1G60040.1 | Symbols: AGL49 | AGAMOUS-like 49 | chr1:22119075-22119929 REVERSE LENGTH=855

ATGGCTCCTCGTCAGAAGAAACCTAACAAGTCCGATGATGATGATGGTGATTTGCATAGGAAGAAACAGAGCTTTTTCAAACAGAGATTTCCAGGCTTTA

AGAAGAAAGCCTCAGAGCTCTCTGTTCTCTGCGGCAACTCTGTCGGTTTCATCTGTTACGGTCCCGACAACGATCTCCATGTTTGGCCTCAGTCTCAAGA

TCATAACCCACAAGCCCTACATGAGATCGTGGCCAAGTTCAATGCCTTGAGTGATGAGAGGAGGAAGAATCATGCATGTGATCTCAATGACTTCCCTCAT

CATCTCAAGGGTTTGTCTCGTGAGGAGTTAAGGAAGCATCTCCTTCACCTTGACTCTCAATTACTTGGAGTCAGGGAACAAAAGATAGAAATTCTTAAGA

AGACGCTCACGGGTTCTTCCGAAAAAGATGGTGCAAGGGTTTCAGAGAACTCGGCCATCTCCGATCACAAGTTGAAGATAGAGCCTAATTTGACGGATAT

ATTGTCAGAAGATCATCTAATTAGGGTTTCAGATAAGAAGCTGGGTTCGTGTGATGTATTTGACGAGTTGGCTTACGTGGTCCGCGGGTCAAGGAATTTG

AACGAGAATGTTTCCAATTACGAATCAAAAGATGCAGCTTACACGGGAATGGATCATCTTGGCACTTTCGGCGGTAATTATCTTCAGGAGGCGGCGGCAG

AACTTTACCAAACTTACAATCTAGGGAATTTTTGTGATGATCATGTTTGGGATCTGGAGTTTGCCTCAAGACTACCACCACTTCATACATTTAGCGATCC

TCTCATGACCACCAATACTTGCCAAACGATGAGCTCCGATATGATTTCAATTTGA

>AT1G60880.1 | Symbols: AGL56 | AGAMOUS-like-56 | chr1:22411575-22412180 FORWARD LENGTH=606

ATGGGAGGCAAAAAAACAAAGATTGAGATTAAGAAGATCATAAACAAACCCGCAAAAACGGTTGCTTTCACAAAACGCAGGGAAGGTCTCTTCCGCAAAG

CTTCACAGCTTTGTCTCCTCTCTCCAGCCACTCAAATCGCAATCTTAGCGGCTCCTATGACTTCCAAATCTCACGCTTCTTTCTACTCTTTCGGTCATTC

CTCTGTCGATAACGTTGTCTCCTCTCTGCTTTATGACCATCCTCCTCTTACGGCAAACCAAGACAACAGGTCAGGGTTAGGGTTTTGGTGGGAAGACAAA

CGTTTTGACGTATCGGAGAACGTTGAGGAGTTAAAAGAGGCGGTCGATGCTGTTTCGAGGATGTTGAACAATGTGAGATGCCGATTAAATGATGCCGTGA

AGAGTACTCAAAGAGATGGAGGTTTAGAGATTCTTCATCATCAGGAGGAGGAAGTTCTTCAGACTCGCAACGACGAGACAAAGACGAATCAAACTCACGA

ATTTGAAGGCGGCGAAACTTCTGGTTCTGCAAGTTGGTTAGAGAACGAGGATGATATTCTTCACTTTGATGATGATTTTTATACTGGTATTGATCCTTTG

TTTTGA

>AT1G60920.1 | Symbols: AGL55 | AGAMOUS-like 55 | chr1:22429692-22430267 REVERSE LENGTH=576

ATGGGAGGCACAAAAAGAAAGATAGAGATGAAGAGAATCGAAGATAAGAACGTGAGAGCAGTTGCTTTCACAAAACGCAAAAGCGGACTCTTCCACAAAG

CTTCAGAGCTCTGTCTCCTCTCTCCGGGGACTCAAATCGCAATCTTAGCAACTCCTCTTTCTTCCCACTCCCATGCTTCTTTCTACTCTTTCGGTCATTC

CTCTGTCGATCACGTTGTCTCCTCTCTCCTTCACAATCAGCATCCATCTCTTCCCACAAACCAAGATAACAGATCAGGGTTAGGGTTTTGGTGGGAAGAC

CAAGCCTTTGACAGATTGGAGAACGTCGACGAGTTGAAAGAGGCAGTCGATGCGGTTTCGAGGATGTTGAACAATGTGAGGCTACGATTAGATGATGCCG

TGAAGAGCAATCAAAGAGATGGAAGTTTAGTGATTCATCAGGAGGATGAGGAGGTTCTTCAGCTTGGCTACAAAGACACAAATCAAATTACCAAACTCGA

AGGTGAAACTTCCGCTTCTGCAAGTTTGTTAAAGAACGTAGTGGATAATCTACACATTGATGATCGTTATTACTGA

>AT1G65300.1 | Symbols: PHE2, AGL38 | PHERES2, AGAMOUS-like 38 | chr1:24254929-24255765 FORWARD LENGTH=837

ATGAAGAGAAAGATGAAGTTATCGTTAATAGAAAACAGTGTATCGAGGAAAACAACATTCACCAAAAGGAAGAAAGGGATGACGAAGAAACTAACCGAGC

TAGTCACTCTATGTGGTGTTGAAGCATGTGCGGTCGTCTATAGTCCGTTCAACTCGATCCCGGAGGCTTGGCCGTCAAGGGAAGGCGTTGAAGACGTGGT

GTCGAAATTTATGGAGTTGTCGGTGTTGGACCGGACCAAGAAGATGGTGGATCAAGAGACTTTTATAAGTCAAAGGATCGCCAAAGAAAAAGAGCAGCTG

CAGAAGCTACGTGATGAGAACCATAATTCTCAGATTCGGGAGTTAATGTTTGGTTGTCTCAAAGGGGAGACGAATGTGTATAATCTTGATGGAAGGGATC

TTCAAGATTTGAGTTTATATATTGATAAGTATCTTAATGGTCTTACTCGCAGGATTGAGATCCTTATTGAGAACGGTGAGTCTTCTTCATCTTTACCTCT

TCCTATTGTTGCGAATGCAGCTGCACCAGTCGGATTTGATGGTCCTATGTTTCAATATCATAATCAAAATCAGCAAAAGCCGGTTCAATTCCAATATCAG

GCTCTTTATGATTTTTATGATCAGATTCCAAAGAAAATTCATGGTTTTAATATGAATATGAATAAGGATTCGAATCAAAGTATGGTTTTGGATTTGAATC

AAAATCTTAATGATGGAGAGGACGAGGGCATTCCTTGCATGGACAACAACAACTACCACCCCGAAATCGATTGTCTCGCTACCGTCACCACTGCCCCCAC

TGATGTTTGTGCTCCTAACATCACCAATGATCTCTAG

>AT1G65330.1 | Symbols: PHE1, AGL37 | AGAMOUS-like 37, PHERES1 | chr1:24266481-24267320 REVERSE LENGTH=840

ATGAGGGGGAAGATGAAGTTATCGTTCATAGAAAATGATTCAGTGAGGAAAACAACATTCACCAAAAGGAAGAAAGGGATGCTGAAGAAATTCAACGAGC

TAGTAACTCTATGTGGTGTTGACGCATGTGCGGTCATCCGTAGCCCGTACAACTCGATCCAGGAGCCTTGGCCATCAAGGGAAGGCGTTGAAGAAGTGAT

GTCGAAGTTTATGGAGTTTTCGGTGTTGGACCGGACCAAGAAGATGGTGGATCAAGAGACGTTTTTACGTCAAAGGATCGCCAAAGAAACAGAACGTCTC

CAGAAGCTACGTGATGAGAACCGTAATTCTCAGATTCGAGATTTAATGTTTGGTTGTCTCAAAGGAGAGGTGGACGTGTCTCATCTTCATGGAAGAGATC

TTCTTGATTTGAATGTATTTCTTAACAAGTATCTCAATGGTGTTATTCGTAGGGTTGAGATCCTTAAGGAGAACGGTGAGTCTTCTTCATCTGTACCTCC

TCCTATTGGTGTAGCTCCTACTGTTGTGGATGCATCTGTCCCAATCGGTTTTGATGGTCGTATGATTCAAGATCAAAACCAAAATCAGCAAGAGCCGGTT

CAATTCCAATACCAGGCTCTTTATGATTTTTATGATCAGATTCCAAAGAAACTTCATGATTTTAACATGAAAATGAATATAGATCCAAATCAGAGTATGA

ATTTGGATCTTAATGATGGAGAGGACGAGGGCATTCCTTGCATGGACAACAACAACTACCACCCCGAAATCGATTGTCTCGCTACCGTCACCACTGCCCC

CACTGATGTTTGTGCTCCTAACATCATCAATGATCTCTAG

>AT1G65360.1 | Symbols: AGL23 | AGAMOUS-like 23 | chr1:24281337-24282151 FORWARD LENGTH=681

ATGGTGAAAAAAACTCTTGGTCGTAGAAAGGTAGAGATAGTGAAAATGACTAAGGAATCAAACCTTCAAGTCACATTTTCCAAGAGAAAAGCTGGTCTTT

TTAAGAAGGCTAGTGAATTTTGCACATTATGTGATGCAAAAATTGCGATGATCGTGTTTTCACCAGCTGGAAAAGTATTTTCTTTTGGTCATCCAAATGT

TGATGTTCTGCTTGACCACTTTCGAGGGTGTGTTGTAGGACACAACAACACAAACCTTGATGAAAGCTACACAAAGCTTCATGTTCAAATGCTCAACAAA

TCCTACACTGAGGTGAAGGCGGAAGTAGAAAAAGAACAAAAGAATAAGCAGTCGCGGGCTCAAAATGAAAGAGAAAACGAAAACGCTGAGGAGTGGTGGA

GTAAGTCTCCATTAGAACTCAACTTAAGTCAATCAACCTGTATGATACGTGTTCTTAAAGATTTGAAGAAGATAGTTGATGAAAAAGCAATTCAATTAAT

CCATCAAACAAACCCAAACTTCTATGTTGGAAGTTCTAGCAATGCTGCTGCTCCAGCAACTGTTAGTGGTGGTAATATCTCCACAAACCAGGGGTTCTTT

GATCAAAACGGAATGACGACTAATCCTACTCAAACACTTCTGTTTGGATTTGATATTATGAATCGCACACCAGGAGTTTAA

>AT1G69120.1 | Symbols: AP1, AtAP1, AGL7 | AGAMOUS-like 7, APETALA1 | chr1:25982576-25986102 REVERSE LENGTH=771

ATGGGAAGGGGTAGGGTTCAATTGAAGAGGATAGAGAACAAGATCAATAGACAAGTGACATTCTCGAAAAGAAGAGCTGGTCTTTTGAAGAAAGCTCATG

AGATCTCTGTTCTCTGTGATGCTGAAGTTGCTCTTGTTGTCTTCTCCCATAAGGGAAAACTCTTCGAATACTCCACTGATTCTTGTATGGAGAAGATACT

TGAACGCTATGAGAGGTACTCTTACGCCGAAAGACAGCTTATTGCACCTGAGTCCGACGTCAATACAAACTGGTCGATGGAGTATAACAGGCTTAAGGCT

AAGATTGAGCTTTTGGAGAGAAACCAGAGGCATTATCTTGGGGAAGACTTGCAAGCAATGAGCCCTAAAGAGCTTCAGAATCTGGAGCAGCAGCTTGACA

CTGCTCTTAAGCACATCCGCACTAGAAAAAACCAACTTATGTACGAGTCCATCAATGAGCTCCAAAAAAAGGAGAAGGCCATACAGGAGCAAAACAGCAT

GCTTTCTAAACAGATCAAGGAGAGGGAAAAAATTCTTAGGGCTCAACAGGAGCAGTGGGATCAGCAGAACCAAGGCCACAATATGCCTCCCCCTCTGCCA

CCGCAGCAGCACCAAATCCAGCATCCTTACATGCTCTCTCATCAGCCATCTCCTTTTCTCAACATGGGTGGTCTGTATCAAGAAGATGATCCTATGGCAA

TGAGGAGGAATGATCTCGAACTGACTCTTGAACCCGTTTACAACTGCAACCTTGGCTGCTTCGCCGCATGA

>AT1G69540.1 | Symbols: AGL94 | AGAMOUS-like 94 | chr1:26145306-26147159 REVERSE LENGTH=1035

ATGGGTAGAGTGAAGCTAAAGATAAAGAAACTACAGAACATGAATGGACGTCAATGTACGTATACGAAAAGGAGACATGGGATTATGAAAAAGGCTAAGG

AGTTATCGATCTTATGCGACATAGATGTTGTGCTTCTCATGTTTTCTCCCATGGGAAAGGCTTCAATTTGCATAGGCAAACACAGCATTGGAGAAGTCAT

TGCTAAGTTTGCTCAACTCTCTCCTCAAGAAAGAGCAAAGAGGAAGTTGGAAAACCTTGAAGCCTTGAGGAAAACTTTCATGAAAGCTAACCACGATATA

GATATATCAAAGTTTCTAGACAGAATTAGTACACCAACAGTTGAGGTGCTTAGCGAAAAAATCAGGTTTCTGCAAACACAATTATCAGATATACACACAC

GACTAAGCTACTGGACCGATGTAGATAATATCGACAGCGTAGATGTTTTGCAGCAACTAGAACATTCACTAAGACAATCTCTGGCTCAAATCTATGGTCG

TAAGGCGAGCATGCCGCAACGTCAGCAGCAACAACTTATGTCCTCACAATGCAAAAACCAGTTGCAGACTGAAATAGATATAGATTTCGGAATGGAGATG

GAGCAACAACTTGAGAATTTCTCATGGGTTCGTACCGATGAAAACATGAATGTTCCTATAGAAGAAGAAGACCCTAATCTGCAGCTTCATCACATGTACA

AGGACATAACGTGTTCTGCAAGTTCAGCTCTTGGAAATTACTCAGGACTCTTTAGTAAAAGCTCAGATATCTTACAAAAACTAGAAACCGGTAGCATTCC

CGGGACATCGGCTGATCCGAACCAACAATTCAGCAATCTCAGTTTCTTAAATGATCAAAAGCTTAAGCAACTAGCTGAGTGGAATCTATTAGGCAGTCCT

GCGGATTACTACGTTAGCCAGATCTTGGAAGCTTCTTATAAGCCTCAGATTGGAGGAAAAAACAACGGCGCTTCTTCTGAAACATTACCTTATGTTGCGG

TCTTCGATGATCCTCTATATTTTTGGCCAAACTGA

>AT1G71692.1 | Symbols: XAL1, AGL12 | XAANTAL1, AGAMOUS-like 12 | chr1:26952903-26954939 REVERSE LENGTH=636

ATGGCTCGTGGAAAGATTCAGCTTAAGAGGATTGAGAACCCGGTTCACAGACAAGTGACTTTTTGCAAGAGGAGAACTGGTCTTCTCAAGAAGGCTAAGG

AGCTCTCTGTGCTCTGTGATGCCGAGATCGGTGTTGTGATCTTCTCTCCTCAGGGCAAGCTCTTTGAGCTCGCTACTAAAGGAACAATGGAGGGAATGAT

TGATAAGTACATGAAGTGTACTGGTGGTGGTCGTGGTTCTTCTTCTGCTACTTTTACTGCTCAAGAACAACTTCAACCACCAAATCTTGATCCGAAAGAT

GAGATCAACGTGCTTAAGCAAGAGATTGAGATGCTTCAGAAAGGGATAAGCTATATGTTTGGAGGAGGAGATGGGGCTATGAATCTTGAAGAACTTCTTT

TGCTTGAGAAGCATCTTGAGTATTGGATTTCTCAGATTCGCTCTGCTAAGATGGATGTTATGCTTCAAGAAATTCAGTCATTGAGGAACAAGGAAGGAGT

CCTCAAAAACACCAACAAGTATCTCCTCGAAAAGATAGAGGAAAACAACAATAGCATATTAGATGCTAACTTCGCAGTCATGGAGACAAACTATTCCTAT

CCGCTAACAATGCCAAGTGAAATATTTCAGTTCTAG

>AT1G72350.1 | Symbols: AGL60 | agamous-like 60 | chr1:27239273-27239947 REVERSE LENGTH=675

ATGGAGGATGGGGAAGCTTCAACAATCACTTTCTTACCAACCACGGAACCAAAACCCCTACAAAACCCTAACTTGCTGGCCAAACCAAAAAAAGAGACTA

AACAAAAAAAACCTAAAACCACCAAAGGTCGACAGAAGATAGAGATCAAGGAGATCATGCTGGAGACCCGAAGGCAAGTGACGTTTTCCAAACGACGATC

CGGGCTTTTCAAAAAAGCGGCAGAATTAAGCGTTCTCTGCGGCGCACAGATTGGTATCATAACGTTTTCACGTTGCGATAGGATCTACTCGTTTGGTAAC

GTGAACTCACTCATCGATAAATACTTGCGTAAGGCTCCGGTGATGCTGAGGTCACATCCCGGTGGTAACGTGGCAAACGGAGAGGAAGATAACGACGGTT

TGATGTGGTGGGAGAGAGCGGTGGAGAGTGTGCCGGAGGAGCATATGGAAGAGTACAAGAATGCCTTGAGTGTGTTAAGGGAGAATTTGTTGACGAGGAT

CTACCAGATGAGTGGTGATCGGACGGTTGAGAATCTTCCGGCATTTCCAAATGAGATGGCTATGGCTGACTGGAAATTAACGAATGAAAATCTGATGGCT

AGGAACGATCGAGGTTATGGAGGTAACAATGGTGATTTGGAGTTTGCGTTTATGCCTCAAAACGGTAGACAGTGA

>AT1G77080.2 | Symbols: FLM, AGL27, MAF1 | FLOWERING LOCUS M, AGAMOUS-like 27, MADS AFFECTING FLOWERING 1 | chr1:28955679-28959845 FORWARD LENGTH=579

ATGGGAAGAAGAAAAATCGAGATCAAGCGAATCGAGAACAAAAGCAGTCGACAAGTCACTTTCTCCAAACGACGCAATGGTCTCATCGACAAAGCTCGAC

AACTTTCGATTCTCTGTGAATCCTCCGTCGCTGTTGTCGTCGTATCTGCCTCCGGAAAACTCTATGACTCTTCCTCCGGTGACGAGATAGAAGCGCTGTT

CAAGCCGGAGAAACCTCAATGTTTTGAACTCGATCTTGAAGAAAAAATTCAGAATTATCTTCCACACAAGGAGTTACTAGAAACAGTCCAAAGCAAGCTT

GAAGAACCAAATGTCGATAATGTAAGTGTAGATTCTCTAATTTCTCTGGAGGAACAACTTGAGACTGCTCTGTCCGTAAGTAGAGCTAGGAAGGCAGAAC

TGATGATGGAGTATATCGAGTCCCTTAAAGAAAAGGAGAAATTGCTGAGAGAAGAGAACCAGGTTCTGGCTAGCCAGATGGGAAAGAATACGTTGCTGGC

AACAGATGATGAGAGAGGAATGTTTCCGGGAAGTAGCTCCGGCAACAAAATACCGGAGACTCTCCCGCTGCTCAATTAG

>AT1G77950.1 | Symbols: AGL67 | AGAMOUS-like 67 | chr1:29307029-29309667 FORWARD LENGTH=759

ATGGGTCGGGTTAAATTGGAGTTAAAGCGTATAGAGAAGAGCACGAACCGACAAATTACGTTCTCAAAACGTAAAAAAGGTTTAATAAAAAAGGCTTATG

AATTGTCAACACTTTGTGACATCGATCTTGCCCTCCTCATGTTCTCTCCCTCTGATCGACTATGTCTCTTTTCCGGTCAAACAAGGATCGAGGACGTTTT

GGCGAGGTACATCAATCTTCCTGATCAAGAAAGAGAAAACGCCATAGTTTTCCCTGATCAGAGCAAGCGCCAAGGTATCCAAAACAAAGAGTATTTGTTA

AGGACTTTGGAAAAACTCAAAATTGAGGATGACATGGCTCTCCAAATCAACGAACCTCGTCCAGAAGCCACCAACTCCAATGTCGAGGAACTTGAGCAAG

AAGTTTGTAGATTACAACAACAGCTTCAGATATCAGAGGAAGAACTCAGGAAATTCGAACCAGATCCAATGAGGCTAACATCAATGGAGGAGATTGAAGC

ATGTGAAGCTAACCTCATCAATACGTTGACACGTGTCGTCCAGAGAAGGGAACATTTGTTGAGAAAGTCCTGCGAAGCACAAAGTAACCAACAAAGCATG

GACGGGATCCTTCTAAATGACATCGTCGAGGATTGGGGACCTGAGCCCGAGCCCAAACAAGCCCATATGATAGCTAATTCGGCCCATCATTCAAATCAGC

CAAGCTATGATTTACTCTTACGCAGGAGTAATTCGAGTTCGAACCAAAATCCAAAATGA

>AT1G77980.1 | Symbols: AGL66 | AGAMOUS-like 66 | chr1:29315212-29317067 REVERSE LENGTH=999

ATGGGTCGAGTGAAATTGGAGATAAAACGAATAGAAAACACAACGAATCGACAAGTTACATTCTCCAAAAGACGAAATGGTCTTATAAAGAAAGCTTATG

AATTGTCCATTCTTTGTGACATCGACATTGCTCTCCTCATGTTCTCTCCCTCTGATCGCCTTAGCCTCTTTTCGGGCAAAACAAGGATTGAAGACGTTTT

CTCTAGATACATCAATCTTTCTGATCAAGAACGAGAGAATGCTCTAGTATTCCCCGATCAAAGTCGACGCCCAGATTTCCAGAGCAAAGAGTATCTACTC

CGGACTTTGCAGCAACTCAAGGCTGAGAATGATATTGCTCTTCAACTTACCAACCCTACAGCTATCAACTCCGACGTCGAGGAACTTGAGCATGAAGTTT

ATAAGTTACAACAACAACTTCTCATGGCAGAGGAAGAACTAAGGAAATACGAACCAGATCCAATCAGGTTTACAACAATGGAAGAGTACGAAACTTGTGA

GAAGCAGCTCATGGACACCTTAACACGTGTCAATCAACGACGGGAACATATATTGAGTCAAGACCAATTATCTTCATATGAAGCATCTGCTTTACAACAA

CAGCAAAGCATGGGTGGGCCTTTTGGAAACGACGTCGTTGGAGGATGGCTGACTGAAAATGGGCCTAACGAAGCCCATTTATTCGACGCATCGGCACATT

CAGCAATGTATGAAACTTTATTGCAAGGAAGTAGCTCAAGCTCGAACCAAAACAACATTATGGGTGAATCCAATGTGTCAAATCATAACGGTGACATGTT

TCAAGAATGGGCCCAAGCCTATAATTCTACTACGGCCCATAACCCTTCGACTCTATTTCCTCCTATGCAGCATCAGCATGGACTGGTGGTTGATCCCAAT

ATAGAAGAAATTGAAATACCGGTTATGAAGAAGGATGCACAAGCAGACCACGAGGTCTCCGACTATGATATAAGAATGCCTCAGCTCAGTAGCCAATAA

>AT2G03060.1 | Symbols: AGL30 | AGAMOUS-like 30 | chr2:901614-903639 FORWARD LENGTH=1146

ATGGGAAGGGTAAAATTGAAGATAAAGAAGTTAGAGAACACAAATGGACGCCAATCTACATTTGCTAAAAGGAAAAATGGGATCTTGAAAAAGGCTAATG

AGCTATCTATTCTTTGTGACATTGATATTGTTCTTCTTATGTTCTCTCCTACTGGCAAGGCTGCAATATGTTGCGGTACACGAAGTAGCATGGAAGAGGT

GATTGCTAAGTTTTCTCAAGTAACACCGCAGGAAAGAACGAAAAGGAAGTTCGAGAGTCTTGAAAACTTGAAGAAAACTTTCCAAAAGTTGGATCACGAT

GTAAATATACGCGAATTTATAGCCTCAAGTAATTCAACAGTAGAGGACTTGAGTACTCAAGCAAGGATTCTGCAGGCTCGGATTTCTGAGATACATGGAA

GATTAAGTTATTGGACGGAACCAGATAAGATTAACAATGTTGAACACTTGGGACAGCTCGAAATTTCGATTAGGCAATCCCTTGATCAATTGCGTGCACA

CAAGGAACATTTTGGGCAGCAGCAACAGGCAATGCAAATAGAAAACGCAAACTTTGTTAAGGATTGGTCAACATGCTCGATGCAAGATGGGATTCAGATT

CCTTTAGAACAACAGCTTCAATCTATGTCATGGATTCTTAATAGCAACACCACCAACATTGTCACCGAGGAACACAATTCAATCCCGCAGAGGGAAGTCG

AGTGCTCAGCGAGTTCTTCATTCGGGAGCTATCCAGGCTACTTTGGAACAGGGAAATCTCCTGAAATGACAATTCCGGGTCAAGAAACAAGCTTTCTTGA

TGAACTAAACACCGGACAGCTGAAACAGGACACAAGCTCGCAGCAGCAGTTCACTAATAATAATAATATCACAGCATACAATCCCAATCTTCACAATGAT

ATGAATCATCACCAAACGTTGCCTCCTCCTCCTCTTCCTCTTACTCTTCCGCATGCTCAGGTGTATATTCCAATGAATCAGAGAGAGTATCATATGAATG

GATTCTTTGAAGCACCACCACCTGATTCTTCTGCTTACAACGACAACACCAACCAAACCAGGTTTGGTTCTAGCAGCAGCTCCTTGCCTTGCTCAATCTC

AATGTTCGACGAATACTTGTTTTCCCAGATGCAGCAGCCGAACTGA

>AT2G03710.1 | Symbols: SEP4, AGL3 | SEPALLATA 4, AGAMOUS-like 3 | chr2:1129622-1131628 FORWARD LENGTH=777

ATGGGAAGAGGGAAAGTTGAGCTGAAGAGGATAGAGAACAAGATCAATAGACAAGTTACTTTTGCAAAGAGAAGAAATGGTTTGCTCAAGAAGGCTTATG

AGCTTTCTGTCCTTTGTGATGCTGAGATTGCTCTTCTCATTTTCTCTAACCGTGGCAAGCTCTACGAATTCTGCAGCAGCCCTAGTGGTATGGCGAGGAC

GGTTGATAAGTATAGAAAACATAGTTATGCAACAATGGATCCAAATCAATCAGCTAAAGACTTGCAGGATAAGTATCAAGACTACTTGAAGCTTAAATCA

AGAGTTGAGATCCTTCAACATTCACAAAGGCATTTGCTAGGTGAAGAGCTATCCGAGATGGATGTGAATGAGCTTGAGCATCTCGAACGCCAAGTAGATG

CATCACTAAGACAAATAAGATCTACCAAGGCTCGGTCTATGCTTGATCAACTATCTGACCTCAAAACTAAGGAGGAAATGTTATTGGAAACCAATAGAGA

TCTTAGGAGAAAGTTGGAGGACAGTGATGCAGCACTTACTCAATCGTTTTGGGGAAGTTCTGCTGCAGAACAACAACAACAACATCAACAACAGCAACAA

GGCATGAGCTCTTATCAATCAAACCCTCCAATTCAGGAAGCAGGTTTCTTCAAGCCTCTACAAGGCAATGTAGCATTGCAAATGAGCAGTCATTACAATC

ACAATCCTGCAAATGCAACCAACTCTGCAACAACATCACAGAATGTTAATGGATTCTTCCCTGGATGGATGGTCTGA

>AT2G14210.1 | Symbols: ANR1, AtANR1, AGL44 | ARABIDOPSIS NITRATE REGULATED 1, AGAMOUS-like 44 | chr2:6018841-6023585 FORWARD LENGTH=705

ATGGGGAGAGGGAAGATAGTTATACGAAGGATCGATAACTCTACAAGTAGACAAGTGACTTTCTCCAAGAGAAGGAGTGGTTTGCTTAAGAAAGCTAAAG

AGTTATCGATCCTTTGTGATGCAGAAGTTGGTGTTATCATATTCTCTAGCACCGGAAAGCTCTACGACTACGCAAGCAATTCAAGTATGAAAACAATCAT

TGAGCGGTACAACAGAGTAAAAGAGGAGCAGCATCAACTTCTGAATCATGCCTCAGAGATAAAGTTTTGGCAAAGAGAGGTTGCAAGTTTGCAGCAGCAG

CTCCAATATCTACAAGAATGCCACAGGAAACTAGTGGGAGAGGAACTTTCTGGAATGAATGCTAACGACCTACAAAACCTTGAAGACCAGCTAGTAACAA

GTCTAAAAGGTGTTCGTCTCAAAAAGGATCAACTTATGACAAATGAAATCAGAGAACTTAATCGTAAGGGACAAATCATCCAAAAAGAGAATCACGAGCT

ACAAAATATTGTAGATATAATGCGTAAGGAAAATATTAAATTGCAAAAGAAGGTTCATGGAAGAACAAATGCGATTGAAGGCAATTCAAGTGTAGATCCA

ATAAGCAATGGAACCACAACATATGCACCACCGCAACTTCAACTCATACAACTACAACCAGCTCCTAGAGAAAAATCAATCAGACTAGGGCTACAACTTT

CCTAG

>AT2G22540.1 | Symbols: AGL22, FAQ1, SVP | SHORT VEGETATIVE PHASE, Flowering Arabidopsis QTL1, AGAMOUS-like 22 | chr2:9580417-9583603 FORWARD LENGTH=723

ATGGCGAGAGAAAAGATTCAGATCAGGAAGATCGACAACGCAACGGCGAGACAAGTGACGTTTTCGAAACGAAGAAGAGGGCTTTTCAAGAAAGCTGAAG

AACTCTCCGTTCTCTGCGACGCCGATGTCGCTCTCATCATCTTCTCTTCCACCGGAAAACTGTTCGAGTTCTGTAGCTCCAGCATGAAGGAAGTCCTAGA

GAGGCATAACTTGCAGTCAAAGAACTTGGAGAAGCTTGATCAGCCATCTCTTGAGTTACAGCTGGTTGAGAACAGTGATCACGCCCGAATGAGTAAAGAA

ATTGCGGACAAGAGCCACCGACTAAGGCAAATGAGAGGAGAGGAACTTCAAGGACTTGACATTGAAGAGCTTCAGCAGCTAGAGAAGGCCCTTGAAACTG

GTTTGACGCGTGTGATTGAAACAAAGAGTGACAAGATTATGAGTGAGATCAGCGAACTTCAGAAAAAGGGAATGCAATTGATGGATGAGAACAAGCGGTT

GAGGCAGCAAGGAACGCAACTAACGGAAGAGAACGAGCGACTTGGCATGCAAATATGTAACAATGTGCATGCACACGGTGGTGCTGAATCGGAGAACGCT

GCTGTGTACGAGGAAGGACAGTCGTCGGAGTCTATTACTAACGCCGGAAACTCTACCGGAGCGCCTGTTGACTCCGAGAGCTCCGACACTTCCCTTAGGC

TCGGCTTACCGTATGGTGGTTAG

>AT2G22630.1 | Symbols: AGL17 | AGAMOUS-like 17 | chr2:9618372-9621641 FORWARD LENGTH=684

ATGGGGAGAGGGAAGATTGTGATCCAGAAGATCGATGATTCCACGAGTAGACAAGTCACTTTCTCCAAAAGAAGAAAGGGTCTCATCAAGAAAGCTAAAG

AACTTGCTATTCTCTGCGACGCCGAGGTCTGTCTCATCATTTTCTCCAACACTGACAAGCTCTATGACTTTGCCAGCTCCAGTGTGAAATCTACTATTGA

ACGATTCAATACGGCTAAGATGGAGGAGCAAGAACTAATGAACCCTGCATCAGAAGTTAAGTTTTGGCAGAGAGAGGCTGAAACTCTAAGGCAAGAATTG

CACTCATTGCAAGAAAATTATCGGCAACTAACGGGAGTGGAATTAAATGGTTTGAGCGTTAAGGAGTTACAAAACATAGAGAGTCAACTTGAAATGAGTT

TACGTGGAATTCGTATGAAAAGGGAACAAATTTTGACCAATGAAATTAAAGAGCTAACCAGAAAGAGGAATCTTGTTCATCATGAAAACCTCGAATTGTC

GAGAAAAGTACAAAGGATTCATCAAGAAAATGTCGAACTATACAAGAAGGCTTATGGAACGTCGAACACAAATGGATTGGGACATCATGAGCTAGTAGAT

GCAGTTTATGAATCCCATGCACAGGTTAGGCTGCAGCTAAGCCAGCCTGAGCAGTCCCATTATAAGACATCTTCAAACAGCTAA

>AT2G24840.1 | Symbols: DIA, AGL61 | DIANA, AGAMOUS-like 61 | chr2:10581082-10581876 FORWARD LENGTH=795

ATGTATGTTACAAAATATAAAAATAAATTACCAACTTTGCAAGTTGATCTCATAATAATGCCACACCAGACTCAAGCATGTATATATAAACAAACACTTT

CTCTCCACCAAATATTCCAACAAAGAAAAGCAACTAAAACCCTCCATACAAAAAAAACTATGATGTCGAAGAAGAAAGAAAGCATCGGACGACAAAAAAT

TCCAATGGTAAAAATAAAGAAAGAGAGCCACCGGCAAGTCACATTCTCCAAACGCAGAGCCGGTCTCTTCAAGAAAGCTAGTGAGCTTTGCACTTTGTGT

GGTGCAGAGATTGGGATCATCGTGTTTTCTCCCGCGAAAAAGCCTTTCTCATTCGGACATCCAAGTGTTGAATCTGTATTGGATCGCTACGTGTCTCGAA

ACAATATGTCCTTAGCTCAGTCGCAGCAACTGCAAGGAAGCCCTGCAGCGAGCTGCGAACTGAATATGCAGTTAACGCATATTTTGAGCGAGGTAGAAGA

AGAGAAGAAGAAGGGTCAAGCGATGGAAGAGATGAGAAAAGAGAGTGTGAGGCGGTCGATGATTAATTGGTGGGAAAAGCCAGTAGAGGAGATGAATATG

GTTCAGTTACAGGAAATGAAGTATGCATTGGAGGAGTTGAGGAAGACGGTTGTGACAAACATGGCGTCGTTTAATGAGGCCAAAGACGATGTGTTTGGTT

TCTTGGACAACAAAGTGACAGTCCCTCCTTACGTGAACATGCCTTCTGGTCCCTCAAATATTTATAATTTTGCCAATGGAAATGGTTGTTTCTGA

>AT2G26320.1 | Symbols: AGL33 | AGAMOUS-like 33 | chr2:11205389-11206287 REVERSE LENGTH=330

ATGAAGAGAACAATCAAAAATAAAAATAAACAAATAGTTAAAGAAAATATGGGGAGGAAAAAACTAAAATTGAAGAGAATAGAGTCCCTAAAAGAGAGAA

GTAGCAAATTTTCAAAACGTAAAAAAGGTCTTTTTAAAAAGGCAGAAGAAGTAGCATTGTTATGCGACTCGGATATAATGTTAATTGTTGTTTCTCCCAC

CGAGAAGCCTACAGTTTTTAACACTCGTTCTAGATCATTCCATACAATCCTTGAGAGGTTTTGCATGCTTTCATTACAAGAACGGGAAGAGAGGTGTGAT

CTTTCATATTTTTATATAATTATTACATAA

>AT2G28700.1 | Symbols: AGL46 | AGAMOUS-like 46 | chr2:12317384-12318724 REVERSE LENGTH=990

ATGGCAAGAAAAAAGTTAAATCTAACTTACATTTTCAATGATAGAATGAGGAAAAGATCGTTCAAGCAGAGGAGAGAAGGATTTCTGAAGAAACTCAATG

ATCTCAAGGTTTTATGCGATGTTAATGCATGTGCGGTCGTCTACAATCCATTCAACTCAAATCCAGATGTGTGGCCATCAAAATCGGAGGTGAATAATAT

CATCAAGAAATTTGAGATGTTACCGGAGACCCAAAAGAAAGTTAAATCGGTGAACCACGAAGAATTTCTCAATCTATACATCTCAAAAGTCGAAAAGCAA

AGTAAGAAATTAATTGTAGAAAATAAGGAGACTTGCTTAAAGGAGGTCATGTTCAAGTGTCTTGGTGGGAACATGGGAGATTTTGTTATGAATGATAATG

ATCGTCTTGACTTATGTAAGTTTATTGATCACTATCTTAGAAATCTTTATCATCATAAAAATGTAACCCTCAACAATCCAAATTTTGAAATCGGAGAATC

TTCTTCATTAATGGATATGGCACCAACCGCTACAACTGGAAACATGGCAACAACTGTAGTAGATGAAGGCATGACACCACTTCTTATTGCTGAAGGGAGT

TCCTCCTCATTTTTGAATTCTCCACTCTTCAACTCTCCTCAGCTAACCAACGAGTTGCAGCTTATAGTCTCTCAAAACCATAGATTAGAGAATTCTCTGG

CAAGTAATCTCTTTTTCAGTGAAGGGCAAGACATTTGCATTCCAGATATGAATCAGTCCATAATCCCATCAAACCAAGGAGCAGAACATGTGGATTTTCT

TGAAAGTAATTTTCTACCTAATAACAATCAAGAAGTTTATATCCCAGTTATGGATCAGGATGAAGTTTACAATCCAAATCAAAATCATTATGAGAACCAA

CAAGGATTCATTGACGAGATGATGAAATATGCTGAGAAAACAAGTTTTCCTTGGATGGTAGAGAACCACTGTTATAATCACAACCAGTAA

>AT2G34440.1 | Symbols: AGL29 | AGAMOUS-like 29 | chr2:14526950-14527468 FORWARD LENGTH=519

ATGGGTCGGAGAAAGATCAAGATGGAGATGGTTCAGGACATGAACACACGACAGGTTACCTTTTCAAAACGGAGGACTGGTTTGTTCAAGAAGGCGAGCG

AGTTAGCCACGCTCTGCAACGCTGAGTTGGGCATCGTTGTCTTTTCACCAGGAGGCAAGCCTTTCTCCTACGGGAAACCGAATCTTGATTCTGTTGCAGA

GCGATTCATGAGAGAATATGATGATTCAGACAGTGGCGATGAAGAAAAAAGTGGTAATTACAGGCCTAAACTGAAGAGGCTGAGTGAACGTCTCGATTTG

CTCAACCAAGAGGTTGAAGCTGAGAAGGAACGAGGCGAGAAGAGTCAGGAGAAGCTTGAATCTGCTGGGGATGAGAGATTCAAGGAGTCCATTGAGACGC

TTACCCTCGATGAACTCAATGAATACAAAGATAGGCTTCAGACAGTCCATGGTAGGATTGAAGGTCAAGTCAATCACTTGCAGGCTTCGTCTTGCCTCAT

GCTTCTCTCCAGAAAATAG

>AT2G40210.1 | Symbols: AGL48 | AGAMOUS-like 48 | chr2:16793213-16794328 REVERSE LENGTH=1116

ATGACAAGAAAGAAAGTAAAACTTGTATGGATCGAGAATGACAAGTCAAGAGCAACAAGTTTGCAAAAAATGAGGGTAGGATTACTTAAAAAGGTGAAAG

AGCTAACCATATTGTGTGCTGTAAGGGCTATTGTAATCATTTTCAGCCCCGATAAGGTTGGACCATTGGTGTGGCCATCTCCTCAGGCGACTCATGGTCT

CCTAGACGAATTTTTCGCTTTACCGAAGTCCGTGCAGAAGAAGAAAGAGTCAAATGTCGAATCCTACCTAAAAGAGAAGACACATAAGTTTCAAGAACAA

TTGAAGAAGAGTAAAAAGAAGAACAAGGAGCATGTCATTGATGAGCTGATGATGCAACTCCAAAGCGGTCGTGAAATTGCTGATCTTAACCAAAGTGAGA

TGTATGCCTTGTTATCTTTCTCAAGAGATACTATCCTACTTTGTAGGAAGAAACTAGCTTTCATGCAATTTCCTCCTCTTCGCGATCCACCAGTGTTTCC

ATTTGAAATACAAGTCGAAGAGTTCAAAACCACCACAAATGATGGTTTTGTAGGAGGCGGTCAAGATAATAAAAGAGCTGGAAGGACAGATGAAGCAACA

AGATTCATCAACACCGATATATTTAAGCAGAGTAAAAGTTATTACTTTTTTGATGAATGGGTTTTTCCACCTAGTCCACCTAAATATGAAATACCGCAAC

AAATGGAGAACGGAAATCCAAACCCTAAAAGCTATCGTCTTTATCAAGGAAGCAGTAGCAACGGAAACCCTCATTTGGAAATGGATCCATTTCGTCTCCA

AATGATGACTTCCCAAGGTTTGGCTGGGTCAGTCTCCCAACCTTTACAACATCACAGCATGATCAATAATCCAACAATGGCTATGAATCAACCAAGCCAA

GATCCTTTTGATTACATGAGAAGTGAGCTGGGAATAAACGAAGGAATCAACATTAACAACTCACAATTTTACATGAGCAACAACACTATAACGGCTAATG

ATGGTGTTCGCCAAGAACCATATCCTAATGTAACAACCGCTGGAGAAAACAATGGTGATGCGACGACGTCTAATACCAACATGGTGTGGCCGGGTTTTAA

CAATCATCATTTCTAA

>AT2G42830.1 | Symbols: AGL5, SHP2 | AGAMOUS-like 5, SHATTERPROOF 2 | chr2:17820602-17823806 FORWARD LENGTH=741

ATGGAGGGTGGTGCGAGTAATGAAGTAGCAGAGAGCAGCAAGAAGATAGGGAGAGGGAAGATAGAGATAAAGAGGATAGAGAACACTACGAATCGTCAAG

TCACTTTCTGCAAACGACGCAATGGTTTACTCAAGAAAGCTTATGAGCTCTCTGTCTTGTGTGACGCTGAGGTTGCTCTTGTCATCTTCTCCACTCGAGG

CCGTCTCTACGAGTACGCCAACAACAGTGTGAGAGGAACAATAGAAAGGTACAAGAAAGCTTGCTCCGACGCCGTTAACCCTCCGACCATCACCGAAGCT

AATACTCAGTACTATCAGCAAGAGGCGTCTAAACTCCGGAGACAGATTCGGGACATTCAGAATTTGAACAGACACATTCTTGGTGAATCTCTTGGTTCCT

TGAACTTTAAGGAACTCAAGAACCTTGAAAGTAGGCTTGAGAAAGGAATCAGTCGTGTCCGATCCAAGAAGCACGAGATGTTAGTTGCAGAGATTGAATA

CATGCAAAAAAGGGAAATCGAGCTGCAAAACGATAACATGTATCTCCGCTCCAAGATTACTGAAAGAACAGGTCTACAGCAACAAGAATCGAGTGTGATA

CATCAAGGGACAGTTTACGAGTCGGGTGTTACTTCTTCTCACCAGTCGGGGCAGTATAACCGGAATTATATTGCGGTTAACCTTCTTGAACCGAATCAGA

ATTCCTCCAACCAAGACCAACCACCTCTGCAACTTGTTTGA

>AT2G45650.1 | Symbols: AGL6, RSB1 | AGAMOUS-like 6, REDUCED SHOOT BRANCHING 1 | chr2:18804453-18806291 FORWARD LENGTH=759

ATGGGAAGAGGGAGAGTGGAGATGAAGAGGATAGAGAACAAGATTAATAGACAAGTGACCTTCTCAAAAAGAAGAAACGGTTTGCTGAAGAAAGCTTATG

AGCTTTCTGTTCTTTGCGATGCCGAAGTTGCTCTCATCATCTTCTCAAGCCGTGGCAAGCTCTACGAGTTTGGTAGTGTTGGAATTGAAAGCACAATCGA

ACGGTATAATCGTTGTTACAACTGCTCTCTAAGCAATAATAAGCCTGAAGAGACTACACAGAGTTGGTGTCAGGAGGTGACAAAGCTTAAATCCAAATAC

GAATCTCTTGTTCGTACTAACAGGAATTTGCTTGGAGAAGATCTTGGAGAAATGGGTGTGAAGGAACTGCAAGCGCTCGAGAGGCAGCTCGAAGCCGCTC

TTACCGCGACTCGACAGCGCAAGACACAAGTTATGATGGAAGAAATGGAAGACCTTAGGAAAAAGGAGAGGCAACTAGGAGACATAAACAAACAACTCAA

GATTAAGTTTGAAACGGAAGGCCATGCTTTCAAAACCTTTCAAGACTTATGGGCAAACTCGGCGGCATCGGTGGCCGGGGATCCAAACAATTCTGAATTT

CCGGTAGAGCCTTCTCATCCTAATGTATTGGATTGCAACACCGAACCCTTTTTACAAATAGGGTTTCAACAACATTACTACGTGCAAGGTGAAGGGTCTT

CGGTATCAAAGAGTAACGTGGCAGGTGAGACTAATTTCGTCCAAGGTTGGGTTCTTTGA

>AT2G45660.1 | Symbols: AGL20, SOC1, ATSOC1 | SUPPRESSOR OF OVEREXPRESSION OF CO 1, AGAMOUS-like 20 | chr2:18807799-18810193 REVERSE LENGTH=645

ATGGTGAGGGGCAAAACTCAGATGAAGAGAATAGAGAATGCAACAAGCAGACAAGTGACTTTCTCCAAAAGAAGGAATGGTTTGTTGAAGAAAGCCTTTG

AGCTCTCAGTGCTTTGTGATGCTGAAGTTTCTCTTATCATCTTCTCTCCTAAAGGCAAACTTTATGAATTCGCCAGCTCCAATATGCAAGATACCATAGA

TCGTTATCTGAGGCATACTAAGGATCGAGTCAGCACCAAACCGGTTTCTGAAGAAAATATGCAGCATTTGAAATATGAAGCAGCAAACATGATGAAGAAA

ATTGAACAACTCGAAGCTTCTAAACGTAAACTCTTGGGAGAAGGCATAGGAACATGCTCAATCGAGGAGCTGCAACAGATTGAGCAACAGCTTGAGAAAA

GTGTCAAATGTATTCGAGCAAGAAAGACTCAAGTGTTTAAGGAACAAATTGAGCAGCTCAAGCAAAAGGAGAAAGCTCTAGCTGCAGAAAACGAGAAGCT

CTCTGAAAAGTGGGGATCTCATGAAAGCGAAGTTTGGTCAAATAAGAATCAAGAAAGTACTGGAAGAGGTGATGAAGAGAGTAGCCCAAGTTCTGAAGTA

GAGACGCAATTGTTCATTGGGTTACCTTGTTCTTCAAGAAAGTGA

>AT3G02310.1 | Symbols: AGL4, SEP2 | AGAMOUS-like 4, SEPALLATA 2 | chr3:464554-466687 REVERSE LENGTH=753

ATGGGAAGAGGAAGAGTAGAGCTCAAGAGGATAGAGAACAAAATCAACAGACAAGTGACGTTTGCTAAACGTAGAAATGGTTTGCTGAAAAAAGCTTATG

AGCTTTCTGTTCTCTGCGATGCTGAAGTCTCTCTCATCGTCTTCTCCAACCGTGGCAAGCTCTACGAGTTCTGCAGCACCTCCAACATGCTCAAGACACT

GGAAAGGTATCAGAAGTGTAGCTATGGCTCCATTGAAGTCAACAACAAACCTGCTAAAGAGCTTGAGAACAGCTACAGAGAGTACTTGAAGCTGAAAGGT

AGATATGAAAATCTGCAACGTCAGCAGAGAAATCTTCTTGGAGAGGATCTTGGACCTCTGAATTCAAAGGAGCTAGAGCAGCTTGAGCGTCAACTAGACG

GCTCTCTGAAGCAAGTTCGCTGCATCAAGACACAGTATATGCTTGACCAGCTCTCTGATCTTCAAGGTAAGGAGCATATCTTGCTTGATGCCAACAGAGC

TTTGTCAATGAAGCTGGAAGATATGATCGGCGTGAGACATCACCATATAGGAGGAGGATGGGAAGGTGGTGATCAACAGAATATTGCCTATGGACATCCT

CAGGCTCATTCTCAGGGACTATACCAATCTCTTGAATGTGATCCCACTTTGCAAATTGGATATAGCCATCCAGTGTGCTCAGAGCAAATGGCTGTGACGG

TGCAAGGTCAGTCCCAACAAGGAAACGGCTACATCCCTGGCTGGATGCTGTGA

>AT3G04100.1 | Symbols: AGL57 | AGAMOUS-like 57 | chr3:1075299-1075922 FORWARD LENGTH=624

ATGTCATCTACGAAGCAAGCAAAGGGAAGAAAAACAAAGGGGAAGCAAAAGATCGAGATGAAGAAGGTGGAGAACTATGGAGATAGGATGATTACGTTCT

CAAAACGTAAAACCGGAATTTTTAAGAAAATGAACGAGCTCGTAGCAATGTGTGACGTTGAAGTGGCTTTCTTGATTTTCTCTCAACCCAAGAAGCCCTA

TACATTCGCACATCCGTCTATGAAGAAAGTGGCTGACCGGTTAAAGAACCCTTCGAGACAAGAACCATTAGAGAGAGACGATACCAGACCCCTCGTCGAA

GCTTATAAGAAACGAAGGCTCCACGACCTCGTAAAAAAAATGGAGGCGCTCGAAGAGGAGCTTGCGATGGATCTAGAGAAGTTGAAACTGTTGAAGGAAT

CGAGAAATGAAAAGAAGTTAGATAAAATGTGGTGGAACTTTCCTTCGGAAGGTTTGAGCGCGAAGGAGCTGCAGCAAAGGTACCAAGCGATGCTCGAGTT

ACGTGATAACTTATGCGACAATATGGCTCACTTACGATTGGGAAAAGACTGTGGTGGTTCATCTTCTGTTCGTGTGGGACGTCGAGTTTCTGGTGGTGTT

CGTCTGTTCGATCGTGAAGCATGA

>AT3G05860.1 | Symbols: AGL45 | agamous-like 45 | chr3:1751406-1752355 REVERSE LENGTH=783

ATGACGAGGAAGAAGCTAAACCTATCTTACATCACCAATGAGTCAATGAGAAAAGCAACATTCAATAAGAGAAAGAAAGGGCTTGTGAAAAAAATCCACG

AGCTTTCCGTTCTCTGCGGAATCGAAGCATGTGCAGTGATTTATAGTCCGTTCAACTCAAACCCTGAAGTCTGGCCATCGAATTCAGAAGTTAAAAATGT

AATGGAAAATTTTGAGATGTTGACAAAGTTGGAGCAAGAGAAGAAAATGGTGAGCCACGAAGGTTTTATCAGACAAAACATCTCAAAAACTATGGAGAGT

AACAACAAAAAGATGATCGATAATGCGGAGAGGACGATGAAAGAAGCCATGTTCCAACTTCTTAGTGGAAAAGGGGAGAAGCTTAATTTGACTGATAGAA

ACCGTGAAGATTTGTGTAAGTACATTGATCAATATCTTAAAGAACTTTATCACCACAAAAACAAAACCATAAATCAATCACATATTGAACCTGGAGAATC

TTCGGGAGCTACAAATGCCATGACACCAACATCTGTTGTTGAACCTATTATCTCTTCAATCCAGCGACCAAATCAGAATCCAAATTTTAATCATCTTAGT

CATAATCAATATCAATATCAACAACAGTTTGGATATCCGATTTTGGTTCAAGATGGAATTTACAATCCGAGTCAAATTCAGAATCAACATGAGGAATGGC

TGGATGATCATATGATGAATCATTCTAAGGAAATAAGTCATCCTTTGATGGATGATAACAACTTTTACTACCAACAACCCTAG

>AT3G18650.1 | Symbols: AGL103 | AGAMOUS-like 103 | chr3:6417344-6418504 REVERSE LENGTH=1161

ATGGCTTCTTCTTCGTCCTCTTCTCTTTCGTTTTCAACATCGAAGAAGAACAAAACCTTTTTCAAGAAACCTAATTCTGCTTTTTCTTCTTCGAGGGCCA

CAAGTTTGATCAAAAGACAACAGACTGTCTTTAAGAAAGCTAAAGAGCTTTCAATTCTCTGTGACATCGATGTCTGCGTCATCTGTTACGGATCAAACGG

AGAGCTTAAGACATGGCCTGAAGAGAGAGAGAAGGTCAAAGCCATAGCTCGAAGGTACGGTGAATTAAGCGAGACGAAGCGTCGCAAAGGAAGCGTTGAT

CTTCACGAGTTCCTCGAGAAGATGAACAAGGATGATCCCGAGAAGGAGGAGAAGAAGAAGATTAAAGTGAGACGTGTACCCAAAGTCAAGTATCCAGTTT

GGGACCCTAGGTTTGACAATTACTCTGTGGAGCAACTCATGGGACTGGTTCAGTCCTTGGAACGAAACCTAACCAGGATCCAACATAGGACTTGCGCTGT

TGTTGAAGCTCAGGGGCAGAGGAGGGTACAGTACACGAACATGGCTAATCAAGAACTGATGATGGCTAATACGATGAATCAACTTCAACAACACTCAAAC

CAGGTTTCCATGTATCTGTGGAACCATGGAAACGGTGCTTTCTCACAAATCCCAGTCTCAGCATTGGCTTCAAACCAAACGCAATCTCTAGCTCCGATTC

CGCCTGAACTGATGATTTACCCGAATTCGGATGCGGGAAATTACTCGGGGTCTCTCGGGGTACAAGGAACTGGGATCAATGGGCTTCAGAACATGAACAT

GTTAACCTATAACAACATCAACAGCGTTAATGATTTCTCAAAACAGTTTGACCAGAATTCCAGAGCGGAAAGTTACTCTAGCTTACTTGGGGTACACGAA

GACGGAAACAATGAGTTTGAGAACCCTAACATGTCCAGCCGCAACAACTTCAATGTTCAAGACTGTGCAGGGTTACTTGGGATGCAAGGAGCTGGAACTA

ACGGGTTGCAGAGCATGAACATGCATGACTACAGTAACAACAACAGCATTAACTCTAATGGTCTTTCGCATCAGTATGTTCAATTTCCAACGTACAACAG

CCAACATCAGGACCGGGTTTTCAATTTGGATCAAAACGGAAACAATACTAGGTCTCTCTAA

>AT3G30260.1 | Symbols: AGL79 | AGAMOUS-like 79 | chr3:11909119-11912880 FORWARD LENGTH=750

ATGGGAAGAGGAAGGGTTCAGCTACGGCGGATCGAGAATAAGATAAGGAGACAAGTGACATTTTCAAAGCGAAGGACCGGTTTGGTAAAGAAAGCTCAAG

AGATCTCAGTGTTATGTGATGCTGAAGTTGCTTTGATTGTTTTCTCCCCCAAAGGCAAACTCTTTGAGTACTCTGCTGGTTCCAGCATGGAGAGAATTCT

TGATCGATATGAGAGGTCTGCCTACGCCGGTCAAGATATTCCTACACCAAATTTGGATTCACAGGGAGAGTGCTCAACAGAATGTTCAAAGCTCTTGAGG

ATGATTGATGTCCTGCAAAGAAGCCTGAGGCACTTAAGAGGAGAAGAGGTGGATGGTCTAAGTATCAGAGACCTTCAAGGTGTGGAGATGCAACTTGATA

CTGCCCTCAAGAAAACTCGCTCTAGAAAGAACCAGCTCATGGTAGAGTCCATAGCACAGCTTCAGAAAAAGGAGAAGGAACTAAAAGAACTGAAGAAACA

GCTAACAAAGAAGGCTGGTGAAAGGGAAGACTTTCAAACGCAAAACCTCAGCCATGACTTAGCCTCCTTGGCAACACCGCCATTTGAATCACCCCATGAG

CTTCGCCGGACAATATCTCCTCCTCCTCCTCCTTTATCTTCGGGGGATACATCACAAAGGGATGGAGTTGGAGAAGTAGCTGCCGGAACCCTAATTCGAA

GGACGAATGCAACGTTGCCTCATTGGATGCCCCAGCTCACCGGAGAATAG

>AT3G54340.1 | Symbols: AP3, ATAP3 | APETALA 3 | chr3:20119428-20121087 REVERSE LENGTH=699

ATGGCGAGAGGGAAGATCCAGATCAAGAGGATAGAGAACCAGACAAACAGACAAGTGACGTATTCAAAGAGAAGAAATGGTTTATTCAAGAAAGCACATG

AGCTCACGGTTTTGTGTGATGCTAGGGTTTCGATTATCATGTTCTCTAGCTCCAACAAGCTTCATGAGTATATCAGCCCTAACACCACAACGAAGGAGAT

CGTAGATCTGTACCAAACTATTTCTGATGTCGATGTTTGGGCCACTCAATATGAGCGAATGCAAGAAACCAAGAGGAAACTGTTGGAGACAAATAGAAAT

CTCCGGACTCAGATCAAGCAGAGGCTAGGTGAGTGTTTGGACGAGCTTGACATTCAGGAGCTGCGTCGTCTTGAGGATGAAATGGAAAACACTTTCAAAC

TCGTTCGCGAGCGCAAGTTCAAATCTCTTGGGAATCAGATCGAGACCACCAAGAAAAAGAACAAAAGTCAACAAGACATACAAAAGAATCTCATACATGA

GCTGGAACTAAGAGCTGAAGATCCTCACTATGGACTAGTAGACAATGGAGGAGATTACGACTCAGTTCTTGGATACCAAATCGAAGGGTCACGTGCTTAC

GCTCTTCGTTTCCACCAGAACCATCACCACTATTACCCCAACCATGGCCTTCATGCACCCTCTGCCTCTGACATCATTACCTTCCATCTTCTTGAATAA

>AT3G57230.1 | Symbols: AGL16 | AGAMOUS-like 16 | chr3:21177710-21180671 FORWARD LENGTH=723

ATGGGAAGGGGCAAGATCGCGATTAAGAGGATCAATAACTCTACGAGCCGTCAGGTTACGTTCTCGAAGCGAAGGAATGGATTGTTGAAGAAAGCTAAGG

AGCTTGCGATTCTCTGCGATGCTGAGGTTGGTGTCATCATCTTCTCCAGCACCGGTAGGCTCTACGATTTCTCCAGCTCCAGCATGAAATCGGTCATAGA

GAGATACAGCGATGCCAAAGGAGAAACCAGTTCAGAAAATGATCCCGCTTCAGAAATTCAGTTCTGGCAAAAGGAGGCTGCGATTCTAAAGCGTCAGCTA

CATAACTTGCAAGAAAACCACCGGCAAATGATGGGGGAGGAGCTCTCTGGACTAAGTGTAGAAGCTTTACAGAATTTGGAAAATCAGCTTGAATTGAGCC

TTCGTGGCGTTCGAATGAAAAAGGATCAAATGTTAATCGAAGAAATACAAGTACTTAACCGAGAGGGGAATCTCGTTCACCAAGAGAATTTAGACCTCCA

CAAGAAAGTAAACCTAATGCACCAACAGAACATGGAACTACATGAAAAGGTTTCAGAGGTCGAGGGTGTGAAAATCGCAAACAAGAATTCTCTTCTCACA

AATGGTCTAGACATGAGAGATACCTCGAACGAACATGTCCATCTTCAGCTCAGCCAACCGCAGCATGATCATGAGACGCATTCAAAAGCTATCCAACTCA

ACTATTTTTCCTTCATTGCATAA

>AT3G57390.1 | Symbols: AGL18 | AGAMOUS-like 18 | chr3:21233910-21235735 FORWARD LENGTH=771

ATGGGGAGAGGAAGGATTGAGATTAAGAAGATTGAGAATATCAACAGTCGTCAAGTCACTTTCTCTAAGAGACGAAACGGTTTGATCAAGAAGGCTAAAG

AGCTTTCGATTCTCTGTGACGCCGAGGTTGCTCTTATCATCTTCTCCAGCACCGGCAAGATTTACGATTTCTCCAGCGTCTGTATGGAGCAAATTCTTTC

TAGATATGGATACACTACTGCGTCCACTGAGCATAAACAACAAAGAGAACACCAACTTCTAATTTGTGCTTCACATGGAAATGAAGCTGTGTTGCGAAAT

GATGATTCTATGAAGGGGGAACTTGAAAGATTACAGCTTGCAATTGAGAGACTTAAGGGTAAGGAGCTTGAAGGTATGAGTTTCCCGGATCTTATTTCTC

TTGAAAACCAGTTGAACGAGAGCTTGCATAGTGTCAAGGATCAAAAGACACAAATCCTGCTCAACCAGATTGAGAGATCCAGGATACAGGAGAAAAAAGC

ATTGGAAGAAAACCAAATCTTGCGCAAACAGGTTGAGATGTTGGGGAGAGGTTCAGGACCAAAAGTGTTGAATGAAAGGCCTCAAGATTCTAGCCCAGAA

GCCGATCCCGAGAGCTCTTCATCAGAAGAGGATGAGAATGACAACGAGGAGCACCATTCCGACACTTCCTTGCAGTTGGGGTTGTCGTCGACGGGGTATT

GCACAAAGAGAAAGAAGCCGAAGATCGAACTGGTCTGCGATAACTCTGGGAGTCAAGTGGCTTCTGATTGA

>AT3G58780.1 | Symbols: SHP1, AGL1 | SHATTERPROOF 1, AGAMOUS-like 1 | chr3:21739150-21741766 FORWARD LENGTH=747

ATGGAGGAAGGTGGGAGTAGTCACGACGCAGAGAGTAGCAAGAAACTAGGGAGAGGGAAAATAGAGATAAAGAGGATAGAGAACACAACAAATCGTCAAG

TTACTTTCTGCAAACGACGCAATGGTCTTCTCAAGAAAGCTTATGAACTCTCTGTCTTGTGTGATGCCGAAGTTGCCCTCGTCATCTTCTCCACTCGTGG

CCGTCTCTATGAGTACGCCAACAACAGTGTGAGGGGTACAATTGAAAGGTACAAGAAAGCTTGTTCCGATGCCGTCAACCCTCCTTCCGTCACCGAAGCT

AATACTCAGTACTATCAGCAAGAAGCCTCTAAGCTTCGGAGGCAGATTCGAGATATTCAGAATTCAAATAGGCATATTGTTGGGGAATCACTTGGTTCCT

TGAACTTCAAGGAACTCAAAAACCTAGAAGGACGTCTTGAAAAAGGAATCAGCCGTGTCCGCTCCAAAAAGAATGAGCTGTTAGTGGCAGAGATAGAGTA

TATGCAGAAGAGGGAAATGGAGTTGCAACACAATAACATGTACCTGCGAGCAAAGATAGCCGAAGGCGCCAGATTGAATCCGGACCAGCAGGAATCGAGT

GTGATACAAGGGACGACAGTTTACGAATCCGGTGTATCTTCTCATGACCAGTCGCAGCATTATAATCGGAACTATATTCCGGTGAACCTTCTTGAACCGA

ATCAGCAATTCTCCGGCCAAGACCAACCTCCTCTTCAACTTGTGTAA

>AT3G61120.1 | Symbols: AGL13 | AGAMOUS-like 13 | chr3:22618414-22620466 REVERSE LENGTH=735

ATGGGAAGAGGCAAAGTGGAAGTGAAGAGAATTGAGAACAAGATCACTAGACAAGTAACCTTCTCCAAAAGAAAAAGTGGTTTGCTCAAGAAAGCCTACG

AACTCTCTGTTCTTTGTGATGCAGAGGTTTCTCTCATAATCTTCTCCACCGGTGGCAAGCTCTACGAGTTCAGCAATGTCGGAGTTGGCAGAACCATTGA

GAGGTACTATCGCTGTAAAGACAATCTTTTGGACAATGATACGCTTGAAGATACTCAGGGCTTGCGTCAAGAGGTGACAAAGCTCAAATGCAAATACGAA

TCTCTTCTTCGCACTCATAGGAATTTGGTTGGGGAAGATCTTGAAGGGATGAGTATAAAGGAATTGCAGACGCTGGAGAGACAGCTTGAAGGAGCTCTTT

CCGCAACCCGAAAACAAAAGACGCAGGTTATGATGGAACAAATGGAAGAGCTTCGGAGAAAGGAGAGGGAGCTCGGAGATATAAACAATAAGCTAAAGCT

TGAGACAGAAGATCATGATTTTAAAGGCTTTCAAGACCTACTGCTTAACCCGGTGCTCACCGCCGGTTGTTCCACTGATTTTTCCTTGCAATCAACTCAT

CAAAATTATATATCGGATTGCAACCTTGGATATTTTTTACAGATTGGGTTTCAACAACACTATGAGCAAGGTGAAGGATCTTCGGTGACAAAGAGTAATG

CAAGAAGTGATGCTGAGACCAACTTTGTCCAATGA

>AT3G66656.1 | Symbols: AGL91 | AGAMOUS-like 91 | chr3:2091262-2091798 REVERSE LENGTH=537

ATGGGTAGGAGAAAGATTAAGATGGAGAAAGTGCAAGACACAAACACGAAGCAAGTTACCTTCTCAAAACGTAGGCTGGGTTTGTTCAAGAAAGCGAGCG

AGCTTGCGACTCTGTGCAACGCGGAGGTTGGTATTGTTGTCTTTTCTCCAGGAAACAAACCGTATTCCTTCGGGAAACCGAATTTTGATGTGATTGCAGA

ACGGTTTAAGAATGAATTCGAAGAAGAAGAAGAAGGAGATAGCTGTGAAACATCAGGCTATAGTAGAGGCAATAGAGCTAGACAGGAGAAGAAGATATGT

AAACGCCTCAACTCGATTACTGAAGAAGCTGAAGCTGAGAAGAAACATGGTGAAGATCTTCACAAGTGGCTTGAATCTGCTGAACAGGATAAGTTCAATA

AGCCCATTGAGGAGCTTACGCTTGAGGAACTCAAGGAATTTGAGGCTAAGATTAAGAAAATAAGCTGTGGGATCCAAAGTAACATTAGTCATATGCAGGC

TTCGTCTTCTCTCATGTTTCTCTCTAATGATAATTAG

>AT4G02235.1 | Symbols: AGL51 | AGAMOUS-like 51 | chr4:980955-981711 FORWARD LENGTH=591

ATGAAGCAATCTTCTTTTTCTTCTTCTTCTTCGTCACGTAATTCAACTAGTTTAACAAACAGACTCAAAACCATCTTCAAAAAAGCTGAAGAGCTTTCGA

TTCTCTGTGCTATTGACGTTTGCGTCATCTATTACGGACCAGACGGTGAACTGAGAACATGGCCTAAGGAGAGAAATACAGTGAAAGACATGGCTTCGAG

ATATAAAGAGGCCACAAAACGCAAGAAGAAACGGACTCTTTCTACTCTGCAAGAAAGGCTTCGTATTGTTGAGTCGCAGAAACAACAAAACAAAAACTTG

GTGCATCAGAGTTTAACACCATCGTATCTGAACCAGATCCAACACTTGAATCCTAGCAACTTCTCGCCGTATATGTACAACCATGGAGATGCTGCTACTC

TCTCACAACTCCCACTGTCTGCTTCACTCTCCAATCAACTCCAATTACCTGAATCACTTGATGCGGCATGGTTTTGGTCAGAACATGTGTTTGGACAACA

TCACCAACAACAACAACTTTCAACATCCTGGCGTGTCAAACACACAAGAATACTCACCGTTTCTTTCGGTACAAGCATCTGCAGTGAATAA

>AT4G09960.1 | Symbols: STK, AGL11 | AGAMOUS-like 11, SEEDSTICK | chr4:6236713-6239409 REVERSE LENGTH=693

ATGGGAAGAGGAAAGATAGAAATAAAGAGGATAGAGAACTCAACAAATCGACAAGTGACGTTTTGCAAAAGAAGAAATGGACTTCTGAAGAAAGCCTATG

AGCTTTCGGTCCTTTGCGATGCAGAAGTTGCGCTCATTGTTTTCTCCACTCGTGGCCGTCTCTATGAATACGCCAATAACAACATAAGATCAACCATTGA

GAGGTACAAGAAAGCTTGTTCTGATAGCACCAACACTAGCACTGTCCAAGAAATCAATGCCGCGTACTATCAACAAGAATCTGCTAAGCTGAGACAACAG

ATCCAAACGATTCAAAACTCCAACAGGAATCTGATGGGAGACTCTTTGAGTTCCTTAAGTGTCAAGGAACTAAAACAAGTTGAGAATCGCCTTGAGAAAG

CTATCTCTAGGATCAGGTCCAAGAAGCATGAGTTGCTTTTAGTTGAAATCGAAAACGCGCAGAAAAGGGAGATTGAGCTTGACAATGAGAACATCTATCT

AAGAACTAAGGTAGCAGAAGTGGAGAGGTATCAACAACACCATCATCAAATGGTTAGTGGTTCAGAGATTAATGCAATTGAAGCTTTAGCCTCACGCAAT

TACTTTGCTCATAGCATTATGACTGCTGGTTCTGGATCTGGTAATGGAGGTTCTTACTCTGATCCCGACAAGAAAATTCTTCATCTCGGATAA

>AT4G11250.1 | Symbols: AGL52 | AGAMOUS-like 52 | chr4:6849578-6850567 FORWARD LENGTH=990

ATGAAGCAAGCTTCTTCTTCTTCGTCATGTAATCCAACAAGTTTAACAAACAGACTTAAAACAATCTTTAAGAAAGCAGAAGAGCTTTCGATTCTCTGTG

CTATTGACGTTTGCGTCATCTATTACGGACCAGACGGTGATCTGAGAACATGGCCTAAGGATAGAGAGACAGTGAAAAATATGGCTTTGAGGTATAAAGA

GGACAGAAAACGCAAGAAGTGCCTCAATCTTCATGAGTTCCTCGAGAAAGAGAAGGTGAAGGACAAAGACAAGTATAAGGGGAAGACGAATTATGTGAAG

AACCCTAATTGGTATCCAAACTTTGATCATTACTCTCCTCAACAACTCTCTCAATTGATTCAGTCCTTAGAACGAACACTTTCTACTCTCCAAAAAAGGC

TTCGTATTGTTGAGTCGCAGAAAAAACAGAACACAAACTTGGTGCATCAGAGTTTAACACCATCGTATCTGAACCAGACACAACACTTGGATCCTAGCAA

GTTCTCGCTGTATATGTACAACCATGGAGATGCTACTCTCTCACAACTCCCACTGTCTGCTTCACAATCCAATCAACTCATCAATTACCAGATGCAGCAT

GGTTTTGGTCAGAACATGTGTTTGGACAACATCACCAACAACAACAACTTTCAACATCCTGGCGTGTCAAACACACAAGACTACTCACCGTTACTTTCGG

CGAATAACTACGGGTTGAATAATCACTTGATGCAGCAACAGGATCAGCTTCATGGTTTTGATCAGAACTTGTGTATGATGAGTGAAATCATCAATAACAA

CAACGGTTTACAACATCCTAATCTCTCAAACACTGTTCCACATGAATTCCCTTATGGTAACACTAGCTTCTCTCAAGATATGTTTTCGAGCTATGATGGG

AGCAGTTTGCTACAGACATCTTCTCTGCCACCTCTCCACAACATTCCTAACAGCTATTGTTTTTCTGACAACTCAAGACTTCTCTGCTAA

>AT4G11880.1 | Symbols: AGL14, XAL2 | XAANTAL2, AGAMOUS-like 14 | chr4:7143512-7147108 FORWARD LENGTH=666

ATGGTGAGGGGAAAGACAGAGATGAAGAGGATAGAGAACGCAACGAGCAGGCAAGTGACTTTCTCAAAAAGAAGAAATGGACTTTTGAAGAAAGCTTTTG

AATTATCAGTCCTTTGTGATGCTGAAGTTGCCCTAATCATCTTCTCTCCTAGAGGCAAACTCTATGAGTTCTCTAGCTCCTCCAGTATACCTAAAACAGT

AGAAAGATATCAAAAGCGAATACAAGATCTCGGGTCTAACCATAAGAGAAATGATAATTCACAGCAATCGAAGGACGAAACCTATGGCTTGGCGAGAAAG

ATCGAACATTTGGAGATTTCGACACGAAAAATGATGGGAGAAGGACTTGACGCATCTTCTATAGAAGAGTTACAACAATTGGAGAACCAGTTGGACAGAA

GCTTAATGAAAATAAGAGCCAAAAAGTACCAGTTATTACGAGAAGAAACTGAGAAATTAAAAGAAAAGGAGAGGAACCTCATTGCAGAAAATAAAATGCT

GATGGAGAAGTGTGAGATGCAAGGAAGAGGAATAATAGGAAGAATATCATCATCATCATCAACATCAGAACTGGATATAGATGACAATGAAATGGAAGTG

GTGACTGATTTGTTCATTGGACCTCCTGAGACTCGACACTTCAAAAAGTTTCCTCCTTCAAACTAA

>AT4G18960.1 | Symbols: AG | AGAMOUS | chr4:10383917-10388272 FORWARD LENGTH=759

ACGGCGTACCAATCGGAGCTAGGAGGAGATTCCTCTCCCTTGAGGAAATCTGGGAGAGGAAAGATCGAAATCAAACGGATCGAGAACACAACGAATCGTC

AAGTCACTTTTTGCAAACGTAGAAATGGTTTGCTCAAGAAAGCTTACGAGCTCTCTGTTCTTTGTGATGCTGAAGTCGCACTCATCGTCTTCTCTAGCCG

TGGTCGTCTCTATGAGTACTCTAACAACAGTGTAAAAGGGACTATTGAGAGGTACAAGAAGGCAATATCGGACAATTCTAACACCGGATCGGTGGCAGAA

ATTAATGCACAGTATTATCAACAAGAATCAGCCAAATTGCGTCAACAAATAATCAGCATACAAAACTCCAACAGGCAATTGATGGGTGAGACGATAGGGT

CAATGTCTCCCAAAGAGCTCAGGAACTTGGAAGGCAGATTAGAGAGAAGTATTACCCGAATCCGATCCAAGAAGAATGAGCTCTTATTTTCTGAAATCGA

CTACATGCAGAAAAGAGAAGTTGATTTGCATAACGATAACCAGATTCTTCGTGCAAAGATAGCTGAAAATGAGAGGAACAATCCGAGTATAAGTCTAATG

CCAGGAGGATCTAACTACGAGCAGCTTATGCCACCACCTCAAACGCAATCTCAACCGTTTGATTCACGGAATTATTTCCAAGTCGCGGCATTGCAACCTA

ACAATCACCATTACTCATCCGCGGGTCGCCAAGACCAAACCGCTCTCCAGTTAGTGTAA

>AT4G22950.1 | Symbols: GL19, AGL19 | AGAMOUS-like 19 | chr4:12023946-12027421 REVERSE LENGTH=660

ATGGTGAGGGGCAAAACGGAGATGAAGAGGATAGAGAACGCAACAAGCAGGCAAGTGACGTTTTCGAAGAGAAGAAATGGACTCTTGAAGAAAGCCTTCG

AATTATCGGTCCTTTGTGATGCTGAAGTTGCTTTGGTTATCTTCTCTCCAAGATCCAAACTCTATGAGTTCTCTAGCTCTAGTATAGCAGCAACAATTGA

ACGCTATCAGAGACGAATAAAAGAAATTGGGAATAATCATAAGAGAAATGATAATTCTCAGCAAGCGAGAGACGAAACATCTGGATTGACAAAAAAGATT

GAACAGCTAGAGATATCTAAACGAAAATTGCTTGGAGAAGGCATTGATGCATGTTCCATCGAGGAGCTGCAACAGTTAGAGAATCAGTTGGACCGAAGCT

TGAGCAGGATAAGAGCCAAGAAGTACCAATTACTCCGTGAAGAAATTGAGAAGTTGAAGGCAGAGGAGAGGAATCTCGTTAAGGAAAATAAAGATCTGAA

GGAGAAGTGGCTTGGAATGGGAACAGCAACAATAGCATCATCACAATCAACGTTATCATCATCAGAAGTGAACATAGATGACAATATGGAAGTGGAGACT

GGTTTGTTCATTGGACCTCCTGAGACAAGACAATCCAAAAAATTCCCTCCTCAAAATTAA

>AT4G24540.1 | Symbols: AGL24 | AGAMOUS-like 24 | chr4:12671160-12673645 REVERSE LENGTH=663

ATGGCGAGAGAGAAGATAAGGATAAAGAAGATTGATAACATAACAGCGAGACAAGTTACTTTCTCAAAGAGAAGAAGAGGAATCTTCAAGAAAGCCGATG

AACTTTCAGTTCTTTGCGATGCTGATGTTGCTCTCATCATCTTCTCTGCCACCGGAAAGCTCTTCGAGTTCTCCAGCTCAAGAATGAGAGACATATTGGG

AAGGTATAGTCTTCATGCAAGTAACATCAACAAATTGATGGATCCACCTTCTACTCATCTCCGGCTTGAGAATTGTAACCTCTCCAGACTAAGTAAGGAA

GTCGAAGACAAAACCAAGCAGCTACGGAAACTGAGAGGAGAGGATCTTGATGGATTGAACTTAGAAGAGTTGCAGCGGCTGGAGAAACTACTTGAATCCG

GACTTAGCCGTGTGTCTGAAAAGAAGGGCGAGTGTGTGATGAGCCAAATTTTCTCACTTGAGAAACGGGGATCGGAATTGGTGGATGAGAATAAGAGACT

GAGGGATAAACTAGAGACGTTGGAAAGGGCAAAACTGACGACGCTTAAAGAGGCTTTGGAGACAGAGTCGGTGACCACAAATGTGTCAAGCTACGACAGT

GGAACTCCCCTTGAGGATGACTCCGACACTTCCCTGAAGCTTGGGCTTCCATCTTGGGAATGA

>AT4G36590.1 | Symbols: AGL40 | agamous-like 40 | chr4:17261146-17262189 REVERSE LENGTH=747

ATGGTGAGAAGTACCAAAGGTCGTCAGAAAATAGAGATGAAAAAAATGGAAAACGAAAGCAACCTTCAGGTTACTTTCTCAAAAAGAAGATTCGGTCTTT

TCAAAAAAGCTAGTGAACTTTGCACATTAAGTGGTGCAGAGATTCTGTTGATTGTGTTCTCTCCTGGTGGGAAAGTGTTTTCTTTTGGCCATCCAAGTGT

TCAAGAACTCATTCATCGCTTTTCGAATCCTAACCATAATTCTGCCATTGTCCATCATCAGAACAACAATCTCCAACTTGTTGAAACCCGTCCGGATAGA

AATATCCAATATCTCAACAATATACTCACTGAGGTGCTGGCAAACCAGGAAAAGGAGAAACAGAAGAGAATGGTTTTGGACCTATTGAAAGAATCCAGAG

AACAAGTAGGAAACTGGTATGAAAAAGATGTGAAAGATCTCGACATGAATGAAACCAACCAGCTGATATCTGCTCTTCAAGATGTGAAAAAGAAACTGGT

AAGAGAAATGTCTCAATATTCTCAAGTAAATGTTTCGCAGAATTACTTTGGTCAAAGTTCTGGCGTGATTGGTGGTGGTAATGTTGGCATTGATCTTTTT

GATCAAAGAAGAAATGCATTCAACTATAATCCAAACATGGTGTTTCCCAATCATACACCACCAATGTTTGGATACAACAATGATGGAGTTCTCGTTCCGA

TATCCAACATGAACTACATGTCAAGTTACAACTTCAACCAGAGCTAG

>AT4G37940.1 | Symbols: AGL21 | AGAMOUS-like 21 | chr4:17835695-17838621 REVERSE LENGTH=687

ATGGGAAGAGGGAAGATTGTGATCCAAAGGATCGATGATTCAACGAGTAGACAAGTCACTTTCTCCAAACGAAGAAAGGGCCTTATCAAGAAAGCCAAAG

AGCTAGCTATTCTCTGTGATGCCGAGGTCGGTCTCATCATCTTCTCTAGCACCGGAAAGCTCTATGACTTTGCAAGCTCCAGCATGAAGTCGGTTATTGA

TAGATACAACAAGAGCAAGATCGAGCAACAACAACTATTGAACCCCGCATCAGAAGTCAAGTTTTGGCAGAGAGAAGCTGCTGTTCTAAGACAAGAACTG

CATGCTTTGCAAGAAAATCATCGGCAAATGATGGGAGAACAGCTAAATGGTTTAAGTGTTAACGAGCTAAACAGTCTTGAGAATCAAATTGAGATAAGTT

TGCGTGGAATTCGTATGAGAAAGGAACAACTGTTGACTCAAGAAATCCAAGAACTAAGCCAAAAGAGGAATCTTATTCATCAGGAAAACCTCGATTTATC

TAGGAAAGTACAACGGATTCATCAAGAAAATGTGGAGCTCTACAAGAAGGCTTATATGGCAAACACAAACGGGTTTACACACCGTGAAGTAGCTGTTGCG

GATGATGAATCACACACTCAGATTCGGCTGCAACTAAGCCAGCCTGAACATTCCGATTATGACACTCCACCAAGAGCAAACGAATAA

>AT5G04640.1 | Symbols: AGL99 | AGAMOUS-like 99 | chr5:1332825-1333793 FORWARD LENGTH=969

ATGGGGGGCGTGAAGAGAAAGATTTCTATAGAGCTGATAGAGAAGAAAGATTCAAGAGCTGTTGCTTTCTCAAAACGCAGTAGGGGTCTATATAGCAAAG

CCTCTGATCTTTGTCTTCTCTCCGATGCACAAATTGCGATCATAGCCACTCCGGTCTCTTCCAAATCAAACGTTTCTTTCTACACCTTTGGCCATTCCTC

TGTTGATAACGTTGTAGCCGCTTTCCTCACGAATCAGCGTCCTCGGGAAGGTCTAGGGTTAGACTATTGGTGGGAAGATGAGAGGCTTTCAAAATCAGAG

GACCTGGAGGAACTGAGAGACGCAATGGACTCGATGTCGAAGATGTTGAAAGATCTGAAGGATTTGCAGAATCAACGAGATTGTGAGGAGGACGTGAAGA

AGAAGGGTGTTTTACACGGAACTCATCAAAAGCAAACCTTTAATCCTGAATCATGTTCTGTGAATTTCGATGGGTTTAACAAGAACACTGAAGAATTTGA

TCTTGATGAAATATTTGATTATGTGTCAACAGCTGAAGCTCTATCGATGAACTTGGACATGGACGATGTCTCTGTGGTGACCACGAATCAAAACCCCGTC

TCTGCTTCTGAAACTGTTGAAGATAGAGAATTGGTTGTTCACAAAAATATGGATGAGGACAATATCCACGTGTCTGATATGGACGACAAGGATACGATGC

TAATGATTTCTGACAAGAACAATGTTTTACCTGAAAACTTGGATGAATTCGATCAAGAGCTAGATCTTGATCAGCTACTTGATTTTGAGACAAACTATGA

AAGCCTTTTGAAGAGTTGTGAGATGGAGGATTATGCCTCGATGGTGACTACAAAGCAAAATCTGTGCTCAAATCCTGAAGCTGTTGAAGATGGAGGATTA

ATGATTCAAAAAGATTTACCGGAGGATAATCTCTGCTTTTCTGATTATTTCAGTGACTTACATTGCTGA

>AT5G06500.1 | Symbols: AGL96 | AGAMOUS-like 96 | chr5:1982444-1983172 FORWARD LENGTH=729

ATGGCTCGGAAAAAAGTACGAGCTGCATGGATCCGTGACGATAGGATGAGGAGGGCAAGCCTCAAGAGGAGGTTAACGGGGCTTATCAAAAAAGTGAATG

AACTGTCCATTCTGTGCGATATGCGCGCTAGTGTGGTCGTTTTCAATCGAGAAGAAGAACAATTGACGGCGTGGCCATCTCCCGAGGCAGCTAATTCACT

CATCGACAACTTTTATTCTCTAACCGACCATGAAAGGACCATGAAGGCCGTTGATCCGGAGTCATACGTCCAGACCGTTATCGAAAAGATTGAGAAGAAA

CGAGCCGATACTCGGAAGGTTATCACGGAGTTTGAGATGGACGAACTCATGTTCCAAGTCCAAAATGGTCGTGAACTTGCTGATCTCTCCCCAACCGAGG

CTGATAAACTAATACCATATGCGGATAAGAAACTTATGTGGTTGAGTAAACGGATGGGTTCTACGGGGGTCGACGCCCTGAGGGCTTCGAATGTTGCGTC

TGGGTCAGGTGGAAATGGGTTAAACATGATGGAAACTGGAAGAAGTTTCTATTATGTTGATAAATGGGTCTTCGTTGATCCACAAGTCCAAAACCCTTGT

GATGTTGAGACTCATTTACCCACAATGGTCAGTGGCTTAGACCTAAACATGGAACCATCTGATGAAGACTTGGGGACCTATAAAGGAGAGAGCAGCATGG

CCGGTGGTGCTGAAGACGATGCCGAATGA

>AT5G10140.1 | Symbols: FLC, RSB6, AGL25, FLF | AGAMOUS-like 25, FLOWERING LOCUS F, REDUCED STEM BRANCHING 6, FLOWERING LOCUS C | chr5:3173724-3179339 REVERSE LENGTH=591

ATGGGAAGAAAAAAACTAGAAATCAAGCGAATTGAGAACAAAAGTAGCCGACAAGTCACCTTCTCCAAACGTCGCAACGGTCTCATCGAGAAAGCTCGTC

AGCTTTCTGTTCTCTGTGACGCATCCGTCGCTCTTCTCGTCGTCTCCGCCTCCGGCAAGCTCTACAGCTTCTCCTCCGGCGATAACCTGGTCAAGATCCT

TGATCGATATGGGAAACAGCATGCTGATGATCTTAAAGCCTTGGATCATCAGTCAAAAGCTCTGAACTATGGTTCACACTATGAGCTACTTGAACTTGTG

GATAGCAAGCTTGTGGGATCAAATGTCAAAAATGTGAGTATCGATGCTCTTGTTCAACTGGAGGAACACCTTGAGACTGCCCTCTCCGTGACTAGAGCCA

AGAAGACCGAACTCATGTTGAAGCTTGTTGAGAATCTTAAAGAAAAGGAGAAAATGCTGAAAGAAGAGAACCAGGTTTTGGCTAGCCAGATGGAGAATAA

TCATCATGTGGGAGCAGAAGCTGAGATGGAGATGTCACCTGCTGGACAAATCTCCGACAATCTTCCGGTGACTCTCCCACTACTTAATTAG

>AT5G13790.1 | Symbols: AGL15 | AGAMOUS-like 15 | chr5:4449128-4450802 REVERSE LENGTH=807

ATGGGTCGTGGAAAAATCGAGATAAAGAGGATCGAGAATGCGAATAGCAGACAAGTCACTTTTTCCAAGAGGCGTTCTGGGTTACTTAAGAAAGCTCGTG

AGCTCTCTGTTCTTTGTGATGCTGAAGTTGCTGTCATCGTCTTCTCTAAGTCTGGCAAGCTCTTCGAGTACTCCAGTACTGGAATGAAGCAAACACTTTC

CAGATACGGTAATCACCAGAGTTCTTCAGCTTCTAAAGCAGAGGAGGATTGTGCAGAGGTGGATATTTTAAAGGATCAACTTTCAAAGCTTCAAGAGAAA

CATTTACAACTGCAGGGCAAGGGCTTGAATCCTCTGACCTTTAAAGAGCTGCAAAGCCTTGAGCAGCAACTATATCATGCATTGATTACTGTCAGAGAGC

GAAAGGAACGATTGCTGACTAACCAACTTGAAGAATCACGCCTCAAGGAACAACGAGCAGAGTTGGAAAACGAGACCTTGCGTAGACAGGTTCAAGAACT

GAGGAGCTTTCTCCCGTCGTTCACCCACTATGTTCCATCCTACATCAAATGCTTTGCTATAGATCCAAAGAACGCTCTCATAAACCACGACAGTAAATGC

AGCCTCCAGAACACCGATTCAGACACAACTTTGCAATTAGGGTTGCCGGGAGAGGCACATGATAGAAGGACGAATGAAGGAGAAAGAGAGAGCCCGTCAA

GCGATTCAGTGACAACAAACACGAGCAGCGAAACTGCAGAAAGAGGGGATCAGTCTAGTTTAGCAAATTCTCCACCTGAAGCCAAAAGACAAAGGTTCTC

TGTTTAG

>AT5G15800.1 | Symbols: AGL2, SEP1 | SEPALLATA1, AGAMOUS-like 2 | chr5:5151594-5153767 REVERSE LENGTH=756

ATGGGAAGAGGAAGAGTAGAGCTGAAGAGGATAGAGAACAAAATCAACAGACAAGTAACGTTTGCAAAGCGTAGGAACGGTTTGTTGAAGAAAGCTTATG

AATTGTCTGTTCTCTGTGATGCTGAAGTTGCTCTCATCATCTTCTCCAACCGTGGAAAGCTCTATGAGTTTTGCAGCTCCTCAAACATGCTCAAGACACT

TGATCGGTACCAGAAATGCAGCTATGGATCCATTGAAGTCAACAACAAACCTGCCAAAGAACTTGAGAACAGCTACAGAGAATATCTGAAGCTTAAGGGT

AGATATGAGAACCTTCAACGTCAACAGAGAAATCTTCTTGGGGAGGATTTAGGACCTTTGAATTCAAAGGAGTTAGAGCAGCTTGAGCGTCAACTGGACG

GCTCTCTCAAGCAAGTTCGGTCCATCAAGACACAGTACATGCTTGACCAGCTCTCGGATCTTCAAAATAAAGAGCAAATGTTGCTTGAAACCAATAGAGC

TTTGGCAATGAAGCTGGATGATATGATTGGTGTGAGAAGTCATCATATGGGAGGAGGAGGAGGATGGGAAGGTGGTGAACAGAATGTTACCTACGCGCAT

CATCAAGCTCAGTCTCAGGGACTATACCAGCCTCTTGAATGCAATCCAACTCTGCAAATGGGGTATGATAATCCGGTATGCTCAGAGCAAATAACTGCGA

CAACCCAAGCTCAGGCGCAGCAGGGAAACGGTTACATCCCGGGGTGGATGCTCTGA

>AT5G20240.1 | Symbols: PI | PISTILLATA | chr5:6829203-6831208 FORWARD LENGTH=627

ATGGGTAGAGGAAAGATCGAGATAAAGAGGATAGAGAACGCAAACAACAGAGTGGTGACGTTCTCAAAGAGGAGGAATGGATTGGTGAAGAAGGCTAAAG

AGATCACAGTTCTTTGTGATGCAAAAGTTGCCCTCATAATCTTTGCAAGTAATGGTAAGATGATTGATTACTGTTGTCCTTCCATGGATCTTGGTGCTAT

GTTGGACCAATACCAGAAGTTATCTGGCAAGAAACTATGGGATGCTAAGCATGAGAACCTTAGCAATGAGATTGATAGGATCAAGAAAGAGAATGATAGC

TTACAACTGGAGCTCAGGCATTTGAAGGGAGAAGATATACAGTCTCTCAACTTGAAAAATCTGATGGCTGTCGAGCACGCCATTGAACATGGCCTCGACA

AAGTCCGAGACCACCAGATGGAGATCCTTATATCAAAGAGGAGAAATGAGAAGATGATGGCGGAGGAGCAACGGCAACTCACTTTCCAGCTGCAACAACA

GGAGATGGCTATAGCAAGCAACGCAAGAGGAATGATGATGAGAGATCATGATGGGCAGTTTGGATATAGAGTGCAACCGATTCAGCCAAATCTTCAGGAA

AAGATTATGTCTTTGGTCATCGATTGA

>AT5G23260.1 | Symbols: TT16, ABS, AGL32 | TRANSPARENT TESTA16, ARABIDOPSIS BSISTER, AGAMOUS-like 32 | chr5:7836294-7838340 FORWARD LENGTH=792

ATGAATATTGAGGAAGAGGGAGCCACTCATAAGAGGAAGAAGAGAGAGATGGGTAGAGGGAAGATAGAGATAAAGAAGATAGAGAATCAGACGGCGAGGC

AAGTGACCTTCTCCAAGAGAAGAACTGGTCTTATAAAGAAGACTCGTGAGCTCTCTATTCTCTGTGACGCTCACATCGGTCTCATCGTCTTCTCAGCCAC

CGGAAAGCTTTCCGAGTTCTGCTCCGAACAGAACAGGATGCCTCAACTCATTGACCGATACTTGCATACCAACGGATTGCGACTTCCTGATCATCATGAC

GACCAGGAGCAATTGCACCATGAGATGGAACTACTAAGAAGAGAGACATGTAACCTTGAGCTTCGTCTGCGTCCATTCCATGGACATGACTTAGCCTCCA

TTCCTCCTAATGAGCTTGACGGACTCGAGAGACAGCTAGAACATTCTGTCCTCAAAGTCCGTGAGCGTAAGCAACAGTTGGAGAATCTAAGCAGAAAGAG

GAGGATGCTAGAAGAAGATAACAACAACATGTACCGTTGGCTTCATGAGCATCGTGCAGCGATGGAGTTTCAACAAGCTGGGATAGATACCAAACCAGGG

GAGTATCAACAGTTTATAGAGCAGCTTCAGTGCTATAAACCAGGGGAGTATCAGCAGTTTCTAGAGCAGCAGCAACAACAACCAAACAGCGTTCTTCAGC

TTGCTACACTTCCTTCTGAGATTGATCCTACTTACAATCTCCAGCTTGCTCAGCCTAATCTTCAAAACGATCCAACGGCCCAGAATGATTAA

>AT5G26580.1 | Symbols: AGL34 | AGAMOUS-like-34 | chr5:9393065-9394102 REVERSE LENGTH=1038

ATGGGGATGAAGAAGGTAAAGCTATCTTTGATAGCTAATGAAATATCAAGGGAAACATCCTTCATGAAGAGGAAAAATGGGATAATGAAGAAACTCTACG

AGTTGTCAACTCTATGTGGTGTCCAAGCTTGTACTCTCATCTACAGTCCATTCATTCCGGTTCCAGAGTTTCTGGAGATGTCGCCGACAGCCCGAACAAG

GAAGATGATGAATCAAGAAACGTATCTAATGGAGAGGATTACCAAAGCAAAAGAGCAACTACAGAACCTGGTTGGTGCGAACCAAGAGTTACAGGTTAGA

CGATTTATGTTTGATTGTGTTGAAGGCAAAATGTCGCAGTATCGTTATGATGCAAAAGACCTTCAAGATTTGTTATCTTGTATAAATCTATATCTCGATC

AGCTTAACGGAAGGATCGAGATCCTTAAAGAACACGGTGACTCGTTGCCTTCCGTCTCTCCTTTTCCTACTAGAATTGGTGTTGAAGAAACTGGTGATGA

GTCGTCTTCCGACTCTCCTATTCTTGCTACAACTGGGGTTGTAGATACTCCTAATGCTACAAATCCTCGTGTTCTTGTGGCCGATACGACTCATTTTCTT

GATGCGAATGCAACTGCGGTAACTGCTCCCTTTGGATTTTCTAATCATATTCAATATAAGAATATGAATATGAGTCAAGATCTGCATCGACCGTTTCAAC

ACCTTGTTCCTACTAACTTTTGTGATTTTTTTCAAAATCAGAATATGAATCAGGTTCAATACCAGGCTCCTCCTAATGATATGTTTAATCAGATTCAACG

AGAGTTCTACAACATTAATTTGAATCAGAAATCAAATCAGTATATGAATCAACAACAACCATTCATGAATCCGATGGTGGAACAACATATGAGTCATGTT

GGAGGGCGTGAGAGCATTCCTTTCATGGACGGAAACTACTACAACTACAATCAACTACCAGTCGTTGATCATGGTTCCACAAGTTACATGCCTTCCACCA

CCGGTGTTTATGATCCTTACTTCAACAATAATCTCTAA

>AT5G26630.1 | Symbols: AGL35 | agamous-like 35 | chr5:9350815-9351471 FORWARD LENGTH=657

ATGACGAGACAGAAAGTGAAAATGACTTTCATAGAAAATGAAACAGCAAGAAAATCAACATTCAAGAAAAGAAAGAAAGGTCTTTTGAAGAAAGCTCAAG

AGTTGGGAATTCTTTGTGGCGTCCCGATCTTTGCTGTCGTCAACAGTCCGTACGAGCTGAACCCGGAGGTGTGGCCATCGAGAGAGGCTGCGAACCAGGT

GGTGTCCCAATGGAAGACGATGTCGGTGATGGACAAGACCAAGAAGATGGTGAACCAAGAGACCTTTCTCCAACAGAGGATCACCAAAGCAACCGAGTCT

TGGAAGAAGTTGCGGAAAGAGAACAAAGAGTTGGAGATGAAGAACATTATGTTTGATTGTCTTAGTGGCAAAACTTTGGTTTCTAGTATCGAAAAAACTG

AGCTTCGAGATTTTGGTTATGTCATTGAACAACAACTCAAAGATGTTAATCGTAGGATCGAGATTCTGAAAAGGAACAATGAACCATCTTCCGCCCTCGT

TCCTGTTGCTGCCCCTACAACATCTAGTGTCATGCCTGTGGTTGAGATGGGTTCTTCTTCGGTTGGATTTTACGACAAGGTTCGAGATCAAATTCAAATT

ACTTTGAATATGAAACAGACCACAAATGATTTGGATCTGAACAAGAAACAGTGGTGA

>AT5G26650.1 | Symbols: AGL36 | AGAMOUS-like 36 | chr5:9343785-9344885 FORWARD LENGTH=1101

ATGAAGAAGGTGAAGCTATCTTTGATAGCTAATGAAAGATCAAGGAAAACATCCTTCATAAAGAGGAAAGACGGGATTTTTAAGAAACTCCACGAGTTGT

CAACTCTGTGTGGTGTCCAAGCTTGTGCTCTCATCTACAGTCCATTCATACCGGTTCCAGAGTCATGGCCGTCAAGGGAAGGTGCTAAAAAGGTGGCTTC

AAGGTTTCTGGAGATGCCGCCGACAGCCCGAACCAAGAAGATGATGGATCAAGAGACTTACCTTATGGAGAGGATTACCAAAGCAAAAGAGCAACTAAAG

AACCTGGCTGCTGAGAACCGAGAGTTACAGGTTAGACGATTTATGTTTGATTGTGTTGAAGGCAAAATGTCCCAGTATCATTATGATGCAAAAGACCTTC

AAGATTTGCAATCTTGTATAAATCTATATCTCGATCAGCTTAACGGAAGGATCGAGTCCATTAAAGAAAATGGTGAGTCGTTGTTGTCTTCCGTCTCTCC

TTTTCCTACTAGAATTGGTGTTGACGAAATTGGTGATGAGTCATTTTCCGACTCTCCTATTCATGCTACAACTGGGGTTGTAGATACTCTTAATGCTACC

AATCCTCATGTTCTTACGGGCGATATGACTCCTTTTCTTGATGCGGACGCAACTGCGGTAACTGCTTCCAGTAGATTTTTTGATCATATTCCATATGAAA

ATATGAATATGAGTCAAAATCTGCATGAACCGTTTCAACACCTTGTTCCTACTAACGTTTGTGATTTTTTTCAAAATCAGAATATGAATCAGGTTCAATA

CCAGGCTCCTAATAATCTGTTTAATCAGATTCAACGAGAATTCTACAACATAAATTTGAATCTGAATTTGAATCTGAATTCGAATCAGTATCTGAATCAA

CAACAATCATTCATGAATCCGATGGTGGAACAACATATGAATCATGTTGGAGGGCGTGAAAGCATTCCTTTCGTGGACGGAAACTGCTACAACTACCATC

AACTACCATCCAATCAACTACCAGCCGTTGATCATGCTTCCACCAGTTACATGCCTTCCACCACCGGTGTCTATGATCCTTACATCAACAATAATCTCTA

A

>AT5G26880.1 | Symbols: AGL26 | AGAMOUS-like 26 | chr5:9457950-9459190 REVERSE LENGTH=792

ATGGAGAGTTGCTGTCGGAGCGTCATAGCTTCAAGGACGTTTCATCTGCGGTCAAGTGGCCGTCTATTTCCCTCTCTTAGCTTAACTCATCTCAAGGGCA

AGCTATCTCTCTCCATCAACTCGTTTTCCTCCAAGATTCAGTCTCATGCTCTCCGAGGAGTTGGTATAGGCGAGTCAGATAAGAAAAATCCCCTGCCGCG

AGGGGCCGGAGAAGGAGTCAAGGAAGATGCGAGGAGTAAGCTGCTTCATGTAGTTTTGGTCTCTCCTCAGATTCCTGGGAATACAGGTTGCATTGCAAGA

ACATGTGCTGCCTCAGCTGTTGGTCTGCATTTAGTCGGGCCATTAGGTTTTCAAGTGGATGACGCCAGAGTTAAGCGAGCTGGTTTGGATTATTGGCCCT

TTGTGGTTGTCAAAGCGCATAGCTCATGGGCTGAGTTTCAAGAATATTTCAGGCTTCAGGAGGGAGAAAAACGAATGATAGCTTTTACAAAAAGAGGAAC

AAGGATACATTCAGATTTTTCTTACCGATCAGGCGATTACCTCTTGTTCGGGTCAGAGACAAGCGGTCTACCTCCTGAAGCGCTGTCAGACTGCAATCAC

GAACCATATGGAGGGGGGACCCTACGTATTCCCATGGTAGAAACGTATGTGAGATGTCTGAATCTTTCGGTGAGTGTAGGGATTGCTCTGTATGAAGCGT

CCAGACAACTTAACTATGAGCAAATCGAGTGTGCACCTCAAGGCTGTGTGAATGGTGAAGAACCATTGTTGACAGAGGATATCTTTGCTTGA

>AT5G26950.1 | Symbols: AGL93 | AGAMOUS-like 93 | chr5:9483251-9484120 REVERSE LENGTH=870

ATGGATTCTTCAATGTCGACGAAGAAGAAAACCAAATTGTCTGTGAGAAACCAAACTTGTTTCAAGAAATCGTCCCTGTCTTCTTCTTCCACTGCAAAAA

AGACCACGAATTTGTCTATGAGAGAACAGACCATGTTTAAGAAAGCCCTCGAGCTTTCGACTCTGTGCAACATCGATGTCTGTGTCATATACTATGGTCG

TGACGGAAAACTCATCAAGACATGGCCGGATGATCAATCAAAAGTTCGAGACATGGCTGAGAGGTTTAGCAGATTACACGAGAGAGAGCGATGCAAGAAA

AGAACCAACCTTTCTCTGTTTCTACGTAAGAAGATCCTCGACGACACTAAATTGTCGGAGAAAGTCTTAGAGATGGAGGATTCGTTAGAAAGTGGTCTAC

GAGTACTACAAGATAAGCTTCTGTTACTCCAACCCGAGAAAAACCAGACCGAGTTTGGTCAGACCCGTGCGGTTTCTTCCACAACGAATCCATTGTCTCC

TCCTCCTTCGCTAATTGAGGATCATCGTCATCAACAACGGACAGAACCATTGATGAGTGGTGTGTCAAACACAGAGCAAGACCTATCGACGTCATCATTG

AGTCAAAATCAGAGCAAATTTTCTGTCTTTCTCTATAACCATGACAACTGTAGCTTCTATCAAGTACCTGACTCTGTTTCTAGCTTTGACAGTTTGACGA

GTACTGGTTTACTTGGGGAACAAGGATCTGGTCTGGGAAGTAGCTTTGATCTTCCCATGGTTTTTCCTCCTCAGATGCAGACACAAACCCCACTTGTCCC

CTTTGATCAGTTTGCGCCATGGAATCAAGCACCGTCGTTTGCAGATCCAATGATGTTCCCTTATAATTAG

>AT5G27050.1 | Symbols: AGL101 | AGAMOUS-like 101 | chr5:9520276-9520638 FORWARD LENGTH=363

ATGTTCAAGAAAGCCTTGGAGCTTTCAACTCTGTGCAACATCGAAGTCTGTGTCATATATTATGGTCGTGACGGAGAACTCTTCAAGACATGGCCGGAGG

ATGAATCCAAGGTTCGAGACATGGCAGAGAGGTTTACCAAACTAAACGAGAGAGAGCGACGGAAGAAAAGAACCAATCTTTCTCTGTTTCTACGTAAGAA

GATCCTCGACGACAATAAGCTCTCAGGGAAAGTCTTAGAGATGAAGGATTCGTTAGAAAGGGGTCTACGGGTGCTACAAGATAAGCTTCTGTTACTACAA

CCCGAGAATCAGACCAAGTCCCTGACTCGTTCTGTTTCTAGTTTAGATTATGTGTTCGTTTAA

>AT5G27070.1 | Symbols: AGL53 | AGAMOUS-like 53 | chr5:9527741-9528604 FORWARD LENGTH=864

ATGGATTCTTCAATGTCGACGAAGAAGAAAACCAAATTGTCTGTGAGAAACCAAACTTGTTTCAAGAAATCGTCCCTGTCTTCTTCTTCTACTGCAAAAA

AGACCACGAATTTGTCTATGAGAGAACAGACCATGTTCAAGAAAGCCTTGGAGCTTTCAACTCTGTGCAACATCGATGTCTGTGTCATATACTATGGTCG

TGACGGAAAACTCATCAAGACATGGCCGGAGGATCAATCAAAAGTTCGAGACATGGCTGAGAGGTTTAGCAGATTACACGAGAGAGAGCGATGCAAGAAA

AGAACCAACCTTTCTCTGTTTCTACGTAAGAAGATCCTCGACGACACTAAATTGTCGGAGAAAGTCTTAGAGATGGAGGATTCGTTAGAAAGTGGTCTAC

GAGTACTACAAGATAAGCTTCTGTTACTCCAACCCGAGAAAAACCAGACCGAGTTTGGTCAGACCCGTGCGGTTTCTTCCACAACGAATCCATTGTCTCC

TCCTCCTTCGCTAATTGAGGATCATCGTCATCAACAATGGACAGAACCATTGATGAGTGGTGTGTCAAACACAGAGCAAGACCTATCGACGTCATCATTG

AGTCAAAATCAGAGCAGAATTTCAGTCTTTCTCTATAACCATGACAACCGTAGCTTCTATCAAGTCCCTGACTCAGTTTCTAGCTTTGACCAATCGGCTT

TACTTGGGGAACAAGGATCTGGTCTAGGAAGTAACTTTGATCTTCCTCCCATGGTTTTTCCTCCTCAGATGCAGACACAAACCCCACTTGTCCCCTTTGA

TCAGTTTGCGGCATGGAATCAAGCACCGTCGTTTGCAGATCCAATGATGTTCCCTTATAATTAG

>AT5G27090.1 | Symbols: AGL54 | AGAMOUS-like 54 | chr5:9531845-9532408 FORWARD LENGTH=564

ATGGATTCTTCAACGTCTACGAAGAAGAATACCAAATTGTTTGTAAGAAACCAAACTTGTTTCAAGAAATCGTCTCTGTCTTCTTCCAATGCAAAAAAGA

CCACGAATTTGTCGATGAGAGAACAGACCATGTTTAAGAAAGCCTTGGAGCTTTCGACTCTGTGCGACATCGAAGTCTGTGTCATATACTATGGTCGTGA

CGGAAAACTCATCAAGACATGGCCGGAAGATCAATCTAAGGTTCGAGACATGGCAGAGAGGTTTAGCAGATTACACGAGAGAGAACGATGCAAAAAAAGA

ACCAACCTTTCTCTGTTTCTACGTAAGCAGATCCTCCACGACAAGAAATTGTCGGAGAAAGTCTTAGAGATGGAGGATTCGTTAGAAAGTGGTCTACGGG

TACTACAAGATAAGCTTCTGTTACTCCAACCCGAGAAAAACCAGACCGAGTTAGGTCAGAGCTGTGCGGTTTATTCCACAACGTATCCATTGTCTTCTCC

TTCGCTGATTGAGGATCATCAACATCAACAACAATGGACAGAACCATTGTCAAACACAGAGTAA

>AT5G27130.1 | Symbols: AGL39 | AGAMOUS-like 39 | chr5:9546633-9547553 FORWARD LENGTH=921

ATGCCTTCATCTGATTCCACGATGATGAAGAAAGGAACAAAGCGCAAAATCGAAATAAAGAAACGAGAAACCAAAGAGCAACGAGCCGTGACTTGCTCTA

AACGCCGTCAAACTGTTTTTTCCAAAGCCGCCGATCTCTGTCTTATCTCCGGTGCTAACATCGCCGTCTTCGTAACCTCTCCCTCCGATAGTTCCGATGT

TGTCTATTCCTTCTCCGGTTACTCCTCTGCCTACGAAATCGCTGATTGTTACCTAAATCGCAAGCCTCCACCCAAGATTGTTAACCCGGCCGGATCCAAA

CTAGGATTTTGGTGGGAAGACCCTGATCTCTACCATTCGTGTGATGATCTCTCTGAGTTAAGCATTATCGAGGATCGTTTACAGAGAATGAAGAAGCATG

TGATGGCTTGCCTTGAGAAGGAAGAAAAATCTCAACTTGTTTCTAGTTTCGACCAAAACCCTAATAGTACTTGTTCTCTCGACGTCGAGGATTGTGATGG

ATCTTCTTATTCGCAAATTGCTTCTACTTTTACTCCAAACTCTGTCAACGAGTATTGCAGCGATCAAACCTTTTCTTCTTTCCATGGCGATCAAAACCCT

AACCTCTCGTCTCCGTCTTTTGATCAGGACTGTTACTCCTCTCTCTATCAGATCTGTGGAGAATCATCTTCACAAGTTGCTTCTTTTGATCAAAACCCTA

GCTCCGAGATCCAAGGATTTGAAACAGAGGAAGAGATTAACCAAATCAATCTACTACTACAAGAAACACAAACAGAGGCCAATGTCAATTTGGATGATGA

GATCTGCTTTTGGAATGATCTATCCAACGATGATGTGTTTGGTCTAAACAGCTACTTCGGTCTCGATAACACCAATGCAATGATCAACTTCGGAGATTCC

GACTTTAGACGTCATGTCTGA

>AT5G27580.1 | Symbols: AGL89 | AGAMOUS-like 89 | chr5:9736651-9737322 FORWARD LENGTH=672

ATGGATTCTTCAATGTCGACGAAGAAGAAAACCAAATTGTCTGTGAGAAACCAAACTTGTTTCAAGAAATCGTCTCTTTCTTCTTCTTCTACTGCAAAAA

AGACCACGAATTTGTCTATGAGAGAAGAGACCATGTTTAAGAAAGCCTTGGAGCTTTCAACTCTGTGCGACATCGAAGTCTGTGTCATATATTATGGTCG

TGACGGAGAACTCATCAAGACATGGCCGGAAGATCAATCCAAGGTTCGAGACATGGCTGAGAGGTTTAGCAAACTACACGAGAGAGAGCGACGCAAGAAA

AGAACAAACCTTTCTCTGTTTCTACGTAAAAAGATCCTCGACGACAATAAATTGTCGGAGAAAGTCTTAGAGATGAAGGATTCGCTAGAAAGTGGTCTAC

GGGTATTACAAGATAAGCTTCTGTTACTCCAACCCGAGAACCAGACCGAGCTAGGTCAGAGCCGTGCGGTTTCTTCCACAACGAATCCATTGTCTTCTCC

TGAGGATCATCATCATCAACAATGGACAGAACCATTGGTGACTGGTGTGTCAAACACAGAGCAAGACCTATCGACGTCACCATTGAGTAACCATCAGAGC

AAATATTCAGTCTTTGTCTATAACCATGACAGCGGAAGCTTCTATCAAGTCCCTGACTCGATCTGTTTCTAG

>AT5G27810.1 | Symbols: no symbol available | no full name available | chr5:9855827-9856186 FORWARD LENGTH=360

ATGAAGAAAGTACATGAACTCTCAACTCTATGTGGCATCACATCGTGTGCGATCATCTACAGTCCGTATGATACCAGTCATGAAGTGTGGCCATCAAATT

CTGGTGTGCAAAGAGTGGTTTCCGAATTTAGGACACTCCCGGAAATGGATCAACACAAAAAGATGGTAGATCAAGAAGGTTTTCTCAAACAAAGGATCGC

GAAACCGACAGAGAATTTGAGGAGACAAAGAAAGGACAATAAGGAGCTCGAGATGACTGAAGTCATGTTTCGATGCTTAATTGGAAACATGGAGATGCTT

AAATCAGAATCACAATCAGAATCAACAACAATGGTTTATGAAAATGATGAACCATCTTGA

>AT5G27944.1 | Symbols: no symbol available | no full name available | chr5:9975918-9976592 REVERSE LENGTH=675

ATGTATTCTTCAATGTCGACGAAGAAGAAAACCAAATTGTCTGTGAGAAACCAAACTTGCTTCAAGAAATCGTCTCTGTCTTCTTCTTCCACTGCAAAAA

AGACCACGAATTTGTCTATGAGAGAACAGACCATGTTTAAGAAAGCCTTGGAGCTTTCGACTCTCTGCGACATCGAAGTCTGTGTCATACTTTATGGTCG

TGACGGAGAACTCATCAAGACATGGCCGGAAGATCAATCAAAAGTTCGAGACATGGCTGAGAGGTTTAGCAGATTACACGAGAGAGAGCGATGCAAGAAA

AGGACAAACCTTTCTCTGTTTCTACGTAAGAAGATCCTCGACGACAATAAATTGTCTGAGAAAGTCTTAGAGATGGAGGATTCGTTAGAAAGTGGTCTAC

GGGTACTGCAAAATAAGCTTCTGTTACTCCAACCCGAGAAAAACCAGACCAAGTTAGGTCAGAGCCGTGCGGTTTCTTCCACAACGAATCTATTGTCTTC

TCCTGAGAATCACCATAATCAACAATGGACAGAACCATTGGAGAATGGTGTGTCAAACACAGAGCAAGAGCTATCGACTTCATCATTGAGTCAACATCAG

AGCAAATATTCTGTCTTTCTTTATAACCATGACAACGGAAGCTTCTATCAAGTCCCTGACTCGATCTGTTTCTAG

>AT5G27960.1 | Symbols: AGL90 | AGAMOUS-like 90 | chr5:9991685-9992770 REVERSE LENGTH=963

ATGAAGAAGGTAAAGCTATCTTTGATAGCTAATGAAAGATCAAGGAAAACATCCTTCATGAAGAGGAAAAACGGGATATTCAAGAAACTCCACGAGTTGT

CAACTCTATGTGGTGTCCAAGCTTGTGCTCTCATCTATAGTCCATTCATACCGGTTCCAGAGTCATGGCCGTCAAGGGAAGGTGCTAAAAAGGTAGCTTC

AAAGTTTCTGGAGATGCCGCGGACAGCCCGAACCAGGAAGATGATGGATCAAGAAACCCATCTTATGGAGAGGATTACCAAAGCAAAAGAGCAACTAAAG

AATTTGGCTGCTGAGAACCGAGAATTACAGGTTAGACGATTTATGTTTGATTGTGTTGAAGGCAAAATGTCCCAGTATCGTTATGATGCAAAAGACCTTC

AAGATTTGCTATCTTGTATGAATCTATATCTCGATCAGCTTAACGGAAGGATCGAGTCCATTAAAGAAAACGGTGAGTCGTTGTTGTCTTCCGTCTCTCC

TTTTCCTACTAGAATTGGTGTTGACGAAATTGGTGATGAGTCGTTTTCCGACTCTCCTATTCATTCTACAACTAGGGTTGTAGATACTCCTAATGCTACC

AATCCTCATGTTCTTGCGGGCGATATGACTCCTTTTCTTGATGCGGACGCAAATGCGAATATGAATCAGGTTCAATACCAGGCTCCTAATAATCTGTTTA

ATCAGATTCAACGAGAATTCTACAACATAAATTTGAATCTGAATTTGAATCTGAATTCAAATCAGTATCTGAATCAACAACAATCATTCATGAATCCGAT

GGTGGAACAACATATGAATCATGTTGGAGGGCGTGAAAGCATTCCTTTCGTGGACAGAAACTACTACAACTACAATCAACTACCAGCCGTTGATCTTGCT

TCCACCAGTTACATGCCTTCAACCACCGATGTTTATGATCCTTACATCAACAACAATCTCTAA

>AT5G37415.1 | Symbols: AGL105 | AGAMOUS-like 105 | chr5:14839047-14840301 REVERSE LENGTH=951

ATGAGAGACCCAGATATTGTACAAAATACGCGATCTTTGATATTGTTGAGAGTCTTGACGCATGAAAAGTTGATTCAAAGTCATCGCTATTTCTTCGTTG

GTTGGATTCACAAGAATCTCTCTCGCTTTCGACGGAAGATTCGAAGAAGATTCCTCCATTGCGATTTCTCAGGATTCGGATTTTTTCCGAGAAGATTTCT

TTTCGGTTTTGTTCTGGAGAAGATGGACCATCTTCAAGAAAGCTTCAGAGGCTCAGAGCTTTGCATACTCTGTGATATCGAAGCCTGCGTCATCTATTAC

GGACCGGACGGAGAACTCAAGACATGGCCTAAGGAGCGAGAGAAAGTGGAAGACATTGCTCTCAGGTATAGTCAATTAAACGAAGCCTTGAGAAGGAAGA

AAAGTGTTACTCTTTATGACTTCCTGAACAAGAAGAAGGACAAGACGAATTTGGAGAAGAAAGCAATGATAACGGACAATGATGACTTGAAGACTTGTTT

GAAGAACGTGAATGTTTTAAAGTCTCCAATCGCTGATCATTACTTTAATGACCAAATTTCTCAACTGATTCAGTCCTTAGAACCTCATGTCTCTAAAGTC

CAAGAAAGGATTCGTTTTGTTGAGTCGCAGAAACATAAGGAGACAAAACTGGATCATCAGAGTTTAGCATCAATATATTCTCTGAACCAATCTTTGAACC

CTAGCCAGTTCACGCTGTTTTTGTACAACCATGGAGACAATACTATGTCTCAGATCCCAAACATGTTTATGAACAACAACAACTTCCAACATTCTTTTGT

CTCAAACACACAAGATTACTCTGCTCTCCAAGAATCGGTGAATAACAACTATGGGTTGATGCCGAATGTTCTTTGTGGTTATGATCAGAACCTGTTCACG

AGTGATATTACCAACAACAACCTTCTTATCGACAACTCGATGTATCTCTGA

>AT5G38620.1 | Symbols: AGL73 | agamous-like 73 | chr5:15463858-15464907 REVERSE LENGTH=1050

ATGGTGAAAGGCACGAAGAGAAAGATTGCGATTGAGACGATACAGAAGAGAGATTCCCTTAGGGTTACCTGCACCAAACGCCGTAAAGGTCTCTATAGCA

AAGCCTCTCAGCTTTGTCTTCTCTCCGACGCACAAATCGCTATCTTAGCGACTCCTCCTTCTTCTGAATCCGACGTCTCTTTCTACTCTTTCGGTCACTC

CTCTGTTGATGCCGTTGTCTCTGCTTTTCTCTCCGGAAAACGTCCTGTTTCGGCTCCGAAGGATAACAAAGAGACGAGGGAGGACGTTGGTATCTGTTTG

ACTCGCAAGAATCTAGGGTTAGGGTTTTGGTGGAATGACGAGAGCCTTGTGAGATCGGAGAATCCTCAAGAGATTAGTGAAGCGATCGGTTCCATGTGGA

CTCTGTTGAGTAATCTCAAGGAGTTGCGTGCGGATGAAGCTTGCGTTAACGATCACAAGGACTTGAAGAAGAATGAGAAGAGCGATGTTCACGGAACTCA

AGATCAAACCCTAATTTTTCAGTCCGCTTCTGCAGTTTGTTGCATCCCTGAAAATCTCAACGATATTACTCAAGAGCCAAATCAAACCCTAGATATTCAG

TCCAGTAGTTCTGCAATCTGTTGCGTCCCTGATAAATCACCTGAAATTTTCAATGAAATCACTGAAGAACAAGATCAGATTCTCTCGATTTGTGAAACTT

TTTGTGTTACGGATAATAACAACAACAACAACAACAACGCTGCTTTACCTGAAGTTAACCTATATTACAATCAAGACATGGCTATTGATCAACTCATTGA

CTTCAACACCCCTTTTGAAAGCTCGATAGATGATTGGTTTTCGGACAACACTACTCATCAGGAGACGACTAGTGCAAGTATTTTGAACGATGTTGGTGTT

GATGATCAAGTCTCGGTGGATACAAATCCGTTTTCTTATTTTCAAAGCCTTGAAGATGCAGATTTGGTGTTTCAGAGATGTTTGGATGGAGATAATCTAC

GATTCTCTGATTGTTTCAATGACTTTGCAAACACTATTGCAGCAGTTTGA

>AT5G38740.1 | Symbols: AGL77 | AGAMOUS-like 77 | chr5:15513025-15514305 REVERSE LENGTH=1281

ATGACGACCATTCGATCTTCACCTTCTTCCTCTCGTTGTTCCAATTCGTCTTCCTCTTCTTCTTATTCACTCGCTTCCACAAGTTTGTCTAACAGACTTG

AGACTATATTCAAGAAAGCTTCAGAGCTTTGCACTCTCTGTGATATCGAAGCCTGCGTCATCTATTACGGACCAGACGGAGAACTTAAAACATGGCCTAA

GGAGCGAGAGAAAGTGCGAGACATTGCTCTGAGGTTTAATCAATTAAACGAAGCCTTGAGACACAAGAAAAGCGTTAATCTTCATGGGTTCCTGAACAAG

AAGAAGAAGAACAAGGGTTTGAAGAATCCGAACAAGAAGAAGAAGACGAGTCTTAAGAACGTGAATGTTTTAAAGTATCCACTCGCTGATCATTACTCTC

CCGACCAAGTTTCTCAATTGACTCAGTCCTTAGAACTCAATGTCTCTAAATTCCAAGAAAGGCTTCGATTTCTTGAGTCGCAGAAACAGAATGAGACAAA

ACCGGATCATCAGAGTTTAACATCAATATCTTCTCTGAACCAATCTTTGAACCCTAGCCAGTTCTCGCTGTTTATGTATAACCATGGATATAATACTCTG

TCTCAGATCCCAGTCTCTGCATCAAATTTCAATCAGGATTATATCTCAGCGTTACTTGAACAATCTGAGTTGAAGAGTCAGATAATGAAGCAGGAGGTTT

GTGGTTATGAGCAGAACATGTGCATGAGTAACCATGGAGATGCTACGCTCTCTCAAATCCCATTCTCTGCATCAAATTTCAATCAAGATTTTTCAGCCAA

CAACAACTTCCAACATTCTTTTGTCTCAAACACACAAGATTACTACTCTGTTCAAAAATCTGTGAATAACAACTATGGGTTGAAGAATCAGTTAATGAAG

CACGATCTTTGTGGTTATGAGCACAACATGTGCATGAGTAACCATGGAGATGCTACCTTCTCTCAAATCCCACTCTCTGCATCAAATTTCAATCAAGATT

TTTCAGTCTCAATACAAGAAGAATCTGGCTTGATGCAGCAGGAGCTTTGTGGTTATGATCAGAATCAGAACATGAGCATGGGTGACATCACAAACAACAA

TTTTCAAGTTACTTGCGCCTCAGTACTAGAATCTGTGAACAACTTTGGGTTGAATCAGTTGATGCACAAGGAGTTTTATGGTTGTCATCAAAACATGTCT

ATGGGTAACATCAATAACAACAGCTTTCAACATCCTTGGGTCTCAAACGCAGACCATACTCGGCGGTACAAGAATCTGTGA

>AT5G39750.1 | Symbols: EMB3008, AGL81 | EMBRYO DEFECTIVE 3008, AGAMOUS-like 81 | chr5:15906875-15907942 FORWARD LENGTH=1068

ATGGCCATTCGATCTTTACCTTCTTCCTCTCGCTGTTCCTCTTCTTCTTCTTCTTCTTCTTATTCACTCGCTTCAACGAGTCTAAGCAATAGACTTGAGA

CCATCTTCAAGAAAGCTTCAGAGCTTTGCACTCTCTGTGATATCGAAGCATGCGTCATCTATTACGGACCAGACGGAGAACTCAAGACATGGCCTCCGGA

GCGAGAGAAAGTGGAAGACATCGCTCTCAGGTATAGTCAATTAAACGAAGCCTTGAGAAGGAAGAAAAGTGTTACTCTTTATGACTTCCTGAACAAGAAG

AAGGACAAGACGAACTTGGAGAAGAAAGCAAAGATAACGGACAATGATGACTTGAAGACTTGTCTGAAGAACGTGAATATTTTAAAGTATCCACTCGCTG

ATCATTACTCTCCCGACCAAGTTTCTCAACTGATTCAGTCCTTAGAACCTCATGTCTCTAAAGTCCGAGAAAGGATTCGTTTTGTTGAGTCGCAGAAACA

CAAGGAGACAAAACCGGATCATCAGAGTTTAGCATCATCCTCTCTGAATCATCAGACCCAATCTTTGAACCCTAGCCAGTTTTCGCTGTTTATGTATAAC

CATGGAGACAATACTCTGTCTCAGATCCCAGTCTCTGCATCAAATTTCAATCAGGATTATTTCTCAGCGTTACTTGAACAATCTGAGTTGAAGAGTCAGA

TAATGAAGCAGGATCTTTGTGGTTATGAGCAGAACATGTGCATGAGTAACCATGGAGATGCTACGCTCTCTCAAATCCCACTCTCTGCATCAAATCTCAA

TCAAGATTTCTCAGCCTTACTACAAGACGAATCTGGCTTGATGCAACAGGAGCTTTGTGGTTATGATCAGAACATGTTTATGAACAACAACAACTTTCAA

CATTCTTTTGTCTCAAACACACAAGATCACTCTGCTCCTGTGGTACAAGAATCTGTGAACAACAACTATGGGTTGATGCCGCATGTTCCTTGTGGTTATG

ATCAGAACCTGTTCACGAGTGATATTACCAACAACAACCTTCTTATCAACAACTCGATGTTTCTCTGA

>AT5G39810.1 | Symbols: AGL98 | AGAMOUS-like 98 | chr5:15937278-15938344 REVERSE LENGTH=990

ATGGCCATTCGATCTTTACCTTCTTCCTCAGGCTGTTCCAATTCCTCTTCGTCTTCTTCTTATTCACTCGCTTCAACAAGTTTGAGCAATAGACTTGAGA

CCATCTTCAGGAAAGCTTCAGAGCTTTGCACTCTCTGTGATATCGAAGCATGCGTCATCTATTACGGACCAGACGGAGAACTCAAGACATGGCCTCCGGA

GCGAGAGAAAGTGGAAGACATTGCTCTCAGGTATAGTCAACTAAACGAAGCCTTGAGACGCAAGAAGAGCGTTACTCTTTATGACTTCCTGAACAAGAAG

AAGAACAAGACGAACTTGGAGAAGAAAGCAAAGATAAAGGACAACGACTTGAAAAGACTTTCCTTAGAACCTCATGTCTCTAAAGTCCGAGAAAGGATTC

GTTTTGTTGAGTCGCAGAAACATAAGGAGACAAAACCGGATCATCAGAGTTTAGCATCATCCTCTCTGAATAATCAGACCCAATCTTTGAACCCTAGCCA

GTTTTCGCTGTTTATGTATAACCATGGAGATAATATTCTGTCTCAGATCCCAGTCTCTGCATCAAATTTCAATCAGGATTATTTCTCAGCGTTACTTGAA

CAATCTGAGTTGAAGAGTCAGATAATGAAGCAGGAGGTTTGTGGTTATGAGCAGAACATGTGCATGAGTAACAATGGAGATGCTACGCTCTCTCAAATCC

CACTCTCTGCATCAAATTTCAATCAAGAGTTCTCAGCCTTACTACAAGAAGAATCTGGCTTGATGCAACAGGAGCTTTGTAATTATGATCAGAACATGTT

TATGAACAACAACAACTTCCAACATTCTTTTGTCTCAAACACACAAGATCACTCTGCTCCTGCGGTACAAGAATCTGTGAACAACAACTATGGGTTGATG

CCGCATGTTCCTTGTGGTTATGATCAGAACCTGTTTACGAGTGATATTACCAACAACAACCTTCTTATCGACAACTCGATGTTTCTCTGA

>AT5G40120.1 | Symbols: AGL76 | AGAMOUS-like 76 | chr5:16051879-16053036 FORWARD LENGTH=1158

ATGACTATGCGATCATTACCTTTTTCCTCTTCTTCTTATTCACTCGCTTCCACAAGTTTGAGTAACAGACTTGAGACTATATTCAAGAAAGCTTCAGAGC

TTTGCACTCTCTGTGATATCGAAGCGTGCGTCATCTATTACGGACCAGACGGAGAACTTAAGACATGGCCTAAGGAGAAAGAGAAAGTGAGAGACATTGC

TCTGAGGTATAGTCAATTAAACGAAGCCTTGAGAAGCAAGAAAAGCGTTAATCTTCATGGGTTCCTGAACAAGAAGAAGAAGAAGAAGAAGAAGGGTTTG

AAGAATCCGAACAATAAGAGGAAGACTTGTCTTAAGAAGAACGTGAATGTTTTAAAGTATCCACTCGCTGATCATTACCCTCCCGACCAAGTTTCTCAAC

TGACTCAGTCCTTAAAACTCCATGTCTCTAAATTCCAAGAAAGGCTTCGATTTCTTGAGTCGCAGAAACAGACAAAACCGGATCATCAGAGTTTAACACC

ATCCTCTTTGAATCATCAGACCCAATCTTTGAACCCTAGACAGTTTTCGCTGTTTATGTATAACCATGGAGACAATACTCTGTCTCAGATCTCAGTCTCT

GCATCAAATTTCAATCAGAATTATTTCTCAGCGTTACTTGAACAATCTGAGTTGAAGAATCAGTTAATGAAGCAGGATGGTTATGATCAGAATCAGAACA

TGAGGATGGGTGACATCACCAACAACAATTTTCAACTTCCTTACTTCTCAAAGAAAGAAGCGGTACAAGAATCTGTGAACTACTTTGGGATGAATCAGTT

GATGCTGAAGGAGTTATATGGTTGTGATCAAAACATGTGTATGGGTAACATCAATAGCAACAGCTTTCAACATCCTTGCGTCTCAAAGGCACAACATTAC

TCGGCGGTAGAAGGATCTGTCAATAACCAACGACAATCTGAGTTAATGCAGCAGGAGCTTTGTGGTTATGAGCAGAACATGTGCTTTACCAACAACAATT

TTCAAGTCTCAAACAAAGAAGCGGTTCAAGAATCTGTGACTAATTTTGGGTTGATGCAGCATGAACTTTATGGGTGTGATCAAAACATGTCTATGGGCAA

CATCATTAACAACAGCTTTCAACAGCGTCTCAAACACAGAACAAGAATCTGTGAATAA

>AT5G40220.1 | Symbols: AGL43 | AGAMOUS-like 43 | chr5:16078390-16079364 REVERSE LENGTH=975

ATGACTATGCGATCATCTTTACCTTCTTCCTCTTCCGCTTATTCACTCGCTTCAACAAGTTTGAGCAACAGACTTGAGACCATCTTCAAGAAAGCTTCAG

AGCTTTGCACTCTCTGTGATATTGAAGCCTGCGTCATCTATTACGGACCAGACGGAGAACTCAAGACATGGCCTCCGGAGCGAGAGAAAGTGAGAGACAT

TGCTCTTAGGTATAGTCAATTAAACGAAGCATTGAGACGCAAGAAAAGCGTTAATCTTCATGGGTTCCTGAACAAGAAGAAGAAGAACAAGGGTTTGAAG

AATACGGACAAGAAGAGGAAGACGAGTCTTAAGAAGGTGAATGTTTTAAAGTATCCACTCGCTGATCATTACCCTCCCGACCAAGTTTCTCCACTGATTC

AGTCCTTGGAACTCCATGTCTCTAAATTCCACGAAAGGCTTGAATTTCTTGAGTCGCGGAAACAGAATGAGACACAACCGGATCACCACAGTTTAGCATC

ATCCTCTCTGAATCATCAGACCCAATCTTTGAACCCTAGCCAGTTCTCGCTGTTTATGTATAATCATGGAGACAATACTCTGTCTCAGATCCCAGTCTCT

GCATCAAATTTCAATCAGGATTATTTCTCAGCGTTACTTGAACAATCTGAGTTGAAGAATCAGTTAATGAAGCAGGAGATTTGTGGTAATGATCAGAATC

AGAACATGTGGATGGGTAACATCACCAACAACAATTTTCAACTTCCTTGCGTCTCAGTACAAGAATCTGTGAACAACTTTGGGTTGATGCACAAGGAGTT

TTATGGTTGTGATCATAACATGTCTGTGGGTAACATCAATAGCAACAGCTGTGAACATCCTTGCGTCTCAAGCACACAACATTACTCGGCGGTAGAAGAA

TCTGTGAATAACCCCTGGTTGAATCAGTTGATGCAGAATGAACTTTATGGTTACGGTTATGCAGACTTTTGTTAA

>AT5G41200.1 | Symbols: AGL75 | AGAMOUS-like 75 | chr5:16490544-16491536 FORWARD LENGTH=993

ATGACTATGCGATCATCTTCACCTTCGTCCTCTTCTTCTTATTCACTCGCTTTCACAAGTTTGAGCAATAGGCTTGAGACCATCTTCAAGAAAGCTTCAG

AGCTTTGCACTCTTTGTGATATCGAAGCCTGCGTTATCTATTACGGACCAGACGGAGAACTTAAGACATGGCCTAAGGAGAAAGAGAAAGTGAGAGACAT

TGCTCTTAGGTATAGTCTATTGAACGAAGCATTGAGACGCAAGAAAAGCGTTAATCTTCATGGGTTCCTGAACAAGAAGAAGAACAAGGGTTTGAAGAAT

CCGAACAAGAAGATGAAGACGAGTCTTAAGAACGTGAATATTTTAAAGTATCCACTCGCTGATCATTACCCTCCCGACCAAGTTTCTCCACTGATTCAGT

CCTTGGAACTCCATGTCTCTAAATTCCAAGAAAGGCTTCGATTTCTTGAGTCGCAGAAACAGAATCAGACAAAACCGGATCATCAGAGTTTAACACCATC

CTCTCTGAATCATTACACCCAATCTTTGAACCCTAGCCAGTTCTCGCTGTTTATGTATAACCATGGAGACAATACTCTGTCTCAGATCCCAGTCTCTGCA

TCAAATTTCAATCAGGATTATTTCTCAGCGTTACTTGAAGAATCTGAGTTGAAGAATCAGTTAATGAAGCCGGAGATTTGTGGTTATGATCAGAATCAGA

ACATGAGCATGGGTGACATCACAAACAACAAATTTCAAGATCCTTGCGTCTCAAACAAAGAAGCGGTACAAGAATCTGTCAACAACTTTGGGTTGAATCA

ATTGATGTACAAGGAGTTTTATGGTTGTGATCAAAACATGTCTATGGGTAACATCAATAGCAACAGCTTTCAAAATCCTTGCGTCTCAAACACACAACAT

TACTCGGCGGTAGAAGAATCTGTGAAGAACCCCTGGTTGAATCAGTTAATGCAGAATGAACTTTACGGTTACGGTTATGCAGGCTTTTGTTAA

>AT5G48670.1 | Symbols: FEM111, AGL80 | AGAMOUS-like 80 | chr5:19738825-19739790 REVERSE LENGTH=966

ATGACAAGAAAGAAAGTGAAACTTGCTTACATTTCCAACGATTCTTCAAGAAAAGCGACTTTCAAAAAAAGAAAGAAGGGTTTGATGAAGAAGGTACACG

AACTCTCGACTCTTTGTGGCATCACTGCATGTGCGATCATCTACAGCCCGTATGATACCAACCCTGAAGTGTGGCCATCAAATTCCGGTGTGCAAAGGGT

GGTTTCAGAATTTAGGACACTCCCAGAGATGGATCAACACAAAAAGATGGTAGATCAAGAGGGTTTTCTCAAACAAAGAATCGCGAAAGCGACAGAAACT

TTGAGGAGACAAAGAAAGGATAGTAGGGAGCTAGAGATGACTGAAGTCATGTTCCAATGTTTGATTGGAAACATGGAGATGTTTCATTTGAATATTGTGG

ATCTTAATGATTTGGGTTATATGATTGAGCAATATCTTAAAGATGTTAATCGCAGGATTGAGATTTTACGAAATTCTGGTACGGAGATTGGTGAATCTTC

TTCTGTTGCGGTAGCTGCTTCTGAAGGGAATATACCAATGCCGAATTTGGTCGCTACAACAGCTCCCACCACTACAATTTATGAGGTGGGTTCTTCTTCC

TCATTTGCGGCTGTCGCGAATTTTGTTAATCCTATTGATCTTCAACAATTTCGTCATCCGGCGGCTCAACATGTTGGACTTAATGAGCAACCTCAAAATC

TAAATCTGAATTTAAATCAGAACTATAATCAAAATCAAGAATGGTTTATGGAAATGATGAACCATCCTGAGCAAATGAGGTATCAGACAGAGCAAATGGG

ATATCAATTCATGGATGATAACCACCACAACCACATCCATCACCAGCCACAGGAGCACCAACATCAGATCCATGACGAATCTTCCAACGCTCTTGATGCC

GCCAACTCAAGCAGCATCATCCCTGTTACTAGTTCAAGTATTACCAATAAAACATGGTTCCATTAG

>AT5G49420.1 | Symbols: AGL84 | agamous-like 84 | chr5:20035166-20036170 REVERSE LENGTH=1005

ATGGTGAAAAAAGGTGGCACGAAGAGGAAGATTGCGATTGAGACGATACAGAAGAGAGATTCCCTTAGGGTTACCTGCACCAAACGCCGTGAAGGTCTCT

ATAGCAAAGCCTCTCAGCTTTGTCTTCTCTCAGACGCACAGATCGCTATCTTAGCGACTCCTCCTTCTTCTGAATCCAACGTATCCTTCTACTCTTTCGG

TCACTCCTCTGTTGATGCCGTTGTCTCTGCTTTTCTCTCCGGACAACGTCCTGTTCCGAAGGATAACAAAGAGACGAGGGAGGACGTTGGTATCTGTTTG

ACTCGCAATAATCTAGGGTTAGGTTTTTGGTGGAACGACGAGAGCCTTGCCAGATCGGAGAATCCTCAAGAGATTAGTGAAGCGATCGACTCTATGCGGA

CGCTGTTGAGGAATCTCAAGGAATTGCGTGCGGATGAAGCTTTAGCCTGTAATCAAGCTTTCGTTAATGATCGCGAGGACCTGAAGAACAACGACAAGTG

CGATTTTGTTTCAGACCACGAAACTCATGATCAAACCCTAATTCTTCAGTCCGCTTCTCCAATTTGTTGCATCCCAGAAAATCTCAACGAGATTACTCAA

GAGCCAAATCAAACCCTAAATATTCAGTCGAGTACTTCTGCAATTTGTTGCGTCCCTGATAATTCACCTGAAAATTTCAATGAGATCACTGAAGAACAAG

ATCAGATTCGCTCGATTTGTGAAACTTTTTGTGTTATGGATAACAACGCTGCTTTACCTGAAATGAACCTGGATTACGATCAAGACATTGGCTTCGACAC

GCCTTTTGAGAGCGCGCTAAATGATTGGTTTTCGGACAACACTACTCATCAGGAGATTAGTGCAAGTATTTTGAACGCTGTTGTTGATGATCAAGTCTCG

GTGGATCTAACTCCGTTTTCTTATTTTCAGAGATGTTTGGATGGAGATAATCTACGATTCTCTGATTGTTTCAAAGACTTTGCAAACACTATTTCAGCAC

TTTGA

>AT5G49490.1 | Symbols: AGL83 | AGAMOUS-like 83 | chr5:20075328-20076185 FORWARD LENGTH=858

ATGAGATTCGTTCCTTATTTATACGAGATTGAGAGATTATGGCTTTCACTTGTCAATTATCTTTCTCCACGCAAAAACAAAAACCGTCGCTGTGGTGAAA

TAGATAAAATCAGAATGGTGAAAAAAGGAGGTACGAAGAGGAAGATTGCGATTGAGACGATACAGAAGAGCGATTACCTTAGGGTTACTTGCACCAAACG

TCGTGAAGGTCTCTTTAGCAAAGCTTCTCAGCTTTGTCTTCTCTCCGATGCACAGATCGCTATCTTAGCGACTCCTCCTACTTCTGAATCCAACATCTCC

TTCTACTCTTTCGGTCACTCCTCCGTTGATGCCGTTGTCTCTTCTTTCCTCTCCGGGCAACGTTGTGTTCCTCTTCAGGAGGATACCAAAGAGATGAGAG

AGGACGTTGCTATTTGCTTGTCTCGTACAAATCTAGGGTTAGGGTTTTGGTGGAACAACGAGAGCCTCAACAAATCGGAGAATCCTCAAGAGATTAGTGA

CGCTATCAACTCTATGTTAACGCTCTTGAGTAATCTCAAGGAATTGAGTGGGGAGGAAGCTTTAGTTAACGATCACAAGGACTTGAAGAAGAATGAGAGG

AGCGATGTTGTTTTACAACACGGAACTCAATATGAAACCCTAAATCCTAACTCCAACACTACTACAATTTGTTGCGTCCCTGATGAATTACCTGCCAATT

CCAACGAGATTGTTGGAATCTCACCAAATCCTTTGATTATGCTAGAAAAGAAGAAATCACAAATTGAAGAAAAGTTTGAGAAGGAATGGCAAGTTTCAGT

TACTCGAATTGAAAATGAAGCCACGAGTTCTTATGCAAAACGAAGAAGAAGTATATGA

>AT5G51860.1 | Symbols: AGL72 | AGAMOUS-like 72 | chr5:21081844-21084126 REVERSE LENGTH=636

ATGGTGAGAGGAAAGATCGAAATCAAGAAGATTGAGAACGTGACAAGTAGACAAGTCACGTTTTCGAAGCGAAGGAGTGGTCTCTTTAAGAAGGCTCATG

AGCTTTCTGTTCTATGTGATGCTCAAGTGGCAGCTATGATCTTCTCTCAGAAAGGAAGATTATATGAATTCGCTAGCTCCGATATCAGGAACACGATAAA

GCGATACGCTGAGTACAAGAGAGAGTATTTTGTTGCAGAAACTCATCCTATAGAGCAATACGTGCAGGGGCTAAAGAAGGAAATGGTGACAATGGTGAAA

AAGATTGAAGTGCTTGAAGTCCATAACCGGAAGATGATGGGACAAAGTTTGGATTCTTGTTCGGTAAAAGAACTTTCAGAGATAGCCACACAGATAGAGA

AAAGCCTTCATATGGTTAGATTAAGAAAGGCTAAGTTATATGAAGATGAGCTACAGAAACTAAAAGCCAAGGAGAGGGAACTCAAGGACGAGAGAGTCAG

GCTTTCTCTAAAGAAAACAATTTATACTCACTTATGCCAGGTTGGAGAAAGACCAATGGGGATGCCGTCGGGAAGCAAAGAGAAAGAGGATGTTGAAACT

GATCTATTTATTGGATTTCTGAAGAACCGACCATAA

>AT5G51870.1 | Symbols: AGL71 | AGAMOUS-like 71 | chr5:21085635-21087923 REVERSE LENGTH=624

ATGGTGAGAGGGAAGATCGAGATCAAGAAGATTGAGAACGTGACGAGCAGACAAGTCACGTTCTCGAAGCGAAGAAGCGGTCTATTTAAGAAGGCTCATG

AGCTTTCGGTTCTATGCGATGCTCAAGTAGCCGCCATTGTCTTTTCTCAGAGCGGAAGATTACACGAATACTCTAGCTCCCAGATGGAGAAGATTATAGA

TAGATATGGCAAGTTTAGTAATGCCTTCTATGTGGCAGAGAGGCCTCAAGTAGAACGATACTTGCAGGAGCTGAAGATGGAAATTGATAGAATGGTGAAA

AAGATTGATCTCCTTGAAGTTCATCACCGTAAGCTGTTGGGGCAAGGTTTGGATTCGTGTTCAGTGACAGAACTTCAAGAGATTGACACTCAAATTGAGA

AAAGCCTTCGTATTGTCAGGTCAAGAAAGGCTGAGTTATATGCAGATCAACTGAAAAAGCTAAAAGAAAAGGAGAGGGAGCTCTTGAACGAGAGAAAAAG

GCTGCTTGAAGAGGTAAATATGCACCACTCATCAAAGGGTAATACTGAAGGTGGTCACAGAACCAAACATTCATCAGAGGTTGAAACCGACCTATTTATC

GGATTGCCCGTGACTCGGCTATAA

>AT5G55690.1 | Symbols: AGL47 | agamous-like 47 | chr5:22548790-22549623 REVERSE LENGTH=834

ATGGGTCGAAAGATGGTAAAGATGACGAGGATAACGAACGAGAAAACGAGGATAACGACTTACAAGAAGAGGAAAGCATGTTTGTATAAGAAAGCCAGTG

AGTTCTCAACACTCTGCGGTGTGGACACTTGTGTCATTGTGTACGGCCCGAGCAGAGCAGGGGACGAAATGGTCATGGAGCCCGAGTTATGGCCAAAGGA

TGGGAGCAAAGTCCGTGAAATCTTAACCAAGTACAGAGACACTGCGTCAAGCAGCTGCACCAAGACATACACCGTGCAAGAATGCTTGGAGAAAAACAAC

ACTAAGGTGGAGAAACCGACGATTGCGACAAAGTATCCTACATGGGACAAAAAGCTCGACCAGTGTTCTTTAAATGACCTCTATGCGGTTTTCATGGCAG

TAGAAAACAAGATCCAAGAGGCTACGAATAGGAATCAGACATTTCCTGACACTAGTTGTTGGTCTAATGACCAACTTGGTTTATGCGGTTACAATCGGCA

ATGTTTTGAGCAGTATCAGTTGTTTCCTCTGCCTACTATGGATTACAACGGGCTCTCTTTCTTCCCTTTTAATAACCAGATGACCTCAAATACTGCGGAA

GTGTCTTCCTTCTCGAATGTGACAGAGCCGATGATAGCGAACGGGCAAAGCTTGTTTTACGGGAGTTGTTCGGATGGTCCATATGGTCCGATGGTACAGA

GGACAGCTTATATGGAGCCAATACATTGGGGTTTAGGAAACAGTATGTTCAACAATGTGAAGCAGTTCCAAGATTATCCCTTCAGGTTTGCACAAGTTAA

TGATTTGGAGGATTCAAGTAAACTTTCTATGTGA

>AT5G58890.1 | Symbols: AGL82 | AGAMOUS-like 82 | chr5:23780832-23781716 FORWARD LENGTH=885

ATGGTTCCGAAAGTGGTCGACCTACAAAGGATAGCGAACGATAAGACAAGGATAACAACTTACAAGAAGAGGAAAGCTAGTCTTTACAAGAAGGCACAAG

AGTTCTCAACTCTCTGCGGCGTCGAGACATGTCTCATCGTCTACGGTCCCACGAAGGCTACCGATGTGGTGATTTCCGAGCCAGAGATATGGCCGAAGGA

CGAGACCAAAGTCAGGGCCATCATACGCAAGTACAAAGACACAGTGTCGACCAGCTGCAGGAAAGAAACCAACGTGGAGACTTTCGTCAACGATGTAGGG

AAAGGAAACGAGGTGGTGACTAAAAAGAGAGTGAAGCGTGAGAATAAGTATTCTAGTTGGGAGGAGAAGCTAGACAAGTGTTCACGAGAGCAACTACATG

GGATTTTCTGTGCCGTGGATAGCAAGTTAAATGAAGCTGTAACGAGACAGGAGCGTAGTATGTTTAGGGTTAATCATCAAGCCATGGACACACCATTCCC

GCAGAATTTAATGGACCAACAATTCATGCCACAGTATTTTCATGAGCAGCCACAGTTTCAAGGCTTCCCTAATAATTTCAATAATATGGGTTTCTCGTTG

ATTTCACCTCATGATGGTCAGATTCAAATGGACCCAAATCTCATGGAGAAGTGGACCGACTTGGCTTTGACTCAAAGCTTGATGATGTCAAAGGGAAACG

ATGGTACTCAATTCATGCAGAGGCAAGAACAACCATACTATAATCGTGAACAGGTTGTATCGAGGTCTGCAGGTTTCAATGTTAACCCGTTTATGGGATA

TCAAGTCCCGTTTAATATTCCTAATTGGAGATTATCGGGAAATCAAGTTGAAAATTGGGAGCTTTCAGGGAAGAAAACGATATGA

>AT5G60440.1 | Symbols: AGL62 | AGAMOUS-like 62 | chr5:24306329-24307520 FORWARD LENGTH=900

ATGGTGAAAAAAAGCAAAGGTCGTCAAAAAATAGAGATGGTCAAAATGAAAAATGAAAGTAACCTTCAAGTTACTTTTTCAAAAAGAAGATCTGGACTTT

TCAAAAAGGCTAGTGAGCTTTGCACACTTTGTGGTGCAGAAGTGGCCATAGTTGTGTTCTCACCTGGTCGAAAAGTCTTTTCTTTTGGTCATCCAAATGT

TGATTCTGTAATTGATCGATTCATAAACAATAACCCTCTACCTCCTCACCAACACAACAACATGCAACTTAGAGAAACTCGTCGAAATTCGATTGTTCAG

GATCTGAATAATCATCTTACTCAGGTGTTGAGTCAATTAGAAACAGAGAAAAAGAAGTACGACGAGTTAAAGAAAATAAGAGAAAAGACAAAAGCCCTTG

GGAATTGGTGGGAAGATCCCGTTGAGGAACTTGCGTTATCTCAACTCGAGGGGTTCAAAGGTAATCTTGAAAATTTGAAGAAAGTAGTTACAGTCGAAGC

TTCCAGATTTTTTCAGGCAAATGTTCCAAACTTCTATGTGGGAAGTTCTAGTAATAATGCTGCTTTTGGGATTGATGATGGTAGTCATATCAACCCTGAT

ATGGATCTCTTTAGCCAAAGAAGAATGATGGACATAAATGCCTTCAACTACAACCAGAACCAAATTCACCCTAATCATGCATTACCACCCTTTGGAAACA

ATGCTTATGGTATTAATGAAGGGTTTGTTCCAGAATACAATGTGAACTTCAGACCAGAGTATAACCCAAACCAAAACCAAATCCAAAACCAAAATCAAGT

TCAAATCCAAATCCAAAACCAGAGTTTTAAGAGAGAAAACATCTCTGAATATGAACATCATCATGGTTATCCTCCCCAGTCTAGATCTGATTACTATTAA

>AT5G60910.1 | Symbols: FUL, AGL8 | FRUITFULL, AGAMOUS-like 8 | chr5:24502736-24506013 REVERSE LENGTH=729

ATGGGAAGAGGTAGGGTTCAGCTGAAGAGGATAGAGAACAAGATCAATAGGCAAGTTACTTTCTCAAAGAGAAGGTCTGGTTTGCTCAAGAAAGCTCATG

AGATCTCTGTTCTCTGCGATGCTGAGGTTGCTCTCATCGTCTTCTCTTCCAAAGGCAAACTCTTCGAATATTCCACCGACTCTTGCATGGAGAGGATACT

TGAACGCTATGATCGCTATTTATATTCAGACAAACAACTTGTTGGCCGAGACGTTTCACAAAGTGAAAATTGGGTTCTAGAACATGCTAAGCTCAAGGCA

AGAGTTGAGGTACTTGAGAAGAACAAAAGGAATTTTATGGGGGAAGATCTTGATTCGTTGAGCTTGAAGGAGCTCCAAAGCTTGGAGCATCAGCTCGATG

CAGCTATCAAGAGCATTAGGTCAAGAAAGAACCAAGCTATGTTCGAATCCATATCTGCGCTCCAGAAGAAGGATAAAGCCTTGCAAGATCACAACAATTC

GCTTCTCAAAAAGATTAAGGAGAGGGAGAAGAAAACGGGTCAGCAAGAAGGACAATTAGTCCAATGCTCCAACTCTTCTTCAGTTCTTCTGCCTCAATAC

TGCGTAACCTCCTCCAGAGATGGCTTTGTGGAGAGAGTTGGGGGAGAGAACGGTGGTGCATCGTCGTTGACGGAACCAAACTCTCTGCTTCCGGCTTGGA

TGTTACGTCCTACCACTACGAACGAGTAG

>AT5G62165.1 | Symbols: AGL42, FYF | AGAMOUS-like 42, FOREVER YOUNG FLOWER | chr5:24965075-24968437 FORWARD LENGTH=633

ATGGTTAGAGGAAAGATAGAGATGAAGAAAATAGAAAACGCAACGAGTAGACAAGTGACTTTCTCAAAAAGAAGAAATGGTTTGTTGAAGAAAGCTTATG

AGCTCTCAGTACTCTGCGATGCTCAACTCTCTCTCATCATCTTCTCCCAGAGAGGAAGGCTTTATGAATTCTCTAGCTCTGATATGCAGAAGACGATCGA

ACGCTACCGCAAGTACACAAAAGATCATGAAACCAGCAATCACGACTCACAAATTCACTTGCAGCAATTGAAACAAGAAGCAAGCCACATGATAACAAAG

ATTGAACTCCTTGAGTTTCACAAGCGGAAGCTATTGGGACAAGGAATTGCTTCTTGTTCTCTAGAAGAGCTTCAAGAAATCGATAGTCAACTCCAAAGAA

GTCTGGGAAAGGTCCGAGAAAGAAAGGCTCAATTGTTCAAGGAGCAGTTGGAGAAACTAAAAGCAAAGGAGAAACAATTGTTAGAAGAGAACGTCAAGTT

ACATCAAAAGAATGTTATTAATCCATGGAGAGGATCATCGACTGATCAGCAGCAAGAGAAATACAAAGTTATAGATTTGAATTTGGAAGTTGAAACTGAC

TTATTCATCGGTTTGCCAAATAGAAACTGCTAG

>AT5G65050.1 | Symbols: MAF2, AGL31 | AGAMOUS-like 31, MADS AFFECTING FLOWERING 2 | chr5:25982415-25986114 FORWARD LENGTH=549

ATGGGTAGAAAAAAAGTCGAGATCAAGCGAATCGAGAACAAAAGTAGTCGACAAGTCACTTTCTCCAAACGACGCAATGGTCTCATCGAGAAAGCTCGAC

AACTTTCAATTCTCTGTGAATCTTCCATCGCTGTTCTCGTCGTCTCCGGCTCCGGAAAACTCTACAAGTCTGCCTCCGGTGACAACATGTCAAAGATCAT

TGATCGTTACGAAATACATCATGCTGATGAACTTGAAGCCTTAGATCTTGCAGAAAAAACTCGGAATTATCTGCCACTCAAAGAGTTACTAGAAATAGTC

CAAAGCAAGCTTGAAGAATCAAATGTCGATAATGCAAGTGTGGATACTTTAATTTCTCTGGAGGAACAGCTCGAGACTGCTCTGTCCGTAACTAGAGCTA

GGAAGACAGAACTAATGATGGGGGAAGTGAAGTCCCTTCAAAAAACGGTGGGGAAGAAGACGTTTCTGGTTATAGAAGGTGACAGAGGAATGTCATGGGA

AAATGGCTCCGGCAACAAAGTACGGGAGACTCTTCCGCTGCTCAAGTAA

>AT5G65060.1 | Symbols: AGL70, FCL3, MAF3 | AGAMOUS-like 70, MADS AFFECTING FLOWERING 3 | chr5:25987527-25991065 FORWARD LENGTH=591

ATGGGAAGAAGAAAAGTCGAGATCAAGCGAATCGAGAACAAAAGCAGTCGACAAGTCACTTTCTCCAAACGACGCAAAGGTCTCATCGAAAAAGCTCGAC

AACTTTCAATTCTCTGTGAATCTTCCATCGCTGTTGTCGCCGTCTCCGGTTCCGGAAAACTCTACGACTCTGCCTCCGGTGACAACATGTCAAAGATCAT

TGATCGTTATGAAATACATCATGCTGATGAACTTAAAGCCTTAGATCTTGCAGAAAAAATTCGGAATTATCTTCCACACAAGGAGTTACTAGAAATAGTC

CAAAGCAAGCTTGAAGAATCAAATGTCGATAATGTAAGTGTAGATTCTCTAATATCTATGGAGGAACAGCTCGAGACTGCTCTGTCAGTAATTAGAGCTA

AGAAGACAGAACTAATGATGGAGGATATGAAGTCACTTCAAGAAAGGGAGAAGTTGCTGATAGAAGAGAACCAGATTCTGGCTAGCCAGGTGGGGAAGAA

GACGTTTCTGGTTATAGAAGGTGACAGAGGAATGTCACGGGAAAATGGCTCCGGCAACAAAGTACCGGAGACTCTTTCGCTGCTCAAGTAA

>AT5G65070.1 | Symbols: MAF4, FCL4, AGL69 | AGAMOUS-like 69, MADS AFFECTING FLOWERING 4 | chr5:25992310-25995930 FORWARD LENGTH=603

ATGGGAAGAAGAAAAGTAGAGATCAAACGAATTGAGAACAAAAGCTCTCGACAAGTTACTTTCTGTAAACGACGAAATGGTCTCATGGAGAAAGCTCGTC

AACTCTCAATTCTTTGTGAATCCTCCGTCGCTCTTATCATCATCTCTGCCACCGGAAGACTCTACAGCTTCTCCTCAGGTGATAGCATGGCCAAGATCCT

CAGTCGTTATGAATTAGAACAGGCTGATGATCTTAAAACCTTGGATCTAGAAGAAAAAACTCTTAATTATCTTTCGCACAAGGAGTTGCTAGAAACAATC

CAATGCAAGATTGAAGAAGCGAAAAGCGATAATGTAAGTATAGATTGTCTAAAGTCCCTGGAAGAGCAGCTCAAGACTGCTCTGTCTGTAACTAGAGCTA

GGAAGACAGAACTAATGATGGAGCTTGTGAAGACCCATCAAGAGAAGGAGAAGCTGCTGAGAGAGGAGAACCAGAGTTTGACTAACCAGCTTATAAAGAT

GGGGAAGATGAAGAAGTCTGTGGAAGCAGAGGATGCAAGAGCAATGTCACCGGAAAGTAGCTCTGACAACAAGCCACCGGAGACTCTCCTGCTTCTCAAG

TAA

>AT5G65080.1 | Symbols: MAF5, AGL68 | AGAMOUS-like 68, MADS AFFECTING FLOWERING 5 | chr5:26000866-26002211 FORWARD LENGTH=354

GATCTTGAAGACAAAACTCAGGATTATCTTTCACACAAGGAGTTACTAGAAATAGTTCAAAGAAAGATTGAAGAAGCAAAAGGGGATAATGTAAGTATAG

AATCTCTAATTTCCATGGAAGAGCAGCTCAAGAGTGCTCTGTCTGTAATTAGAGCTAGGAAGACAGAGTTATTGATGGAGCTTGTGAAGAACCTTCAGGA

TAAGGAGAAGTTGCTGAAAGAAAAGAACAAGGTTCTAGCTAGCGAGGTGGGGAAGCTGAAGAAAATTTTGGAAACAGGGGATGAAAGAGCAGTAATGTCA

CCGGAAAATAGCTCTGGCCACAGCCCACCGGAGACTCTCCCGCTTCTCAAGTAA

>AT5G65330.1 | Symbols: AGL78 | AGAMOUS-like 78 | chr5:26110326-26111351 FORWARD LENGTH=1026

ATGAAGCAAGCTTCTTCTTCTTCTTCGTCACGTAATTCAACAAGTTTAACAAATAGACTTAAAACCATCTTCAAGAAAGCTGAAGAGCTTTCGATTCTCT

GTGCTATTGAGGTTTGCGTCATCTATTACGGACCAGACGGTGAACTGAGAACATGGCCTAAGGAGAGAGAGACAGTGAAAGACATGGCTTTGAGGTATAA

AGAGGCCAGAAAACGCAAGAAGAGCCGCAATCTTCATGAGTTCCTCGAGAAGGAGAAGGACAAGGACAAGGGGAAGACGAATTTGAAGAAGAATTGGTAT

CCAAACTTTGATCATTACTCTCCTCAACAACTCTCTCAATTGATTCAGTCCTTAGAACGGACTCTTTCTACTCTGCAAGAAAGGCTTCGTATTGTTGAGG

CGCAGAAACTACAGAACACAAACTTGGTGCATCAGAGTTTAACACCATCGTATCTGAACCAGACCCAACACTTGAATCCTAGCAAGTTCTCGCTGTTTAT

GTACAACCATGGAGATGCTACTCTCTCACAACTCCCACTGTCTGCTCCACACTCCAATCAACTCATCAATTACCAGAATCACTTGATGCAGCATGGTTTT

GGTCAGAACATGTGTTCGGACAACATCACCAACAACAACTTTGAACATCCTGGCGTGTCAAACACACAAGACTACTCACCGTTACTTTCGGTACAAGCAT

CTGCAGTGAATAACTACGGGTTGAATAATCACTTGATGCAGCAACAGGATCAGCTTCATGGTTTTGATCAGAACATGTGTATGGTGAGTGAAATCATCAA

CAACAACAACGGTCTACAACATCCTAATCTCTCAAACACGGTTCCACATGAATTCTCTTCTGATTTCAACCAGAACCCTTATGGTAATGCGGTCGGTAAC

ATTAGCTTCTCTCAAGACATGTTTTCAAGCTATGATGCGAGCAGTCTGCTACAGACATCTTCTCTGCCACCTCTCCACAACATTCCTAGCAGTTATTGTT

TTCCTGGCAACTCAAGACTTCTCTGA

#amino acid sequences of MADS-box genes in Arabidopsis thaliana

>AT1G01530.1 | Symbols: AGL28 | AGAMOUS-like 28 | chr1:192640-193662 REVERSE LENGTH=744

MARKNLGRRKIELVKMTNESNLQVTFSKRRSGLFKKGSELCTLCDAEIAIIVFSPSGKAYSFGHPNVNKLLDHSLGRVIRHNNTNFAESRTKLRIQMLNE

SLTEVMAEKEKEQETKQSIVQNERENKDAEKWWRNSPTELNLAQSTSMKCDLEALKKEVDEKVAQLHHRNLNFYVGSSSNVAAPAAVSGGNISTNHGFFD

QNGNSTSAPTLPFGFNVMNRTPAGYNSYQLQNQEVKQVHPQYWARYY*

>AT1G17310.1 | Symbols: AGL100 | agamous-like 100 | chr1:5928014-5928667 REVERSE LENGTH=654

MKDLFMEGERETSSMTCLTPKDSVQSPNMLVRQPKKETTTQTPKTTRGRQKIEIKKIEEETKRQVTFSKRRRGLFKKSAELSVLTGAKIAVITFSKCDRI

YRFGHVDALIDKYLRKSPVKLEGYSGDNAADEESRRPWWERPVESVPEEELEEYMAALSMLRENIGKKIVAMGNDRTVDMVPAWPINVMGWKPTMDMQKL

ENLTDGVNRCRVGQNGD*

>AT1G18750.1 | Symbols: AGL65 | AGAMOUS-like 65 | chr1:6467266-6469640 FORWARD LENGTH=1170

MGRVKLKIKRLESTSNRQVTYTKRKNGILKKAKELSILCDIDIVLLMFSPTGRATAFHGEHSCIEEVISKFAQLTPQERTKRKLESLEALKKTFKKLDHD

VNIHDFLGARNQTIEGLSNQVAIYQAQLMECHRRLSCWTNIDRIENTEHLDLLEESLRKSIERIQIHKEHYRKNQLLPIECATTQFHSGIQLPMAMGGNS

SMQEAHSMSWLPDNDHQQTILPGDSSFLPHREMDGSIPVYSSCFFESTKPEDQICSNPGQQFEQLEQQGNGCLGLQQLGEEYSYPTPFGTTLGMEEDQEK

KIKSEMELNNLQQQQQQQQQQQQQDPSMYDPMANNNGGCFQIPHDQSMFVNDHHHHHHHHHQNWVPDSMFGQTSYNQVCVFTPPLELSR*

>AT1G22130.1 | Symbols: AGL104 | AGAMOUS-like 104 | chr1:7812387-7814259 REVERSE LENGTH=1008

MGRVKLEIKRIENTTNRQVTFSKRRNGLIKKAYELSILCDIDIALIMFSPSDRLSLFSGKTRIEDVFSRFINLPKQERESALYFPDQNRRPDIQNKECLL

RILQQLKTENDIALQVTNPAAINSDVEELEHEVCRLQQQLQMAEEELRRYEPDPIRFTTMEEYEVSEKQLLDTLTHVVQRRDHLMSNHLSSYEASTMQPN

IGGPFVNDVVEGWLPENGTNQTHLFDASAHSNQLRELSSAMYEPLLQGSSSSSNQNNMSECHVTNHNGEMFPEWAQAYSSSALFASMQQQHEGVGPSIEE

MMPAQQSDIPGVTAETQVDHEVSDYETKVPQLSSQ*

>AT1G22590.2 | Symbols: AGL87 | AGAMOUS-like 87 | chr1:7983511-7984002 FORWARD LENGTH=492

MGRRKVTHQLISDNATRRVTFRKRKDGLLKKIYELTVLCGLPACAIIYSEYKDGPELWPNLNEVRSILNRLSELPVEKQTKYMMDQKDLMNKMIQDAEKK

LEKEKMHTRAMKLGLMAGSNDLITDTDCSEELARAADVVDKKLKAIRERIKAVEAGAPIIKRD*

>AT1G24260.1 | Symbols: AGL9, SEP3 | SEPALLATA3, AGAMOUS-like 9 | chr1:8593790-8595862 REVERSE LENGTH=753

MGRGRVELKRIENKINRQVTFAKRRNGLLKKAYELSVLCDAEVALIIFSNRGKLYEFCSSSSMLRTLERYQKCNYGAPEPNVPSREALAELSSQQEYLKL

KERYDALQRTQRNLLGEDLGPLSTKELESLERQLDSSLKQIRALRTQFMLDQLNDLQSKERMLTETNKTLRLRLADGYQMPLQLNPNQEEVDHYGRHHHQ

QQQHSQAFFQPLECEPILQIGYQGQQDGMGAGPSVNNYMLGWLPYDTNSI*

>AT1G26310.1 | Symbols: CAL1, AGL10, CAL | AGAMOUS-like 10, CAULIFLOWER | chr1:9100330-9103510 REVERSE LENGTH=768

MGRGRVELKRIENKINRQVTFSKRRTGLLKKAQEISVLCDAEVSLIVFSHKGKLFEYSSESCMEKVLERYERYSYAERQLIAPDSHVNAQTNWSMEYSRL

KAKIELLERNQRHYLGEELEPMSLKDLQNLEQQLETALKHIRSRKNQLMNESLNHLQRKEKEIQEENSMLTKQIKERENILRTKQTQCEQLNRSVDDVPQ

PQPFQHPHLYMIAHQTSPFLNMGGLYQEEDQTAMRRNNLDLTLEPIYNYLGCYAA*

>AT1G28450.1 | Symbols: AGL58 | AGAMOUS-like 58 | chr1:10003966-10004523 FORWARD LENGTH=558

MNPKKTKGKQKINIKKIEKDEDRSVTLSKRLNAIYTMIIELSILCGVEVAFIGYSCSGKPYTFGSPSFQAVVERFLNGEASSSSSSSLQRSVKNAHKQAK

IQELCKRYNRLVEELKVDEVKVKKAAALAETRAVNKDAWWKADPNDVKDHEKAKKMMEKYQELKEKLREEVALRIKRGHDENNNK*

>AT1G28460.1 | Symbols: AGL59 | AGAMOUS-like 59 | chr1:10006230-10006778 FORWARD LENGTH=549

MNPKKTKGKQKINIKKIEKDEGRSVTFSKRLNGIYTKISELSILCGVEVAFIGYSCSGKPYTFGSPSFQAVAERFLNGDASSSSSSSLVMNAHKQAKIQE

LCKKYNRLVEELKVDEVKVKKAAALAETRVVNKDVWWKVDPNDVKDHEKAKKMMEKYQELYDKLCEQAASRIKRGHDENNNK*

>AT1G29962.1 | Symbols: AGL64 | AGAMOUS-like 64 | chr1:10496730-10497287 FORWARD LENGTH=558

MKPKKTKGKQRINIKKIEKDEDRLVTLSKRRNGIYTKLSELSILCGAEVAFLGYSCSGKPYTFGSPSFQAVAERFLNGEASSSSSSSLQRSVMNAHQQAK

IQELCKVYNRLVEEITVEEVKLKKTAALAEMMPMNEDAWWKVDPNDVKDREEVKKMMEKHQELYEKLCEEAASRIKRGHDENNNK*

>AT1G31140.1 | Symbols: GOA, AGL63 | AGAMOUS-like 63, GORDITA | chr1:11118031-11119673 FORWARD LENGTH=642

MRKGKRVIKKIEEKIKRQVTFAKRKKSLIKKAYELSVLCDVHLGLIIFSHSNRLYDFCSNSTSMENLIMRYQKEKEGQTTAEHSFHSCSDCVKTKESMMR

EIENLKLNLQLYDGHGLNLLTYDELLSFELHLESSLQHARARKSEFMHQQQQQQTDQKLKGKEKGQGSSWEQLMWQAERQMMTCQRQKDPAPANEGGVPF

LRWGTTHRRSSPP*

>AT1G31630.1 | Symbols: AGL86 | AGAMOUS-like 86 | chr1:11318528-11319547 REVERSE LENGTH=1020

MRSKIKLSLIANKTSRRTTFRKRKGGITNKLHELTTLCGVKACAVISSPYENPVVWPSTEGVQEAVSMFMERPATEQSKLMMSHETYLQDKITKETKKLE

SLRRENRESQLRQFMFDCVEGKMSEHQYGARDLQDLSLYIDHYINQLNSSVMLLTNNGASSSSFPPPLHTSVAGAGAGAGAAPLVVAGAGAAPLAVAGAG

ASPLAVAGVGAAPLAVAGAGPPMAQNQYEPIQPYIPTAFSDNIQYQAPVDFNHQIQHGIYDNLSLDPNHQYPFQDDPFMEMLMEYPYEQVGYAAEHAHIP

FMNGNYYNYHQPPTVGLTTTGHMPSNNATTTTTTNTTVV*

>AT1G31640.1 | Symbols: AGL92 | AGAMOUS-like 92 | chr1:11322692-11324176 REVERSE LENGTH=1395

MRTKTKLVLIPDRHFRRATFRKRNAGIRKKLHELTTLCDIKACAVIYSPFENPTVWPSTEGVQEVISEFMEKPATERSKTMMSHETFLRDQITKEQNKLE

SLRRENRETQLKHFMFDCVGGKMSEQQYGARDLQDLSLFTDQYLNQLNARKKFLTEYGESSSSVPPLFDVAGANPPVVADQAAVTVPPLFAVAGANLPVV

ADQAAVTVPPLFAVAGANLPVVADQAAVNVPTGFHNMNVNQNQYEPVQPYVPTGFSDHIQYQNMNFNQNQQEPVHYQALAVAGAGLPMTQNQYEPVHYQS

LAVAGGGLPMSQLQYEPVQPYIPTVFSDNVQYQHMNLYQNQQEPVHYQALGVAGAGLPMNQNQYEPVQPYVPTGFSDHFQFENMNLNQNQQEPVQYQAPV

DFNHQIQQGNYDMNLNQNMKHAHIPFMDGNYYNYHQPPTVGLTSTGHMPSTTTTTTNNNNNNNV*

>AT1G33070.1 | Symbols: no symbol available | no full name available | chr1:11982889-11985299 FORWARD LENGTH=498

MPMRKEGITKKLYELATLCDIKACAGKISEHQYGARDLQDLSFHIDHYINQLNSRVKILTNNGESSSSVPPLLHTSVAGAGAAPLPVAGADLPMDQNQYE

PIQLYIPIGLSYHIQYQHEIYDNFKHAHILFMNGNYYNYHQTPTVGLTTTGHMPSNNNTTPTTDV*

>AT1G46408.1 | Symbols: AGL97 | AGAMOUS-like 97 | chr1:17232135-17232935 REVERSE LENGTH=801

MGGVKRKIAIEKIQNKNPRAVSFSKRRKGLYSKASELCLLSDAEIAIIATPVSSNSNAAFYSFGHSSVDNVVAAFLANQRPCDERFWWEDESLLKSENLE

ELREAMDSMSTMLRDLKELEKQRDHQTQTLIHQPCSARVCIQDYVTVNFDGFNTEEQTLAVSDNSNNNGLLGNLDECNEDFDDLDQIFDTVTNSEFLSVN

LEMDDVTVNSEGNTEEQTLAVSDNSNNNGLLGNLDECNEDFDDLDQIIEYLTSSEALSMNLKMDDV*

>AT1G47760.1 | Symbols: AGL102 | AGAMOUS-like 102 | chr1:17572451-17573159 FORWARD LENGTH=555

MGRRKIEIKFIEDSIERKATFSRRRNGIFKKADELAKLCNVEIAVLVISPTNIPYTYGYPCFNDVVERIQNPSASSKLRSLMKELEQIKEFQEDLRKKQQ

RNLEKSNMKENVDLKLEDLVAFKAKLEAYQAGLKRKHVEMEDLSSPSILSKNTKNKMMRTEYSSGQSKGMYEFRAFGPGFLGTI*

>AT1G48150.1 | Symbols: AGL74 | agamous-like 74 | chr1:17785397-17786368 FORWARD LENGTH=972

MQSSNVTDCTMRKRGTKRKIEIEKRMTKQQRSVACSKRRPTLFSKAADLCLLSGANIAVFVTSPDENSDVVYSFSGYSHASEIVDCYLNNKSPPKTTINP

ESAKFWWEEPDLYRDCDDLSELRIIEDRLMRTKKHLMDYLEKKEKSHSVSKSDQNPNNDSGSSSSSSQIASDFGQNPSTLSPSSLKIVSFDQNSYSSLEP

SSQVTTCFDQNPVFSVGSESSSDQSRYLVNEDSGFVDGLLCETEEENNGMRLPQETQTQPMFTEEDQSFWENLDVDDVFGLFKDDNNLEVPLQDHSSTNE

DDELLIDISEYLSEEAMECPCFS*

>AT1G54760.1 | Symbols: AGL85 | AGAMOUS-like 85 | chr1:20433912-20434397 FORWARD LENGTH=486

MKTDWSHYLSVEMESTISNELSILCGAEVAFLGYSCSGKPYTFGSPSFQAVAERFLNREASSSLQRSVMNAHQQAKIQELCKVYNRMVEEAKTEEAKVKK

AAALAETMPVDEDAWWKVDPKEVEDHEEAKKIMEKCEGLYEKLCNEAAARIQRGDAENNNK*

>AT1G59810.1 | Symbols: AGL50 | AGAMOUS-like 50 | chr1:22008604-22009455 FORWARD LENGTH=852

MAPRQKKPNKSDDDDDLRRKKQSFFKQRFPGFKKKASELSVLCGNSVGFICYGPDSDLHVWPQSQDHNPQALHEIVAKFNALSDERRKNHACDLNDFPHH

LKGLSREELRKHLLHLDSQLLGVREQKIEILKKTLTGSSEKDGARVSENSAISDHKLKIEPHLKDILSEDHLIRVSDKKLGSCDVFDELAYVVRGSRNLN

ENVSKYESKDADNTGLDHLVTLGGDYLQEAAAELYQTYNLGNFCDDHVWDLEFASRLPLLHTFSDPLMTTNTCQTMSTDMISI*

>AT1G60040.1 | Symbols: AGL49 | AGAMOUS-like 49 | chr1:22119075-22119929 REVERSE LENGTH=855

MAPRQKKPNKSDDDDGDLHRKKQSFFKQRFPGFKKKASELSVLCGNSVGFICYGPDNDLHVWPQSQDHNPQALHEIVAKFNALSDERRKNHACDLNDFPH

HLKGLSREELRKHLLHLDSQLLGVREQKIEILKKTLTGSSEKDGARVSENSAISDHKLKIEPNLTDILSEDHLIRVSDKKLGSCDVFDELAYVVRGSRNL

NENVSNYESKDAAYTGMDHLGTFGGNYLQEAAAELYQTYNLGNFCDDHVWDLEFASRLPPLHTFSDPLMTTNTCQTMSSDMISI*

>AT1G60880.1 | Symbols: AGL56 | AGAMOUS-like-56 | chr1:22411575-22412180 FORWARD LENGTH=606

MGGKKTKIEIKKIINKPAKTVAFTKRREGLFRKASQLCLLSPATQIAILAAPMTSKSHASFYSFGHSSVDNVVSSLLYDHPPLTANQDNRSGLGFWWEDK

RFDVSENVEELKEAVDAVSRMLNNVRCRLNDAVKSTQRDGGLEILHHQEEEVLQTRNDETKTNQTHEFEGGETSGSASWLENEDDILHFDDDFYTGIDPL

F*

>AT1G60920.1 | Symbols: AGL55 | AGAMOUS-like 55 | chr1:22429692-22430267 REVERSE LENGTH=576

MGGTKRKIEMKRIEDKNVRAVAFTKRKSGLFHKASELCLLSPGTQIAILATPLSSHSHASFYSFGHSSVDHVVSSLLHNQHPSLPTNQDNRSGLGFWWED

QAFDRLENVDELKEAVDAVSRMLNNVRLRLDDAVKSNQRDGSLVIHQEDEEVLQLGYKDTNQITKLEGETSASASLLKNVVDNLHIDDRYY*

>AT1G65300.1 | Symbols: PHE2, AGL38 | PHERES2, AGAMOUS-like 38 | chr1:24254929-24255765 FORWARD LENGTH=837

MKRKMKLSLIENSVSRKTTFTKRKKGMTKKLTELVTLCGVEACAVVYSPFNSIPEAWPSREGVEDVVSKFMELSVLDRTKKMVDQETFISQRIAKEKEQL

QKLRDENHNSQIRELMFGCLKGETNVYNLDGRDLQDLSLYIDKYLNGLTRRIEILIENGESSSSLPLPIVANAAAPVGFDGPMFQYHNQNQQKPVQFQYQ

ALYDFYDQIPKKIHGFNMNMNKDSNQSMVLDLNQNLNDGEDEGIPCMDNNNYHPEIDCLATVTTAPTDVCAPNITNDL*

>AT1G65330.1 | Symbols: PHE1, AGL37 | AGAMOUS-like 37, PHERES1 | chr1:24266481-24267320 REVERSE LENGTH=840

MRGKMKLSFIENDSVRKTTFTKRKKGMLKKFNELVTLCGVDACAVIRSPYNSIQEPWPSREGVEEVMSKFMEFSVLDRTKKMVDQETFLRQRIAKETERL

QKLRDENRNSQIRDLMFGCLKGEVDVSHLHGRDLLDLNVFLNKYLNGVIRRVEILKENGESSSSVPPPIGVAPTVVDASVPIGFDGRMIQDQNQNQQEPV

QFQYQALYDFYDQIPKKLHDFNMKMNIDPNQSMNLDLNDGEDEGIPCMDNNNYHPEIDCLATVTTAPTDVCAPNIINDL*

>AT1G65360.1 | Symbols: AGL23 | AGAMOUS-like 23 | chr1:24281337-24282151 FORWARD LENGTH=681

MVKKTLGRRKVEIVKMTKESNLQVTFSKRKAGLFKKASEFCTLCDAKIAMIVFSPAGKVFSFGHPNVDVLLDHFRGCVVGHNNTNLDESYTKLHVQMLNK

SYTEVKAEVEKEQKNKQSRAQNERENENAEEWWSKSPLELNLSQSTCMIRVLKDLKKIVDEKAIQLIHQTNPNFYVGSSSNAAAPATVSGGNISTNQGFF

DQNGMTTNPTQTLLFGFDIMNRTPGV*

>AT1G69120.1 | Symbols: AP1, AtAP1, AGL7 | AGAMOUS-like 7, APETALA1 | chr1:25982576-25986102 REVERSE LENGTH=771

MGRGRVQLKRIENKINRQVTFSKRRAGLLKKAHEISVLCDAEVALVVFSHKGKLFEYSTDSCMEKILERYERYSYAERQLIAPESDVNTNWSMEYNRLKA

KIELLERNQRHYLGEDLQAMSPKELQNLEQQLDTALKHIRTRKNQLMYESINELQKKEKAIQEQNSMLSKQIKEREKILRAQQEQWDQQNQGHNMPPPLP

PQQHQIQHPYMLSHQPSPFLNMGGLYQEDDPMAMRRNDLELTLEPVYNCNLGCFAA*

>AT1G69540.1 | Symbols: AGL94 | AGAMOUS-like 94 | chr1:26145306-26147159 REVERSE LENGTH=1035

MGRVKLKIKKLQNMNGRQCTYTKRRHGIMKKAKELSILCDIDVVLLMFSPMGKASICIGKHSIGEVIAKFAQLSPQERAKRKLENLEALRKTFMKANHDI

DISKFLDRISTPTVEVLSEKIRFLQTQLSDIHTRLSYWTDVDNIDSVDVLQQLEHSLRQSLAQIYGRKASMPQRQQQQLMSSQCKNQLQTEIDIDFGMEM

EQQLENFSWVRTDENMNVPIEEEDPNLQLHHMYKDITCSASSALGNYSGLFSKSSDILQKLETGSIPGTSADPNQQFSNLSFLNDQKLKQLAEWNLLGSP

ADYYVSQILEASYKPQIGGKNNGASSETLPYVAVFDDPLYFWPN*

>AT1G71692.1 | Symbols: XAL1, AGL12 | XAANTAL1, AGAMOUS-like 12 | chr1:26952903-26954939 REVERSE LENGTH=636

MARGKIQLKRIENPVHRQVTFCKRRTGLLKKAKELSVLCDAEIGVVIFSPQGKLFELATKGTMEGMIDKYMKCTGGGRGSSSATFTAQEQLQPPNLDPKD

EINVLKQEIEMLQKGISYMFGGGDGAMNLEELLLLEKHLEYWISQIRSAKMDVMLQEIQSLRNKEGVLKNTNKYLLEKIEENNNSILDANFAVMETNYSY

PLTMPSEIFQF*

>AT1G72350.1 | Symbols: AGL60 | agamous-like 60 | chr1:27239273-27239947 REVERSE LENGTH=675

MEDGEASTITFLPTTEPKPLQNPNLLAKPKKETKQKKPKTTKGRQKIEIKEIMLETRRQVTFSKRRSGLFKKAAELSVLCGAQIGIITFSRCDRIYSFGN

VNSLIDKYLRKAPVMLRSHPGGNVANGEEDNDGLMWWERAVESVPEEHMEEYKNALSVLRENLLTRIYQMSGDRTVENLPAFPNEMAMADWKLTNENLMA

RNDRGYGGNNGDLEFAFMPQNGRQ*

>AT1G77080.2 | Symbols: FLM, AGL27, MAF1 | FLOWERING LOCUS M, AGAMOUS-like 27, MADS AFFECTING FLOWERING 1 | chr1:28955679-28959845 FORWARD LENGTH=579

MGRRKIEIKRIENKSSRQVTFSKRRNGLIDKARQLSILCESSVAVVVVSASGKLYDSSSGDEIEALFKPEKPQCFELDLEEKIQNYLPHKELLETVQSKL

EEPNVDNVSVDSLISLEEQLETALSVSRARKAELMMEYIESLKEKEKLLREENQVLASQMGKNTLLATDDERGMFPGSSSGNKIPETLPLLN*

>AT1G77950.1 | Symbols: AGL67 | AGAMOUS-like 67 | chr1:29307029-29309667 FORWARD LENGTH=759

MGRVKLELKRIEKSTNRQITFSKRKKGLIKKAYELSTLCDIDLALLMFSPSDRLCLFSGQTRIEDVLARYINLPDQERENAIVFPDQSKRQGIQNKEYLL

RTLEKLKIEDDMALQINEPRPEATNSNVEELEQEVCRLQQQLQISEEELRKFEPDPMRLTSMEEIEACEANLINTLTRVVQRREHLLRKSCEAQSNQQSM

DGILLNDIVEDWGPEPEPKQAHMIANSAHHSNQPSYDLLLRRSNSSSNQNPK*

>AT1G77980.1 | Symbols: AGL66 | AGAMOUS-like 66 | chr1:29315212-29317067 REVERSE LENGTH=999

MGRVKLEIKRIENTTNRQVTFSKRRNGLIKKAYELSILCDIDIALLMFSPSDRLSLFSGKTRIEDVFSRYINLSDQERENALVFPDQSRRPDFQSKEYLL

RTLQQLKAENDIALQLTNPTAINSDVEELEHEVYKLQQQLLMAEEELRKYEPDPIRFTTMEEYETCEKQLMDTLTRVNQRREHILSQDQLSSYEASALQQ

QQSMGGPFGNDVVGGWLTENGPNEAHLFDASAHSAMYETLLQGSSSSSNQNNIMGESNVSNHNGDMFQEWAQAYNSTTAHNPSTLFPPMQHQHGLVVDPN

IEEIEIPVMKKDAQADHEVSDYDIRMPQLSSQ*

>AT2G03060.1 | Symbols: AGL30 | AGAMOUS-like 30 | chr2:901614-903639 FORWARD LENGTH=1146

MGRVKLKIKKLENTNGRQSTFAKRKNGILKKANELSILCDIDIVLLMFSPTGKAAICCGTRSSMEEVIAKFSQVTPQERTKRKFESLENLKKTFQKLDHD

VNIREFIASSNSTVEDLSTQARILQARISEIHGRLSYWTEPDKINNVEHLGQLEISIRQSLDQLRAHKEHFGQQQQAMQIENANFVKDWSTCSMQDGIQI

PLEQQLQSMSWILNSNTTNIVTEEHNSIPQREVECSASSSFGSYPGYFGTGKSPEMTIPGQETSFLDELNTGQLKQDTSSQQQFTNNNNITAYNPNLHND

MNHHQTLPPPPLPLTLPHAQVYIPMNQREYHMNGFFEAPPPDSSAYNDNTNQTRFGSSSSSLPCSISMFDEYLFSQMQQPN*

>AT2G03710.1 | Symbols: SEP4, AGL3 | SEPALLATA 4, AGAMOUS-like 3 | chr2:1129622-1131628 FORWARD LENGTH=777

MGRGKVELKRIENKINRQVTFAKRRNGLLKKAYELSVLCDAEIALLIFSNRGKLYEFCSSPSGMARTVDKYRKHSYATMDPNQSAKDLQDKYQDYLKLKS

RVEILQHSQRHLLGEELSEMDVNELEHLERQVDASLRQIRSTKARSMLDQLSDLKTKEEMLLETNRDLRRKLEDSDAALTQSFWGSSAAEQQQQHQQQQQ

GMSSYQSNPPIQEAGFFKPLQGNVALQMSSHYNHNPANATNSATTSQNVNGFFPGWMV*

>AT2G14210.1 | Symbols: ANR1, AtANR1, AGL44 | ARABIDOPSIS NITRATE REGULATED 1, AGAMOUS-like 44 | chr2:6018841-6023585 FORWARD LENGTH=705

MGRGKIVIRRIDNSTSRQVTFSKRRSGLLKKAKELSILCDAEVGVIIFSSTGKLYDYASNSSMKTIIERYNRVKEEQHQLLNHASEIKFWQREVASLQQQ

LQYLQECHRKLVGEELSGMNANDLQNLEDQLVTSLKGVRLKKDQLMTNEIRELNRKGQIIQKENHELQNIVDIMRKENIKLQKKVHGRTNAIEGNSSVDP

ISNGTTTYAPPQLQLIQLQPAPREKSIRLGLQLS*

>AT2G22540.1 | Symbols: AGL22, FAQ1, SVP | SHORT VEGETATIVE PHASE, Flowering Arabidopsis QTL1, AGAMOUS-like 22 | chr2:9580417-9583603 FORWARD LENGTH=723

MAREKIQIRKIDNATARQVTFSKRRRGLFKKAEELSVLCDADVALIIFSSTGKLFEFCSSSMKEVLERHNLQSKNLEKLDQPSLELQLVENSDHARMSKE

IADKSHRLRQMRGEELQGLDIEELQQLEKALETGLTRVIETKSDKIMSEISELQKKGMQLMDENKRLRQQGTQLTEENERLGMQICNNVHAHGGAESENA

AVYEEGQSSESITNAGNSTGAPVDSESSDTSLRLGLPYGG*

>AT2G22630.1 | Symbols: AGL17 | AGAMOUS-like 17 | chr2:9618372-9621641 FORWARD LENGTH=684

MGRGKIVIQKIDDSTSRQVTFSKRRKGLIKKAKELAILCDAEVCLIIFSNTDKLYDFASSSVKSTIERFNTAKMEEQELMNPASEVKFWQREAETLRQEL

HSLQENYRQLTGVELNGLSVKELQNIESQLEMSLRGIRMKREQILTNEIKELTRKRNLVHHENLELSRKVQRIHQENVELYKKAYGTSNTNGLGHHELVD

AVYESHAQVRLQLSQPEQSHYKTSSNS*

>AT2G24840.1 | Symbols: DIA, AGL61 | DIANA, AGAMOUS-like 61 | chr2:10581082-10581876 FORWARD LENGTH=795

MYVTKYKNKLPTLQVDLIIMPHQTQACIYKQTLSLHQIFQQRKATKTLHTKKTMMSKKKESIGRQKIPMVKIKKESHRQVTFSKRRAGLFKKASELCTLC

GAEIGIIVFSPAKKPFSFGHPSVESVLDRYVSRNNMSLAQSQQLQGSPAASCELNMQLTHILSEVEEEKKKGQAMEEMRKESVRRSMINWWEKPVEEMNM

VQLQEMKYALEELRKTVVTNMASFNEAKDDVFGFLDNKVTVPPYVNMPSGPSNIYNFANGNGCF*

>AT2G26320.1 | Symbols: AGL33 | AGAMOUS-like 33 | chr2:11205389-11206287 REVERSE LENGTH=330

MKRTIKNKNKQIVKENMGRKKLKLKRIESLKERSSKFSKRKKGLFKKAEEVALLCDSDIMLIVVSPTEKPTVFNTRSRSFHTILERFCMLSLQEREERCD

LSYFYIIIT*

>AT2G28700.1 | Symbols: AGL46 | AGAMOUS-like 46 | chr2:12317384-12318724 REVERSE LENGTH=990

MARKKLNLTYIFNDRMRKRSFKQRREGFLKKLNDLKVLCDVNACAVVYNPFNSNPDVWPSKSEVNNIIKKFEMLPETQKKVKSVNHEEFLNLYISKVEKQ

SKKLIVENKETCLKEVMFKCLGGNMGDFVMNDNDRLDLCKFIDHYLRNLYHHKNVTLNNPNFEIGESSSLMDMAPTATTGNMATTVVDEGMTPLLIAEGS

SSSFLNSPLFNSPQLTNELQLIVSQNHRLENSLASNLFFSEGQDICIPDMNQSIIPSNQGAEHVDFLESNFLPNNNQEVYIPVMDQDEVYNPNQNHYENQ

QGFIDEMMKYAEKTSFPWMVENHCYNHNQ*

>AT2G34440.1 | Symbols: AGL29 | AGAMOUS-like 29 | chr2:14526950-14527468 FORWARD LENGTH=519

MGRRKIKMEMVQDMNTRQVTFSKRRTGLFKKASELATLCNAELGIVVFSPGGKPFSYGKPNLDSVAERFMREYDDSDSGDEEKSGNYRPKLKRLSERLDL

LNQEVEAEKERGEKSQEKLESAGDERFKESIETLTLDELNEYKDRLQTVHGRIEGQVNHLQASSCLMLLSRK*

>AT2G40210.1 | Symbols: AGL48 | AGAMOUS-like 48 | chr2:16793213-16794328 REVERSE LENGTH=1116

MTRKKVKLVWIENDKSRATSLQKMRVGLLKKVKELTILCAVRAIVIIFSPDKVGPLVWPSPQATHGLLDEFFALPKSVQKKKESNVESYLKEKTHKFQEQ

LKKSKKKNKEHVIDELMMQLQSGREIADLNQSEMYALLSFSRDTILLCRKKLAFMQFPPLRDPPVFPFEIQVEEFKTTTNDGFVGGGQDNKRAGRTDEAT

RFINTDIFKQSKSYYFFDEWVFPPSPPKYEIPQQMENGNPNPKSYRLYQGSSSNGNPHLEMDPFRLQMMTSQGLAGSVSQPLQHHSMINNPTMAMNQPSQ

DPFDYMRSELGINEGININNSQFYMSNNTITANDGVRQEPYPNVTTAGENNGDATTSNTNMVWPGFNNHHF*

>AT2G42830.1 | Symbols: AGL5, SHP2 | AGAMOUS-like 5, SHATTERPROOF 2 | chr2:17820602-17823806 FORWARD LENGTH=741

MEGGASNEVAESSKKIGRGKIEIKRIENTTNRQVTFCKRRNGLLKKAYELSVLCDAEVALVIFSTRGRLYEYANNSVRGTIERYKKACSDAVNPPTITEA

NTQYYQQEASKLRRQIRDIQNLNRHILGESLGSLNFKELKNLESRLEKGISRVRSKKHEMLVAEIEYMQKREIELQNDNMYLRSKITERTGLQQQESSVI

HQGTVYESGVTSSHQSGQYNRNYIAVNLLEPNQNSSNQDQPPLQLV*

>AT2G45650.1 | Symbols: AGL6, RSB1 | AGAMOUS-like 6, REDUCED SHOOT BRANCHING 1 | chr2:18804453-18806291 FORWARD LENGTH=759

MGRGRVEMKRIENKINRQVTFSKRRNGLLKKAYELSVLCDAEVALIIFSSRGKLYEFGSVGIESTIERYNRCYNCSLSNNKPEETTQSWCQEVTKLKSKY

ESLVRTNRNLLGEDLGEMGVKELQALERQLEAALTATRQRKTQVMMEEMEDLRKKERQLGDINKQLKIKFETEGHAFKTFQDLWANSAASVAGDPNNSEF

PVEPSHPNVLDCNTEPFLQIGFQQHYYVQGEGSSVSKSNVAGETNFVQGWVL*

>AT2G45660.1 | Symbols: AGL20, SOC1, ATSOC1 | SUPPRESSOR OF OVEREXPRESSION OF CO 1, AGAMOUS-like 20 | chr2:18807799-18810193 REVERSE LENGTH=645

MVRGKTQMKRIENATSRQVTFSKRRNGLLKKAFELSVLCDAEVSLIIFSPKGKLYEFASSNMQDTIDRYLRHTKDRVSTKPVSEENMQHLKYEAANMMKK

IEQLEASKRKLLGEGIGTCSIEELQQIEQQLEKSVKCIRARKTQVFKEQIEQLKQKEKALAAENEKLSEKWGSHESEVWSNKNQESTGRGDEESSPSSEV

ETQLFIGLPCSSRK*

>AT3G02310.1 | Symbols: AGL4, SEP2 | AGAMOUS-like 4, SEPALLATA 2 | chr3:464554-466687 REVERSE LENGTH=753

MGRGRVELKRIENKINRQVTFAKRRNGLLKKAYELSVLCDAEVSLIVFSNRGKLYEFCSTSNMLKTLERYQKCSYGSIEVNNKPAKELENSYREYLKLKG

RYENLQRQQRNLLGEDLGPLNSKELEQLERQLDGSLKQVRCIKTQYMLDQLSDLQGKEHILLDANRALSMKLEDMIGVRHHHIGGGWEGGDQQNIAYGHP

QAHSQGLYQSLECDPTLQIGYSHPVCSEQMAVTVQGQSQQGNGYIPGWML*

>AT3G04100.1 | Symbols: AGL57 | AGAMOUS-like 57 | chr3:1075299-1075922 FORWARD LENGTH=624

MSSTKQAKGRKTKGKQKIEMKKVENYGDRMITFSKRKTGIFKKMNELVAMCDVEVAFLIFSQPKKPYTFAHPSMKKVADRLKNPSRQEPLERDDTRPLVE

AYKKRRLHDLVKKMEALEEELAMDLEKLKLLKESRNEKKLDKMWWNFPSEGLSAKELQQRYQAMLELRDNLCDNMAHLRLGKDCGGSSSVRVGRRVSGGV

RLFDREA*

>AT3G05860.1 | Symbols: AGL45 | agamous-like 45 | chr3:1751406-1752355 REVERSE LENGTH=783

MTRKKLNLSYITNESMRKATFNKRKKGLVKKIHELSVLCGIEACAVIYSPFNSNPEVWPSNSEVKNVMENFEMLTKLEQEKKMVSHEGFIRQNISKTMES

NNKKMIDNAERTMKEAMFQLLSGKGEKLNLTDRNREDLCKYIDQYLKELYHHKNKTINQSHIEPGESSGATNAMTPTSVVEPIISSIQRPNQNPNFNHLS

HNQYQYQQQFGYPILVQDGIYNPSQIQNQHEEWLDDHMMNHSKEISHPLMDDNNFYYQQP*

>AT3G18650.1 | Symbols: AGL103 | AGAMOUS-like 103 | chr3:6417344-6418504 REVERSE LENGTH=1161

MASSSSSSLSFSTSKKNKTFFKKPNSAFSSSRATSLIKRQQTVFKKAKELSILCDIDVCVICYGSNGELKTWPEEREKVKAIARRYGELSETKRRKGSVD

LHEFLEKMNKDDPEKEEKKKIKVRRVPKVKYPVWDPRFDNYSVEQLMGLVQSLERNLTRIQHRTCAVVEAQGQRRVQYTNMANQELMMANTMNQLQQHSN

QVSMYLWNHGNGAFSQIPVSALASNQTQSLAPIPPELMIYPNSDAGNYSGSLGVQGTGINGLQNMNMLTYNNINSVNDFSKQFDQNSRAESYSSLLGVHE

DGNNEFENPNMSSRNNFNVQDCAGLLGMQGAGTNGLQSMNMHDYSNNNSINSNGLSHQYVQFPTYNSQHQDRVFNLDQNGNNTRSL*

>AT3G30260.1 | Symbols: AGL79 | AGAMOUS-like 79 | chr3:11909119-11912880 FORWARD LENGTH=750

MGRGRVQLRRIENKIRRQVTFSKRRTGLVKKAQEISVLCDAEVALIVFSPKGKLFEYSAGSSMERILDRYERSAYAGQDIPTPNLDSQGECSTECSKLLR

MIDVLQRSLRHLRGEEVDGLSIRDLQGVEMQLDTALKKTRSRKNQLMVESIAQLQKKEKELKELKKQLTKKAGEREDFQTQNLSHDLASLATPPFESPHE

LRRTISPPPPPLSSGDTSQRDGVGEVAAGTLIRRTNATLPHWMPQLTGE*

>AT3G54340.1 | Symbols: AP3, ATAP3 | APETALA 3 | chr3:20119428-20121087 REVERSE LENGTH=699

MARGKIQIKRIENQTNRQVTYSKRRNGLFKKAHELTVLCDARVSIIMFSSSNKLHEYISPNTTTKEIVDLYQTISDVDVWATQYERMQETKRKLLETNRN

LRTQIKQRLGECLDELDIQELRRLEDEMENTFKLVRERKFKSLGNQIETTKKKNKSQQDIQKNLIHELELRAEDPHYGLVDNGGDYDSVLGYQIEGSRAY

ALRFHQNHHHYYPNHGLHAPSASDIITFHLLE*

>AT3G57230.1 | Symbols: AGL16 | AGAMOUS-like 16 | chr3:21177710-21180671 FORWARD LENGTH=723

MGRGKIAIKRINNSTSRQVTFSKRRNGLLKKAKELAILCDAEVGVIIFSSTGRLYDFSSSSMKSVIERYSDAKGETSSENDPASEIQFWQKEAAILKRQL

HNLQENHRQMMGEELSGLSVEALQNLENQLELSLRGVRMKKDQMLIEEIQVLNREGNLVHQENLDLHKKVNLMHQQNMELHEKVSEVEGVKIANKNSLLT

NGLDMRDTSNEHVHLQLSQPQHDHETHSKAIQLNYFSFIA*

>AT3G57390.1 | Symbols: AGL18 | AGAMOUS-like 18 | chr3:21233910-21235735 FORWARD LENGTH=771

MGRGRIEIKKIENINSRQVTFSKRRNGLIKKAKELSILCDAEVALIIFSSTGKIYDFSSVCMEQILSRYGYTTASTEHKQQREHQLLICASHGNEAVLRN

DDSMKGELERLQLAIERLKGKELEGMSFPDLISLENQLNESLHSVKDQKTQILLNQIERSRIQEKKALEENQILRKQVEMLGRGSGPKVLNERPQDSSPE

ADPESSSSEEDENDNEEHHSDTSLQLGLSSTGYCTKRKKPKIELVCDNSGSQVASD*

>AT3G58780.1 | Symbols: SHP1, AGL1 | SHATTERPROOF 1, AGAMOUS-like 1 | chr3:21739150-21741766 FORWARD LENGTH=747

MEEGGSSHDAESSKKLGRGKIEIKRIENTTNRQVTFCKRRNGLLKKAYELSVLCDAEVALVIFSTRGRLYEYANNSVRGTIERYKKACSDAVNPPSVTEA

NTQYYQQEASKLRRQIRDIQNSNRHIVGESLGSLNFKELKNLEGRLEKGISRVRSKKNELLVAEIEYMQKREMELQHNNMYLRAKIAEGARLNPDQQESS

VIQGTTVYESGVSSHDQSQHYNRNYIPVNLLEPNQQFSGQDQPPLQLV*

>AT3G61120.1 | Symbols: AGL13 | AGAMOUS-like 13 | chr3:22618414-22620466 REVERSE LENGTH=735

MGRGKVEVKRIENKITRQVTFSKRKSGLLKKAYELSVLCDAEVSLIIFSTGGKLYEFSNVGVGRTIERYYRCKDNLLDNDTLEDTQGLRQEVTKLKCKYE

SLLRTHRNLVGEDLEGMSIKELQTLERQLEGALSATRKQKTQVMMEQMEELRRKERELGDINNKLKLETEDHDFKGFQDLLLNPVLTAGCSTDFSLQSTH

QNYISDCNLGYFLQIGFQQHYEQGEGSSVTKSNARSDAETNFVQ*

>AT3G66656.1 | Symbols: AGL91 | AGAMOUS-like 91 | chr3:2091262-2091798 REVERSE LENGTH=537

MGRRKIKMEKVQDTNTKQVTFSKRRLGLFKKASELATLCNAEVGIVVFSPGNKPYSFGKPNFDVIAERFKNEFEEEEEGDSCETSGYSRGNRARQEKKIC

KRLNSITEEAEAEKKHGEDLHKWLESAEQDKFNKPIEELTLEELKEFEAKIKKISCGIQSNISHMQASSSLMFLSNDN*

>AT4G02235.1 | Symbols: AGL51 | AGAMOUS-like 51 | chr4:980955-981711 FORWARD LENGTH=591

MKQSSFSSSSSSRNSTSLTNRLKTIFKKAEELSILCAIDVCVIYYGPDGELRTWPKERNTVKDMASRYKEATKRKKKRTLSTLQERLRIVESQKQQNKNL

VHQSLTPSYLNQIQHLNPSNFSPYMYNHGDAATLSQLPLSASLSNQLQLPESLDAAWFWSEHVFGQHHQQQQLSTSWRVKHTRILTVSFGTSICSE*

>AT4G09960.1 | Symbols: STK, AGL11 | AGAMOUS-like 11, SEEDSTICK | chr4:6236713-6239409 REVERSE LENGTH=693

MGRGKIEIKRIENSTNRQVTFCKRRNGLLKKAYELSVLCDAEVALIVFSTRGRLYEYANNNIRSTIERYKKACSDSTNTSTVQEINAAYYQQESAKLRQQ

IQTIQNSNRNLMGDSLSSLSVKELKQVENRLEKAISRIRSKKHELLLVEIENAQKREIELDNENIYLRTKVAEVERYQQHHHQMVSGSEINAIEALASRN

YFAHSIMTAGSGSGNGGSYSDPDKKILHLG*

>AT4G11250.1 | Symbols: AGL52 | AGAMOUS-like 52 | chr4:6849578-6850567 FORWARD LENGTH=990

MKQASSSSSCNPTSLTNRLKTIFKKAEELSILCAIDVCVIYYGPDGDLRTWPKDRETVKNMALRYKEDRKRKKCLNLHEFLEKEKVKDKDKYKGKTNYVK

NPNWYPNFDHYSPQQLSQLIQSLERTLSTLQKRLRIVESQKKQNTNLVHQSLTPSYLNQTQHLDPSKFSLYMYNHGDATLSQLPLSASQSNQLINYQMQH

GFGQNMCLDNITNNNNFQHPGVSNTQDYSPLLSANNYGLNNHLMQQQDQLHGFDQNLCMMSEIINNNNGLQHPNLSNTVPHEFPYGNTSFSQDMFSSYDG

SSLLQTSSLPPLHNIPNSYCFSDNSRLLC*

>AT4G11880.1 | Symbols: AGL14, XAL2 | XAANTAL2, AGAMOUS-like 14 | chr4:7143512-7147108 FORWARD LENGTH=666

MVRGKTEMKRIENATSRQVTFSKRRNGLLKKAFELSVLCDAEVALIIFSPRGKLYEFSSSSSIPKTVERYQKRIQDLGSNHKRNDNSQQSKDETYGLARK

IEHLEISTRKMMGEGLDASSIEELQQLENQLDRSLMKIRAKKYQLLREETEKLKEKERNLIAENKMLMEKCEMQGRGIIGRISSSSSTSELDIDDNEMEV

VTDLFIGPPETRHFKKFPPSN*

>AT4G18960.1 | Symbols: AG | AGAMOUS | chr4:10383917-10388272 FORWARD LENGTH=759

TAYQSELGGDSSPLRKSGRGKIEIKRIENTTNRQVTFCKRRNGLLKKAYELSVLCDAEVALIVFSSRGRLYEYSNNSVKGTIERYKKAISDNSNTGSVAE

INAQYYQQESAKLRQQIISIQNSNRQLMGETIGSMSPKELRNLEGRLERSITRIRSKKNELLFSEIDYMQKREVDLHNDNQILRAKIAENERNNPSISLM

PGGSNYEQLMPPPQTQSQPFDSRNYFQVAALQPNNHHYSSAGRQDQTALQLV*

>AT4G22950.1 | Symbols: GL19, AGL19 | AGAMOUS-like 19 | chr4:12023946-12027421 REVERSE LENGTH=660

MVRGKTEMKRIENATSRQVTFSKRRNGLLKKAFELSVLCDAEVALVIFSPRSKLYEFSSSSIAATIERYQRRIKEIGNNHKRNDNSQQARDETSGLTKKI

EQLEISKRKLLGEGIDACSIEELQQLENQLDRSLSRIRAKKYQLLREEIEKLKAEERNLVKENKDLKEKWLGMGTATIASSQSTLSSSEVNIDDNMEVET

GLFIGPPETRQSKKFPPQN*

>AT4G24540.1 | Symbols: AGL24 | AGAMOUS-like 24 | chr4:12671160-12673645 REVERSE LENGTH=663

MAREKIRIKKIDNITARQVTFSKRRRGIFKKADELSVLCDADVALIIFSATGKLFEFSSSRMRDILGRYSLHASNINKLMDPPSTHLRLENCNLSRLSKE

VEDKTKQLRKLRGEDLDGLNLEELQRLEKLLESGLSRVSEKKGECVMSQIFSLEKRGSELVDENKRLRDKLETLERAKLTTLKEALETESVTTNVSSYDS

GTPLEDDSDTSLKLGLPSWE*

>AT4G36590.1 | Symbols: AGL40 | agamous-like 40 | chr4:17261146-17262189 REVERSE LENGTH=747

MVRSTKGRQKIEMKKMENESNLQVTFSKRRFGLFKKASELCTLSGAEILLIVFSPGGKVFSFGHPSVQELIHRFSNPNHNSAIVHHQNNNLQLVETRPDR

NIQYLNNILTEVLANQEKEKQKRMVLDLLKESREQVGNWYEKDVKDLDMNETNQLISALQDVKKKLVREMSQYSQVNVSQNYFGQSSGVIGGGNVGIDLF

DQRRNAFNYNPNMVFPNHTPPMFGYNNDGVLVPISNMNYMSSYNFNQS*

>AT4G37940.1 | Symbols: AGL21 | AGAMOUS-like 21 | chr4:17835695-17838621 REVERSE LENGTH=687

MGRGKIVIQRIDDSTSRQVTFSKRRKGLIKKAKELAILCDAEVGLIIFSSTGKLYDFASSSMKSVIDRYNKSKIEQQQLLNPASEVKFWQREAAVLRQEL

HALQENHRQMMGEQLNGLSVNELNSLENQIEISLRGIRMRKEQLLTQEIQELSQKRNLIHQENLDLSRKVQRIHQENVELYKKAYMANTNGFTHREVAVA

DDESHTQIRLQLSQPEHSDYDTPPRANE*

>AT5G04640.1 | Symbols: AGL99 | AGAMOUS-like 99 | chr5:1332825-1333793 FORWARD LENGTH=969

MGGVKRKISIELIEKKDSRAVAFSKRSRGLYSKASDLCLLSDAQIAIIATPVSSKSNVSFYTFGHSSVDNVVAAFLTNQRPREGLGLDYWWEDERLSKSE

DLEELRDAMDSMSKMLKDLKDLQNQRDCEEDVKKKGVLHGTHQKQTFNPESCSVNFDGFNKNTEEFDLDEIFDYVSTAEALSMNLDMDDVSVVTTNQNPV

SASETVEDRELVVHKNMDEDNIHVSDMDDKDTMLMISDKNNVLPENLDEFDQELDLDQLLDFETNYESLLKSCEMEDYASMVTTKQNLCSNPEAVEDGGL

MIQKDLPEDNLCFSDYFSDLHC*

>AT5G06500.1 | Symbols: AGL96 | AGAMOUS-like 96 | chr5:1982444-1983172 FORWARD LENGTH=729

MARKKVRAAWIRDDRMRRASLKRRLTGLIKKVNELSILCDMRASVVVFNREEEQLTAWPSPEAANSLIDNFYSLTDHERTMKAVDPESYVQTVIEKIEKK

RADTRKVITEFEMDELMFQVQNGRELADLSPTEADKLIPYADKKLMWLSKRMGSTGVDALRASNVASGSGGNGLNMMETGRSFYYVDKWVFVDPQVQNPC

DVETHLPTMVSGLDLNMEPSDEDLGTYKGESSMAGGAEDDAE*

>AT5G10140.1 | Symbols: FLC, RSB6, AGL25, FLF | AGAMOUS-like 25, FLOWERING LOCUS F, REDUCED STEM BRANCHING 6, FLOWERING LOCUS C | chr5:3173724-3179339 REVERSE LENGTH=591

MGRKKLEIKRIENKSSRQVTFSKRRNGLIEKARQLSVLCDASVALLVVSASGKLYSFSSGDNLVKILDRYGKQHADDLKALDHQSKALNYGSHYELLELV

DSKLVGSNVKNVSIDALVQLEEHLETALSVTRAKKTELMLKLVENLKEKEKMLKEENQVLASQMENNHHVGAEAEMEMSPAGQISDNLPVTLPLLN*

>AT5G13790.1 | Symbols: AGL15 | AGAMOUS-like 15 | chr5:4449128-4450802 REVERSE LENGTH=807

MGRGKIEIKRIENANSRQVTFSKRRSGLLKKARELSVLCDAEVAVIVFSKSGKLFEYSSTGMKQTLSRYGNHQSSSASKAEEDCAEVDILKDQLSKLQEK

HLQLQGKGLNPLTFKELQSLEQQLYHALITVRERKERLLTNQLEESRLKEQRAELENETLRRQVQELRSFLPSFTHYVPSYIKCFAIDPKNALINHDSKC

SLQNTDSDTTLQLGLPGEAHDRRTNEGERESPSSDSVTTNTSSETAERGDQSSLANSPPEAKRQRFSV*

>AT5G15800.1 | Symbols: AGL2, SEP1 | SEPALLATA1, AGAMOUS-like 2 | chr5:5151594-5153767 REVERSE LENGTH=756

MGRGRVELKRIENKINRQVTFAKRRNGLLKKAYELSVLCDAEVALIIFSNRGKLYEFCSSSNMLKTLDRYQKCSYGSIEVNNKPAKELENSYREYLKLKG

RYENLQRQQRNLLGEDLGPLNSKELEQLERQLDGSLKQVRSIKTQYMLDQLSDLQNKEQMLLETNRALAMKLDDMIGVRSHHMGGGGGWEGGEQNVTYAH

HQAQSQGLYQPLECNPTLQMGYDNPVCSEQITATTQAQAQQGNGYIPGWML*

>AT5G20240.1 | Symbols: PI | PISTILLATA | chr5:6829203-6831208 FORWARD LENGTH=627

MGRGKIEIKRIENANNRVVTFSKRRNGLVKKAKEITVLCDAKVALIIFASNGKMIDYCCPSMDLGAMLDQYQKLSGKKLWDAKHENLSNEIDRIKKENDS

LQLELRHLKGEDIQSLNLKNLMAVEHAIEHGLDKVRDHQMEILISKRRNEKMMAEEQRQLTFQLQQQEMAIASNARGMMMRDHDGQFGYRVQPIQPNLQE

KIMSLVID*

>AT5G23260.1 | Symbols: TT16, ABS, AGL32 | TRANSPARENT TESTA16, ARABIDOPSIS BSISTER, AGAMOUS-like 32 | chr5:7836294-7838340 FORWARD LENGTH=792

MNIEEEGATHKRKKREMGRGKIEIKKIENQTARQVTFSKRRTGLIKKTRELSILCDAHIGLIVFSATGKLSEFCSEQNRMPQLIDRYLHTNGLRLPDHHD

DQEQLHHEMELLRRETCNLELRLRPFHGHDLASIPPNELDGLERQLEHSVLKVRERKQQLENLSRKRRMLEEDNNNMYRWLHEHRAAMEFQQAGIDTKPG

EYQQFIEQLQCYKPGEYQQFLEQQQQQPNSVLQLATLPSEIDPTYNLQLAQPNLQNDPTAQND*

>AT5G26580.1 | Symbols: AGL34 | AGAMOUS-like-34 | chr5:9393065-9394102 REVERSE LENGTH=1038

MGMKKVKLSLIANEISRETSFMKRKNGIMKKLYELSTLCGVQACTLIYSPFIPVPEFLEMSPTARTRKMMNQETYLMERITKAKEQLQNLVGANQELQVR

RFMFDCVEGKMSQYRYDAKDLQDLLSCINLYLDQLNGRIEILKEHGDSLPSVSPFPTRIGVEETGDESSSDSPILATTGVVDTPNATNPRVLVADTTHFL

DANATAVTAPFGFSNHIQYKNMNMSQDLHRPFQHLVPTNFCDFFQNQNMNQVQYQAPPNDMFNQIQREFYNINLNQKSNQYMNQQQPFMNPMVEQHMSHV

GGRESIPFMDGNYYNYNQLPVVDHGSTSYMPSTTGVYDPYFNNNL*

>AT5G26630.1 | Symbols: AGL35 | agamous-like 35 | chr5:9350815-9351471 FORWARD LENGTH=657

MTRQKVKMTFIENETARKSTFKKRKKGLLKKAQELGILCGVPIFAVVNSPYELNPEVWPSREAANQVVSQWKTMSVMDKTKKMVNQETFLQQRITKATES

WKKLRKENKELEMKNIMFDCLSGKTLVSSIEKTELRDFGYVIEQQLKDVNRRIEILKRNNEPSSALVPVAAPTTSSVMPVVEMGSSSVGFYDKVRDQIQI

TLNMKQTTNDLDLNKKQW*

>AT5G26650.1 | Symbols: AGL36 | AGAMOUS-like 36 | chr5:9343785-9344885 FORWARD LENGTH=1101

MKKVKLSLIANERSRKTSFIKRKDGIFKKLHELSTLCGVQACALIYSPFIPVPESWPSREGAKKVASRFLEMPPTARTKKMMDQETYLMERITKAKEQLK

NLAAENRELQVRRFMFDCVEGKMSQYHYDAKDLQDLQSCINLYLDQLNGRIESIKENGESLLSSVSPFPTRIGVDEIGDESFSDSPIHATTGVVDTLNAT

NPHVLTGDMTPFLDADATAVTASSRFFDHIPYENMNMSQNLHEPFQHLVPTNVCDFFQNQNMNQVQYQAPNNLFNQIQREFYNINLNLNLNLNSNQYLNQ

QQSFMNPMVEQHMNHVGGRESIPFVDGNCYNYHQLPSNQLPAVDHASTSYMPSTTGVYDPYINNNL*

>AT5G26880.1 | Symbols: AGL26 | AGAMOUS-like 26 | chr5:9457950-9459190 REVERSE LENGTH=792

MESCCRSVIASRTFHLRSSGRLFPSLSLTHLKGKLSLSINSFSSKIQSHALRGVGIGESDKKNPLPRGAGEGVKEDARSKLLHVVLVSPQIPGNTGCIAR

TCAASAVGLHLVGPLGFQVDDARVKRAGLDYWPFVVVKAHSSWAEFQEYFRLQEGEKRMIAFTKRGTRIHSDFSYRSGDYLLFGSETSGLPPEALSDCNH

EPYGGGTLRIPMVETYVRCLNLSVSVGIALYEASRQLNYEQIECAPQGCVNGEEPLLTEDIFA*

>AT5G26950.1 | Symbols: AGL93 | AGAMOUS-like 93 | chr5:9483251-9484120 REVERSE LENGTH=870

MDSSMSTKKKTKLSVRNQTCFKKSSLSSSSTAKKTTNLSMREQTMFKKALELSTLCNIDVCVIYYGRDGKLIKTWPDDQSKVRDMAERFSRLHERERCKK

RTNLSLFLRKKILDDTKLSEKVLEMEDSLESGLRVLQDKLLLLQPEKNQTEFGQTRAVSSTTNPLSPPPSLIEDHRHQQRTEPLMSGVSNTEQDLSTSSL

SQNQSKFSVFLYNHDNCSFYQVPDSVSSFDSLTSTGLLGEQGSGLGSSFDLPMVFPPQMQTQTPLVPFDQFAPWNQAPSFADPMMFPYN*

>AT5G27050.1 | Symbols: AGL101 | AGAMOUS-like 101 | chr5:9520276-9520638 FORWARD LENGTH=363

MFKKALELSTLCNIEVCVIYYGRDGELFKTWPEDESKVRDMAERFTKLNERERRKKRTNLSLFLRKKILDDNKLSGKVLEMKDSLERGLRVLQDKLLLLQ

PENQTKSLTRSVSSLDYVFV*

>AT5G27070.1 | Symbols: AGL53 | AGAMOUS-like 53 | chr5:9527741-9528604 FORWARD LENGTH=864

MDSSMSTKKKTKLSVRNQTCFKKSSLSSSSTAKKTTNLSMREQTMFKKALELSTLCNIDVCVIYYGRDGKLIKTWPEDQSKVRDMAERFSRLHERERCKK

RTNLSLFLRKKILDDTKLSEKVLEMEDSLESGLRVLQDKLLLLQPEKNQTEFGQTRAVSSTTNPLSPPPSLIEDHRHQQWTEPLMSGVSNTEQDLSTSSL

SQNQSRISVFLYNHDNRSFYQVPDSVSSFDQSALLGEQGSGLGSNFDLPPMVFPPQMQTQTPLVPFDQFAAWNQAPSFADPMMFPYN*

>AT5G27090.1 | Symbols: AGL54 | AGAMOUS-like 54 | chr5:9531845-9532408 FORWARD LENGTH=564

MDSSTSTKKNTKLFVRNQTCFKKSSLSSSNAKKTTNLSMREQTMFKKALELSTLCDIEVCVIYYGRDGKLIKTWPEDQSKVRDMAERFSRLHERERCKKR

TNLSLFLRKQILHDKKLSEKVLEMEDSLESGLRVLQDKLLLLQPEKNQTELGQSCAVYSTTYPLSSPSLIEDHQHQQQWTEPLSNTE*

>AT5G27130.1 | Symbols: AGL39 | AGAMOUS-like 39 | chr5:9546633-9547553 FORWARD LENGTH=921

MPSSDSTMMKKGTKRKIEIKKRETKEQRAVTCSKRRQTVFSKAADLCLISGANIAVFVTSPSDSSDVVYSFSGYSSAYEIADCYLNRKPPPKIVNPAGSK

LGFWWEDPDLYHSCDDLSELSIIEDRLQRMKKHVMACLEKEEKSQLVSSFDQNPNSTCSLDVEDCDGSSYSQIASTFTPNSVNEYCSDQTFSSFHGDQNP

NLSSPSFDQDCYSSLYQICGESSSQVASFDQNPSSEIQGFETEEEINQINLLLQETQTEANVNLDDEICFWNDLSNDDVFGLNSYFGLDNTNAMINFGDS

DFRRHV*

>AT5G27580.1 | Symbols: AGL89 | AGAMOUS-like 89 | chr5:9736651-9737322 FORWARD LENGTH=672

MDSSMSTKKKTKLSVRNQTCFKKSSLSSSSTAKKTTNLSMREETMFKKALELSTLCDIEVCVIYYGRDGELIKTWPEDQSKVRDMAERFSKLHERERRKK

RTNLSLFLRKKILDDNKLSEKVLEMKDSLESGLRVLQDKLLLLQPENQTELGQSRAVSSTTNPLSSPEDHHHQQWTEPLVTGVSNTEQDLSTSPLSNHQS

KYSVFVYNHDSGSFYQVPDSICF*

>AT5G27810.1 | Symbols: no symbol available | no full name available | chr5:9855827-9856186 FORWARD LENGTH=360

MKKVHELSTLCGITSCAIIYSPYDTSHEVWPSNSGVQRVVSEFRTLPEMDQHKKMVDQEGFLKQRIAKPTENLRRQRKDNKELEMTEVMFRCLIGNMEML

KSESQSESTTMVYENDEPS*

>AT5G27944.1 | Symbols: no symbol available | no full name available | chr5:9975918-9976592 REVERSE LENGTH=675

MYSSMSTKKKTKLSVRNQTCFKKSSLSSSSTAKKTTNLSMREQTMFKKALELSTLCDIEVCVILYGRDGELIKTWPEDQSKVRDMAERFSRLHERERCKK

RTNLSLFLRKKILDDNKLSEKVLEMEDSLESGLRVLQNKLLLLQPEKNQTKLGQSRAVSSTTNLLSSPENHHNQQWTEPLENGVSNTEQELSTSSLSQHQ

SKYSVFLYNHDNGSFYQVPDSICF*

>AT5G27960.1 | Symbols: AGL90 | AGAMOUS-like 90 | chr5:9991685-9992770 REVERSE LENGTH=963

MKKVKLSLIANERSRKTSFMKRKNGIFKKLHELSTLCGVQACALIYSPFIPVPESWPSREGAKKVASKFLEMPRTARTRKMMDQETHLMERITKAKEQLK

NLAAENRELQVRRFMFDCVEGKMSQYRYDAKDLQDLLSCMNLYLDQLNGRIESIKENGESLLSSVSPFPTRIGVDEIGDESFSDSPIHSTTRVVDTPNAT

NPHVLAGDMTPFLDADANANMNQVQYQAPNNLFNQIQREFYNINLNLNLNLNSNQYLNQQQSFMNPMVEQHMNHVGGRESIPFVDRNYYNYNQLPAVDLA

STSYMPSTTDVYDPYINNNL*

>AT5G37415.1 | Symbols: AGL105 | AGAMOUS-like 105 | chr5:14839047-14840301 REVERSE LENGTH=951

MRDPDIVQNTRSLILLRVLTHEKLIQSHRYFFVGWIHKNLSRFRRKIRRRFLHCDFSGFGFFPRRFLFGFVLEKMDHLQESFRGSELCILCDIEACVIYY

GPDGELKTWPKEREKVEDIALRYSQLNEALRRKKSVTLYDFLNKKKDKTNLEKKAMITDNDDLKTCLKNVNVLKSPIADHYFNDQISQLIQSLEPHVSKV

QERIRFVESQKHKETKLDHQSLASIYSLNQSLNPSQFTLFLYNHGDNTMSQIPNMFMNNNNFQHSFVSNTQDYSALQESVNNNYGLMPNVLCGYDQNLFT

SDITNNNLLIDNSMYL*

>AT5G38620.1 | Symbols: AGL73 | agamous-like 73 | chr5:15463858-15464907 REVERSE LENGTH=1050

MVKGTKRKIAIETIQKRDSLRVTCTKRRKGLYSKASQLCLLSDAQIAILATPPSSESDVSFYSFGHSSVDAVVSAFLSGKRPVSAPKDNKETREDVGICL

TRKNLGLGFWWNDESLVRSENPQEISEAIGSMWTLLSNLKELRADEACVNDHKDLKKNEKSDVHGTQDQTLIFQSASAVCCIPENLNDITQEPNQTLDIQ

SSSSAICCVPDKSPEIFNEITEEQDQILSICETFCVTDNNNNNNNNAALPEVNLYYNQDMAIDQLIDFNTPFESSIDDWFSDNTTHQETTSASILNDVGV

DDQVSVDTNPFSYFQSLEDADLVFQRCLDGDNLRFSDCFNDFANTIAAV*

>AT5G38740.1 | Symbols: AGL77 | AGAMOUS-like 77 | chr5:15513025-15514305 REVERSE LENGTH=1281

MTTIRSSPSSSRCSNSSSSSSYSLASTSLSNRLETIFKKASELCTLCDIEACVIYYGPDGELKTWPKEREKVRDIALRFNQLNEALRHKKSVNLHGFLNK

KKKNKGLKNPNKKKKTSLKNVNVLKYPLADHYSPDQVSQLTQSLELNVSKFQERLRFLESQKQNETKPDHQSLTSISSLNQSLNPSQFSLFMYNHGYNTL

SQIPVSASNFNQDYISALLEQSELKSQIMKQEVCGYEQNMCMSNHGDATLSQIPFSASNFNQDFSANNNFQHSFVSNTQDYYSVQKSVNNNYGLKNQLMK

HDLCGYEHNMCMSNHGDATFSQIPLSASNFNQDFSVSIQEESGLMQQELCGYDQNQNMSMGDITNNNFQVTCASVLESVNNFGLNQLMHKEFYGCHQNMS

MGNINNNSFQHPWVSNADHTRRYKNL*

>AT5G39750.1 | Symbols: EMB3008, AGL81 | EMBRYO DEFECTIVE 3008, AGAMOUS-like 81 | chr5:15906875-15907942 FORWARD LENGTH=1068

MAIRSLPSSSRCSSSSSSSSYSLASTSLSNRLETIFKKASELCTLCDIEACVIYYGPDGELKTWPPEREKVEDIALRYSQLNEALRRKKSVTLYDFLNKK

KDKTNLEKKAKITDNDDLKTCLKNVNILKYPLADHYSPDQVSQLIQSLEPHVSKVRERIRFVESQKHKETKPDHQSLASSSLNHQTQSLNPSQFSLFMYN

HGDNTLSQIPVSASNFNQDYFSALLEQSELKSQIMKQDLCGYEQNMCMSNHGDATLSQIPLSASNLNQDFSALLQDESGLMQQELCGYDQNMFMNNNNFQ

HSFVSNTQDHSAPVVQESVNNNYGLMPHVPCGYDQNLFTSDITNNNLLINNSMFL*

>AT5G39810.1 | Symbols: AGL98 | AGAMOUS-like 98 | chr5:15937278-15938344 REVERSE LENGTH=990

MAIRSLPSSSGCSNSSSSSSYSLASTSLSNRLETIFRKASELCTLCDIEACVIYYGPDGELKTWPPEREKVEDIALRYSQLNEALRRKKSVTLYDFLNKK

KNKTNLEKKAKIKDNDLKRLSLEPHVSKVRERIRFVESQKHKETKPDHQSLASSSLNNQTQSLNPSQFSLFMYNHGDNILSQIPVSASNFNQDYFSALLE

QSELKSQIMKQEVCGYEQNMCMSNNGDATLSQIPLSASNFNQEFSALLQEESGLMQQELCNYDQNMFMNNNNFQHSFVSNTQDHSAPAVQESVNNNYGLM

PHVPCGYDQNLFTSDITNNNLLIDNSMFL*

>AT5G40120.1 | Symbols: AGL76 | AGAMOUS-like 76 | chr5:16051879-16053036 FORWARD LENGTH=1158

MTMRSLPFSSSSYSLASTSLSNRLETIFKKASELCTLCDIEACVIYYGPDGELKTWPKEKEKVRDIALRYSQLNEALRSKKSVNLHGFLNKKKKKKKKGL

KNPNNKRKTCLKKNVNVLKYPLADHYPPDQVSQLTQSLKLHVSKFQERLRFLESQKQTKPDHQSLTPSSLNHQTQSLNPRQFSLFMYNHGDNTLSQISVS

ASNFNQNYFSALLEQSELKNQLMKQDGYDQNQNMRMGDITNNNFQLPYFSKKEAVQESVNYFGMNQLMLKELYGCDQNMCMGNINSNSFQHPCVSKAQHY

SAVEGSVNNQRQSELMQQELCGYEQNMCFTNNNFQVSNKEAVQESVTNFGLMQHELYGCDQNMSMGNIINNSFQQRLKHRTRICE*

>AT5G40220.1 | Symbols: AGL43 | AGAMOUS-like 43 | chr5:16078390-16079364 REVERSE LENGTH=975

MTMRSSLPSSSSAYSLASTSLSNRLETIFKKASELCTLCDIEACVIYYGPDGELKTWPPEREKVRDIALRYSQLNEALRRKKSVNLHGFLNKKKKNKGLK

NTDKKRKTSLKKVNVLKYPLADHYPPDQVSPLIQSLELHVSKFHERLEFLESRKQNETQPDHHSLASSSLNHQTQSLNPSQFSLFMYNHGDNTLSQIPVS

ASNFNQDYFSALLEQSELKNQLMKQEICGNDQNQNMWMGNITNNNFQLPCVSVQESVNNFGLMHKEFYGCDHNMSVGNINSNSCEHPCVSSTQHYSAVEE

SVNNPWLNQLMQNELYGYGYADFC*

>AT5G41200.1 | Symbols: AGL75 | AGAMOUS-like 75 | chr5:16490544-16491536 FORWARD LENGTH=993

MTMRSSSPSSSSSYSLAFTSLSNRLETIFKKASELCTLCDIEACVIYYGPDGELKTWPKEKEKVRDIALRYSLLNEALRRKKSVNLHGFLNKKKNKGLKN

PNKKMKTSLKNVNILKYPLADHYPPDQVSPLIQSLELHVSKFQERLRFLESQKQNQTKPDHQSLTPSSLNHYTQSLNPSQFSLFMYNHGDNTLSQIPVSA

SNFNQDYFSALLEESELKNQLMKPEICGYDQNQNMSMGDITNNKFQDPCVSNKEAVQESVNNFGLNQLMYKEFYGCDQNMSMGNINSNSFQNPCVSNTQH

YSAVEESVKNPWLNQLMQNELYGYGYAGFC*

>AT5G48670.1 | Symbols: FEM111, AGL80 | AGAMOUS-like 80 | chr5:19738825-19739790 REVERSE LENGTH=966

MTRKKVKLAYISNDSSRKATFKKRKKGLMKKVHELSTLCGITACAIIYSPYDTNPEVWPSNSGVQRVVSEFRTLPEMDQHKKMVDQEGFLKQRIAKATET

LRRQRKDSRELEMTEVMFQCLIGNMEMFHLNIVDLNDLGYMIEQYLKDVNRRIEILRNSGTEIGESSSVAVAASEGNIPMPNLVATTAPTTTIYEVGSSS

SFAAVANFVNPIDLQQFRHPAAQHVGLNEQPQNLNLNLNQNYNQNQEWFMEMMNHPEQMRYQTEQMGYQFMDDNHHNHIHHQPQEHQHQIHDESSNALDA

ANSSSIIPVTSSSITNKTWFH*

>AT5G49420.1 | Symbols: AGL84 | agamous-like 84 | chr5:20035166-20036170 REVERSE LENGTH=1005

MVKKGGTKRKIAIETIQKRDSLRVTCTKRREGLYSKASQLCLLSDAQIAILATPPSSESNVSFYSFGHSSVDAVVSAFLSGQRPVPKDNKETREDVGICL

TRNNLGLGFWWNDESLARSENPQEISEAIDSMRTLLRNLKELRADEALACNQAFVNDREDLKNNDKCDFVSDHETHDQTLILQSASPICCIPENLNEITQ

EPNQTLNIQSSTSAICCVPDNSPENFNEITEEQDQIRSICETFCVMDNNAALPEMNLDYDQDIGFDTPFESALNDWFSDNTTHQEISASILNAVVDDQVS

VDLTPFSYFQRCLDGDNLRFSDCFKDFANTISAL*

>AT5G49490.1 | Symbols: AGL83 | AGAMOUS-like 83 | chr5:20075328-20076185 FORWARD LENGTH=858

MRFVPYLYEIERLWLSLVNYLSPRKNKNRRCGEIDKIRMVKKGGTKRKIAIETIQKSDYLRVTCTKRREGLFSKASQLCLLSDAQIAILATPPTSESNIS

FYSFGHSSVDAVVSSFLSGQRCVPLQEDTKEMREDVAICLSRTNLGLGFWWNNESLNKSENPQEISDAINSMLTLLSNLKELSGEEALVNDHKDLKKNER

SDVVLQHGTQYETLNPNSNTTTICCVPDELPANSNEIVGISPNPLIMLEKKKSQIEEKFEKEWQVSVTRIENEATSSYAKRRRSI*

>AT5G51860.1 | Symbols: AGL72 | AGAMOUS-like 72 | chr5:21081844-21084126 REVERSE LENGTH=636

MVRGKIEIKKIENVTSRQVTFSKRRSGLFKKAHELSVLCDAQVAAMIFSQKGRLYEFASSDIRNTIKRYAEYKREYFVAETHPIEQYVQGLKKEMVTMVK

KIEVLEVHNRKMMGQSLDSCSVKELSEIATQIEKSLHMVRLRKAKLYEDELQKLKAKERELKDERVRLSLKKTIYTHLCQVGERPMGMPSGSKEKEDVET

DLFIGFLKNRP*

>AT5G51870.1 | Symbols: AGL71 | AGAMOUS-like 71 | chr5:21085635-21087923 REVERSE LENGTH=624

MVRGKIEIKKIENVTSRQVTFSKRRSGLFKKAHELSVLCDAQVAAIVFSQSGRLHEYSSSQMEKIIDRYGKFSNAFYVAERPQVERYLQELKMEIDRMVK

KIDLLEVHHRKLLGQGLDSCSVTELQEIDTQIEKSLRIVRSRKAELYADQLKKLKEKERELLNERKRLLEEVNMHHSSKGNTEGGHRTKHSSEVETDLFI

GLPVTRL*

>AT5G55690.1 | Symbols: AGL47 | agamous-like 47 | chr5:22548790-22549623 REVERSE LENGTH=834

MGRKMVKMTRITNEKTRITTYKKRKACLYKKASEFSTLCGVDTCVIVYGPSRAGDEMVMEPELWPKDGSKVREILTKYRDTASSSCTKTYTVQECLEKNN

TKVEKPTIATKYPTWDKKLDQCSLNDLYAVFMAVENKIQEATNRNQTFPDTSCWSNDQLGLCGYNRQCFEQYQLFPLPTMDYNGLSFFPFNNQMTSNTAE

VSSFSNVTEPMIANGQSLFYGSCSDGPYGPMVQRTAYMEPIHWGLGNSMFNNVKQFQDYPFRFAQVNDLEDSSKLSM*

>AT5G58890.1 | Symbols: AGL82 | AGAMOUS-like 82 | chr5:23780832-23781716 FORWARD LENGTH=885

MVPKVVDLQRIANDKTRITTYKKRKASLYKKAQEFSTLCGVETCLIVYGPTKATDVVISEPEIWPKDETKVRAIIRKYKDTVSTSCRKETNVETFVNDVG

KGNEVVTKKRVKRENKYSSWEEKLDKCSREQLHGIFCAVDSKLNEAVTRQERSMFRVNHQAMDTPFPQNLMDQQFMPQYFHEQPQFQGFPNNFNNMGFSL

ISPHDGQIQMDPNLMEKWTDLALTQSLMMSKGNDGTQFMQRQEQPYYNREQVVSRSAGFNVNPFMGYQVPFNIPNWRLSGNQVENWELSGKKTI*

>AT5G60440.1 | Symbols: AGL62 | AGAMOUS-like 62 | chr5:24306329-24307520 FORWARD LENGTH=900

MVKKSKGRQKIEMVKMKNESNLQVTFSKRRSGLFKKASELCTLCGAEVAIVVFSPGRKVFSFGHPNVDSVIDRFINNNPLPPHQHNNMQLRETRRNSIVQ

DLNNHLTQVLSQLETEKKKYDELKKIREKTKALGNWWEDPVEELALSQLEGFKGNLENLKKVVTVEASRFFQANVPNFYVGSSSNNAAFGIDDGSHINPD

MDLFSQRRMMDINAFNYNQNQIHPNHALPPFGNNAYGINEGFVPEYNVNFRPEYNPNQNQIQNQNQVQIQIQNQSFKRENISEYEHHHGYPPQSRSDYY*

>AT5G60910.1 | Symbols: FUL, AGL8 | FRUITFULL, AGAMOUS-like 8 | chr5:24502736-24506013 REVERSE LENGTH=729

MGRGRVQLKRIENKINRQVTFSKRRSGLLKKAHEISVLCDAEVALIVFSSKGKLFEYSTDSCMERILERYDRYLYSDKQLVGRDVSQSENWVLEHAKLKA

RVEVLEKNKRNFMGEDLDSLSLKELQSLEHQLDAAIKSIRSRKNQAMFESISALQKKDKALQDHNNSLLKKIKEREKKTGQQEGQLVQCSNSSSVLLPQY

CVTSSRDGFVERVGGENGGASSLTEPNSLLPAWMLRPTTTNE*

>AT5G62165.1 | Symbols: AGL42, FYF | AGAMOUS-like 42, FOREVER YOUNG FLOWER | chr5:24965075-24968437 FORWARD LENGTH=633

MVRGKIEMKKIENATSRQVTFSKRRNGLLKKAYELSVLCDAQLSLIIFSQRGRLYEFSSSDMQKTIERYRKYTKDHETSNHDSQIHLQQLKQEASHMITK

IELLEFHKRKLLGQGIASCSLEELQEIDSQLQRSLGKVRERKAQLFKEQLEKLKAKEKQLLEENVKLHQKNVINPWRGSSTDQQQEKYKVIDLNLEVETD

LFIGLPNRNC*

>AT5G65050.1 | Symbols: MAF2, AGL31 | AGAMOUS-like 31, MADS AFFECTING FLOWERING 2 | chr5:25982415-25986114 FORWARD LENGTH=549

MGRKKVEIKRIENKSSRQVTFSKRRNGLIEKARQLSILCESSIAVLVVSGSGKLYKSASGDNMSKIIDRYEIHHADELEALDLAEKTRNYLPLKELLEIV

QSKLEESNVDNASVDTLISLEEQLETALSVTRARKTELMMGEVKSLQKTVGKKTFLVIEGDRGMSWENGSGNKVRETLPLLK*

>AT5G65060.1 | Symbols: AGL70, FCL3, MAF3 | AGAMOUS-like 70, MADS AFFECTING FLOWERING 3 | chr5:25987527-25991065 FORWARD LENGTH=591

MGRRKVEIKRIENKSSRQVTFSKRRKGLIEKARQLSILCESSIAVVAVSGSGKLYDSASGDNMSKIIDRYEIHHADELKALDLAEKIRNYLPHKELLEIV

QSKLEESNVDNVSVDSLISMEEQLETALSVIRAKKTELMMEDMKSLQEREKLLIEENQILASQVGKKTFLVIEGDRGMSRENGSGNKVPETLSLLK*

>AT5G65070.1 | Symbols: MAF4, FCL4, AGL69 | AGAMOUS-like 69, MADS AFFECTING FLOWERING 4 | chr5:25992310-25995930 FORWARD LENGTH=603

MGRRKVEIKRIENKSSRQVTFCKRRNGLMEKARQLSILCESSVALIIISATGRLYSFSSGDSMAKILSRYELEQADDLKTLDLEEKTLNYLSHKELLETI

QCKIEEAKSDNVSIDCLKSLEEQLKTALSVTRARKTELMMELVKTHQEKEKLLREENQSLTNQLIKMGKMKKSVEAEDARAMSPESSSDNKPPETLLLLK

*

>AT5G65080.1 | Symbols: MAF5, AGL68 | AGAMOUS-like 68, MADS AFFECTING FLOWERING 5 | chr5:26000866-26002211 FORWARD LENGTH=354

DLEDKTQDYLSHKELLEIVQRKIEEAKGDNVSIESLISMEEQLKSALSVIRARKTELLMELVKNLQDKEKLLKEKNKVLASEVGKLKKILETGDERAVMS

PENSSGHSPPETLPLLK*

>AT5G65330.1 | Symbols: AGL78 | AGAMOUS-like 78 | chr5:26110326-26111351 FORWARD LENGTH=1026

MKQASSSSSSRNSTSLTNRLKTIFKKAEELSILCAIEVCVIYYGPDGELRTWPKERETVKDMALRYKEARKRKKSRNLHEFLEKEKDKDKGKTNLKKNWY

PNFDHYSPQQLSQLIQSLERTLSTLQERLRIVEAQKLQNTNLVHQSLTPSYLNQTQHLNPSKFSLFMYNHGDATLSQLPLSAPHSNQLINYQNHLMQHGF

GQNMCSDNITNNNFEHPGVSNTQDYSPLLSVQASAVNNYGLNNHLMQQQDQLHGFDQNMCMVSEIINNNNGLQHPNLSNTVPHEFSSDFNQNPYGNAVGN

ISFSQDMFSSYDASSLLQTSSLPPLHNIPSSYCFPGNSRLL*

#nucleotide sequences of MADS-box genes in Oryza sativa

>LOC_Os03g11614.1 Oryza sativa subsp. japonica|MIKC_MADS|MIKC_MADS family protein

ATGGGGAGGGGGAAGGTGGAGCTGAAGCGGATCGAGAACAAGATCAGCCGGCAGGTGACGTTCGCCAAGCGCAGGAACGGCCTGCTCAAGAAGGCCTACG

AGCTCTCCCTCCTCTGCGACGCCGAGGTCGCCCTCATCATCTTCTCCGGCCGCGGCCGCCTCTTCGAGTTCTCCAGCTCATCATGCATGTACAAAACCTT

GGAGAGGTACCGCAGCTGCAACTACAACTCACAGGATGCAGCAGCTCCAGAAAACGAAATTAATTACCAAGAATACCTGAAGCTGAAAACAAGAGTTGAA

TTTCTTCAAACCACACAGAGAAATATTCTTGGTGAGGATTTGGGCCCACTAAGCATGAAGGAGCTGGAGCAGCTTGAGAACCAGATAGAAGTATCCCTCA

AACAAATCAGGTCAAGAAAGAACCAAGCACTGCTTGATCAGCTGTTTGATCTGAAGAGCAAGGAGCAACAGCTGCAAGATCTCAACAAAGACTTGAGGAA

AAAGTTACAGGAAACCAGTGCAGAGAATGTGCTCCATATGTCCTGGCAAGATGGTGGTGGGCACAGCGGTTCTAGCACTGTTCTTGCTGATCAGCCTCAT

CACCATCAGGGTCTTCTCCACCCTCACCCAGATCAGGGTGACCATTCCCTGCAGATTGGGTATCATCACCCTCATGCTCACCATCACCAGGCCTACATGG

ACCATCTGAGCAATGAAGCAGCAGACATGGTTGCTCATCACCCCAATGAACACATCCCATCCGGCTGGATATGA

>LOC_Os12g10540.1 Oryza sativa subsp. japonica|MIKC_MADS|MIKC_MADS family protein

ATGGGGAGGGGCAGGATTGAGATCAAGAGGATCGAGAACACGACAAGCCGCCAGGTGACCTTCTGCAAGCGCCGCAACGGACTTCTCAAGAAGGCGTATG

AGCTCTCCGTCCTCTGCGATGCCGAGGTGGCTCTCATCGTCTTCTCCAGCCGTGGCCGCCTCTACGAGTACTCCAACAACAACAATGTGAAGGCTACAAT

TGACAGGTACAAGAAGGCGCATGCTTGTGGCTCAACTTCTGGTGCACCTCTCATAGAGGTCAATGCTCAGCAATACTACCAGCAGGAGTCTGCCAAACTG

CGCCACCAGATTCAGATGCTGCAAAACACCAACAAGCACCTGGTTGGCGATAATGTGAGCAACCTGTCACTGAAGGAGCTGAAGCAACTTGAAAGCCGCC

TGGAGAAAGGCATTTCAAAGATCAGAGCCAGGAAGAATGAACTGCTGGCTTCAGAGATCAATTACATGGCCAAAAGGGAGATTGAGCTTCAGAACGACAA

CATGGACCTCAGAACCAAGATTGCTGAGGAGGAGCAGCAGCTGCAGCAGGTGACGGTGGCCCGGTCGGCCGCCATGGAGCTGCAGGCTGCGGCGGCGGCG

CAGCAGCAGCAGCAGAATCCGTTCGCGGTGGCGGCGGCGCAGCTGGACATGAAGTGCTTCTTCCCGTTGAACCTGTTCGAGGCGGCGGCGCAGGTGCAGG

CCGTGGCGGCGCAGCGCCAGCAGATCATCCCCACCGAGCTCAACCTCGGCTACCACCACCACCTTGCCATTCCCGGCGCCGCCGCCGCCGACGCGCCGCC

TCCTCACTTCTGA

>LOC_Os03g54160.1 Oryza sativa subsp. japonica|MIKC_MADS|MIKC_MADS family protein

ATGGGGCGGGGCAAGGTGCAGCTGAAGCGGATCGAGAACAAGATCAACCGGCAGGTGACCTTCTCCAAGCGCAGGTCGGGGCTGCTCAAGAAGGCGAATG

AGATCTCCGTGCTCTGCGACGCCGAGGTCGCGCTCATCATCTTCTCCACCAAGGGCAAGCTCTACGAGTACGCCACCGACTCATGTATGGACAAAATCCT

TGAACGTTATGAGCGCTACTCCTATGCAGAAAAGGTCCTTATTTCAGCTGAATCTGACACTCAGGGCAACTGGTGCCACGAATATAGGAAACTGAAGGCT

AAGGTTGAGACAATACAGAAATGTCAAAAGCACCTCATGGGAGAGGATCTTGAATCTTTGAATCTCAAAGAGCTGCAGCAGCTGGAGCAGCAGCTGGAAA

ATTCGTTGAAACATATCAGATCCAGAAAGAGCCAACTAATGCTCGAGTCCATTAACGAGCTTCAACGGAAGGAAAAGTCACTGCAGGAGGAGAATAAGGT

CCTACAGAAAGAAAACCCTTGCTCCTTCCTACAGCTGGTGGAGAAGCAGAAAGTCCAGAAGCAACAAGTGCAATGGGACCAGACACAACCTCAAACAAGT

TCCTCATCATCCTCCTTCATGATGAGGGAAGCCCTTCCAACAACTAATATCAGTAACTACCCTGCAGCAGCTGGCGAAAGGATAGAGGATGTAGCAGCAG

GGCAGCCACAGCATGTTCGCATTGGGCTGCCACCATGGATGCTGAGCCACATCAACGGCTAA

>LOC_Os07g01820.1 Oryza sativa subsp. japonica|MIKC_MADS|MIKC_MADS family protein

ATGGGGCGGGGGAAGGTGCAGCTGAAGCGGATAGAGAACAAGATCAACAGGCAGGTGACGTTCTCCAAGAGGAGGAATGGATTGCTGAAGAAGGCGCACG

AGATCTCCGTCCTCTGCGACGCCGAGGTCGCCGCCATCGTCTTCTCCCCCAAGGGCAAGCTCTACGAGTACGCCACTGACTCCAGGATGGACAAAATCCT

TGAACGTTATGAGCGCTATTCATATGCTGAAAAGGCTCTTATTTCAGCTGAATCCGAGAGTGAGATAACTCTTCCCCAGTTGACAACATGCACTGCATCC

AGAAGTACTCATGGTATTTGTTTTCAGTACTGTTTGATGAGTAAAACTTTGGGAAATTGGTGCCATGAATACAGGAAACTTAAGGCAAAGATTGAGACCA

TACAAAAATGTCACAAACACCTCATGGGAGAGGATCTAGAATCCCTGAATCTCAAAGAACTCCAACAGCTAGAGCAGCAGCTGGAGAGTTCATTGAAGCA

CATAATATCAAGAAAGAGCCACCTTATGCTTGAGTCCATTTCCGAGCTGCAGAAAAAGGAGAGGTCACTGCAGGAGGAGAACAAGGCTCTGCAGAAGGAA

CTGGTGGAGAGGCAGAAGAATGTGAGGGGCCAGCAGCAAGTAGGGCAGTGGGACCAAACCCAGGTCCAGGCCCAGGCCCAAGCCCAACCCCAAGCCCAGA

CAAGCTCCTCCTCCTCCTCCATGCTGAGGGATCAGCAGGCACTTCTTCCACCACAAAATATCTGCTACCCGCCGGTGATGATGGGCGAGAGAAATGATGC

GGCGGCGGCGGCGGCGGTGGCGGCGCAGGGCCAGGTGCAACTCCGCATCGGAGGTCTTCCGCCATGGATGCTGAGCCACCTCAATGCTTAA

>LOC_Os06g49840.1 Oryza sativa subsp. japonica|MIKC_MADS|MIKC_MADS family protein

ATGGGGAGGGGCAAGATCGAGATCAAGCGGATCGAGAACGCGACCAACAGGCAGGTGACCTACTCGAAGCGCCGCACGGGGATCATGAAGAAGGCCAGGG

AGCTCACCGTGCTCTGCGACGCCCAGGTCGCCATCATCATGTTCTCCTCCACCGGCAAGTACCACGAGTTCTGCAGCCCTTCCACCGACATCAAGGGGAT

CTTTGACCGCTACCAGCAAGCCATCGGCACCAGCCTTTGGATCGAGCAGTATGAGAATATGCAGCGCACGCTGAGCCATCTCAAGGACATCAACCGCAAC

CTGCGCACCGAGATCAGGCAAAGGATGGGAGAAGATCTGGACGGGCTGGAGTTCGACGAGCTGCGCGGTCTTGAGCAAAATGTCGATGCCGCCCTCAAGG

AGGTTCGCCACAGGAAGTATCATGTGATCACCACACAGACTGAAACCTACAAGAAAAAGGTGAAGCACTCCTACGAGGCGTACGAGACTCTGCAGCAGGA

GCTGGGGTTGCGCGAGGAGCCGGCGTTCGGGTTCGTGGACAACACCGGCGGCGGGTGGGACGGCGGCGCCGGCGCCGGCGCGGCGGCGGACATGTTCGCC

TTCCGCGTGGTGCCCAGCCAGCCCAACCTGCACGGCATGGCCTACGGCGGCAACCACGACCTGCGCCTCGGTTGA

>LOC_Os04g49150.1 Oryza sativa subsp. japonica|MIKC_MADS|MIKC_MADS family protein

ATGGATCGATCAGAGATGGGGAGGGGAAGGGTTGAGCTGAAGCGCATCGAGAACAAGATCAACCGGCAGGTCACCTTCTCCAAGCGCCGCAACGGCCTCC

TCAAGAAGGCGTACGAGCTCTCCGTGCTCTGCGACGCGGAGGTGGCGCTCATCATCTTCTCCAGCCGTGGCAAGCTGTACGAATTCGGTAGCGCCGGAAT

AAACAAGACATTGGAAAAGTACAATAGTTGCTGTTACAACGCTCAAGGTTCAAATAGTGCTCTTGCTGGTGGTGAACATCAGAGCTGGTACCAAGAGATG

TCAAGGCTCAAGACTAAGCTTGAATGTCTCCAACGCTCTCAGAGGCACATGCTTGGTGAAGATCTTGGACCATTGAGCATAAAGGAACTGCAGCAGCTGG

AGAAGCAACTTGAGTACTCACTGTCACAGGCTCGACAACGAAAGACACAAATCATGATGGAGCAGGTCGACGATCTTCGCCGGAAGGAACGCCAGCTTGG

AGAGCTCAATAAGCAACTGAAAAACAAGCTAGAAGCTGAAGCCGATAGCAGCAACTGCAGATCAGCCATCCAGGATTCCTGGGTCCATGGCACCGTCGTC

AGTGGCGGCAGAGTGCTGAATGCTCAACCACCACCAGATATTGACTGTGAGCCTACTCTGCAAATTGGGTACTATCAATTTGTCCGTCCTGAGGCGGCCA

ATCCAAGAAGCAATGGAGGAGGAGGGGATCAGAACAACAACTTTGTGATGGGATGGCCCCTCTGA

>LOC_Os07g41370.1 Oryza sativa subsp. japonica|MIKC_MADS|MIKC_MADS family protein

ATGGGGAGAGGGCCGGTGCAGCTGCGGCGGATCGAGAACAAGATAAACAGGCAGGTGACCTTCTCCAAGCGGAGGAACGGGCTGCTGAAGAAGGCGCACG

AGATCTCCGTGCTCTGTGACGCCGACGTCGCGCTCATCGTCTTCTCCACCAAGGGCAAGCTCTACGAGTTCTCCAGCCACTCCAGTATGGAAGGGATCCT

TGAACGCTACCAGCGTTACTCGTTTGATGAAAGAGCCGTACTGGAGCCAAATACTGAGGACCAGGAAAACTGGGGTGATGAATATGGAATTTTGAAGTCC

AAACTGGATGCACTTCAGAAGAGCCAAAGGCAACTCTTAGGTGAACAATTGGACACACTAACAATAAAAGAACTCCAGCAATTGGAACATCAACTGGAAT

ATTCTCTGAAGCATATAAGATCAAAAAAGAATCAGCTTCTGTTTGAATCAATTTCTGAGCTTCAGAAGAAGGAAAAGTCACTTAAAAACCAGAATAATGT

TCTGCAAAAGCTCATGGAGACAGAAAAGGAGAAAAACAATGCTATAATAAACACTAACCGGGAGGAGCAAAATGGAGCAACACCAAGCACATCATCACCA

ACACCAGTGACGGCTCCAGATCCCATCCCGACAACAAATAACAGTCAAAGCCAACCAAGAGGATCAGGGGAGTCAGAAGCTCAACCGTCTCCGGCACAAG

CAGGCAACAGCAAGCTTCCGCCATGGATGCTCCGGACAAGTCACACATGA

>LOC_Os01g66030.1 Oryza sativa subsp. japonica|MIKC_MADS|MIKC_MADS family protein

ATGGGGCGCGGGAAGATCGAGATCAAGAGGATCGAGAACTCCACCAACCGCCAGGTGACCTTCTCCAAGCGCAGGAGCGGGATCCTCAAGAAGGCCCGCG

AGATCAGCGTCCTGTGCGACGCCGAGGTCGGCGTCGTCATCTTCTCCAGCGCCGGCAAGCTCTACGACTACTGCTCCCCCAAGACCTCGCTATCAAGAAT

CTTGGAGAAGTACCAGACCAATTCCGGAAAGATACTGTGGGATGAGAAGCACAAGAGCCTTAGCGCGGAGATTGATCGAATCAAGAAAGAGAACGATAAT

ATGCAGATTGAGCTCAGGCACTTGAAAGGTGAAGATCTAAACTCTCTGCAGCCCAAAGAGCTCATCATGATTGAGGAGGCACTTGACAATGGGATAGTGA

ACGTGAATGATAAACTGATGGACCACTGGGAAAGGCACGTGAGAACTGATAAGATGCTGGAAGACGAGAACAAGCTGCTGGCTTTTAAACTGCACCAGCA

AGATATAGCGCTGAGCGGGAGCATGAGGGATCTTGAGCTTGGGTACCATCCAGACAGGGACTTTGCGGCCCAGATGCCGATCACCTTCCGCGTGCAGCCC

AGCCACCCCAACCTGCAGGAGAACAATTAA

>LOC_Os12g31748.1 Oryza sativa subsp. japonica|MIKC_MADS|MIKC_MADS family protein

ATGGGGAGGGGGAAGGTGCAGGTGCGGCGGATCGAGAACGAGGTGAGCCGGCAGGTGACCTTCTCCAAGCGGCGGCCGGGGCTGCTCAAGAAGGCCCACG

AGATCGCCGTCCTCTGCGACGTCGACGTCGCCGCCATCGTCTTCTCCGCCAAGGGCAACCTCTTCCACTACGCCTCCTCCCACACCACTATGGAGCGAAT

CCTTGAGAAGTATGACAGACATGAGTTATTATCTGAAGGAAATAATGTGATTGAAGAGTTCCCTGAGCTGGAGGGAAGCATGAGCTATGACCACATCAAG

CTGAGGGGCAGGATTGAAGCTCTAAAAAAGAGCCAAAGGAATCTTATGGGGCAGGAACTTGACTCGCTGACACTGCAAGATATCCAGCAGCTTGAGAACC

AGATAGACACTTCTCTGAATAACATAAGATCAAGAAAGGAAAAGTTGCTGATGGAGAAAAACACTATTCTGGAGAAGAAAATTACTGAACTGGAGACACT

GCATACATGCATCAGGGCGTCACCCACTAAAGCTGCTGCTCCTCCTGCCTGCAATACTGCTGATGCATTTGTTCCCAACCTCAACATCTGCTGCGGCGAT

TCCGGCGAGCCGGAAACCGTGACGGCGCCACTTGGCTGGACCAGCAGCAACAATGGCTTGCCATGGTGGATGCTCCAGTCATCATCGAACGGCAAGAGCT

AG

>LOC_Os01g66290.1 Oryza sativa subsp. japonica|MIKC_MADS|MIKC_MADS family protein

ATGGGGAGGGGGAAGATTGAGATAAAGAGAATCGAGAACAAGACGAGCCGTCAGGTGACCTTCTGCAAGCGCAGGAATGGGCTGCTGAAGAAGGCCTACG

AGCTCGCCATACTCTGCGACGCTGAGATCGCACTCATCGTCTTCTCCAGCCGAGGACGCCTCTATGAGTTCTCCAATGTAAACAGCACAAGGTCAACAAT

CGAGAGGTACAAGAAAGCCTCTGCCAGCACTTCAGGATCCGCTCCAGTGATAGACGTCAATTCTCATCAATACTTTCAGCAAGAAGCAGCAAAAATGCGC

CACCAGATACAGACCTTGCAGAATGCAAACAGGCACCTCATCGGTGAGTCCATTGGCAATATGACCGCAAAGGAGCTCAAGAGCCTTGAAAATCGGCTTG

AAAAGGGCATCAGCCGAATTCGATCAAAGAAGCATGAGCTGCTGTTCTCGGAGATCGAATACATGCAGAAAAGGGAAGCAGATCTTCAGAACGAGAACAT

GTTCCTGAGAGCCAAGGTGGCAGAGGCCGAGCGAGCTGAACATGATGATCAGCAGGCGGCGGAGGACGACGAGATGGCGCCGGCGCCGGCGGTCGGCGGC

GGGTCATCGTCGGGGACAGAGCTGGAGGCGCTGCCGGCGACGTTCGACACAAGGGAATACTACCAGCCGGCGCCGCCGGTGAGCATGCTGGCCGCCGCGG

CGGCGGCGGCGGCGGCGCAGTACTCGTCGGACCACCACCAGACTGCTCTCCACCTCGGCTACTTCAAGGTCGACTCCGGCAAAGGCGGCCTCCTCTAG

>LOC_Os02g52340.1 Oryza sativa subsp. japonica|MIKC_MADS|MIKC_MADS family protein

ATGGCGCGGGAGAGGCGGGAGATAAAGAGGATAGAGAGCGCGGCGGCGCGGCAGGTCACCTTCTCCAAGCGCCGGCGCGGCCTGTTCAAGAAGGCCGAGG

AGCTCTCCGTGCTCTGCGACGCCGACGTCGCGCTCATCGTCTTCTCCTCCACGGGGAAGCTATCCCACTTCGCAAGCTCCAGTATGAATGAAATCATCGA

CAAGTACAACACACATTCTAATAATCTGGGGAAAGCAGAACAGCCTTCGCTCGACTTGAATTTAGAACATAGCAAGTATGCACATTTGAATGAGCAACTT

GCGGAAGCTAGTCTCCGGCTTAGACAAATGAGAGGTGAGGAGCTTGAGGGATTGAGCATTGATGAACTCCAGCAGCTAGAGAAGAACCTGGAAGCTGGTC

TGCACAGGGTGATGCTGACAAAGGATCAACAATTTATGGAGCAGATCAGTGAACTCCAGCGGAAGAGTTCACAGCTGGCAGAGGAGAACATGCAACTCAG

AAACCAGGTATCCCAGATATCACCAGCTGAGAAGCAAGTTGTTGATACTGAAAATTTTGTTACCGAAGGACAGTCCTCTGAATCTGTGATGACTGCATTG

CATTCTGGAAGTTCACAGTCGCAGGATAATGATGATGGCTCGGATGTATCCCTGAAATTAGGGCTGCCTTGTGGTGCATGGAAGTAA

>LOC_Os08g33488.1 Oryza sativa subsp. japonica|MIKC_MADS|MIKC_MADS family protein

ATGGGGAGAGGGAAGATAGAGATAAAGAGGATCGACAACGCGACGAGCCGACAGGTGACATTCTCGAAGCGGCGGAGCGGGCTGTTCAAGAAGGCGAGGG

AGCTCTCCATCCTCTGCGATGCCGAGGTCGGCCTCCTCGTCTTCTCCAGCACCAGCCGTCTCTATGACTTTGCCAGCTCCAGCATGAAATCCATAATTGA

GAGATACAATGAGACGAAAGAAGATCCCCATCAAACCATGAACGCAAGTTCTGAGGCAAAGCTTTGGCAACAGGAGGCAGCAAGCTTGAGGCAGCAACTG

CATAACTTGCAAGAATATCATCGACAGTTGTTGGGACAACAGCTTTCTGGTCTGGATGTAGAAGACTTGCAAAATTTGGAAAGTAAGCTGGAGATGAGCT

TAAAAAATATCCGTCTGAGGAAGGACAATGTCATGATGGATCAAATTCAAGAATTAAGCAGGAAGGTTGTTACCACTTAG

>LOC_Os04g23910.1 Oryza sativa subsp. japonica|MIKC_MADS|MIKC_MADS family protein

ATGGGGAGAGGGAAGATTGCCATCAAGAGGATCGACAACACGATGAACCGGCAGGTGACCTTCTCGAAGCGGCGCGGCGGGCTGATGAAGAAGGCCCGGG

AGCTGGCCATCCTCTGCGACGCCGACGTCGGCCTCATTGTCTTCTCCTGCACCGGCCGCCTCTACGACTTCTCCAGCTCAAGCATGAAATCAATAATAGA

GCGGTACCAGGAGGCAGGAGAGGAGCATTGTCGGTTGCTGAACCCAATGTCAGAGGCTAAGTTTTGGCAGCGGGAGGTTACAACTTTGAGGCAGCAAGTG

CAAAACTTACACCACAACAACAGGCAACTTTTGGGAGAGGAAATCTCCAACTTCACAGTTAGAGATCTGCAGCTTCTCCAGAACCAAGTTGAGATGAGCC

TACATTCCATAAGAAATAAAAAGGATCAACTTTTGGCAGAGGAGATTCTAAAACTCAATGAAAAGGGGTCTCTTGTTCAAAAGGAGAACAGTGAACTTCG

CAAGAAGTTCAACATTGCTCATCAACGCAACATAGAATTACACAAGAAGCTTAACTCTGGAGAAAGCACGTCAAGTGAGCAAGTTACCAGAAGCTCAAAG

GATCCCGGAGAATCGAGTACACCCCGTGATTCACGTGTGTGTATTGACCTTGAATTGAGTCAAAAAGAAGTTGAAGATGAATAA

>LOC_Os08g02070.1 Oryza sativa subsp. japonica|MIKC_MADS|MIKC_MADS family protein

ATGGCGCGAGGCAAGGTGCAGCTCCGTCGCATCGAGAACCCGGTTCACCGTCAGGTCACCTTCTGCAAGCGCCGTGCCGGCCTGCTGAAGAAGGCCAGGG

AGCTCTCCATCCTCTGCGAGGCCGACATCGGCATCATCATCTTCTCCGCCCACGGCAAGCTCTACGACCTCGCCACCACCGGAACCATGGAGGAGCTGAT

CGAGAGGTACAAGAGTGCTAGTGGCGAACAGGCCAACGCCTGCGGCGACCAGAGAATGGACCCAAAACAGGAGGCAATGGTGCTCAAACAAGAAATCAAT

CTACTGCAGAAGGGCCTGAGGTACATCTATGGGAACAGGGCAAATGAACACATGACTGTTGAAGAGCTGAATGCCCTAGAGAGGTACTTAGAGATATGGA

TGTACAACATTCGCTCCGCAAAGATGCAGATAATGATCCAAGAGATCCAAGCACTAAAGAGCAAGGAAGGCATGTTGAAAGCTGCTAACGAAATTCTCCA

AGAAAAGATAGTAGAACAGAATGGTCTGATCGACGTAGGCATGATGGTAGCAGATCAACAGAATGGGCATTTTAGTACAGTCCCACTGTTAGAAGAGATC

ACTAACCCACTGACTATACTGAGTGGCTATTCTACTTGTAGGGGCTCGGAGATGGGCTATTCCTTCTAA

>LOC_Os02g36924.1 Oryza sativa subsp. japonica|MIKC_MADS|MIKC_MADS family protein

ATGGGGAGGGGGAAGATTGTGATCCGCCGGATCGACAACTCGACGAGCCGGCAGGTGACGTTCTCGAAGCGGAGGAACGGGATCTTCAAGAAGGCCAAGG

AGCTGGCCATCCTCTGCGACGCCGAGGTCGGCCTCATGATCTTCTCCAGCACCGGCCGCCTCTACGAGTACTCCAGCACCAGCATGAAGTCAGTTATAGA

TCGGTATGGCAAGTCCAAGGATGAGCAGCAAGCCGTCGCAAATCCCAACTCGGAGCTTAAGTTTTGGCAAAGGGAGGCAGCAAGCTTGAGACAACAACTG

CACAACTTGCAAGAAAATCATCGGCAGTTGATGGGCGAAGATCTATCTGGGCTGAATGTTAAGGAATTGCAATCTCTAGAGAATCAGCTGGAAATAAGTC

TACGTAGTGTCCGTACAAAGAAGGACCACGTCTTGATTGATGAAATTCATGAACTGAATCGGAAGGGAAGTCTAGTTCACCAAGAAAACATGGAATTATA

CAAGAAGATCAGTTTAATTCGTCAAGAAAATGCTGAGTTATATAAGAAGATCTACGAGACTGAAGGACCAAGTGAAGTCAATCGGGATTCACCAACTCCT

TACAATTTTGCAGTAATTGAAAAAACAAATGTTCCTGTGCAACTTGGACTCAGCACACTACCACAACATAGTGACGCCGAACAATCAACTGCTCCTAAGC

TAGGGTTACAGTTGAATCCATGA

>LOC_Os02g07430.1 Oryza sativa subsp. japonica|MIKC_MADS|MIKC_MADS family protein

ATGGGGCGCGGCAAGATCGAGATCAAGAGGATCGAGAACGCGACGAACAGGCAGGTGACATTCTCGAAGAGGCGGGGAGGGCTACTGAAGAAGGCGAACG

AGCTCGCCGTGCTCTGCGACGCCCGCGTCGGCGTCGTCATCTTCTCCAGCACCGGCAAGATGTTCGAGTACTGCAGCCCTACCTGCAGTTTGAGGGAACT

CATCGAGCATTACCAGACCGTCACCAACACTCATTTCGAGGAGATCAACCACGATCAGCAAATATTTGTGGAGATGACTCGGATGAGGAACGAGATGGAG

AAGCTGGACGGTGGCATCAGGAGGTTCACCGGCGACGACCTCTCCAACCTCACGCTCGCCGACATCAACGATCTCGAGCAGCAGCTCGAATTCTCCGTCA

CCAAAGTCCGTGCGAGAAAGCATCAGCTCCTGAACCAGCAGCTGGACAACCTTCGTCGCAAGGAGCACATCCTGGAAGACCAGAACAGCTTCCTGTGCCG

CATGATCAACGAGAACCATCATCAGGCGGCGGTGGGCGGCGGCGACGTGAAGGCGATGGTGGAGATGGCGCCGGTGCTGTCGATGCTGACGGCGGCGCCG

GCGTACTACGGCGAGGAGTCGTCGAGCACCGCGCTGCAGCTCACCCCGCCGCTGCACGCCGTCGACGCCGCCGCCGCCGCCGGGTTCCGGCTGCAGCCGA

CGCAGCCCAACCTGCAGGACCCCGGCTGCAGCAGCAGCAGCTTCCATGCCGCCGCCGCCGGCCACGGCCTGCAGCTGTGGTAA

>LOC_Os01g10504.1 Oryza sativa subsp. japonica|MIKC_MADS|MIKC_MADS family protein

ATGATGAACATGATGACCGATCTGAGCTGCGGGCCATCGTCGATGACGGAGCTGACCGCGGCAGCGGCGCCGGCTGGGTCAGGATCGTCGGCGGCGGTGG

CGGCGGGGAGCAGCGAGAAGATGGGGAGGGGGAAGATCGAGATAAAGCGGATCGAGAACACGACGAACCGGCAGGTGACCTTCTGCAAGCGCCGCAATGG

CCTCCTGAAGAAGGCGTACGAGCTGTCCGTCCTCTGCGACGCCGAGGTTGCCCTCATCGTCTTCTCCAGCCGCGGCCGCCTCTACGAGTACGCCAACAAC

AGTGTGAAATCCACCGTTGAGAGGTACAAGAAGGCAAACAGTGACACCTCCAACTCTGGCACAGTTGCAGAAGTCAATGCCCAGCACTACCAGCAGGAGT

CCTCCAAACTGCGCCAACAAATCAGTAGCTTACAGAACGCAAACAGTAGGACCATAGTGGGGGATTCTATCAACACCATGAGCCTCAGGGACCTTAAACA

GGTAGAGAACAGGCTGGAGAAAGGCATAGCTAAGATAAGGGCTAGAAAGAATGAGCTGTTATATGCTGAAGTTGAGTACATGCAGAAAAGGGAAGTTGAG

CTGCAGAATGACAACATGTACCTGAGGAGCAAGGTTGTTGAGAATGAGAGGGGACAGCAGCCACTGAACATGATGGGGGCAGCATCAACAAGTGAATACG

ATCATATGGTTAATAACCCATATGATTCCAGGAACTTTCTTCAAGTGAACATCATGCAGCAGCCTCAGCATTACGCCCATCAGCTGCAGCCAACTACCCT

TCAACTCGGCAGCCGGCCTTCAATTAGTTTTGGTGTAGACACCGTACGTACACACGTACGTTAG

>LOC_Os06g45650.1 Oryza sativa subsp. japonica|MIKC_MADS|MIKC_MADS family protein

ATGGGGCAAGGGAAGATCGAGATGAAGAGGATCGAGGACGCGACGAGGCGGCAGGTGACGTTCAGCAAGCGCAGGGCTGGGTTTCTCAAGAAGGCGAACG

AGCTCGCCGTGCTGTGCGATGCGCAGGTCGGCGTCGTCGTCTTCTCCGACAAGGGCAAGCTCTTCGACTTCTGCAGCCCGCCGGTCATCTTGATGGAGCT

GTTTCACCGTTATGAGATCACCACCAGAAACACTCGGCTTCAGGAGACAAACCGTGATGATGAGCAAATGGTCATGGAGATCACAAGGCTAAGGAATGAG

ATCGACCAGCTCGAAGCCAGTTTAAGGAGGCAAACTGGAGAAGACCTGTCATCTGTGTCCACAGTGGATGAGCTCAGCCAGCTGCAGCTGCAGCTTGAAT

CATCTCTCAGCAAAGTTCATGCGAGGAAGGATGAGCTCATGAGCCAGCAGCTGGAGGATATGCGTCGCATGCATCAGACCGTGCATGAGCAGAACAATTT

CTTGTGCCGCATGGTAACTAAAATACTCCTACTAAATGTGATCACAATAATGATACTTGCTGCCATGATTTCTATCACTCATGCACGCATTGCCCTTGAT

GATTGTATGCAGTTGGGATATATTGTCATCAAGCAAGAAAATTCTTGGCGATTCTGGTTTATATGA

>LOC_Os04g52410.1 Oryza sativa subsp. japonica|MIKC_MADS|MIKC_MADS family protein

ATGGGGCGTGGGAGAGTAGAGCTCAAAAAGATTGAAAATCCGACAAACCGCCAAGTTACCTTCTCCAAGAGGAGGATGGGGCTGCTCAAGAAAGCAAATG

AGCTGGCCATTCTTTGTGATGCACAAATTGGGGTGATTGTGTTCTCAGGCACTGGCAAGATGTACGAGTACTCCAGCCCCCCTTGGAGGATTGCAAATAT

CTTTGATAGATACCTGAAAGCCCCCAGCACCCGTTTTGAGGAGATGGATGTTCAGCAGAGAATCATCCAAGAGATGACAAGAATGAAGGATGAGAACAAC

AGGCTTAGGATCATCATGAGGCAGTATATGGGTGATGACTTGGCTTCACTGACTCTGCAAGATGTGAGTAATCTTGAGCAGCAGATTGAGTTTTCTCTGT

ACAAAGTTCGTCTAAGGAAGCAACAGCTACTTGATCAGCAGCTGCTTGAGATGCACAGCCGGGTATGTAACAAGAGGATATACCGGTTCAGTATTCTTCC

CTACCTTATACACTATATTGAAATAATCAAGTTTTGA

>LOC_Os01g52680.1 Oryza sativa subsp. japonica|MIKC_MADS|MIKC_MADS family protein

ATGGGGAGGGGGCGCAGCGAGATAAAGAGGATAGAGAACCCCACGCAGCGGCAGTCCACCTTCTACAAGCGCAGGGACGGCCTGTTCAAGAAGGCCAGGG

AGCTCGCCGTCCTCTGCGACGCCGACCTCCTCCTCCTCCTCTTCTCCGCCTCCGGCAAGCTCTACCACTTCCTCTCCCCCACCGTCCCCTCCGTGAGGGA

GTTTGTCGAGAGGTACGAGGCCACCACGCACACCAAGGTTTGGGCAGATATCAGGCAGGAGAGGCGCGCCGAGCTGGAGAAGGTGGGCAGCATGTGCGAC

CTCCTGGAGAAACAGCTGAGGTTCATGACGGTGGACGACGGCGAGGAGTACACGGTGCCGTCGCTGGAGGCGCTGGAGCACAATCTGGAGGCCGCCATGC

GCAAGGTGCGCTCCGAGAAGGACCGCAAGATCGGAGGCGAGATCTGCTACCTCCAGAACATTATTAGGGGGCGACAAGAGGAGCGGTACGGGCTGTGCGA

CAAGATTGCTCATGCACAGACTCTGAAGGATGTGGAATGTGGATCCACCTCACTAAGCAATGGCTTGGACCTTAAACTGGGGTTCAACTAG

>LOC_Os12g10520.1 Oryza sativa subsp. japonica|MIKC_MADS|MIKC_MADS family protein

ATGGTCAGAGGAAAGGTGCAAATGCGACGAATAGAGAACCCTGTCCACAGACAGGTAACCTTCTGTAAGCGCCGTGGGGGGCTCCTCAAGAAGGCCAGGG

AACTATCAGTGCTTTGTGATGCTGATGTAGGTGTTATCATATTCTCCTCTCAGGGAAAACTCCATGAATTAGCTACTAATGGAAATATGCATAACTTGGT

TGAAAGGTACCAGAGCAATGTAGCAGGTGGTCAAATGGAACCAGGAGCTCTACAAAGACAGCAGGTAGCAGAACAAGGGATTTTCTTGTTAAGGGAAGAA

ATAGATCTACTACAGAGGGGTCTTAGGTCTACATATGGAGGGGGAGCAGGGGAAATGACACTAGATAAATTGCATGCACTAGAGAAAGGTCTTGAACTAT

GGATTTACCAAATACGCACAACAAAGATGCAGATGATGCAACAAGAGATTCAATTTCTTAGAAACAAGGAAGGCATACTAAAAGAAGCAAATGAAATGCT

TCAAGAGAAGGTGAAAGAACAGCAAAAGCTATACATGTCTTTGTTGGATCTCCATAGTCAACAACCAACACAACCAATGACCTACGGAAATCGCTTCTTC

TCAATCTAG

>LOC_Os03g54170.1 Oryza sativa subsp. japonica|MIKC_MADS|MIKC_MADS family protein

ATGGGGCGAGGCAAGGTGGTGCTTCAGCGGATCGAGAACAAGATCAGCCGGCAGGTGACGTTCGCCAAGCGGCGGAACGGCCTGCTCAAGAAGGCCTACG

AGCTCTCCATCCTCTGCGACGCCGAGGTCGCCCTCGTCCTCTTCTCCCACGCCGGCCGCCTCTACCAGTTCTCCTCCTCATCCAACATGCTTAAGACGCT

TGAGAGATACCAGAGGTACATTTATGCTTCGCAAGATGCTGCCGCACCAACTAGCGATGAGATGCAGAACAACTATCAGGAATATGTGAACTTGAAGGCA

CATGTTGAGATTCTGCAACAATCACAAAGGAACCTTCTAGGTGAGGATTTAGCTCCACTGGCTACAAATGAACTTGAGCAGCTTGAGAGTCAAGTAGTCA

GAACCTTGAAGCAAATCAGATCAAGAAAGACTCAGGTACTACTTGATGAACTCTGCGACCTAAAGAGAAAGGAACAAATGCTACAAGATGCAAACAGGGT

CCTGAAAAGGAAGCTTGACGAGATCGACGTAGAGGCAGCTCCCCCACAGCCTCCATGGAACGGAAACTGCAGCAATGGCCATGGCGGCGGCGGCGGCGTG

TTTTCCAGTGAGCCTCCCCAACCAGAGCACTTCTTCCAGGCCCTCGGGCTTCATGCCGTGGACGTGAACCAGCCGCCGGCGCCGCCACCGGGCGGTTATC

CTCCTGAGTGGATGGCCTAG

>LOC_Os08g41960.1 Oryza sativa subsp. japonica|MIKC_MADS|MIKC_MADS family protein

ATGGAGGGAGGAGGGAGGAGGAGGAAGAGGGGGAAGGTGGAGCTGCGGCGGATAGAGGACCGGACGAGCCGGCAGGTGCGATTCTCGAAGCGGCGGAGCG

GGCTGTTCAAGAAGGCGTACGAGCTGTCCGTGCTCTGCGACGCCCAGGTCGCCCTCCTCGTCTTCTCCCCCGCCGGCCGCCTCTACGAGTTCGCCTCTTC

CACCTCCAGCATTGATACAATTTTTGGTCGGTATTGGGACCTTCTGGACACAACAATTGATCTCAATATTGAAGCAAGGGAATCTCGGGTTGATTGCAAT

ATACAGCTTCGTCAGAAAGAGCGTTCAGATGACCCGGTGCCTAAGATAAACCACATTACTCAATGTGTGTTGGAATCAAATGTCAACGAGCTGAACATCG

CTGAGCTAAGAGGTTTGGAGGAAGCGATGACTAATGCTTTGACAGTTGTTAAGAACAAACTGATGATGAAGGTGGCTAGTGTGCTCCCCCAAAGCGAGAA

GAAGAGGAAGAGTTGCTCGATTTCAGAGCCAAGATCAGGAGTGAGCTCTTAA

>LOC_Os05g34940.1 Oryza sativa subsp. japonica|MIKC_MADS|MIKC_MADS family protein

ATGGGGCGCGGCAAGATCGAGATCAAGAGGATCGAGAACTCGACGAACCGGCAGGTGACGTTCTCGAAGCGCCGCGCCGGGATACTCAAGAAGGCCCGCG

AGATCGGCGTGCTCTGCGACGCCGAGGTCGGCGTCGTCATCTTCTCCAGCGCCGGCAAGCTCTCCGACTACTGCACGCCCAAGACCACGTCCGTGTTTCC

CCCGCTGTCAAGGATCTTGGAGAAGTACCAGACCAACTCCGGGAAGATACTCTGGGATGAGAAGCACAAGAGCCTCAGCGCAGAGATCGATCGTGTCAAG

AAGGAGAACGACAACATGCAGATCGAGCTCAGGCATATGAAAGGGGAGGATCTGAACTCCCTGCAGCCCAAGGAGCTGATCGCGATCGAGGAGGCGCTCA

ACAACGGCCAGGCCAATCTGCGGGACAAGATGATGGACCACTGGAGGATGCATAAAAGGAATGAGAAGATGCTGGAGGACGAGCACAAGATGTTGGCTTT

TAGGGTGCACCAGCAGGAGGTCGAGCTGAGCGGCGGCATAAGGGAGCTGGAGCTCGGCTACCACCACGACGACAGGGACTTCGCGGCCTCGATGCCGTTC

ACCTTCAGGGTGCAGCCCAGCCACCCCAACCTCCAGCAGGAGAAGTAG

>LOC_Os03g08754.1 Oryza sativa subsp. japonica|MIKC_MADS|MIKC_MADS family protein

ATGGCTGGCGGCGGCGGTGGCGGGGGAAGGGGGGAGGGGGAGGGGAGGGCGGCGACGGGGAAGAGGGAGAGGATAGCAATACGGAGGATCGACAACCTGG

CGGCGAGGCAGGTGACCTTCTCGAAGCGGAGGAGGGGGCTGTTCAAGAAGGCCGAGGAGCTCTCCATCCTCTGCGACGCCGAGGTCGGGCTCGTCGTCTT

CTCCGCCACCGGCAAGCTCTTCCAATTCGCCAGCACCAGCATGGAACAGATTATTGACCGGTACAACTCGCATTCCAAGACACTTCAGAGAGCAGAACCT

TCTCAACTCGACTTACAAGGGGAGGACAGCAGTACTTGTGCCAGACTAAAGGAGGAGCTTGCAGAAACTAGCCTTAGGCTGAGGCAGATGAGAGGAGAGG

AGCTGCACAGGCTGAATGTGGAACAGCTGCAGGAGCTAGAGAAGAGCCTCGAGTCCGGTCTAGGCTCTGTTCTCAAGACCAAGAGCAAGAAAATTCTGGA

TGAGATCGATGGACTGGAACGAAAGAGGATGCAATTGATAGAGGAGAATTTAAGGCTGAAGGAGCAACTGCAGGTGTCCAGGATGTCAAGAATGGAGGAG

ATGCAGCCTGGGCCTGATTCAGAAATCGTGTACGAGGAAGGGCAGTCGTCTGAATCTGTCACCAATGCTTCTTACCCGCGACCTCCACCCGACAACGACT

ACAGCTCTGATACATCTCTCAGGCTCGGGTTGTCACTCTTCAGCTCCAAGTGA

>LOC_Os06g06750.1 Oryza sativa subsp. japonica|MIKC_MADS|MIKC_MADS family protein

ATGGGGCGAGGGAAAGTAGAGCTGAAGCGGATCGAGAACAAGATAAGCCGGCAGGTGACGTTCGCGAAGAGGAGGAACGGGCTGCTGAAGAAGGCGTACG

AGCTGTCCGTGCTCTGCGACGCCGAGGTCGCCCTCATCATCTTCTCCACCCGCGGCCGCCTCTTCGAGTTCTCCACCTCCTCCTGTATGTACAAGACACT

GGAGCGATACCGCAGTTGCAACTACAACCTTAACTCATGTGAAGCATCTGCTGCACTGGAAACTGAACTAAGCAATTACCAAGAGTACTTAAAGTTAAAG

ACAAGAGTTGAGTTCCTACAAACAACTCAGAGAAATCTTCTTGGCGAGGACTTGGTTCCACTTAGCTTGAAGGAGCTCGAGCAACTTGAGAACCAGATCG

AGATATCCCTCATGAATATCAGGTCATCAAAGAATCAACAGTTGCTTGATCAAGTATTTGAGCTCAAACGTAAGGAACAACAACTTCAAGATGCTAATAA

AGACTTAAAAAGGAAGATACAAGAAACTAGTGGAGAAAATATGCTTCATATATCTTGCCAAGATGTAGGGCCCAGTGGCCATGCTAGTGAAGCTAACCAA

GAGTTTCTCCATCATGCAATTTGTGACCCTTCCCTGCATATAGGGTATCAAGCTTACATGGATCACCTCAACCAATGA

>LOC_Os03g03100.1 Oryza sativa subsp. japonica|M-type_MADS|M-type_MADS family protein

ATGGTGCGGGGGAAGACGCAGATGAAGCGGATAGAGAACCCCACGAGCCGCCAGGTCACCTTCTCCAAGCGCCGCAACGGCCTGCTCAAGAAGGCCTTCG

AGCTCTCCGTCCTCTGCGACGCCGAGGTCGCGCTCATCGTCTTCTCCCCGCGCGGCAAGCTCTACGAATTCGCCAGCGCCAGGAAAATTCGGCCTGAAAA

GACGGCAAAAACTATATTTCCCCGTGTGGCGATTGAGTTGCCGTCTAAACAGTCACACTACTTCCATAAAGAAATTAGTTGTGAGACTGGCAAGGTTAGA

AATTCTGAAAACATAGGCAACTTAGTACTGGCATTCCGGAGGGCAATATGA

>LOC_Os06g11330.1 Oryza sativa subsp. japonica|MIKC_MADS|MIKC_MADS family protein

ATGGCGAGGGAGAGGAGGGAGATACGGAGGATAGAGAGCGCGGCGGCGCGGCAGGTGACGTTCTCGAAGCGGCGGCGGGGGCTGTTCAAGAAGGCGGAGG

AGCTGGCGGTGCTGTGCGACGCCGACGTCGCGCTCGTCGTCTTCTCCTCCACCGGCAAGCTCTCCCAGTTCGCCAGCTCCAATATGAACGAGATCATTGA

CAAGTATACTACACATTCAAAGAACCTGGGGAAAACAGATAAGCAGCCTTCTATTGATCTGAATATGAGAGGTGAGGAGCTTGAGGGATTGAGTGTGGAA

GAGCTGCAGCAGATGGAAAAGAACCTCGAGGCAGGACTGCAGCGGGTGCTCTGTACAAAGGACCAGCAATTCATGCAAGAAATCAGTGAGCTCCAACGAA

AGGGCATTCAGCTGGCAGAAGAGAATATGCGCCTCAGAGACCAAATGCCTCAGGTGCCTACTGCTGGCTTGGCGGTTCCTGATACTGAAAATGTTCTTAC

TGAAGATGGACAATCATCTGAATCTGTGATGACTGCATTAAATTCGGGAAGCTCGCAGGATAATGATGATGGTTCTGATATATCCCTGAAACTAGGGTGA

>LOC_Os10g39130.1 Oryza sativa subsp. japonica|MIKC_MADS|MIKC_MADS family protein

ATGGTGCGGGGGAGGACGGAGCTGAAGCGGATTGAGAACCCGACGAGCCGGCAGGTGACCTTCTCCAAGCGCCGGAATGGCCTCCTCAAGAAGGCGTTCG

AGCTCTCCGTCCTCTGCGACGCCGAGGTCGCCCTCATCGTCTTCTCCCCCCGCGGCCGCCTCTACGAGTTCGCCAGCGCCCCCAGCCTACAGAAAACCAT

CGACCGCTATAAAGCATACACAAAGGATCATGTCAACAATAAGACAATTCAACAAGATATCCAGCAAGTCAAAGATGATACTTTAGGCTTGGCCAAGAAA

CTTGAAGCTCTTGATGAGTCCAGACGGAAAATATTGGGAGAAAATTTAGAAGGATTCTCTATTGAAGAACTGCGTGGTCTAGAAATGAAACTTGAGAAGA

GCCTCCACAAGATAAGACTAAAGAAGACCGAGCTTCTGGAGCAGCAGATAGCCAAGCTGAAAGAGAAGGAGCGGACTTTGCTTAAAGACAACGAAAATTT

ACGCGGAAAGCATCGCAACCTTGAGGCTGCGGCGCTGGTGGCTAACCACATGACGACGACGACGGCGCCGGCGGCGTGGCCGCGGGACGTGCCTATGACG

AGCAGCACAGCCGGCGCCGCCGACGCCATGGACGTGGAGACTGATCTGTACATTGGATTGCCCGGCACTGAGCGCTCCTCCAACCGGTCGGAGACAGGTT

GA

>LOC_Os02g49840.1 Oryza sativa subsp. japonica|MIKC_MADS|MIKC_MADS family protein

ATGGGGAGGGGGAAGATAGTGATAAGGAGGATAGACAACTCGACGAGCAGGCAGGTGACGTTCTCGAAGCGTCGGAACGGGCTTCTGAAGAAGGCGAAGG

AGCTATCCATCCTCTGCGATGCGGAGGTCGGCCTTGTCGTCTTCTCCAGCACCGGCAGGCTCTATGAGTTCTCCAGCACCAACATGAAAACTGTGATAGA

CCGGTATACCAACGCAAAGGAGGAGCTACTTGGCGGGAATGCAACTTCAGAAATTAAGATTTGGCAGAGGGAGGCAGCAAGCTTGAGGCAGCAACTGCAC

AACTTGCAAGAAAGCCACAAGCAACTGATGGGTGAGGAGCTTTCTGGCCTAGGTGTTAGAGACCTACAAGGTTTAGAGAATAGGCTTGAAATAAGTCTAC

GTAATATCAGAATGAGAAAGGACAATCTTTTGAAAAGTGAAATCGAGGAGTTACATGTGAAGGGAAGCCTAATTCACCAGGAAAACATCGAACTTTCTAG

AAGCCTAAATGTCATGTCGCAACAAAAATTGGAACTGTATAACAAGCTTCAGGCCTGTGAACAGAGAGGTGCCACAGATGCAAATGAAAGTTCCAGCACT

CCATACAGCTTTCGTATCATACAAAATGCTAATATGCCTCCTAGTCTTGAATTGAGCCAATCACAGCAAAGAGAAGGGGAGTGCAGCAAAACAGCTGCTC

CAGAACTGGGACTTCATCTGCCTTAA

>LOC_Os05g11414.1 Oryza sativa subsp. japonica|MIKC_MADS|MIKC_MADS family protein

ATGCACATATACAAAGAGCAGGAGGCTGAACCATCCACTGGCCTGATGATGCCAGAGCCAGCACCTGTTGCTTCCCCCGGCTCCGGTGGCTCAGGTGGCT

CAGGCTCGGTGGGAGCTGAGAAGATTGGGAGCAGGGGAAAGATTGAGATCAAGCGCATCGAGAACACGACGAACCGTCAAGTGACCTTCTGCAAGCGCCG

CAGCGGGCTACTCAAGAAGGCGTATGAGCTCTCCGTGCTCTGCGATGCCGAGGTCGCCCTCGTCGTCTTCTCCAGCCGTGGCCGCCTCTACGAGTACTCC

AACAACAGCGTGAAGGAAACTATTGAGAGGTACAAGAAAGCCAACAGTGACACCTCCAACGCCAGTACAGTTGCAGAGATCAATGCCCAGCACTACCAGC

AGGAAGCTGCTAAGCTGAAGCAACAGATCACCAACCTGCAGAACTCCAACAGGACCCTAGTAGGTGATAATATCACCACCATGAACCACAGAGAACTTAA

GCAGCTGGAAGGCAGACTGGACAAAGGCCTAGGAAAGATTAGAGCACGGAAGAACGAATTGCTGTGTGCTGAAATTGAGTATATGCAGAGAAGGGAAACG

GAGCTGCAGAATGACAACATGTACTTAAAGAGCAAAGTTGCTGAGAGTGAAAGAGGACTGCAAACAGTGAACATGATGGGTTCAGCATCTACGAGCGAGT

ACGTGCAAAATATGATCCATTATGATCCAAGAAACTTCCTGCAATTCAACATCATGCATCAGCCTCAGTATTATCCTGAACAGGAGGACCGAAAGGCCTT

TATGTCAGGTAAAAAATATTCTCAGTGTAACATAGTCAGAGTTCATAGTTCAACAAATGAAATTTGA

>LOC_Os06g23950.1 Oryza sativa subsp. japonica|M-type_MADS|M-type_MADS family protein

ATGGTTCGTGGGAAGACCGTCATCAGCAGGATCGAGAACACGACGAGCCGCCAGGTGACCTTCTCCAAGAGGAGGAGCGGGCTATTCAAGAAGGCCAAGG

AGCTGGCCATCCTCTGTGATGCCCAGGTCGGCGTCCTGGTCTTCTCAAGCACTGGCCGTCTCTATGACTACTCCAACTCCAGTAATTCTCGCAATCTTAC

CTATGATCAGTTATTGGGGGATACCGTCTGTTAA

>LOC_Os02g45770.1 Oryza sativa subsp. japonica|MIKC_MADS|MIKC_MADS family protein

ATGGGGAGGGGAAGAGTTGAGCTGAAGCGCATCGAGAACAAGATCAACAGGCAGGTCACCTTCTCCAAGCGCCGCAACGGCCTCCTCAAGAAGGCCTACG

AGCTGTCCGTTCTCTGCGACGCCGAGGTCGCGCTCATCATCTTCTCCAGCCGCGGCAAGCTCTACGAGTTCGGCAGCGCCGGCATAACAAAGACTTTAGA

AAGGTACCAACATTGTTGCTACAATGCTCAAGATTCCAACAATGCACTTTCTGAAACCCAGAGTTGGTACCATGAAATGTCAAAGTTGAAAGCAAAATTT

GAAGCTTTGCAGCGCACTCAAAGGCACTTGCTTGGGGAGGATCTTGGACCACTCAGCGTCAAAGAATTGCAGCAGCTGGAGAAACAGCTTGAATGTGCAC

TATCACAGGCGAGACAGAGAAAGACGCAACTGATGATGGAACAAGTGGAGGAACTTCGCAGAAAGGAGCGTCAGCTGGGTGAAATTAATAGGCAACTCAA

GCACAAGCTCGAGGTTGAAGGTTCCACCAGCAACTACAGAGCCATGCAGCAAGCCTCCTGGGCTCAGGGCGCCGTGGTGGAGAATGGCGCCGCATACGTG

CAGCCGCCGCCACACTCCGCGGCCATGGACTCTGAACCCACCTTGCAAATTGGGTATCCTCATCAATTTGTGCCTGCTGAAGCAAACACTATTCAGAGGA

GCACTGCCCCTGCAGGTGCAGAGAACAACTTCATGCTGGGATGGGTTCTTTGA

>LOC_Os04g38770.1 Oryza sativa subsp. japonica|M-type_MADS|M-type_MADS family protein

ATGGGGAGGGGCAAGATAGTGATCCGGCGGATCGACAACTCGACGAGCCGGCAGGTGACGTTCTCGAAGCGGCGCAACGGGATCTTCAAGAAGGCCAAGG

AGCTGGCCATCCTGTGCGACGCCGAGGTCGGCCTCGTCATCTTCTCCAGCACCGGCCGCCTCTACGAGTATGCCAGCACCAGCATGAAGTCAGTGATTGA

TCGATATGGGCGAGCTAAGGAGGAGCAGCAGCACGTCGCAAACCCCAACTCGGAGCTGAAGGAGTTTTGTAGCGTCTTCATATATATAACAGAGAACTAA

>LOC_Os08g38590.1 Oryza sativa subsp. japonica|M-type_MADS|M-type_MADS family protein

ATGGGGAGGGTGAAGCTGCCGATCAAGAGGATCGAGAACACGACGAACAGGCAGGTGACGTTCTCGAAGCGGCGGAACGGGCTGATCAAGAAGGCGTACG

AGCTGTCCGTGCTCTGCGACATCGACGTCGCCCTCCTCATGTTCTCCCCCTCCGGCCGCCTCAGCCACTTCTCCGGCCGCCGCGGGGTGGAGGACGTGAT

TCTCCGGTACATGAACCTCTCGGAGCACGACAGGGGAGAAGCCATCCAGAATCGGGAGGAGATTCAGCAAGAGATATATTCTTCCCAGCAACAGCTGCAA

ATCACCGAGGACCGCCTTAGGATGTTCGAGCCAGATCCTGCGGCGTTCGGCACATCCAGCGAGGTCGACGGATGCGAGAAGTATCTCATGGAATTGCTGA

CCCGAGTCGTCGAAAGGAAGAACAACTTGTTGAGCAGTCACATGGCGCCGTTCGATGCAACGACAGCTGCGATGCAGGGTGCAGATGGGACGCAGATGTA

CGTGAGCCAGGCGGACGGGTTGGCCACGTTCGGTGGTGACGCTGCCATGTGGGGCCCAGATGGAGGCGCGGATCCTGGCCATCCGATGTTCAGTGCATCT

GATCCGTTGATCTACCTCAGGGACCACGACGTGTACGACGCCAACTCACAGGTGGCCGGGCTGCACGGCGGCGACCCGTGCGCGGCCGGCGGCGCCGCGG

CGGCGGCGGCCGCGGTGGGGTGCGTCGACGACGACGTCGCCGGAGGCCACGCCGCCGCCGCCGACGCGTGGAGGCAGGCGTACACCTGCACCGAGCTGCT

CTCCACGCTCATCCCCACCACGCCGTTCCCCCTCATGCCGCATTGCCTGGGGCCGGAGGACCAGTACCTGTCGATGGAGCATGGCATGGTGGCGGCGGCG

CAGGAGCCGGTGGAGGCGTCGACGGCGAGCTGCTCCTACGTTCCCAGCGACGAGAACTCCGGCACGCCGGTCATGGCGTACGACAGCAACCCGCCGCCGG

CGAACATCGCCTGA

>LOC_Os06g11970.1 Oryza sativa subsp. japonica|M-type_MADS|M-type_MADS family protein

ATGGGACGGGTGAAGCTGCAGATCAAGAGGATCGAGAACATCCCGAACAGGCAGGTGACGTTCTCCAAGCGGCGAAACGGGCTGATCAAGAAGGCCTACG

AGCTGTCCGTCCTCTGCGACATCGACATCGCCCTCCTCATGTTCTCCCCCTCCGGCCGCCTCAGCCACTTCTCCGGCCGCCGCAGGATCGAGGACGTGCT

CACAAGGTACATCAATCTCCCAGAAAGTGACAGAGGAGGAACTATCCAAAACAGAGAGTATTTGATCAACATGCTCACCCAGCTCAAATGCGAGAGCGAC

GTCACCGAGGATCTCACTAACACGAGCAGTAAGGCGCCGGTTAACTCCAACATTGAGGAGCTTCAGCAAGAGATCAGGAGATGCCAGCACCAGATGCAGC

TCACAGAGGAGCAATTGAGGATGTTTGAGCCGGACCCGGCCAGGTCTGCATCCATGGAGGACGTCGAGGCCAGCGAGAAGTTCATCGCCGGCATCCTCAG

CCGCGTCGAGGAGAGGAAGAGGTATTTGCTGTGCAGCATGGGCTCGTTCGACGTGACGGCGTCAACATCTGCCATGCAGCATCTGTATCTACCGCAGCAG

CATCAGCACGGGGACATCACTGATAATGGGTTTGGAAGCGATGAGGTGGCCTCGTGGGTCTCTGAGGGGATGCCGCCGACAACCTCGTCGGTGGCGTCGA

TATTCGCCGGAACTTCAGATTCCATGATGTCTTTTAGAGATCAAGCGGTGTACGACACGATGAGACAAGATGCATGTGTGGATCAGACAGTGGTCCCGGA

GATGGGGATGTGCCATGTGGACCAGCAGAACCAGAGTGACGACTGGCAGGCGTACACGTCGGCGGAGTTCCTCAACGCGCTAATCCCGCCCACGCCGTTC

CCTCTCGACGACGAGGATACGATGGGCCCGATGTTGGCGTCATCACCGCTATTGATGCCCGGCATCCATGACCAGCAGCCGCCGGTGGAGGATATGGCGA

CGGCGGGCTGCTCGCAAGCGCCGGCTAACGATGGCAACGGGTTATACGCAGCTGAGGACATTGCTCCGGTTAACGTTGGTTAA

>LOC_Os04g31804.1 Oryza sativa subsp. japonica|M-type_MADS|M-type_MADS family protein

ATGGGGAGGCGGGGGCGGGTGGTGCTGCGGCGGATCGAGGACCGGGTGCGGCGCGGGATCTGCTTCCGGAAGAGGCTCGCCGGGCTGGAGAAGAAGGTGG

AGGAGCTCGCCGTGCTCTGCGACGCCCACGTCGGCTTCGTCGTCCTCTCCTGCTCCGGCGACGACGCCAACCCCCACCACTTCGCCGCGCCCGCCACTAT

TGAAAACATTGTGGAACGTTATGAGCACTCTCAAGCAGCACAAAAAGGAGTACATGGCAGGTGCATTCTGCAAAAGAGGAAGAGCAAGGATTTTCAAGTT

CTCAAGGAAACAATAGATAAAGGACCAATCAATGATGACATGCGACCTATTGATGAAAAAGACATATCCACATTAAACATGGACCAGATTAGCCAAATTG

AAATATTATTGGAAGATGAACTGAGGTGGACAAGGGCAAGAAAGGTGGTGGCAGATAGAATTGCCAGGTTGCAGAAAAAGGTACAGAAGAAACCGGCGAC

TGCTGAGACAGAGAGCAACTCCACTGAGATGCCCTCTGATCATGAGAAGAAACAGGTAGCAGGAGGAAGCCAGCAGAGCGCCGAGGAGGAGGAGGAGGAG

ATGGAGGTGGTGCTCAGGCACCGCCTGAGCCTGGGCACCGGCGACCGTGACGATGGTGGCGGTGGCGCGGCGGAGCAACGGCACCGGACGACGCCGCCGC

CGGCGGTCGACCTCAACGTGCCGTGCCGGGACGCGGGTCAGCTGCAGTAG

>LOC_Os01g69850.1 Oryza sativa subsp. japonica|M-type_MADS|M-type_MADS family protein

ATGCCCCCCCCCCCCCCCCCCCACTCCATCGATCCCCTCCTCCTCCTCCCCCACTTCTCCCCACCACCACACCACCACACACACGCACGCACGCACGCAC

GCCTCCGTTTTCCCCCACCGACGAGAGGCGGAGAGGGAGGGGAGCTAGGGTTTTTCGGGGCGGGGATGGCGCGGAGGGGGAGAGTGCAGCTGAGGCGGAT

CGAGGACAAGGCGAGCCGGCAGGTGCGGTTCTCCAAGAGGAGGGCGGGGCTGTTCAAGAAGGCGTTCGAGCTCGCCCTGCTCTGCGACGTGGAGGTGGCG

CTCCTCGTCTTCTCCCCCGTCGGCAAGCTCTACGAGTACTCCTCCTCCAGCATTGAAGGTACCTATGATCGCTATCAGCAATTCGCTGGAGCCAGGAGAG

ACCTGAACGAAGGAAGTACAAGCATCAACAGTGATGAAAATGCAAGTATACACTCCAGGCTTAGGGACATAACGGCCTGGTCTCTCCAAAACAATGCTGA

CGAGTCGGATGCTAATCAGCTAGAGAAACTGGAGAAACTGCTGACAAATGCTTTGAGGGATACGAAATCAAAGAAGATGTTGGCAAAACAAAATGGTGAA

GGGAGTAGGAGCAGAGCAAACTCCAGTGGCTCTAGGGGGCAGGAGGAAGGAAGTGCATGA

>LOC_Os05g11380.1 Oryza sativa subsp. japonica|MIKC_MADS|MIKC_MADS family protein

ATGCACATATACAAAGAGCAGGAGGCTGAACCATCCACTGGCCTGATGATGCCAGAACCAGCACCTGCTGCTTCCCCCGGCTCCGGCAGCTCAGAGGGCT

CAAGCATCGAGGACACAGCGGACCGTCAGGTCACCTTCTGCAAGCGCTGCAACGGGCTACTCAAGAAGGCGTATGAGCTCTCCATGCTCTGCGATGCCGA

GGTTGCCCTCATCGTCTTCTCCAGCCGCGGCCGCCTCTACGAGTACTCCAACAACAGCGTGGAGGAAACTATTGAGAGGTACAAGAAAGCCAACAGTGAC

ACCTCCAATACCAGTACAGTTGCAGAGATCAATGCCCAGCACTACCAGCAGGAGGCTGCTAAGCTGAAGCAACATATCACCTACCTGCAGAACTCCAACA

GGTTCATTATTTCACTCAGCTGCTTAGATTTTTTTTTTTCAACTTGA

>LOC_Os11g43740.1 Oryza sativa subsp. japonica|M-type_MADS|M-type_MADS family protein

ATGGGGAGGGTCAAGCTCAAGATAAAGAAGCTGGAGAACAGCAGCGGCCGACATGTCACGTACTCGAAACGGAGGTCCGGGATCCTCAAGAAAGCCAAGG

AGCTCTCCATTTTGTGCGACATCCCTCTCATCCTGCTGATGTTTTCACCCAACGACAAGCCCACGATTTGCGTTGGCGATCACAGCAGCATAGAGGATGT

CATAACAAAGTATGCACAGCAAACTCCTCAGGAAAGGGCTAAAAGGAAGTTGGAGAGCTTAGAAGCCCTAAAGAAGACATTCAAGAAACTAGATCATGAT

GTCAATATTCAGGACTTTTTAGGCTCAGGGGGTCAGACTGTTGAGATGCTGATTAAGTCAATTGTTTGGCAGGAATTATCGAGCCATCTTGGTGCTTTGC

AATGTCAAATGGCAGATGTCGAAAAGCGCCTCAGTTATTGGAGCGATCCCGAGAAGGTTGAGAATATTGACCATATAAGAGCGATGGAGCAATCTCTCAA

AGAATCTCTGAATCGCATCCGGATTCATAAGGAGAACTTTGCAAAGCAGCATCTGATGAGCCTACAGTGTGCAGCTGCTCAGTTCCAGAACGACATGAAG

CTCCCACTAGGACTAACAGGTGACCCGAACACTTCATCGTGGTTCCATGGCGGCGGCGGCGCAGAGGCGCAGCAGCCAATGATGCTACCTGAAGACCCCA

GCTTGCTCCATCAGAGAGACATCGGGTGCTCAGCGAGCACGTCGTTGCAGAGCTACCCGGGCTACTTCAGCATGGGCAAGCAGTCCACCGACAACGCCGG

CGGCGGCGAGCAGCACCACCACGCGGCGGTGCAGCAGCAGCCGGAGTTCAGCCAGGCCGACTGCCTGACGTCGCTGCAGCTCGGCGCGCAGTTCCCGTAC

CCGTCGGCGTTCGACAACGCCGGGCTGCTCAGCGACCGGCTGTTCGACAACGCGGCGGCGGCGGCGGCGGCGATGGACTTCGGCGGCCACTACGACCTGC

CGAGGCCCGGCGACGAGGCCAGCTTCCAGAACTGGGCGTCGGCGGCCTGCGGCGCCACCATGTATGATCATCAGCAGCAGCAGCAGCAACAGCAGCAGCC

TGCCCAACTCCCTGCAGCAGCAACTGTAGAAGCACCTTCATTCAATCATCCCTCACCACACCGGCAGCTCATGATTTGA

>LOC_Os08g41950.1 Oryza sativa subsp. japonica|MIKC_MADS|MIKC_MADS family protein

ATGGCAGAGAAAAAGAAGAAGAAGAAGAAGAAGAAGCCGCAATCACTCCTAGTCCTTACAAGCTGGAGATCGATCGGGATGGGGAGGGGTCGGGTGGAGC

TGAAGAGGATCGAGAACAAGATCAACCGGCAGGTGACGTTCGCCAAGCGCAGGAATGGCCTGCTCAAGAAGGCGTACGAGCTCTCCGTCCTCTGCGACGC

CGAGGTCGCCCTCATCATCTTCTCCAACCGCGGCAAGCTCTACGAGTTCTGCAGCACCCAGAGCATGACTAAAACGCTTGAGAAGTATCAGAAATGCAGT

TACGCAGGACCCGAAACAGCTGTCCAAAATAGAGAAAGTGAGCAATTGAAAGCTAGCCGCAATGAATACCTCAAACTGAAGGCAAGGGTTGAAAATTTAC

AACGGACTCAAAGGCAATACTACAAATCTAAACATAGGCTGTGTTTAGTTCGGTCCAAAGTTTGGAATTTGGTTAAAATTAGAGACGATGTGACTGAAAA

GTTGTGTATGTATGAAAGAAATTTGCTGGGTGAAGATCTTGATTCATTAGGCATAAAAGAGCTCGAGAGCCTAGAGAAGCAGCTTGATTCATCCCTGAAG

CACGTCAGAACTACAAGGACAAAACATCTGGTTGACCAACTGACGGAGCTTCAGAGAAAGGAACAAATGGTTTCTGAAGCAAATAGATGCCTTAGGAGAA

AACTGGAGGAAAGCAACCATGTTCGCGGGCAGCAAGTGTGGGAGCAGGGCTGCAACTTAATTGGCTATGAACGTCAGCCTGAAGTGCAGCAGCCTCTTCA

CGGCGGCAATGGGTTCTTCCATCCACTTGATGCTGCTGGTGAACCCACCCTTCAGATTGGGTACCCTGCAGAGCATCATGAGGCGATGAACAGTGCGTGC

ATGAACACCTACATGCCCCCATGGCTACCATGA

>LOC_Os05g23780.1 Oryza sativa subsp. japonica|M-type_MADS|M-type_MADS family protein

ATGGTGAAGGGAAAGGGAAGGGCTGGCAGGAAAAAGGTTGAGATCAAGCGCATCGAAAAAAAGGATGCACGTGACGTTTGCTTCTCGAAGCGTCGCCAAA

CCCTATTTAATAAAGCTGGTGAGCTTTCATTGCTATGTAATGCAAATATAGCTGCTGTTGTGATTTCTCCTGCTGGTAGGGGTTTCTCCTTTGCCCATCC

CTCTGTTGATGATGTTGCTGACCGCTTGGCCTCCATGGCCATGGGCATTCCCAATAACCATTCCTTGGGTGGTGGGTACCATGATAGCGGTGAGGTGACA

AACATAGCGCAACAGCAGAAAATTGAATATGTGGAGCTCCAAAAGTCATTGGAAAAGTCGGAAAAGAAGAAGAGGGTTCAGGAGGCAATGGAGAAGGAGA

GGGCAGGACATCTTATGCAATCACTGACTTCTGAAGTTAATCTCCTGGGGCAGGATGAATTGGAGGAGCTGCACAACAAACTCTCAGCTCTACCTTACAC

TTCCATTGCAAAGTTCTACCAGGTACTGCAAGATGCAAAGGGTACTAGAATGCCATTGCCACAGCCACATATTGAAATAGCATGTCAATCACAATTTCTA

TTTGAAGAGCAGGCTGTCACACCCGCAAATGCTGATTTTCCGGGATCTAGCACATGA

>LOC_Os06g22760.1 Oryza sativa subsp. japonica|M-type_MADS|M-type_MADS family protein

ATGACAAAGCGGAAGATTGAGATCAAGCGCATCAAGAATGAGGAAGCACGCCAAGTGTGTTTCTCTAAGCGTCGTCCTAGTGTGTTCAAGAAGGCCAGCG

AGCTCTATACCGTCTGTGGTGCAGAGGTTGCTATGCTTGTCAAATCTCCTGCTGGTAAATTTTTCTCCTTTGGTGCCCCCTCTGTCGGATTTGTTCTTAG

CCGCTTCCATGCTACTACTACTTCTAGAAAACACTCCAGTATGGGTGTTACGATCCAACATGATAATAGTGCAACAATCAAACTACATGAGTTGAATCAA

CAACACATAGAGTTGCAAAACCAACTGCAAGCTCAAAATGAGAAAATGAAAGCGCTACAAGAGGTTGCTAAGAAGGAAAGTGGGGGAAAAGTGATGGGTT

GGTTGAATAGCAAGGTTGAAGATATATGCCAAGAAGATTTGGAAGAGTTCAAGATGGTGCTTGAGTCTCTAAAGTACTTGACGAGGGGGATAATTAATCA

ACTCTTTCAGAATTATGCTATGTTCTCTAATATGATGCGTGTGCAGCATTGTGTGACCGCTTTGCCCAATCAACAATTCCTTCCGAGCAGTGAAGATGTC

AAACCGATGATTCACCATGTTCCTAGTTCAAGTTATGGATGGAACACAAGCATAGATAGCAAACCTAATTCAAGTGATGCTCATGTTGTTGGGGCCAGGA

GGTACTTTCCGAAGTGA

>LOC_Os03g14850.1 Oryza sativa subsp. japonica|M-type_MADS|M-type_MADS family protein

ATGGTGAAGTGTAGGGCACGCACGACTAGGAAAAAGATTGAGATCAAGCGCGGGGACAAGAAGGTACGAGATGCATGCTTTTCCAAGCGCCACACTACCA

TCTTCAACAAGGCCAATGAGCTCGCTATCCTCTGTGGCGTGATGGTTGCTGTTGTTTTTGTCTCTCCCAATGCCAATGGCGGGATTTTCTCATTTGGCTA

CCCATCTGTCAGCTCTGTGGCAAATCGCTTCCTCGCCAATGCTCCAAACAACACCAGTGTGAGTAGCTCAACCCAAAGCGGGAGGGATGTGGAAATCCGT

GAACTAGAACGTGAAGAAAGAGAGTTGAAAGAGCATCTACAAGCTTCCACAGATCAGAATAAGCTTCTGCGGGAGGCAATAGCAGCAAGGGATGGAGGGC

AATTGATGCTTCTCCTGCAGAGTGATTGTAGTGAGTTGGGACCAAAAGGTCTGGTGGCAGTTGTGCGGTTGTGGTTCCGCAATTCTCTTGTTGGCATTGG

GCTTGATCGGTTGTGCTGGCTGCTGCAGCAAATACTCTTTGGTTTGGATTGGGGCTGA

>LOC_Os12g21850.1 Oryza sativa subsp. japonica|M-type_MADS|M-type_MADS family protein

ATGGTGCATCCGCTGGGGAGGACGAGTATGGGGCGGCAGCGCATCGAGATCCGCCGCATCGACAACAAGGAAAGGCGGCAGGTCACCTTCACCAAGCGGA

GGGGCGGGCTGTTCAAGAAGGCGTCGGAGCTCGCCCTGCTCACCGGCGCGAGCGTCGCCGTCGTTGTCTTCTCCCCTGCCAAGCACGTCTACGCCTTCGG

CCACCCGTCCGTCGACGCCGTTCTCCGCTCATACGCCTCCGTCCCCGGGGAGGCCGCCGCGGTCGCACCCGTCCCCGTCCACGGCGGCAGCGGCGGCGAG

GACGTCGACCTCCTCGGGCTGAGGCTGGCGGCAGATGATACCGGGGCGCAGGTCGCGGCGGAGCATGCGCGGATGCGCGACGTCGCGGCGAGGATCGTGC

AGGCGAAGGCCGGGAGGCGGTTCTGGTGGGAGGCCGACGTGGACGCGCTCGGGGAGGCCGAGTTGCTGGAGTTCTTCACGGCGCTCAAGAAGCTCAGGGA

CAACGTCGGCCGCCACGCCAACGCCCTGCTCGCCCCCCAGCCGCCGCCGCTTCCGCTGCAGCAGAAGCAGCGGCGCCGGCGGTAG

>LOC_Os12g21880.1 Oryza sativa subsp. japonica|M-type_MADS|M-type_MADS family protein

ATGCGTCCGCTCGGGAGGACTAGTAAGGGGAGGCAGCACATCGACAACAAGGAAAGGCGGCAGGTCACCTTCACCAAGCGGCGGGGCGGGCTGTTCAAGA

AGGCGTCGGAGCTCGCCCTGCTCGCCGGCGCGAGCATCGCCGTCGTCGTCTTCTCCGAGACCAACCTTGCCTACGCCTTCGGTGACCCATCCGTCGACGC

CGTCCTCCTCTCCTACGGCCCCGTCCCCGGGGAGGATGCTGAGCCCGCACCCGTCCACAGCGGCGGGTTAGGCAAGGACGTCGACCTCGAGATGCTGAGG

CATAACAATTGTGCTTCTTCTCTGCAATTTGTTCTCGTCTCGCTTTCTTTCATCATGTAA

>LOC_Os06g30810.1 Oryza sativa subsp. japonica|M-type_MADS|M-type_MADS family protein

ATGGTGAGGTCGCGTGGGAGGCCGAGCCTGGGGAGGCAGAGGATCGAGATCCGCCGCATCGACAACAGCGGGCGGCGGCAGGTGACCTTCTCCAAGCGCC

GCAACGGGCTCTTCAAGAAGGCGTCCGAGCTGTCCACGCTCTGCGGCGCCTCCGTCGCCGTCGTCGCCTTCTCCTCCGCCGGCAACGTCTTCGCCTTCGG

TCAACCCACCGTCGACGCCGTCGTCCGCCGCTTCGACCCGCTCCACGCCGACGGCGCCGACCCCGCCCCCGCGGCCGTCGAAGACGGCGGCGGCGGCGGC

GACGACGTCGTCGTCGCAGACCCCGAGGAGCTGGATGCGTTGAGGCGCGCGGAGGAGCAGACCAAGGCGCAGGTGGCGGCGGAGCAGGCGCGGATGCGCG

ACGTCGGGGACAAGGTCACGCAGGCCATGGCGGGGAGGGCCCTCTGGTGGGAGGCGGACGTCGAGGCGCTCGGGGAGGCGGAGCTGCCGGAGTTCGTCAG

GGCGCTCGAGCGGCTCAGGGACAGCGTCCACCGCCACGCCAGCACGCTGGCCTCCACAGCCACGCCGCTGCCGCCGCCGCCGGAGCAGGAAGAAGAAGTT

CCTGAGCTAGACGTCTCGGATTATTCCTTTTAG

>LOC_Os06g30830.1 Oryza sativa subsp. japonica|M-type_MADS|M-type_MADS family protein

ATGGGTCGGCCGCGAGGGGGGACTAGCAAGGGGAAGCAGAAGATCGAGATGTGCTGCATCGACGGCAAGGAGAAACGGCAGGTCACCTTCTCCAAGCGCC

GCCGGGGCCTCTTCAAGAAGGCGTCCGAGATATCCACGCTCTCCGGCGCGTCCATCGCCATCGTCTCCTTCTCCAAGGCCGGCAACGTCTTCGCCTTCGG

CTCGCCGAGCGTCGACGCCGTCCTCCGCCGCCACGTCGTGGCCGGGCCTTCCACCTCCACCTCCCACGCCCACGCCGGCGGCGACGTCTTCGCGGACGAC

GGCGGCGACAACCCCGAGGTGCTGAACGCGCTGAAGCGGGCGACGGACGAGGCCGCGGCGGAGGTGGCGGCGGAGGACGCGCGGCAGAGCGGCGTCGAGG

GAAAGATCACGGAGGCCATGGCGGCGGGGAGGCGGCGGTTCTGGTGGGACGCCGCCAACGTCGAGGCGCTCGGGGAGGCGGAGCTGCCGGTGTTCGAAAG

GGCGCTCCATAAGCTCAGGGGCGCCGTCGCACAGGGAGGCAACAATCCAATCCAGGAGAGATGGGATAGGATCCCAGCTCAAGGGAGCCCTGGTGGCAGC

TACGGTTGGGAGGACCTAAGCCCTACAGGCAAGCTCTATAATCAACCCCCTTTTGCTTACCGGCTTCCAGTGATGCAGTCAATAGTTTCTGATCAGCTTG

GAGTGGTCTGTGCCATGTTGCTGTGA

>LOC_Os09g02780.1 Oryza sativa subsp. japonica|M-type_MADS|M-type_MADS family protein

ATGGAGAGCGAGGAGGCACGGAAGGTGTGCTTCTCCAAGCGCCGTGCGGACCTCTTCAAGATGGCGAGCGAGCTGTCCGTTCATTTCAACGCCGACGTGG

CGGCTGTCGTCTTCTCCCCCGCCGGCAACAGGGCCTACTCCATCGGCGACCCGTCGGTCATGGACCGCTTCCTGTCGTCGTTGCCGGCGCCGGCGCCGCC

GGCGGAGACCGAGCCAGAGCCAGAGGTGGATTGGTCGGTGATGGAGGAGCTGAGCAGGCTGTGTGGCCAGCTGCAGGCCATGGTGGACGCGCACAAGGCG

CGCCTGGAGAAGGCGGAGGAGAAGCTTCGCGAGAGCGGCGCGGCGGCGTGGATGATGGATCTGGAGGCGGAGGTGGGTCGCATGGCGCCGGAGGATGTGC

TGGCCCTGGTGACTAAGCTGGCGGTGCTGCGGGACGGCGTGGCGGAGCGCGCCCACGAGATGCTGCGCGAGGCGCTGCTCGCTGTCGCTGCCCCGACCCC

GACCACCCCCACCACCCCGCCGCCCGCGGGCTTCTGA

>LOC_Os09g02830.1 Oryza sativa subsp. japonica|M-type_MADS|M-type_MADS family protein

ATGGTGACGGCGGCAGCGGCGAGGAGGCGGCCGAGCCTGGGGCGGCAGAAGATCGAGATCCGGCGCATCGAGAGCGAGGAGGCACGGCAGGTGTGCTTCT

CCAAGCGCCGTGCGGGCTTCTTCAAGAAGGCCAGCGAGCTGTCCATCCTGTGCAGCGCCGACGTGGCGGCCGTCGTCTTCTCCCCCGCCGGCAAGGCCTA

CTCCTTCGGCCACCCGTCGGTGGAGTTCCTCCTGGACCGCTTCCTCTCGTCGTCGTTGCCCGCGACGGCCGGGAAGGAGGAGGGGTCGTCGGTCTCGGTG

GTGGCGGAGCTCAACAGGCAGTACGGCGAGCTGCGCGCGATGGTGGACGCCCACAAGGCGCGCCGGGAGAGGGCGGAGAAGACGATGGAGAAGCAGCGGC

AGCGGCAGCCCGCGGCGTGGATGGATCCGGAGGCGGAGGTGGGTCGCATGGCGCCGGAGGAGCTGATGGCCTTAGGGACGAAGCTGGTGGCCGTGCAAGG

TGGCGTGGCGGCGCGCGCGGACCAGATGCTGCGTGACGCGCTGCTTCTTGGCCGGAGGCCGAACACCACCACCACCACCACCACCAGGGCGCCGCCCGGC

TTCTTCCACCTGCACCCGCACTTCTGA

>LOC_Os01g74440.1 Oryza sativa subsp. japonica|M-type_MADS|M-type_MADS family protein

ATGGCGATGCAGGTGGCGGCGCCGTCGCGGAGGAGGCCGAGCTTGGGGCGGCAGAAGATCGAGATCCGGCGCATCGAGAGCGAGGAGGCACGTCAGGTGT

GCTTCTCCAAGCGCCGTGCCGGCTTCTTCAAGAAGGCCAGCGAGCTGTCCATTCTGTGCAGCGCCGACGTGGCCGCCGTCGTCTTCTCCCCCGCCGGCAA

GGCCTACTCCTTCGGCCACCCCTCCGTGGAGTGCCTCCTGGAGCGATTCCTGCCGGACTCGTCGTCGGGAGCGGCGGCGCGGGTGCGGCGAGGAGCGAAT

AATAATGGTGGTGGTGGGATGGTCGGGGAGCTCAACAGGCAGTACGGCGAGCTGCGCGCGATGGTGGAGGCGCACAAGGCGCGGCGGGAGAGGGCGGACG

AGAAGATAGAGATGGAGCGCGCGGCGGGGAGGTGGCTGCCCATGGACGCCGATGTGCGCCGCATGTCGCCGGAGGAGCTCATGGCTTTCGGGACGGGCCT

CATGGCTGTGCAGGCTGCCGTCTCCGCGCGCGCCGACCAGATGCTGCGCGACGCGCTCCTCATTGGCCGCAGGCCGCCCACCACCACCACCGCCGGCTTC

GGCTTCTTCCACATGCCACACTACTGA

>LOC_Os09g32948.1 Oryza sativa subsp. japonica|MIKC_MADS|MIKC_MADS family protein

ATGGGGAGAGGGAGGGTGGAGCTGAAGAGGATCGAGAACAAGATCAACAGGCAGGTGACGTTCGCGAAGCGGAGGAATGGGCTGCTCAAGAAGGCGTACG

AGCTCTCCGTGCTCTGCGACGCCGAGGTCGCCCTCATCATCTTCTCCAACCGCGGCAAGCTCTACGAGTTCTGCAGCGGCCAAAGCATGACCAGAACTTT

GGAAAGATACCAAAAATTCAGTTATGGTGGGCCAGATACTGCAATACAGAACAAGGAAAATGAGTTAGTGCAAAGCAGCCGCAATGAGTACCTCAAACTG

AAGGCACGGGTGGAAAATTTACAGAGGACCCAAAGGAATCTTCTTGGTGAAGATCTTGGGACACTTGGCATAAAAGAGCTAGAGCAGCTTGAGAAACAAC

TTGATTCATCCTTGAGGCACATTAGATCCACAAGGACACAGCATATGCTTGATCAGCTCACTGATCTCCAGAGGAGGGAACAAATGTTGTGTGAAGCAAA

TAAGTGCCTCAGAAGAAAACTGGAGGAGAGCAACCAGTTGCATGGACAAGTGTGGGAGCACGGCGCCACCCTACTCGGCTACGAGCGGCAGTCGCCTCAT

GCCGTCCAGCAGGTGCCACCGCACGGTGGCAACGGATTCTTCCATTCCCTGGAAGCTGCCGCCGAGCCCACCTTGCAGATCGGGTTTACTCCAGAGCAGA

TGAACAACTCATGCGTGACTGCCTTCATGCCGACATGGCTACCCTGA

>LOC_Os02g06860.1 Oryza sativa subsp. japonica|M-type_MADS|M-type_MADS family protein

ATGGCGAAGGCGGCGGCGGAGATGGACGTCGGCGGCGGCGAGGGGAGGAGGTGGAAGCGGACGCGGGGGCGGCAGAGGATCGAGATGAAGCTGATCGAGA

ACAAGGAGGCGCGGCAGGTGTGCTTCTCCAAGCGCCGCGAGGGAGTCTTCAAGAAGGCCAGCGAGCTGTCCGTGCTCTGCGGCGCGCGCGTCGCCGTCGT

CTTCTTCTCCCCCGCCGGGAGGCCGCACTGCTTCGGCCACCCCTCCGTCTCCGCCGTCGCCGACCGCTTCCTCCTCGGCCGCTCCCCCGCCGACGCCGCG

GCGGCGGCGGCGGAGGAGGAGGAGGCGGTCGTGCGCGAGTTCAACCGCGTGGAGGAGCGCCTGAAGGACGCGCTCGGCGCGGCGGCGCGCAGGCGGGACG

CGCTCGACGAGGCGGCGCGCGTGGCGGGCGTCTGGAACGACGCCGACGTGCGCCGCGCGGGGCTCGCCGACCTCGTGTCCATGCTCGCCGCGCTCCAGAG

GGTCCAGGCCGAGGCGTCGGAGCGCGTCCACGACATCATCGTCGAGGAGGCGATGACACACTACACGGGCGCCGCCGCCGCCGCCGCCAACCTCATCGAC

TACCTCGACGCCGGCCCGTTAGTCTCTCACAGCCCCGGGAGCCACGACACCACGACGAAGTTGATCGGCGGCAACGCTGTCCATGCGCCGCCGTTGTCCT

TCCCGCCGATGATAATGCCGCCGCCTCTCCCCCCGCAGTTCAGCCATGGATTTGGGTACACTGACCTCGCCGCCGGCTACGGCTACAACCTTGACCACGG

CCATGGCGCCGCCTATGAAACGGAAGAATTTCACAACGCGGCAGCATGCGACTTCTTTTAA

>LOC_Os04g25870.1 Oryza sativa subsp. japonica|M-type_MADS|M-type_MADS family protein

ATGGCTCTTAGGAAGATGAAGCTTCAGCGGATTGTCATTGATGTGAAGCGGCAGGTGACATTCAAGAAGCGTCTCAATGGCTTGACGAAGAAGGTTAGTG

AGTTCGCAACACTATTCCTCATGGTGTACGGTGAGGTTGAAGTGCAAGCAACGAAGGTTTGGCCGTTGGTGTGGAAGGCGACCAGAGTTCTCGAACACTT

CAAGGCCATGCCGCAGCTAGACAGATACAAGAAGATGACTGACCTAGAGGACATCCTCAACGAGCAGGTCGACAAGCTCAAAGAGGAGTTGCACAAGGTG

GGACGCGATGCTGATGAAAGCGACACCAAGCTAATCCTCGTTGAAGCCCTCAATGGCCACCGCCCGAGCCTCGAGGGGCTCACGATCGAGCAGATTACCA

GCCTCGGATTGATGGCAAATGCACACCTCAAGATTGTCAACGACCGCCTCAAGAAGCTCCGTGAGCAAGGCCTCATACCAGCATCCTTGTTATTGTCTGG

CACGGAGGTTCCAATCCAGAGGGAGGGATGGCTGATGGATGTTGCAAGGGGCATTGGCTCAATGGGGAACAATCGGTTCGGAGGTACCAGCGGAAGCGGC

ATTGCTGGATCCAATGGTGACATGGCTTAG

>LOC_Os03g37670.1 Oryza sativa subsp. japonica|M-type_MADS|M-type_MADS family protein

ATGTTCAAGAAGCGGCAAAAGAGCCTAATGAAGAAGGCAAGCGAGCTCTCGACGCTATATGGCGTGGATGCATGCGTTGTGATGTATGCTGAAGGCGAGG

CGCAACCTATGATGGTGTGGCCATCGGTACCAGAGGCAAGGCGTGTGATCGAGCGCTTCAGGGCCTTGCCGCAGAAGGACCAATACGAGAACACGACTAA

CCTGGAGGGCTTCCTCAAGCAGCGCATCGCCAATCTCCAAGAGAAGGTGGATAAGGCAAAGCATGAGAACGATGAGCTCGAGACAAAGCTCCTCCTCCTC

AACAGCCTTGACTGCTGCCTCCCTAGCCTTGTGGGCCTCACGGTCAAGCAGATTACTAGCCTCAACTCGATGGTAGAGGAACGCCTCAAGAAGCTCCGTG

GGAATGGTCTCCTGGCGACACCAGTCCCAACCAGCTCGAAGAAGAAAATTATATGGGATGTCCGGTACAACCTCAAAGCGAGGTATGTCACGGATGATTC

TGCCAAGAATGGTAGCCCTAGGTTGCGGTGTACCCCGGCAACCTCCTCCCAACGCCAGAGCTCGCCGGAATTCCGACATCGCCGTTGCCTCCCCTAG

>LOC_Os03g38610.1 Oryza sativa subsp. japonica|M-type_MADS|M-type_MADS family protein

ATGGCTCGCAACAAGGTGAAGCTGCAGCGGATCATCAATGATGCGAAGCGGCGGGCAACATTCAAGAAGCGGCTCAAGGGCTTGATGAAGAAGGCAAGCG

AGCTGGCAACACTATGCAATGTGGACACATGCCTCATGGTGTATGGTGAGGGTGAGGCGCAAGCAACGGTGGTTTGGCCGTCGGAGTCGGAGGTGATGAG

AGTTCTCGAGCGCTTCAAGACCTTGCCACAGCTGGACAAATATAAGAAGATGACCGACCTGGAGGGCTTCATCCAGGAGCGCATCAACAAGTTCCAAGAG

CAGTTAGACAAGGTGAGGCGTGATGCCGATGAAAGTGAGACCAAGCTACTCCTCATTGAAGCTCTCGAAGGCCGCCGCCCAGGCCTTGAGGGGATCACCA

TTGAGCAACTTACCAGCCTTGGCTGGCTTGTAGATGCACGCCTCAACATCGTCAATGATCAACTCCAGAAGCTCCACGAGCAGGGCCTCCTACCAGCGTC

TATTTCACTGCCAACCATGGGGGTACTGCCTTACACCACGGCGGGCTACACTGTTGCGCAAGAGGCACCAATCCAGAGGGGGGGATGGCTCATGGGTGTT

GTAAGGGGCATTGGCTCATTGGGCTATAGTTTGTTCAGAGGTAGCGGCAGAAGCAACACTGCTGGACCCAGTGGTGACATGGTGCAACCTTTCAACATTG

GCGCAGGATCCTCATTGGCCAACCAGGGTATTTCATTCCCTCCCAAGTAA

>LOC_Os01g18420.1 Oryza sativa subsp. japonica|M-type_MADS|M-type_MADS family protein

ATGGCTCGCAATAGGATCATCCTCAAGAAGGTCGCTAAGGATTCCACTCGGCGCCTCACATTCAAGAAGCGGCGCAGGGGCTTGATCAAGAAGGCCGGAG

AGCTGGCCTCGTTGTGTGGTATTGGCGTGTGTGTGGTGGTGTATGGAGAGGGCGAGGTGAAGCCAGAGGTTTGGCCTTCTGCCCCCGAGGCACGAGCAAT

CCTCTCCCGCTTCAACTCGGCGCCGAACATTGATAGGTTCAAGAGGGTGACGAACCAGGAGCAGTACCTCCGAAAGCGCATCGCCAAGGCTCGGGAGCGC

ACGAGCAAGGCGGACGATGTCAACCGTGAGCGTGATGCTACCATCATGCTTTATGAGGCCGCCACCGATAAGCGCCCAGTGGCAGACCTCAATGTCCAGG

AGCTCACCAACCTCGGATTGGTGATCAATGAGCGCATCAACCACCTGAAGGAACGCATTGAGCGCCTTGGAGGTGCGGCCCTCATGGCGCCACCGCCATC

GACGCAACCGACGGAGGCATCATCATCCTTGCCACCGCTAGTGCCATACGCCAATGGTGCTGGCATGGAAGGGAACAAGAGGATGAAGGTGAGCACGCAT

CAGAAAGGCTGGTTCATTAACATGAGCACCATGACAGGTGACGCAGGGACCTCTGCCGACGTTGAAGGCAATACTGGTGTTGGCACCAGCGCTAGGGGTG

ACATGATGCATCTTTCCAACTAG

>LOC_Os01g18440.1 Oryza sativa subsp. japonica|M-type_MADS|M-type_MADS family protein

ATGGCTCGCAAGAAGATCGTCCTGGACCGCATCGCCAACGACGCGACGCGGCGGGCGACGTTCAAGAAGCGGCGGCGCGGGCTGCTGAAGAAGGCGAGCG

AGCTGGCGACGCTGTGCGACGTGGACGCGTGCCTGGTGGTGTACGGGGAGGGGGACGCGGAGCCAGAGGTGTGGCCGTCGACGGAGGTGGCCATGAACGT

GCTGCGGCAGTTCAGGGCGCTGCCGGAGATGGAGCAGTGCAAGAAGATGATGAACCAGGAGGACTTCCTCCGCCTCCGGATCGGCAAGCTCAAGGAGCAG

CTGCGCAAGATGGACCGCGACAACCACGAGCGGGAGACGCTCATCCTCCTCCACGACGCCCTCCAGGGACGCCTCGGCACCTACGAGTCCCTCTCCGTCG

AGCAGCTCACCAGCGTCGACTGCCTCGCCAGCGCCAGGCTCAAGGTCATCACCGACCGCCTCGTCGAGATCCGCGCCCCCAACGAGGACGGCCAGGTGCT

CGTTCCCCCACCTCCTCCTCCCCCGCCGGCGCTCCCCGCTCCTCCTCCGCCTCCGGCGCCAATGCTTCCTCTCGCGCCGCCGCCGACTCATGTTACTCCG

GCCATGCCGCTGTCTTCTATGCCGCCGCCGGCGTTCCACGGGATGAACCACCACCACCACCAGAACCACTTCATCAACCATGGCGGCAACGACCAGAACG

CCTGGCTCATGAACGTCGCCAGGAACGGCGGCGACCTCGGCGCCCTCGTCTACAGCGCCTTCGCCAGCAGCTCCTCCTCCAACACCGGTGGCGCCGGCAC

CAGCGCCGCCGGCGCCGCCGCCCCGGGCCCTGACATGATGGATCTCGCCAACCCGGACATGCCGGGATTCGGGTGTCCCTGGGACGACGATTCTGCGGGG

CCATCTTTCCCTCCCATGTAA

>LOC_Os07g04170.1 Oryza sativa subsp. japonica|M-type_MADS|M-type_MADS family protein

ATGGAGAGGGAATTCTGTGGAGAGATGGAGAGCCAAATTTGCCAATTTGGTACCTTTCTAGAAGTCAACAATCAGCATTCAGCAACTCCAAAAGGAAAAA

CCATTCCAGTTAAATGCACAGACTGCCTCCCAGGAGTGGCTTCCCGCTCGCTCACCGTAATCCCACTTGAAGTTCCACACGACGTTCTTCTTTCTGCAAG

GGAAGCCAAAACTCAATACAATAGCACATTTGAACACAATCTCAGTGAGACCAACTTCGTAACTGGTACTTACAATGGAGCTCACCATGCCAATGTGAAG

GATGTACCACCTGACATTTATGAGACAGAATTTAGGCATCAATTTCCAAGGATTGAAACTTCTTTTTCAATCATTGGAGCACATTTCCTGTCAAGTATGG

TGAGGAGGGGTAGAAGGAAAGGCGTGAGGTACATCGAAGAGGATAGAGATCGCAGCCTCACCCTCAGCAAGAGGCGTGATGGGCTGTTCAAGCTTGCAAA

TGACCTCTCCCTTCTCACTGATGCAAGCGTCGCAATTTGCCTACATGACAGCAATAAGGCACAATTCTTTGGGGCACCATCGGTGAAACCTGTTGTCGAT

GCTTTTGTATCTGAGGCAGAGCCATTTGCCGATGAACAACTGAAGGCCAAGCTTACGTCAATGCAAAGTGAGCTGGTCCAGCTAGAGAATGAAGAAGAAG

AGAAGGACAAGAAGACTGAAGAATCAATCCAGCGCTTCAAGGAGGCCCAAGAAGAGAGCTTAGGTATGGGTATGGCCAAGCATCTATTCTCCAGGCTAGA

GGATCTCAGCCACGATGATATGCGTGAACTTTTAGATGTACTCTTGCCGCTCCAGCAAGATTTCAAGAAGCGGCTGCCTCCCCTACGTCGTGGCAGCAAG

CTGCAAATTGGTGGATCAAGTGCGTGGGCACATCAGCAGCCCTCGTGTTCTCGCTTCTTGGCCTCACACCGTCCATTCACTCCATTACTTCCTGGAGGGA

CATCAGGAGTTCCGATGATACCTCCGCCTCCAGTGCCGGGCTCACCATGGTCTCAGATATTTCCGCTACGGCCACCACTGTTTCCTTCTCCAGAGTTAGT

GCCATCCCAACAATTACCACCAGTATCACCGCCGCAGAACACAGTGGCACCTCCTCCAATGCATGCTCCATTGGTGCAACAGCCACTTACTAATCAATCA

TCGGCGGTTCCCCTGCTGACGCAATGGCAAATGCGTTTTGGGGATCAGCCACCCGCTGAAGTGCAAGCATGCACTCCCGTAGAGCAACCTCAGAATGACA

ATGCAGTCCATACCCCAACCTTCAGCGACAGTTTCCTTTTGGAGTTGTTGGCTGATGTCAGCGATGATGGCATTGCTACTGCTGAACCATTGTGTTCTCC

TCCTATTGATGACCAGTTTTTGGCTGACATCGACTGGCTTGCTGAATTGGACACTATCGATGGAAATTTATAG

>LOC_Os01g11510.1 Oryza sativa subsp. japonica|M-type_MADS|M-type_MADS family protein

ATGCCGAGGAGGGCGAGGAGGACCGGCGCGGCGTACGTCGACGACGAGAGGGAGCGCGACATCACCTTCTTCAAGCGGCGGAACGGGCTGTTCAAGTGCG

CCAGCGACCTCTCCATCCTCACCGGCGCTAGCGTCGCCGTGGTCATCGAGGACCAGAACCGCAGCAAGTTCCACGCGGTCGGGACGCCGACGGTGCAGGC

CGTCGTCGACGCCGCCCTCTCAAGCGACGTGGAGGAGGCGGCGGCTGAGGCGCGGCCGGTCGCCGACGAGCAGCTGATGGAGAGGATCGCGCCGCTGGAG

AGGGAGCTGGCTTGGCTGAAGGGTGAAGCTGCCGAGAAGGATGAGACGACGAAAGCATCCAAAGCTCGGTTCAAGATGGCCCAGAAGAAGGAGGAGAACG

AAGAGGAGGGTGACACGAAGAAGAAGAAGCTCTTCTTCTCCAAGGCCGACAAGCTCAGCTCAGACGAGATGAATGAACTGTTAGCTGAGATGCTTGAGAT

CAAGAAGGAACTCAACGTGCGGCTACCCCCGCTACGTCGTCGCGGTGGCAAGCGGCCAATCCAGGGCTCTAGCGTGCCACCTCCACCGCCGCCGCCGCCA

CCACCGCCGCAGCCGGAGCAACAGCTGCAGCTGCCGCAATGGCCGAATCTGTCCGGTCCTCATAACCAGTTGCTTCCTGTGGCGCCGCCGCCATTTGTCG

CTGATCAGCCACCTCCACCTCCACCGCCAGCAGCAGGAGGGTCTCTCTGGATCCCTGAGCTGCCTCCACCTCCGGTGGAGGGGTCGCCATGGGCTGGTCT

GCTTCCGCTGCGACCACCACGGTTTGCCGGGATGGAGCCATCCTTTCTTGAATCACAGCAAGCTCCGCCTCCGGCACAGGTCAGCACACAGCTAGCACCA

CTACCTCTGATACGGGAGGAAGCTCCATTGCTGCAGGAGCCATTTCTGTTTGCTGATCAAGCACCAGTACTTGCTCCTCTGCCGGCGCCATTGCAGATGC

CTGTGGCAGAAACTCACTTGCCACTGCAGGCTCCATTGCTGCAGGAGCCATTTCTATTCTCTGATCATGCACCGACGCTTGCTCCTCTGCCATCGCCATT

GCAGATGCCTGTGGCTGAAACTCACCTGCCACTGCAAGTGCAGGCTCCGTTCATGCAAGAACCATTTCTGTTTTCTGATCAGGCACCAGTGCTTGCTCCT

CCGCCGACGCCATTGCAGATGCCAGTGGAAGCTCACATGCCACTGGAGGCTCCATGGATCCAAGAACCATTTCTCATGCCTGATCAAGCACCGGTCCATG

CTCCTCCACCAACGCCATTGCTGATGCCAGTGGGAGCTGATCACTTCCCACTCGAGGCTCCATTGTTCCAAGAATCACTTATAATGGCTGATCAGAAATC

TGTGCATGCTCTTCCTCCACCTCCATTGCAGATGCCTTTGGAAGCTCACCTGCCACCTGCAGCACAAGAGTATAACCAAGATCTGGCCGTGCAGCAGCAA

CCCCAAGAGTATGAAAACTATGACTACATGTTTGAGAACGTGGGGCTCTCACAGGCACAGCCGGTGGCCGCTGGCGCCGGCGACGCAGGCTTTGCAGCTA

TAGGCAACGACGACAACCCGTTCGGCTACCAACAGTTGGTTGCCTCTCCTCTCTATGATGGACAGATCTACTTCGGCTCCGGGGTTGATAACATGGGCGT

TCCTCCTGCTGGTGATTTTGGTGGTGTTCCTGAAGCTGCTTTGCCGGAAGTCGAGCATGCGTCTTCGTCCGGTTGGGGAAACAACATTACTGGTGATGCT

GGTGCATGGTTCTGA

>LOC_Os01g23750.1 Oryza sativa subsp. japonica|M-type_MADS|M-type_MADS family protein

ATGGCCGGGAGGAAGGAGACCGTGATAAAAATGGCGAAGGAGCTCTCCGTGCTCTGCGACGTCCCCGTCGCGCTCGTCTGCGCCGTCGGCGGCGCGGTGG

AGGTGTGGGAGTCGGAGGAGGGCGTCCTCGACCGGTACCGCGCCCTCCCGCCCGAGGTCCGCGCCACGCGCGCGCACACGCACCGGGGCTACCTCGAGCG

GGAGCTCCGCGCGAGGAGAGCCAAGCTGGCCAAGGTCCGGGAGGAAGGCGCGTTCAAGTCGTGGGGGCGCGACGCGCTCAGCGGCATCATCACGGCGGAG

GAGGCGCCGGCGCTGCTCGAGTCCATCGACGCTGCGATCGCCGCCGCGACCGCGAGGCAGGAGGCGCTTGCTCTACTGGACGGCGGCGGGCTCCATCTGC

AGCACGTCCCGGCGAGCGCTTCCGACGCCGTCGCGCCTGTCGTCGGCGGCCACGGCGTCCAGGTCCAGTACATCGGCGGCAGCGGCGGCGGCGGCGGGAG

CCAGCAGGAGATGACGCCGGCAGCCGACGGAGATGGCGCCCGCAACGCCGACCAGTACGACGACATCCTCCCGTGGGATGGCAACACCTTCGAGGCGCAC

AACGCCCACGTGATGCTGCCCGCGTGCGGCTTCCAATGCACCGGCGACTACCGCGTGGACATGGACGGCTACGTGTGGGGAGCCCCGGATGATGCCAACG

CCTACCATGGCTGGCCCGATGAGGCAATGTGGTGCACCGACGAGTCGTGCTCGTGCAACGCCGCCACCGCCACCGCCGTGCCAGCCATGTATCATCCCCC

CACCCTGGACACCGTCCACGGGAGCTTCCTCGCCGCGCCAGCTCAACCACTCGCCTTCAGCACCGGCGCCGACTTCATCAACGCGCCGAACGACTTTCTC

ACCGTGGGCGTCGGCGGCAGCTTCATCAACGTCGGCGACTACTCGGCGCAGAGCTTGGCCGACGAGTTCCACCACCTCAGCGACGCCACCAACCAGCTCG

ATCAGACGCACTACCCACCATTCGGTGGAACGGGCGGCGCCGAGCCCGGCGACACGCAGTCCCATAGCTGGGGGGACTACTACTTGGCGCAGAGCTCCGC

CAATGAGTGCCAGCTGCTCGGCGTCGACGGCGGCGACATCCATCTCGATCAGACGCATTGCCTCGGTGGTGCGGGCGGCGCCGAGCCCGGCGACACGAAG

TCCCACAATTGGGGTGGCTGA

>LOC_Os01g23760.1 Oryza sativa subsp. japonica|M-type_MADS|M-type_MADS family protein

ATGGCGCGACGAAAGATCCCGATAGGCCTCATCGCGCACCGGCAGAAGCGCGCCGCGACGTACGCGAAGAGGAAGGAGAGCCTGAGGAAGAAGGCGGAGG

AGCTCTCCACGCTCTGCGGCGTCCGCGTCGCGTTCGTCTGCGCGGGCCCCGTCGTCCCCGGCGGCGGCGGCGGCGCGGCGGGGAAGGAGGAGGTGTGGGA

GTCGGAGGAGGGCGTCCTCGCGGAGTACCGCGCCCTCCCGCCGGAGGCTCGCGCGCAGCACGCGCACCGGGTGTACCTCGAGGAGGAGGTCGGCAAGGAG

AGGGCCAAGCTCGCCAGGGTGAGGCAGGACGGCGCGTTCCCGTCGTGGGACGCCGCGCTCGACGGCATCACGGCGGACGAGGCGCGGGCGCTGCTCGAGT

CCATCGACGCGGCGAGGGCGGCCGCGAACGCGAGGCGGGAGGCGCTGGGTCTGCCGGACGACGGCAACGGCGTGGACGATGACGGCGGACTAGATCTGCA

GCAGCAGCAGGAGCACGTCCCGCCGGGCGGTTCTGACGCCGTCGTCGTGCCTGTCGGCCATGGCGTCCTCCAATACACCGGCAGCGGCGGCGGCAACCAG

ATGCAGACGACGCCGGCCGCAGATGGCATCAACTGCGCCGACCTGTACGGCGCCGTTCCGTGGGATGACACCTTCCAGCCGCAGGTGATGCGCACCGGTG

ACCACTTCGTGCCCATGGACGGCTACCTGTGGCAAGCCCCGGGCAATGGCTGGCCCGATCTCGCCACGGGGTGCACCAACGAGTCGTGCTCGTGCAACGC

CGCCGCCGCCGCAGCCGCCATGCCAGCCATGTATCCCCCCACCCTAGACACCGTCCACGGAAGCTTTCTCGCCGCGCCGGCTCAACCCATACCCATCGCA

TTCAGCACCAGCACCGACTTCATCGACGCGCCGAACGACTTCCTCACCATGGGCCTGTGTGGCGGCTTCACCAACGTCGGCGACTACTCGGCGGCGCAGC

CTCAGAGCTCGGCCGATGGAGGATTCCAGCTCGGTGACACGTTCGCCGCCGAGCCCGGCGACACGCAATCCCAGAATTGGGGGAGCTTCATCAACGTCGT

GAGCGACGACTCGGCGCAGTGCAACTGCAACGCCGCAATCCATCTTGATCAGATGTATTACCTATTCGGTGGCACGGGCGGCGGCGAGCCCAGCGACACG

CAGTCCCGGCATTGGGGGAGCTGA

>LOC_Os01g23770.1 Oryza sativa subsp. japonica|M-type_MADS|M-type_MADS family protein

ATGCCGCGCGCGAAGACCCCGATGGGCCTCATCCCGTTCCCGAAGAAGCGCGCCGCGACGTTCGCGAGGAGGAAGGAGACCGTGATGAAAATGGCGAAGG

AGCTCTCGGTGCTCTGCGACGCCCAAGTCGCGGTCCGCGCCAAGCGCTCGCTCACGCACCGGGAGTACCTCCGGGGGGAGCTCCGCAAGCAGAGGGCCAA

GCTGGCCAAGGTCCGGGAGGAAGGCGCGTTCAAGCCGTGGGACGACGCGCTCGACGGCATCGCGGAGGAGGAGACGCGGAAGCTGCACAAGTACCTCAGC

GACAAGATCGAGGCCGCGAGGGCGAGAATGGAGGCGATGGGTCTGCAGCTGGGCGACGTCGACGACAACGGCGTGAACGGCGACGACGGCGGCGGACTCG

ATCTGCAGCAGCACGTCCCGCCGAGCGCTTCCGACGCCAAGGAGTTCGAGTCCGTGCCTGTCGTCCATGGCGGCCAATACATCGGCAGCAGCAGCGGCGG

CGGGGGCGGTGACATCCAGATGCAGACGACGCCGGCCGCAGATGGCATCAGCTTCGCCGAGCACTTTCTCGTCGCGCCGCGCGCTCAGCCCCTCGCCTTC

AGCACCGCCGGCGCCGACTTCATCAACGCACCGAACAACTTCCTCACCACGGGCGTCAGCGTCAGCGACTACTCGGTGCAGAGCTCGGGCTACGGGATCG

GAAACCAGATCGACAACGCCAAGCAACTCCTCTATCAGATGCAAATGCAATACCCAGTCGGTGGCACGGGTGGCGCCGAGCCCAGCAACACGCAGACGCA

GTCCCCGGATTTGCGGAGCTTCAGCAACGTCGTCGGCGACTACTCGTACACGGCGGCGCAGAGCTCGGCCAACCGCCTCGATCAGATGCATCACCCAGTC

GGTGGCCGTGGCAGCACGGGTGGCGCCGCCGCCGATCCCAGCGACACGCAGTCCAAGAATCGGGGGAGCTGA

>LOC_Os01g23780.1 Oryza sativa subsp. japonica|M-type_MADS|M-type_MADS family protein

ATGAAGATGGAGGAGGACGCCACGTACGGGAAGATGCAGGAGTCCCTGATGGAGGAGGCGAGGGAGCTCTCCATCCTCTGCGGCGTCGACGTCGCTCTCC

TCTGTGCCGGCGGCCCCGGCACCGGAGACGGAGACGGCGGCGGTGCGGTGTCGACGGCGGAGGTGGCGGTGTGGGAGTCGGAGGAGGGCGTCCTCGCCTC

GTACCGCGCCATCCTGCCTAGGCCGGAGGCGCACACGCTCCGAGAGTGCCTCGAGTTGAAGCTCGCCAGGGAGAGGGCCAAGCTCGCCATGCTACATCAG

TACGGCGGAGGAGGCGCGGGCGCTGCTCGAGTCCATGGACGCGGCGATGGTGGCCGCGACCGCGAGGCGGGAGGCGCTGCGCTGGGTCTACTGGACGACG

GCAACGCCGTGAACGGCGACGGCGGCGGACCCGATCTGCTGCACATCCCGATCCAGCCGGGCGCTCCCGACTCCGTCGTTGTGCCTGTCATCAGCCATGC

TCAGCCCATCGCCTCCAGCACTGGCGCTGACTTCGCCGACGCGCCCAACGGCTTCCTCGCCATGGACGTCGGTGGCAGCCTCATCAAGTCGGCGACTACT

CAGCGCAGTGCTCGGCCGATGGGTTCCAGCTCGGGGACGCCAACCATGGCCAACCTCGATCAGATACATTACCTAGTCGGTGGCTCGGCATTCTCCATAG

ATGCATTAGAAAGTCCTACACTCCTAGTTAGGTGCAAGACATTGCGAATTGCGATTCAGTTCATGTTGCCGTTGCGCCACGGCCGATTCCGAGCAAATGT

ACCGTCCAACCATCGACGTACGGAGACGAAATCTTAG

>LOC_Os01g67890.1 Oryza sativa subsp. japonica|M-type_MADS|M-type_MADS family protein

ATGGTCCGCACGCCCACCATAATCCCGCTACGGCCCAACTACAGCCCGCCGGGCGACGGTGCCGCATTCCGCCGCGAGCCCGCGACGTATTCCCGTTTCC

CACCCGCCGCGGCTCGCAACGAATGTGATCTCACCTTCGGTCAGGAAAGGAAACGAGAGGGAATGAAAGGCAAGCCCCCGATATCAAGGAAAATCATTTA

TAATCACCGAGATTTTGTTTCCATGGGTGAAAGATTTGTTGCTCTTTGCTTTATATTTACGATGTCTCCATGCATTGATGCAACGCTATTCGTTCGCGTG

GTTTTGGATTTCGTCCGGCGAGAGGAGACGAGACGAGACGAGATGCCTCGCACGAAGCTTGTGTTGAAGCTCATCGAGAACGAGAAGAAGCGGAAGGCCA

CGTTCAAGAATCGCCGGGACGGCCTCAAGCAGAAGGTGTCGCAGTTCGCGACGCTGTGCGGCGTCGAGGCCTTGCTCATCTGCGTCGCCCCCGCCGTGGC

GGGCGGCGAGGTCACGACGTGGCCGCCCGACCGCGCCGCCGTGCTGGACCTCATCGCGAGGCTCCGGGCGACGCCGCCGGAGAAGATCCGGCAGCTGCAC

AACACCCAGTCCCAACTCCGGGACGATCTGGACAAGCAGCAGCGGTTGCTCCTCAAAGTCCAGAAGTGCGGCGCCGACGACGTGCTCACACCGTGGCACT

GCAGCCTGTACGACCTCTCCCTCGACGGCCTCAATGCGCTCCACGACACCCTGTCCGAGACGCTGGACAGGGCGCACCGGCGGATAGCCGCGCTCGGCGG

CGGCCACGGCCACGTCCACGACGACGCCGCGTCGTCCTCCGAGTTCTCGGTCCCCGCGCCCGCGCCGCACGCCGTGGCATTGCCGGACAACGCCTTCGAC

TTCCCGTTCGCCCCGTCGAACACCGGCCCCGTCGTCGGCGCCCACTACTTCTACCCTCTCCACGACACCCTGCCGCTGCCGCTGCCGCTGCCACAGCAGG

TTCCCGGCCAGCATCCGCCGTGCATCGCCTACCAGATGCCGCCGCCGCCGTGCCTCGCCTATCAGATGCCGCCGCCGCCGCCGCCGTCGCTCGCCGCGGC

GCCATTCGACCAGTGTATGAGCGCCACGGGCTTCATGGACAGCAACCCTTACGCCACCCACATCATGCACGGTGGCTCGACGGCTGCTGGCCTCCTCGAC

GATCATGGCCAGATCTTCTCCGCCGGCGCAGGGTACGACGACGACGACATTCTTGGGCACGGCTTCGGCTTCGCCGCCGGCACCGGGTACGATCTTGACC

CGCGCATGGCCACCGCCGACGTTTGGCCGATGAACACGCTCAACAATATTCCAAACGATGGCGGCATCGGCTTCCAGCTGCAGAACGATCTGAAGTGGAT

GCTTCCAGGTGGTAGCAACGGGAGTAATTTGCAAGGCGGCTTTCAGATTTAG

>LOC_Os01g68560.1 Oryza sativa subsp. japonica|M-type_MADS|M-type_MADS family protein

ATGTCTCGCCGGAAGACATCCATAGCCCTCATCGCGAACCCGCAGACGCGCGCTACGACCTACAAGAAGCGGAAGGCGGGGCTGATCAAGAAGGCCGGGG

AGCTCGCCACGCTGTGCGACATCCCCGTCGCCGTCGTGTGCGCCGGGCCCGACGGCGGCGCGCCGACCGTGTGGGTGTCGCCGGAGGGCGGCGACGCCAT

CGAGAGGTACCGCGCGCTGCCGGCGGAGAAGCGGGCGAGGCACACGCACGTGGCTTACCTCCAGGAGGAGCTCGACAAGGAGAGGGCCAAGCTCGCCAGG

CTGCGGCAGAAGGGCCGCCCCGGCGAGCTCGACCCGCCGGACGCCGTGCTGGACGGGATGTCCCAGGACGAGCTGCAGCAGCTGCTCGCGTCCATCGACG

CCACGCTGCTGGCTACGGCCAAGAGGCGGGAGGCGCTCGGGTTGCTGCCCGGCGCCGACGACGACGCGGACGGCGGCGGCCGGCGGCGCGACGCCGACGT

CGCCGGAACCAACTCCGTCGGCGTACACGGCTACCAGCACCAGGAGGTGCATGCACCGGCGACATGTGACCCCTTCCATCCGTACAACGCCGGCGTGACG

CTGATGCAGCCAGGGTACAACAACGCCCAGTACATGGGCGGCCATGGCGCCGTGGACATGAGCGGCTACCAGCTGCAGATGCAGATGCCGGGCAATGGCA

GCAACAACCACAGCCGGCTCGCGTGGGGAGGCTTCCAGCCATGCAACGCCACCTTCGTCCAGCCCGTGTACGGCAACCTCCAATGCTGGTACAACAATGT

CGTCGACGGCAACGGCGAGCCCTGCGACGCCATCGTGCCATCAGCTGGTGACCCCTACATGGACATCGCCGGCAACGACGTCTACGGCAACCAGATGCAG

CCGGCACCGGCGGCCAACGGCGGGTGGCACGATCCGGGCACGTGGGGCTACGACGGCGGCGAGCCGTGCAAAGCCATCGTGCCATCATTTGGTGACCCCT

ACATGGGCATTGGCGTCTACGGCAACCAAATGCAGCCGGCGCCGGCGCCGGCGGCCAACGGCTGCTGGCACAATCCGGCCGGCACATGGGGCAACGACGG

CGAGCCGTGCAACGCCATCGTGCCATCAGCTGGTCACCCCTACATCGACATCGAGTGCGACATCGACGGCAACTACATCGACACAACCGTGTTCGATTAC

CAAACCACCAGCACCAGCGACAACTTCATGGACGCGCCCGTCCAGTTCATCGCCACGGGCAGTGACGAGAGCATCGTCACGAACGTGGCCGGCTGCGACG

AGACGGAGTTCTCCATTGACGATCTCCTCCAGTGCTCCGACGCCTCCCAGCATTCTTCCGGCCTCGAGGAGCTGCATTATCTCAGTGATCTGGCCGATGG

CTTCGACTTTGGATGCAACTTCGACGTGCTATTGGACTGA

#amino acid sequences of MADS-box genes in Oryza sativa

>LOC_Os03g11614.1 Oryza sativa subsp. japonica|MIKC_MADS|MIKC_MADS family protein

MGRGKVELKRIENKISRQVTFAKRRNGLLKKAYELSLLCDAEVALIIFSGRGRLFEFSSSSCMYKTLERYRSCNYNSQDAAAPENEINYQEYLKLKTRVE

FLQTTQRNILGEDLGPLSMKELEQLENQIEVSLKQIRSRKNQALLDQLFDLKSKEQQLQDLNKDLRKKLQETSAENVLHMSWQDGGGHSGSSTVLADQPH

HHQGLLHPHPDQGDHSLQIGYHHPHAHHHQAYMDHLSNEAADMVAHHPNEHIPSGWI*

>LOC_Os12g10540.1 Oryza sativa subsp. japonica|MIKC_MADS|MIKC_MADS family protein

MGRGRIEIKRIENTTSRQVTFCKRRNGLLKKAYELSVLCDAEVALIVFSSRGRLYEYSNNNNVKATIDRYKKAHACGSTSGAPLIEVNAQQYYQQESAKL

RHQIQMLQNTNKHLVGDNVSNLSLKELKQLESRLEKGISKIRARKNELLASEINYMAKREIELQNDNMDLRTKIAEEEQQLQQVTVARSAAMELQAAAAA

QQQQQNPFAVAAAQLDMKCFFPLNLFEAAAQVQAVAAQRQQIIPTELNLGYHHHLAIPGAAAADAPPPHF*

>LOC_Os03g54160.1 Oryza sativa subsp. japonica|MIKC_MADS|MIKC_MADS family protein

MGRGKVQLKRIENKINRQVTFSKRRSGLLKKANEISVLCDAEVALIIFSTKGKLYEYATDSCMDKILERYERYSYAEKVLISAESDTQGNWCHEYRKLKA

KVETIQKCQKHLMGEDLESLNLKELQQLEQQLENSLKHIRSRKSQLMLESINELQRKEKSLQEENKVLQKENPCSFLQLVEKQKVQKQQVQWDQTQPQTS

SSSSSFMMREALPTTNISNYPAAAGERIEDVAAGQPQHVRIGLPPWMLSHING*

>LOC_Os07g01820.1 Oryza sativa subsp. japonica|MIKC_MADS|MIKC_MADS family protein

MGRGKVQLKRIENKINRQVTFSKRRNGLLKKAHEISVLCDAEVAAIVFSPKGKLYEYATDSRMDKILERYERYSYAEKALISAESESEITLPQLTTCTAS

RSTHGICFQYCLMSKTLGNWCHEYRKLKAKIETIQKCHKHLMGEDLESLNLKELQQLEQQLESSLKHIISRKSHLMLESISELQKKERSLQEENKALQKE

LVERQKNVRGQQQVGQWDQTQVQAQAQAQPQAQTSSSSSSMLRDQQALLPPQNICYPPVMMGERNDAAAAAAVAAQGQVQLRIGGLPPWMLSHLNA*

>LOC_Os06g49840.1 Oryza sativa subsp. japonica|MIKC_MADS|MIKC_MADS family protein

MGRGKIEIKRIENATNRQVTYSKRRTGIMKKARELTVLCDAQVAIIMFSSTGKYHEFCSPSTDIKGIFDRYQQAIGTSLWIEQYENMQRTLSHLKDINRN

LRTEIRQRMGEDLDGLEFDELRGLEQNVDAALKEVRHRKYHVITTQTETYKKKVKHSYEAYETLQQELGLREEPAFGFVDNTGGGWDGGAGAGAAADMFA

FRVVPSQPNLHGMAYGGNHDLRLG*

>LOC_Os04g49150.1 Oryza sativa subsp. japonica|MIKC_MADS|MIKC_MADS family protein

MDRSEMGRGRVELKRIENKINRQVTFSKRRNGLLKKAYELSVLCDAEVALIIFSSRGKLYEFGSAGINKTLEKYNSCCYNAQGSNSALAGGEHQSWYQEM

SRLKTKLECLQRSQRHMLGEDLGPLSIKELQQLEKQLEYSLSQARQRKTQIMMEQVDDLRRKERQLGELNKQLKNKLEAEADSSNCRSAIQDSWVHGTVV

SGGRVLNAQPPPDIDCEPTLQIGYYQFVRPEAANPRSNGGGGDQNNNFVMGWPL*

>LOC_Os07g41370.1 Oryza sativa subsp. japonica|MIKC_MADS|MIKC_MADS family protein

MGRGPVQLRRIENKINRQVTFSKRRNGLLKKAHEISVLCDADVALIVFSTKGKLYEFSSHSSMEGILERYQRYSFDERAVLEPNTEDQENWGDEYGILKS

KLDALQKSQRQLLGEQLDTLTIKELQQLEHQLEYSLKHIRSKKNQLLFESISELQKKEKSLKNQNNVLQKLMETEKEKNNAIINTNREEQNGATPSTSSP

TPVTAPDPIPTTNNSQSQPRGSGESEAQPSPAQAGNSKLPPWMLRTSHT*

>LOC_Os01g66030.1 Oryza sativa subsp. japonica|MIKC_MADS|MIKC_MADS family protein

MGRGKIEIKRIENSTNRQVTFSKRRSGILKKAREISVLCDAEVGVVIFSSAGKLYDYCSPKTSLSRILEKYQTNSGKILWDEKHKSLSAEIDRIKKENDN

MQIELRHLKGEDLNSLQPKELIMIEEALDNGIVNVNDKLMDHWERHVRTDKMLEDENKLLAFKLHQQDIALSGSMRDLELGYHPDRDFAAQMPITFRVQP

SHPNLQENN*

>LOC_Os12g31748.1 Oryza sativa subsp. japonica|MIKC_MADS|MIKC_MADS family protein

MGRGKVQVRRIENEVSRQVTFSKRRPGLLKKAHEIAVLCDVDVAAIVFSAKGNLFHYASSHTTMERILEKYDRHELLSEGNNVIEEFPELEGSMSYDHIK

LRGRIEALKKSQRNLMGQELDSLTLQDIQQLENQIDTSLNNIRSRKEKLLMEKNTILEKKITELETLHTCIRASPTKAAAPPACNTADAFVPNLNICCGD

SGEPETVTAPLGWTSSNNGLPWWMLQSSSNGKS*

>LOC_Os01g66290.1 Oryza sativa subsp. japonica|MIKC_MADS|MIKC_MADS family protein

MGRGKIEIKRIENKTSRQVTFCKRRNGLLKKAYELAILCDAEIALIVFSSRGRLYEFSNVNSTRSTIERYKKASASTSGSAPVIDVNSHQYFQQEAAKMR

HQIQTLQNANRHLIGESIGNMTAKELKSLENRLEKGISRIRSKKHELLFSEIEYMQKREADLQNENMFLRAKVAEAERAEHDDQQAAEDDEMAPAPAVGG

GSSSGTELEALPATFDTREYYQPAPPVSMLAAAAAAAAAQYSSDHHQTALHLGYFKVDSGKGGLL*

>LOC_Os02g52340.1 Oryza sativa subsp. japonica|MIKC_MADS|MIKC_MADS family protein

MARERREIKRIESAAARQVTFSKRRRGLFKKAEELSVLCDADVALIVFSSTGKLSHFASSSMNEIIDKYNTHSNNLGKAEQPSLDLNLEHSKYAHLNEQL

AEASLRLRQMRGEELEGLSIDELQQLEKNLEAGLHRVMLTKDQQFMEQISELQRKSSQLAEENMQLRNQVSQISPAEKQVVDTENFVTEGQSSESVMTAL

HSGSSQSQDNDDGSDVSLKLGLPCGAWK*

>LOC_Os08g33488.1 Oryza sativa subsp. japonica|MIKC_MADS|MIKC_MADS family protein

MGRGKIEIKRIDNATSRQVTFSKRRSGLFKKARELSILCDAEVGLLVFSSTSRLYDFASSSMKSIIERYNETKEDPHQTMNASSEAKLWQQEAASLRQQL

HNLQEYHRQLLGQQLSGLDVEDLQNLESKLEMSLKNIRLRKDNVMMDQIQELSRKVVTT*

>LOC_Os04g23910.1 Oryza sativa subsp. japonica|MIKC_MADS|MIKC_MADS family protein

MGRGKIAIKRIDNTMNRQVTFSKRRGGLMKKARELAILCDADVGLIVFSCTGRLYDFSSSSMKSIIERYQEAGEEHCRLLNPMSEAKFWQREVTTLRQQV

QNLHHNNRQLLGEEISNFTVRDLQLLQNQVEMSLHSIRNKKDQLLAEEILKLNEKGSLVQKENSELRKKFNIAHQRNIELHKKLNSGESTSSEQVTRSSK

DPGESSTPRDSRVCIDLELSQKEVEDE*

>LOC_Os08g02070.1 Oryza sativa subsp. japonica|MIKC_MADS|MIKC_MADS family protein

MARGKVQLRRIENPVHRQVTFCKRRAGLLKKARELSILCEADIGIIIFSAHGKLYDLATTGTMEELIERYKSASGEQANACGDQRMDPKQEAMVLKQEIN

LLQKGLRYIYGNRANEHMTVEELNALERYLEIWMYNIRSAKMQIMIQEIQALKSKEGMLKAANEILQEKIVEQNGLIDVGMMVADQQNGHFSTVPLLEEI

TNPLTILSGYSTCRGSEMGYSF*

>LOC_Os02g36924.1 Oryza sativa subsp. japonica|MIKC_MADS|MIKC_MADS family protein

MGRGKIVIRRIDNSTSRQVTFSKRRNGIFKKAKELAILCDAEVGLMIFSSTGRLYEYSSTSMKSVIDRYGKSKDEQQAVANPNSELKFWQREAASLRQQL

HNLQENHRQLMGEDLSGLNVKELQSLENQLEISLRSVRTKKDHVLIDEIHELNRKGSLVHQENMELYKKISLIRQENAELYKKIYETEGPSEVNRDSPTP

YNFAVIEKTNVPVQLGLSTLPQHSDAEQSTAPKLGLQLNP*

>LOC_Os02g07430.1 Oryza sativa subsp. japonica|MIKC_MADS|MIKC_MADS family protein

MGRGKIEIKRIENATNRQVTFSKRRGGLLKKANELAVLCDARVGVVIFSSTGKMFEYCSPTCSLRELIEHYQTVTNTHFEEINHDQQIFVEMTRMRNEME

KLDGGIRRFTGDDLSNLTLADINDLEQQLEFSVTKVRARKHQLLNQQLDNLRRKEHILEDQNSFLCRMINENHHQAAVGGGDVKAMVEMAPVLSMLTAAP

AYYGEESSSTALQLTPPLHAVDAAAAAGFRLQPTQPNLQDPGCSSSSFHAAAAGHGLQLW*

>LOC_Os01g10504.1 Oryza sativa subsp. japonica|MIKC_MADS|MIKC_MADS family protein

MMNMMTDLSCGPSSMTELTAAAAPAGSGSSAAVAAGSSEKMGRGKIEIKRIENTTNRQVTFCKRRNGLLKKAYELSVLCDAEVALIVFSSRGRLYEYANN

SVKSTVERYKKANSDTSNSGTVAEVNAQHYQQESSKLRQQISSLQNANSRTIVGDSINTMSLRDLKQVENRLEKGIAKIRARKNELLYAEVEYMQKREVE

LQNDNMYLRSKVVENERGQQPLNMMGAASTSEYDHMVNNPYDSRNFLQVNIMQQPQHYAHQLQPTTLQLGSRPSISFGVDTVRTHVR*

>LOC_Os06g45650.1 Oryza sativa subsp. japonica|MIKC_MADS|MIKC_MADS family protein

MGQGKIEMKRIEDATRRQVTFSKRRAGFLKKANELAVLCDAQVGVVVFSDKGKLFDFCSPPVILMELFHRYEITTRNTRLQETNRDDEQMVMEITRLRNE

IDQLEASLRRQTGEDLSSVSTVDELSQLQLQLESSLSKVHARKDELMSQQLEDMRRMHQTVHEQNNFLCRMVTKILLLNVITIMILAAMISITHARIALD

DCMQLGYIVIKQENSWRFWFI*

>LOC_Os04g52410.1 Oryza sativa subsp. japonica|MIKC_MADS|MIKC_MADS family protein

MGRGRVELKKIENPTNRQVTFSKRRMGLLKKANELAILCDAQIGVIVFSGTGKMYEYSSPPWRIANIFDRYLKAPSTRFEEMDVQQRIIQEMTRMKDENN

RLRIIMRQYMGDDLASLTLQDVSNLEQQIEFSLYKVRLRKQQLLDQQLLEMHSRVCNKRIYRFSILPYLIHYIEIIKF*

>LOC_Os01g52680.1 Oryza sativa subsp. japonica|MIKC_MADS|MIKC_MADS family protein

MGRGRSEIKRIENPTQRQSTFYKRRDGLFKKARELAVLCDADLLLLLFSASGKLYHFLSPTVPSVREFVERYEATTHTKVWADIRQERRAELEKVGSMCD

LLEKQLRFMTVDDGEEYTVPSLEALEHNLEAAMRKVRSEKDRKIGGEICYLQNIIRGRQEERYGLCDKIAHAQTLKDVECGSTSLSNGLDLKLGFN*

>LOC_Os12g10520.1 Oryza sativa subsp. japonica|MIKC_MADS|MIKC_MADS family protein

MVRGKVQMRRIENPVHRQVTFCKRRGGLLKKARELSVLCDADVGVIIFSSQGKLHELATNGNMHNLVERYQSNVAGGQMEPGALQRQQVAEQGIFLLREE

IDLLQRGLRSTYGGGAGEMTLDKLHALEKGLELWIYQIRTTKMQMMQQEIQFLRNKEGILKEANEMLQEKVKEQQKLYMSLLDLHSQQPTQPMTYGNRFF

SI*

>LOC_Os03g54170.1 Oryza sativa subsp. japonica|MIKC_MADS|MIKC_MADS family protein

MGRGKVVLQRIENKISRQVTFAKRRNGLLKKAYELSILCDAEVALVLFSHAGRLYQFSSSSNMLKTLERYQRYIYASQDAAAPTSDEMQNNYQEYVNLKA

HVEILQQSQRNLLGEDLAPLATNELEQLESQVVRTLKQIRSRKTQVLLDELCDLKRKEQMLQDANRVLKRKLDEIDVEAAPPQPPWNGNCSNGHGGGGGV

FSSEPPQPEHFFQALGLHAVDVNQPPAPPPGGYPPEWMA*

>LOC_Os08g41960.1 Oryza sativa subsp. japonica|MIKC_MADS|MIKC_MADS family protein

MEGGGRRRKRGKVELRRIEDRTSRQVRFSKRRSGLFKKAYELSVLCDAQVALLVFSPAGRLYEFASSTSSIDTIFGRYWDLLDTTIDLNIEARESRVDCN

IQLRQKERSDDPVPKINHITQCVLESNVNELNIAELRGLEEAMTNALTVVKNKLMMKVASVLPQSEKKRKSCSISEPRSGVSS*

>LOC_Os05g34940.1 Oryza sativa subsp. japonica|MIKC_MADS|MIKC_MADS family protein

MGRGKIEIKRIENSTNRQVTFSKRRAGILKKAREIGVLCDAEVGVVIFSSAGKLSDYCTPKTTSVFPPLSRILEKYQTNSGKILWDEKHKSLSAEIDRVK

KENDNMQIELRHMKGEDLNSLQPKELIAIEEALNNGQANLRDKMMDHWRMHKRNEKMLEDEHKMLAFRVHQQEVELSGGIRELELGYHHDDRDFAASMPF

TFRVQPSHPNLQQEK*

>LOC_Os03g08754.1 Oryza sativa subsp. japonica|MIKC_MADS|MIKC_MADS family protein

MAGGGGGGGRGEGEGRAATGKRERIAIRRIDNLAARQVTFSKRRRGLFKKAEELSILCDAEVGLVVFSATGKLFQFASTSMEQIIDRYNSHSKTLQRAEP

SQLDLQGEDSSTCARLKEELAETSLRLRQMRGEELHRLNVEQLQELEKSLESGLGSVLKTKSKKILDEIDGLERKRMQLIEENLRLKEQLQVSRMSRMEE

MQPGPDSEIVYEEGQSSESVTNASYPRPPPDNDYSSDTSLRLGLSLFSSK*

>LOC_Os06g06750.1 Oryza sativa subsp. japonica|MIKC_MADS|MIKC_MADS family protein

MGRGKVELKRIENKISRQVTFAKRRNGLLKKAYELSVLCDAEVALIIFSTRGRLFEFSTSSCMYKTLERYRSCNYNLNSCEASAALETELSNYQEYLKLK

TRVEFLQTTQRNLLGEDLVPLSLKELEQLENQIEISLMNIRSSKNQQLLDQVFELKRKEQQLQDANKDLKRKIQETSGENMLHISCQDVGPSGHASEANQ

EFLHHAICDPSLHIGYQAYMDHLNQ*

>LOC_Os03g03100.1 Oryza sativa subsp. japonica|M-type_MADS|M-type_MADS family protein

MVRGKTQMKRIENPTSRQVTFSKRRNGLLKKAFELSVLCDAEVALIVFSPRGKLYEFASARKIRPEKTAKTIFPRVAIELPSKQSHYFHKEISCETGKVR

NSENIGNLVLAFRRAI*

>LOC_Os06g11330.1 Oryza sativa subsp. japonica|MIKC_MADS|MIKC_MADS family protein

MARERREIRRIESAAARQVTFSKRRRGLFKKAEELAVLCDADVALVVFSSTGKLSQFASSNMNEIIDKYTTHSKNLGKTDKQPSIDLNMRGEELEGLSVE

ELQQMEKNLEAGLQRVLCTKDQQFMQEISELQRKGIQLAEENMRLRDQMPQVPTAGLAVPDTENVLTEDGQSSESVMTALNSGSSQDNDDGSDISLKLG*

>LOC_Os10g39130.1 Oryza sativa subsp. japonica|MIKC_MADS|MIKC_MADS family protein

MVRGRTELKRIENPTSRQVTFSKRRNGLLKKAFELSVLCDAEVALIVFSPRGRLYEFASAPSLQKTIDRYKAYTKDHVNNKTIQQDIQQVKDDTLGLAKK

LEALDESRRKILGENLEGFSIEELRGLEMKLEKSLHKIRLKKTELLEQQIAKLKEKERTLLKDNENLRGKHRNLEAAALVANHMTTTTAPAAWPRDVPMT

SSTAGAADAMDVETDLYIGLPGTERSSNRSETG*

>LOC_Os02g49840.1 Oryza sativa subsp. japonica|MIKC_MADS|MIKC_MADS family protein

MGRGKIVIRRIDNSTSRQVTFSKRRNGLLKKAKELSILCDAEVGLVVFSSTGRLYEFSSTNMKTVIDRYTNAKEELLGGNATSEIKIWQREAASLRQQLH

NLQESHKQLMGEELSGLGVRDLQGLENRLEISLRNIRMRKDNLLKSEIEELHVKGSLIHQENIELSRSLNVMSQQKLELYNKLQACEQRGATDANESSST

PYSFRIIQNANMPPSLELSQSQQREGECSKTAAPELGLHLP*

>LOC_Os05g11414.1 Oryza sativa subsp. japonica|MIKC_MADS|MIKC_MADS family protein

MHIYKEQEAEPSTGLMMPEPAPVASPGSGGSGGSGSVGAEKIGSRGKIEIKRIENTTNRQVTFCKRRSGLLKKAYELSVLCDAEVALVVFSSRGRLYEYS

NNSVKETIERYKKANSDTSNASTVAEINAQHYQQEAAKLKQQITNLQNSNRTLVGDNITTMNHRELKQLEGRLDKGLGKIRARKNELLCAEIEYMQRRET

ELQNDNMYLKSKVAESERGLQTVNMMGSASTSEYVQNMIHYDPRNFLQFNIMHQPQYYPEQEDRKAFMSGKKYSQCNIVRVHSSTNEI*

>LOC_Os06g23950.1 Oryza sativa subsp. japonica|M-type_MADS|M-type_MADS family protein

MVRGKTVISRIENTTSRQVTFSKRRSGLFKKAKELAILCDAQVGVLVFSSTGRLYDYSNSSNSRNLTYDQLLGDTVC*

>LOC_Os02g45770.1 Oryza sativa subsp. japonica|MIKC_MADS|MIKC_MADS family protein

MGRGRVELKRIENKINRQVTFSKRRNGLLKKAYELSVLCDAEVALIIFSSRGKLYEFGSAGITKTLERYQHCCYNAQDSNNALSETQSWYHEMSKLKAKF

EALQRTQRHLLGEDLGPLSVKELQQLEKQLECALSQARQRKTQLMMEQVEELRRKERQLGEINRQLKHKLEVEGSTSNYRAMQQASWAQGAVVENGAAYV

QPPPHSAAMDSEPTLQIGYPHQFVPAEANTIQRSTAPAGAENNFMLGWVL*

>LOC_Os04g38770.1 Oryza sativa subsp. japonica|M-type_MADS|M-type_MADS family protein

MGRGKIVIRRIDNSTSRQVTFSKRRNGIFKKAKELAILCDAEVGLVIFSSTGRLYEYASTSMKSVIDRYGRAKEEQQHVANPNSELKEFCSVFIYITEN*

>LOC_Os08g38590.1 Oryza sativa subsp. japonica|M-type_MADS|M-type_MADS family protein

MGRVKLPIKRIENTTNRQVTFSKRRNGLIKKAYELSVLCDIDVALLMFSPSGRLSHFSGRRGVEDVILRYMNLSEHDRGEAIQNREEIQQEIYSSQQQLQ

ITEDRLRMFEPDPAAFGTSSEVDGCEKYLMELLTRVVERKNNLLSSHMAPFDATTAAMQGADGTQMYVSQADGLATFGGDAAMWGPDGGADPGHPMFSAS

DPLIYLRDHDVYDANSQVAGLHGGDPCAAGGAAAAAAAVGCVDDDVAGGHAAAADAWRQAYTCTELLSTLIPTTPFPLMPHCLGPEDQYLSMEHGMVAAA

QEPVEASTASCSYVPSDENSGTPVMAYDSNPPPANIA*

>LOC_Os06g11970.1 Oryza sativa subsp. japonica|M-type_MADS|M-type_MADS family protein

MGRVKLQIKRIENIPNRQVTFSKRRNGLIKKAYELSVLCDIDIALLMFSPSGRLSHFSGRRRIEDVLTRYINLPESDRGGTIQNREYLINMLTQLKCESD

VTEDLTNTSSKAPVNSNIEELQQEIRRCQHQMQLTEEQLRMFEPDPARSASMEDVEASEKFIAGILSRVEERKRYLLCSMGSFDVTASTSAMQHLYLPQQ

HQHGDITDNGFGSDEVASWVSEGMPPTTSSVASIFAGTSDSMMSFRDQAVYDTMRQDACVDQTVVPEMGMCHVDQQNQSDDWQAYTSAEFLNALIPPTPF

PLDDEDTMGPMLASSPLLMPGIHDQQPPVEDMATAGCSQAPANDGNGLYAAEDIAPVNVG*

>LOC_Os04g31804.1 Oryza sativa subsp. japonica|M-type_MADS|M-type_MADS family protein

MGRRGRVVLRRIEDRVRRGICFRKRLAGLEKKVEELAVLCDAHVGFVVLSCSGDDANPHHFAAPATIENIVERYEHSQAAQKGVHGRCILQKRKSKDFQV

LKETIDKGPINDDMRPIDEKDISTLNMDQISQIEILLEDELRWTRARKVVADRIARLQKKVQKKPATAETESNSTEMPSDHEKKQVAGGSQQSAEEEEEE

MEVVLRHRLSLGTGDRDDGGGGAAEQRHRTTPPPAVDLNVPCRDAGQLQ*

>LOC_Os01g69850.1 Oryza sativa subsp. japonica|M-type_MADS|M-type_MADS family protein

MPPPPPPHSIDPLLLLPHFSPPPHHHTHARTHARLRFPPPTRGGEGGELGFFGAGMARRGRVQLRRIEDKASRQVRFSKRRAGLFKKAFELALLCDVEVA

LLVFSPVGKLYEYSSSSIEGTYDRYQQFAGARRDLNEGSTSINSDENASIHSRLRDITAWSLQNNADESDANQLEKLEKLLTNALRDTKSKKMLAKQNGE

GSRSRANSSGSRGQEEGSA*

>LOC_Os05g11380.1 Oryza sativa subsp. japonica|MIKC_MADS|MIKC_MADS family protein

MHIYKEQEAEPSTGLMMPEPAPAASPGSGSSEGSSIEDTADRQVTFCKRCNGLLKKAYELSMLCDAEVALIVFSSRGRLYEYSNNSVEETIERYKKANSD

TSNTSTVAEINAQHYQQEAAKLKQHITYLQNSNRFIISLSCLDFFFST*

>LOC_Os11g43740.1 Oryza sativa subsp. japonica|M-type_MADS|M-type_MADS family protein

MGRVKLKIKKLENSSGRHVTYSKRRSGILKKAKELSILCDIPLILLMFSPNDKPTICVGDHSSIEDVITKYAQQTPQERAKRKLESLEALKKTFKKLDHD

VNIQDFLGSGGQTVEMLIKSIVWQELSSHLGALQCQMADVEKRLSYWSDPEKVENIDHIRAMEQSLKESLNRIRIHKENFAKQHLMSLQCAAAQFQNDMK

LPLGLTGDPNTSSWFHGGGGAEAQQPMMLPEDPSLLHQRDIGCSASTSLQSYPGYFSMGKQSTDNAGGGEQHHHAAVQQQPEFSQADCLTSLQLGAQFPY

PSAFDNAGLLSDRLFDNAAAAAAAMDFGGHYDLPRPGDEASFQNWASAACGATMYDHQQQQQQQQQPAQLPAAATVEAPSFNHPSPHRQLMI*

>LOC_Os08g41950.1 Oryza sativa subsp. japonica|MIKC_MADS|MIKC_MADS family protein

MAEKKKKKKKKKPQSLLVLTSWRSIGMGRGRVELKRIENKINRQVTFAKRRNGLLKKAYELSVLCDAEVALIIFSNRGKLYEFCSTQSMTKTLEKYQKCS

YAGPETAVQNRESEQLKASRNEYLKLKARVENLQRTQRQYYKSKHRLCLVRSKVWNLVKIRDDVTEKLCMYERNLLGEDLDSLGIKELESLEKQLDSSLK

HVRTTRTKHLVDQLTELQRKEQMVSEANRCLRRKLEESNHVRGQQVWEQGCNLIGYERQPEVQQPLHGGNGFFHPLDAAGEPTLQIGYPAEHHEAMNSAC

MNTYMPPWLP*

>LOC_Os05g23780.1 Oryza sativa subsp. japonica|M-type_MADS|M-type_MADS family protein

MVKGKGRAGRKKVEIKRIEKKDARDVCFSKRRQTLFNKAGELSLLCNANIAAVVISPAGRGFSFAHPSVDDVADRLASMAMGIPNNHSLGGGYHDSGEVT

NIAQQQKIEYVELQKSLEKSEKKKRVQEAMEKERAGHLMQSLTSEVNLLGQDELEELHNKLSALPYTSIAKFYQVLQDAKGTRMPLPQPHIEIACQSQFL

FEEQAVTPANADFPGSST*

>LOC_Os06g22760.1 Oryza sativa subsp. japonica|M-type_MADS|M-type_MADS family protein

MTKRKIEIKRIKNEEARQVCFSKRRPSVFKKASELYTVCGAEVAMLVKSPAGKFFSFGAPSVGFVLSRFHATTTSRKHSSMGVTIQHDNSATIKLHELNQ

QHIELQNQLQAQNEKMKALQEVAKKESGGKVMGWLNSKVEDICQEDLEEFKMVLESLKYLTRGIINQLFQNYAMFSNMMRVQHCVTALPNQQFLPSSEDV

KPMIHHVPSSSYGWNTSIDSKPNSSDAHVVGARRYFPK*

>LOC_Os03g14850.1 Oryza sativa subsp. japonica|M-type_MADS|M-type_MADS family protein

MVKCRARTTRKKIEIKRGDKKVRDACFSKRHTTIFNKANELAILCGVMVAVVFVSPNANGGIFSFGYPSVSSVANRFLANAPNNTSVSSSTQSGRDVEIR

ELEREERELKEHLQASTDQNKLLREAIAARDGGQLMLLLQSDCSELGPKGLVAVVRLWFRNSLVGIGLDRLCWLLQQILFGLDWG*

>LOC_Os12g21850.1 Oryza sativa subsp. japonica|M-type_MADS|M-type_MADS family protein

MVHPLGRTSMGRQRIEIRRIDNKERRQVTFTKRRGGLFKKASELALLTGASVAVVVFSPAKHVYAFGHPSVDAVLRSYASVPGEAAAVAPVPVHGGSGGE

DVDLLGLRLAADDTGAQVAAEHARMRDVAARIVQAKAGRRFWWEADVDALGEAELLEFFTALKKLRDNVGRHANALLAPQPPPLPLQQKQRRRR*

>LOC_Os12g21880.1 Oryza sativa subsp. japonica|M-type_MADS|M-type_MADS family protein

MRPLGRTSKGRQHIDNKERRQVTFTKRRGGLFKKASELALLAGASIAVVVFSETNLAYAFGDPSVDAVLLSYGPVPGEDAEPAPVHSGGLGKDVDLEMLR

HNNCASSLQFVLVSLSFIM*

>LOC_Os06g30810.1 Oryza sativa subsp. japonica|M-type_MADS|M-type_MADS family protein

MVRSRGRPSLGRQRIEIRRIDNSGRRQVTFSKRRNGLFKKASELSTLCGASVAVVAFSSAGNVFAFGQPTVDAVVRRFDPLHADGADPAPAAVEDGGGGG

DDVVVADPEELDALRRAEEQTKAQVAAEQARMRDVGDKVTQAMAGRALWWEADVEALGEAELPEFVRALERLRDSVHRHASTLASTATPLPPPPEQEEEV

PELDVSDYSF*

>LOC_Os06g30830.1 Oryza sativa subsp. japonica|M-type_MADS|M-type_MADS family protein

MGRPRGGTSKGKQKIEMCCIDGKEKRQVTFSKRRRGLFKKASEISTLSGASIAIVSFSKAGNVFAFGSPSVDAVLRRHVVAGPSTSTSHAHAGGDVFADD

GGDNPEVLNALKRATDEAAAEVAAEDARQSGVEGKITEAMAAGRRRFWWDAANVEALGEAELPVFERALHKLRGAVAQGGNNPIQERWDRIPAQGSPGGS

YGWEDLSPTGKLYNQPPFAYRLPVMQSIVSDQLGVVCAMLL*

>LOC_Os09g02780.1 Oryza sativa subsp. japonica|M-type_MADS|M-type_MADS family protein

MESEEARKVCFSKRRADLFKMASELSVHFNADVAAVVFSPAGNRAYSIGDPSVMDRFLSSLPAPAPPAETEPEPEVDWSVMEELSRLCGQLQAMVDAHKA

RLEKAEEKLRESGAAAWMMDLEAEVGRMAPEDVLALVTKLAVLRDGVAERAHEMLREALLAVAAPTPTTPTTPPPAGF*

>LOC_Os09g02830.1 Oryza sativa subsp. japonica|M-type_MADS|M-type_MADS family protein

MVTAAAARRRPSLGRQKIEIRRIESEEARQVCFSKRRAGFFKKASELSILCSADVAAVVFSPAGKAYSFGHPSVEFLLDRFLSSSLPATAGKEEGSSVSV

VAELNRQYGELRAMVDAHKARRERAEKTMEKQRQRQPAAWMDPEAEVGRMAPEELMALGTKLVAVQGGVAARADQMLRDALLLGRRPNTTTTTTTRAPPG

FFHLHPHF*

>LOC_Os01g74440.1 Oryza sativa subsp. japonica|M-type_MADS|M-type_MADS family protein

MAMQVAAPSRRRPSLGRQKIEIRRIESEEARQVCFSKRRAGFFKKASELSILCSADVAAVVFSPAGKAYSFGHPSVECLLERFLPDSSSGAAARVRRGAN

NNGGGGMVGELNRQYGELRAMVEAHKARRERADEKIEMERAAGRWLPMDADVRRMSPEELMAFGTGLMAVQAAVSARADQMLRDALLIGRRPPTTTTAGF

GFFHMPHY*

>LOC_Os09g32948.1 Oryza sativa subsp. japonica|MIKC_MADS|MIKC_MADS family protein

MGRGRVELKRIENKINRQVTFAKRRNGLLKKAYELSVLCDAEVALIIFSNRGKLYEFCSGQSMTRTLERYQKFSYGGPDTAIQNKENELVQSSRNEYLKL

KARVENLQRTQRNLLGEDLGTLGIKELEQLEKQLDSSLRHIRSTRTQHMLDQLTDLQRREQMLCEANKCLRRKLEESNQLHGQVWEHGATLLGYERQSPH

AVQQVPPHGGNGFFHSLEAAAEPTLQIGFTPEQMNNSCVTAFMPTWLP*

>LOC_Os02g06860.1 Oryza sativa subsp. japonica|M-type_MADS|M-type_MADS family protein

MAKAAAEMDVGGGEGRRWKRTRGRQRIEMKLIENKEARQVCFSKRREGVFKKASELSVLCGARVAVVFFSPAGRPHCFGHPSVSAVADRFLLGRSPADAA

AAAAEEEEAVVREFNRVEERLKDALGAAARRRDALDEAARVAGVWNDADVRRAGLADLVSMLAALQRVQAEASERVHDIIVEEAMTHYTGAAAAAANLID

YLDAGPLVSHSPGSHDTTTKLIGGNAVHAPPLSFPPMIMPPPLPPQFSHGFGYTDLAAGYGYNLDHGHGAAYETEEFHNAAACDFF*

>LOC_Os04g25870.1 Oryza sativa subsp. japonica|M-type_MADS|M-type_MADS family protein

MALRKMKLQRIVIDVKRQVTFKKRLNGLTKKVSEFATLFLMVYGEVEVQATKVWPLVWKATRVLEHFKAMPQLDRYKKMTDLEDILNEQVDKLKEELHKV

GRDADESDTKLILVEALNGHRPSLEGLTIEQITSLGLMANAHLKIVNDRLKKLREQGLIPASLLLSGTEVPIQREGWLMDVARGIGSMGNNRFGGTSGSG

IAGSNGDMA*

>LOC_Os03g37670.1 Oryza sativa subsp. japonica|M-type_MADS|M-type_MADS family protein

MFKKRQKSLMKKASELSTLYGVDACVVMYAEGEAQPMMVWPSVPEARRVIERFRALPQKDQYENTTNLEGFLKQRIANLQEKVDKAKHENDELETKLLLL

NSLDCCLPSLVGLTVKQITSLNSMVEERLKKLRGNGLLATPVPTSSKKKIIWDVRYNLKARYVTDDSAKNGSPRLRCTPATSSQRQSSPEFRHRRCLP*

>LOC_Os03g38610.1 Oryza sativa subsp. japonica|M-type_MADS|M-type_MADS family protein

MARNKVKLQRIINDAKRRATFKKRLKGLMKKASELATLCNVDTCLMVYGEGEAQATVVWPSESEVMRVLERFKTLPQLDKYKKMTDLEGFIQERINKFQE

QLDKVRRDADESETKLLLIEALEGRRPGLEGITIEQLTSLGWLVDARLNIVNDQLQKLHEQGLLPASISLPTMGVLPYTTAGYTVAQEAPIQRGGWLMGV

VRGIGSLGYSLFRGSGRSNTAGPSGDMVQPFNIGAGSSLANQGISFPPK*

>LOC_Os01g18420.1 Oryza sativa subsp. japonica|M-type_MADS|M-type_MADS family protein

MARNRIILKKVAKDSTRRLTFKKRRRGLIKKAGELASLCGIGVCVVVYGEGEVKPEVWPSAPEARAILSRFNSAPNIDRFKRVTNQEQYLRKRIAKARER

TSKADDVNRERDATIMLYEAATDKRPVADLNVQELTNLGLVINERINHLKERIERLGGAALMAPPPSTQPTEASSSLPPLVPYANGAGMEGNKRMKVSTH

QKGWFINMSTMTGDAGTSADVEGNTGVGTSARGDMMHLSN*

>LOC_Os01g18440.1 Oryza sativa subsp. japonica|M-type_MADS|M-type_MADS family protein

MARKKIVLDRIANDATRRATFKKRRRGLLKKASELATLCDVDACLVVYGEGDAEPEVWPSTEVAMNVLRQFRALPEMEQCKKMMNQEDFLRLRIGKLKEQ

LRKMDRDNHERETLILLHDALQGRLGTYESLSVEQLTSVDCLASARLKVITDRLVEIRAPNEDGQVLVPPPPPPPPALPAPPPPPAPMLPLAPPPTHVTP

AMPLSSMPPPAFHGMNHHHHQNHFINHGGNDQNAWLMNVARNGGDLGALVYSAFASSSSSNTGGAGTSAAGAAAPGPDMMDLANPDMPGFGCPWDDDSAG

PSFPPM*

>LOC_Os07g04170.1 Oryza sativa subsp. japonica|M-type_MADS|M-type_MADS family protein

MEREFCGEMESQICQFGTFLEVNNQHSATPKGKTIPVKCTDCLPGVASRSLTVIPLEVPHDVLLSAREAKTQYNSTFEHNLSETNFVTGTYNGAHHANVK

DVPPDIYETEFRHQFPRIETSFSIIGAHFLSSMVRRGRRKGVRYIEEDRDRSLTLSKRRDGLFKLANDLSLLTDASVAICLHDSNKAQFFGAPSVKPVVD

AFVSEAEPFADEQLKAKLTSMQSELVQLENEEEEKDKKTEESIQRFKEAQEESLGMGMAKHLFSRLEDLSHDDMRELLDVLLPLQQDFKKRLPPLRRGSK

LQIGGSSAWAHQQPSCSRFLASHRPFTPLLPGGTSGVPMIPPPPVPGSPWSQIFPLRPPLFPSPELVPSQQLPPVSPPQNTVAPPPMHAPLVQQPLTNQS

SAVPLLTQWQMRFGDQPPAEVQACTPVEQPQNDNAVHTPTFSDSFLLELLADVSDDGIATAEPLCSPPIDDQFLADIDWLAELDTIDGNL*

>LOC_Os01g11510.1 Oryza sativa subsp. japonica|M-type_MADS|M-type_MADS family protein

MPRRARRTGAAYVDDERERDITFFKRRNGLFKCASDLSILTGASVAVVIEDQNRSKFHAVGTPTVQAVVDAALSSDVEEAAAEARPVADEQLMERIAPLE

RELAWLKGEAAEKDETTKASKARFKMAQKKEENEEEGDTKKKKLFFSKADKLSSDEMNELLAEMLEIKKELNVRLPPLRRRGGKRPIQGSSVPPPPPPPP

PPPQPEQQLQLPQWPNLSGPHNQLLPVAPPPFVADQPPPPPPPAAGGSLWIPELPPPPVEGSPWAGLLPLRPPRFAGMEPSFLESQQAPPPAQVSTQLAP

LPLIREEAPLLQEPFLFADQAPVLAPLPAPLQMPVAETHLPLQAPLLQEPFLFSDHAPTLAPLPSPLQMPVAETHLPLQVQAPFMQEPFLFSDQAPVLAP

PPTPLQMPVEAHMPLEAPWIQEPFLMPDQAPVHAPPPTPLLMPVGADHFPLEAPLFQESLIMADQKSVHALPPPPLQMPLEAHLPPAAQEYNQDLAVQQQ

PQEYENYDYMFENVGLSQAQPVAAGAGDAGFAAIGNDDNPFGYQQLVASPLYDGQIYFGSGVDNMGVPPAGDFGGVPEAALPEVEHASSSGWGNNITGDA

GAWF*

>LOC_Os01g23750.1 Oryza sativa subsp. japonica|M-type_MADS|M-type_MADS family protein

MAGRKETVIKMAKELSVLCDVPVALVCAVGGAVEVWESEEGVLDRYRALPPEVRATRAHTHRGYLERELRARRAKLAKVREEGAFKSWGRDALSGIITAE

EAPALLESIDAAIAAATARQEALALLDGGGLHLQHVPASASDAVAPVVGGHGVQVQYIGGSGGGGGSQQEMTPAADGDGARNADQYDDILPWDGNTFEAH

NAHVMLPACGFQCTGDYRVDMDGYVWGAPDDANAYHGWPDEAMWCTDESCSCNAATATAVPAMYHPPTLDTVHGSFLAAPAQPLAFSTGADFINAPNDFL

TVGVGGSFINVGDYSAQSLADEFHHLSDATNQLDQTHYPPFGGTGGAEPGDTQSHSWGDYYLAQSSANECQLLGVDGGDIHLDQTHCLGGAGGAEPGDTK

SHNWGG*

>LOC_Os01g23760.1 Oryza sativa subsp. japonica|M-type_MADS|M-type_MADS family protein

MARRKIPIGLIAHRQKRAATYAKRKESLRKKAEELSTLCGVRVAFVCAGPVVPGGGGGAAGKEEVWESEEGVLAEYRALPPEARAQHAHRVYLEEEVGKE

RAKLARVRQDGAFPSWDAALDGITADEARALLESIDAARAAANARREALGLPDDGNGVDDDGGLDLQQQQEHVPPGGSDAVVVPVGHGVLQYTGSGGGNQ

MQTTPAADGINCADLYGAVPWDDTFQPQVMRTGDHFVPMDGYLWQAPGNGWPDLATGCTNESCSCNAAAAAAAMPAMYPPTLDTVHGSFLAAPAQPIPIA

FSTSTDFIDAPNDFLTMGLCGGFTNVGDYSAAQPQSSADGGFQLGDTFAAEPGDTQSQNWGSFINVVSDDSAQCNCNAAIHLDQMYYLFGGTGGGEPSDT

QSRHWGS*

>LOC_Os01g23770.1 Oryza sativa subsp. japonica|M-type_MADS|M-type_MADS family protein

MPRAKTPMGLIPFPKKRAATFARRKETVMKMAKELSVLCDAQVAVRAKRSLTHREYLRGELRKQRAKLAKVREEGAFKPWDDALDGIAEEETRKLHKYLS

DKIEAARARMEAMGLQLGDVDDNGVNGDDGGGLDLQQHVPPSASDAKEFESVPVVHGGQYIGSSSGGGGGDIQMQTTPAADGISFAEHFLVAPRAQPLAF

STAGADFINAPNNFLTTGVSVSDYSVQSSGYGIGNQIDNAKQLLYQMQMQYPVGGTGGAEPSNTQTQSPDLRSFSNVVGDYSYTAAQSSANRLDQMHHPV

GGRGSTGGAAADPSDTQSKNRGS*

>LOC_Os01g23780.1 Oryza sativa subsp. japonica|M-type_MADS|M-type_MADS family protein

MKMEEDATYGKMQESLMEEARELSILCGVDVALLCAGGPGTGDGDGGGAVSTAEVAVWESEEGVLASYRAILPRPEAHTLRECLELKLARERAKLAMLHQ

YGGGGAGAARVHGRGDGGRDREAGGAALGLLDDGNAVNGDGGGPDLLHIPIQPGAPDSVVVPVISHAQPIASSTGADFADAPNGFLAMDVGGSLIKSATT

QRSARPMGSSSGTPTMANLDQIHYLVGGSAFSIDALESPTLLVRCKTLRIAIQFMLPLRHGRFRANVPSNHRRTETKS*

>LOC_Os01g67890.1 Oryza sativa subsp. japonica|M-type_MADS|M-type_MADS family protein

MVRTPTIIPLRPNYSPPGDGAAFRREPATYSRFPPAAARNECDLTFGQERKREGMKGKPPISRKIIYNHRDFVSMGERFVALCFIFTMSPCIDATLFVRV

VLDFVRREETRRDEMPRTKLVLKLIENEKKRKATFKNRRDGLKQKVSQFATLCGVEALLICVAPAVAGGEVTTWPPDRAAVLDLIARLRATPPEKIRQLH

NTQSQLRDDLDKQQRLLLKVQKCGADDVLTPWHCSLYDLSLDGLNALHDTLSETLDRAHRRIAALGGGHGHVHDDAASSSEFSVPAPAPHAVALPDNAFD

FPFAPSNTGPVVGAHYFYPLHDTLPLPLPLPQQVPGQHPPCIAYQMPPPPCLAYQMPPPPPPSLAAAPFDQCMSATGFMDSNPYATHIMHGGSTAAGLLD

DHGQIFSAGAGYDDDDILGHGFGFAAGTGYDLDPRMATADVWPMNTLNNIPNDGGIGFQLQNDLKWMLPGGSNGSNLQGGFQI*

>LOC_Os01g68560.1 Oryza sativa subsp. japonica|M-type_MADS|M-type_MADS family protein

MSRRKTSIALIANPQTRATTYKKRKAGLIKKAGELATLCDIPVAVVCAGPDGGAPTVWVSPEGGDAIERYRALPAEKRARHTHVAYLQEELDKERAKLAR

LRQKGRPGELDPPDAVLDGMSQDELQQLLASIDATLLATAKRREALGLLPGADDDADGGGRRRDADVAGTNSVGVHGYQHQEVHAPATCDPFHPYNAGVT

LMQPGYNNAQYMGGHGAVDMSGYQLQMQMPGNGSNNHSRLAWGGFQPCNATFVQPVYGNLQCWYNNVVDGNGEPCDAIVPSAGDPYMDIAGNDVYGNQMQ

PAPAANGGWHDPGTWGYDGGEPCKAIVPSFGDPYMGIGVYGNQMQPAPAPAANGCWHNPAGTWGNDGEPCNAIVPSAGHPYIDIECDIDGNYIDTTVFDY

QTTSTSDNFMDAPVQFIATGSDESIVTNVAGCDETEFSIDDLLQCSDASQHSSGLEELHYLSDLADGFDFGCNFDVLLD*

#nucleotide sequences of MADS-box genes in Solanum lycopersicum

>Solyc01g087990.2.1 Solanum lycopersicum|MIKC_MADS|MIKC_MADS family protein

ATGGGTAGAGGAAAAATTGACATAAAATTGATTGAGAATCTAAATAATAGACAAGTAACATTTTCTAAGAGGCGTGCTGGCTTGTTGAAGAAAGCCGGGG

AGCTTTCTGTTCTTTGTGATTCTGAAGTTGCTGTTATTATTTTCTCAAGTACTGGAAAGCTTTTTGAGTTTTCAAGTACTAGCATGAAACAGACGCTTTC

CAGATACAACAAATGTGTAGCCTCGACAGATAATTCTGCCGTAGAAAAGAAGTCAGAGGACAACGAGCAGCCACAGTTGCAGCAGCAGACACATGTGCTG

AAGCAGGAACAAAAAGAGGTGGACAGTCTTAAAGACGAACTCGCAAAGCTCAAGATGAAACAACAGCGGTTGTTAGGCAAGGACCTTAATGGTATGGGTT

TGAATGAGCTACGGCTTCTTGAACATCAACTAAATGAAGGACTACTAGCCATAAAGGAGAGAAAGGAGGAATTGCTGATACAGCAACTAGAGTATTCTAG

GAAACAGGAGGAGAGGTCTGCGCTGGAGTGTGAGACCTTACGTAGACAGGTAGAAGAGCTCCGAGGGTTATTTCCTTTAAGTGCTAGTTTACCGCCACCT

TTTCTTGAATATGATCGCCCATTGGAAAAGAAGTATTCAATTTTAAAAGAGAGTAAGGAGAGTCTGGATTCCGACACTGCATGTGAAGATGGAGTAGATG

ATGAAGATTCCAACACAACTTTGCAATTGGGGCTTCCAACTATTTGTCGAAAGAGAAAGAGAACAGAGCAGGAATCTCCTTCAAGCAATTCAGAGAATCA

AGTTGGCTCAAAGTGA

>Solyc01g093960.2.1 Solanum lycopersicum|MIKC_MADS|MIKC_MADS family protein

ATGGGGAGAGGGAGAGTGGAACTAAAGAGAATAGAGAACAAAATCAACCGTCAAGTGACATTTTCTAAGAGGAGGAATGGTTTGTTGAAGAAAGCTTATG

AATTATCAGTGCTTTGTGAGGCTGAAGTTGCTCTCATCATCTTCTCTAGTCGTGGAAAGCTCTATGAGTTTGGTAGTGCAGGTATCACTAAAACCCTTGA

GAGGTACCAACGTTGTTGCCTTAATCCTCAAGACAATTGTGGTGAAAGAGAAACACAGAGCTGGTACCAAGAGGTCTCTAAATTAAAGGCCAAGTTTGAA

GCACTTCAACGAACTCAAAGGCACTTGCTTGGTGAAGATCTTGGAGCACTAAGTGTGAAGGAGTTGCAAAATCTTGAAAAACAACTTGAAGGTGCACTTG

CACAAGCTAGACAAAGAAAGACACAAATAATGATGGAACAGATGGAGGAGCTTCGTAGAAAGGAGCGTCATCTTGGTGATGTGAACAAGCAGTTGAAGAT

TAAGGTTTCTCTTGAACTATCATCGTTTGAGGGTGAAGGACAAGGTGTTCCTTTTCCATGGAGTAATTGTAATGCATCTTTAGATGAAGCAGGAAGCAGC

ACCTTTCATGTCCACCATTCTCAATCAAATCACATGGACTGTGATTTACCTGATCCAGTTCTTCAAATAGGGTATCATCAGTATATGGCTGCAGATGGAG

CCTCAGGGTCAAGGAACATGGCTGTTGAGAGTAACATTATCCATGGTTGGGGTCTTTAA

>Solyc01g105800.2.1 Solanum lycopersicum|MIKC_MADS|MIKC_MADS family protein

ATGGAACAAATAACTATTCAATTAAAAAGCTCCAACTCAAACTCGCATGAAGAAATACAATTACTCCTTTGTAACCAAAAATACCCTAATTACTACTTTT

TCTCTTTCTATTGTAATAACATACCTTTTTTTTCTTTCTTCAGCTCACAACTTCAGATCTCTTCTCCATTTCTTGCAAATTTCAGTAAGGAAATGGTGAG

ACAAAAAATTCAGATCAAGAAGATAGACAATTTGACAGCAAGACAAGTGACATTTTCAAAGAGAAGAAGAGGGCTTTTCAAAAAAGCTCAAGAGCTTTCA

ACTCTTTGTGATGCTGATATTGGACTCATTGTTTTCTCTGCTACTGGAAAACTTTTTGAGTATTCAAGCTCCAGCATGATGCAACTGATTGAGAAGCACA

AGATGCAGTCAGAAAGGGATGGTATGGATAATCCAGAACAACTGCATTCTTCTAACATTCTGAGCGAGAAGAAAACCCATGCAATGCTTAACAGGGACTT

TGTGGAGAAGAATCGGGAGTTAAGGCAACTACATGGAGAAGAGCTGCAAGGACTTGGTTTGGATGAATTAATGAAATTGGAGAAATTAGTCGAGGGAGGA

ATAAGTCGTGTCCTCAAAATTAAGGGTGACAAGTTTATGAAAGAGATCAGTTCCCTCAAGAAAAAGGAAGCTAAACTCCAGGAAGAGAATTCACAGTTGA

AAAAGCAATCACAAGCAAGATTGAATGAAGAAGGGCAAAATGTAATTGAGCAAGGACATTCAGCAGACTCCATCACGAACAATCGCAGCTTAGTCAATGA

CTCAGATACTAGTCTCAAGTTATGCTTGGCTTTTCCTTAG

>Solyc02g065730.1.1 Solanum lycopersicum|MIKC_MADS|MIKC_MADS family protein

ATGGGGCGGGGTAGGGTGGAGATGAAGCGTATCGAAAATAAAATAAGCAGACAAGTTACATTCTCAAAGAGACGATCCGGTTTGTTGAAGAAAACCAACG

AGATCTCTGTGCTATGTGATGCTGAGGTGGCATTAATTGTTTTCTCTTCAAATGGAAAACTATTTGAGTACTCTACTCAATCAAGCATGGAAAATATATT

GGAAAGATATGAAAATTACTCATACGAGGAGATGAACTTGAATACAACTTATAAGGAAAATTGGACTCTTGAGTACCCAAAGCTCATGGCAAGAGTTGAA

CTTCTGCAAAGAAATATAAGGCATTTTATGGGAGAAGATCTGGACGCCTTTAATCTGCGTGAATTTCGGGGTTTAGAGAAACAGCTCGATACAGCTCTAA

AGCGAGTGCGATCTAAGAAGAACCAACTGATGCACGAGTCCATTTCCCAGCTGCAGAAAAAGGAAAAAGAACTGCAACAGCGAAACAACTTAATTTCTAA

CAAGCTTAAAGAAAATGAGAAGAAGCAAATTGTGCAAACAAATCCAGGCCAAAGCTCTACTATGACTTTCTTGCTACAATCTCCCACAGTCACTAACCAA

ACAATTGGCGGTCCTTCTCAAGCAACGGATCAAAGTCAAAATCGCGATGGTTACAATAGCTTGATGCCTCCATGGATGTTCCACCATGTCCACAACAAAG

GATGA

>Solyc02g071730.2.1 Solanum lycopersicum|MIKC_MADS|MIKC_MADS family protein

ATGGACTTCCAAAGTGATCTAACCAGAGAGATCTCACCACAAAGGAAACTAGGAAGGGGGAAAATTGAGATCAAAAGGATCGAAAACACGACGAATCGAC

AAGTAACATTCTGCAAGAGGCGCAATGGTTTGCTTAAAAAGGCTTATGAATTGTCTGTGCTCTGTGATGCTGAGGTTGCTTTGGTTGTCTTCTCAAACAG

AGGCAGACTCTATGAGTATGCCAACAACAGTGTGAAAGCAACAATCGAGAGGTACAAGAAAGCATGCTCAGATTCCTCAAACACTGGTTCAGTATCCGAG

GCCAATGCTCAGTATTACCAGCAAGAAGCCTCCAAACTGCGCGCACAAATTGGAAATCTGATGAACCAAAACAGGAACATGATGGGTGAAGCTCTTGCTG

GAATGAAACTCAAAGAACTGAAGAATCTGGAGCAAAGAATTGAAAAAGGGATTAGCAAAATCCGATCCAAAAAGAATGAGCTGTTGTTTGCTGAAATTGA

GTATATGCAGAAGAGGGAAGTTGATTTACACAACAACAATCAGTACCTGAGAGCAAAGATTGCTGAAACTGAGAGAGCTCAGCATCAGCATCAGCAGATG

AACTTGATGCCAGGGAGTTCATCAAACTATCATGAGCTTGTGCCTCCACCTCAGCAATTCGATACTCGAAACTATCTACAAGTTAATGGATTGCAAACCA

ACAACCATTACCCTAGACAAGACCAACCCCCTATTCAACTAGTCTAA

>Solyc02g084630.2.1 Solanum lycopersicum|MIKC_MADS|MIKC_MADS family protein

ATGGGCCGTGGAAAAATTGAGATCAAGAAGATTGAAAACTCGACAAACAGGCAGGTCACTTACTCCAAGAGAAGAAACGGTATTTTCAAGAAAGCTAAAG

AACTTACTGTTCTTTGTGACGCTAAGATCTCTCTCATCATGCTATCAAGCACCAGGAAGTATCATGAGTACACAAGCCCAAACACTACGACAAAAAAGAT

GATTGATCAGTATCAGAGTGCACTTGGAGTTGATATCTGGAGCATTCACTACGAGAAAATGCAAGAAAACTTGAAGAGATTGAAAGAGATCAATAACAAG

CTAAGAAGAGAGATAAGGCAGAGAACAGGGGAAGACATGAGCGGACTAAATTTGCAGGAACTATGTCACTTGCAGGAGAACATCACTGAATCTGTTGCTG

AGATTCGTGAACGAAAGTACCACGTGATCAAGAATCAAACAGACACCTGCAAGAAGAAGGCGAGGAACTTAGAAGAGCAAAATGGAAACCTTGTACTTGA

CTTGGAAGCAAAATGTGAAGATCCAAAGTATGGTGTTGTGGAAAATGAGGGGCATTACCACTCTGCTGTGGCATTTGCGAATGGAGTACACAATCTTTAT

GCTTTTCGCCTACAACCATTGCACCCCAATCTTCAAAACGAAGGAGGATTTGGTTCTCGTGATCTACGTCTCTCCTGA

>Solyc02g089200.2.1 Solanum lycopersicum|MIKC_MADS|MIKC_MADS family protein

ATGGGTAGAGGAAGAGTTGAGCTGAAGAGGATAGAAAACAAGATAAATAGACAAGTCACTTTTGCAAAGAGGAGAAATGGATTGCTCAAAAAAGCTTATG

AACTATCTGTGCTTTGTGATGCTGAAGTTGCTCTACTCGTTTTCTCTAATCGTGGAAAACTCTATGAATTCTGCAGCACAAACAATATGCTCAAAACACT

TGATAGGTACCAAAAGTGCAGCTATGGAACATTGGAAGTCAATCGATCAATCAAAGATAATGAGCAAAGCAGCTATAGGGAATACTTGAAACTCAAAGCC

AAATATGAGTCGCTGCAGCGATATCAAAGACACCTTCTTGGAGATGAGTTGGGGCCTCTGACTATAGATGATCTTGAGCATCTTGAAGTCCAACTAGATA

CTTCCCTCAAACACATTAGGTCCACCAGGACACAAATGATGCTTGATCAGCTTTCTGATCTTCAAACTAAGGAGAAATTGTGGAATGAGGCTAACAAGGT

TCTTGAAAGAAAGATGGAAGAAATATATGCTGAAAACAACATGCAACAAGCATGGGGTGGTGGTGAGCAAAGTCTCAATTATGGTCAGCAGCAACATCCT

CAATCTCAGGGTTTCTTCCAACCTCTAGAGTGCAACTCTTCCTTGCAAATTGGGTACGATCCAATAACAACTTCAAGCCAAATAACAGCAGTAACAAATG

CCCAAAACGTGAATGGTATGATACCTGGTTGGATGCTGTGA

>Solyc02g089210.2.1 Solanum lycopersicum|MIKC_MADS|MIKC_MADS family protein

ATGGGAAGAGGTAGGGTAGAGTTGAAACGGATCGAGAACAAAATAAGCAGACAAGTAACATTCTCAAAGAGACGATCTGGATTATTGAAGAAAGCTAATG

AGATCTCAGTATTATGTGATGCTGATGTTGCATTGATTGTGTTTTCTACCAAAGGCAAACTTTTCGAGTATTCCTCAAATGACTCAAGTATGGAAAGTAT

TCTTGAAAGATATGAAAGATGCTCATATGCAGAGAGACAGATGAATGCTAATGATTCTGATCCCAAGGAAAATTGGAGTGTGGAGTATCCGAAGCTCATG

TCAAGAATTGAACTTTTACAAAGAAATATAAGGCATTACATGGGTCAGGATCTGGACCCTCTCAGTTTGCGTGAGCTCCAGAGTATAGAGCAACAGATTG

ATACTTCATTAAAGAGAATTAGAAGCAGGAAGAATCAACTGATGCACGAGTCCATTTCTGAGCTGCAGAAAAAGGAGAAAGCGCTCCAAGAACAAAACAA

CTTGATTACTAAGAAGCTAAAAGAAAATGAGAAGACACAACCCAACTCATCAGGCCAAAACTCAGCAACAGTTCATGTGTTTCCATCACATTCTCACCAC

CAACTTCCTAACCTTACAATTGGGGGGGCTTTTGGAGGAATGAACAGAGATGGATCGGGTCAGGCTCATCACTATCCGGGTTCAAATAACAATAATAATA

ATTCCTCATTGATACCGCCATGGATGCTCCGCCACGTCAGCAACGAAGGATGA

>Solyc02g091550.1.1 Solanum lycopersicum|MIKC_MADS|MIKC_MADS family protein

ATGGGAAGGGGAAAGATAGTGATCCGAAGGATCGATAACTCGACGAGTAGACAAGTTACGTTCTCAAAGAGAAGGAATGGATTGTTGAAGAAAGCTAAGG

AGCTTGCGATTCTTTGTGATGCTGAAGTTGGATTGATTATTTTCTCTAGTACTGGGAAGCTCTATGAGTTTTCTAACACAAGCATGAAATCAGTTATTGA

ACGATACAACAAAACGAAGGACGATTGTCAGCAGTTGCACAATCCAGTTTCAGAACTCAAGTTGTGGCAGAGGGAGGCAGAGATTTTGCGGCAACAACTA

CAGGACCTGCAAGATAATCATCGGCAATTATTGGGAGAGGAGCTCAGTGGTTTGGGCGTAAAAGAACTAACTAATCTGGAAAATCAACTGGAAATGAGCT

TAAAGGGCATCCGTATGAAAAAGGAGCAAATATTAAAGGACGAAATTCAAGAGCTAACTCGAAAGGGGAGCATTATACATCAAGAAAATATGGAACTCTA

CAAGAAGGTAAATCTCATTCGACAAGAAAATGCAGAATTGTATAAAAAGGCTTATGGTGCAAGAGATGCTAATGCAGTGAATGGAAACATCAACTATCCG

TATCGCTTCACTGTGAGTAGAGAAGTTCAGGCACCCATCCATCTGCAGCTAAGCCAGCCTGAGCCACAATATTTTGAGATGCAAGCAGGAACATCTGATT

CAAGGTAG

>Solyc03g006830.2.1 Solanum lycopersicum|MIKC_MADS|MIKC_MADS family protein

ATGAAAAGAATAGAAAATTCAACGAGCAGGCAAGTGACGTTTTCGAAAAGACGAAATGGACTTACGAAGAAAGCTTATGAATTATCAGTTCTTTGTGATG

CTGAAGTTGCTTTCATTATTTTCTCACATAAAGGAAGACTTTATGAATTTGCTAGCTCCAACATGCAGAAGATAATTGAGAGATACCGAGGACGTGCAAG

AGAAACAACGACGGTGGACAAAAGCACTGAACTCGAGCACTACATGGAGAACTTGAAGCATGAAACAGCTAATATGGCGAAGAAGATAGAGATCCTCGAA

ATTTCTAAACGGAAGCTAATGGGGCAAGGATTAGGGTCATGTTCAATGGATGAACTAGAAGACATTGACAGCCAACTGGAGAGGACCCTCAAAATTATCA

GGGCTAGAAAGACTCAATTGTTCAAAGAGGAAATAGAAAGTCTAAAAGCAAAGGAGAGACTATTGCTCCAACAAAATGCAAGTTTACGTGAAAAGTGCGG

GCTTAGGCCAATGCTATCAGAGTCAGCATCTGCACCCGAACCCATACCAGCACCACCATCAACACCACCAGCTCAATCAAAAGAAAGGGGAAATTGTAGC

CAAAGTACAAAGAGTTGGGAAGTGGAGACTGAATTGTTTATTGGCCTTCCTCAAACGCGCTGCTTATAG

>Solyc03g019710.2.1 Solanum lycopersicum|MIKC_MADS|MIKC_MADS family protein

ATGGGGAGAGGAAAAGTTGAATTGAAGAGAATAGAGAATCAAACAAATAGGCAAGTTACCTTCTCCAAGAGAAGAAATGGTTTACTTAAAAAAGCTTATG

AACTCTCTATTCTATGTGATGCTGAAGTTGCCCTTCTCCTTTTCTCTCCTTCTGGCAAAGCTTATCATTTCGCGAGCCACGACATCGAAAGGACTATTTT

AAGGTACAAGAATGAAGTTGGATTGTCCAAAAATAGTGATCAAGGCCCCAGAGCTATGGAGGTTTGGAGAACTAAGATTGATGACATGACAAGAACAATA

CATGAACTTGAAGCTAGAGATAAGCATTTTGCTGGAGAAGAGTTATCAAATCTTGGTATGAAAGAATTGAAGCAGTTGGAGCGTCAACTCAGAGTTGGAG

TTGAACGCATTCGATCTAAAAAGCATAAAATCCTTCATGAGGAGAACATCCATCTTCAAAAGCAAGTAAAATTATATGAAGTAGAAGGGAGCTCAAGGAT

TCTCGATACAAATCCAAGGATGAGGATTATATGA

>Solyc03g114830.2.1 Solanum lycopersicum|MIKC_MADS|MIKC_MADS family protein

ATGGGTAGAGGAAGAGTACAATTGAAGAGAATTGAGAACAAAATTAATCGTCAAGTTACTTTTTCAAAGAGGCGATCTGGTTTGCTTAAAAAAGCTCATG

AGATCTCTGTGCTTTGCGATGCTGAAGTTGGACTCATTGTTTTCTCAACTAAAGGAAAACTCTTTGAGTATTCTACTGACTCTTGCATGGAAAGGATTCT

TGAAAGGTATGAAAGGTACTCATATGCTGAAAGGCAGCTTAATGCTACTGATATTATAACCCCGGGTAGCTGGACTTTGGAACATGCTAAGCTTAAGGCC

AGACTTGAGGTTTTGCAAAGAAACCAAAAGCATTATGCAGGAGAAGAGTTGGACACATTGAGTATGAAAGAGCTTCAGAATCTGGAACACCAGCTCGATT

CTGCTCTTAAGCACATTCGCTCTAGAAAGAACCAATTGATGCATGAATCCATTTCTGAGCTTCAAAAGAAGGACAAGGCATTGCAAGAACAAAACAACAA

TCTTTCAAAGCAGGTTAAGGAAAGGGAGAAAGAGATGGCCCAACAGACTCCGTGGGAGCAACAGAGTCATGATCATCTCAATTCATCTTCGTTTGTTTTG

CCACACCCCTTTAACAATCTTCACATAGGGGAAGCATACCCAAATGCAGGAGACAATGGAGAAGTAGAAGGATCATCGCGGCAACAACAACAAAACAGTG

CTTCTGTGATGCCTCCATGGATGCTTCGCCATCTCAACGGTTAA

>Solyc03g114840.2.1 Solanum lycopersicum|MIKC_MADS|MIKC_MADS family protein

ATGGGAAGAGGAAGAGTTGAGCTTAAGAGAATAGAAAATAAAATAAATAGGCAAGTCACTTTTGCTAAGAGAAGAAATGGACTTCTTAAAAAAGCTTATG

AACTTTCTGTTCTTTGTGATGCTGAAGTTGCCCTTATAATCTTCTCTAATAGGGGTAAACTCTATGAATTTTGCAGCACTTCAAGCATGGTGAAAACAAT

TGAAAAGTACCAACGTTGCAGCTATGCTACTTTGGAAGCCAACCAATCAGTTACTGATACTCAGAATAACTACCACGAATATCTGAGGCTAAAAGCTAGA

GTTGAGCTCCTCCAACGATCTCAGAGAAACTTTCTTGGTGAAGATTTGGGCACGTTAAGCTCGAAGGACCTTGAGCAGCTTGAGAATCAATTAGAGTCTT

CCTTAAAGCAAATCAGGTCAAGGAAGACACAATTCATGCTGGATCAGCTTGCAGATCTTCAACAAAAGGAGCAAATGCTTGCAGAATCTAATAGATTACT

CCGTAGAAAGTTAGAAGAAAGTGTAGCTGGATTTCCACTTCGATTGTGTTGGGAAGATGGAGGTGATCATCAACTTATGCATCAACAAAATCGTCTCCCT

AACACAGAGGGTTTCTTTCAGCCTCTTGGATTGCATTCTTCTTCTCCACATTTTGGGTACAATCCTGTTAATACAGATGAGGTGAATGCAGCGGCAACTG

CACACAATATGAATGGATTTATTCATGGATGGATGCTTTAA

>Solyc04g005320.2.1 Solanum lycopersicum|MIKC_MADS|MIKC_MADS family protein

ATGGGAAGAGGTAAGGTAGAATTGAAGAGAATAGAAAATAAGATAAACAGGCAAGTTACTTTTGCTAAGAGAAGAAATGGATTACTCAAAAAAGCTTATG

AGCTTTCTATTTTGTGTGAAGCTGAAGTTGCTCTTATCATTTTCTCTAATAGAGGCAAACTCTATGAATTTTGCAGTACCTCTAGTATGTCTGATACACT

GGAGAGATACCATAGATGCAGCTATGGTGACCTTGAAACTGGCCAGTCTTCAAAGGATTCACAGAATAACTACCAAGAGTATATGAAGCTGAAAGCAAGA

GTTGAAGTGCTACAACAGTCACAAAGGCATATACTTGGAGAGGACTTAGGACAATTAAACACAAAAGATTTGGAACAGCTTGAGCGTCAACTGGATTCAT

CTTTGAGGCTAATAAGATCAAGAAGGACACAAAACATGCTTGATCAACTTTCTGATCTTCAACAAAAGGAACAATCTCTTCTTGAAATCAACAGATCCTT

GAAAACAAAGTTGGAAGAAAACTCTGTAGCACATTGGCATATCACTGGAGAGCAAAATGTACAATTCAGACAACAACCTGCTCAGTCAGAGGGGTTCTTT

CAGCCTTTACAATGCAATACTAATATAGTGCCAAACAGGTACAATGTGGCTCCATTGGATAGTATAGAACCATCAACACAGAATGCTACTGGAATTTTAC

CAGGATGGATGCTTTGA

>Solyc04g078300.2.1 Solanum lycopersicum|MIKC_MADS|MIKC_MADS family protein

ATGGGGAGAGTAAAGCTTCAAATCAAGAAAATAGAGAATACAACAAATAGGCAAGTCACTTTCTCCAAAAGAAGAAATGGTCTTATCAAGAAAGCTTATG

AACTTTCTGTACTTTGTGATGTTGATGTTGCTCTCATCATGTTCTCTCCCTCTGGTCGAGTTAGTACTTTCTCTGGAAATAAAAGCATTGAAGATATTAT

GGCACGTTATGTGAATCTTCCGGAGCATGATCGAGGAAGGCTACATAACCAAGAGCATCTCCAAAGGGCTATTGCTAAGCTAAAATGTGAAGCAGATCGA

ACTTATCAAGCACCAAGTAGCCCATCAAGTGTTGATTCCCACATAGAGGAATTTCAACAAGAAATTATTAGATACAAAACTCAAGTAGAAGACATGGAAA

GGCGACTAAGAATGTATGAAGGTGGTTTTTGTGAAATTACCACAGTATGTGAGGCACAATATAGGGAAGAAATACTCCAAGAGACACTAAAACAAGTTCA

AGCTCGCAAACAAGTATTGGAGGAAAATTATCACTCTCCACAAACACAAAACACTACACAGCCACAGATGGATTTTTCAGGACAAAATGTGAACATGGTG

AATAATGTGGCAACAAGTGATGCAATTGCAAATAGTACATTTATGGATTGGGTTCCACACTCACAAAGAGATCCTCATGTCCAAATTCTCAATTTTCTTG

ATTCCAGTGGCCTTCTTCCCTTCAGAGATGAAGCTGATCAACACATGTTACCACCATCTTTAAATCAACTTCACGGTGTGAATGTCCCTGCTGGAACAGA

TCATTTGAGTTCAAATAGTCGATTTGATCAAAATAATCCGCCTCGTCCATCATCGTTCGATGGCATAATCGATGTCAATAACGCTCCGTGGCCACCGTTA

TATACAACAGGGGATGATCCATTTCCAGTGTCACAACCTAGAGAAAGAGCAATTCTTGAACTCTTCTTGTCTCAACTCACTCCAGTGAACCAAGATCACA

TATGA

>Solyc04g081000.2.1 Solanum lycopersicum|MIKC_MADS|MIKC_MADS family protein

ATGGCTCGTGGTAAGATCCAGATCAAGAAAATAGAAAACCAAACAAATAGACAAGTGACTTATTCAAAGAGAAGAAATGGGCTATTCAAGAAGGCTAATG

AACTTACTGTTCTTTGTGATGCTAAAGTTTCAATTGTTATGATTTCTAGTACTGGAAAACTTCATGAGTTTATAAGTCCCTCTATCACGACCAAACAATT

GTTCGATCTGTACCAGAAGACTATTGGAGTTGATATTTGGACTACTCACTATGAGAAAATGCAAGAGCAGCTAAGGAAGCTAAAGGATGTGAATAGGAAT

CTACGAAAAGAGATCAGACAGAGGATGGGAGAAAGCCTAAATGATCTGAACTATGAACAGTTGGAAGAACTCATGGAAAATGTGGACAATTCTCTGAAGC

TTATTCGTGAAAGAAAGTTTAAGGTGATTGGCAATCAGATTGAAACTTACAGGAAAAAGGTTAGGAATGTGGAAGAAATAAATAGAAATCTCCTACTTGA

ATTTGATGCAAGACAAGAGGATCCATATGGTGGATTAGTTGAGCATGATGGAGACTACAATTCCGTGCTTGGATTTCCAACTGGAGGGCCTCGTATATTA

GACTTACGCCTTCAACCCAACAACAATTATCATAATCATCTTCACAGTGGAGGTGGCTCTGATATTACTACTTTTGCTCTAGGTTGA

>Solyc05g012020.2.1 Solanum lycopersicum|MIKC_MADS|MIKC_MADS family protein

ATGGGTAGAGGGAAAGTAGAATTGAAGAGAATTGAGAACAAAATAAATAGACAAGTTACCTTTGCAAAGAGAAGAAATGGACTCCTAAAGAAAGCTTATG

AACTTTCTATACTTTGTGATGCTGAAATTGCTCTTATTATTTTCTCTAGTCGTGGCAAGCTTTATGAATTTTGCAGCAATTCAAGTATGTCCAAGACATT

GGAGAGATACCACAGATACAATTATGGTACACTTGAAGGAACCCAAACTTCATCAGATTCACAGAACAACTACCAAGAGTATTTGAAGCTTAAAACAAGA

GTGGAAATGTTACAACAGTCTCAAAGGCATTTGCTAGGTGAGGATTTGGGACAATTGGGCACAAAAGACTTGGAACAGCTTGAACGTCAATTGGATTCAT

CATTGAGGCAAATTAGGTCAACAAAGACACAACACATTCTTGATCAACTTGCTGAACTTCAACAAAAGGAACAATCTCTTACTGAAATGAACAAATCTTT

GAGAATAAAGTTGGAAGAACTTGGTGTTACCTTTCAAACATCATGGCATTGTGGTGAGCAAAGTGTACAATATAGACATGAACAGCCTTCTCATCATGAG

GGATTTTTTCAACATGTAAATTGCAATAATACATTGCCTATAAGTTACGGATACGATAATGTACAACCCGAAAATGCAGCACCATCAACACATGATGCTA

CTGGAGTTGTACCTGGATGGATGCTTTGA

>Solyc05g015750.2.1 Solanum lycopersicum|MIKC_MADS|MIKC_MADS family protein

ATGGGAAGGGGTAGGGTTGAGCTAAAGAGAATAGAGAACAAGATCAACAGGCAAGTGACCTTTGCTAAGAGAAGAAATGGGCTTTTGAAGAAAGCTTATG

AGCTTTCAGTTCTTTGTGATGCTGAGGTTGCTCTAATCATCTTCTCTAATAGGGGAAAACTCTATGAGTTCTGCAGTAGTTCTAGCATGCTAAAGACGTT

GGAGAGGTACCAGAAGTGCAACTATGGAGCACCTGAACCGAATATATCAACACGAGAAGCACTGGAAATTAGTAGCCAGCAGGAGTACTTGAAGCTTAAA

GGACGTTACGAAGCATTGCAGCGATCACAGAGGAATCTTCTTGGTGAAGATCTTGGTCCTTTGAACAGCAAAGAACTTGAATCACTTGAGAGACAACTTG

ATATGTCGCTCAAACAGATCAGATCAACTCGGACTCAGTTAATGTTGGATCAACTTACAGATTATCAGAGAAAGGAACATGCATTGAACGAAGCCAACAG

AACCTTGAAACAAAGGTTGATGGAAGGAAGCCAACTAAATCTGCAGTGGCAGCCAAATGCACAAGATGTGGGCTATGGCCGGCAAACAACTCAAACTCAG

GGCGATGGCTTCTTTCATCCTTTGGATTGTGAACCTACTTTGCAAATTGGGTATCAGAATGATCCAATAACAGTAGGAGGAGCAGGGCCTAGTGTGAATA

ACTACATGGCTGGCTGGTTGCCTTGA

>Solyc05g056620.1.1 Solanum lycopersicum|MIKC_MADS|MIKC_MADS family protein

ATGGGAAGAGGAAAAGTTGAATTAAGAAAAATAGAGAATAAAATAAATAGACAAGTAACATTTTCAAAGAGAAGAGGTGGATTAGTGAAAAAAGCTCATG

AAATTTCAGTTTTATGTGATGCTGAAGTTGCTTTAATTGTTTTCTCTCAAAAGGGAAAAATCTTTGAGTATTCTTCTGATTCATGTATGGAACAAATTCT

TGAACGATATGAAAGATACTCATATGCAGAGAGACGTTTGCTTGCAAATAATTCTGAATCACCGGTGCAGGAAAACTGGAGCTTGGAATATACTAAACTC

AAGGCTAGGATTGATCTCCTTCAAAGGAACCACAAGCATTATATGGGAGAAGATCTTGATTCAATGAGCTTGAAGGACTTGCAAAACTTGGAACAACAGC

TTGATTCTGCTCTTAAGCTAATTCGATCGAGAAAGAACCAACTCATGCATGAATCAATCTCTGAACTGCAGAAAAAGGAAAGAGCTATCCTAGAGGAGAA

TAACATGCTAACCAAGAAGATTAAGGAGAAGGATAAGATAGTAGAACAGCAAGGTGAATGGCACCAGCAAACTAATCAAGTTTCTACTTCAACATCTTTC

CTCTTACAACCACATCAATGCCTAAATATGGGAGGTAATTACCAAGATGAAGTAGCAGAAGCAAGGAGGAATAATGAGCTTGACCTAAATCTTGATTCAT

TATATCCACTTTACAACATGAATAAACATCTATGA

>Solyc06g059970.2.1 Solanum lycopersicum|MIKC_MADS|MIKC_MADS family protein

ATGGGGAGAGGTAAAATAGAGATAAAGAGAATAGAAAACACAAACAACAGGCAAGTAACTTATTCAAAAAGAAGAAATGGTATAATAAAGAAAGCTAAAG

AAATTACTGTTCTTTGTGAAGCTAAGGTTTCACTTATAATCTTTGCTAGTTCTGGAAAGATGCATGAATATTGTAGCCCTTCTACTACGATAAGTGATAT

GTTGGATGGTTATCAAAAAGCTTCTGGGAGGAGACTATGGGATGCTAAGCATGAGAATTTGAGTAATGAAATTGATAGAATCAAGAAAGAGAATGACAGT

ATGCAGGTTAAGCTCAGGCACCTCAAAGGAGAAGATATCAATCAACTTACCCATAAAGAGCTTATAATTATGGAAGAAGCCTTACAAAATGGACTTTCTA

GTATCAGTGCCAAGCAGTCTGAAATCTTGAGGATGGTCAGGAAAAATGATCAAATTCTGGAGGAGGAAAATAAGCAACTTCAATATGCTTTGCACCAAAA

GGAGATGGGAGCCATTGGTGGAAGTGGAAATATGAGAGGAATTCATGAAGAAGTGTATCATCAAAGAGAAAGGGATTATGAGTACCAAATGCCATTTGGC

CTACGAGTTCAGCCAATGCAGCCAAATCTACATGAAAGAATGTAA

>Solyc06g069430.2.1 Solanum lycopersicum|MIKC_MADS|MIKC_MADS family protein

ATGGGAAGAGGAAGAGTCCAGTTGAAGCGAATAGAGAACAAAATTAACCGTCAAGTTACCTTCTCGAAACGTCGATCTGGTTTGCTGAAGAAAGCCCATG

AGATCTCTGTGCTTTGTGATGCTGAGGTTGGTTTGATTGTTTTTTCTACTAAAGGAAAACTCTTTGAATATGCCAACGATTCCTGCATGGAGAGGATACT

TGAAAGATATGAAAGATACTCATTTGCTGAGAAACAGCTTGTTCCTACTGATCATACCTCCCCGGTAAGCTGGACCCTTGAACATGCAAAACTTAAGGCC

AGACTTGAGGTTCTGCAGAGGAACCAAAAGCATTATGTGGGAGAAGATTTGGAGTCCTTAAGTATGAAGGAACTTCAGAATCTGGAGCACCAGCTTGATT

CAGCTCTTAAACACATTCGATCAAGAAAGAATCAATTGATGCATGAGTCCATTTCTGTGCTTCAAAAAAAGGACAGAGCATTGCAGGAGCAAAACAACCA

GCTTTCGAAGAAGGTGAAGGAGAGGGAGAAAGAGGTGGCACAGCAAAATCAGTGGGAAATCAACTCATCTTCATTTGTTTTGCCACAACAACTGGACTCT

CCTCACCTTGGGGAAGCATACCAGAGTACTAATGTAATAGATAATGGGGAAGTGGAAGGAGGTAGTTCTTCACAGCAGCAAGGTGCAGCTAATAATACTG

TGATGCCACAATGGATGCTTCGTCATCTTAATAATTAA

>Solyc07g055920.2.1 Solanum lycopersicum|MIKC_MADS|MIKC_MADS family protein

ATGGTTTTTCCTATTAATCAGGAATTACTTGTCGATGAGTCGTCTTCTCAGTTGAGAAAAACAAGTGGAGGAACTGGTGGAGGAGGTAGAGGGAAGATTG

AAATTAAAAGGATCGAAAATACGACAAATCGACAAGTTACGTTCTGCAAGCGTAGAAATGGGCTATTGAAAAAAGCTTATGAACTTTCTGTTCTTTGTGA

TGCTGAAGTTTCACTAATTGTATTTTCCAGCCGCGGCCGTCTCTATGAATATGCCAATAACAGTGTTAGGGCAACTATTGATAGGTACAAGAAACACCAT

GCTGATTCCACTAGTACTGGATCTGTTTCTGAAGCTAACACTCAGTACTACCAGCAAGAAGCATCCAAACTGCGACGACAAATTCGAGATATACAGACTT

ATAACAGGCAAATAGTTGGAGAGGCATTGGGCAGTTTAAGCCCTAGAGACCTCAAGAATTTGGAAGGGAAACTTGAAAAGGCCATTGGTAGAGTCCGTTC

CAAAAAGAATGAATTGCTTTTCTCAGAAATAGAGCTCATGCAAAAGAGGGAGATTGAGCTGCAGAACGCCAACATGTATCTACGCATAGCAGAGGTAGAG

AGAGCACAAGAGCAAATGAACTTGATGCCTGGAGGCGGAGGCGGAGGCGGAGGCGGAGGAGGAGGAGGATCTGATCATCAATACCATCATCAGCCAAATT

ACGAAGATGCTCGCAATAACTTCCTGCCTGTAAATCTCCTGGAACCAAATCCTCATTACTCTCGTCGCGACAATGGTGACCAAACTCCTCTCCAGCTTGT

CTGA

>Solyc08g067230.2.1 Solanum lycopersicum|MIKC_MADS|MIKC_MADS family protein

ATGGGAAGAGGAAAGATAGAGATAAAGAGAATAGAAAACTCAAGCAATAGACAAGTAACATACTCAAAGAGAAGAAATGGGATCTTGAAAAAAGCTAAGG

AAATTAGTGTTCTTTGTGATGCTCATGTTTCTGTTATCATTTTTGCTACTTCTGGAAAAATGCATGAATTCTCGTCTACTTCTTTGGTTGATATTTTGGA

TCAATACCACAAGCTTACTGGAAGAAGATTGTGGGATGCTAAGCATGAGAACTTGGACAATGAAATCAACAAAGTCAAGAAAGACAATGACAACATGCAA

ATAGAACTCAGGCACCTAAAGGGTGAAGATATATCATCTTTGAATTATAGAGAACTCATGATATTGGAAGATGCACTTGAAAATGGACTCACTGGTATCC

GTGAAAAACAGAATGAGTTTATGAGGATGATGAGGAAAAAGACTCAAAATATGGAGCAGGAGCAAGATCAACTTAACTGTCAATTGAGACAACTAGAGAT

AGCAAGCATGAATAGGAACATGGGAGAAATAGGGGAAGTGTTTGAGCAGACAAGGGAGAATCATGATTATGGGCAAATGCCTTTTGCTTTCAGAGTCCAA

CCAATGCAGCCTAATTTGCACCAAAGGTTCTAA

>Solyc08g080100.2.1 Solanum lycopersicum|MIKC_MADS|MIKC_MADS family protein

ATGGTGAGAGGAAAAACTGAGTTGAAAAGAATTGAAAATGCAACAAGTAGACAAGTGACCTTCTCAAAAAGAAGAAGTGGGCTTCTAAAAAAAGCATTTG

AGCTTTCAGTTTTATGTGATGCTGAAGTTGCTCTTATTGTTTTCTCTCCAAAAGGAAAGCTTTATGAGTTCTCAAGTTCCAGTACAAACAAGACAATAGA

ACGCTATCAGAAGAATGAGAAGAGCCTGGGACGACTCAACAGAAAATTAACTGATCAACTAACCACTGAGCATTTGAAAGAGGAAGTTGCAACCATGACT

AGAAAGCTTGAATTTCTTGAAGACTCTAAGAGAAAACTTTTAGGACATGGTCTAGAATCTTCCACCTTTGATGAACTTCAAAAGGTAGAAGAACAGTTGG

AAAAAAGTTTAAGCAACATTAGGGCAAGAAAGAATCTGTTATTCAAGGAACAGATTGCTCAACTGAAGGAAGAGGAAAAAATTCTATTGAAGGAAAATGT

AGACTTGAAAAAAAAGTGTCAGGTGCTACCATTGACTTTAACACCAGTTCCTCTAGTAGAGAAAGATGTTGAAAGACAAATAATGGAAGTTGAGACAGAG

CTCTTTATAGGACTTCCAGAGACTAGAAAAAGTTCATATTGTCCTAATTTAAACACACTACCTACATTACTTTAA

>Solyc10g080030.1.1 Solanum lycopersicum|MIKC_MADS|MIKC_MADS family protein

ATGGGAAGAGGGAAAATAGAGATGAAGAAAATTGAAAATATAAGTAGTAGACAAGTAACATTTTCAAAACGAAGAGCTGGACTTTTTAAGAAAGCTGAAG

AATTGTCTGTTCTTTGTGATGCTGAAATTGGTGTTATTGTTTTCTCTAATACTGATAGGCTTTATAAATTTGCTAGCTCCAAATCCAGTATGGAAAAAAT

TGTGGAGAGATATAACAGTTCTTCACATTCATTTGAGCATCCTATGATCGAAAATGTGGTAGAGCCTGAATTGAATTCCTTGAAAGCTGAAGTTGCAAAA

TTACGAAAGGCTACCGGAAGGATGATGGGGAAAGAACTTGATGGCCTGGACTTCAAAGAGTTGCAGCAATTAGAGCATCAACTAACTGAAGGCATTTTAT

CTGTTAAGAATAAGAAGGAACAAGTACTATTGGAGCTACTTGAAAAATCAAATCTGCAGATTGAGGAGCTTGGCCACAAATCATGTAATCATTATCCTGA

AAATTATGAAGCAGCAAGGAAAATTTCTGGTGGAAATACAACAGTAATTTGTGATTTTAAATCAGTTGAAGAAGAAAATTCAGACACTTCTTTGAGCTTA

GGACTATCAGTTGCTACAAGTCAGAAGAAAAAAAATCCACAGATTGAATGCACTTCAAATGATTCTGAGAATCTAATGATTTTAGATTAA

>Solyc11g005120.1.1 Solanum lycopersicum|MIKC_MADS|MIKC_MADS family protein

ATGGGGAGAGGAAAGATAGAAGTGAAGAGAATTGAGAACAAAACAAGTAGACAAGTTACTTTCTCAAAGAGAAGAGCTGGACTTTTGAAGAAAACACATG

AACTTTCTGTTCTTTGTGATGCTCAAATTGGACTCATCATTTTCTCAACCAAAGGCAAATTGTTTGAGTACACCACTCAACCTCACAGCATGGGTGAAAT

CATTAATAAGTATCTCCAAACTACTGGTGCCTCACTTCCAATTCATGATCATAGGGTGGAACAATATGATGAAATAACAAAAATGAAAAGAGAAACATTG

AATCTTGAATTAAGTCTTCAAAGATACAAAGGTGATGAATTGAACTCAGCACAATATGATGAATTAAATGAACTTGAGAAGCAGCTTGAAAATTCTATTA

ACAAAATTAGAGCCAGAAAGCTTGAACTCTTGCAACAGCAGATGGAAAACCTGAAGAGAACAGAGAAAATGTTGGAGAAAGAAAATCATGATATGTGTCA

GTGGTTGATGAAGTATGAAATGTACAAGCAACAGCCAGTAGCAATGATGGAGCAACAAGAAGAAGCAGCAATTACTGAACTGAATTTACTTGGAGAACAA

CCATTGTTGTCTCAATTTTCATTCTTTGGAGATCAACACCAACTTGGTACTACAAGTAATTCTTCAGCATATCACCTTCAAACTTCTCACCCTTTTACTC

CCTCTACCTACGATTGA

>Solyc11g010570.1.1 Solanum lycopersicum|MIKC_MADS|MIKC_MADS family protein

ATGGCTAGAGAAAAAATTCAGATCAAGAAAATAGATAACTCCACAGCAAGACAAGTTACATTTTCAAAGAGGAGAAGAGGTTTATTCAAGAAAGCTGAAG

AACTTTCTGTTCTCTGTGATGCTGATGTTGCTCTCATCATTTTCTCTTCTACTGGAAAATTATTTGACTATTCTAGCTCAAGCATGAAACAAATTCTTGA

GAGGCGTGATTTGCATTCCAAAAATCTGGAAAAATTGGATCAACCATCACTTGAACTTCAGCTTGTAGAAAATAGCAACTACTCCAGATTAAGCAAGGAA

ATTTCCGAAAAAAGTCATCGATTAAGGCAAATGAGGGGAGAAGAACTTCAAGGACTAAATATTGAAGAGTTGCAACAATTGGAGAGATCTCTTGAAACTG

GATTGAGCCGCGTCATAGAGAGAAAGGGTGATAAAATAATGAGAGAGATCAACCAACTCCAACAAAAGGGTATGCATCTAATGGAAGAAAATGAAAAATT

AAGGCAACAGGTGATGGAGATATCTAATAATAATAATAATAATAATAATGGATATAGAGAGGCAGGAGTAGTAATATTTGAACCAGAAAATGGATTTAAT

AATAATAATAATGAAGATGGCCAATCATCTGAATCAGTAACAAATCCATGTAACTCAATTGATCCTCCTCCTCAAGATGATGATAGTTCTGATACTTCTC

TCAAATTGGGGTTAGCTACCTTACTCAGGCTGAAGAGATCAAAAGCAAGGTGTGGCTATTTTTGTATGTTATTAGAAGAAGGAGAAAAAAAAAAGTAA

>Solyc11g028020.1.1 Solanum lycopersicum|MIKC_MADS|MIKC_MADS family protein

ATGGGTCGAGGAAAGATAGAGATAAAGAGGATTGAAAACAACACAAATCGACAGGTAACATTCTGCAAGAGAAGAAATGGATTACTGAAAAAGGCATATG

AACTTTCAGTTCTATGTGATGCTGAGATTGCTCTCATTGTTTTCTCTACTCGTGGTCGACTCTATGAATACTCTAACAACAATGTAAAGGCAACTATAGA

ACGATACAAAAAGGCAACAGCAGAAACGTCTAGTGCGTACACTACTCAAGAGCTCAATGCACAGTTTTACCAACAAGAATCAAAGAAGTTGCGGCAACAG

ATACAAATGATGCAGAACACAAATAGGCATCTGGTGGGTGAAGGGCTGAGCTCTTTGAATGTGAGGGAACTGAAGCAATTGGAGAACAGACTTGAACGAG

GCATTACAAGAATTAGGTCCAAAAAGCATGAAGCAATACTAGCTGAGACTGAGGATTTGCACAAGAGGGAAATTCAACTTGAACAGGAAAATGCATTCCT

TAGATCAAAGATAGCAGAAAATGAGAGGTTGCAGGAACTAAGCATGATGCCATCTGGTGGGGAAGAGTATAATGCATTTCAACAATATTTAGCAAGAAAT

ATGCTACAGCTCAACATGATGGAAACTGCACTACCATCTTATGATCCATTGTCTCCTGATCACAAAAGGTAA

>Solyc11g032100.1.1 Solanum lycopersicum|MIKC_MADS|MIKC_MADS family protein

ATGGCTCGTGGTAAGGTTCAAATGAAGAGGATAGAGAATCCAGTTCATCGACAAGTCACTTTCTGCAAACGTCGAGCAGGCCTTCTTAAGAAGGCCAAAG

AGCTCTCCGTTTTGTGCGATGCTGAAATTGGTCTTTTCATTTTCTCCGCTCACGGAAAGCTCTATGAACTTGCTACTAAAGGAAGCATGCAAGGGCTGAT

TGAGAGGTACATCAAGTCAACCAAGGGAGTTGAGGTGGCTGAGGAAGCCAAAGATACACAACCTCTGGACCCAAAAGAGGAGATCAACATGCTGAAGAAT

GAGATTGACGTACTCCAGAAAGGCTTAAGCTACATGTATGGGGGAGGCGCAGGAACAATGACACTAGATGAACTTCATTCACTTGAAAAGTACCTTGAAA

TTTGGATGTATCATATTCGTTCAGCAAAGATGGATATCATGTTTCAAGAGATCCAACTGTTGAAGAATAAGGAAGGGATACTGGAAGCTGCAAACAAATA

TTTACAGGATAAGATAGATGAGCAATACACTGTGACTAACATGACCCAGAATTTGACTGACTTTCAATGCCCACTAACTGTACAAAATGAGATATTTCAG

TTTTAA

>Solyc12g038510.1.1 Solanum lycopersicum|MIKC_MADS|MIKC_MADS family protein

ATGGGAAGAGGAAGAGTAGAACTAAAGAGAATAGAGAACAAAATAAACAGGCAAGTTACTTTTGCTAAGAGAAGAAATGGACTTCTTAAGAAAGCTTATG

AGTTATCTATACTTTGTGATGCTGAAGTTGCTCTCATCATCTTCTCTAGCCGCGGAAAACTCTATGAGTTTTCAAGTGCTTCCAGCATGATGACAACACT

TGAAAAGTATCAACAATGCAGTTACGCATCTTTGGACCCGATGTTACCGGTTAGTGATACTCAGATGAACTACAATGAGTATGTGAGGCTAAAAGCTAGA

GTTGAGCTCCTTCAACGTTCTCAAAGACATATTCTTGGAGAGGATTTGGGCACACTAAACTCGAAAGAACTTGAGCAGCTTGAGCACCAATTGGATGCAT

CTTTGAAGAAAGTTAGATCAAAAAAGACTCAATCTATGCTGGATCAGCTGGCAGACCTTCAAGAAAAGGAGCAAATGCTGGAAGAAGCAAATAAACAACT

AAAAAACAAGCTGGAAGAAAGTGCAGCTAGAATTCCACTTGGATTGTCATGGGGAAATAATGGAGGACAAACAATGGAATACAATCGACTCCCTCCACAA

ACTACTGCACAACCTTTCTTTCAACCTCTCCGTTTGAATTCTTCATCGCCTCAATTCGGATACAATCCAAATATGGGTGCAAATGATCATGAGGTTAATG

CAGCAACAACTGCTCATAATATTAATGGATTTATTCCAGGGTGGATGCTCTAA

>Solyc12g056460.1.1 Solanum lycopersicum|MIKC_MADS|MIKC_MADS family protein

ATGGTGAGGGGAATAACTGAGATGAAAAGGATTGAAAATACAACAAGTAGACAAGTGACATTCTCAAAAAGAAGAGGTGGACTACTTAAGAAGGCATTTG

AACTCTCAGTTCTTTGTGATGCTGAAGTTAGTCTCATCATATTTTCTCAAAAAGGAAAACTTTTTGAGTTTTCAAGTTCAAGTACTAACAAGACAATAGA

ACGTTATCAAAAGAATGACAAGAATTTGGGTCATGAAAATATATTACTTGAACAAACTACAGAGCATTTGAAGGGGGAGGTTATGAGCATGACTAGGAAT

CTGGAGGTTCTTGAAATCTCTAAAAGAAGGCTTTTAGGAGAAGATTTAGAGTCTTGTTCCATTGATGAACTTGAAAAGGTTGAAGGACAATTAGACCAAA

GTTTAAGAAATATTAGGGCAAAAAAGAACCAGCTATTCAAGGAACAAATTTCTCTTCTTAAAGACGAGGAAAAAGTCCTAATGAATAAAAATGCAGAATT

GCGGGAAAAGTATGAGGCTCGATCACTACCTTTATTTATTGATCGACGAGAAGATGAAAGTCCTCAAACACAAAATATGGAGGTAGATACACAACTATTT

ATTGGACTTCCTGAAAGATGA

>Solyc12g087830.1.1 Solanum lycopersicum|MIKC_MADS|MIKC_MADS family protein

ATGGGGCGAAGGAAGGTAGAAATTAAGCGAATTCAAGATAAAAATTGCAGGCAAGTTGCGTTCTGTAAACGGAGGAAGGGTTTATTGAAGAAAGCTAAAG

AAATTTCTATTCTCTGCGATGTCGATGTTGCTGTTGTTATCATCTCAAATCGTGGTAGACTCCATGAATTCTCCAGCAATAACAGTATGACAGCGATGCT

TCGACGATATGAAAGCCATGTTGGAGCAGAAAAAGAGATCAATGCAGAAATCCAGGTCGCAGAGGTGTCGGGGTTCACAACAATGGGAGAACTGCTACAA

ACAACAGAAAGGCAACTCGAGGAAACAAATGCTGATGGTCTCACTTTGACTGACCTTATCCATTTGGAAAACGAACTTCAAACTGCTCTAATACATCTCA

GAGCTAGAAAGACACATTTGATGCTTGAATCTGCTAAGGTTCTTCATGAGAAGGAAAAACTGCTGCTAGAGGAAAAGAAACATCTGGAGGACAATATAGC

TAGTATCAAGAAAAACACAAAAGTGAATGAAATGTCTGACCTTCCAGCACCCCACATGATTTGTGGACAACAAAAAGTTACCCTGAATTTCTTCTAG

>Solyc00g179240.1.1 Solanum lycopersicum|M-type_MADS|M-type_MADS family protein

ATGAGGCGTATAGAGAACGCCACAAGCAGACAAGTTACTTTCTCAAAGAGAAGAAATGGTTTGCTAAAAAAAGCTTTTGAGCTTTCTGTCTTATGTGATG

CTGAAGTGGGATTGATTATATTTTCTCCAAGAGGAAAACTATATGAATTTGCTAGTTCAAGGNNN

>Solyc01g010300.1.1 Solanum lycopersicum|M-type_MADS|M-type_MADS family protein

ATGGCACAAACTAAAATTCTACGCCGGCAAAAGATTAAACGGAAAATGATAACATCTAAAGTGAAACGTATGGTACTTTTCAAAAAGCTATGGGCAAATT

TTGTCAAGGAAGCACATGATCTTTATATGACAACGGGAGCACATGTTACTATTGTCGCTTTTTCCCCGACTGGCAAGGCATATGCTTATGATTCCTCCAA

CAATTTTGATACTATAGAGAGGTTCCTTAATGATTCAAAAGCTTCTGCAATTGAAGGCGGTCATTGA

>Solyc01g060300.1.1 Solanum lycopersicum|M-type_MADS|M-type_MADS family protein

ATGAGAAAACCTAATGGCCGCAAAAAAATTGAAATTGCGAAGATTCAAAATCAAACCAACTTGCAAGTAACATTATCAAAAAGACGTGCCGGCCTATTTA

AAAAGGCAAGTGAGCTCTCGACTTTGTGTGGTGCTAATGTTGCTATTGTAGCTTTTTCTCCTAGCAACAAAGTATACGCATGTGGACACCCTTCCGTCGA

GTCAATTGTGGATAAATTTATCGGAGAGAATCCTCCACCTGAAACTGATGATCCTAACCCCATCATTGTAATGACTGCTGACACAATTCCTAAGGGATCT

CCATTTACGAGCATGCAAGAGTTTTCATTAGGAATAGACTTTGGAAAACTGTGTCAGATTACTTGTGTTCCACAGTTGATTGAGAGTTGCCCCAATTCGA

GTATACTTCAGATTTGGACCGATTACCATCTTGGATCTACAAATGAACTAGCCCATCAGGCGTTATTGGACTTAGGAGGGTACCGACTCGTTATCCTTCC

ACTGCATAGAGCGGGAACTGTTCGTTGCTCTGTGAAACCAGCCTCACGCAGTCCGTTCCACTTTAGGAACCCATCAGCCCATTCCTTCGATTGGATAGCA

AGGCCCAACTCTATGCTTGGACAGGGCAGGCCATAA

>Solyc01g060310.1.1 Solanum lycopersicum|M-type_MADS|M-type_MADS family protein

ATGAGAAAACCTAATGGCCGCAAAAAAATTGAAATTGCAAAGATTCAAAATCAAACCAACTTACAAGTAACATTCTCAAAAAGACGTGCCGGCCTATTTA

AAAAGGCAAGTGAGCTCTCGACTTTGTGTGGTGCTAATGTAGCTATTGTATCTTTTTCTCCTAGCAACAAAGTATACGCATGTGGACACCCTTCCGTCGA

GTCAATTGTGGATAAATTTATCGGAGAGAATACTCCACCTGAAACTGATGATCTTAACCCCATCATTGTAATGGGCTTAAGTTTCCTTGAGACCGATTAC

CATCTTGGACCTACAACTGAACTAGCTCAAGTATGTGTGCCCGCAGCACGACAGATGTCTGGTGCCCGCAGCATAATAGTCGTCTCACAATGTGTGCTTG

AGAGGCTCATTGCAACGTGA

>Solyc01g066500.1.1 Solanum lycopersicum|M-type_MADS|M-type_MADS family protein

ATGACGAAAGGAACAGGGAAGAAGAAGATTGACATTAAAAAGCTCGCCAGTAATTCATCAAGAAAAGTCACATTCTCGAAAAGACGCACTGGTCTTTTCA

AGAAAGCTGAAGAACTCGCAACCAAATTCGGCGCTCGAATTGCTCTGTTAGTCTTCTCGCCGGCCGGACGACTCTACACTTCCGGCGATGTTTCCATCTT

CGAGAATAATGGGTTTCCTTCAAAACCCCATCTTTCTACTGAAACTGCGTCGAATTCATACGTAAAACCAAGTACTCTGCGTCTGAAAAATCTCAATGTT

GAAGGATGTAGTGAAATTTTCTCGCCAGACGGCGTACATTATAGTGCCGGCGAGATTCCCATCGTCGAAGACAACTTGCAGAATAATGGGTTTTCTTCAA

ATTCTCATCTTTCTACTGATTTCAGTTCGAATTTTTATGAAGATTGGAGTACTCTCTGGCTGAAAAATTTCAAGGGAAATAATGAAATTTTCTCGCCGCC

CGGCGGCTTTTACAACTCTGGTGAATTTCCCATTGTTGCGGACGAGTTGAAGAATAATGAGTTTCCTTCAAACTCCGATCTTTCTATTGAAATTTCATCG

AATTCATATGCAAAACCAAGTACTCTGCATCTGAAAAATTTCAATGCTGAAGGATGTAGTGAAATTTTCTCGACAGACGGTAGATTTTACAGTTCCAACG

AGTTTCCCATTATCAACGACACCTTGAAGAATAATGGGTTTTCTTCAAATTCCGATCTTTCTACTGATTTGGGGTCAAATTTGTATGAATATTGGAGTAT

GGAAAATTTTAATGTTGAAGAATGTAGCAGTATTGAAGAACTTATGTTATTGAAGGAGAAATTAGAAGAAAAGAGAGATGAAATTATTTTGAAGATGGAG

GCTGAGTTTATTGATTCATTACTTGTTTAA

>Solyc01g066730.2.1 Solanum lycopersicum|M-type_MADS|M-type_MADS family protein

ATGGGTAAAGGGAAGCAGAAAATTGAAATTAAGAAAATCACTAAAGAATCAGCAAGAAAGGTGGCATTTTCGAAGAGACGCAAAGGACTTTTCAAGAAAG

CTGTGCAACTTGAATCCAAGACTGGTGCTAAAGTTGCCATTCTTGTTTTCTCTTCCTCAGGAAAACCCTACACTTGTGGGGACGTGGAAACGCTTTGTGG

TATTTCCGATTCATTCAATCTTCAAACGCATTCTGAGTCAAATAGTATTTGGGATTCATTCAATCTTCAAACGCATTCTGAGTCGAATGGTATTTGGGAC

TCATTCAATCTTGAAAGGCCTTGTGAGTCGAATGGTATGTGGGATTCATTCAATATAATTGAAGGGCCTTGTTCATCCTCTGGGAAGAATGGTATGTGGG

ATTCATTCAATCTTGAAAGGCCTTGTTCATCCTCTGGGCAGAGTGGTATGTGGGACTCATTCAATCTTGGAAGGCCTTGTGAGTCGAATGGTATGTGGGA

TTCATTCAATATAATTGAAGGGCCTTGTTCATCCTCTGGGCAGAATGGTATGTGGGATTCATTCAATCTTGAAAGGCCTTGTTCATCCTCTGGGCAGAGT

GGTATGTGGGACTCATTCAATCTTGAAAGGCATTGTTCATCCTCAGGAATCCCATCTGGGTCGAATGGTATTTGGGATTCGTTCAATGTTGAAACGCATT

GTTCATCCTCTGAGTCGTGTGGTATGTCTGATTCATTCAATGTTGAAACACATTGTTCATCCTCTGGGCAGAGTGGTACGTGGGATTCATTCAATGTTGA

AGCATGTCATAACGTGAATGAACTCTTGCTGTTGAAGGCACACTTGGAAAGTACAAGAGAAAAGCTGCTGGAGTCTCAGTTTCTTGACTCTCTCTGGTCA

TGA

>Solyc01g097850.1.1 Solanum lycopersicum|M-type_MADS|M-type_MADS family protein

ATGGAGAATAAGAAGAGTAGAGGACGTCAAAAAATACCAATGAAAAAAATAGAAAAACTAGGTGACTTATATGCTAGCTTCTCAAAGCGCCGTTTGAGTT

TGTATAAAAAAGCTAGCGATCTCGTTTTTGAATGTGATGTGGACATTGGAATGATATATTTTTCTCCTAAAGGTAATCCTTACTCATTTTTTCATCCAAA

TGTTAATACCGTTGTTTCTCGTTTTCAGAATCCTGATATGGAATTCAGTGAAAGTGATCTTCTGATCACGACTGATAACCAAGTAAAAGTGAAGGAACTC

AAAAGCAGGCTTGATGAACTTGATATCATAGAAGACATTGCAATTGCTACGAAGAAATCATATGATGAAGTGATTAAAGCTAGAAAGAGAGGTTGGTGGG

AGTCGATTGAGCAGCTTAATGAACCTCAAGTAACCAAGTTTGAAGCTTGGATGGATACTATTATTTTTAACATGCAGAATCGTTTGAATGAAATGGAAAA

TGGAGCTTCGTCCTCATAA

>Solyc01g098050.1.1 Solanum lycopersicum|M-type_MADS|M-type_MADS family protein

ATGGGCACAGGGAAGAAGAAAATAGAGATCGAGAAAATTATAAAGGAAACTTCTAGAATGGTTACATTCTCGAAAAGACGAAAAGGGCTTTTCAAAAAAG

CTAAACAATTTGAATCCATGACGGGTTCTCGTGTTGCTTCAATTGTATTGTCACCCACAGGAAGGCCATATACTTGTGGAGACGTTGATTATGCTATAAG

AACACATTTTTCTAATAGTGGTCGATGTATGAAATTATTAATAACAGACATCATGAATTCTCACGATTCTAATTCTAGTTCCAATGTTGTTGTTTATGGC

GAAACTTCGAGATCGAAATTCTCTTCGGCTCCAAAGAAGAATAGTCTCCACAATTGGGTGAAGCGTATAGATGTTGAACAATGTCAAAATTTGAATTGGC

TCTTAATGTTGAAACAACAATTGGAAGGGACAAAAGAAAAGATTGGTGAAGACGTTGAGTCATTTAAAGCTTTTTTTGTGTAA

>Solyc01g098060.1.1 Solanum lycopersicum|M-type_MADS|M-type_MADS family protein

ATGGGAACAAGGAAGAGGAGGACAGAAATCGAGAAACTCACAAAGCAAAGTGATAGATTGACGACATTTTCAAAAAGAAAGAAAGGAATTTTTAAAAAAG

CTGAACTACTTGAATCCTTGACAAGTTCTCGTGTTACCTCCGTTGTTTTTTCACCTTCTGGCATTCCTTATACTTACGGAAATGTTAACTCTGTTATCAA

AAAGCATTTTCCTAGCTGTAATCGATCAGAAATATCAACAACGGTAATGAATTCTCATCATGATGTTTCTGGCGAATCTTCGGGGTCAAAATCATTATCG

ATCCCAAAGGAGAATGGTCTCCGTCGTTGGGTGGAGGATATAGATGTGGAAGGGTGTCAAAATTTAAATCAACTGTTTATGTTGAAGGAGCAATTAGAAG

GAACCAGAGAGAAAATTATTTCCAGTGATCCTGAGTCATTTGAAGCTTTGTTTATGTAG

>Solyc01g098070.1.1 Solanum lycopersicum|M-type_MADS|M-type_MADS family protein

ATGGGAACAGGAAAGAAGAAAATAGAGATTGAGAAAATCACAAAGCAAACTGCTAGAATGGTGGCATTTTCGAAAAGAAGGAAAGGACTTTTCAGAAAAG

CTGAAGAACTTGAGTCCATGTCAAGTTCTCGTGTTACCTCTGTTGTTATTTCACCTTTTGGCAAGCCTTATACTTATGGAAATGTTAACTCTGTTATAAA

GAAGTATTTTTCCATCTGTATCCGCCCAGAAATATCAACACCAGTGATGAATTCTCATCCTTCCTCTTCTAATGTTTCTGGTGAATCTTTGGGGTCAAAA

TCTTCATCGACCCCAAATGGAAATGCTCTCTGCAATTGGGTGGAAGGTATAGATGTCGAAGAATGTCAAAATCTGAACCAACTGTTAATGTTGAAGAAGC

AATTGGAAGGAACTAGAGAGAAGATTGTTTCCAAAGAATCTGAATCATTTCAAGCTTTGTTTATTTAG

>Solyc01g102260.2.1 Solanum lycopersicum|M-type_MADS|M-type_MADS family protein

ATGACAAGAAAGAAAGTGAAATTAGCCTTCATCACTAATGACTCGGCAAGAAAAGCAACATTCAAGAAAAGGAAGAAGGGTCTGATGAAGAAGGTGAGTG

AATTGAGCACCCTTTGTGGGATTGATGCTTGTGCTATTATTTATAGCCCTTATGACACTTCACCAGAAGTATGGCCAAACACCATGGGAGCTCAACGCGT

GCTCGCCGAGTTTAAAAGGATGCCTGAGATGGAACAGAGCAAGAAAATGGTGAATCAAGAGAGTTTCATAAGGCAGAGGATAGCTAAAGCGAGCGAGCAA

TTGAAGAAACAGAGCAAGGAAAACAGAGAAAAGGAAATGACCGAAGTTATGTATCAAGGGTTGACTGGAAAAGGGCTTCAGAATTTGAATTTGGGAGATT

TGAATGACCTTGGATGGGTAATTGATCAGAATTTGAAAGAGGTTTATAAGAGGATTGAGGCAGTTAAAAAGGGGGCTTCGACTTCCTCTTCTTCTTCTGT

TGCTGCTGCTGCAGTTGCTGCGGCAGCAGTAGCATCGCAGGCAGTTGCTCCACCGATGGAGCAAAAGCCTGCTGTGGTGGAATTGGGATTGGATTCGATG

CAGAGGACACAAACAGAGTGGTTTACTGATTGGATGAACAGCAACGCAAGTGATCAGCATATTGGTTATGGACATGCAGATGAGATGATTTTGCCCAATT

TTAATGATAATCATAATGCTAATGTGTGGCCTAATAATTTCTATCCTTAA

>Solyc01g103550.1.1 Solanum lycopersicum|M-type_MADS|M-type_MADS family protein

ATGGGTCGTGCGAAATTGAAGATGGAATTAATAAGCAAGGAGAAATCAAGGAATGCAACGTTTAAGAAAAGGAAAGAAGGTTTATTGAAAAAGTTGTATG

AATTCACTACACTTTGCAATGTGAATGGCCTTATGATAATGTATGGACCAAAACAAGGGAATGGATCAGAGTGTAGGCCTGAGATTTGGACAAATAGTAG

TGGAAGTAGTAGTAGTACAAATAGTAAAAGCTTACAACAACAACAAGAAGAGATTGAGAATTTAATCGATGAGTATAAAAAGGAGAATAGTTTGCAATCT

GGTAGTAGTAAAACCTTTGGATTGTCTGATTATTTCGTTGATCGAAACAAGAGGGTTGAGGAAGAGTTTATCAAGTTGAGAAAGATGAATATGGAGAAAA

AATATCCATGTTGGCTAGAGTTTATGGATCAGTTATCTGAGTTTAAGTTGAGGGATTTTTTAACTTTGTTGGATGACAGAGTTGAAAATGTCAAGGCTAG

GATTCATTTGCTTAAAGGGAATTTTAGTGGTTTGATGGGAGGAGAAATGATTGATTTGGGAGGAGGAAACCAATGGACTCATTACAATGACAATGTTATG

GTCCAAGGAGGGGGGATGGAATATGGAGATTATAATCAATTGCAAGCTCCAATATATCATCAAGAAATGAGGATGGTGATGATGAATGAGAATGATTGGC

CACAGTATAATAATGGTGCATCGTCGTCGTCCTCCGCGGGGAATGGGAGCAACAATATGATGTGTGCATTGATGAAATATGAAACTATGATGCCAAGTAA

TAATCACTTGGCATACTCACCCTATGTAGCTCCAACAATTCTGCAGCAAACTCCATGCATGATGATGCCACAGCATTCTTGGAGAGACAACGACAGAGAT

GACAAAGCTAAATTTTCACCTTATATGACCAAGTAA

>Solyc01g103870.1.1 Solanum lycopersicum|M-type_MADS|M-type_MADS family protein

ATGGCAGACGAAGACCAAAGCAAGAAGGGAGAAGAAGATCAAAGGAAGAAGAAAACAAACAATTCCAAATCTTATCAAGTGAGGAAAGAATGCATAAAGA

GGAAATCAATGGAGCTTGCAACTCTCTGCGATATCAAGGTTTGTACGGTTATTACTGGTCCTAATAGGGAATTGCAAACTTGGCCTGACAATTTGAATGC

CTGTAAGGAAGTTCTTGATATCTACTCTCAAAATTTGAAACCCGAAAAGAAACACAAGCAAGAAGACAAAGATCTCCCGACGCTAGTTGAATCAAAGCTT

GCAGCAGTCAACAGGAGAATTTGTTTCTTGGAGAACAAGAATGTTGCTGATAAGGGAAAAGGAAAGAGAATTGAATGA

>Solyc01g106170.2.1 Solanum lycopersicum|M-type_MADS|M-type_MADS family protein

ATGATATTTCTAGACAGGTGTCAGCGAGTGAGGCTATTATGGTGTACTCGCGCGAGTAGGGGAAGTAAAATATCTGATTATTTTTTCACTAAACCAATAT

TTCTCCTCTCTCTCTCTCTCTCTATCTGTAGAATCAGTCTACTATCAAAATCTCTCAAATTATCTCGTTTAATTAAGGAGATGGGGAGAGGAAAAATAGT

GATACAAAGGATTGATAATACAACAAGTAGACAAGTTACTTTCTCAAAGAGAAGAAATGGACTTCTCAAAAAGGCTAAAGAGCTTGCAATTCTTTGTGAT

GCTCAAGTTGGACTCATTATTTTCTCAAGCACTGGAAAACTCTATGAATTTGCCAATAACAGCATGAAGTCTACAATTGATAGATACAACAAGATAAAGG

AGGAGAATAATAACATGAATCCCATGTCAGAGGTCAAGGTAACCTGA

>Solyc01g106700.2.1 Solanum lycopersicum|M-type_MADS|M-type_MADS family protein

ATGACCACCATTAACGCCGTCAAGAAAACTCAAGGACGGAGGAAAATTGCAATCAAACCGATAGACAATCAAAACAGTCGGCACGTTACTTTCTCCAAAC

GCCGTTTAGGACTTTTCAAAAAAGCTAGTGAACTTTGCATCTTAAGCGGTGCAGAAATTGCTATACTTGTACAGTCATTAAAACGACAGCGTCTCTTCAC

ATTCGGTCATCCTAGCCCCGACGCCGTCATCGACCGTTACCTCACTGGAAAATCAGTATCTCCCGGTGACGGCGATCAATTCAACTTGCAACAGAGTAAT

CAGTATTATTCCCAGATTTGTAGAGATCTGGAATTAGAGAAGCAGAAGAAGGAGAATATTGAAGAATCGAAGATGGTTAATAATGGGGGATTTTGGTGGA

ACGAACCAATTGATGATATGGGGATTGAAGGACTTGAAGAATTTATGAGTGCTTTGGAAGAATTGAAGAAGAAAGTAACAATGAGAGCTGATGAATTGAG

TATGATAAATGGGTCTTCTTCGAATTCGACCATGAAAATAGCAAGATTTGGGGCTGAAGATCAATATTTTAATGAATCAATCGATTATTGTTCTTCCATT

GTTCCTTTTGATTTCAATCAACCAGGAGATAGACAATTCTGA

>Solyc01g106710.1.1 Solanum lycopersicum|M-type_MADS|M-type_MADS family protein

ATGGCAAAAAAACCTAGTATGGGTCGTCAAAAGATCAAAATTGCCAAGATAGAGGTCAAGAATCACCTCCAAGTTACCTTCTCAAAACGTCGTTCTGGTC

TATTCAAGAAAGCTAGTGAACTATGCACGTTATGTGGTGTTGAAATAGCCATCATAGTTTTTTCTCCAGCGAGAAAAGTATTTTCTTTTGGTCACCCTAA

TGTTGAGTCTATTATCGATAGGTTCCTCACAAGAGCTAATTCTAATAATAATCCAATCGCGAATAATTCAATTCAACTTGTTGAGGCTCATCGAAATGCT

AGTGTTCGTGGGCTCAATTTACAACTTACTCAAATTCTTGGTGAGGTTGAAATTGAGAAGAAAAGAGGAGAATCACTTGATCAAATGAGGAAAACTAGTC

AAAGCCAATATTGGTGGGAAGCTCCTATTAATCAACTTGATTTGCAAGAACTTGAACAATTAAAGGATTCAATGGAGGTTTTGAAAAAAAATGTTACTAA

TCAAGCAAACAAGTTTATGGTTAATGAGACTCCTAATCCTTCTTTTTTTGGTGTTAATGCTAATGGGATTTTTGACAATTATGACATCAAGCCACCAAGA

AACATGAATGCTTCAAATAATCTTCATAACCATAACCTTGGTTTTGATTCATCCACATTTTTCTAA

>Solyc01g106720.1.1 Solanum lycopersicum|M-type_MADS|M-type_MADS family protein

ATGGCAAAAAAAATGAGTAAAGGTAGGCAAAAGATCCAAATGACCAAAATGTCAAAAGAAAGTAACCTTCTTGTTACCTTCTCGAAACGTCGTTCTGGTC

TTTTCAAAAAAGCTAGTGAACTTTGTACCCTTTGTGGTGTTGAAATAGCGATTGTTGTCTTTTCACCAGGTCATAAAGTCTTCTCTTTTGGTCATCCTAA

TGTTGATTCTATCGTAAATCGCTTCCTTACTCGTAACCCTACGAGTTCTTCTTCTACAACTTGTCAACTTGTTGAAGCTCATAGAAACGCAAATGTACGC

GAATTGAACGCACAACTCACGGAGATTCTCAATCAATTAGAGTTTGAGAAAAAACGCGAGGTTGAAATCGAAAAAATTCAAAAGGGTAAAATTGGAAAGA

ATTGGTGGGAAGGTCCATTGAATGAACTTGAACATGGTGAATTGGAACAATTGAAATTGGGAATGGAAGAACTCAAGAAAAATGTTACAAAACAAATGCA

AAAAATAATTTTTGAGGCTTCAAATGCACCTACATTTTTTCTTGGAGGATCATCTTCTTCTAATGGAGATGTCAAAAATATGAAGGGGCTTGGACTTTCT

ATGGCAACCAATGGTGGGCATACAAGTTTTCCATGA

>Solyc01g106730.1.1 Solanum lycopersicum|M-type_MADS|M-type_MADS family protein

ATGGCAAGAAGGGTTAGCAAGGGTCGTCAAAAGGTTGAGATGGTGAAAATGAAGAATGCAAGTAACTTACAAGTTACGTTTTCTAAGCGTCGAGCTGGTT

TATTTAAAAAAGCTAGCGAACTTTGTACGTTATGTGGTGCTGAAATTGCCATAGTGGTATTTTCCCCTGGTGATAAAGTTTTCTCCTTTGGTCATCCCAA

TGTAGAAACGTTGGTGGATAGGTTCCTTGGGAGGGACCTCCCTTTACCAAATAACGATGTCCACAATCATCTAATCGTGGCTCATCGAGAAGCTGGTATT

CGTGAGCTCAATACGAAGCTCATGAATCTCGAGGGGGTTCTCCATATGGAGAAAAAACGCGGAGAATCCCTACAAGAAATTAGGAGGAGAGCTAATGGTC

AATGGTGGGAATCTCCTATAGAAGAACTTAACTTATTCCAACTTCAACACTTGAAGGAGGCCTTGGAAAATCTAGAGCAAAAGGTTGAAAAAGTGGCACA

TCAACAACAAATGCTAAATAATATTGCATTCCCATTCCGCACACTTGGAAGTGCTTTGACTCCTCCTAATTGTGCTAGGGAAACTTCTTCATATGGACTC

AATGTTGGTGCACCCTTTGCTAATAGGGATATTGGATCTACTTCTTCCATAGTTCCCAATCACTAG

>Solyc02g032000.1.1 Solanum lycopersicum|M-type_MADS|M-type_MADS family protein

ATGGAAAATGAGACTAAAAAAGGTAAGAAAAAAATTGAAATGAAGCTTATCGAGTCTGAGAGAGCACGTGCTGTGAGTTTCTCAAAAAGAAAAAAAACTT

TGTTTGAGGATGCAAAGAAGTTTGCAACTCAAACCGGTGCAGATGTTGCTGTGATGCTCTTTTCACCAGGTGGAAAACCATATTCCTGTGGTTCCACAAG

CGTAGAAGATATAATTGAAAATTTTCTCAAAATGAAAGTGGCGGACCCCCGGCGTCATTATGCTGAGGGTGAATCAAAGGTTTTGAGGAATTGA

>Solyc03g007020.1.1 Solanum lycopersicum|M-type_MADS|M-type_MADS family protein

ATGGGATCCAAAATTCGTGGTCTAACCTTATCAAATCACGAGACATTTTCTCAAGTGTTCTTACCAATAAACTTATTTTTTAGAAAAAATATCTTACATC

AAAAGTTGAGTCATGGAAAAAAAAAGACTTTAGGACGTCAAAAGATTTCAATGGTCAAAATAGAAAATGAAGATGCTCGATACACAACATTTTCAAAGCG

TCGATCAACATTATATAAAAAAGCTAGTGAATTGGTTGGAAAATACGATGTTGATGTGGGGATAACATTATTTTCTCCGACTGATAAACCCTACTCTTTT

TTTCACCCTACAGTTGATGTTGTTGTTGATCGTATTCTCAATCCAAATACACAGCCAAGTGAAGATAACAGCATCGCGATTGCAAGTTATCGAAACAAAG

TGAAGGATCAAAAAGTTGAGCTTGACGAGCTTGATATCATAGAAAGAGGTATATCTAATTCGACATTTGGCACTAAGGAAACTAACATGAAAAATATATG

GGAATCCTTTATGAAATTTAATGAAGATGAGGTGAATGAACTCGAGCTTTGGCTGAACTCTATTGATTTTGATTTGAAAAATTATTTATCGCAGCTGGAA

AATAATGTTTCTTCCTCTACACAAGCACCTCCAAAAAATGTTGGTTAG

>Solyc03g034260.1.1 Solanum lycopersicum|M-type_MADS|M-type_MADS family protein

ATGGATAGCAAGAAGACAAGAGTGAGACAAACATTTCCAATTTCAAAAATAGAAAATCAACGTGTTAGCGATGTAACATTTTTCAACCGTCGCTCTAGTC

TATACAGAATGGCAAACGAACTTGTTGATTTCTGTGATGTTGATATTGGAATAGTACTATTTTCACCATCAAACCACCCTTTCTCCTTTTTTCACCCAAC

AAGTGAAGCAGTCATTGAACGTTTTTTGAATCCCGATTCACAACTAAGTGAGAAAACTCGCCTAGATGCGGAGCAAGCACGAAATAAGGTGAATCAACTC

AACAATCGCCTAGATGCTATGGAGAAAAGGATTGAGCAAATAGAAGAAATTGAACATGCTCAAACTCTACTTCAACTTAGCCAAACGGAAGAAAATGGCG

AAAGAAGTAAGTGGAAATCGATTGATCAATTAAATGCAAATGAAATACCAACATTTGAAGCATGGTTAAGAACCACCGTCTCTAAAATGAATTATCGTTT

GGAGAAGTTGGAAAATGAGGCTTCATCATTGAAAAATGCACGTGGAACTTCATAA

>Solyc03g062820.1.1 Solanum lycopersicum|M-type_MADS|M-type_MADS family protein

ATGGGCACAGGAAAGAAGAAAATAGAGATGGAGAAAATTACAAAGAAAAGTTGTAGAATGGTGACATTCTCCAAAAGAAGGAACGGACTTTTCAAGAAAA

ATGAAGAACTTGAATCCTTGACGGGTTCTCAAGTTTCCTCTGTTGTTCTTTCACCCGCTGGAAGGATTTATACTTACGGAGACGTTAACACTGCTATCAA

CATGCATTTTTCCAAAATTGATTGCATGAGACAATCAGATTCTGATGTTGTTGTTTCTAGTGGATCCTCAGAGTTGAGATCATCATCGAAAAGTCTCCGA

GATTGGCTGGAGGATATAGATGTTGAACAGTGTCAAAATTTGAATCAACTTTTATTGTTGAAGGAGCAATTGGAAGGAACTAAAAAAAAGATTGTTTCCA

TTGAAGATTCTAAGTCCTTTCAAGCTTTGTTTATGTAG

>Solyc03g115910.1.1 Solanum lycopersicum|M-type_MADS|M-type_MADS family protein

ATGGCATCTTTTGCATGTGTTTTTTGTCCTCTAGAATTTCACATCAGCGAACCCCTTCCAGCGTTCAAACTTGGGTCTTGCCCGACAAGTAATCCCAAGT

TCCAAAGTGCGCCGATATGTTTTTTCCATAGGATGGGAAGGGTAAGGCTAAGCGTGAAGCGATTGGAGAGTCATAGTAACAGGCAATCCACTTATTGCAA

ACGAAGATGTGGAATCCTGAAGAAAGCCCAGGAGATATCTGTGCTATGTGATATAGACATTATCCTTCTTTTGTTTTCCCCAACTGGAAAACCAACGCTA

TTTCAAGGCGGACAAAGCAATTTTGATGAGATAATTGCAAAATTTGCTCAATTGACTCCTCAAGAAAGGGCAAAAAGGAAGTTGGAGAGCCTTGAAGTAA

GTCTGATATTCATCACCACTATGTGTTCTCCTCTTGCTATGCTTTGTTGTTACTTCATAATCCAAGTGAAAGTAATAACAATGCTAATGGTTACACTCCA

GACATTGAGGAAGACTTTTAAGAAGCTTGACAACGATATAGGCGTGCCAGAGTTTCTAGATGCTAGTGATCCATCAGTTGAAGAATTGCATAGTCAAGTG

AAACTTTTGCAATCTCGGCTTACTGATGTAGAAATGAGACTCAACTGGTGGAGCAATCCTGATAATATAAATAAAGTGGAAGATTTCGCGCTAATGGAGT

GTGCACTAAGGGAGTCACTTAATGCAGTTCATGTTCGGAAGCTCCAGAAGACGACGCATTTGCATCTTTTGATGAACAGTGAACTAGATGGGAATACTCA

TCAGTGGCATCCAGAAAATAAAATCCTGCGCATGCCATTCCCTCAGACTCCAAACATTCTACCCCAGGAAAATATGGGATACTTTGGAGATAATTCAGTT

GCTGAATCTTCTCATGTTCAGGGCTCTGGAGAAGTTGATCAAGCAAGACAGGACACAACTGCAATGCTAGATAACGGTGTCTTGAATGATCTAACTAGTA

TAGCATGCTTGAGACAGCAACTAAGTGAGCAGTACTCATACAACCCCAATGAGGATCTTGATCTGCTTGAAAGGAACATGTTGGATCCTCAGAGTGATGC

CAATTTGAAAGGATATTTGATGGATTATGCATTTCAAAGAAACTTTAACCTAACAAGGTCTGTGGATTCAGTCAATTATAGTGCAGTCGATGCCGTTGCT

GTTCCAGATTTTGATGAAAAATCATATGCGCAGCCAGCAACTTCCAGTGATTAG

>Solyc03g119680.1.1 Solanum lycopersicum|M-type_MADS|M-type_MADS family protein

ATGGAAAATGAGACCAAAAAAGGTAAGCAAAAGATTGAAATGAAGTTGATTGAATCCGAGAAAGCACGCACAGTGAGTTTCTCAAAAAGAAAAAGAACTT

TGTTCCAAGATGTAGATAAGTTTGCTGCTCAGACCGGAGCAGATGTAGGTGTTATGCTCTTTTCACCAAGTGGAAAACCATATTCCCATGGTTCCACAAG

TATAGAAGAAATCATCGATCAATATCTCAAAGTGAAACTAGAGGATCACCAACGTGATCATGCTGAGGGAAAAATGAACGGCTTTGAGGTATTGGGAGCT

CTCCATAAAGAATTACAAGCATGGAACGAGAAAGAAAAAAATCGAAAACTAATGTATAAGATTATGCACTCTGGCTCAGAAGCACCTCCAGATAAACACA

TGGAGGAGCAGAAGTTGGCATTGAAGTTGAGGGTAGAGAAAATTAAAAAAGAAACACAAGCTGCTATTCTGGTTGAGCATCTAAAGTTTGATTTAAATGT

TGCCCCTGAGCCAGAAGAAGATGAGAGTTCTTGA

>Solyc04g025030.1.1 Solanum lycopersicum|M-type_MADS|M-type_MADS family protein

ATGACAACAAGGATTAACAAGGGTCGTCAAAGGGTTGACATGGTGAAAATGAAGAATGCGAGGAATTTACAAGTTACATTTTCTACGCGTCTTGCTGGTC

TGTTCAAAAAGTCTAATGAACTCTGTATGTTGTTTAATGCTGAAATTTTCATTGTGGTATTTTCACAGGGTGATAAAGGAGTTTTGTGCTTTGATCACCC

TAGCGTGAACCCATTGGCAGAAGGGTTCTTTGAGTGGAACCTCCCTCAACCACATATTAATGTCCACAATCAACATATTGTGGCTCGTAAAGAAGGCGGT

ACTCGTGATTTGAGTACCAAGCTCATGAGCCTCGAGGCGATTCTCGAGAAAGAAAAAAATTGCGGACAAATCCTTATAGAAATTAGGAAGAGAGCTAATA

GTCTATGA

>Solyc04g025050.1.1 Solanum lycopersicum|M-type_MADS|M-type_MADS family protein

ATGAAGAATACTAGAAACTTACGAGTTACATTTTCTAAGCATCGTGTTGGTTTATTCAAAAAGGCTAGTAAACTTTGTATGCTATGTGGTGCTGAAATTT

CCATTGTGGTATTTTCCCCGAACGGTAAAGTTTTCTCCTTTGGTCACCTTAGCATGGACACGTTGATATAG

>Solyc04g025110.1.1 Solanum lycopersicum|M-type_MADS|M-type_MADS family protein

ATGGCAACAAGGATCAACAAGGGTTCTCAAAGGGTTGACATGGTGAAAATGAAGAATGCGAGGAACTTACAAGTTACGTTCTCTAAGCGTCTTGCTGGTC

TGTTAAAAAAGGCTCTTGAACTTTGTATGTTGTGTGGTGCTAAAATTATCATTGTGGCATTTTCACCGAGCGATAATGGAGATTTCTCCTTTGGTCCCAC

TAGCATAAGCCCATCGGTGGAGAGGTTCCTTGGGAGGAAATTTCCACAACCAAATAATGATGTTCACAATCAACAAATCGTGGCTCTTATAGAAGGTGGT

ATTTGTGAGCTCAATACCAAGCTCAGGAACCTCGAGGGGATTCTTGAGATGGAAATAAATCGCGGACCATCCCTTGGAGAATTAGGAAGATAG

>Solyc04g025970.1.1 Solanum lycopersicum|M-type_MADS|M-type_MADS family protein

ATGGAGGGTAAGAAAAAAGCAGGCTGCCAAAGAATTCCACTAGAGAAAATAGAAAAAAAAGTTGCTCGATATGCCTCATTTTTTAAACGTCGTTTATGTT

TGTATAAAAAAGCAAGTGAACTCATTCAAGAACGTGATGTCGATATTGGAGTATTTATTTCTTCTCAAACTGGTAAGCCGTATTCATTTGTTCATCAGAC

TGCTAATGTAGTCATCAATCATTTTAAAAGTCCCACAACAATAGATCTAGGTGCACAATTCGCTGGTGCAGAGGCACGCAACAATGTGATTCAAATGAAC

GATATGCTAAATGACTTTGATGCAAGGGAAAAAGTTACAAAGAACCATATATGA

>Solyc04g047870.1.1 Solanum lycopersicum|M-type_MADS|M-type_MADS family protein

ATGTCAAGAAGAATTTTCAAGGGTCGTCAGAGGGTTGACATGGTGAAAATAACGAATGGGAGAAACTTAGGAGTAACATTTTCTAAGCGACGTGCTGGTC

TGTACAAAAAGGCTTGTGAACTTTGTATGCTATGTGGTGCTGAAATTGCCATTGTTATATTTTCTCCGGAGGGTAAAATTTTCTCCTTTGGTCACCCTAG

CGTGGAAACGCTGGTGGAGAGGTTCCTTGGGAGGAACCTTCCTCCACCAAATAACGATGTCCACAATCAACAAATTGTGGCTCATAGAGAAGCTGGAATT

CGTGAGCTCAATACAAGGCTCATGAACGTCGAGGGGGCTCTCCAGATGGAGAAAAATCGTGGAGAATCCCTTCAAGAAATTAGGAAGAAAGCTGATGGTG

TATGGTGGCAATCTCCTATCAAAGAACTTAACTTGTTCCATCTTCAACACTTGAAGAGGGCATTGGAAATTCTAAAGCAAAAGGTTGTAAAAGAGGCACA

GATGGTGAATAATAATGCATTCCCATTTCAGACATTAGGAAGTGCTTGGAGTCCTCCTAATTATACAAGCTAA

>Solyc04g056550.1.1 Solanum lycopersicum|M-type_MADS|M-type_MADS family protein

ATGGAGAGAAAGAAGACTAAAGGGCGTCAAAAGATACCAATGCAAAAAATAGAAAATAAGAATGCCCTGCTAACCACATTTTCAAAGCGTCGGAAGGGTT

TGTTCAAAAAAGCTAGTGAAGTTGTTACAGAATGTGATGTTGACATTGGAATAATGATGATTTCACCTTCTGGTAAGCCACACTCATTTTTTCACCCTAC

TGCTGATGCAATTGTTTCTCGTTTTCAGAATCCTGATATGCAGCTAAGCGAAGGTATTCGTCTAGACGCGACTACTGCTCGAAATCGAGTGAATCAACTC

AAAACCAGGCTCGAAGAACTTGATGCTATAGAAGATGCTCTGTTTGCTCAAACAATTTTTTATGACCAAATGGCAGAAACGCAACAAAAAAGTTCGTGGG

AGTCAATTGAACAACTCGATGCAGATGAACTAATCATAAATGAAGCTTGGTTACGTGACACTAATTTCAAAATTTGCGATCGTTTGAGCCAATTAGAAAT

TGGAGCTTCATCCTCATTGGGATGTGAATTTTTGGAGTATGAAGTTTGA

>Solyc04g056740.1.1 Solanum lycopersicum|M-type_MADS|M-type_MADS family protein

ATGGAGAGAAAGAAGACTAAAGGACGTCAAAAGATACCAATAAAAAAAATAGAAAATGAGGATGCCCTCCTTACCACATTTTCAAAGCGTCGCGAGGGTT

TGTACAAGAAAGCGAGTGAACTCGTTAGAGAATGCGACGTTGACATTGGAATAATGATGATCTCCCCTGCTGGTAAGCCACATTCATTTTTCCACCCTAC

TCTTGATGCAATTGTTACTCGTTTTCAGAATCCTGATATGCAGTTAAGCCAAGGTATTCTTCTAGACACGATCACTGCACGAAATAAAGTGAACGAACTC

AAAAACAGGCTTGAAGAACTTGACGTTGTAGAAGATGCTACGATTGCTCAAACAACTTTTTATGACCAAATGGCTGAAATAAGACAAAAAGGTTGGTGGG

AATCAATTGAACAGCTCAATGCAGATGAAGTGACCATATTTAATGCTTGGTTGAGTGACACTTGTTCCAAAATGTGCCATCGTCTAAAACAATTAGAAAA

TGGAGCTTCATCATCATTGGGACGTGGATCTTTTGGAGTGTGA

>Solyc04g064860.1.1 Solanum lycopersicum|M-type_MADS|M-type_MADS family protein

ATGAATATTAATAACAATACCAACGCCGCCGCCAACTCCGCCGCCGTCGCCGTCAAGAAAACTCAAGGGCGGAGGAAAATAGCAATTAAACCAATAGCAA

ATCAAAACAGTAGGCACGTTACCTTTAGCAAACGCCGTTTAGGTCTCTTCAAAAAAGCTAGCGAACTTTGCATTTTAACCGGCGCTGAAATCGCTATTAT

GGTTCAGTCGCTAAAACGACAGCGTTTGTTCACTTTTGGTCATCCGAGCGCCGACGCCGTCATTAACCGGTACCTTACCGGAAAATCAGAGGAACAAAAA

CCGGCGGTCGATGATCAGTTGAACTATGTGCAACAGAGCAATGAATATTATTCTCAGATCTGTAGAGAATTGGAGTTGGAGAAGAAGGTAAAGGAGGAGA

TTGTTATTGATGAATCGAAGATGGTGAATGGTGGTAGTAGTAGTAATAATGGAGGAGGAGGATTTTGGTGGAATGAATCGATTGATGAAATGGGAATTGA

AGAACTTGAGAAATTTATGTTTGCATTGGAAGAATTGAAGAAGAAAGTAAATATGAGATGTGATGAATTGAGTATGATAAATGGTTCTTCTTCAATGGCG

GCGGCGGCGACGGCGTCTACTTCAAGTATGAATCAAGCTATTGATTATTGTGCTTCCATTGTTCCTTTTGATTTCAATTACCCAGGAAATGCCCAATTTT

GA

>Solyc04g076680.2.1 Solanum lycopersicum|M-type_MADS|M-type_MADS family protein

ATGGGGAGAGGTAAGATAGTGATAAGGAGGATCGATAATTCGACGAGCAGGCAAGTGACGTTCTCGAAGAGGAGAAATGGATTGTTGAAGAAGGCGAAGG

AGCTAGCGATTCTGTGCGATGCGGAGGCCGGAGTTATTATCTTCTCCAGTACTGGAAAACTCTATGAATATTCAAACACCAGTAAAAAATAA

>Solyc05g013370.1.1 Solanum lycopersicum|M-type_MADS|M-type_MADS family protein

ATGGAGAAGAAGAATAAGGGCAACATGCTAAATTACAAGAAGAAAAAAGAAACTATCAAGAAAAAAACTAGAGAGCTTTCAATCCTCTGTGACGTTAAAG

CTTGTGTCATTCTTGTTGATCCTAATGGAAAAGTCGATACATGGCCTGAAAATCCCACTGATTTCAACCCCATTATTCAATCCTACAAAGAAAATCTCTG

TCACGGCAAGAGAAAACGAATTGACGATGATGGGTGTTTTGAGAAAAAGTCGAAAAAGAATCATGCTCTGTTTTGCGATGATGATGAAAATCAGTGGCTT

AATGATGTGTTTAGAGAGTCGAATGAAAGTTTGTTGGTGAAATTGAATTCGAAATTAGAAGCTGTGGATAGAAGAATTGAATTCCTGAAGATGATGAATT

ATGGGAATGGGGTTGTTGGAGGATCAAGTTCAAGTGCGAAAGAAAGTTTACTTGCTAATCAAGAAACCCATAATCGACTTGAAAACAGCAACGCGTATAA

TCAAGAAACAGAAATTGCTATGGCGGCTGAGTTTTGGGTGATTGGTGGTGATGAATCTGCTAATGATCGGGGCAAAGAAATTGATTTTCTGAGAGATAAT

GCGACAGTAAACAATTTGAATAATGTGCAGAATTTTGGGTATGATGATCATCTTTGGCCAGTCATCGCTGCTTCTGAATTTTCGACTTGTATTAATTAA

>Solyc05g015730.1.1 Solanum lycopersicum|M-type_MADS|M-type_MADS family protein

ATGGGGAGGAAGAAAGTGGAAATAAAGCGAATTGAAGATAAGAGCAGTAGGCAAGCAACTTTCTCCAAACGGAGAAATGGACTCATGAAGAAAGCTAAAC

AGCTCTCTGTTCTCTGCGATGTTGATGTCGCCGTCCTCGTCTTCTCCAGTCGCGGACGCCTCTTTGAATTCTCCAGTACCAACAGGTTCTTCTTTTATTT

ATTTTTGGCGAATAGTCTGCTAGATCCTTATATTGTATTGTGA

>Solyc05g051830.2.1 Solanum lycopersicum|M-type_MADS|M-type_MADS family protein

ATGGGAAGAAGTAAATTGCCATTGTTGAAGATTGAGAGTTTGACCAATAGACAAGTCACTTTTAGCAAGAGAAGAAATGGAATTCTCAAAAAAGTTTATG

AATTATCTGTTTTATGTGATGTAGATGTTGGTATTATAATGTTCTCTCCTTCTGGTCGTCTCACACATTATTCGCGTAAAAGAAGAATTGAAGATATTCT

TTCTGAGCTCATTAGTCTCCCAGATAGTGAAAGAGGATTCTACATCAACAATAAAGAGTCTGTACTTTGGAACTTAAGGAAGATTGAAATTGAAGATAAA

TTTTGCGATATTGAAAGGATAAATCCTGCATACGTCAACGCAAATGACACAACAAAGAAAATTCAAGATGAAATCAATGGCTTGCATTGTAAACTCGATG

AGGCTGAGGGATTATTAAGAATATTTGAACCAGATACACAAAGGATTACATCACTCCATGAGCTTGATTTATGTGAAAAACGTCTTCAAGTTGCATTAAA

TCAAGTTAGACAAAGAATGGAACAACTCTCTAGCAATAATACACCAAGTTATGAAGATAATATGGCGCAAATAAATGAACTTCTTCAACACATAGACAAC

ACACAAGTTCATGAGAAACCTCCTTATGACTTATGGTTAGAGCTTGAAGATTATAATCATGAGAACAACAATATTAATAGTCCTCTCTATACTGCCTCAG

AAACATCTTCAATTTCTCAAAGTTCTATGAACCTTCCATCCTCAACTACTTATGATACAATGTCCCAAACAAGTCTAAGTGGTGAGACTTATCAAAATAA

TAACAACTTTAAGCAATCACAACATTCAACAAGGACCTTACCAAATCTCACTTTACAAACTTCATTCAAATTTGCCAAGCCTGAAATGTCCCAAACAAGT

ATTGAAGGAAGTTTTAGTTGCCTCACTGATGAAAATTTGAAGAAATCAATATGTTCAAATAGGGTCTTCCCAGCTATCACTCCATTACAAACTTCATTCT

CTTTTGCCAAGGCTGAAATGGAAACTCCAACCTCAGCATTAAGACCATTGGCACCATATCTACAGGCTGAAGCAACAACATCTTCTTGTACTAATCAAGA

AGGGAACAATGAAATGTCTTGGTTTCAGCCCAAAGTGAAAAAATCCAAGCAATATCATTCAATTGACTAA

>Solyc06g033820.1.1 Solanum lycopersicum|M-type_MADS|M-type_MADS family protein

ATGGATCGCAATAAGATAATGAGGAAGAAAATTGAGGATCCAGTATCTCGTCAACAGTTCTACTTAAAGTGCAAGGATATCATTGTTAAGAAGTCAGATG

AGTTGGGGTTTTTATGTAACTCAAATATTGCACTGTTAATGGTTTCTCAAAATGGTGAAGTGACCAGCTATTCTAGAGGAGAAAGTTTTGAGGATATTAT

GGTCAAAGCAATGAACCAGCCTGTTCAACTGAATCGACGATCCATTCCAAATCCAGATGAAGAGCATTTGATGCAGAGTCTTGTGCAATCAAAATCTGAA

AGAGGAATGATTGAAAAGATTGCTATGTATGACACTCTACTTTTCTCGAGTTCTTTTATTTTGATCTATTTCTTTGTTGTGGTACTTTTCTACATGATTG

AAAAATTCTTGTTATTTTTGTTGGGTGTTTTTACGATGCTGATGATTGGACAACGTGCTTCAGTGTTGAGTTCTTATGAACCACAAGTGGAGAATATCAA

CACAACGGAAGAAGCAGATGCATATAAGGAATATATTCTGGGTGCTATTGAACGGGTTCAACGATCCAAAGTGAGCATATCTAAAAGCTAA

>Solyc06g033830.1.1 Solanum lycopersicum|M-type_MADS|M-type_MADS family protein

ATGGGTCGCAATAAGGTTGTAATGAAGAAAATTGAGGATCCAGAATCGCGTAAACACTTCTACTCAAAGCGCAAGGATGGTCTTGTTAAGAAGTCGAATG

AGTTGGGAGTTGTATGTCACACAAATATTGCATTGTTAATGTTTTCTCCAACTGGTGAAGTGACGACCTATTCTCGCGCAAAAAGTTCTTATGAACCACA

AGTGGAGAACATCAACAATGTTGAAGAAGCTGATGCATATAAGGGGTATCTTCTGGGTGCTATGGAACGGGTTCAACGATCCAAAGAACCATTAGGGGAT

GAAAGATTTGATGAACTAAATTGGATCACAATGGAGAGGTAG

>Solyc06g035570.1.1 Solanum lycopersicum|M-type_MADS|M-type_MADS family protein

ATGAAAAGGATTGAGAATGCAACCAGCCGGCAAGTGACTTTTTCTAAACGGAGAAATGGAGTTATAAAGAAAGCTTATGAACTATCCGTTCTTTGTGATG

CTCAAGTTGCTCTTATTATTTTCTCTAACAAAGGAAGACTCTTTCAATTCTCAAGCTCCTGGTAA

>Solyc06g048380.1.1 Solanum lycopersicum|M-type_MADS|M-type_MADS family protein

ATGGATCGCAATAAGATTATGATCAAGATAATTGAGGATCCAATATCCCGTCAACAGTTCTACTCAAAATGTAAGGATAGCATTGTTAAGAAGTCAAATG

AGTTGGGGCTTTTGTGTGACACAAATATTGCACTGTTAATGGTCTCTCCAAATGGTGAAGTGACCAGCTGTTCTGGAGGAGAAAGTTTTGAGGATATTAT

GAGCAATGCAATGAACCAATTTGATGAACTAAATCGACATTCTTACGAACCACAAGCGGAGAATATCAACACAGTCGAAGAAGCTGATGCATATGAGCAG

TATCTTCTGGGTGCTATAGGACGGATTCAACTATCCAAAGCAAAATTCTTGGATAATCAGGAATTTCTGAAGAGAAATGAAAATGTCGCGGAACCAGTAA

GGGATGGAAGACCTGAGAGAAACATATTGGATCACAATGGATGTAGCTGCAGCAGAAATTATGTTATATTAGTTTATATATTAAAACTTAGTACAATGAA

TAACTTGCACCAAGCAAGCAAGCAAGATTAA

>Solyc06g054680.1.1 Solanum lycopersicum|M-type_MADS|M-type_MADS family protein

ATGGAGAGAAAAAAAACTAAAGGACGTCAAAAGATACCAATGAAAAAAATTGAAAATAAGGATTCGATGTTTGCTTCATTTACAAAGCGTCGTGAGGGTT

TGTATAAAAAATCTAGCAAACTCGCTACAGAATACAATGTTGACATTGGAATAATGATGATTTCTGCTACTGGTAAGCCTCATTCCTTTTTTCACCCAAC

ATTTGATGCAGTTATTTCTCGTTTTCAGAATCATGATATGCAGTTTGGTGAAAGGACAAATCTAGAGGCAAATGATGCTAGAAATGAAGTGAATCAACTC

AAAACTAGGCTTGAAGAACTTGATGTCAGAGAAGACATTGCGATTGCTAAGAAAAATTCTTATGAACAAATGGAAGAAACAAGACAAAAAGGTTGGTGGG

AGTCGACTGAGCAGCTCAATGCAGATGAAGTGTTCATATTTGAAACTTGGTTGAATGAAACTAGTTCTAACTTACACCATCGTTTAAATCAATTAGAAAT

TGAAGCTTCATCCTCAATGAGACATGAATCTTTTGGAGTGTGA

>Solyc06g059780.1.1 Solanum lycopersicum|M-type_MADS|M-type_MADS family protein

ATGGAGAGAAAAAAAACTAAAGGACGTCAAAAGATACCAATGAAAAAAATTGAAAATAAGGATTCGATGTTTGCTTCATTTACAAAGCGTCGTGAGGGTT

TGTATAGAAAAGCTAGCGAACTCGCTACAGAATACAATGTTGACATTGGAATAATGATGATTTCCTCTACTGGTAAGCCTCATTCCTTTTTTCACCCAAC

ATTTGATGCAGTTATTTCTCGTTTTCAGAATCCTGATATGCAGTTTGGTGAAAGCACAAATCTAGAGGCAAATGCTGCTAGAAATGAAGTGAATCAACTC

AAAACTAAGCTTGAAGAACTTGATGTCAGAGAAGACATTGCAATTGCTAAGAAAAATTCTTATGAACAAATGGAAGAAACAAGACAAAAAGATTGGTGGG

AGTCGACTGAGCAGCTCAATGCAGATCAAGTGTTCATATTTGAAACTTGGTTGAATGAAACTAGTTCTAACTTACACCATCGTTTAAATCAATTAGAAAT

TGAAGCTTCATCCTCAATGAGACATGAATCTTTTGGAGTGTGA

>Solyc06g071300.1.1 Solanum lycopersicum|M-type_MADS|M-type_MADS family protein

ATGGCAGAGGAAGATGAAATCAAGAGGAGAACGAACAATCCCAAGTCTTATCAAGTGAGGAAAGAATGCATAAAAAGGAAATCGATGGAGCTTGCGACTC

TCTGCGATATCAAGGTTTGTACGGTTATTACTGGTCCTAATGGGGAACTGCAAACTTGGCCTGACGATTTCGATGCCTGTAAGCAAGTTCTCGATCTCTA

CTCTCAAAATTTGAAACCCGAAAAGAAGTACAAGGAATCATCGACACCAGAGCCAGAGCCAGAGCCAGAACCAGAACGAGGCGAAGAACAAGGAGAGAAA

GATCTCCTGACGCTAGTTGAATCAACGCTTGCTGCTGTCAATAGGAGAATTTGTATATTGGAGAATAAGGGGAAAAGAAAGAGAATCGAGTGA

>Solyc07g052700.2.1 Solanum lycopersicum|M-type_MADS|M-type_MADS family protein

ATGAAGAAAATTGAGGATTCAACATCCCGTAAACAGTTCTATTCAAATCGCAAGGATAGCATTGTGAAGAAGTCAAATGAGCTGGCGGTTGTATGTGGTA

CAGATGTGGGGTTGTTGATGTTTTCTCCATCTGGTCAGCTGACTACCTATTCTAGCAAAGAAAGTATTGAGGACATCATGATCGAAGCTATGAACAAGTC

TGTGAATCCGCGACCCATACCAAATCTAAATGAACAGCTTTTGATGCAGAGTCTCAAACAGTCAAAATCTGAAGGCCAAATGGTTGGAAAAATAGCTATT

GCTGAGGCTCATGAGAAGAAGCTTAATGAGCTCAAAGAAACACTAAGGGAGGCACAACAGAAAATAAGGTATTGCAATCCGCAAGTGGAGAATATCAGCT

CAGTCCAAGAAGCTGAAGCATATGAGCAGTTCCTTAGGAGTAATATGGAACAGATTCAACAATCAAAAGCAAAACTCTTAGGTGTCCAAGGATTAGTCCA

TAGAAATGAATATCCTGCGGTCAACACAGAGGATACGGCTGCTGCAGGAACCAGTAGTGGATGGATGTTTTGA

>Solyc09g061950.1.1 Solanum lycopersicum|M-type_MADS|M-type_MADS family protein

ATGGAGAATGAGAACAAAAAAGGCAAGCAAAAAATTGAAATGAAGTTGATTGAGAATGAGAGAGCACGTATGGTGAGTTTCTCAAAAAGAAAAAAAACTT

TGTTCGAGGATGCACATAAGTTTGCAACTCGGACTGGAGCAGATGTTGGTGTTATGCTATTTTCACCAAGTGGAAAACCATATTCTAATGATTCCGCAAC

CATAGCAGATATAATTGATAGATTTCTCAAAGTGAAACAGGAGGATCACAAACGTGATTATGCTGAGGGAGAATCAAATGGTTTCGAGGCATTGAAAGAT

CTCCATAAAGAATTACAAGCATGGAACGACAAAGAGAAAAAACGAAAACTAATGCATAAGATTATGCACCCTAGCTTAGAAATACCTTCAGATAAACACA

TGGAGGAGCAGAAGCTAGCATTGAAGTTGAAGGTAGAGAAATTTAAAAATGAAATACAAAGTGCTATTACGACTGAGCATCTAAAGTTTGACCTAAATGT

CGTTCCTGATCCAGAAGAGTAG

>Solyc10g012180.1.1 Solanum lycopersicum|M-type_MADS|M-type_MADS family protein

ATGGAATCAAAAAATTCCAATGTTGATTCAGCAGTTGCAAAAGATAAACGTGGTGAGGGTGGTAAAATCTCAAAAATGCAAAAAGCATTGTTCAAAAAAG

CAAGCGACCTATCCATATTGTGTGGAATTCAGGTCGCCATCATAATTCTCTTCATTAACCGTCAACCAATTGTATTTGGAAAGCCTGATGCAGAATCGGT

TATTAACCAGTTCATTGAGGCCAACCATCCAACCGCACCTCGATTTTATATGAAGATGAAAAAGAAGGAAGAAGAAAATAAGGAGAAAGGAAAATCCATT

GAAGATGATATACAATCACAAGATTTTGAGTCTCCTTATTTGGGAAGTCTTTTAAAGTTGTACGAAGGGCTCACAGAATTTGAAAATCAATTGACTAAAG

AGATTGATCTCACACAATTGAATCAAGAGATTGAGAAGCATGAAGATCCAAAATTAATGAATGTGGCTAGTTCTTCGACTTTACCAACTAATTTTAGTCC

ATAG

>Solyc10g012200.1.1 Solanum lycopersicum|M-type_MADS|M-type_MADS family protein

ATGGAATCAAAGAATTCCAATGTTGATTCAGCAGTTGCAAAAGATAAACGTGCTGAGGGTGGTAAAATCTTAAAGATGCAAAAAGCTTTGTTCAAAAAAG

CAAGCGACCTTTCAATTTTGTGTGGTATTCAGGTCGCCATCATCATCCTCTTCATCAACCGTCAACCAATTGTATTTGGGAAGCCTGATGCAGAATTGGT

TATTCACCAATTTATTGAGGCCAACCATCCAACCGCACCTCGATTTTATATGAAGATGAAAAAGAAGGAAGAAGAAAATAAGAAGAAAGGAAAATCCGTT

GAAGATGATATACAATCACAAGATTTTGAGTCTCCTTATTTGGAAAGTCTTTTAAAGTTGTACGAAGGGCTCAGAGAATTTGAAAATCAATTGACTAAAG

AGATGGATCTCAAACAATTGAATCAAGAGATTGAAAAGCACAAAGATCCAAAATTAATGAATGTGGCTAGTTCTTCGACTTTACCAACTAATTTTAATCT

ATAG

>Solyc10g012380.1.1 Solanum lycopersicum|M-type_MADS|M-type_MADS family protein

ATGCAGAAATCACTTTTTAAGAGAGCAAACGACCTTGCTATCTTATGTGGAATCCACATAGCTATCCTAATTTTCTCAGTCGGCCGTCAACCAATTTTTT

TTGGAATGCCCGATGTTGAAACGGTTGTCCAAAAATTCATGGAGGCTAACCATCCAACCGCACCTCGATTTTATATGAAGATAAAAAAGACTGAAGAAGA

AAACAAGGAGAAAGGAAAATCTGTTGAAGATAATATCGCACATCGGCAACTAGAAGATTTCGAGTCTCCTTATTTGGGAAGTCTTCTGAAGTTGTACCAA

GGGCTCACAGAATTTGAAGATCTATTGAATAAGGAGATCGATCCCACACAATTGAATCAAGAGATCGAAAAACATGAAGATCCAAAAATAGTATCTAAAA

TGAATGTGGCTAGTTCTTCGTATTTACCAACTGATATGCTTAGTCCTTAA

>Solyc10g012390.1.1 Solanum lycopersicum|M-type_MADS|M-type_MADS family protein

ATGCAGAAATCACTTTTTAAGAGAGCAAACGACCTTGCTATCTTATGTGGAATCCACATAGCTATCCTAATTTTCTCAGTCGGCCGTCAACCAATTTTTT

TTGGAATGCCCGATGTTGAAACGGTTGTCCAAAAATTCATGGAGGCTAACCATCCAACCGCACCTCGATTTTATATGAAGATAAAAAAGACTGAAGAAGA

AAACAAGGAGAAAGGAAAATCTGTTGAAGATAATATCGCACATCGGCAACTAGAAGATTTCGAGTCTCCTTATTTGGGAAGTCTTCTGAAGTTGTACCAA

GGGCTCACAGAATTTGAAGATCTATTGAATAAGGAGATCGATCCCACACAATTGAATCAAGAGATCGAAAAACATGAAGATCCAAAAATAGTATCTAAAA

TGAATGTGGCTAGTTCTTCGTATTTACCAACTGATATGCTTAGTCCTTAA

>Solyc10g017640.1.1 Solanum lycopersicum|M-type_MADS|M-type_MADS family protein

ATGGTGAGAGGGAAAACTGAAATGAGGCGTATCGAAAACGCGACAAGCAGGCAAGTCACTTTCTCAAAGAGAAGAAATGGATTGTTAAAAAAAGCCTTTG

AGCTTTCTGTTCTTTGTGATGCTCAAGTTGGATTAGTTATTCTTTCTCCTAGAGACAAACTTTATGAATTTTCAACCTCAAGGTAA

>Solyc10g018070.1.1 Solanum lycopersicum|M-type_MADS|M-type_MADS family protein

ATGGAGCATAAGAAGACAGCAGGACGCCAAAAAATTTCATTGGCAAAAATAGAGAATGAATCTGCTAGACTCACCAAATTCTCTAAACGTCGTTCTGGCT

TATACAAAAAAGCTTGTGAACTTGTTAGAGAATGTGATGTAGATCTTGGAATTGTTATGTCTTCACTGAAAGGTATACCTTATTCCTTTAGTAGTCCAAC

CTCTAATGTGGTCATTGATCGTTTTATAAATCCTACAGCAAATTTAAGTTCAAGTGACCGTCTTGTTGCTGTAGAAACACGCAAAAAACTAGAAAATGGA

GCTTCATCCTCCTCACAAATTCCATCAGATGATGCAAATATCTCACCAAATGTCTTCTAG

>Solyc10g018080.1.1 Solanum lycopersicum|M-type_MADS|M-type_MADS family protein

ATGGAGCATAAGAAGACAGCAGGATGCCAAAAAACTTCATTGGCGAAAATAGAGAATGAATCTGCTCGACTCACCACATTCTCTAAACGACGTTCTGGCT

TATACAAAATAGCTTGTGAACTTGTTAGAGAATGTGATGTAGATCTTGGAATTGTTATGTCTTCACCGAAAGGTATACCTTATTCCTTTAGTAGTCAAAC

CTCTAATGTGGTCATTAATCATTTTATAAATCCTACAGCAAAATTAAGTTCAAGTGACCACCTTGTTGCTTCAGAAGCACGCAAAAGAGTAAGTCAATTT

AATGATATTTTAAACGAATTGGACGAAAGAGAAAAAATTGCAAATGAAAAGTTAGACCAAATGAATGAGGCTAGAGATCTAGGTTGGTGGGAGTCCATAG

ATCGATTGAATGTTCATGATGTAATGAAGTTGGAAGCATGGCTGAATTTACATTGA

>Solyc10g018110.1.1 Solanum lycopersicum|M-type_MADS|M-type_MADS family protein

ATGGAGCATAAGAAGACGGCAGGACGCCAAAAAATTTCATTGGCGAAAATAGAGAATGAATCTGCTCGACTCACCACATTCTCTAAACGACGTTCTGGCT

TATACAAAAAAGCTTGTGAACTTGTTAGAGAATGTGATGTCGCTCTTGGAATTGTTATGTCTTCACCGAAAGGTATACCTTATTCCTTTAGTAGTCCAAC

CTCTAATGTGGTCATTGATCGTTTTATAAATCCTACAGCAAATTTAAGTTCAAGTGACCGCCTTGTTGCTGCAGAAGCACGCAAAAAAGTAAGTCAATTT

TATGATATTCTAAACGAATTGGACGAAAGAGAAAAAATTGCAAATGAAAAATTAGATCGAATGAATGAGGCTAGAGATCTAGGTTGGTGGGAGTCCATAG

ATCAATTGAATGTACGTGATGTAAAGAAGTTGGAAGCATGGCTTATATCTGGTGAATTTAAATTGAACGAACATTTGGAGCAGCTTGAAAATGGAGCTTC

ATCCTCCTCACAGATTCCATCAGATGATGCAAATATCTCACCAAATGTCTTCTAG

>Solyc10g050900.1.1 Solanum lycopersicum|M-type_MADS|M-type_MADS family protein

ATGGAGAATAAGAAGACTAAAGGACGTCAAAAGATACCAATGAAAAAAATAGAAAATGAAAAGGCCTTGTTAAGTTCATTTTCAAAGCGCCGTAATGGTT

TGTTCAAAGCAGCGAATAATCTCGTAAAAAAATTTGATGTAGACATTGGAATAATAGTGTTTTCTCCTACTGGTAAGCCTCATTCATATTTTCATCCTAC

AGTTGATGCAGTCATTTCTCGTTTTCAGAATCCTGATATGCAGTTAAGTGATGAAACTCACCTGGCCATGATTTTTGCTCGAAATTCGGTGAATCAACTC

GAAAAAAAGCTTGAAGAACTTGACATCCAAGAAAAAATTGAAATTGATCGAACAAATTATCTTGACCAAATGACAGAAACTAGACAGAAAGGTTGGTGGG

AATCAATTGAGCAACTTAATGAAGATGAAGTGTCCAAGTTTGAAGAATGGTTGAATGTTGCTAGTTTTACTATGCACTACCGTTTGAACCAATCAATTGT

GTCATCCTAA

>Solyc10g050940.1.1 Solanum lycopersicum|M-type_MADS|M-type_MADS family protein

ATGGAGAATAAAAAGGCTAAAGGACGTCAAAAGATACCAATGAAAAAAATGAAAAATGAAAAGGCCTTGTTAAGTTCATTTTCAAAGCGCCGTGATGGTT

TGTTCAAAGTAGCGAATAATCTCGTAAAAGAATTTGATGTTGACATTGGAATAATAGTGTTTGCTCCTACTGGTAAGCCTTATTCATTTTTTCACCCTAC

AGCTGATGCAGTCATTTCTCGTTTTCAGAATCCTGATACGCAGTTAAGTGATGAAACTCACCTGGCCATGGTTTTTGCTCAAAATTCGGTGAATCAACTC

GAAAAAAAGCTTGAAGAACTTGACATCAAAGAAAAAATTGAAAGTGATCGAACAAATTATCTTGACCAAATGACAGAAACTAGACAGAAAGGTTGGTGGG

AATCGATTGAGCAACTTAATGAAGATGAAGTGTCCAAGTTTGAAGCATGGTTGAATGTTGCTAGTTTTACTATGCACTACCGTTTGAACCAATCAATTGT

TTCATCCTAA

>Solyc10g050950.1.1 Solanum lycopersicum|M-type_MADS|M-type_MADS family protein

ATGGAGAATAAGAAGATTAAAGGACGTCAAAAGATACCAATGAAAAAAATAGAAAATGAAAAAGCCTTGTTAAGTTCATTTTCAAAGCGTCGTAATGGTT

TGTTCAAAGCAACGAATAATCTCATAAAAGAATTTGATGTTGACATTGGAATAATAGTGTTTTCTCCTACTAGTAAGCCTCATTCATTTTTTCACCCTAC

AACTGATGCAGTCATTTCTCGTTTTCAGAATCCTGATATGCAGTTAAGTGATGAAACTCACCTGGCCACAGTTTTTGCTCGAAATTCGGTGAATCAACTC

GAAAAAAAGCTTGAAGAACTTGACATCAAAGAAAAAATTGGAAGTGATCGAACAAATTATCTTGACCAAATGACAGAAACTAGACAGAAAGGTTGGTGGG

AATCAATTGAGCAACTTAATGAAGATGAAGTGTCCAAGTTGGAAGCATGGTTGGATGTTGCTAGTTTACTATGCACTACCGTTTAA

>Solyc11g020320.1.1 Solanum lycopersicum|M-type_MADS|M-type_MADS family protein

ATGGGAAGATCAAAAATCAAAATGGAACTTATTGAAGATCACAAGAAGAGGAAGTCTACTTTGGTAAATCGAAAAGCTGGCTTAGTCAAGAAAATCTCAG

AACTCTCAATACTTTGTGACATTAAAGCAAGCATGATCATATATGAAGGAAATTATAATTATCAAATTTGGCCAAATGATTCAAATGACGTTCAAGACCT

AATAAATCTCTACAAAAATCAGTCACAGGATGGGCGGACTAAGAGGGGTAAGACCTTGTCCAACTTCTTCAAAAATGATGAAAAGAAAAACAATGAGTTC

AAAGTAGAAAAGTATCCTACTTGGGATTCAAGATTTGGCTATTTATCTCAAATAGAATTACAAAATCTTGTTGGTGTTGTTGAGAAAAGGATTGAGAAGG

CAAAGGAAAAAATCGAATTGTTGAAGAGCATGAATGATCAAGATCCAAATATTGGAAGTTCCTCGTTTTCTCATCAACAACAAATATGGAACAACAACAA

CTTGATGAACCAAACTACTCAATGGCCTTTTTCTTATGTGAATCCTTTTACTCACTACGACAACTTCTTTCAAGCAAATATTCCAATTAGTGGTGCAAAT

TCAATGGGCGCGGGAATGGATGATGGACTTCTTACTATCGATGATTATCAATTTAACAACACAGATTATTCTACGATGATTGAGACAGAAAATTGTTTGG

TGAAGAATGGAATTGGTTCAAGTTCAATAATGCATCCTATGATGAATAATGGAATTGATTCAAGTTCAACAATGGAGTACCCTTTCATATACAATGGCTA

TACTCATATGCCTTATGGATTTCAGTAA

>Solyc11g020620.1.1 Solanum lycopersicum|M-type_MADS|M-type_MADS family protein

ATGGGAAGATCAAAAATCAAGGTGGAACTAATTCAAGATGACAAGAAGAGGATGAAAACTTTAGTGACACGAAAGGCTGGCTTGTTCAAGAAAATTTCAG

AACTCTCTATACTTTGCGATATCAAAGCATGCATGCTCATATATGATGAAGGAAATAATAATAATTGTGAAATGTGGCCGAATGATCCAAATGAGCTCAT

AAATCTATACAAAAATCAACCATTTGAAGGACCAACCAAAAGGGGTAAAACATTGTCCGGAGATGAGATCAAAGTAGAAAAGGATCCGGATTCAAGATTT

GACTATTTCGAGAATAATGAGAAGAAAAAGGCTGATGCGATCCAAGTAGAAAAGTATCCTACTTGGGATTCAAGATTTGACTATTTATCTCAAAAAGAAT

TGCAAAATCTTGCTGGTGTTCTTGAGAATAGGATGGAGAATGCAAAAGGAAGAATAGAATTGTTGAAGAGCATGAATGGAAGTTGCTCACTTTCTCATCA

ACAACAAATATGGGACTATAACAACTTGATGAACCAAACTACTACTCTGTCTAACAACAACTTGTTTCAATCAAATATTCCAATTAGTAGTACTGTGAAT

TCAATGGGAGCAGGGATGGATTATCACTTTTGTAATGCTAATTATTGTAAGATGAATGAGTCTGAAAACTGGTATGGAGTTGGTTCAAGTTTGACAATGA

TGCAGCATAATATGGCAAATAATGGAATTGGTTCAAGTTCAACAATATTGCATCTTATGGGGGATGATTATGGGATTATTGATTCAAGTTCAACAATGAT

GCTGCCTATGGGGAATAACGAAATTGGTTCAAGTTCGACAATGCAGCAGCCTATGTATCAGTACCCTTTCATGTACAATGGCTCTACCCATGTCATTGGT

TCAAGTTCGACAATGCAGCCTCCTATGGGTCAGTACCCTTTCACAAACAATGACGACTCTACTGGGATATTGGTTCAAGTTCAACAATGCAGCCTATGGG

GAATAATAATGAGATTGGTTCAACTTTGA

>Solyc11g020660.1.1 Solanum lycopersicum|M-type_MADS|M-type_MADS family protein

ATGAACACTTCTGTAAAACGGAAGGCTAGCTTAGTCAAGAAAATTTCAGAACTCTCAATACTTTGCGACATCAAAGCCTGCATGATCATATATGATGAAG

GAAATAGTAATTGTGAAATGTGGCCAAATGAAGTTAAAGAGCTAATAAATGTCTACAAAGATCAACCATTTGAAGGACGAACCAAAAGGGGTAAGACATT

GTCCAACTATTTCAAAGATGAGATCAAAGTAGAAAAGGATCTGGATTCAAGATTTGACTATTTCGAGAATGATGAAAAGAAAAAGGCTGATGCAATCAAT

GTAGAAAAGTATCCGACTTGGGATTCAAGATTTGACTATTTATCTCAAAAAGAAATACAAAATCTTGTTGGTGTTATCTCAAAAAGGATGGAGAATGCAA

AAGGAAGAATAGAATTGTTGAAGAGCATGAATGGAAGTTGCTCACTTTCTCACCGACAACAAATATGGGACTACAACAACTTGATGAACCAAACATCTCC

ATGGCCTATTTCTGATCATGTGAATTCTTTCAACAACTTTTTTCAATCAAATATTGGTGTGAATTCAATGATGGAGTCAGAAAACTGGTTGGCAAATGAT

GAAATTGGTTCAAGTTTGGCAATGCATCCTATGGGGAATAATTATGGGATTATTGATTCAAGTACGACAATGATGCAGCCTATGGGGAATAACGAAATTG

GTTCAAGTTCAACAATGCAGCAGCCTATGTTTCAGTACCCTTTCATGTACAATGGCTCTACCCATGTCATTGGTTCAAGTTCGACAATGCAGCCTCCTAT

GGGTCAGTACCCTTTCACAAACAATGACGACTCTACTGGGGATATTGGTTCAAGTTCAATGCAGCCTATCGGGAATAATAATGAGATTGGACCCATGGAC

AATGACTCTACTTATGATTCCTGTACATTTTATTATTGA

>Solyc11g069770.1.1 Solanum lycopersicum|M-type_MADS|M-type_MADS family protein

ATGGGAAGAGATTTAATCATGGCAAGGCTTGGTCGCAGAAGAATCCGCATTGAAAAGATAGAAAACAAAAAGAAGAGGAGTGTCACATTCTCTAAAAGGC

GTTATGGCCTTTTTAAGAAAGAAAGTGAGTTATCAATGCTTTGCGACAGTCCAAATGCCACGGTAGTTTTTTCCCCTGACGACGAAACTTGTGTTTACTC

GATTGGGTACCCTTGTGTCAATTCAGTTCTGGATAAGTTTATGGACGTGAATCCTCCACAAAACCTTGATGACACTGGCTCCCTCAGTGTACATCGTCGA

AATGCTATTAGAGAGGGGGTGTTGGCTCTAATGGAGATTGAAGAAGAGTTTGAAGAAGAGAAAAAACGCGAAAAAAGCCTAGACACGGGAATTTCATATG

AAAATTTGAATTCCTCTGTGTATCAGAATTTCATCGAAAAAATTGAGATCGGCTATATGGAAGCGGAACAGCTAGCTATCGAACTAAAGGAGCGTAACGT

ACCATTTCCCTACAGTACCTTTGGAGATGCCTTAGCTCCTAAATAA

>Solyc12g005210.1.1 Solanum lycopersicum|M-type_MADS|M-type_MADS family protein

ATGACTATGAAGGGGGTTAGGAGCACTAGGAATGTAGATCCTAGAATGAAAAAATTCATTTTAGATAAAAGAATAGAAGCACTATTTAAGCAAGCAAATG

ACTTGTCAATTCTATGTGATATTGAAGTTGGTGTAATTGTTTTTGGTCCAGGAGAAAACAATGCTGTTGTTTGGCCATCTCTAGCACAGGCTAGTGATAG

AGTGAAAAACTATTTAGCTAGGCATCGAGACTGCAAAGGTAAAGAAGTTGTCAACGTACGTCAATGA

>Solyc12g016150.1.1 Solanum lycopersicum|M-type_MADS|M-type_MADS family protein

ATGGCTACAAAAAGGTTGAGAGATACTGCGAACTACAATGAAAATGTGAGAAATTCCATCTTAGATAAAAGAGTAACAAGTTTGTTCAAGAAAGCAGAAG

AGCTATCTATTGTGTGTGACGTAGAAGTTGCCATAATTATTTTTAGGCCAGGAAAAATCCAACCCATTACTTGGAAATCCCCAAGTCTGGCTCAGGATGT

CTTGACGAGGTATTTAAGTTTTATTGAATTCAAAAGGCTGTCAAAGTTGGTCACACATGAAGACTATCTTCAAAAGAAAGTTGATAAGAAAGAAGAACAA

ATTAGTAAATTAGAGAAAATGAATGAGATGGAAGAATCAATCAATGATCCGTGGTTTATTCAGACTATAGCTACATTAGGGGATGTGAGCGGTGTAGAGT

CTGCACAAAAAGAAGGCAAAGGCGTCAATGTTGAAGATGATGGACATTCCAAGGACCTCGATTGA

>Solyc12g016170.1.1 Solanum lycopersicum|M-type_MADS|M-type_MADS family protein

ATGGCTATAAAAAGGCTTAGAAATACTAGGAACTACAGTGAAAATGTGAGAAATTCCATCTTAGATAGAAGAGAAAAAAGTTTGTTCAAGAAAGCAGAAG

AGCTTTCTATTTTGTGTGATGTGGAAGTTGCCATAGTTATTTTTAGGCCAGGAAAAATCCAACCCATCACTTGGAAATCCGCAAGTCTGGCTCAGGATGT

CTTGACAAGGTATTTAGGTTTCATTGAGTTCAAAAGACTTAATAAGTTGGTCACACATGAAGACTATCTTCAAAAGAAAATTGATAAGAAAGAAGAACAA

ATTAGCAAATTAGAGAAAATGAATGAGGCGAAGGAAATGGAAATCCTCTTCAACCAACTAGTGGAGGGAAAAAGTATTAATGAACTTGATGCTAGAGAAA

TGAAAGGCTTGTTAAAGGTGTTTGCTGCAAAAATGGCTAAACTAGATGAAAGAAAGAAAGAACTTAACCAGACTCCAAATCCTCCATCAAACAAAGAAAA

CATTACTTTATCAGGAAGTCCAATGGAAGAATCATTCAATGGCCCGTGGTTTATTCAGACTATAGCTACGTTAGGGGATGGAAGTGATATAGAGTTTTCA

CCAAAAGAAGGCAATGGCGTCAATGTTGAAGATGATGGACATTCCAAGGACCTCGATTGA

>Solyc12g016180.1.1 Solanum lycopersicum|M-type_MADS|M-type_MADS family protein

ATGGCTATAAAAAGGCTTAGAAATACTAGGAACTACAGTGAAAATGTGAGAAATTCCATCTTAGATAGAAGAGAAAGAAGTTTGTTCAAGAAAGCAGAAG

AGCTTTCTATTTTGTGTGATGTGGAAGTTGCCATTGTTATTTTTAGGCCAGGAAAAATCCAACCCATCACTTGGAAATCCGCAAGTCTGGATCAGGATGT

CTTGACAAGGTATTTAGGTTTCATTGAGTTCAAAAGACTTAATAAGTTGGTCACACATGAATACTATCTTCAAAAGAAAATTGATAAGAAAGAAGAACAA

ATTAGCAAATTAGAGAAAATGAATGAGGCGAAGGAAATGGAAATCCTCTTCAACCAACTAGTGGAGGGAAAAAGTATTAATGAACTTGATGCTAGAGAAA

TGAAAGGCTTGTTAAAGGTGTTTGCTGCAAAAATGGCTAAACTAGATGAAAGAAAGAAAGAACTTAACCAGACTCCAAATCCTCCATCCAACAAAGAAAA

CATTACTTTATCAGGAAGTCCAATGGAAGAATCATTCAATGGCCCGTGGTTTATTCAGACTATAGCTACGTTAGGGGATGGAAGTGATATAGAGTTTTCA

CCAAAAGAAGGCAATGGCGTCAATGTTGAAGATGATGGACATTCCAAGGACCTCGATTGA

>Solyc12g017300.1.1 Solanum lycopersicum|M-type_MADS|M-type_MADS family protein

ATGGCTACAAAAAGGCTCAGAGACAGTAGGAATTACAGTGAAAATGTGAGAAATTCCATCTTAGATAGAAGAGAAATAAGTTTGTTCAAGAAGGCAGAAG

AGCTTTCTATTTTGTGTGACGTAGAAGCTGCCATAATTATTTTTAGGCCAGGAAAAATTCAACCCATTGCTTGGAAATCCGCAAGTCTGGCTCAGGATGT

CTTGACAAGGTATTTAAGTTTTCTTGAGTTCAAAAGGCTCGATAAGTTGGTCACACATGAAGACTATCTTCAAAAGTTAGTTGATAAGAAAGAAGAACAA

ATTACCAAATTACAGAAAATGAATGAGATGGAAGAGTCATTCAATGACCCCTGGTTTATTCAGTCTATAGCTACATTAGGGGATGGAAGTGGTATAGAGT

CTACACCAAAAGAAGGCAATGGCGTCAATGTTGAATATGATGGACATTCAAAGGACCTCGATTGA

>Solyc12g087820.1.1 Solanum lycopersicum|M-type_MADS|M-type_MADS family protein

ATGGGGCGAAGGAAGGTAGAAATTAAGCGAATTCAAGATAAAAATTGCAGGCAAGTTGCGTTCTGTAAACGGAGGAAAGGTTTATTGAAGAAAGCTAAAG

AAATTTCCGTTCTCTGCGATGTCGATGTTGCTGTTGTTATCATCTCAAATCGAGGCAGGCTCCATGAATTCTCCAGCAATAACAGGACTAGCACACCATG

TTGGCTTGATAAAATCTCCCCAATGCATCAGGTTGAATTGATAACTCTAGGAAGCTCCACTTTAGGTGGCACAATGTGTGCATTACTTCAAGTTACTCGT

CGTGCTTTGCCTACATTCTAG

>Solyc12g088080.1.1 Solanum lycopersicum|M-type_MADS|M-type_MADS family protein

ATGGGGAGAGGAAAGATATTGATAAGGAGGATCGATAATTCAACGAGCAGACAAGTGACGTTTTCGAAGAGGAGGAATGGATTGTTGAAGAAGGCGAAGG

AGTTAGCGATTTTGTGTGATGCGGAGGTTGGAGTCATCATCTTCTCCAGTACTAGCAAGCTCTATGACTATGCAAACACCAGGTTTTCCCCCCCTATTTC

TCACACTTTTTTTGCCTGTCAGATGCTTGAACTGCAATTTATATTTAATTTTCTGGAAAAAAACTTTTTGCGTGTGTTTTCATTCGTCTATTAG

#amino acid sequences of MADS-box genes in Solanum lycopersicum

>Solyc01g087990.2.1|Solanum_lycopersicum|MIKC_MADS|Solyc01g087990.2.1

MGRGKIDIKLIENLNNRQVTFSKRRAGLLKKAGELSVLCDSEVAVIIFSSTGKLFEFSSTSMKQTLSRYNKCVASTDNSAVEKKSEDNEQPQLQQQTHVL

KQEQKEVDSLKDELAKLKMKQQRLLGKDLNGMGLNELRLLEHQLNEGLLAIKERKEELLIQQLEYSRKQEERSALECETLRRQVEELRGLFPLSASLPPP

FLEYDRPLEKKYSILKESKESLDSDTACEDGVDDEDSNTTLQLGLPTICRKRKRTEQESPSSNSENQVGSK*

>Solyc01g093960.2.1|Solanum_lycopersicum|MIKC_MADS|Solyc01g093960.2.1

MGRGRVELKRIENKINRQVTFSKRRNGLLKKAYELSVLCEAEVALIIFSSRGKLYEFGSAGITKTLERYQRCCLNPQDNCGERETQSWYQEVSKLKAKFE

ALQRTQRHLLGEDLGALSVKELQNLEKQLEGALAQARQRKTQIMMEQMEELRRKERHLGDVNKQLKIKVSLELSSFEGEGQGVPFPWSNCNASLDEAGSS

TFHVHHSQSNHMDCDLPDPVLQIGYHQYMAADGASGSRNMAVESNIIHGWGL*

>Solyc01g105800.2.1|Solanum_lycopersicum|MIKC_MADS|Solyc01g105800.2.1

MEQITIQLKSSNSNSHEEIQLLLCNQKYPNYYFFSFYCNNIPFFSFFSSQLQISSPFLANFSKEMVRQKIQIKKIDNLTARQVTFSKRRRGLFKKAQELS

TLCDADIGLIVFSATGKLFEYSSSSMMQLIEKHKMQSERDGMDNPEQLHSSNILSEKKTHAMLNRDFVEKNRELRQLHGEELQGLGLDELMKLEKLVEGG

ISRVLKIKGDKFMKEISSLKKKEAKLQEENSQLKKQSQARLNEEGQNVIEQGHSADSITNNRSLVNDSDTSLKLCLAFP*

>Solyc02g065730.1.1|Solanum_lycopersicum|MIKC_MADS|Solyc02g065730.1.1

MGRGRVEMKRIENKISRQVTFSKRRSGLLKKTNEISVLCDAEVALIVFSSNGKLFEYSTQSSMENILERYENYSYEEMNLNTTYKENWTLEYPKLMARVE

LLQRNIRHFMGEDLDAFNLREFRGLEKQLDTALKRVRSKKNQLMHESISQLQKKEKELQQRNNLISNKLKENEKKQIVQTNPGQSSTMTFLLQSPTVTNQ

TIGGPSQATDQSQNRDGYNSLMPPWMFHHVHNKG*

>Solyc02g071730.2.1|Solanum_lycopersicum|MIKC_MADS|Solyc02g071730.2.1

MDFQSDLTREISPQRKLGRGKIEIKRIENTTNRQVTFCKRRNGLLKKAYELSVLCDAEVALVVFSNRGRLYEYANNSVKATIERYKKACSDSSNTGSVSE

ANAQYYQQEASKLRAQIGNLMNQNRNMMGEALAGMKLKELKNLEQRIEKGISKIRSKKNELLFAEIEYMQKREVDLHNNNQYLRAKIAETERAQHQHQQM

NLMPGSSSNYHELVPPPQQFDTRNYLQVNGLQTNNHYPRQDQPPIQLV*

>Solyc02g084630.2.1|Solanum_lycopersicum|MIKC_MADS|Solyc02g084630.2.1

MGRGKIEIKKIENSTNRQVTYSKRRNGIFKKAKELTVLCDAKISLIMLSSTRKYHEYTSPNTTTKKMIDQYQSALGVDIWSIHYEKMQENLKRLKEINNK

LRREIRQRTGEDMSGLNLQELCHLQENITESVAEIRERKYHVIKNQTDTCKKKARNLEEQNGNLVLDLEAKCEDPKYGVVENEGHYHSAVAFANGVHNLY

AFRLQPLHPNLQNEGGFGSRDLRLS*

>Solyc02g089200.2.1|Solanum_lycopersicum|MIKC_MADS|Solyc02g089200.2.1

MGRGRVELKRIENKINRQVTFAKRRNGLLKKAYELSVLCDAEVALLVFSNRGKLYEFCSTNNMLKTLDRYQKCSYGTLEVNRSIKDNEQSSYREYLKLKA

KYESLQRYQRHLLGDELGPLTIDDLEHLEVQLDTSLKHIRSTRTQMMLDQLSDLQTKEKLWNEANKVLERKMEEIYAENNMQQAWGGGEQSLNYGQQQHP

QSQGFFQPLECNSSLQIGYDPITTSSQITAVTNAQNVNGMIPGWML*

>Solyc02g089210.2.1|Solanum_lycopersicum|MIKC_MADS|Solyc02g089210.2.1

MGRGRVELKRIENKISRQVTFSKRRSGLLKKANEISVLCDADVALIVFSTKGKLFEYSSNDSSMESILERYERCSYAERQMNANDSDPKENWSVEYPKLM

SRIELLQRNIRHYMGQDLDPLSLRELQSIEQQIDTSLKRIRSRKNQLMHESISELQKKEKALQEQNNLITKKLKENEKTQPNSSGQNSATVHVFPSHSHH

QLPNLTIGGAFGGMNRDGSGQAHHYPGSNNNNNNSSLIPPWMLRHVSNEG*

>Solyc02g091550.1.1|Solanum_lycopersicum|MIKC_MADS|Solyc02g091550.1.1

MGRGKIVIRRIDNSTSRQVTFSKRRNGLLKKAKELAILCDAEVGLIIFSSTGKLYEFSNTSMKSVIERYNKTKDDCQQLHNPVSELKLWQREAEILRQQL

QDLQDNHRQLLGEELSGLGVKELTNLENQLEMSLKGIRMKKEQILKDEIQELTRKGSIIHQENMELYKKVNLIRQENAELYKKAYGARDANAVNGNINYP

YRFTVSREVQAPIHLQLSQPEPQYFEMQAGTSDSR*

>Solyc03g006830.2.1|Solanum_lycopersicum|MIKC_MADS|Solyc03g006830.2.1

MKRIENSTSRQVTFSKRRNGLTKKAYELSVLCDAEVAFIIFSHKGRLYEFASSNMQKIIERYRGRARETTTVDKSTELEHYMENLKHETANMAKKIEILE

ISKRKLMGQGLGSCSMDELEDIDSQLERTLKIIRARKTQLFKEEIESLKAKERLLLQQNASLREKCGLRPMLSESASAPEPIPAPPSTPPAQSKERGNCS

QSTKSWEVETELFIGLPQTRCL*

>Solyc03g019710.2.1|Solanum_lycopersicum|MIKC_MADS|Solyc03g019710.2.1

MGRGKVELKRIENQTNRQVTFSKRRNGLLKKAYELSILCDAEVALLLFSPSGKAYHFASHDIERTILRYKNEVGLSKNSDQGPRAMEVWRTKIDDMTRTI

HELEARDKHFAGEELSNLGMKELKQLERQLRVGVERIRSKKHKILHEENIHLQKQVKLYEVEGSSRILDTNPRMRII*

>Solyc03g114830.2.1|Solanum_lycopersicum|MIKC_MADS|Solyc03g114830.2.1

MGRGRVQLKRIENKINRQVTFSKRRSGLLKKAHEISVLCDAEVGLIVFSTKGKLFEYSTDSCMERILERYERYSYAERQLNATDIITPGSWTLEHAKLKA

RLEVLQRNQKHYAGEELDTLSMKELQNLEHQLDSALKHIRSRKNQLMHESISELQKKDKALQEQNNNLSKQVKEREKEMAQQTPWEQQSHDHLNSSSFVL

PHPFNNLHIGEAYPNAGDNGEVEGSSRQQQQNSASVMPPWMLRHLNG*

>Solyc03g114840.2.1|Solanum_lycopersicum|MIKC_MADS|Solyc03g114840.2.1

MGRGRVELKRIENKINRQVTFAKRRNGLLKKAYELSVLCDAEVALIIFSNRGKLYEFCSTSSMVKTIEKYQRCSYATLEANQSVTDTQNNYHEYLRLKAR

VELLQRSQRNFLGEDLGTLSSKDLEQLENQLESSLKQIRSRKTQFMLDQLADLQQKEQMLAESNRLLRRKLEESVAGFPLRLCWEDGGDHQLMHQQNRLP

NTEGFFQPLGLHSSSPHFGYNPVNTDEVNAAATAHNMNGFIHGWML*

>Solyc04g005320.2.1|Solanum_lycopersicum|MIKC_MADS|Solyc04g005320.2.1

MGRGKVELKRIENKINRQVTFAKRRNGLLKKAYELSILCEAEVALIIFSNRGKLYEFCSTSSMSDTLERYHRCSYGDLETGQSSKDSQNNYQEYMKLKAR

VEVLQQSQRHILGEDLGQLNTKDLEQLERQLDSSLRLIRSRRTQNMLDQLSDLQQKEQSLLEINRSLKTKLEENSVAHWHITGEQNVQFRQQPAQSEGFF

QPLQCNTNIVPNRYNVAPLDSIEPSTQNATGILPGWML*

>Solyc04g078300.2.1|Solanum_lycopersicum|MIKC_MADS|Solyc04g078300.2.1

MGRVKLQIKKIENTTNRQVTFSKRRNGLIKKAYELSVLCDVDVALIMFSPSGRVSTFSGNKSIEDIMARYVNLPEHDRGRLHNQEHLQRAIAKLKCEADR

TYQAPSSPSSVDSHIEEFQQEIIRYKTQVEDMERRLRMYEGGFCEITTVCEAQYREEILQETLKQVQARKQVLEENYHSPQTQNTTQPQMDFSGQNVNMV

NNVATSDAIANSTFMDWVPHSQRDPHVQILNFLDSSGLLPFRDEADQHMLPPSLNQLHGVNVPAGTDHLSSNSRFDQNNPPRPSSFDGIIDVNNAPWPPL

YTTGDDPFPVSQPRERAILELFLSQLTPVNQDHI*

>Solyc04g081000.2.1|Solanum_lycopersicum|MIKC_MADS|Solyc04g081000.2.1

MARGKIQIKKIENQTNRQVTYSKRRNGLFKKANELTVLCDAKVSIVMISSTGKLHEFISPSITTKQLFDLYQKTIGVDIWTTHYEKMQEQLRKLKDVNRN

LRKEIRQRMGESLNDLNYEQLEELMENVDNSLKLIRERKFKVIGNQIETYRKKVRNVEEINRNLLLEFDARQEDPYGGLVEHDGDYNSVLGFPTGGPRIL

DLRLQPNNNYHNHLHSGGGSDITTFALG*

>Solyc05g012020.2.1|Solanum_lycopersicum|MIKC_MADS|Solyc05g012020.2.1

MGRGKVELKRIENKINRQVTFAKRRNGLLKKAYELSILCDAEIALIIFSSRGKLYEFCSNSSMSKTLERYHRYNYGTLEGTQTSSDSQNNYQEYLKLKTR

VEMLQQSQRHLLGEDLGQLGTKDLEQLERQLDSSLRQIRSTKTQHILDQLAELQQKEQSLTEMNKSLRIKLEELGVTFQTSWHCGEQSVQYRHEQPSHHE

GFFQHVNCNNTLPISYGYDNVQPENAAPSTHDATGVVPGWML*

>Solyc05g015750.2.1|Solanum_lycopersicum|MIKC_MADS|Solyc05g015750.2.1

MGRGRVELKRIENKINRQVTFAKRRNGLLKKAYELSVLCDAEVALIIFSNRGKLYEFCSSSSMLKTLERYQKCNYGAPEPNISTREALEISSQQEYLKLK

GRYEALQRSQRNLLGEDLGPLNSKELESLERQLDMSLKQIRSTRTQLMLDQLTDYQRKEHALNEANRTLKQRLMEGSQLNLQWQPNAQDVGYGRQTTQTQ

GDGFFHPLDCEPTLQIGYQNDPITVGGAGPSVNNYMAGWLP*

>Solyc05g056620.1.1|Solanum_lycopersicum|MIKC_MADS|Solyc05g056620.1.1

MGRGKVELRKIENKINRQVTFSKRRGGLVKKAHEISVLCDAEVALIVFSQKGKIFEYSSDSCMEQILERYERYSYAERRLLANNSESPVQENWSLEYTKL

KARIDLLQRNHKHYMGEDLDSMSLKDLQNLEQQLDSALKLIRSRKNQLMHESISELQKKERAILEENNMLTKKIKEKDKIVEQQGEWHQQTNQVSTSTSF

LLQPHQCLNMGGNYQDEVAEARRNNELDLNLDSLYPLYNMNKHL*

>Solyc06g059970.2.1|Solanum_lycopersicum|MIKC_MADS|Solyc06g059970.2.1

MGRGKIEIKRIENTNNRQVTYSKRRNGIIKKAKEITVLCEAKVSLIIFASSGKMHEYCSPSTTISDMLDGYQKASGRRLWDAKHENLSNEIDRIKKENDS

MQVKLRHLKGEDINQLTHKELIIMEEALQNGLSSISAKQSEILRMVRKNDQILEEENKQLQYALHQKEMGAIGGSGNMRGIHEEVYHQRERDYEYQMPFG

LRVQPMQPNLHERM*

>Solyc06g069430.2.1|Solanum_lycopersicum|MIKC_MADS|Solyc06g069430.2.1

MGRGRVQLKRIENKINRQVTFSKRRSGLLKKAHEISVLCDAEVGLIVFSTKGKLFEYANDSCMERILERYERYSFAEKQLVPTDHTSPVSWTLEHAKLKA

RLEVLQRNQKHYVGEDLESLSMKELQNLEHQLDSALKHIRSRKNQLMHESISVLQKKDRALQEQNNQLSKKVKEREKEVAQQNQWEINSSSFVLPQQLDS

PHLGEAYQSTNVIDNGEVEGGSSSQQQGAANNTVMPQWMLRHLNN*

>Solyc07g055920.2.1|Solanum_lycopersicum|MIKC_MADS|Solyc07g055920.2.1

MVFPINQELLVDESSSQLRKTSGGTGGGGRGKIEIKRIENTTNRQVTFCKRRNGLLKKAYELSVLCDAEVSLIVFSSRGRLYEYANNSVRATIDRYKKHH

ADSTSTGSVSEANTQYYQQEASKLRRQIRDIQTYNRQIVGEALGSLSPRDLKNLEGKLEKAIGRVRSKKNELLFSEIELMQKREIELQNANMYLRIAEVE

RAQEQMNLMPGGGGGGGGGGGGGSDHQYHHQPNYEDARNNFLPVNLLEPNPHYSRRDNGDQTPLQLV*

>Solyc08g067230.2.1|Solanum_lycopersicum|MIKC_MADS|Solyc08g067230.2.1

MGRGKIEIKRIENSSNRQVTYSKRRNGILKKAKEISVLCDAHVSVIIFATSGKMHEFSSTSLVDILDQYHKLTGRRLWDAKHENLDNEINKVKKDNDNMQ

IELRHLKGEDISSLNYRELMILEDALENGLTGIREKQNEFMRMMRKKTQNMEQEQDQLNCQLRQLEIASMNRNMGEIGEVFEQTRENHDYGQMPFAFRVQ

PMQPNLHQRF*

>Solyc08g080100.2.1|Solanum_lycopersicum|MIKC_MADS|Solyc08g080100.2.1

MVRGKTELKRIENATSRQVTFSKRRSGLLKKAFELSVLCDAEVALIVFSPKGKLYEFSSSSTNKTIERYQKNEKSLGRLNRKLTDQLTTEHLKEEVATMT

RKLEFLEDSKRKLLGHGLESSTFDELQKVEEQLEKSLSNIRARKNLLFKEQIAQLKEEEKILLKENVDLKKKCQVLPLTLTPVPLVEKDVERQIMEVETE

LFIGLPETRKSSYCPNLNTLPTLL*

>Solyc10g080030.1.1|Solanum_lycopersicum|MIKC_MADS|Solyc10g080030.1.1

MGRGKIEMKKIENISSRQVTFSKRRAGLFKKAEELSVLCDAEIGVIVFSNTDRLYKFASSKSSMEKIVERYNSSSHSFEHPMIENVVEPELNSLKAEVAK

LRKATGRMMGKELDGLDFKELQQLEHQLTEGILSVKNKKEQVLLELLEKSNLQIEELGHKSCNHYPENYEAARKISGGNTTVICDFKSVEEENSDTSLSL

GLSVATSQKKKNPQIECTSNDSENLMILD*

>Solyc11g005120.1.1|Solanum_lycopersicum|MIKC_MADS|Solyc11g005120.1.1

MGRGKIEVKRIENKTSRQVTFSKRRAGLLKKTHELSVLCDAQIGLIIFSTKGKLFEYTTQPHSMGEIINKYLQTTGASLPIHDHRVEQYDEITKMKRETL

NLELSLQRYKGDELNSAQYDELNELEKQLENSINKIRARKLELLQQQMENLKRTEKMLEKENHDMCQWLMKYEMYKQQPVAMMEQQEEAAITELNLLGEQ

PLLSQFSFFGDQHQLGTTSNSSAYHLQTSHPFTPSTYD*

>Solyc11g010570.1.1|Solanum_lycopersicum|MIKC_MADS|Solyc11g010570.1.1

MAREKIQIKKIDNSTARQVTFSKRRRGLFKKAEELSVLCDADVALIIFSSTGKLFDYSSSSMKQILERRDLHSKNLEKLDQPSLELQLVENSNYSRLSKE

ISEKSHRLRQMRGEELQGLNIEELQQLERSLETGLSRVIERKGDKIMREINQLQQKGMHLMEENEKLRQQVMEISNNNNNNNNGYREAGVVIFEPENGFN

NNNNEDGQSSESVTNPCNSIDPPPQDDDSSDTSLKLGLATLLRLKRSKARCGYFCMLLEEGEKKK*

>Solyc11g028020.1.1|Solanum_lycopersicum|MIKC_MADS|Solyc11g028020.1.1

MGRGKIEIKRIENNTNRQVTFCKRRNGLLKKAYELSVLCDAEIALIVFSTRGRLYEYSNNNVKATIERYKKATAETSSAYTTQELNAQFYQQESKKLRQQ

IQMMQNTNRHLVGEGLSSLNVRELKQLENRLERGITRIRSKKHEAILAETEDLHKREIQLEQENAFLRSKIAENERLQELSMMPSGGEEYNAFQQYLARN

MLQLNMMETALPSYDPLSPDHKR*

>Solyc11g032100.1.1|Solanum_lycopersicum|MIKC_MADS|Solyc11g032100.1.1

MARGKVQMKRIENPVHRQVTFCKRRAGLLKKAKELSVLCDAEIGLFIFSAHGKLYELATKGSMQGLIERYIKSTKGVEVAEEAKDTQPLDPKEEINMLKN

EIDVLQKGLSYMYGGGAGTMTLDELHSLEKYLEIWMYHIRSAKMDIMFQEIQLLKNKEGILEAANKYLQDKIDEQYTVTNMTQNLTDFQCPLTVQNEIFQ

F*

>Solyc12g038510.1.1|Solanum_lycopersicum|MIKC_MADS|Solyc12g038510.1.1

MGRGRVELKRIENKINRQVTFAKRRNGLLKKAYELSILCDAEVALIIFSSRGKLYEFSSASSMMTTLEKYQQCSYASLDPMLPVSDTQMNYNEYVRLKAR

VELLQRSQRHILGEDLGTLNSKELEQLEHQLDASLKKVRSKKTQSMLDQLADLQEKEQMLEEANKQLKNKLEESAARIPLGLSWGNNGGQTMEYNRLPPQ

TTAQPFFQPLRLNSSSPQFGYNPNMGANDHEVNAATTAHNINGFIPGWML*

>Solyc12g056460.1.1|Solanum_lycopersicum|MIKC_MADS|Solyc12g056460.1.1

MVRGITEMKRIENTTSRQVTFSKRRGGLLKKAFELSVLCDAEVSLIIFSQKGKLFEFSSSSTNKTIERYQKNDKNLGHENILLEQTTEHLKGEVMSMTRN

LEVLEISKRRLLGEDLESCSIDELEKVEGQLDQSLRNIRAKKNQLFKEQISLLKDEEKVLMNKNAELREKYEARSLPLFIDRREDESPQTQNMEVDTQLF

IGLPER*

>Solyc12g087830.1.1|Solanum_lycopersicum|MIKC_MADS|Solyc12g087830.1.1

MGRRKVEIKRIQDKNCRQVAFCKRRKGLLKKAKEISILCDVDVAVVIISNRGRLHEFSSNNSMTAMLRRYESHVGAEKEINAEIQVAEVSGFTTMGELLQ

TTERQLEETNADGLTLTDLIHLENELQTALIHLRARKTHLMLESAKVLHEKEKLLLEEKKHLEDNIASIKKNTKVNEMSDLPAPHMICGQQKVTLNFF*

>Solyc00g179240.1.1|Solanum_lycopersicum|M-type_MADS|Solyc00g179240.1.1

MRRIENATSRQVTFSKRRNGLLKKAFELSVLCDAEVGLIIFSPRGKLYEFASSRX

>Solyc01g010300.1.1|Solanum_lycopersicum|M-type_MADS|Solyc01g010300.1.1

MAQTKILRRQKIKRKMITSKVKRMVLFKKLWANFVKEAHDLYMTTGAHVTIVAFSPTGKAYAYDSSNNFDTIERFLNDSKASAIEGGH*

>Solyc01g060300.1.1|Solanum_lycopersicum|M-type_MADS|Solyc01g060300.1.1

MRKPNGRKKIEIAKIQNQTNLQVTLSKRRAGLFKKASELSTLCGANVAIVAFSPSNKVYACGHPSVESIVDKFIGENPPPETDDPNPIIVMTADTIPKGS

PFTSMQEFSLGIDFGKLCQITCVPQLIESCPNSSILQIWTDYHLGSTNELAHQALLDLGGYRLVILPLHRAGTVRCSVKPASRSPFHFRNPSAHSFDWIA

RPNSMLGQGRP*

>Solyc01g060310.1.1|Solanum_lycopersicum|M-type_MADS|Solyc01g060310.1.1

MRKPNGRKKIEIAKIQNQTNLQVTFSKRRAGLFKKASELSTLCGANVAIVSFSPSNKVYACGHPSVESIVDKFIGENTPPETDDLNPIIVMGLSFLETDY

HLGPTTELAQVCVPAARQMSGARSIIVVSQCVLERLIAT*

>Solyc01g066500.1.1|Solanum_lycopersicum|M-type_MADS|Solyc01g066500.1.1

MTKGTGKKKIDIKKLASNSSRKVTFSKRRTGLFKKAEELATKFGARIALLVFSPAGRLYTSGDVSIFENNGFPSKPHLSTETASNSYVKPSTLRLKNLNV

EGCSEIFSPDGVHYSAGEIPIVEDNLQNNGFSSNSHLSTDFSSNFYEDWSTLWLKNFKGNNEIFSPPGGFYNSGEFPIVADELKNNEFPSNSDLSIEISS

NSYAKPSTLHLKNFNAEGCSEIFSTDGRFYSSNEFPIINDTLKNNGFSSNSDLSTDLGSNLYEYWSMENFNVEECSSIEELMLLKEKLEEKRDEIILKME

AEFIDSLLV*

>Solyc01g066730.2.1|Solanum_lycopersicum|M-type_MADS|Solyc01g066730.2.1

MGKGKQKIEIKKITKESARKVAFSKRRKGLFKKAVQLESKTGAKVAILVFSSSGKPYTCGDVETLCGISDSFNLQTHSESNSIWDSFNLQTHSESNGIWD

SFNLERPCESNGMWDSFNIIEGPCSSSGKNGMWDSFNLERPCSSSGQSGMWDSFNLGRPCESNGMWDSFNIIEGPCSSSGQNGMWDSFNLERPCSSSGQS

GMWDSFNLERHCSSSGIPSGSNGIWDSFNVETHCSSSESCGMSDSFNVETHCSSSGQSGTWDSFNVEACHNVNELLLLKAHLESTREKLLESQFLDSLWS

*

>Solyc01g097850.1.1|Solanum_lycopersicum|M-type_MADS|Solyc01g097850.1.1

MENKKSRGRQKIPMKKIEKLGDLYASFSKRRLSLYKKASDLVFECDVDIGMIYFSPKGNPYSFFHPNVNTVVSRFQNPDMEFSESDLLITTDNQVKVKEL

KSRLDELDIIEDIAIATKKSYDEVIKARKRGWWESIEQLNEPQVTKFEAWMDTIIFNMQNRLNEMENGASSS*

>Solyc01g098050.1.1|Solanum_lycopersicum|M-type_MADS|Solyc01g098050.1.1

MGTGKKKIEIEKIIKETSRMVTFSKRRKGLFKKAKQFESMTGSRVASIVLSPTGRPYTCGDVDYAIRTHFSNSGRCMKLLITDIMNSHDSNSSSNVVVYG

ETSRSKFSSAPKKNSLHNWVKRIDVEQCQNLNWLLMLKQQLEGTKEKIGEDVESFKAFFV*

>Solyc01g098060.1.1|Solanum_lycopersicum|M-type_MADS|Solyc01g098060.1.1

MGTRKRRTEIEKLTKQSDRLTTFSKRKKGIFKKAELLESLTSSRVTSVVFSPSGIPYTYGNVNSVIKKHFPSCNRSEISTTVMNSHHDVSGESSGSKSLS

IPKENGLRRWVEDIDVEGCQNLNQLFMLKEQLEGTREKIISSDPESFEALFM*

>Solyc01g098070.1.1|Solanum_lycopersicum|M-type_MADS|Solyc01g098070.1.1

MGTGKKKIEIEKITKQTARMVAFSKRRKGLFRKAEELESMSSSRVTSVVISPFGKPYTYGNVNSVIKKYFSICIRPEISTPVMNSHPSSSNVSGESLGSK

SSSTPNGNALCNWVEGIDVEECQNLNQLLMLKKQLEGTREKIVSKESESFQALFI*

>Solyc01g102260.2.1|Solanum_lycopersicum|M-type_MADS|Solyc01g102260.2.1

MTRKKVKLAFITNDSARKATFKKRKKGLMKKVSELSTLCGIDACAIIYSPYDTSPEVWPNTMGAQRVLAEFKRMPEMEQSKKMVNQESFIRQRIAKASEQ

LKKQSKENREKEMTEVMYQGLTGKGLQNLNLGDLNDLGWVIDQNLKEVYKRIEAVKKGASTSSSSSVAAAAVAAAAVASQAVAPPMEQKPAVVELGLDSM

QRTQTEWFTDWMNSNASDQHIGYGHADEMILPNFNDNHNANVWPNNFYP*

>Solyc01g103550.1.1|Solanum_lycopersicum|M-type_MADS|Solyc01g103550.1.1

MGRAKLKMELISKEKSRNATFKKRKEGLLKKLYEFTTLCNVNGLMIMYGPKQGNGSECRPEIWTNSSGSSSSTNSKSLQQQQEEIENLIDEYKKENSLQS

GSSKTFGLSDYFVDRNKRVEEEFIKLRKMNMEKKYPCWLEFMDQLSEFKLRDFLTLLDDRVENVKARIHLLKGNFSGLMGGEMIDLGGGNQWTHYNDNVM

VQGGGMEYGDYNQLQAPIYHQEMRMVMMNENDWPQYNNGASSSSSAGNGSNNMMCALMKYETMMPSNNHLAYSPYVAPTILQQTPCMMMPQHSWRDNDRD

DKAKFSPYMTK*

>Solyc01g103870.1.1|Solanum_lycopersicum|M-type_MADS|Solyc01g103870.1.1

MADEDQSKKGEEDQRKKKTNNSKSYQVRKECIKRKSMELATLCDIKVCTVITGPNRELQTWPDNLNACKEVLDIYSQNLKPEKKHKQEDKDLPTLVESKL

AAVNRRICFLENKNVADKGKGKRIE*

>Solyc01g106170.2.1|Solanum_lycopersicum|M-type_MADS|Solyc01g106170.2.1

MIFLDRCQRVRLLWCTRASRGSKISDYFFTKPIFLLSLSLSICRISLLSKSLKLSRLIKEMGRGKIVIQRIDNTTSRQVTFSKRRNGLLKKAKELAILCD

AQVGLIIFSSTGKLYEFANNSMKSTIDRYNKIKEENNNMNPMSEVKVT*

>Solyc01g106700.2.1|Solanum_lycopersicum|M-type_MADS|Solyc01g106700.2.1

MTTINAVKKTQGRRKIAIKPIDNQNSRHVTFSKRRLGLFKKASELCILSGAEIAILVQSLKRQRLFTFGHPSPDAVIDRYLTGKSVSPGDGDQFNLQQSN

QYYSQICRDLELEKQKKENIEESKMVNNGGFWWNEPIDDMGIEGLEEFMSALEELKKKVTMRADELSMINGSSSNSTMKIARFGAEDQYFNESIDYCSSI

VPFDFNQPGDRQF*

>Solyc01g106710.1.1|Solanum_lycopersicum|M-type_MADS|Solyc01g106710.1.1

MAKKPSMGRQKIKIAKIEVKNHLQVTFSKRRSGLFKKASELCTLCGVEIAIIVFSPARKVFSFGHPNVESIIDRFLTRANSNNNPIANNSIQLVEAHRNA

SVRGLNLQLTQILGEVEIEKKRGESLDQMRKTSQSQYWWEAPINQLDLQELEQLKDSMEVLKKNVTNQANKFMVNETPNPSFFGVNANGIFDNYDIKPPR

NMNASNNLHNHNLGFDSSTFF*

>Solyc01g106720.1.1|Solanum_lycopersicum|M-type_MADS|Solyc01g106720.1.1

MAKKMSKGRQKIQMTKMSKESNLLVTFSKRRSGLFKKASELCTLCGVEIAIVVFSPGHKVFSFGHPNVDSIVNRFLTRNPTSSSSTTCQLVEAHRNANVR

ELNAQLTEILNQLEFEKKREVEIEKIQKGKIGKNWWEGPLNELEHGELEQLKLGMEELKKNVTKQMQKIIFEASNAPTFFLGGSSSSNGDVKNMKGLGLS

MATNGGHTSFP*

>Solyc01g106730.1.1|Solanum_lycopersicum|M-type_MADS|Solyc01g106730.1.1

MARRVSKGRQKVEMVKMKNASNLQVTFSKRRAGLFKKASELCTLCGAEIAIVVFSPGDKVFSFGHPNVETLVDRFLGRDLPLPNNDVHNHLIVAHREAGI

RELNTKLMNLEGVLHMEKKRGESLQEIRRRANGQWWESPIEELNLFQLQHLKEALENLEQKVEKVAHQQQMLNNIAFPFRTLGSALTPPNCARETSSYGL

NVGAPFANRDIGSTSSIVPNH*

>Solyc02g032000.1.1|Solanum_lycopersicum|M-type_MADS|Solyc02g032000.1.1

MENETKKGKKKIEMKLIESERARAVSFSKRKKTLFEDAKKFATQTGADVAVMLFSPGGKPYSCGSTSVEDIIENFLKMKVADPRRHYAEGESKVLRN*

>Solyc03g007020.1.1|Solanum_lycopersicum|M-type_MADS|Solyc03g007020.1.1

MGSKIRGLTLSNHETFSQVFLPINLFFRKNILHQKLSHGKKKTLGRQKISMVKIENEDARYTTFSKRRSTLYKKASELVGKYDVDVGITLFSPTDKPYSF

FHPTVDVVVDRILNPNTQPSEDNSIAIASYRNKVKDQKVELDELDIIERGISNSTFGTKETNMKNIWESFMKFNEDEVNELELWLNSIDFDLKNYLSQLE

NNVSSSTQAPPKNVG*

>Solyc03g034260.1.1|Solanum_lycopersicum|M-type_MADS|Solyc03g034260.1.1

MDSKKTRVRQTFPISKIENQRVSDVTFFNRRSSLYRMANELVDFCDVDIGIVLFSPSNHPFSFFHPTSEAVIERFLNPDSQLSEKTRLDAEQARNKVNQL

NNRLDAMEKRIEQIEEIEHAQTLLQLSQTEENGERSKWKSIDQLNANEIPTFEAWLRTTVSKMNYRLEKLENEASSLKNARGTS*

>Solyc03g062820.1.1|Solanum_lycopersicum|M-type_MADS|Solyc03g062820.1.1

MGTGKKKIEMEKITKKSCRMVTFSKRRNGLFKKNEELESLTGSQVSSVVLSPAGRIYTYGDVNTAINMHFSKIDCMRQSDSDVVVSSGSSELRSSSKSLR

DWLEDIDVEQCQNLNQLLLLKEQLEGTKKKIVSIEDSKSFQALFM*

>Solyc03g115910.1.1|Solanum_lycopersicum|M-type_MADS|Solyc03g115910.1.1

MASFACVFCPLEFHISEPLPAFKLGSCPTSNPKFQSAPICFFHRMGRVRLSVKRLESHSNRQSTYCKRRCGILKKAQEISVLCDIDIILLLFSPTGKPTL

FQGGQSNFDEIIAKFAQLTPQERAKRKLESLEVSLIFITTMCSPLAMLCCYFIIQVKVITMLMVTLQTLRKTFKKLDNDIGVPEFLDASDPSVEELHSQV

KLLQSRLTDVEMRLNWWSNPDNINKVEDFALMECALRESLNAVHVRKLQKTTHLHLLMNSELDGNTHQWHPENKILRMPFPQTPNILPQENMGYFGDNSV

AESSHVQGSGEVDQARQDTTAMLDNGVLNDLTSIACLRQQLSEQYSYNPNEDLDLLERNMLDPQSDANLKGYLMDYAFQRNFNLTRSVDSVNYSAVDAVA

VPDFDEKSYAQPATSSD*

>Solyc03g119680.1.1|Solanum_lycopersicum|M-type_MADS|Solyc03g119680.1.1

MENETKKGKQKIEMKLIESEKARTVSFSKRKRTLFQDVDKFAAQTGADVGVMLFSPSGKPYSHGSTSIEEIIDQYLKVKLEDHQRDHAEGKMNGFEVLGA

LHKELQAWNEKEKNRKLMYKIMHSGSEAPPDKHMEEQKLALKLRVEKIKKETQAAILVEHLKFDLNVAPEPEEDESS*

>Solyc04g025030.1.1|Solanum_lycopersicum|M-type_MADS|Solyc04g025030.1.1

MTTRINKGRQRVDMVKMKNARNLQVTFSTRLAGLFKKSNELCMLFNAEIFIVVFSQGDKGVLCFDHPSVNPLAEGFFEWNLPQPHINVHNQHIVARKEGG

TRDLSTKLMSLEAILEKEKNCGQILIEIRKRANSL*

>Solyc04g025050.1.1|Solanum_lycopersicum|M-type_MADS|Solyc04g025050.1.1

MKNTRNLRVTFSKHRVGLFKKASKLCMLCGAEISIVVFSPNGKVFSFGHLSMDTLI*

>Solyc04g025110.1.1|Solanum_lycopersicum|M-type_MADS|Solyc04g025110.1.1

MATRINKGSQRVDMVKMKNARNLQVTFSKRLAGLLKKALELCMLCGAKIIIVAFSPSDNGDFSFGPTSISPSVERFLGRKFPQPNNDVHNQQIVALIEGG

ICELNTKLRNLEGILEMEINRGPSLGELGR*

>Solyc04g025970.1.1|Solanum_lycopersicum|M-type_MADS|Solyc04g025970.1.1

MEGKKKAGCQRIPLEKIEKKVARYASFFKRRLCLYKKASELIQERDVDIGVFISSQTGKPYSFVHQTANVVINHFKSPTTIDLGAQFAGAEARNNVIQMN

DMLNDFDAREKVTKNHI*

>Solyc04g047870.1.1|Solanum_lycopersicum|M-type_MADS|Solyc04g047870.1.1

MSRRIFKGRQRVDMVKITNGRNLGVTFSKRRAGLYKKACELCMLCGAEIAIVIFSPEGKIFSFGHPSVETLVERFLGRNLPPPNNDVHNQQIVAHREAGI

RELNTRLMNVEGALQMEKNRGESLQEIRKKADGVWWQSPIKELNLFHLQHLKRALEILKQKVVKEAQMVNNNAFPFQTLGSAWSPPNYTS*

>Solyc04g056550.1.1|Solanum_lycopersicum|M-type_MADS|Solyc04g056550.1.1

MERKKTKGRQKIPMQKIENKNALLTTFSKRRKGLFKKASEVVTECDVDIGIMMISPSGKPHSFFHPTADAIVSRFQNPDMQLSEGIRLDATTARNRVNQL

KTRLEELDAIEDALFAQTIFYDQMAETQQKSSWESIEQLDADELIINEAWLRDTNFKICDRLSQLEIGASSSLGCEFLEYEV*

>Solyc04g056740.1.1|Solanum_lycopersicum|M-type_MADS|Solyc04g056740.1.1

MERKKTKGRQKIPIKKIENEDALLTTFSKRREGLYKKASELVRECDVDIGIMMISPAGKPHSFFHPTLDAIVTRFQNPDMQLSQGILLDTITARNKVNEL

KNRLEELDVVEDATIAQTTFYDQMAEIRQKGWWESIEQLNADEVTIFNAWLSDTCSKMCHRLKQLENGASSSLGRGSFGV*

>Solyc04g064860.1.1|Solanum_lycopersicum|M-type_MADS|Solyc04g064860.1.1

MNINNNTNAAANSAAVAVKKTQGRRKIAIKPIANQNSRHVTFSKRRLGLFKKASELCILTGAEIAIMVQSLKRQRLFTFGHPSADAVINRYLTGKSEEQK

PAVDDQLNYVQQSNEYYSQICRELELEKKVKEEIVIDESKMVNGGSSSNNGGGGFWWNESIDEMGIEELEKFMFALEELKKKVNMRCDELSMINGSSSMA

AAATASTSSMNQAIDYCASIVPFDFNYPGNAQF*

>Solyc04g076680.2.1|Solanum_lycopersicum|M-type_MADS|Solyc04g076680.2.1

MGRGKIVIRRIDNSTSRQVTFSKRRNGLLKKAKELAILCDAEAGVIIFSSTGKLYEYSNTSKK*

>Solyc05g013370.1.1|Solanum_lycopersicum|M-type_MADS|Solyc05g013370.1.1

MEKKNKGNMLNYKKKKETIKKKTRELSILCDVKACVILVDPNGKVDTWPENPTDFNPIIQSYKENLCHGKRKRIDDDGCFEKKSKKNHALFCDDDENQWL

NDVFRESNESLLVKLNSKLEAVDRRIEFLKMMNYGNGVVGGSSSSAKESLLANQETHNRLENSNAYNQETEIAMAAEFWVIGGDESANDRGKEIDFLRDN

ATVNNLNNVQNFGYDDHLWPVIAASEFSTCIN*

>Solyc05g015730.1.1|Solanum_lycopersicum|M-type_MADS|Solyc05g015730.1.1

MGRKKVEIKRIEDKSSRQATFSKRRNGLMKKAKQLSVLCDVDVAVLVFSSRGRLFEFSSTNRFFFYLFLANSLLDPYIVL*

>Solyc05g051830.2.1|Solanum_lycopersicum|M-type_MADS|Solyc05g051830.2.1

MGRSKLPLLKIESLTNRQVTFSKRRNGILKKVYELSVLCDVDVGIIMFSPSGRLTHYSRKRRIEDILSELISLPDSERGFYINNKESVLWNLRKIEIEDK

FCDIERINPAYVNANDTTKKIQDEINGLHCKLDEAEGLLRIFEPDTQRITSLHELDLCEKRLQVALNQVRQRMEQLSSNNTPSYEDNMAQINELLQHIDN

TQVHEKPPYDLWLELEDYNHENNNINSPLYTASETSSISQSSMNLPSSTTYDTMSQTSLSGETYQNNNNFKQSQHSTRTLPNLTLQTSFKFAKPEMSQTS

IEGSFSCLTDENLKKSICSNRVFPAITPLQTSFSFAKAEMETPTSALRPLAPYLQAEATTSSCTNQEGNNEMSWFQPKVKKSKQYHSID*

>Solyc06g033820.1.1|Solanum_lycopersicum|M-type_MADS|Solyc06g033820.1.1

MDRNKIMRKKIEDPVSRQQFYLKCKDIIVKKSDELGFLCNSNIALLMVSQNGEVTSYSRGESFEDIMVKAMNQPVQLNRRSIPNPDEEHLMQSLVQSKSE

RGMIEKIAMYDTLLFSSSFILIYFFVVVLFYMIEKFLLFLLGVFTMLMIGQRASVLSSYEPQVENINTTEEADAYKEYILGAIERVQRSKVSISKS*

>Solyc06g033830.1.1|Solanum_lycopersicum|M-type_MADS|Solyc06g033830.1.1

MGRNKVVMKKIEDPESRKHFYSKRKDGLVKKSNELGVVCHTNIALLMFSPTGEVTTYSRAKSSYEPQVENINNVEEADAYKGYLLGAMERVQRSKEPLGD

ERFDELNWITMER*

>Solyc06g035570.1.1|Solanum_lycopersicum|M-type_MADS|Solyc06g035570.1.1

MKRIENATSRQVTFSKRRNGVIKKAYELSVLCDAQVALIIFSNKGRLFQFSSSW*

>Solyc06g048380.1.1|Solanum_lycopersicum|M-type_MADS|Solyc06g048380.1.1

MDRNKIMIKIIEDPISRQQFYSKCKDSIVKKSNELGLLCDTNIALLMVSPNGEVTSCSGGESFEDIMSNAMNQFDELNRHSYEPQAENINTVEEADAYEQ

YLLGAIGRIQLSKAKFLDNQEFLKRNENVAEPVRDGRPERNILDHNGCSCSRNYVILVYILKLSTMNNLHQASKQD*

>Solyc06g054680.1.1|Solanum_lycopersicum|M-type_MADS|Solyc06g054680.1.1

MERKKTKGRQKIPMKKIENKDSMFASFTKRREGLYKKSSKLATEYNVDIGIMMISATGKPHSFFHPTFDAVISRFQNHDMQFGERTNLEANDARNEVNQL

KTRLEELDVREDIAIAKKNSYEQMEETRQKGWWESTEQLNADEVFIFETWLNETSSNLHHRLNQLEIEASSSMRHESFGV*

>Solyc06g059780.1.1|Solanum_lycopersicum|M-type_MADS|Solyc06g059780.1.1

MERKKTKGRQKIPMKKIENKDSMFASFTKRREGLYRKASELATEYNVDIGIMMISSTGKPHSFFHPTFDAVISRFQNPDMQFGESTNLEANAARNEVNQL

KTKLEELDVREDIAIAKKNSYEQMEETRQKDWWESTEQLNADQVFIFETWLNETSSNLHHRLNQLEIEASSSMRHESFGV*

>Solyc06g071300.1.1|Solanum_lycopersicum|M-type_MADS|Solyc06g071300.1.1

MAEEDEIKRRTNNPKSYQVRKECIKRKSMELATLCDIKVCTVITGPNGELQTWPDDFDACKQVLDLYSQNLKPEKKYKESSTPEPEPEPEPERGEEQGEK

DLLTLVESTLAAVNRRICILENKGKRKRIE*

>Solyc07g052700.2.1|Solanum_lycopersicum|M-type_MADS|Solyc07g052700.2.1

MKKIEDSTSRKQFYSNRKDSIVKKSNELAVVCGTDVGLLMFSPSGQLTTYSSKESIEDIMIEAMNKSVNPRPIPNLNEQLLMQSLKQSKSEGQMVGKIAI

AEAHEKKLNELKETLREAQQKIRYCNPQVENISSVQEAEAYEQFLRSNMEQIQQSKAKLLGVQGLVHRNEYPAVNTEDTAAAGTSSGWMF*

>Solyc09g061950.1.1|Solanum_lycopersicum|M-type_MADS|Solyc09g061950.1.1

MENENKKGKQKIEMKLIENERARMVSFSKRKKTLFEDAHKFATRTGADVGVMLFSPSGKPYSNDSATIADIIDRFLKVKQEDHKRDYAEGESNGFEALKD

LHKELQAWNDKEKKRKLMHKIMHPSLEIPSDKHMEEQKLALKLKVEKFKNEIQSAITTEHLKFDLNVVPDPEE*

>Solyc10g012180.1.1|Solanum_lycopersicum|M-type_MADS|Solyc10g012180.1.1

MESKNSNVDSAVAKDKRGEGGKISKMQKALFKKASDLSILCGIQVAIIILFINRQPIVFGKPDAESVINQFIEANHPTAPRFYMKMKKKEEENKEKGKSI

EDDIQSQDFESPYLGSLLKLYEGLTEFENQLTKEIDLTQLNQEIEKHEDPKLMNVASSSTLPTNFSP*

>Solyc10g012200.1.1|Solanum_lycopersicum|M-type_MADS|Solyc10g012200.1.1

MESKNSNVDSAVAKDKRAEGGKILKMQKALFKKASDLSILCGIQVAIIILFINRQPIVFGKPDAELVIHQFIEANHPTAPRFYMKMKKKEEENKKKGKSV

EDDIQSQDFESPYLESLLKLYEGLREFENQLTKEMDLKQLNQEIEKHKDPKLMNVASSSTLPTNFNL*

>Solyc10g012380.1.1|Solanum_lycopersicum|M-type_MADS|Solyc10g012380.1.1

MQKSLFKRANDLAILCGIHIAILIFSVGRQPIFFGMPDVETVVQKFMEANHPTAPRFYMKIKKTEEENKEKGKSVEDNIAHRQLEDFESPYLGSLLKLYQ

GLTEFEDLLNKEIDPTQLNQEIEKHEDPKIVSKMNVASSSYLPTDMLSP*

>Solyc10g012390.1.1|Solanum_lycopersicum|M-type_MADS|Solyc10g012390.1.1

MQKSLFKRANDLAILCGIHIAILIFSVGRQPIFFGMPDVETVVQKFMEANHPTAPRFYMKIKKTEEENKEKGKSVEDNIAHRQLEDFESPYLGSLLKLYQ

GLTEFEDLLNKEIDPTQLNQEIEKHEDPKIVSKMNVASSSYLPTDMLSP*

>Solyc10g017640.1.1|Solanum_lycopersicum|M-type_MADS|Solyc10g017640.1.1

MVRGKTEMRRIENATSRQVTFSKRRNGLLKKAFELSVLCDAQVGLVILSPRDKLYEFSTSR*

>Solyc10g018070.1.1|Solanum_lycopersicum|M-type_MADS|Solyc10g018070.1.1

MEHKKTAGRQKISLAKIENESARLTKFSKRRSGLYKKACELVRECDVDLGIVMSSLKGIPYSFSSPTSNVVIDRFINPTANLSSSDRLVAVETRKKLENG

ASSSSQIPSDDANISPNVF*

>Solyc10g018080.1.1|Solanum_lycopersicum|M-type_MADS|Solyc10g018080.1.1

MEHKKTAGCQKTSLAKIENESARLTTFSKRRSGLYKIACELVRECDVDLGIVMSSPKGIPYSFSSQTSNVVINHFINPTAKLSSSDHLVASEARKRVSQF

NDILNELDEREKIANEKLDQMNEARDLGWWESIDRLNVHDVMKLEAWLNLH*

>Solyc10g018110.1.1|Solanum_lycopersicum|M-type_MADS|Solyc10g018110.1.1

MEHKKTAGRQKISLAKIENESARLTTFSKRRSGLYKKACELVRECDVALGIVMSSPKGIPYSFSSPTSNVVIDRFINPTANLSSSDRLVAAEARKKVSQF

YDILNELDEREKIANEKLDRMNEARDLGWWESIDQLNVRDVKKLEAWLISGEFKLNEHLEQLENGASSSSQIPSDDANISPNVF*

>Solyc10g050900.1.1|Solanum_lycopersicum|M-type_MADS|Solyc10g050900.1.1

MENKKTKGRQKIPMKKIENEKALLSSFSKRRNGLFKAANNLVKKFDVDIGIIVFSPTGKPHSYFHPTVDAVISRFQNPDMQLSDETHLAMIFARNSVNQL

EKKLEELDIQEKIEIDRTNYLDQMTETRQKGWWESIEQLNEDEVSKFEEWLNVASFTMHYRLNQSIVSS*

>Solyc10g050940.1.1|Solanum_lycopersicum|M-type_MADS|Solyc10g050940.1.1

MENKKAKGRQKIPMKKMKNEKALLSSFSKRRDGLFKVANNLVKEFDVDIGIIVFAPTGKPYSFFHPTADAVISRFQNPDTQLSDETHLAMVFAQNSVNQL

EKKLEELDIKEKIESDRTNYLDQMTETRQKGWWESIEQLNEDEVSKFEAWLNVASFTMHYRLNQSIVSS*

>Solyc10g050950.1.1|Solanum_lycopersicum|M-type_MADS|Solyc10g050950.1.1

MENKKIKGRQKIPMKKIENEKALLSSFSKRRNGLFKATNNLIKEFDVDIGIIVFSPTSKPHSFFHPTTDAVISRFQNPDMQLSDETHLATVFARNSVNQL

EKKLEELDIKEKIGSDRTNYLDQMTETRQKGWWESIEQLNEDEVSKLEAWLDVASLLCTTV*

>Solyc11g020320.1.1|Solanum_lycopersicum|M-type_MADS|Solyc11g020320.1.1

MGRSKIKMELIEDHKKRKSTLVNRKAGLVKKISELSILCDIKASMIIYEGNYNYQIWPNDSNDVQDLINLYKNQSQDGRTKRGKTLSNFFKNDEKKNNEF

KVEKYPTWDSRFGYLSQIELQNLVGVVEKRIEKAKEKIELLKSMNDQDPNIGSSSFSHQQQIWNNNNLMNQTTQWPFSYVNPFTHYDNFFQANIPISGAN

SMGAGMDDGLLTIDDYQFNNTDYSTMIETENCLVKNGIGSSSIMHPMMNNGIDSSSTMEYPFIYNGYTHMPYGFQ*

>Solyc11g020620.1.1|Solanum_lycopersicum|M-type_MADS|Solyc11g020620.1.1

MGRSKIKVELIQDDKKRMKTLVTRKAGLFKKISELSILCDIKACMLIYDEGNNNNCEMWPNDPNELINLYKNQPFEGPTKRGKTLSGDEIKVEKDPDSRF

DYFENNEKKKADAIQVEKYPTWDSRFDYLSQKELQNLAGVLENRMENAKGRIELLKSMNGSCSLSHQQQIWDYNNLMNQTTTLSNNNLFQSNIPISSTVN

SMGAGMDYHFCNANYCKMNESENWYGVGSSLTMMQHNMANNGIGSSSTILHLMGDDYGIIDSSSTMMLPMGNNEIGSSSTMQQPMYQYPFMYNGSTHVIG

SSSTMQPPMGQYPFTNNDDSTGILVQVQQCSLWGIIMRLVQL*

>Solyc11g020660.1.1|Solanum_lycopersicum|M-type_MADS|Solyc11g020660.1.1

MNTSVKRKASLVKKISELSILCDIKACMIIYDEGNSNCEMWPNEVKELINVYKDQPFEGRTKRGKTLSNYFKDEIKVEKDLDSRFDYFENDEKKKADAIN

VEKYPTWDSRFDYLSQKEIQNLVGVISKRMENAKGRIELLKSMNGSCSLSHRQQIWDYNNLMNQTSPWPISDHVNSFNNFFQSNIGVNSMMESENWLAND

EIGSSLAMHPMGNNYGIIDSSTTMMQPMGNNEIGSSSTMQQPMFQYPFMYNGSTHVIGSSSTMQPPMGQYPFTNNDDSTGDIGSSSMQPIGNNNEIGPMD

NDSTYDSCTFYY*

>Solyc11g069770.1.1|Solanum_lycopersicum|M-type_MADS|Solyc11g069770.1.1

MGRDLIMARLGRRRIRIEKIENKKKRSVTFSKRRYGLFKKESELSMLCDSPNATVVFSPDDETCVYSIGYPCVNSVLDKFMDVNPPQNLDDTGSLSVHRR

NAIREGVLALMEIEEEFEEEKKREKSLDTGISYENLNSSVYQNFIEKIEIGYMEAEQLAIELKERNVPFPYSTFGDALAPK*

>Solyc12g005210.1.1|Solanum_lycopersicum|M-type_MADS|Solyc12g005210.1.1

MTMKGVRSTRNVDPRMKKFILDKRIEALFKQANDLSILCDIEVGVIVFGPGENNAVVWPSLAQASDRVKNYLARHRDCKGKEVVNVRQ*

>Solyc12g016150.1.1|Solanum_lycopersicum|M-type_MADS|Solyc12g016150.1.1

MATKRLRDTANYNENVRNSILDKRVTSLFKKAEELSIVCDVEVAIIIFRPGKIQPITWKSPSLAQDVLTRYLSFIEFKRLSKLVTHEDYLQKKVDKKEEQ

ISKLEKMNEMEESINDPWFIQTIATLGDVSGVESAQKEGKGVNVEDDGHSKDLD*

>Solyc12g016170.1.1|Solanum_lycopersicum|M-type_MADS|Solyc12g016170.1.1

MAIKRLRNTRNYSENVRNSILDRREKSLFKKAEELSILCDVEVAIVIFRPGKIQPITWKSASLAQDVLTRYLGFIEFKRLNKLVTHEDYLQKKIDKKEEQ

ISKLEKMNEAKEMEILFNQLVEGKSINELDAREMKGLLKVFAAKMAKLDERKKELNQTPNPPSNKENITLSGSPMEESFNGPWFIQTIATLGDGSDIEFS

PKEGNGVNVEDDGHSKDLD*

>Solyc12g016180.1.1|Solanum_lycopersicum|M-type_MADS|Solyc12g016180.1.1

MAIKRLRNTRNYSENVRNSILDRRERSLFKKAEELSILCDVEVAIVIFRPGKIQPITWKSASLDQDVLTRYLGFIEFKRLNKLVTHEYYLQKKIDKKEEQ

ISKLEKMNEAKEMEILFNQLVEGKSINELDAREMKGLLKVFAAKMAKLDERKKELNQTPNPPSNKENITLSGSPMEESFNGPWFIQTIATLGDGSDIEFS

PKEGNGVNVEDDGHSKDLD*

>Solyc12g017300.1.1|Solanum_lycopersicum|M-type_MADS|Solyc12g017300.1.1

MATKRLRDSRNYSENVRNSILDRREISLFKKAEELSILCDVEAAIIIFRPGKIQPIAWKSASLAQDVLTRYLSFLEFKRLDKLVTHEDYLQKLVDKKEEQ

ITKLQKMNEMEESFNDPWFIQSIATLGDGSGIESTPKEGNGVNVEYDGHSKDLD*

>Solyc12g087820.1.1|Solanum_lycopersicum|M-type_MADS|Solyc12g087820.1.1

MGRRKVEIKRIQDKNCRQVAFCKRRKGLLKKAKEISVLCDVDVAVVIISNRGRLHEFSSNNRTSTPCWLDKISPMHQVELITLGSSTLGGTMCALLQVTR

RALPTF*

>Solyc12g088080.1.1|Solanum_lycopersicum|M-type_MADS|Solyc12g088080.1.1

MGRGKILIRRIDNSTSRQVTFSKRRNGLLKKAKELAILCDAEVGVIIFSSTSKLYDYANTRFSPPISHTFFACQMLELQFIFNFLEKNFLRVFSFVY*
